# Elucidation of the Catalytic Apparatus and Mechanism of Human Chitotriosidase‑1

**DOI:** 10.1021/acscatal.5c00507

**Published:** 2025-09-19

**Authors:** Dorota Niedzialek, Grzegorz Wieczorek, Katarzyna Drzewicka, Anna Antosiewicz, Mariusz Milewski, Agnieszka Bartoszewicz, Jacek Olczak, Zbigniew Zasłona

**Affiliations:** † Institute of Biochemistry and Biophysics of the Polish Academy of Sciences, Pawińskiego 5a, 02-106 Warsaw, Poland; ‡ Molecure S.A., Zwirki i Wigury 101, 02-089 Warsaw, Poland; § ASCENTIA sp. z o.o., BRaIn Laboratories of the Medical University of Lodz, Czechosłowacka 4, 92-216 Lodz, Poland

**Keywords:** enzymatic catalysis, chitotriosidase-1, glycoside hydrolases, QM/MM, impact of ions, substrate-assisted catalysis

## Abstract

Despite extensive research over the past three decades, the catalytic mechanism of human chitotriosidase-1 (hCHIT1) has remained incompletely understood. To address this gap, we reanalyzed all available structural information and, integrating experimental data with multiscale molecular simulations, successfully modeled the full-length structure of hCHIT1 for the first time, including the previously unresolved proline-rich linker essential for domain communication. This comprehensive model enabled us to propose a general mechanism of hCHIT1 catalysis and to elucidate the distinct functional roles of all four highly conserved structural motifs of the glycoside hydrolase 18 (GH18) family, a group comprising over 65,000 known members across all domains of life. We further investigated the influence of monovalent metal ions in achieving optimal catalytic conditions and determined the activation energies for both substrate-assisted hydrolysis and transglycosylation processes. Our simulations revealed coordinated Brownian conformational fluctuations within hCHIT1 subdomains, which collectively harness thermal energy to drive catalysis. Notably, we discovered a previously unreported piston-like mechanism in which a conserved tyrosine residue transduces mechanical energy to the substrate, significantly lowering the activation barrier for catalysis. Additionally, by constructing a complete substrate model, we resolved the long-standing mechanistic enigma of the highly conserved tryptophan ‘lid’ at the active site entrance, demonstrating its multifaceted role in substrate gating, transition state stabilization, and product release. Finally, we demonstrated that binding of the first-in-class inhibitor OATD-01 induces subtle yet far-reaching dynamical changes within the active site, leading to dissociation of the immunoglobulin-like heterodimer and disruption of interactions with biological partners implicated in disease pathogenesis. These findings not only redefine the mechanistic landscape of hCHIT1 but also provide a robust framework for the rational design of next-generation GH18 inhibitors, for example, targeting multidrug-resistant pathogens.

## Graphical abstract

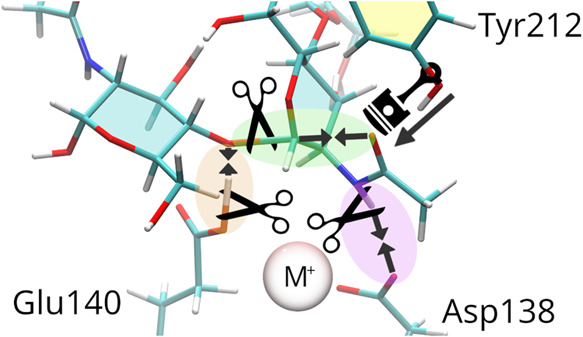

## Introduction

Humans neither produce chitin nor use it as a nutrient source, yet the human genome encodes several chitinases, enzymes from the glycoside hydrolase family 18 (GH18) that cleave glycosidic bonds in GlcNAc-containing glycans (Figure 1).^1,2^ GlcNAc is a widely occurring substance in nature that serves as a crucial structural sugar in various organisms. It is found in bacterial peptidoglycan and fungal chitin cell walls. GlcNAc-containing oligosaccharides are present in the extracellular matrix of animal cells, where they decorate glycosylated proteins and glycolipids. Chitinases are expressed in a wide range of organisms, from prokaryotes to eukaryotes. Humans produce three enzymatically active GH18 chitinases, Di-*N*-acetylchitobiase (**hDIAC**), chitotriosidase-1 (**hCHIT1**), and acidic mammalian chitinase (**hAMCase**) (Figure S1). The rest are chitinase-like proteins which, due to mutations of the catalytic residues, bind but do not catalyze glycans.^3^

**Figure 1:**
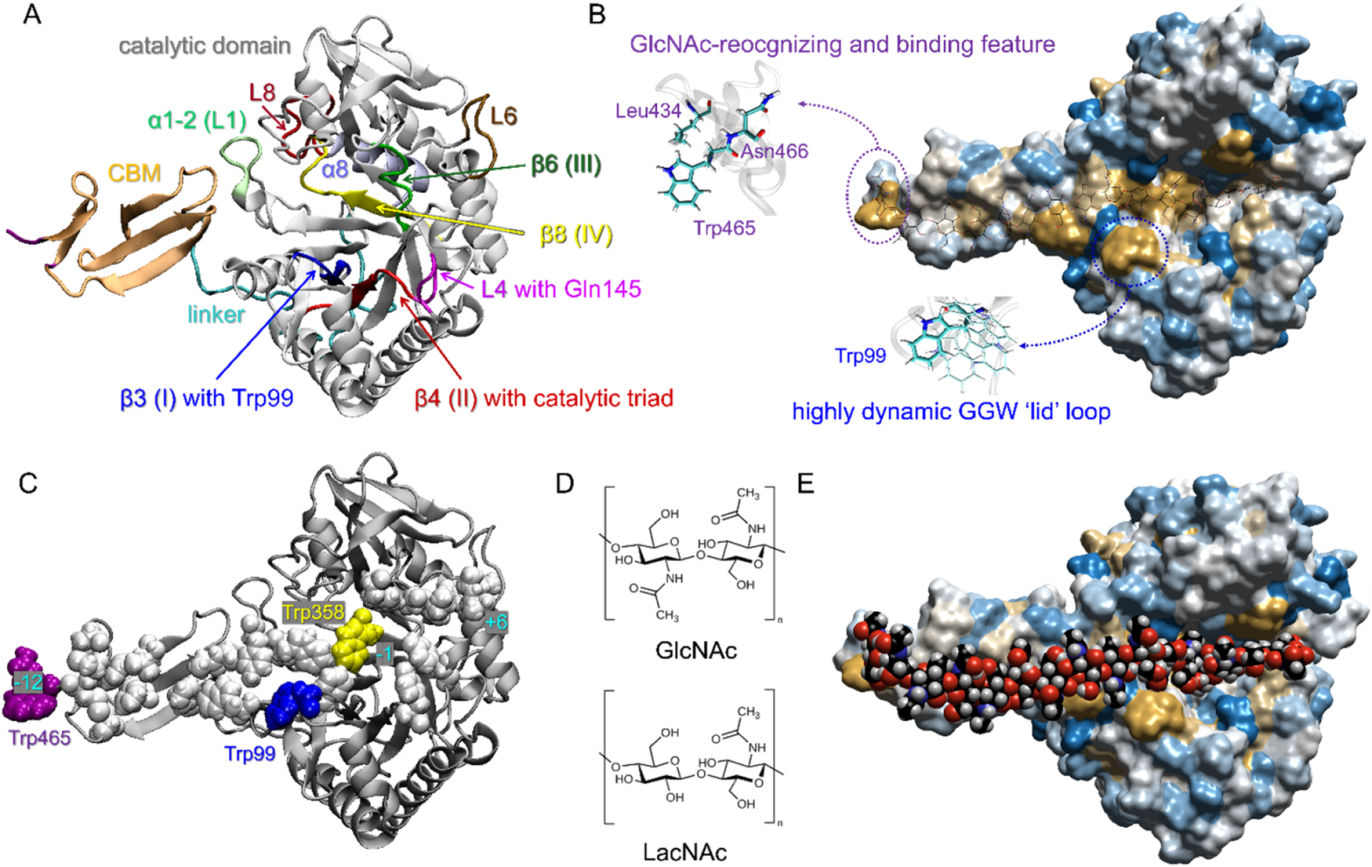
Model of full-length hCHIT1. (A) Cartoon representation of the 50 kDa hCHIT1 heterodimer. The distorted β-sandwich fold of the carbohydrate-binding module (CBM, residues 420–466 marked in orange, PDB ID: 6SO0) is attached to the catalytic domain by a proline-rich linker (residues 388–419 marked in cyan) and topped by the GlcNAc-recognizing feature (residues Trp465 and Asn466 marked in purple). The conserved motifs I (residues 90–99 within the β3 strand), II (residues 132–142 within β4 strand), III (residues 210–216 within β6 strand), and IV (residues 354–364 within β8 strand) are marked in blue, red, green, and yellow, respectively. The L4 loop, which extends from motif II, is marked in magenta. The roles of the remaining loops (L1, L6, and L8) are explained in Figure 5. (B) Two highly conserved tryptophan-containing structural motifs in full-length hCHIT1. The surface representation of the hCHIT1 model colored according to hydrophobic/hydrophilic (yellow/blue) character of amino acids. (C) Cartoon representation of the full-length hCHIT1 model with the sugar-binding hydrophobic residues in the vdW representations. Note the solvent-exposed aromatic residues arranged linearly at a distance equivalent to the spacing between the (GlcNAc)_2_ units. (D) The substrates of chitinases: GlcNAc and GlcNAc-containing LacNAc. (E) The same representation as in (B), with a docked model of the (GlcNAc)_8_ substrate. GlcNAc oligosaccharide in the vdW representation occupy the sugar-binding subsites, ranging from −12 to +4, where −n and +n indicate the nonreducing and reducing end, respectively (i.e., the substrate and product occupying subunits). Schematic representation of the enzymatic process of hCHIT1 is presented in Figure 3.

The ability to degrade chitin suggests a primordial role of hCHIT1 in innate defense mechanisms against GlcNAc-producing pathogens.^4^ However, the role of hDIAC in the degradation of glycoproteins by hydrolyzing the glycosidic bond in the (GlnNAc)_2_ core of asparagine-linked glycans, advocates additional roles for human chitinases, for example, through involvement in protein glycosylation.^5^ As highlighted in Supplementary Discussion 1, extensive evidence indicates that hCHIT1 demonstrates distinct pathogenic roles,^6–10^ which vary depending on the associated diseases^11–17^ and the specific cell types activated during immune responses.^18–23^

hCHIT1 was the first mammalian chitinase discovered and characterized by Aerts and co-workers 30 years ago.^24^ The full-length, 50 kDa hCHIT1 isoform consists of the 39 kDa catalytic domain located at the N-terminus and the carbohydrate-binding module (**CBM**), connected by a 31-amino-acid-long linker (Figure 1A). The 50 kDa hCHIT1 is initially produced by macrophages and stays dominant in the bloodstream. The 39 kDa hCHIT1 is usually cleaved post-translationally in the lysosome of macrophages or, less commonly, formed through differential RNA processing. Both isoforms can act as exochitinases that hydrolyze (GlcNAc)_3_ substrate, which is used in biological assays to evaluate enzymatic activity of hCHIT1 (Figure S2A). The experimental studies demonstrated that CBM is responsible for the high affinity of the full-length hCHIT1 toward GlcNAc-containing polysaccharides.^25–27^ The GlcNAc identification and binding mechanism relies on a platform-like feature composed of adjacent tryptophan and asparagine that were shown to specifically bind acetylated glycans (Figure 1B).^28,29^

The catalytic domain of hCHIT1 exhibits the typical TIM-barrel fold, featuring four regions within strands β3, β4, β6, and β8 that are highly conserved across all GH18 enzymes (Figure S1). The site-directed mutagenesis identified the catalytic triad within motif II, while the roles of the other three conserved motifs remain unidentified.^30^ The catalytic domain of hCHIT1 contains a long and deep substrate-binding cleft, which contains several solvent-exposed aromatic residues arranged in a linear fashion (Figure 1C). These residues continue along the CBM and facilitate the efficient binding of polysaccharide substrates through CH−π interactions (Figure 1B,E).

A substantial number of possible contacts between GlcNAc substrate and the active site cleft provides strong binding that forces a distorted conformation of the −1 sugar and brings the susceptible glycosidic bond (i.e., between −1 and +1 GlcNAc units) in proximity to the proton donor (Figure S3A,B). The architecture of the the active site in hCHIT1 resembles one from the most studied example of the GH18 family—Chitinase A from*Serratia marcescens* (**SmChiA**)—suggesting a common mechanism for hydrolyzing GlcNAc from the reducing end (Figure S4A–C).^31–33^ As both enzymes are classified as ‘bacterial-type’ chitinases,^1^ we expected the full-length hCHIT1 to exhibit an analogous heterodimeric, immunoglobulin-like architecture. However, unlike SmChiA—which features rigidly connected catalytic and carbohydrate-binding domains by a folded linker—the hCHIT1 heterodimer exhibits ‘elbow-bending’ mobility due to its proline-rich linker, which resists folding by maintaining structural integrity (Figure S4D).^34^

Despite the wealth of crystallographic data for hCHIT1 collected during the last two decades, crystal structures of neither the full-length isoform of the enzyme nor a complex of its catalytic domain with a substrate have been solved to this day.^35^ The lack of such critical structural information for hCHIT1 allowed for numerous interpretations of the existing experimental data regarding possible enzymatic mechanisms.^1,36^ Furthermore, conformational changes that are relevant to catalytic mechanisms are difficult to determine from static crystal structures alone. For example, despite the high level of conservation of tryptophan at the entrance of the active site, which indicates its significance within the catalytic apparatus of chitinases across species, its precise role remains unexplored.

The objective of this study was to expand upon the existing understanding of the catalytic apparatus of hCHIT1 and fully comprehend the molecular mechanism of its inhibition by the *first-in-class* inhibitor OATD-01, which is currently undergoing phase 2 clinical trials for the treatment of sarcoidosis.^37–39^ To unravel the enzymatic mechanism of hCHIT1, we modeled the full-length enzyme based on the available structural information and experimental data obtained during our preclinical studies on hCHIT1 inhibitors. Our comprehensive investigations combined classical molecular dynamics (MD) and quantum mechanics/molecular mechanics (QM/MM) simulations of hCHIT1 and its homologues with experimental assays conducted under varying temperatures and ionic conditions. We elucidated the catalytic roles of all four conserved motifs of the GH18 family, examined the influence of the most prevalent cations in the body on its catalytic activity, and determined the activation energy for hCHIT1. Finally, we demonstrated that, by blocking individual components of the hCHIT1 catalytic apparatus, the OATD-01 inhibitor not only inactivates the enzyme but also acts as an allosteric inhibitor of its interactions with biological partners, which are involved in pathological immune responses in certain diseases (Figure S2E).

## Results and Discussion

The first observation from classical MD simulations of unliganded hCHIT1 was the significant mobility of the Trp99 side chain, located at the entrance of the active site on a polyglycine loop (GGW) within β3 strand. This side chain alternately covered and uncovered the active site (Figure 1B). The cycle of opening and closing the active site by the Trp99 ‘lid’ was completed within a few nanoseconds, which is an order of magnitude faster than other local movements observed during the simulations. Furthermore, we observed that the enzymatic activity of hCHIT1 proceeds through multiple successive stages, marked by the sequential progression of distinct conformations arising from thermal fluctuations within specific subdomains of the enzyme (Figure 2A). In the initial, inactive state, the catalytic triad (comprising residues Asp136, Asp138, and Glu140 within the β4 strand) remains concealed within the active site cleft. This state persists until residues Leu135, Leu137, and Trp139, located within the hydrophobic core beneath the catalytic triad, begin to rotate through various rotamers and shift by exchanging vdW contacts with other residues within the hydrophobic core of the enzyme (Figure 2C). The sliding movement of the β4 strand is triggered by Brownian conformational fluctuations in the adjacent L4 loop (Figure 1A). The slide of the β4 strand induces significant conformational changes in its main chain, exposing the catalytic residues to the bulk solvent. The final step in hCHIT1 activation, prior to substrate binding, involves the rotation of the central residue of the catalytic triad (i.e., Asp138) (Figure 2B). The rotation of the central asparagine is closely linked to the rotation of the adjacent Met210, which is located within β6 strand. This process is driven by electrostatic interactions with cations that migrate into the active site from the bulk solvent, guided by the so-called ‘torch of guidance’ effect (Figure S5A).^40^ Strong electrostatic attraction within the active site not only draws in cations but also facilitates the binding of the GlcNAc substrate and stabilizes the positively charged intermediate state (i.e., oxazolinium). Docking the substrate pushes the β4 strand back into the active site cleft. Further accommodation of the substrate involves the formation of numerous strong interactions with residues spanning the −2 to +1 subsites of the binding cleft. These interactions induce a distortion in the −1 sugar, shifting it from the default *chair* to an approximately 30 kJ/mol less energetically favorable *twisted boat* configuration, thereby allowing its 2-acetamido group to position above the Asp138 side chain.

**Figure 2:**
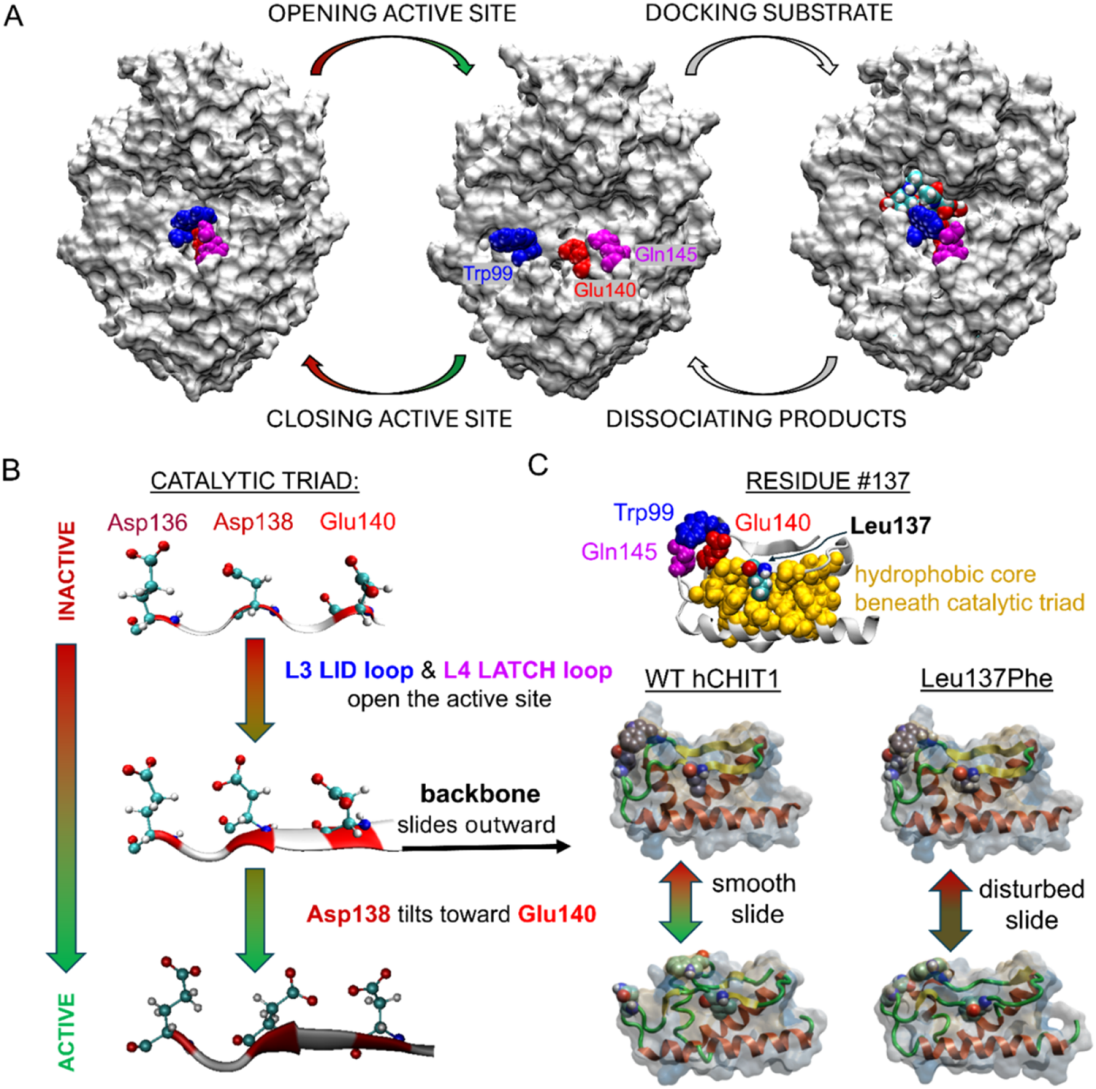
Multiscale activation of the hCHIT1 enzyme. (A) The three main conformations adopted by the hCHIT1 catalytic domain during the catalytic cycle. The enzyme is represented as a white surface. The key residues Trp99 (‘lid’), Glu140 (i.e., proton donor), and Gln145 (‘latch’) as well as the (GlcNAc)_3_ substrate are in the vdW representations, colored in blue, red, magenta, and according to the atom types, respectively. (B) After opening the active site by ‘lid’ and ‘latch’ motifs, the main chain of the sliding β4 strand extends, while the side chain of Asp138 rotates toward Glu140, adopting an optimal position to bind the 2-acetamido group of the −1 sugar. (C) The lateral sections through the regions surrounding the β4 strands in hCHIT1 and its L137F mutant, both subjected to 300 ns MD simulations. The β4 strand of the WT enzyme exhibits uniform sliding behavior on adjacent hydrophobic surfaces, undergoing extensive conformational changes that result in the exposure of the active site toward the bulk aqueous solution prior to substrate binding. The Trp99, Gln145, and Leu/Phe137 residues are in vdW representations while the rest is represented as cartoons and surfaces, colored according to the hydrophobic/hydrophilic (yellow/blue) character of the amino acids.

To better understand the nature of interactions between the substrate and the active site, we conducted a series of steered QM/MM MD simulations of the hCHIT1–(GlcNAc)_3_ model system. We observed that the GGW loop closes the active site with the Trp99 ‘lid’ upon substrate binding. Furthermore, the tryptophan ‘lid’ gets tightened by the 2-acetamido group of the +1 sugar and thereby the substrate locks itself in the active site from the aqueous environment (Figure 2A). This strategy protects the unstable enzymatic reaction intermediate from decomposition by bulk water. The first transition state formation is driven by the Brownian fluctuations within the β6 domain of hCHIT1, with the Tyr212 side chain functioning as a piston. By forming a hydrogen bond with the carbonyl oxygen of the −1 sugar, Tyr212 facilitates the movement of oxygen toward the anomeric carbon, overcoming the activation energy barrier of approximately 60 kJ/mol. This process proceeds through the transition state (TS1 in Figure 3) to form oxazolinium (ox1 in Figure 3), which then donates its hydrogen to Asp138, resulting in the formation of the more stable oxazoline intermediate state (OXA in Figure 3). The oxazolinium ion coexists with oxazoline, and hCHIT1 demonstrates the ability to transition between these two states with remarkable ease. Notably, oxazolinium is observed as a transient intermediate (ox2 in Figure 3) in the pathway leading from oxazoline to the formation of the reaction products (P in Figure 3).^41^ This coexistence results from the oscillatory movement of the proton between the nitrogen atom of the 2-acetamido group and Asp138, as evidenced by QM/MM simulations (cs5c00507_si_005.avi). Following the first catalytic stage, the +1 sugar exits the binding cleft, thereby loosening the Trp99 side chain. This triggers the reopening of the tryptophan ‘lid’, initiating the second stage of catalysis, where bulk water enters the active site and initiates a nucleophilic attack on the C1 anomeric center (TS2 in Figure 3), ultimately completing the hydrolysis. Alternatively, if another oligosaccharide binds to the +n sugar-binding subsites of the binding cleft, then the transglycosylation process can proceed. In both cases, the formation of a new bond with the anomeric carbon leads to the opening of the oxazolinium ring. The 2-acetamido group elongates by approximately 0.8 Å during the ring-opening process, causing the −1 sugar to detach from the active site. After product release, the active site residues rearrange to their initial conformations (resting state in Figure 3), completing the catalytic cycle.

**Figure 3:**
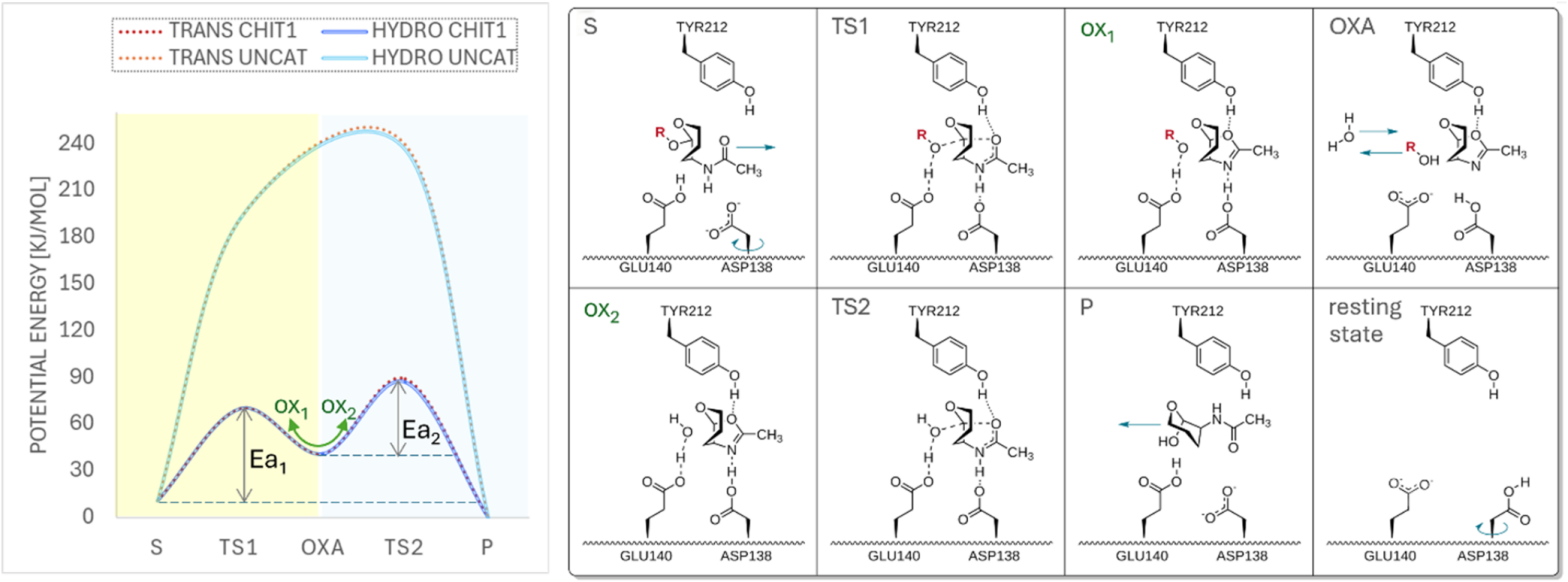
Enzymatic pathway of substrate-assisted catalysis in hCHIT1 as elucidated through QM/MM simulations. The obtained potential energy curves of uncatalyzed (UNCAT) and catalyzed by hCHIT1 (CHIT1) hydrolysis (HYDRO) and transglycosylation (TRANS) processes show the effect of hCHIT1 enzymatic machinery on the activation energy Ea [kJ/mol]. As shown in the potential energy diagram, unlike uncatalyzed single-step processes, the enzymatic pathways for hydrolysis and transglycosylation involve a two-step mechanism (marked in yellow and blue). The catalytic mechanism features two transition states (TS1 and TS2) and an intermediate species, oxazoline (OXA), represented by the valley between the two transition states. The energy Ea_1_ is required to reach the transition state TS1 by driving the carbonyl oxygen toward the anomeric carbon.The green arrows illustrate the coexistence of OXA and transient oxazolinium, as observed in QM/MM simulations. During the second stage of catalysis, this oscillation facilitates the transition to TS2 and helps to overcome Ea_2_. Note that the hydrolysis and transglycosylation processes share the same Ea_1_ value (59 kJ/mol), while their Ea_2_ values are nearly identical (about 46 and 48 kJ/mol, respectively). Schematic representation of the enzymatic process of hCHIT1 from the substrate (S), through the first transition state (TS1) → transient oxazolinium (ox1) → oxazoline intermediate state (OXA) → transient oxazolinium (ox2) and the second transition state (TS2) to the product (P). Green arrows indicate the direction of movements of the substrates into (to the left) and products out of (to the right) the active site as well as the rotation of Asp138 side chain, which initiates the activation of the enzyme and its transformation to the resting state.

To validate our simulation approach, we compared the activation energy obtained from QM/MM trajectories (Figure 3) with the energy calculated from the Arrhenius equation, based on the reaction rate of hCHIT1 in experimental assays determined at four temperatures (Figure 4). We calculated the experimentally determined activation energy to be 47 kJ/mol, corresponding to the rate-determining step (Ea_1_), which is approximately 10 kJ/mol lower than the Ea_1_ value estimated from QM/MM trajectories (59 kJ/mol). The enzymatic hydrolysis by SmChiA, another representative of GH18 chitinases, was estimated to require an activation energy of 50–60 kJ/mol, aligning well with our estimations.^42^ The discrepancy in the activation energy values may result from the high standard deviation observed in the reaction rate (*V*_max_) measurements, which introduces a substantial margin of error. Moreover, we observed an increase in the Michaelis constant (*K*_m_) with rising temperatures, suggesting that elevated temperatures diminish the affinity of hCHIT1 for the substrate. These findings indicate that elevated temperatures may trigger conformational changes in hCHIT1, driven by intensified Brownian conformational fluctuations, which diminish its ability to bind to the substrate efficiently. Notably, the activation energies for hydrolysis and transglycosylation differ by merely a few kJ/mol. This observation is consistent with the established and experimentally verified property of hCHIT1, which demonstrates robust transglycosylation activity, irrespective of conventional ‘transglycosylation conditions’.^7^ Our QM calculations indicate that under mildly basic conditions or when the oxazoline intermediate is stabilized (i.e., oxazolinium ion is less favorable), transglycosylation can become slightly more probable than hydrolysis.

**Figure 4:**
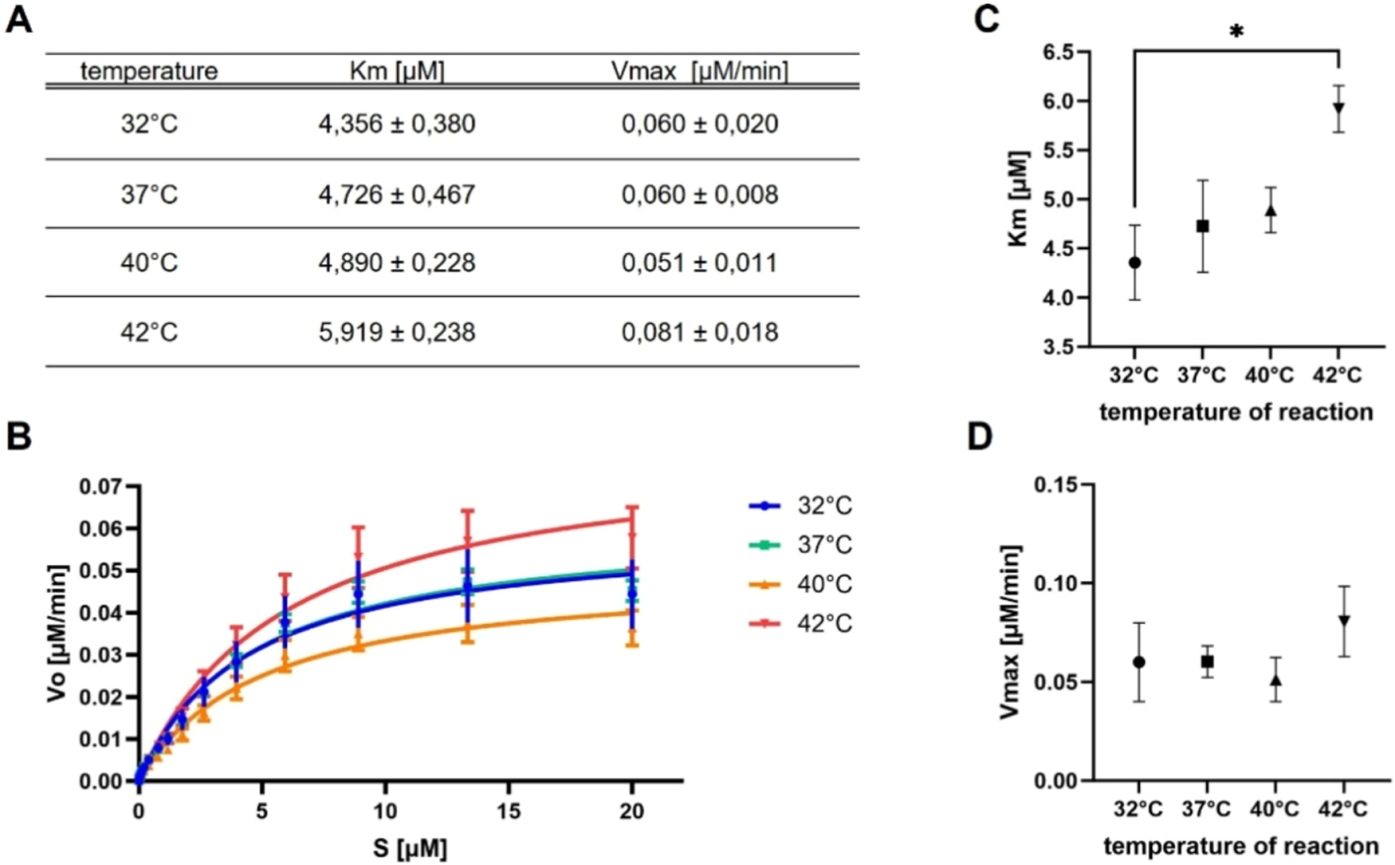
Stable enzymatic activity of hCHIT1 across 32–42 °C. (A) Summary table of *K*_m_ and *V*_max_ values derived from *N* = 3 experiments conducted at four temperature points, including their associated standard deviation values. (B) Michaelis–Menten curves showing hCHIT1 processing of the 4MU-chitotriose substrate at various temperatures. Data represent mean ± SEM from *N* = 3. (C, D) Mean *K*_m_ and *V*_max_ values with standard deviation across different temperatures. Statistical significance between groups is indicated by *­(*p* value of 0.028), assessed using the Kruskal–Wallis test with Dunn’s post hoc test for multiple comparisons.

Noting that electrostatic interactions play a crucial role in facilitating substrate binding, we next examined the impact of the most prevalent cations in the body on the GlcNAc substrate. QM calculations of GlcNAc–cation systems suggest that sodium, potassium, calcium, and magnesium ions have high affinities for interacting with the amide group in agreement with previous experimental studies (Figure S5E).^43^

The alkali metal ions (**M** ^**+**^) polarize the entire GlcNAc molecule resulting in changes to the electrostatic potential charges distribution and the C2–N2 torsional potential energy profile (Figure S6). In the *twisted boat* conformation of GlcNAc, the 2-acetamido group can adopt only a *trans* conformation with the global minimum energy of C2–N2 torsion at 193°. Upon polarization by M^+^, the 2-acetamido group adopts the *cis* orientation in the *twisted boat* conformation with the global minimum of the C2–N2 torsion at the angle optimal for the substrate-assisted GlcNAc hydrolysis. This result indicates involvement of alkali metal ions in achieving an optimal precatalytic conformation of the substrate.

In all the classical MD simulations we conducted, the M^+^ ion eventually settled between the side chains of residues Asp138, Met210, and Trp358 after diffusing through the active site. These residues represent the conserved motifs surrounding the positively charged oxazolinium formed during the catalytic reaction. The presence of M^+^ facilitates the reorientation of these side chains, ensuring their optimal positioning for the substrate binding and proper accommodation of the 2-acetamido group of the −1 sugar. In this way, the entropic penalty of ordering the active site residues to create the hCHIT1-glycan complex is paid by the previously bound cation.

We further analyzed the behavior of monovalent metal ions within the QM/MM trajectories, focusing on their role in modulating the p*K*_a_ value and thereby facilitating the protonation cycling process. The findings from observations on how M^+^ facilitates proton transfer during the catalytic process are discussed in Supplementary Discussion 2 and are presented schematically in Figure S7.^43,44^ The QM/MM results suggest a possible contribution of M^+^ and Met210 in regulating the conjugated protonation cycling of Asp138 and Glu140 residues during the catalytic reaction.^44–46^ However, the p*K*_a_ shift due to alkali metal ions polarization is expected to be rather small and transient, as confirmed by our experiments conducted under varying ionic conditions (Figure S8). The addition of sodium/potassium ions to the biological assay resulted in higher Michaelis constant values without a higher catalytic activity proportional to the cation concentrations, suggesting a cofactor-like involvement of alkali metal ions in the catalytic mechanism of hCHIT1.^47^ The addition of calcium and magnesium ions had inhibitory effects on hCHIT1. This can be explained by the high affinity of divalent metal ions (**M** ^**2+**^) to the carboxyl groups of the catalytic triad and by the lower ligand exchange rates of M^2+^ compared to M^+^. Previous studies have suggested that the conserved tryptophan between the catalytic aspartate and glutamate (i.e., Trp139) is responsible for the ionization of catalytic residues and the formation of the transition state.^48^ It is possible that alkali metal ions occupying the nearby region contribute to this ionization effect. However, the high ionic mobility, low dehydration energy, and small charge/size ratio of potassium/sodium ions contribute to their rapid association/dissociation kinetics and transient polarization. Consequently, the effects of M^+^ on the catalytic activity of hCHIT1 are not discernible in our experimental assays.

Our multiscale simulations of hCHIT1 revealed that residues from all four conserved motifs exhibit distinct dynamic behaviors and mechanical properties that collectively facilitate substrate-assisted catalysis. To achieve a deeper understanding of the enzymatic apparatus of hCHIT1, we conducted comparative computational studies on three closely related homologues, presented in Figure S1. These homologues differ from hCHIT1 by single mutations at specific structural sites of the enzyme that are crucial at various stages of catalysis, thereby exhibiting distinct enzymatic activities.

Motif I incorporates the diffusion-driven GGW loop, capped by the Trp99 ‘lid’, which perpetually opens and closes the active site of hCHIT1. Therefore, Trp99 plays a pivotal role in catalysis by facilitating enzyme activation, enabling substrate binding and accommodation within the active site, protecting the unstable intermediate state from bulk solvent, and promoting product dissociation from the binding cleft after each catalytic cycle. The critical importance of this structural feature is highlighted by its high degree of conservation in chitinases across diverse organisms.

Our MD simulations at pH 7.5 demonstrated that the ‘lid’ remains closed for a significantly longer duration in mAMCase compared to hCHIT1. This observation aligns with previously reported relative catalytic activities under identical conditions, where mAMCase exhibited lower activity than hCHIT1.^49^ This discrepancy in the activation rate can be explained by a different chemical character of the residue 145 within the L4 loop emerging from the β4 strand (Figure 1A), which additionally locks the tryptophan ‘lid’ upon closing the active site. In hCHIT1, this noncovalent ‘latch’ residue is a neutral Gln145 (Figure 2), whereas in mAMCase it is a positively charged Arg145. The energy of tryptophan-asparagine NH−π interaction is weak enough to be affected by solvation effects and thus can be easily overcome in physiological conditions by diffusion forces. At pH 7.5, tryptophan and arginine are connected through a significantly stronger cation−π interaction, making it considerably more stable under physiological conditions.^50^ In the case of hCHIT1, we observed concerted hinge-like movements of the L3 and L4 loops, which serve to expose the active site in preparation for substrate binding.

The dynamics of motifs I and II are intricately interconnected and, as observed in the case of hCHIT1, must be synchronized to ensure efficient catalysis. The synchronized opening and closing of the L3 and L4 loops allow the β4 strand to slide at an optimal rate in and out of the binding cleft, facilitating enzyme activation and subsequently shielding the oxazolinium/oxazoline intermediate from water, thereby ensuring effective catalysis. Only a point mutation of residue 137 within the hydrophobic core, which distinguishes hAMCase enzyme (i.e., the closest homologue of hCHIT1) from its mouse version (mAMCase), significantly alters their relative catalytic activity. The L137F mutation contributes to the lower enzymatic activity of hAMCase compared to mAMCase (i.e., mAMCase, similarly to hCHIT1, possesses leucine while hAMCase phenylalanine, see Figure S1). In the case of the L137F mutant, we observed distortions of the main chain within the catalytic triad, which resulted in a disruption of the β4 strand sliding motion. The decrease in enzymatic activity of the L137F mutant compared to the WT enzyme is attributed to an increased disorder in the β4 strand, characterized by a reduction in its β-sheet character, as observed in the activated L137F mutant enzyme (Figure 2C). The role of hydrophobic amino acids in the core of chitinases, as observed in our study, is consistent with the previously noted trend that mutating amino acids in the hydrophobic core to hydrophilic ones results in the deactivation of chitinases.^51^ Another position where a point mutation in, otherwise conserved, motif II occurs is residue 141. In hCHIT1, Tyr141 forms a hydrogen bond with deprotonated Glu140 that completes the active site closure (Figure S7). The significant decrease in enzymatic activity of mCHIT1 compared with hCHIT1, observed at high substrate concentrations, results from the combined effect of the Y141F and S144G mutations within the L4 loop (Figure S1). Phenylalanine prevents the formation of a hydrogen bond with Glu140, while glycine increases the flexibility of the L4 loop. The resulting structural changes enhance the flexibility of the L4 loop and diminish the effectiveness of active site closure following each catalytic cycle, collectively amplifying the Brownian conformational fluctuations of the L4 loop. Consequently, the motions of the L4 loop that open the active site in mCHIT1 occur too quickly to align with the movement of the L3 loop, thereby failing to sustain efficient catalysis. An excess of catalytically required motions is a well-documented cause of substrate inhibition in enzymes, a phenomenon also observed in mCHIT1 assays.^52^ In contrast, the catalytic machinery of hCHIT1 maintains a high catalytic activity even in the presence of excess substrate. This interpretation aligns with the conclusions drawn from the temperature–activity relationship we observed for hCHIT1 (Figure 4), indicating that heightened dynamics at elevated temperatures (e.g., 42 °C or higher) impede optimal catalytic function. In conclusion, conserved motif II, in conjunction with motif I, facilitates the mechanical activation of the enzymatic apparatus and contributes to shielding the oxazolinium/oxazoline intermediate from water. Additionally, motif II is exclusively responsible for presenting the active site for substrate binding.

Motif III has two distinct functions: it facilitates the transfer of charge and also contributes mechanically to the catalytic process. Reciprocal proton transfer during the formation and breakdown of the oxazolinium/oxazoline ring is facilitated by electrostatic interactions and dispersion forces between the lone electron pair on the sulfur atom of Met210 and the nitrogen atom of the 2-acetamido group. The side chains of Tyr212 and Asp213 together maintain the −1 sugar in the correct configuration for substrate-assisted catalysis by forming tight hydrogen bonds with the carbonyl oxygen and the alcohol hydroxyl group, respectively. The strong attachment of the −1 sugar enables the Tyr212 side chain to efficiently direct the carbonyl oxygen toward the anomeric carbon, thereby inducing the 2-acetamido group to fold into the intermediate oxazolinium/oxazoline ring. The synchronized dynamics of the loops originating from the first three conserved motifs (L3, L4, and L6 in Figure 5A) can be seen as a comprehensive ‘flywheel’ mechanism in the hCHIT1 apparatus.

**Figure 5:**
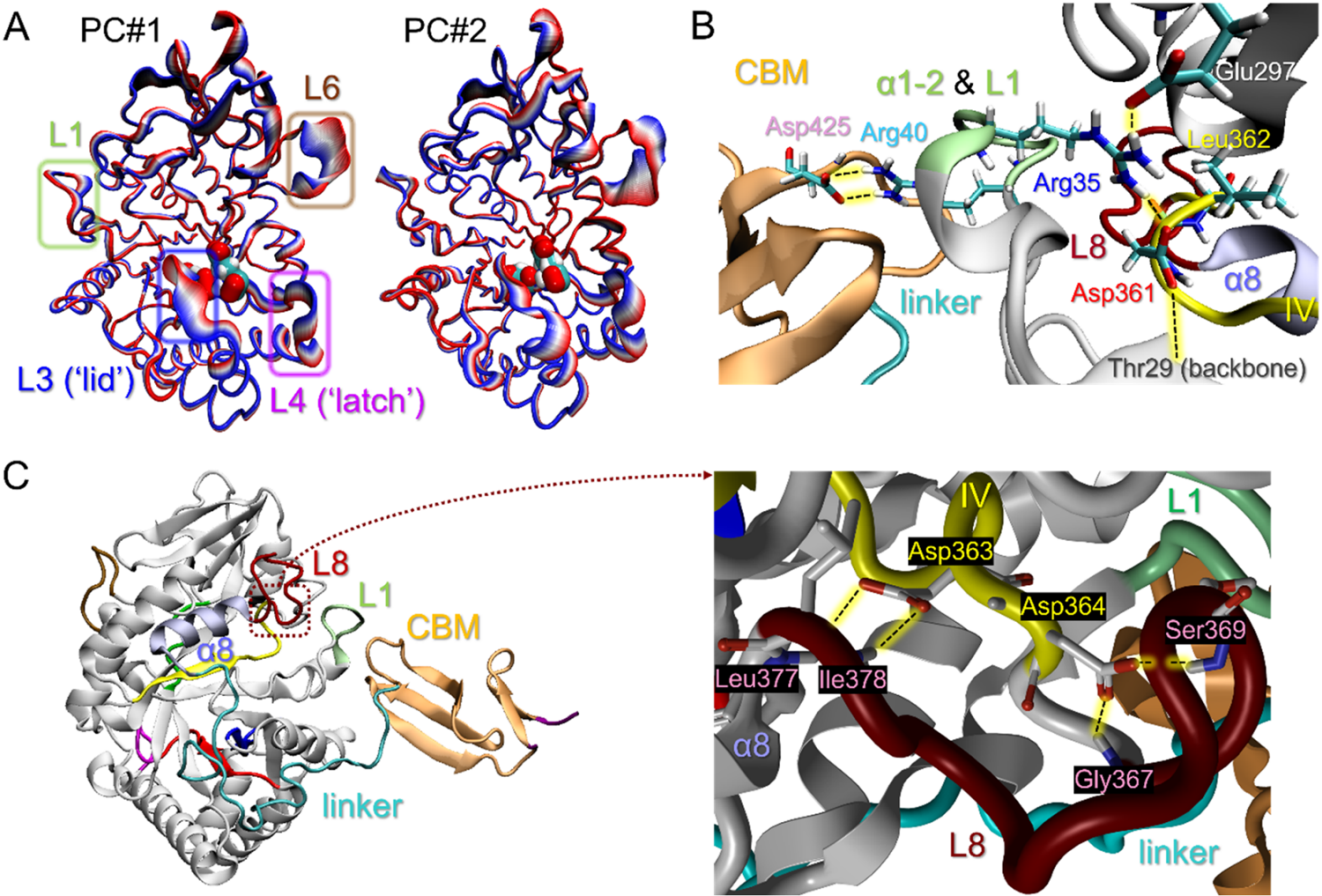
Dynamic features of the catalytic apparatus of hCHIT1. (A) The two first modes of the PC analysis indicate substantial dynamics of the L3, L4, and L6 loops. Note that the L1 loop, which expands from the α1–2 subunit and is involved in docking CBM, also exhibits considerable Brownian conformational fluctuations. The coordinated movement of loops L3 and L4 is required for opening and closing the active site. The substantial movement of the L6 loop triggers the Tyr212-assisted pushing mechanism, facilitating the substrate-assisted GlcNAc catalysis. The enzyme is presented as a cartoon colored in RWB color scale, with the active site residues in vdW representation colored by the atom types. (B) The network of hydrogen bonds (marked as black dotted lines on a yellow background) connecting catalytic and CBM domains in the hCHIT1 heterodimer. The enzyme is in cartoon representation, colored as in Figure 1A. The key residues are in ‘licorice’ representation. Residue Asp361, which belongs to the conserved motif IV, is involved in the molecular ‘wire’ made of two salt bridges (i.e., Asp361–Arg35 and Arg40–Asp425) that promote optimal orientation and flexibility of the L1 loop (colored in lime) for the assembly and stabilization of hCHIT1 heterodimer. (C) Network of hydrogen bonds between conserved residues Asp363 and Asp364 and the L8 loop (dark red), which is adjacent to the α8 helix (violet) that serves as the anchor point of the proline-rich linker. The key residues are in ‘licorice’ representation colored according to the atom types.

Motif IV is the longest conserved region in hCHIT1 and serves multiple purposes. Interestingly, this entire motif is deleted in the most common mutation in the *CHIT1* gene, which causes irreversible inactivation of the enzyme. The QM/MM simulations showed that the Trp358 side chain stabilizes both positively charged oxazolinium and neutral oxazoline intermediates during the enzymatic reaction through cation−π and π–π interactions, respectively. The high mobility observed in MD simulations, coupled with the distinct conformations of the Trp358 side chain between open and closed, unliganded and liganded hCHIT1 crystal structures, indicates a crucial role for this residue in positioning the −1 sugar within the active site and facilitating glycan chain displacement along the binding cleft during hydrolysis and transglycosylation (Figure 1C). The conformational changes of Trp358 are synchronized with the dynamics of the following three conserved aspartic acids (i.e., Asp361, Asp363, and Asp364). These residues, through their interactions with other hCHIT1 subdomains, mediate the mechanical signaling required for the assembly of the catalytic and CBM domains into an immunoglobulin-like heterodimer. They induce structural changes in the proline-rich linker, triggering reciprocal hinge-like movements powered by Brownian conformational fluctuations within the hCHIT1 subdomains, which collectively drive processive catalysis of polysaccharides (Figure S2E). The salt-bridging between Asp361 and Arg35 promotes the correct orientation and optimal flexibility of the α1–2 subunit (i.e., L1 loop) for CBM docking (Figures 5B and S4D). Residue Asp363 establishes hydrogen bonds with the main chain of residues Leu377 and Ile378 on the L8 loop, adjacent to the helix α8, whereas Asp364 forms hydrogen bonds with the main chain of residues Gly367 and Ser369 within the same loop (Figure 5C). These interactions strengthen the anchor point of the proline-rich linker and are therefore directly involved in its ‘elbow-bending’ dynamics. Such attachment of the CBM feature enables hCHIT1 to effectively scan its environment, identify GlcNAc-containing glycans, guide the enzyme to specific regions of polymeric substrates, and carry out processive digestion of crystalline polysaccharides (Figure S2E). In summary, motif IV ensures the precise positioning of the substrate within the binding cleft for substrate-assisted hydrolysis and transglycosylation, stabilizes the intermediate state, and facilitates mechanical communication between the hCHIT1 subdomains, driving the assembly of the catalytic and CBM domains into a functional immunoglobulin-like heterodimer.

The *first-in-class* inhibitor, OATD-01, functions as a three-branched lock, effectively destabilizing the dynamics of the hCHIT1 enzymatic machinery (Figure S3C,D). The first branch, the aminotriazole warhead, exploits M^+^ within the electrostatic substrate attraction mechanism of hCHIT1 to efficiently navigate and anchor the inhibitor within the polarized active site. The second branch, methyl, secures the ‘lid’ loop in a fixed position through CH-π interactions with the Trp99 side chain, while the third branch, 4-chlorobenzene, is connected by a short aliphatic chain. The chlorobenzene moiety participates in hydrophobic and halogen interactions, stabilizing the β8 strand and linking it to the βB1/βB2 subdomains. The short aliphatic chain reinforces the CH–π interactions with the Trp99 side chain, thereby contributing to the immobilization of the ‘lid’ loop (Figure S9). The strong efficacy of OATD-01, establishing it as a *first-in-class* hCHIT1 inhibitor, stems from the synergistic interplay of robust interactions among its three branches and key active site subsections spanning all four conserved hCHIT1 motifs, which together underpin the functionality of the dynamic enzymatic apparatus. As a result, the inhibitor has two distinct and complementary modes of action (Figure S2F). First, it deactivates the catalytic machinery by blocking the active site (Figure S5F). Second, OATD-01 interferes with the mechanical communication between hCHIT1 subdomains by causing structural disturbances in the areas crucial for the integration of the catalytic and CBM domains into a functional immunoglobulin-like heterodimer (Figure S10). The latter effect may result in profound consequences, arising from disrupted cooperativity between immunoglobulin-like hCHIT1 and its biological partners, which are involved in mediating pathological immune responses.

## Conclusions

Building upon both existing and acquired experimental data together with structural analysis and molecular dynamics simulations, we have successfully modeled the full-length structure of hCHIT1 for the first time. Previous structural studies, whether experimental or computational, have been limited to the catalytic domain alone or its complex with the carbohydrate-binding module but without the proline-rich linker. This linker is essential for mediating interactions between the catalytic and CBM domains, and its incorporation in our multiscale molecular dynamics simulations provided new insights into the structural organization and potential regulatory mechanisms of the full-length hCHIT1. Based on the obtained QM/MM results, we proposed a general substrate-assisted catalytic mechanism of hCHIT1 and determined the activation energies for the two-stage, substrate-assisted hydrolysis and transglycosylation processes. We elucidated the catalytic mechanism of hCHIT1 that underlies its strong transglycosylation activity, which remains effective even in the absence of ‘transglycosylation conditions’. Additionally, we identified specific scenarios in which transglycosylation may slightly prevail over hydrolysis. The analysis of enzymatic reaction trajectories obtained from multiscale simulations of hCHIT1 allowed us to identify coordinated conformational changes in the four conserved motifs of GH18 enzymes and, for the first time, comprehensively characterize the functional roles of all these regions within this diverse enzyme family, which comprises over 65,000 members identified across bacteria, fungi, plants, animals, and viruses. These conformational changes were correlated with Brownian fluctuations of enzyme subdomains, collectively driving hCHIT1 activation, facilitating substrate-assisted catalysis, and promoting the assembly of the catalytic and CBM domains into a functional immunoglobulin-like heterodimer. The latter occurs due to hydrogen interactions, which facilitate mechanical signaling from the conserved motif IV to the proline-rich linker, inducing its ’elbow-like’ movements that connect the domains of the heterodimer. We have uncovered the mechanism by which hCHIT1 harnesses collective energy generated by the thermal fluctuations of adjacent subdomains and concentrates this energy on a single tyrosine residue within the conserved motif III. The tyrosine side chain forms a hydrogen bond with the carbonyl oxygen of the 2-acetamido group of the −1 sugar and then, acting like a piston, exerts a mechanical pressure on the oxygen, driving its movement toward the anomeric carbon and advancing the substrate toward the transition state. To the best of our knowledge, this is the first description of such a piston-like mechanism involving a tyrosine residue in enzyme catalysis. This mechanical process effectively reduces the activation energy of the catalytic reaction by approximately 3-fold. The highly conserved tryptophan residue at the active site entrance, despite its evolutionary conservation across species, has long posed a mechanistic puzzle in structural enzymology. Prior studies were unable to determine its role due to the absence of the +1 sugar in experimentally resolved enzyme–substrate complex structures. By constructing a substrate complex model that incorporates this missing sugar within the hCHIT1 catalytic pocket and employing multiscale molecular dynamics simulations, this research has, for the first time, elucidated the multifaceted contributions of this tryptophan residue and its role in specific stages of the catalytic process. This tryptophan acts as a ‘lid’ that continuously opens and closes the active site through diffusion-driven, hinge-like movements of the GGW loop within the conserved motif I. It plays a critical role in the activation of hCHIT1 by facilitating substrate binding and accommodation within the active site, shielding the unstable intermediate states from bulk water, and promoting product dissociation from the binding cleft after each catalytic cycle. Furthermore, we explained the influence of monovalent metal ions on the stabilization of a precatalytic configuration of the active site, achieving an optimal conformation of the substrate, ionization of catalytic residues, and the formation of the transition state. Finally, we demonstrated that, by blocking all four conserved components of the hCHIT1 catalytic apparatus, OATD-01 molecule not only inactivates the enzyme but also causes dissociation of the immunoglobulin-like heterodimer. The described effect leads to the disruption of interactions between hCHIT1 and its biological partners, which play a key role in the pathogenesis of diseases associated with Chitotriosidase-1 overactivity. By demonstrating how the *first-in-class* OATD-01 inhibitor blocks hCHIT1 activity, this research provides mechanistic foundations for designing inhibitors targeting GH18 enzymes such as those present in multidrug-resistant bacterial pathogens like*Serratia marcescens*. Our results pave the way for the development of novel targeted therapies that circumvent multidrug resistance mechanisms and enable reduction in infection-related mortality in high-risk groups (e.g., newborns), where current rates reach 25–58%.

## Methods

### Modeling Dynamics of hCHIT1

Classical MD simulations of the catalytic domain were used for the identification of the key dynamic features within the enzymatic apparatus of hCHIT1. Analysis of the trajectories from MD simulations of hCHIT1 also highlighted the role of ions in this system. To better understand the hCHIT1 results, the same MD simulations were performed for each of the four homologues with known relative activities (Figure S1). The simulated structures were obtained from RCSB Protein Data Bank^53^ or predicted by AlphaFold.^54^ Next, they were visually inspected and structurally analyzed using Coot software.^55^ The Amber ff99sb-ILDN force field^56^ implemented in GROMACS molecular dynamics package^57^ was used for all systems. The TIP4P^58^ water was used to solvate the system, and 0.150 M NaCl was added. After 150,000 steps of minimization using steepest descent^59^ and conjugated gradient^60^ algorithms, 200 ps were run in the NVT ensemble with heating and position restraints on the protein and a further 200 ps of unrestrained NVT equilibration. After removal of the restraints and equilibration of 500,000 steps in the NPT ensemble, production runs of 500 ns were performed with a 1.0 fs time step. The simulation temperature was controlled at 310 K by the V-rescale thermostat,^61^ and the pressure of the system was kept constant at 1 atm using the C-rescale barostat,^62^ in periodic boundary conditions. Both the temperature and pressure were updated every 100 integration steps. Electrostatic interactions were described using the particle mesh Ewald (PME) algorithm.^63^ Nonbonding interactions were calculated within the cutoff of 12 Å. Next, the simulated systems were converted from GROMACS to Tinker format using an in-house script and the simulations were continued with AMOEBA force field^64^ implemented in Tinker 9 software.^65^ The systems were again minimized and gradually heated to 310 K under NVT conditions. After NVT and NpT equilibration runs (1 ns long with a 1 ps time step), production runs of 200 ns were performed. A Nosé–Hoover^66^ thermostat and barostat were used. The trajectories from all MD simulations were visually examined and analyzed using VMD 1.9.4a57 software.^67^ To identify functionally relevant collective motions of hCHIT1, principal component analysis (PCA) of its MD trajectories was done in GROMACS.

### Model Building of hCHIT1 with Substrate

The experimentally resolved structures of catalytically active hCHIT1 contain only −2 and −1 units of the GlcNAc product. Therefore, the missing +1 sugar unit had to be modeled. The human YKL-39 pseudochitinase shows 52% sequence similarity to CHIT1 with a root-mean-square deviation (RMSD) of 1.1 Å on the Cα atomic coordinates after optimal rigid body alignment. Due to the lack of catalytic activity, YKL-39 structures contain GlcNAc oligomers that could be used to model the missing +1 GlcNAc unit in the substrate. The structure of hCHIT1 (PDB ID: 4WKH) with the (GlcNAc)_4_ product was superimposed on the YKL-39 structure (PDB ID: 4P8W) with the (GlcNAc)_4_ with the structural alignment focused on the active site residues and the two same sugar units. The +1 unit from the YKL-39 structure was added to the (GlcNAc)_2_ product to build the model of the (GlcNAc)_3_ substrate. The resulting structure of hCHIT1 with the substrate was subjected to energy minimization with strong torsional restraints imposed on the dihedral angles of the −1 GlcNAc unit to maintain its *twisted boat* conformation. The structural optimization of hCHIT1 with substrate was performed by successive runs of two energy minimization algorithms: 30,000 steepest descent steps and another 30,000 conjugated gradient steps, using Amber03 ^68^ force field and TIP4P explicit water model implemented in GROMACS software. The optimization of the system was followed by a short (5 ns) relaxation run at 310 K, in which the torsional restraints were retained in the −1 sugar.

### QM/MM Calculations of hCHIT1 with Substrate

To observe the substrate-assisted GlcNAc hydrolysis, the system of hCHIT1 with the (GlcNAc)_3_ was divided into regions treated at the quantum (QM) and molecular mechanics (MM) levels of theory. The key active site residues: Asp138, Glu140, Met210, and Tyr212, the ion shared by Asp138 and Glu140, and the (GlcNAc)_3_ substrate was described at the QM DFT using PBE^69^ functional and DZVP-MOLOPT basis set^70^ level, while the rest of the molecular system was treated with AMBER03 force field according to the default QM/MM settings of GROMACS with CP2K software interface.^71^ To test the possible effect of metal ions on the catalytic process of hCHIT1, each of the sodium, potassium, calcium, and magnesium cations was randomly inserted into the system a dozen times, and then the energy of the system was minimized. After minimization, potassium ion tends to migrate to the region of excess density observed in the unliganded hCHIT1 structure, consistent with sodium/potassium ions being incorrectly modeled as water (Figure S5C). This ion was included in the system and described at the quantum level during QM/MM simulations, which were carried out using PBE0 functional^72^ and TZVP-MOLOPT basis set,^70^ truncated Coulomb potential,^73^ long-range Coulomb correction,^74^ Grimme D3 dispersion correction with Becke-Johnson damping^75^ and auxiliary density matrix method.^76^ After fast (10 ps) heating and short (10 ps) equilibration under an NVT ensemble, the system was subjected to several short (40 ps) runs of QM/MM simulations.

PCA indicated that the distinct mechanical motions of the β6 strand observed in the classical MD simulations of hCHIT1 facilitate the substrate-assisted GlcNAc hydrolysis by the Tyr212 side chains pushing the bound carbonyl oxygen of the 2-acetamido group toward the anomeric carbon; an adaptive bias was implemented in the QM/MM MD runs. First, the distance between the carbonyl oxygen and the anomeric carbon (C1) from 3.2 (i.e., in the *cis* conformation of the 2-acetamido group) to 1.43 Å (i.e., the C1–O covalent bond length) was sampled. Second, the distance between the anomeric carbon and the oxygen of the glycosidic bond was changed from the default value of 1.3–4.3 Å. A decrease in the distance between the carbonyl oxygen and the anomeric carbon always corresponded to a spontaneous increase of the distance between the anomeric carbon and the oxygen of the glycosidic bond (and *vice versa*), which eventually led to the formation of the oxazolinium/oxazoline ring from the 2-acetamido group of the −1 sugar and the rupture of the glycosidic bond and dissociation of the +1 product. When exploring the free energy landscape of the substrate-assisted GlcNAc hydrolysis using accelerated weight histogram (AWH) method,^77^ it was impossible to obtain the free energy profile of the simulated process. This suggests that the intermediate state (oxazoline) cannot easily revert to the original reactants.

The analysis of the elemental composition of the coordination sphere and the distances to the nearest atoms from the metal ions was performed using CheckMyMetal server.^78^

### Model Building of the Full-Length hCHIT1

The initial model of the full-length hCHIT1 was based on the 5HBF PDB entry with the crystal structure of the unliganded hCHIT1 catalytic domain cocrystallized with CBM (without the proline-rich linker that connects the heterodimer). That structure had two mutual orientations of the CBM, and catalytic domains were resolved. In chain A, CBM was resolved across from the active site of hCHIT1. In chain B, CBM was found next to the α1–2 subunit, which is structurally closer to the mutual orientation of both domains observed in the immunoglobulin-like Chitinase A from *Serratia marcescens* (SmChiA, PDB ID: 5Z7M), which structurally belongs with hCHIT1 to the same type of ‘bacterial-type’ chitinases. The latter orientation was also proposed by the AlphaFold prediction; therefore, it became the initial conformation of the full-length hCHIT1 (Figure S4B). That initial structure would not allow for the processive catalysis; therefore, we further sampled the structure of the mutual arrangements within heterodimer in two ways. The first approach involved sampling hundreds of poses of CBM docked to the catalytic domain (without considering the linker) using the protein–protein docking method implemented in MOE (Molecular Operating Environment) molecular modeling program.^79^ These heterodimers were generated with and without the routine that addresses the hydrophobic complementarity of the complexed proteins. The 50 top scoring structures of CBM-catalytic domain complexes were selected for visual inspection. Their potential to perform processive hydrolysis of GlcNAc polymers from the reducing end was evaluated with reference to the sugar-binding mode to CBM experimentally determined in ref 26. This ability was further verified by molecular docking simulations of the (GlcNAc)_16_ oligomer to the most promising CBM–catalytic domain heterodimers using default parameters of the Extra Precision docking algorithm implemented in Flare molecular modeling program.^80^ The structure of the full-length hCHIT1 model obtained by this approach had a rich network of noncovalent interactions responsible for the tight binding of CBM to the catalytic domain in the immunoglobulin-like isoform of hCHIT1. The missing linker between the catalytic domain and CBM was manually modeled using the protein editing tools implemented in Flare and the resulting full-length hCHIT1 model was prepared for further simulations (e.g., addition of hydrogens, protonation of histidines, etc.) using Flare’s ProteinPrep protocol. ProteinPrep module was also used to calculate the p*K*_a_ values of the polar amino acids at different stages of the enzyme. The system after a short (200 ns) equilibration under an NPT ensemble was subjected to 500 ns of MD simulation with default settings implemented in Flare (e.g., AMBER ff99SB force field, explicit TIP3P water solvent,^81^ AM1-BCC charges,^82^ PME for treating long-range electrostatics within the cutoff of 12 Å, etc.) except for the temperature (310 K), concentration of NaCl (0.150 M), the time step (1 fs) and hydrogen mass repartitioning option disabled. This simulation allowed the mutual orientation of the catalytic domain, CBM, and the flexible linker that connects them into a heterodimer to be readjusted until the final stable model of the immunoglobulin-like hCHIT1 isoform was reached. The second approach leveraged the structural rigidity conferred by the proline-rich linker of hCHIT1, which resists folding through preservation of conformational integrity. In contrast, SmChiA anchors its carbohydrate-binding module to the catalytic domain via a folded linker domain, resulting in a rigid interdomain architecture. In this case, the initial structure of the full-length hCHIT1 was the starting point of dozens of 100 ns MD simulations performed at room temperature until the most stable model of the immunoglobulin-like hCHIT1 was obtained with a mutual orientation of the catalytic domain and CBM optimal to perform processive hydrolysis of GlcNAc polymers from the reducing end. After less than 200 ns MD simulation, the final model obtained by the first approach (i.e., protein–protein docking of the catalytic domain and CBM with manually modeled linker) changed to the most stable structure simulated by the second approach (i.e., sampling conformational states of the full-length hCHIT1 structure predicted by AlphaFold), which increased our confidence in the proposed full-length hCHIT1 model. The simulation data analysis and visualization were performed by using Flare. Despite differences in the simulation setup, the results obtained from MD calculations performed by Flare and GROMACS matched.

The structure of full-length hCHIT1 with the (GlcNAc)_3_ substrate was superimposed on the structure with OATD-01 (PDB ID: 6ZE8) with the structural alignment focused on the active site residues. The substrate was replaced by the inhibitor and the hCHIT1–OATD-01 complex, after optimization, underwent MD simulations following the same protocol employed for the unliganded hCHIT1.

The calculations of the dipole moment of hCHIT1 were done using Protein Dipole Moments Server.^83^

### QM (DFT) Calculations

All DFT calculations in this work were performed using Gaussian 16 ^84^ software and visualized GaussView program.^85^ The structure of the −1 GlcNAc unit in its *twisted boat* conformation was extracted from a crystal structure of hCHIT1 with the (GlcNAc)_2_ product (PDB ID: 4WKH) and optimized using B3LYP functional with the dispersion correction added explicitly by Grimme’s method (third order) with Becke–Johnson damping^75^ and 6-311++G­(d,p) basis set.^86^ The obtained global minimum energy *trans* conformation of the 2-acetamido group was the starting point for the potential energy scan of the C2–N2 torsion. The calculations were performed for GlcNAc molecule alone and in the presence of a metal cation (sodium, potassium, calcium or magnesium) in vacuum and in the implicit presence of a solvent using the polarizable continuum model (PCM).^87^ Electrostatic potential charges were calculated according to Merz–Kollman (MK) scheme.^88^ The structure of the OATD-01 inhibitor was extracted from a crystal structure of hCHIT1 with the (GlcNAc)_2_ product (PDB ID: 6ZE8) and optimized following the same method employed for unliganded hCHIT1.

### Measuring Enzymatic Activity in Different Temperatures

hCHIT1 was purified from FreeStyle 293-F cells using the previously described method.^89^ The enzymatic assay was performed in a 96-well plate (Greiner Bio-One, catalog no. 675076) and consisted of two sequential steps: (i) 25 μL of Chit1 at a concentration of 2 × 0.25 nM was added to the wells in the assay buffer (20 mM Tris pH 7.5, 150 mM NaCl, 0.25% DMSO final), and (ii) 25 μL of the fluorescent substrate, 4-methylumbelliferyl-β-d-*N*,*N*′,*N*″-triacetylchitotriose (Sigma-Aldrich, cat# M5639), was added at various concentrations, following a 1.5-fold serial dilution in the assay buffer across 15 concentration points starting from 2 × 20 μM. A control without a substrate was also included. Additionally, a standard curve of the 4-Methylumbelliferyl product was prepared using a 1.5-fold serial dilution in the assay buffer with 15 concentration points, starting from either 5 or 1 μM. Following the final addition, the plate was centrifuged at 250*g* for 5 min. Fluorescence was measured using a TECAN SPARK 10 M Multimode Microplate Reader in kinetic mode with excitation at 355 nm and emission at 460 nm. Measurements were taken every minute at different temperatures: 32, 37, 40, and 42 °C. Data were analyzed using GraphPad Prism version 10.0.0. The concentrations of the fluorescent product over time were determined by interpolation from the standard curve. Initial reaction rates (*V*_o_ [μM/min]) were calculated by simple linear regression, representing the slope of the product concentration increase over time. These rates were plotted against substrate concentrations, and *V*_max_ [μM/min] and *K*_m_ [μM] values were determined by using the Michaelis–Menten model.

### Measuring Enzymatic Activity in Different Ionic Conditions

hCHIT1 was purified from FreeStyle 293-F cells using the previously described method.^89^ Prior to the experiments, the enzyme was desalted with Amicon Ultra 2 mL centrifugal filters (Merck Millipore) to replace the existing buffer with the assay buffer: 20 mM Tris, pH 7.4. The enzymatic assay was conducted in a 96-well plate (Greiner Bio-one, cat# 675076**)** and involved three sequential steps: (i) 15 μL of hCHIT1 at a concentration of 3 × 0.25 nM was added to the wells in assay buffer, (ii) 15 μL of assay buffer containing different salts (NaCl, KCl, MgCl_2_, or CaCl_2_) at concentration of 3 × 10 mM was added to each well and incubated for 15 min, (iii) 15 μL of the fluorescent substrate, 4-methylumbelliferyl-β-d-*N*,*N*′,*N*″-triacetylchitotriose, was introduced at various concentrations, creating a 2-fold serial dilution with 7 concentration points starting from 3 × 25 μM. A control with no substrate was also included. After the final addition, the plate was centrifuged at 250*g* for 5 min. The fluorescence signal was measured with excitation at 355 nm and emission at 460 nm using the kinetic mode of the SpectraMax i3x Multi-Mode Microplate Reader. Measurements were taken every 2 min at RT. Data analysis was performed using GraphPad Prism version 10.0.0, employing simple linear regression to calculate the initial reaction rate (*V*_o_, represented by the slope of the fluorescence increase over time, [RFU/min]). The reaction rates were plotted against the substrate concentrations to determine the *V*_max_ [RFU/min] and *K*_m_ [μM] values using the Michaelis–Menten model.

## Supplementary Material













## Notes

Atomic coordinates of the full-length isoform of hCHIT1 model. (PDF)
Atomic coordinates of hCHIT1 catalytic domain with (GlcNAc)­3 model. (PDF)
Dynamic behavior of the apo hCHIT1 heterodimer during 1000 ns MD simulation. (AVI)
Immediate (within the first 200 ns) destabilizing effect of the OATD-01 inhibitor on the otherwise stable hCHIT1 heterodimer. (AVI)
Substrate-assisted catalysis inside the active site of hCHIT1, as simulated by the QM/MM approach. (AVI)
Roles of human chitinases in biological processes and human diseases, the role of M^+^ in facilitating proton transfer during the catalytic process, Figures S1–S10. (PDF)

## Abbreviations

CBM: carbohydrate-binding module
DFT: density functional theory
GH18: glycoside hydrolase 18
GlcNAc: β-1,4-*N*-acetylglucosamine
LacNAc: *N*-acetylglucosamine
M^+^: monovalent metal ion
M^2+^: divalent metal ion
MD: molecular dynamics
NOD-2: nucleotide-binding oligomerization domain containing 2
PC: principal component
PPR: pattern recognition receptor
RWB: red-white-blue
QM/MM: quantum mechanics/molecular mechanics
TLR9: toll-like receptor 9
vdW: van der Waals
WT: wild-type
