# Supplementary material for: Elucidation of the Catalytic Apparatus and Mechanism of Human Chitotriosidase‑1
Source: ACS Catal. 2025 Sep 19;15(19):16748–61. doi: 10.1021/acscatal.5c00507 (PMC12502790; doi:10.1021/acscatal.5c00507)
Supplement: Supplementary file 1 [file cs5c00507_si_001.pdf]

```
TITLE     Atomic coordinates of the full length isoform of hCHIT1 - model
CRYST1   60.857   67.465   66.703  90.00  90.00  90.00 P 1           1
MODEL        1
ATOM      1  N   ALA A  22      25.940  36.521  51.237  1.00  0.00           N
ATOM      2  H1  ALA A  22      25.869  35.825  50.522  1.00  0.00           H
ATOM      3  H2  ALA A  22      26.878  36.550  51.582  1.00  0.00           H
ATOM      4  H3  ALA A  22      25.316  36.295  51.985  1.00  0.00           H
ATOM      5  CA  ALA A  22      25.582  37.835  50.680  1.00  0.00           C
ATOM      6  HA  ALA A  22      24.644  37.711  50.357  1.00  0.00           H
ATOM      7  CB  ALA A  22      25.695  38.896  51.788  1.00  0.00           C
ATOM      8  HB1 ALA A  22      25.453  39.793  51.417  1.00  0.00           H
ATOM      9  HB2 ALA A  22      25.072  38.665  52.535  1.00  0.00           H
ATOM     10  HB3 ALA A  22      26.634  38.920  52.132  1.00  0.00           H
ATOM     11  C   ALA A  22      26.353  38.252  49.442  1.00  0.00           C
ATOM     12  O   ALA A  22      26.718  39.401  49.231  1.00  0.00           O
ATOM     13  N   LYS A  23      26.573  37.219  48.611  1.00  0.00           N
ATOM     14  H   LYS A  23      26.295  36.298  48.884  1.00  0.00           H
ATOM     15  CA  LYS A  23      27.209  37.424  47.322  1.00  0.00           C
ATOM     16  HA  LYS A  23      27.230  38.414  47.186  1.00  0.00           H
ATOM     17  CB  LYS A  23      28.536  36.707  47.437  1.00  0.00           C
ATOM     18  HB1 LYS A  23      28.396  35.738  47.233  1.00  0.00           H
ATOM     19  HB2 LYS A  23      28.874  36.806  48.373  1.00  0.00           H
ATOM     20  CG  LYS A  23      29.566  37.296  46.453  1.00  0.00           C
ATOM     21  HG1 LYS A  23      29.697  38.261  46.682  1.00  0.00           H
ATOM     22  HG2 LYS A  23      29.190  37.221  45.529  1.00  0.00           H
ATOM     23  CD  LYS A  23      30.942  36.627  46.452  1.00  0.00           C
ATOM     24  HD1 LYS A  23      31.596  37.256  46.032  1.00  0.00           H
ATOM     25  HD2 LYS A  23      30.884  35.792  45.905  1.00  0.00           H
ATOM     26  CE  LYS A  23      31.465  36.253  47.836  1.00  0.00           C
ATOM     27  HE1 LYS A  23      32.267  35.662  47.744  1.00  0.00           H
ATOM     28  HE2 LYS A  23      30.752  35.771  48.346  1.00  0.00           H
ATOM     29  NZ  LYS A  23      31.859  37.437  48.613  1.00  0.00           N
ATOM     30  HZ1 LYS A  23      32.195  37.150  49.510  1.00  0.00           H
ATOM     31  HZ2 LYS A  23      32.582  37.929  48.128  1.00  0.00           H
ATOM     32  HZ3 LYS A  23      31.068  38.038  48.730  1.00  0.00           H
ATOM     33  C   LYS A  23      26.485  36.753  46.137  1.00  0.00           C
ATOM     34  O   LYS A  23      25.866  35.747  46.353  1.00  0.00           O
ATOM     35  N   LEU A  24      26.622  37.325  44.934  1.00  0.00           N
ATOM     36  H   LEU A  24      27.216  38.129  44.890  1.00  0.00           H
ATOM     37  CA  LEU A  24      25.993  36.902  43.658  1.00  0.00           C
ATOM     38  HA  LEU A  24      25.866  35.910  43.644  1.00  0.00           H
ATOM     39  CB  LEU A  24      24.593  37.427  43.517  1.00  0.00           C
ATOM     40  HB1 LEU A  24      24.654  38.403  43.725  1.00  0.00           H
ATOM     41  HB2 LEU A  24      24.064  36.967  44.230  1.00  0.00           H
ATOM     42  CG  LEU A  24      23.768  37.319  42.246  1.00  0.00           C
ATOM     43  HG  LEU A  24      24.322  37.885  41.635  1.00  0.00           H
ATOM     44  CD1 LEU A  24      23.737  35.967  41.592  1.00  0.00           C
ATOM     45 HD11 LEU A  24      23.170  36.008  40.770  1.00  0.00           H
ATOM     46 HD12 LEU A  24      24.667  35.696  41.343  1.00  0.00           H
ATOM     47 HD13 LEU A  24      23.354  35.298  42.229  1.00  0.00           H
ATOM     48  CD2 LEU A  24      22.322  37.897  42.348  1.00  0.00           C
ATOM     49 HD21 LEU A  24      21.855  37.784  41.471  1.00  0.00           H
ATOM     50 HD22 LEU A  24      21.817  37.409  43.060  1.00  0.00           H
ATOM     51 HD23 LEU A  24      22.367  38.869  42.579  1.00  0.00           H
```

ATOM	52	C	LEU	A	24	26.922	37.190	42.540	1.00	0.00	C
ATOM	53	O	LEU	A	24	26.929	38.294	41.884	1.00	0.00	O
ATOM	54	N	VAL	A	25	27.734	36.195	42.118	1.00	0.00	N
ATOM	55	H	VAL	A	25	27.760	35.357	42.663	1.00	0.00	H
ATOM	56	CA	VAL	A	25	28.582	36.252	40.917	1.00	0.00	C
ATOM	57	HA	VAL	A	25	28.882	37.196	40.782	1.00	0.00	H
ATOM	58	CB	VAL	A	25	29.832	35.334	41.142	1.00	0.00	C
ATOM	59	HB	VAL	A	25	29.465	34.409	41.242	1.00	0.00	H
ATOM	60	CG1	VAL	A	25	30.783	35.676	39.987	1.00	0.00	C
ATOM	61	HG11	VAL	A	25	31.613	35.123	40.064	1.00	0.00	H
ATOM	62	HG12	VAL	A	25	30.333	35.482	39.116	1.00	0.00	H
ATOM	63	HG13	VAL	A	25	31.023	36.646	40.029	1.00	0.00	H
ATOM	64	CG2	VAL	A	25	30.687	35.477	42.370	1.00	0.00	C
ATOM	65	HG21	VAL	A	25	31.432	34.810	42.337	1.00	0.00	H
ATOM	66	HG22	VAL	A	25	31.068	36.401	42.405	1.00	0.00	H
ATOM	67	HG23	VAL	A	25	30.130	35.315	43.185	1.00	0.00	H
ATOM	68	C	VAL	A	25	27.755	35.888	39.682	1.00	0.00	C
ATOM	69	O	VAL	A	25	26.812	35.075	39.696	1.00	0.00	O
ATOM	70	N	CYS	A	26	28.048	36.513	38.589	1.00	0.00	N
ATOM	71	H	CYS	A	26	28.724	37.248	38.628	1.00	0.00	H
ATOM	72	CA	CYS	A	26	27.413	36.183	37.248	1.00	0.00	C
ATOM	73	HA	CYS	A	26	27.179	35.211	37.261	1.00	0.00	H
ATOM	74	CB	CYS	A	26	26.205	37.134	37.112	1.00	0.00	C
ATOM	75	HB1	CYS	A	26	25.731	36.808	36.294	1.00	0.00	H
ATOM	76	HB2	CYS	A	26	26.619	38.025	36.924	1.00	0.00	H
ATOM	77	SG	CYS	A	26	25.087	37.241	38.479	1.00	0.00	S
ATOM	78	C	CYS	A	26	28.353	36.429	36.023	1.00	0.00	C
ATOM	79	O	CYS	A	26	29.023	37.443	36.098	1.00	0.00	O
ATOM	80	N	TYR	A	27	28.538	35.422	35.152	1.00	0.00	N
ATOM	81	H	TYR	A	27	27.999	34.590	35.286	1.00	0.00	H
ATOM	82	CA	TYR	A	27	29.475	35.450	34.014	1.00	0.00	C
ATOM	83	HA	TYR	A	27	30.203	36.056	34.334	1.00	0.00	H
ATOM	84	CB	TYR	A	27	29.998	34.035	33.710	1.00	0.00	C
ATOM	85	HB1	TYR	A	27	30.479	34.062	32.834	1.00	0.00	H
ATOM	86	HB2	TYR	A	27	29.212	33.420	33.640	1.00	0.00	H
ATOM	87	CG	TYR	A	27	30.949	33.446	34.736	1.00	0.00	C
ATOM	88	CD1	TYR	A	27	30.464	32.580	35.734	1.00	0.00	C
ATOM	89	HD1	TYR	A	27	29.484	32.391	35.799	1.00	0.00	H
ATOM	90	CE1	TYR	A	27	31.357	31.980	36.639	1.00	0.00	C
ATOM	91	HE1	TYR	A	27	31.009	31.354	37.337	1.00	0.00	H
ATOM	92	CZ	TYR	A	27	32.734	32.252	36.572	1.00	0.00	C
ATOM	93	OH	TYR	A	27	33.559	31.688	37.491	1.00	0.00	O
ATOM	94	HH	TYR	A	27	34.501	31.974	37.313	1.00	0.00	H
ATOM	95	CE2	TYR	A	27	33.224	33.112	35.567	1.00	0.00	C
ATOM	96	HE2	TYR	A	27	34.204	33.304	35.508	1.00	0.00	H
ATOM	97	CD2	TYR	A	27	32.333	33.701	34.646	1.00	0.00	C
ATOM	98	HD2	TYR	A	27	32.684	34.301	33.927	1.00	0.00	H
ATOM	99	C	TYR	A	27	28.861	36.038	32.728	1.00	0.00	C
ATOM	100	O	TYR	A	27	27.763	35.658	32.320	1.00	0.00	O
ATOM	101	N	PHE	A	28	29.614	36.901	32.024	1.00	0.00	N
ATOM	102	H	PHE	A	28	30.448	37.222	32.472	1.00	0.00	H
ATOM	103	CA	PHE	A	28	29.368	37.416	30.713	1.00	0.00	C
ATOM	104	HA	PHE	A	28	28.411	37.299	30.448	1.00	0.00	H
ATOM	105	CB	PHE	A	28	29.719	38.891	30.790	1.00	0.00	C

ATOM	106	HB1	PHE	A	28	30.646	38.984	31.154	1.00	0.00	H
ATOM	107	HB2	PHE	A	28	29.073	39.346	31.403	1.00	0.00	H
ATOM	108	CG	PHE	A	28	29.655	39.555	29.422	1.00	0.00	C
ATOM	109	CD1	PHE	A	28	28.581	40.387	28.983	1.00	0.00	C
ATOM	110	HD1	PHE	A	28	27.804	40.561	29.588	1.00	0.00	H
ATOM	111	CE1	PHE	A	28	28.613	40.940	27.748	1.00	0.00	C
ATOM	112	HE1	PHE	A	28	27.849	41.525	27.475	1.00	0.00	H
ATOM	113	CZ	PHE	A	28	29.650	40.742	26.830	1.00	0.00	C
ATOM	114	HZ	PHE	A	28	29.596	41.098	25.897	1.00	0.00	H
ATOM	115	CE2	PHE	A	28	30.766	40.025	27.273	1.00	0.00	C
ATOM	116	HE2	PHE	A	28	31.561	39.946	26.672	1.00	0.00	H
ATOM	117	CD2	PHE	A	28	30.803	39.429	28.503	1.00	0.00	C
ATOM	118	HD2	PHE	A	28	31.609	38.907	28.780	1.00	0.00	H
ATOM	119	C	PHE	A	28	30.139	36.623	29.703	1.00	0.00	C
ATOM	120	O	PHE	A	28	31.388	36.570	29.688	1.00	0.00	O
ATOM	121	N	THR	A	29	29.476	35.992	28.719	1.00	0.00	N
ATOM	122	H	THR	A	29	28.477	36.030	28.702	1.00	0.00	H
ATOM	123	CA	THR	A	29	30.189	35.240	27.656	1.00	0.00	C
ATOM	124	HA	THR	A	29	31.058	35.012	28.095	1.00	0.00	H
ATOM	125	CB	THR	A	29	29.503	33.918	27.274	1.00	0.00	C
ATOM	126	HB	THR	A	29	30.069	33.480	26.575	1.00	0.00	H
ATOM	127	CG2	THR	A	29	29.295	32.994	28.475	1.00	0.00	C
ATOM	128	HG21	THR	A	29	28.847	32.152	28.174	1.00	0.00	H
ATOM	129	HG22	THR	A	29	30.181	32.773	28.883	1.00	0.00	H
ATOM	130	HG23	THR	A	29	28.721	33.453	29.153	1.00	0.00	H
ATOM	131	OG1	THR	A	29	28.281	34.096	26.610	1.00	0.00	O
ATOM	132	HG1	THR	A	29	27.888	33.203	26.390	1.00	0.00	H
ATOM	133	C	THR	A	29	30.489	36.098	26.420	1.00	0.00	C
ATOM	134	O	THR	A	29	29.586	36.584	25.734	1.00	0.00	O
ATOM	135	N	ASN	A	30	31.769	36.247	26.049	1.00	0.00	N
ATOM	136	H	ASN	A	30	32.479	35.769	26.566	1.00	0.00	H
ATOM	137	CA	ASN	A	30	32.168	37.088	24.910	1.00	0.00	C
ATOM	138	HA	ASN	A	30	31.538	37.859	24.999	1.00	0.00	H
ATOM	139	CB	ASN	A	30	33.613	37.621	25.061	1.00	0.00	C
ATOM	140	HB1	ASN	A	30	33.755	37.868	26.020	1.00	0.00	H
ATOM	141	HB2	ASN	A	30	33.705	38.437	24.490	1.00	0.00	H
ATOM	142	CG	ASN	A	30	34.728	36.662	24.668	1.00	0.00	C
ATOM	143	OD1	ASN	A	30	34.550	35.462	24.561	1.00	0.00	O
ATOM	144	ND2	ASN	A	30	35.932	37.142	24.451	1.00	0.00	N
ATOM	145	HD21	ASN	A	30	36.104	38.123	24.545	1.00	0.00	H
ATOM	146	HD22	ASN	A	30	36.676	36.527	24.192	1.00	0.00	H
ATOM	147	C	ASN	A	30	31.855	36.499	23.516	1.00	0.00	C
ATOM	148	O	ASN	A	30	32.272	37.082	22.524	1.00	0.00	O
ATOM	149	N	TRP	A	31	31.047	35.437	23.405	1.00	0.00	N
ATOM	150	H	TRP	A	31	30.747	34.987	24.246	1.00	0.00	H
ATOM	151	CA	TRP	A	31	30.571	34.889	22.123	1.00	0.00	C
ATOM	152	HA	TRP	A	31	30.983	35.492	21.440	1.00	0.00	H
ATOM	153	CB	TRP	A	31	31.103	33.463	21.919	1.00	0.00	C
ATOM	154	HB1	TRP	A	31	32.101	33.505	21.866	1.00	0.00	H
ATOM	155	HB2	TRP	A	31	30.738	33.112	21.057	1.00	0.00	H
ATOM	156	CG	TRP	A	31	30.762	32.464	22.978	1.00	0.00	C
ATOM	157	CD1	TRP	A	31	29.838	31.484	22.872	1.00	0.00	C
ATOM	158	HD1	TRP	A	31	29.260	31.321	22.072	1.00	0.00	H
ATOM	159	NE1	TRP	A	31	29.812	30.742	24.034	1.00	0.00	N

ATOM	160	HE1	TRP	A	31	29.219	29.951	24.182	1.00	0.00	H
ATOM	161	CE2	TRP	A	31	30.704	31.225	24.964	1.00	0.00	C
ATOM	162	CZ2	TRP	A	31	31.047	30.833	26.262	1.00	0.00	C
ATOM	163	HZ2	TRP	A	31	30.547	30.094	26.713	1.00	0.00	H
ATOM	164	CH2	TRP	A	31	32.100	31.485	26.928	1.00	0.00	C
ATOM	165	HH2	TRP	A	31	32.369	31.196	27.847	1.00	0.00	H
ATOM	166	CZ3	TRP	A	31	32.771	32.544	26.295	1.00	0.00	C
ATOM	167	HZ3	TRP	A	31	33.513	33.018	26.769	1.00	0.00	H
ATOM	168	CE3	TRP	A	31	32.402	32.941	24.996	1.00	0.00	C
ATOM	169	HE3	TRP	A	31	32.890	33.697	24.559	1.00	0.00	H
ATOM	170	CD2	TRP	A	31	31.361	32.298	24.298	1.00	0.00	C
ATOM	171	C	TRP	A	31	29.054	35.022	21.889	1.00	0.00	C
ATOM	172	O	TRP	A	31	28.610	34.853	20.754	1.00	0.00	O
ATOM	173	N	ALA	A	32	28.275	35.513	22.862	1.00	0.00	N
ATOM	174	H	ALA	A	32	28.669	35.616	23.775	1.00	0.00	H
ATOM	175	CA	ALA	A	32	26.868	35.910	22.660	1.00	0.00	C
ATOM	176	HA	ALA	A	32	26.415	35.127	22.234	1.00	0.00	H
ATOM	177	CB	ALA	A	32	26.295	36.236	24.029	1.00	0.00	C
ATOM	178	HB1	ALA	A	32	25.338	36.511	23.933	1.00	0.00	H
ATOM	179	HB2	ALA	A	32	26.353	35.428	24.615	1.00	0.00	H
ATOM	180	HB3	ALA	A	32	26.817	36.984	24.439	1.00	0.00	H
ATOM	181	C	ALA	A	32	26.686	37.109	21.703	1.00	0.00	C
ATOM	182	O	ALA	A	32	25.613	37.233	21.041	1.00	0.00	O
ATOM	183	N	GLN	A	33	27.758	37.900	21.503	1.00	0.00	N
ATOM	184	H	GLN	A	33	28.512	37.860	22.158	1.00	0.00	H
ATOM	185	CA	GLN	A	33	27.865	38.819	20.362	1.00	0.00	C
ATOM	186	HA	GLN	A	33	27.018	39.340	20.462	1.00	0.00	H
ATOM	187	CB	GLN	A	33	29.110	39.713	20.495	1.00	0.00	C
ATOM	188	HB1	GLN	A	33	29.064	40.182	21.377	1.00	0.00	H
ATOM	189	HB2	GLN	A	33	29.089	40.388	19.758	1.00	0.00	H
ATOM	190	CG	GLN	A	33	30.451	38.957	20.419	1.00	0.00	C
ATOM	191	HG1	GLN	A	33	30.573	38.609	19.490	1.00	0.00	H
ATOM	192	HG2	GLN	A	33	30.427	38.191	21.062	1.00	0.00	H
ATOM	193	CD	GLN	A	33	31.647	39.844	20.766	1.00	0.00	C
ATOM	194	OE1	GLN	A	33	32.128	40.625	19.960	1.00	0.00	O
ATOM	195	NE2	GLN	A	33	32.116	39.824	21.997	1.00	0.00	N
ATOM	196	HE21	GLN	A	33	31.690	39.236	22.685	1.00	0.00	H
ATOM	197	HE22	GLN	A	33	32.898	40.397	22.244	1.00	0.00	H
ATOM	198	C	GLN	A	33	27.813	38.176	18.960	1.00	0.00	C
ATOM	199	O	GLN	A	33	27.374	38.822	18.008	1.00	0.00	O
ATOM	200	N	TYR	A	34	28.211	36.910	18.811	1.00	0.00	N
ATOM	201	H	TYR	A	34	28.542	36.421	19.618	1.00	0.00	H
ATOM	202	CA	TYR	A	34	28.189	36.196	17.526	1.00	0.00	C
ATOM	203	HA	TYR	A	34	28.231	36.930	16.848	1.00	0.00	H
ATOM	204	CB	TYR	A	34	29.394	35.269	17.422	1.00	0.00	C
ATOM	205	HB1	TYR	A	34	29.404	34.875	16.503	1.00	0.00	H
ATOM	206	HB2	TYR	A	34	29.283	34.539	18.096	1.00	0.00	H
ATOM	207	CG	TYR	A	34	30.741	35.916	17.661	1.00	0.00	C
ATOM	208	CD1	TYR	A	34	31.195	36.992	16.869	1.00	0.00	C
ATOM	209	HD1	TYR	A	34	30.586	37.409	16.195	1.00	0.00	H
ATOM	210	CE1	TYR	A	34	32.509	37.478	17.030	1.00	0.00	C
ATOM	211	HE1	TYR	A	34	32.837	38.233	16.463	1.00	0.00	H
ATOM	212	CZ	TYR	A	34	33.359	36.895	17.994	1.00	0.00	C
ATOM	213	OH	TYR	A	34	34.665	37.248	18.070	1.00	0.00	O

ATOM	214	HH	TYR	A	34	35.106	36.736	18.807	1.00	0.00	H
ATOM	215	CE2	TYR	A	34	32.865	35.875	18.830	1.00	0.00	C
ATOM	216	HE2	TYR	A	34	33.440	35.510	19.562	1.00	0.00	H
ATOM	217	CD2	TYR	A	34	31.575	35.369	18.643	1.00	0.00	C
ATOM	218	HD2	TYR	A	34	31.248	34.615	19.212	1.00	0.00	H
ATOM	219	C	TYR	A	34	26.904	35.397	17.280	1.00	0.00	C
ATOM	220	O	TYR	A	34	26.682	34.918	16.166	1.00	0.00	O
ATOM	221	N	ARG	A	35	26.024	35.300	18.284	1.00	0.00	N
ATOM	222	H	ARG	A	35	26.340	35.479	19.216	1.00	0.00	H
ATOM	223	CA	ARG	A	35	24.615	34.942	18.078	1.00	0.00	C
ATOM	224	HA	ARG	A	35	24.582	34.096	17.546	1.00	0.00	H
ATOM	225	CB	ARG	A	35	23.934	34.680	19.435	1.00	0.00	C
ATOM	226	HB1	ARG	A	35	22.942	34.646	19.313	1.00	0.00	H
ATOM	227	HB2	ARG	A	35	24.166	35.413	20.075	1.00	0.00	H
ATOM	228	CG	ARG	A	35	24.434	33.333	19.988	1.00	0.00	C
ATOM	229	HG1	ARG	A	35	25.433	33.344	20.031	1.00	0.00	H
ATOM	230	HG2	ARG	A	35	24.135	32.596	19.382	1.00	0.00	H
ATOM	231	CD	ARG	A	35	23.883	33.076	21.386	1.00	0.00	C
ATOM	232	HD1	ARG	A	35	22.942	32.744	21.314	1.00	0.00	H
ATOM	233	HD2	ARG	A	35	23.896	33.929	21.907	1.00	0.00	H
ATOM	234	NE	ARG	A	35	24.687	32.057	22.128	1.00	0.00	N
ATOM	235	HE	ARG	A	35	24.928	31.244	21.597	1.00	0.00	H
ATOM	236	CZ	ARG	A	35	25.135	32.059	23.389	1.00	0.00	C
ATOM	237	NH1	ARG	A	35	24.687	32.842	24.350	1.00	0.00	N
ATOM	238	HH11	ARG	A	35	23.957	33.498	24.161	1.00	0.00	H
ATOM	239	HH12	ARG	A	35	25.078	32.780	25.268	1.00	0.00	H
ATOM	240	NH2	ARG	A	35	26.094	31.240	23.721	1.00	0.00	N
ATOM	241	HH21	ARG	A	35	26.483	30.623	23.037	1.00	0.00	H
ATOM	242	HH22	ARG	A	35	26.440	31.229	24.659	1.00	0.00	H
ATOM	243	C	ARG	A	35	23.934	36.047	17.271	1.00	0.00	C
ATOM	244	O	ARG	A	35	24.435	37.170	17.191	1.00	0.00	O
ATOM	245	N	GLN	A	36	22.826	35.732	16.608	1.00	0.00	N
ATOM	246	H	GLN	A	36	22.454	34.807	16.687	1.00	0.00	H
ATOM	247	CA	GLN	A	36	22.117	36.743	15.738	1.00	0.00	C
ATOM	248	HA	GLN	A	36	22.601	37.618	15.739	1.00	0.00	H
ATOM	249	CB	GLN	A	36	22.038	36.209	14.296	1.00	0.00	C
ATOM	250	HB1	GLN	A	36	21.617	36.916	13.728	1.00	0.00	H
ATOM	251	HB2	GLN	A	36	21.457	35.395	14.300	1.00	0.00	H
ATOM	252	CG	GLN	A	36	23.392	35.828	13.662	1.00	0.00	C
ATOM	253	HG1	GLN	A	36	24.060	36.523	13.927	1.00	0.00	H
ATOM	254	HG2	GLN	A	36	23.278	35.836	12.669	1.00	0.00	H
ATOM	255	CD	GLN	A	36	23.969	34.460	14.050	1.00	0.00	C
ATOM	256	OE1	GLN	A	36	23.370	33.619	14.716	1.00	0.00	O
ATOM	257	NE2	GLN	A	36	25.171	34.172	13.610	1.00	0.00	N
ATOM	258	HE21	GLN	A	36	25.669	34.832	13.048	1.00	0.00	H
ATOM	259	HE22	GLN	A	36	25.589	33.292	13.837	1.00	0.00	H
ATOM	260	C	GLN	A	36	20.801	37.048	16.308	1.00	0.00	C
ATOM	261	O	GLN	A	36	20.141	36.259	16.977	1.00	0.00	O
ATOM	262	N	GLY	A	37	20.450	38.347	16.140	1.00	0.00	N
ATOM	263	H	GLY	A	37	21.146	38.965	15.774	1.00	0.00	H
ATOM	264	CA	GLY	A	37	19.113	38.935	16.454	1.00	0.00	C
ATOM	265	HA1	GLY	A	37	18.427	38.421	15.939	1.00	0.00	H
ATOM	266	HA2	GLY	A	37	19.119	39.886	16.145	1.00	0.00	H
ATOM	267	C	GLY	A	37	18.762	38.889	17.963	1.00	0.00	C

ATOM	268	O	GLY	A	37	19.512	39.338	18.856	1.00	0.00	O
ATOM	269	N	GLU	A	38	17.590	38.277	18.307	1.00	0.00	N
ATOM	270	H	GLU	A	38	17.101	37.787	17.585	1.00	0.00	H
ATOM	271	CA	GLU	A	38	17.006	38.277	19.605	1.00	0.00	C
ATOM	272	HA	GLU	A	38	16.913	39.254	19.795	1.00	0.00	H
ATOM	273	CB	GLU	A	38	15.698	37.525	19.500	1.00	0.00	C
ATOM	274	HB1	GLU	A	38	15.314	37.522	20.423	1.00	0.00	H
ATOM	275	HB2	GLU	A	38	15.944	36.592	19.238	1.00	0.00	H
ATOM	276	CG	GLU	A	38	14.567	37.975	18.546	1.00	0.00	C
ATOM	277	HG1	GLU	A	38	13.709	37.561	18.850	1.00	0.00	H
ATOM	278	HG2	GLU	A	38	14.782	37.657	17.623	1.00	0.00	H
ATOM	279	CD	GLU	A	38	14.406	39.529	18.527	1.00	0.00	C
ATOM	280	OE1	GLU	A	38	14.322	40.088	19.606	1.00	0.00	O
ATOM	281	OE2	GLU	A	38	14.445	40.167	17.431	1.00	0.00	O
ATOM	282	C	GLU	A	38	17.863	37.626	20.736	1.00	0.00	C
ATOM	283	O	GLU	A	38	17.753	37.950	21.895	1.00	0.00	O
ATOM	284	N	ALA	A	39	18.721	36.734	20.281	1.00	0.00	N
ATOM	285	H	ALA	A	39	18.738	36.590	19.292	1.00	0.00	H
ATOM	286	CA	ALA	A	39	19.629	35.952	21.060	1.00	0.00	C
ATOM	287	HA	ALA	A	39	19.273	35.907	21.993	1.00	0.00	H
ATOM	288	CB	ALA	A	39	19.644	34.637	20.362	1.00	0.00	C
ATOM	289	HB1	ALA	A	39	20.261	34.013	20.841	1.00	0.00	H
ATOM	290	HB2	ALA	A	39	18.721	34.253	20.355	1.00	0.00	H
ATOM	291	HB3	ALA	A	39	19.960	34.762	19.422	1.00	0.00	H
ATOM	292	C	ALA	A	39	21.066	36.554	21.111	1.00	0.00	C
ATOM	293	O	ALA	A	39	21.880	36.108	21.951	1.00	0.00	O
ATOM	294	N	ARG	A	40	21.403	37.530	20.264	1.00	0.00	N
ATOM	295	H	ARG	A	40	20.814	37.672	19.468	1.00	0.00	H
ATOM	296	CA	ARG	A	40	22.559	38.404	20.406	1.00	0.00	C
ATOM	297	HA	ARG	A	40	23.297	37.738	20.511	1.00	0.00	H
ATOM	298	CB	ARG	A	40	22.810	39.343	19.189	1.00	0.00	C
ATOM	299	HB1	ARG	A	40	22.200	40.133	19.254	1.00	0.00	H
ATOM	300	HB2	ARG	A	40	22.619	38.843	18.344	1.00	0.00	H
ATOM	301	CG	ARG	A	40	24.263	39.847	19.143	1.00	0.00	C
ATOM	302	HG1	ARG	A	40	24.880	39.060	19.134	1.00	0.00	H
ATOM	303	HG2	ARG	A	40	24.441	40.401	19.956	1.00	0.00	H
ATOM	304	CD	ARG	A	40	24.515	40.703	17.883	1.00	0.00	C
ATOM	305	HD1	ARG	A	40	25.239	41.366	18.074	1.00	0.00	H
ATOM	306	HD2	ARG	A	40	23.675	41.188	17.641	1.00	0.00	H
ATOM	307	NE	ARG	A	40	24.935	39.901	16.697	1.00	0.00	N
ATOM	308	HE	ARG	A	40	24.912	38.902	16.743	1.00	0.00	H
ATOM	309	CZ	ARG	A	40	25.335	40.491	15.593	1.00	0.00	C
ATOM	310	NH1	ARG	A	40	26.027	39.837	14.707	1.00	0.00	N
ATOM	311	HH11	ARG	A	40	26.259	38.877	14.863	1.00	0.00	H
ATOM	312	HH12	ARG	A	40	26.327	40.294	13.870	1.00	0.00	H
ATOM	313	NH2	ARG	A	40	25.061	41.753	15.399	1.00	0.00	N
ATOM	314	HH21	ARG	A	40	24.549	42.265	16.089	1.00	0.00	H
ATOM	315	HH22	ARG	A	40	25.363	42.207	14.561	1.00	0.00	H
ATOM	316	C	ARG	A	40	22.435	39.268	21.619	1.00	0.00	C
ATOM	317	O	ARG	A	40	21.463	39.896	21.846	1.00	0.00	O
ATOM	318	N	PHE	A	41	23.501	39.395	22.426	1.00	0.00	N
ATOM	319	H	PHE	A	41	24.384	39.067	22.091	1.00	0.00	H
ATOM	320	CA	PHE	A	41	23.450	39.980	23.758	1.00	0.00	C
ATOM	321	HA	PHE	A	41	22.641	40.566	23.810	1.00	0.00	H

ATOM	322	CB	PHE	A	41	23.256	38.919	24.775	1.00	0.00	C
ATOM	323	HB1	PHE	A	41	23.971	38.235	24.633	1.00	0.00	H
ATOM	324	HB2	PHE	A	41	22.361	38.506	24.608	1.00	0.00	H
ATOM	325	CG	PHE	A	41	23.303	39.327	26.206	1.00	0.00	C
ATOM	326	CD1	PHE	A	41	24.505	39.202	26.925	1.00	0.00	C
ATOM	327	HD1	PHE	A	41	25.351	38.962	26.448	1.00	0.00	H
ATOM	328	CE1	PHE	A	41	24.516	39.410	28.282	1.00	0.00	C
ATOM	329	HE1	PHE	A	41	25.329	39.159	28.807	1.00	0.00	H
ATOM	330	CZ	PHE	A	41	23.435	39.955	28.937	1.00	0.00	C
ATOM	331	HZ	PHE	A	41	23.512	40.240	29.892	1.00	0.00	H
ATOM	332	CE2	PHE	A	41	22.244	40.102	28.257	1.00	0.00	C
ATOM	333	HE2	PHE	A	41	21.453	40.497	28.724	1.00	0.00	H
ATOM	334	CD2	PHE	A	41	22.132	39.699	26.905	1.00	0.00	C
ATOM	335	HD2	PHE	A	41	21.241	39.678	26.452	1.00	0.00	H
ATOM	336	C	PHE	A	41	24.781	40.766	23.996	1.00	0.00	C
ATOM	337	O	PHE	A	41	25.832	40.126	23.758	1.00	0.00	O
ATOM	338	N	LEU	A	42	24.784	42.024	24.383	1.00	0.00	N
ATOM	339	H	LEU	A	42	23.904	42.374	24.704	1.00	0.00	H
ATOM	340	CA	LEU	A	42	25.908	43.002	24.412	1.00	0.00	C
ATOM	341	HA	LEU	A	42	26.782	42.544	24.249	1.00	0.00	H
ATOM	342	CB	LEU	A	42	25.661	44.059	23.284	1.00	0.00	C
ATOM	343	HB1	LEU	A	42	26.401	44.731	23.312	1.00	0.00	H
ATOM	344	HB2	LEU	A	42	24.789	44.515	23.462	1.00	0.00	H
ATOM	345	CG	LEU	A	42	25.620	43.359	21.834	1.00	0.00	C
ATOM	346	HG	LEU	A	42	24.866	42.707	21.762	1.00	0.00	H
ATOM	347	CD1	LEU	A	42	25.322	44.424	20.849	1.00	0.00	C
ATOM	348	HD11	LEU	A	42	25.288	44.029	19.931	1.00	0.00	H
ATOM	349	HD12	LEU	A	42	24.439	44.841	21.066	1.00	0.00	H
ATOM	350	HD13	LEU	A	42	26.038	45.121	20.885	1.00	0.00	H
ATOM	351	CD2	LEU	A	42	26.860	42.444	21.463	1.00	0.00	C
ATOM	352	HD21	LEU	A	42	26.731	42.063	20.548	1.00	0.00	H
ATOM	353	HD22	LEU	A	42	27.696	42.992	21.481	1.00	0.00	H
ATOM	354	HD23	LEU	A	42	26.935	41.700	22.126	1.00	0.00	H
ATOM	355	C	LEU	A	42	26.043	43.570	25.800	1.00	0.00	C
ATOM	356	O	LEU	A	42	25.077	43.675	26.495	1.00	0.00	O
ATOM	357	N	PRO	A	43	27.191	44.165	26.275	1.00	0.00	N
ATOM	358	CD	PRO	A	43	28.451	44.041	25.657	1.00	0.00	C
ATOM	359	HD1	PRO	A	43	28.460	44.623	24.844	1.00	0.00	H
ATOM	360	HD2	PRO	A	43	28.574	43.087	25.385	1.00	0.00	H
ATOM	361	CG	PRO	A	43	29.517	44.463	26.630	1.00	0.00	C
ATOM	362	HG1	PRO	A	43	30.251	44.950	26.156	1.00	0.00	H
ATOM	363	HG2	PRO	A	43	29.896	43.667	27.101	1.00	0.00	H
ATOM	364	CB	PRO	A	43	28.700	45.408	27.607	1.00	0.00	C
ATOM	365	HB1	PRO	A	43	28.726	46.360	27.303	1.00	0.00	H
ATOM	366	HB2	PRO	A	43	29.041	45.348	28.545	1.00	0.00	H
ATOM	367	CA	PRO	A	43	27.279	44.885	27.541	1.00	0.00	C
ATOM	368	HA	PRO	A	43	27.049	44.162	28.192	1.00	0.00	H
ATOM	369	C	PRO	A	43	26.257	46.059	27.861	1.00	0.00	C
ATOM	370	O	PRO	A	43	26.018	46.324	29.061	1.00	0.00	O
ATOM	371	N	LYS	A	44	25.621	46.695	26.868	1.00	0.00	N
ATOM	372	H	LYS	A	44	25.965	46.575	25.937	1.00	0.00	H
ATOM	373	CA	LYS	A	44	24.457	47.552	27.063	1.00	0.00	C
ATOM	374	HA	LYS	A	44	24.896	48.254	27.623	1.00	0.00	H
ATOM	375	CB	LYS	A	44	23.901	48.167	25.793	1.00	0.00	C

ATOM	376	HB1	LYS	A	44	23.792	47.407	25.152	1.00	0.00	H
ATOM	377	HB2	LYS	A	44	24.613	48.785	25.459	1.00	0.00	H
ATOM	378	CG	LYS	A	44	22.598	48.958	25.731	1.00	0.00	C
ATOM	379	HG1	LYS	A	44	21.837	48.323	25.861	1.00	0.00	H
ATOM	380	HG2	LYS	A	44	22.529	49.381	24.828	1.00	0.00	H
ATOM	381	CD	LYS	A	44	22.522	50.063	26.812	1.00	0.00	C
ATOM	382	HD1	LYS	A	44	23.448	50.410	26.962	1.00	0.00	H
ATOM	383	HD2	LYS	A	44	22.182	49.645	27.655	1.00	0.00	H
ATOM	384	CE	LYS	A	44	21.578	51.273	26.424	1.00	0.00	C
ATOM	385	HE1	LYS	A	44	20.624	50.986	26.511	1.00	0.00	H
ATOM	386	HE2	LYS	A	44	21.761	51.542	25.478	1.00	0.00	H
ATOM	387	NZ	LYS	A	44	21.736	52.509	27.263	1.00	0.00	N
ATOM	388	HZ1	LYS	A	44	21.101	53.212	26.943	1.00	0.00	H
ATOM	389	HZ2	LYS	A	44	21.538	52.291	28.219	1.00	0.00	H
ATOM	390	HZ3	LYS	A	44	22.674	52.846	27.186	1.00	0.00	H
ATOM	391	C	LYS	A	44	23.291	46.902	27.816	1.00	0.00	C
ATOM	392	O	LYS	A	44	22.555	47.493	28.554	1.00	0.00	O
ATOM	393	N	ASP	A	45	23.124	45.583	27.505	1.00	0.00	N
ATOM	394	H	ASP	A	45	23.792	45.150	26.900	1.00	0.00	H
ATOM	395	CA	ASP	A	45	22.011	44.766	28.014	1.00	0.00	C
ATOM	396	HA	ASP	A	45	21.234	45.389	27.921	1.00	0.00	H
ATOM	397	CB	ASP	A	45	21.844	43.530	27.121	1.00	0.00	C
ATOM	398	HB1	ASP	A	45	21.074	42.998	27.473	1.00	0.00	H
ATOM	399	HB2	ASP	A	45	22.682	42.988	27.186	1.00	0.00	H
ATOM	400	CG	ASP	A	45	21.581	43.815	25.637	1.00	0.00	C
ATOM	401	OD1	ASP	A	45	21.965	42.939	24.803	1.00	0.00	O
ATOM	402	OD2	ASP	A	45	20.988	44.926	25.351	1.00	0.00	O
ATOM	403	C	ASP	A	45	21.975	44.455	29.525	1.00	0.00	C
ATOM	404	O	ASP	A	45	20.989	43.967	30.085	1.00	0.00	O
ATOM	405	N	LEU	A	46	23.105	44.668	30.171	1.00	0.00	N
ATOM	406	H	LEU	A	46	23.849	45.084	29.648	1.00	0.00	H
ATOM	407	CA	LEU	A	46	23.379	44.360	31.565	1.00	0.00	C
ATOM	408	HA	LEU	A	46	23.056	43.443	31.800	1.00	0.00	H
ATOM	409	CB	LEU	A	46	24.865	44.333	31.772	1.00	0.00	C
ATOM	410	HB1	LEU	A	46	25.019	44.239	32.756	1.00	0.00	H
ATOM	411	HB2	LEU	A	46	25.217	45.216	31.462	1.00	0.00	H
ATOM	412	CG	LEU	A	46	25.654	43.217	31.050	1.00	0.00	C
ATOM	413	HG	LEU	A	46	25.362	43.255	30.094	1.00	0.00	H
ATOM	414	CD1	LEU	A	46	27.193	43.409	31.077	1.00	0.00	C
ATOM	415	HD11	LEU	A	46	27.632	42.652	30.593	1.00	0.00	H
ATOM	416	HD12	LEU	A	46	27.429	44.272	30.630	1.00	0.00	H
ATOM	417	HD13	LEU	A	46	27.510	43.427	32.025	1.00	0.00	H
ATOM	418	CD2	LEU	A	46	25.325	41.854	31.679	1.00	0.00	C
ATOM	419	HD21	LEU	A	46	25.838	41.137	31.208	1.00	0.00	H
ATOM	420	HD22	LEU	A	46	25.578	41.864	32.647	1.00	0.00	H
ATOM	421	HD23	LEU	A	46	24.345	41.675	31.592	1.00	0.00	H
ATOM	422	C	LEU	A	46	22.608	45.331	32.431	1.00	0.00	C
ATOM	423	O	LEU	A	46	22.379	46.489	32.032	1.00	0.00	O
ATOM	424	N	ASP	A	47	22.312	44.954	33.696	1.00	0.00	N
ATOM	425	H	ASP	A	47	22.564	44.025	33.967	1.00	0.00	H
ATOM	426	CA	ASP	A	47	21.662	45.780	34.692	1.00	0.00	C
ATOM	427	HA	ASP	A	47	21.294	46.642	34.343	1.00	0.00	H
ATOM	428	CB	ASP	A	47	20.487	44.909	35.187	1.00	0.00	C
ATOM	429	HB1	ASP	A	47	20.850	44.017	35.455	1.00	0.00	H

ATOM	430	HB2	ASP	A	47	19.843	44.793	34.431	1.00	0.00	H
ATOM	431	CG	ASP	A	47	19.719	45.501	36.390	1.00	0.00	C
ATOM	432	OD1	ASP	A	47	18.498	45.662	36.382	1.00	0.00	O
ATOM	433	OD2	ASP	A	47	20.321	45.818	37.376	1.00	0.00	O
ATOM	434	C	ASP	A	47	22.742	46.178	35.719	1.00	0.00	C
ATOM	435	O	ASP	A	47	23.458	45.285	36.112	1.00	0.00	O
ATOM	436	N	PRO	A	48	22.817	47.403	36.198	1.00	0.00	N
ATOM	437	CD	PRO	A	48	21.992	48.513	35.751	1.00	0.00	C
ATOM	438	HD1	PRO	A	48	21.125	48.526	36.250	1.00	0.00	H
ATOM	439	HD2	PRO	A	48	21.811	48.440	34.770	1.00	0.00	H
ATOM	440	CG	PRO	A	48	22.846	49.720	36.077	1.00	0.00	C
ATOM	441	HG1	PRO	A	48	22.263	50.506	36.281	1.00	0.00	H
ATOM	442	HG2	PRO	A	48	23.440	49.938	35.302	1.00	0.00	H
ATOM	443	CB	PRO	A	48	23.684	49.409	37.266	1.00	0.00	C
ATOM	444	HB1	PRO	A	48	23.233	49.728	38.100	1.00	0.00	H
ATOM	445	HB2	PRO	A	48	24.579	49.848	37.182	1.00	0.00	H
ATOM	446	CA	PRO	A	48	23.801	47.867	37.237	1.00	0.00	C
ATOM	447	HA	PRO	A	48	24.686	47.513	36.935	1.00	0.00	H
ATOM	448	C	PRO	A	48	23.734	47.256	38.646	1.00	0.00	C
ATOM	449	O	PRO	A	48	24.660	47.442	39.418	1.00	0.00	O
ATOM	450	N	SER	A	49	22.634	46.623	39.053	1.00	0.00	N
ATOM	451	H	SER	A	49	21.924	46.448	38.371	1.00	0.00	H
ATOM	452	CA	SER	A	49	22.379	46.156	40.445	1.00	0.00	C
ATOM	453	HA	SER	A	49	23.322	45.958	40.713	1.00	0.00	H
ATOM	454	CB	SER	A	49	21.786	47.275	41.337	1.00	0.00	C
ATOM	455	HB1	SER	A	49	22.438	48.023	41.456	1.00	0.00	H
ATOM	456	HB2	SER	A	49	21.521	46.918	42.233	1.00	0.00	H
ATOM	457	OG	SER	A	49	20.645	47.840	40.793	1.00	0.00	O
ATOM	458	HG	SER	A	49	20.303	48.554	41.404	1.00	0.00	H
ATOM	459	C	SER	A	49	21.639	44.796	40.638	1.00	0.00	C
ATOM	460	O	SER	A	49	21.163	44.553	41.758	1.00	0.00	O
ATOM	461	N	LEU	A	50	21.529	43.954	39.645	1.00	0.00	N
ATOM	462	H	LEU	A	50	21.846	44.325	38.772	1.00	0.00	H
ATOM	463	CA	LEU	A	50	21.045	42.602	39.552	1.00	0.00	C
ATOM	464	HA	LEU	A	50	20.155	42.605	40.008	1.00	0.00	H
ATOM	465	CB	LEU	A	50	20.872	42.227	38.046	1.00	0.00	C
ATOM	466	HB1	LEU	A	50	21.729	42.417	37.568	1.00	0.00	H
ATOM	467	HB2	LEU	A	50	20.142	42.789	37.658	1.00	0.00	H
ATOM	468	CG	LEU	A	50	20.520	40.793	37.759	1.00	0.00	C
ATOM	469	HG	LEU	A	50	21.177	40.176	38.193	1.00	0.00	H
ATOM	470	CD1	LEU	A	50	19.071	40.662	38.276	1.00	0.00	C
ATOM	471	HD11	LEU	A	50	18.747	39.728	38.126	1.00	0.00	H
ATOM	472	HD12	LEU	A	50	19.046	40.870	39.254	1.00	0.00	H
ATOM	473	HD13	LEU	A	50	18.482	41.303	37.783	1.00	0.00	H
ATOM	474	CD2	LEU	A	50	20.514	40.526	36.227	1.00	0.00	C
ATOM	475	HD21	LEU	A	50	20.278	39.569	36.056	1.00	0.00	H
ATOM	476	HD22	LEU	A	50	19.839	41.119	35.788	1.00	0.00	H
ATOM	477	HD23	LEU	A	50	21.421	40.718	35.852	1.00	0.00	H
ATOM	478	C	LEU	A	50	22.085	41.621	40.272	1.00	0.00	C
ATOM	479	O	LEU	A	50	21.732	40.920	41.280	1.00	0.00	O
ATOM	480	N	CYS	A	51	23.366	41.690	39.913	1.00	0.00	N
ATOM	481	H	CYS	A	51	23.582	42.316	39.164	1.00	0.00	H
ATOM	482	CA	CYS	A	51	24.531	40.932	40.506	1.00	0.00	C
ATOM	483	HA	CYS	A	51	24.194	40.216	41.117	1.00	0.00	H

ATOM	484	CB	CYS	A	51	25.316	40.252	39.372	1.00	0.00	C
ATOM	485	HB1	CYS	A	51	26.020	39.740	39.864	1.00	0.00	H
ATOM	486	HB2	CYS	A	51	25.737	41.025	38.898	1.00	0.00	H
ATOM	487	SG	CYS	A	51	24.368	39.169	38.210	1.00	0.00	S
ATOM	488	C	CYS	A	51	25.348	41.783	41.440	1.00	0.00	C
ATOM	489	O	CYS	A	51	25.347	43.024	41.402	1.00	0.00	O
ATOM	490	N	THR	A	52	26.123	41.153	42.369	1.00	0.00	N
ATOM	491	H	THR	A	52	26.082	40.159	42.475	1.00	0.00	H
ATOM	492	CA	THR	A	52	27.037	41.973	43.229	1.00	0.00	C
ATOM	493	HA	THR	A	52	26.578	42.860	43.277	1.00	0.00	H
ATOM	494	CB	THR	A	52	27.230	41.346	44.657	1.00	0.00	C
ATOM	495	HB	THR	A	52	28.009	41.785	45.105	1.00	0.00	H
ATOM	496	CG2	THR	A	52	25.844	41.406	45.436	1.00	0.00	C
ATOM	497	HG21	THR	A	52	25.956	41.009	46.347	1.00	0.00	H
ATOM	498	HG22	THR	A	52	25.550	42.358	45.521	1.00	0.00	H
ATOM	499	HG23	THR	A	52	25.154	40.889	44.929	1.00	0.00	H
ATOM	500	OG1	THR	A	52	27.714	40.042	44.676	1.00	0.00	O
ATOM	501	HG1	THR	A	52	27.802	39.733	45.623	1.00	0.00	H
ATOM	502	C	THR	A	52	28.436	42.111	42.590	1.00	0.00	C
ATOM	503	O	THR	A	52	29.166	43.063	42.844	1.00	0.00	O
ATOM	504	N	HIS	A	53	28.752	41.089	41.742	1.00	0.00	N
ATOM	505	H	HIS	A	53	28.058	40.374	41.663	1.00	0.00	H
ATOM	506	CA	HIS	A	53	29.956	40.880	40.928	1.00	0.00	C
ATOM	507	HA	HIS	A	53	30.323	41.793	40.750	1.00	0.00	H
ATOM	508	CB	HIS	A	53	30.966	39.870	41.737	1.00	0.00	C
ATOM	509	HB1	HIS	A	53	31.841	39.806	41.257	1.00	0.00	H
ATOM	510	HB2	HIS	A	53	30.556	38.960	41.805	1.00	0.00	H
ATOM	511	CG	HIS	A	53	31.317	40.238	43.128	1.00	0.00	C
ATOM	512	ND1	HIS	A	53	30.441	40.151	44.150	1.00	0.00	N
ATOM	513	HD1	HIS	A	53	29.463	39.948	44.113	1.00	0.00	H
ATOM	514	CE1	HIS	A	53	31.203	40.404	45.236	1.00	0.00	C
ATOM	515	HE1	HIS	A	53	30.903	40.279	46.182	1.00	0.00	H
ATOM	516	NE2	HIS	A	53	32.392	40.837	44.883	1.00	0.00	N
ATOM	517	CD2	HIS	A	53	32.584	40.630	43.540	1.00	0.00	C
ATOM	518	HD2	HIS	A	53	33.419	40.734	42.999	1.00	0.00	H
ATOM	519	C	HIS	A	53	29.600	40.314	39.571	1.00	0.00	C
ATOM	520	O	HIS	A	53	28.885	39.356	39.458	1.00	0.00	O
ATOM	521	N	LEU	A	54	30.111	40.945	38.461	1.00	0.00	N
ATOM	522	H	LEU	A	54	30.583	41.818	38.587	1.00	0.00	H
ATOM	523	CA	LEU	A	54	30.003	40.418	37.138	1.00	0.00	C
ATOM	524	HA	LEU	A	54	29.317	39.690	37.138	1.00	0.00	H
ATOM	525	CB	LEU	A	54	29.616	41.590	36.162	1.00	0.00	C
ATOM	526	HB1	LEU	A	54	29.725	41.263	35.223	1.00	0.00	H
ATOM	527	HB2	LEU	A	54	30.243	42.352	36.325	1.00	0.00	H
ATOM	528	CG	LEU	A	54	28.165	42.133	36.307	1.00	0.00	C
ATOM	529	HG	LEU	A	54	28.081	42.652	37.158	1.00	0.00	H
ATOM	530	CD1	LEU	A	54	27.833	42.999	35.122	1.00	0.00	C
ATOM	531	HD11	LEU	A	54	26.901	43.348	35.215	1.00	0.00	H
ATOM	532	HD12	LEU	A	54	28.473	43.766	35.080	1.00	0.00	H
ATOM	533	HD13	LEU	A	54	27.905	42.459	34.284	1.00	0.00	H
ATOM	534	CD2	LEU	A	54	27.168	41.055	36.399	1.00	0.00	C
ATOM	535	HD21	LEU	A	54	26.255	41.452	36.491	1.00	0.00	H
ATOM	536	HD22	LEU	A	54	27.206	40.494	35.572	1.00	0.00	H
ATOM	537	HD23	LEU	A	54	27.365	40.486	37.197	1.00	0.00	H

ATOM	538	C	LEU	A	54	31.362	39.866	36.791	1.00	0.00	C
ATOM	539	O	LEU	A	54	32.360	40.458	37.191	1.00	0.00	O
ATOM	540	N	ILE	A	55	31.443	38.799	36.060	1.00	0.00	N
ATOM	541	H	ILE	A	55	30.633	38.241	35.879	1.00	0.00	H
ATOM	542	CA	ILE	A	55	32.771	38.420	35.494	1.00	0.00	C
ATOM	543	HA	ILE	A	55	33.347	39.152	35.857	1.00	0.00	H
ATOM	544	CB	ILE	A	55	33.268	37.041	35.988	1.00	0.00	C
ATOM	545	HB	ILE	A	55	32.706	36.322	35.578	1.00	0.00	H
ATOM	546	CG2	ILE	A	55	34.728	36.812	35.553	1.00	0.00	C
ATOM	547	HG21	ILE	A	55	35.036	35.918	35.878	1.00	0.00	H
ATOM	548	HG22	ILE	A	55	34.787	36.842	34.555	1.00	0.00	H
ATOM	549	HG23	ILE	A	55	35.308	37.527	35.943	1.00	0.00	H
ATOM	550	CG1	ILE	A	55	33.111	36.923	37.505	1.00	0.00	C
ATOM	551	HG11	ILE	A	55	32.134	36.830	37.697	1.00	0.00	H
ATOM	552	HG12	ILE	A	55	33.450	37.774	37.907	1.00	0.00	H
ATOM	553	CD	ILE	A	55	33.832	35.763	38.205	1.00	0.00	C
ATOM	554	HD1	ILE	A	55	33.648	35.801	39.187	1.00	0.00	H
ATOM	555	HD2	ILE	A	55	33.501	34.894	37.837	1.00	0.00	H
ATOM	556	HD3	ILE	A	55	34.817	35.838	38.047	1.00	0.00	H
ATOM	557	C	ILE	A	55	32.801	38.402	33.929	1.00	0.00	C
ATOM	558	O	ILE	A	55	31.813	37.925	33.395	1.00	0.00	O
ATOM	559	N	TYR	A	56	33.752	38.971	33.234	1.00	0.00	N
ATOM	560	H	TYR	A	56	34.420	39.541	33.713	1.00	0.00	H
ATOM	561	CA	TYR	A	56	33.885	38.803	31.730	1.00	0.00	C
ATOM	562	HA	TYR	A	56	32.937	38.814	31.413	1.00	0.00	H
ATOM	563	CB	TYR	A	56	34.636	39.944	31.090	1.00	0.00	C
ATOM	564	HB1	TYR	A	56	35.576	39.905	31.428	1.00	0.00	H
ATOM	565	HB2	TYR	A	56	34.205	40.792	31.399	1.00	0.00	H
ATOM	566	CG	TYR	A	56	34.680	39.962	29.551	1.00	0.00	C
ATOM	567	CD1	TYR	A	56	35.635	39.258	28.821	1.00	0.00	C
ATOM	568	HD1	TYR	A	56	36.277	38.630	29.261	1.00	0.00	H
ATOM	569	CE1	TYR	A	56	35.647	39.483	27.404	1.00	0.00	C
ATOM	570	HE1	TYR	A	56	36.250	38.923	26.835	1.00	0.00	H
ATOM	571	CZ	TYR	A	56	34.828	40.480	26.766	1.00	0.00	C
ATOM	572	OH	TYR	A	56	34.917	40.718	25.495	1.00	0.00	O
ATOM	573	HH	TYR	A	56	34.271	41.439	25.245	1.00	0.00	H
ATOM	574	CE2	TYR	A	56	33.956	41.220	27.576	1.00	0.00	C
ATOM	575	HE2	TYR	A	56	33.461	42.000	27.194	1.00	0.00	H
ATOM	576	CD2	TYR	A	56	33.777	40.869	28.918	1.00	0.00	C
ATOM	577	HD2	TYR	A	56	33.014	41.255	29.436	1.00	0.00	H
ATOM	578	C	TYR	A	56	34.637	37.478	31.324	1.00	0.00	C
ATOM	579	O	TYR	A	56	35.728	37.235	31.862	1.00	0.00	O
ATOM	580	N	ALA	A	57	34.095	36.659	30.425	1.00	0.00	N
ATOM	581	H	ALA	A	57	33.202	36.905	30.049	1.00	0.00	H
ATOM	582	CA	ALA	A	57	34.717	35.424	29.951	1.00	0.00	C
ATOM	583	HA	ALA	A	57	35.555	35.264	30.472	1.00	0.00	H
ATOM	584	CB	ALA	A	57	33.745	34.264	30.210	1.00	0.00	C
ATOM	585	HB1	ALA	A	57	34.154	33.409	29.892	1.00	0.00	H
ATOM	586	HB2	ALA	A	57	33.557	34.199	31.190	1.00	0.00	H
ATOM	587	HB3	ALA	A	57	32.891	34.429	29.717	1.00	0.00	H
ATOM	588	C	ALA	A	57	35.099	35.580	28.465	1.00	0.00	C
ATOM	589	O	ALA	A	57	34.208	35.672	27.623	1.00	0.00	O
ATOM	590	N	PHE	A	58	36.381	35.644	28.079	1.00	0.00	N
ATOM	591	H	PHE	A	58	36.525	35.808	27.103	1.00	0.00	H

ATOM	592	CA	PHE	A	58	37.614	35.508	28.883	1.00	0.00	C
ATOM	593	HA	PHE	A	58	37.353	35.841	29.789	1.00	0.00	H
ATOM	594	CB	PHE	A	58	38.083	34.039	28.884	1.00	0.00	C
ATOM	595	HB1	PHE	A	58	38.807	33.955	29.569	1.00	0.00	H
ATOM	596	HB2	PHE	A	58	38.457	33.842	27.978	1.00	0.00	H
ATOM	597	CG	PHE	A	58	37.070	32.950	29.184	1.00	0.00	C
ATOM	598	CD1	PHE	A	58	36.970	32.404	30.477	1.00	0.00	C
ATOM	599	HD1	PHE	A	58	37.499	32.799	31.228	1.00	0.00	H
ATOM	600	CE1	PHE	A	58	36.127	31.302	30.715	1.00	0.00	C
ATOM	601	HE1	PHE	A	58	36.050	30.923	31.637	1.00	0.00	H
ATOM	602	CZ	PHE	A	58	35.394	30.734	29.659	1.00	0.00	C
ATOM	603	HZ	PHE	A	58	34.818	29.933	29.823	1.00	0.00	H
ATOM	604	CE2	PHE	A	58	35.472	31.293	28.374	1.00	0.00	C
ATOM	605	HE2	PHE	A	58	34.930	30.908	27.628	1.00	0.00	H
ATOM	606	CD2	PHE	A	58	36.312	32.394	28.136	1.00	0.00	C
ATOM	607	HD2	PHE	A	58	36.372	32.785	27.217	1.00	0.00	H
ATOM	608	C	PHE	A	58	38.781	36.327	28.289	1.00	0.00	C
ATOM	609	O	PHE	A	58	38.781	36.581	27.082	1.00	0.00	O
ATOM	610	N	ALA	A	59	39.852	36.557	29.061	1.00	0.00	N
ATOM	611	H	ALA	A	59	39.723	36.666	30.047	1.00	0.00	H
ATOM	612	CA	ALA	A	59	41.209	36.654	28.509	1.00	0.00	C
ATOM	613	HA	ALA	A	59	41.107	36.952	27.560	1.00	0.00	H
ATOM	614	CB	ALA	A	59	41.973	37.703	29.315	1.00	0.00	C
ATOM	615	HB1	ALA	A	59	42.903	37.787	28.956	1.00	0.00	H
ATOM	616	HB2	ALA	A	59	41.507	38.585	29.242	1.00	0.00	H
ATOM	617	HB3	ALA	A	59	42.010	37.424	30.275	1.00	0.00	H
ATOM	618	C	ALA	A	59	41.943	35.289	28.468	1.00	0.00	C
ATOM	619	O	ALA	A	59	41.648	34.398	29.267	1.00	0.00	O
ATOM	620	N	GLY	A	60	42.948	35.158	27.590	1.00	0.00	N
ATOM	621	H	GLY	A	60	43.050	35.869	26.894	1.00	0.00	H
ATOM	622	CA	GLY	A	60	43.914	34.044	27.574	1.00	0.00	C
ATOM	623	HA1	GLY	A	60	43.989	33.694	26.640	1.00	0.00	H
ATOM	624	HA2	GLY	A	60	43.581	33.318	28.176	1.00	0.00	H
ATOM	625	C	GLY	A	60	45.255	34.495	28.041	1.00	0.00	C
ATOM	626	O	GLY	A	60	45.389	35.304	28.950	1.00	0.00	O
ATOM	627	N	MET	A	61	46.332	33.969	27.348	1.00	0.00	N
ATOM	628	H	MET	A	61	46.160	33.316	26.610	1.00	0.00	H
ATOM	629	CA	MET	A	61	47.689	34.330	27.654	1.00	0.00	C
ATOM	630	HA	MET	A	61	47.662	35.254	28.037	1.00	0.00	H
ATOM	631	CB	MET	A	61	48.258	33.314	28.724	1.00	0.00	C
ATOM	632	HB1	MET	A	61	47.760	33.467	29.578	1.00	0.00	H
ATOM	633	HB2	MET	A	61	49.225	33.530	28.858	1.00	0.00	H
ATOM	634	CG	MET	A	61	48.182	31.790	28.436	1.00	0.00	C
ATOM	635	HG1	MET	A	61	48.939	31.613	27.807	1.00	0.00	H
ATOM	636	HG2	MET	A	61	47.314	31.666	27.956	1.00	0.00	H
ATOM	637	SD	MET	A	61	48.286	30.754	29.945	1.00	0.00	S
ATOM	638	CE	MET	A	61	49.943	31.209	30.546	1.00	0.00	C
ATOM	639	HE1	MET	A	61	50.142	30.707	31.388	1.00	0.00	H
ATOM	640	HE2	MET	A	61	50.624	30.978	29.851	1.00	0.00	H
ATOM	641	HE3	MET	A	61	49.973	32.192	30.729	1.00	0.00	H
ATOM	642	C	MET	A	61	48.516	34.353	26.329	1.00	0.00	C
ATOM	643	O	MET	A	61	48.315	33.598	25.370	1.00	0.00	O
ATOM	644	N	THR	A	62	49.617	35.144	26.278	1.00	0.00	N
ATOM	645	H	THR	A	62	49.748	35.825	26.998	1.00	0.00	H

ATOM	646	CA	THR	A	62	50.643	35.046	25.198	1.00	0.00	C
ATOM	647	HA	THR	A	62	50.831	34.074	25.056	1.00	0.00	H
ATOM	648	CB	THR	A	62	50.113	35.686	23.925	1.00	0.00	C
ATOM	649	HB	THR	A	62	49.690	36.545	24.212	1.00	0.00	H
ATOM	650	CG2	THR	A	62	51.208	35.785	22.891	1.00	0.00	C
ATOM	651	HG21	THR	A	62	50.845	36.207	22.060	1.00	0.00	H
ATOM	652	HG22	THR	A	62	51.956	36.344	23.250	1.00	0.00	H
ATOM	653	HG23	THR	A	62	51.549	34.869	22.678	1.00	0.00	H
ATOM	654	OG1	THR	A	62	49.129	34.926	23.252	1.00	0.00	O
ATOM	655	HG1	THR	A	62	48.835	35.414	22.430	1.00	0.00	H
ATOM	656	C	THR	A	62	51.913	35.785	25.607	1.00	0.00	C
ATOM	657	O	THR	A	62	51.822	36.921	26.036	1.00	0.00	O
ATOM	658	N	ASN	A	63	53.073	35.212	25.287	1.00	0.00	N
ATOM	659	H	ASN	A	63	53.014	34.375	24.744	1.00	0.00	H
ATOM	660	CA	ASN	A	63	54.414	35.655	25.631	1.00	0.00	C
ATOM	661	HA	ASN	A	63	55.037	34.951	25.291	1.00	0.00	H
ATOM	662	CB	ASN	A	63	54.797	36.925	24.883	1.00	0.00	C
ATOM	663	HB1	ASN	A	63	55.731	37.164	25.148	1.00	0.00	H
ATOM	664	HB2	ASN	A	63	54.173	37.650	25.175	1.00	0.00	H
ATOM	665	CG	ASN	A	63	54.763	36.899	23.387	1.00	0.00	C
ATOM	666	OD1	ASN	A	63	54.766	35.914	22.678	1.00	0.00	O
ATOM	667	ND2	ASN	A	63	54.542	38.013	22.757	1.00	0.00	N
ATOM	668	HD21	ASN	A	63	54.400	38.859	23.271	1.00	0.00	H
ATOM	669	HD22	ASN	A	63	54.514	38.025	21.757	1.00	0.00	H
ATOM	670	C	ASN	A	63	54.572	35.732	27.160	1.00	0.00	C
ATOM	671	O	ASN	A	63	55.126	36.668	27.771	1.00	0.00	O
ATOM	672	N	HIS	A	64	54.039	34.668	27.832	1.00	0.00	N
ATOM	673	H	HIS	A	64	53.697	33.901	27.290	1.00	0.00	H
ATOM	674	CA	HIS	A	64	53.931	34.569	29.319	1.00	0.00	C
ATOM	675	HA	HIS	A	64	53.317	33.795	29.478	1.00	0.00	H
ATOM	676	CB	HIS	A	64	55.325	34.346	29.948	1.00	0.00	C
ATOM	677	HB1	HIS	A	64	55.206	34.069	30.901	1.00	0.00	H
ATOM	678	HB2	HIS	A	64	55.834	35.206	29.912	1.00	0.00	H
ATOM	679	CG	HIS	A	64	56.172	33.306	29.290	1.00	0.00	C
ATOM	680	ND1	HIS	A	64	55.871	32.001	29.174	1.00	0.00	N
ATOM	681	CE1	HIS	A	64	56.894	31.382	28.519	1.00	0.00	C
ATOM	682	HE1	HIS	A	64	56.964	30.407	28.310	1.00	0.00	H
ATOM	683	NE2	HIS	A	64	57.818	32.362	28.198	1.00	0.00	N
ATOM	684	HE2	HIS	A	64	58.679	32.198	27.717	1.00	0.00	H
ATOM	685	CD2	HIS	A	64	57.379	33.612	28.640	1.00	0.00	C
ATOM	686	HD2	HIS	A	64	57.816	34.504	28.523	1.00	0.00	H
ATOM	687	C	HIS	A	64	53.253	35.732	29.964	1.00	0.00	C
ATOM	688	O	HIS	A	64	53.394	35.859	31.173	1.00	0.00	O
ATOM	689	N	GLN	A	65	52.572	36.569	29.266	1.00	0.00	N
ATOM	690	H	GLN	A	65	52.667	36.534	28.271	1.00	0.00	H
ATOM	691	CA	GLN	A	65	51.668	37.569	29.830	1.00	0.00	C
ATOM	692	HA	GLN	A	65	51.816	37.620	30.818	1.00	0.00	H
ATOM	693	CB	GLN	A	65	52.031	38.896	29.112	1.00	0.00	C
ATOM	694	HB1	GLN	A	65	51.341	39.570	29.377	1.00	0.00	H
ATOM	695	HB2	GLN	A	65	51.968	38.723	28.129	1.00	0.00	H
ATOM	696	CG	GLN	A	65	53.434	39.532	29.385	1.00	0.00	C
ATOM	697	HG1	GLN	A	65	54.128	38.905	29.031	1.00	0.00	H
ATOM	698	HG2	GLN	A	65	53.541	39.623	30.375	1.00	0.00	H
ATOM	699	CD	GLN	A	65	53.753	40.893	28.797	1.00	0.00	C

ATOM	700	OE1	GLN	A	65	52.865	41.625	28.385	1.00	0.00	O
ATOM	701	NE2	GLN	A	65	54.976	41.186	28.580	1.00	0.00	N
ATOM	702	HE21	GLN	A	65	55.695	40.525	28.797	1.00	0.00	H
ATOM	703	HE22	GLN	A	65	55.216	42.076	28.193	1.00	0.00	H
ATOM	704	C	GLN	A	65	50.178	37.166	29.628	1.00	0.00	C
ATOM	705	O	GLN	A	65	49.824	36.154	28.944	1.00	0.00	O
ATOM	706	N	LEU	A	66	49.267	37.973	30.161	1.00	0.00	N
ATOM	707	H	LEU	A	66	49.590	38.605	30.866	1.00	0.00	H
ATOM	708	CA	LEU	A	66	47.823	38.040	29.825	1.00	0.00	C
ATOM	709	HA	LEU	A	66	47.447	37.127	29.983	1.00	0.00	H
ATOM	710	CB	LEU	A	66	47.066	39.030	30.775	1.00	0.00	C
ATOM	711	HB1	LEU	A	66	46.442	39.614	30.256	1.00	0.00	H
ATOM	712	HB2	LEU	A	66	47.712	39.600	31.282	1.00	0.00	H
ATOM	713	CG	LEU	A	66	46.275	38.216	31.743	1.00	0.00	C
ATOM	714	HG	LEU	A	66	45.782	37.609	31.120	1.00	0.00	H
ATOM	715	CD1	LEU	A	66	47.166	37.447	32.796	1.00	0.00	C
ATOM	716	HD11	LEU	A	66	46.577	36.923	33.412	1.00	0.00	H
ATOM	717	HD12	LEU	A	66	47.785	36.824	32.317	1.00	0.00	H
ATOM	718	HD13	LEU	A	66	47.700	38.106	33.326	1.00	0.00	H
ATOM	719	CD2	LEU	A	66	45.271	39.005	32.514	1.00	0.00	C
ATOM	720	HD21	LEU	A	66	44.780	38.399	33.140	1.00	0.00	H
ATOM	721	HD22	LEU	A	66	45.738	39.718	33.038	1.00	0.00	H
ATOM	722	HD23	LEU	A	66	44.622	39.427	31.881	1.00	0.00	H
ATOM	723	C	LEU	A	66	47.699	38.485	28.303	1.00	0.00	C
ATOM	724	O	LEU	A	66	48.611	38.981	27.701	1.00	0.00	O
ATOM	725	N	SER	A	67	46.613	38.117	27.638	1.00	0.00	N
ATOM	726	H	SER	A	67	45.981	37.501	28.108	1.00	0.00	H
ATOM	727	CA	SER	A	67	46.249	38.530	26.272	1.00	0.00	C
ATOM	728	HA	SER	A	67	46.501	39.496	26.217	1.00	0.00	H
ATOM	729	CB	SER	A	67	47.018	37.639	25.282	1.00	0.00	C
ATOM	730	HB1	SER	A	67	46.523	36.786	25.116	1.00	0.00	H
ATOM	731	HB2	SER	A	67	47.932	37.431	25.630	1.00	0.00	H
ATOM	732	OG	SER	A	67	47.174	38.300	24.025	1.00	0.00	O
ATOM	733	HG	SER	A	67	47.675	37.706	23.396	1.00	0.00	H
ATOM	734	C	SER	A	67	44.744	38.408	25.977	1.00	0.00	C
ATOM	735	O	SER	A	67	44.011	37.748	26.647	1.00	0.00	O
ATOM	736	N	THR	A	68	44.227	39.096	24.915	1.00	0.00	N
ATOM	737	H	THR	A	68	44.859	39.677	24.401	1.00	0.00	H
ATOM	738	CA	THR	A	68	42.822	39.063	24.454	1.00	0.00	C
ATOM	739	HA	THR	A	68	42.249	39.136	25.270	1.00	0.00	H
ATOM	740	CB	THR	A	68	42.656	40.278	23.502	1.00	0.00	C
ATOM	741	HB	THR	A	68	41.710	40.258	23.178	1.00	0.00	H
ATOM	742	CG2	THR	A	68	43.002	41.587	24.155	1.00	0.00	C
ATOM	743	HG21	THR	A	68	42.877	42.331	23.498	1.00	0.00	H
ATOM	744	HG22	THR	A	68	42.404	41.735	24.943	1.00	0.00	H
ATOM	745	HG23	THR	A	68	43.955	41.567	24.458	1.00	0.00	H
ATOM	746	OG1	THR	A	68	43.381	40.210	22.321	1.00	0.00	O
ATOM	747	HG1	THR	A	68	43.211	41.032	21.777	1.00	0.00	H
ATOM	748	C	THR	A	68	42.413	37.709	23.762	1.00	0.00	C
ATOM	749	O	THR	A	68	43.292	36.878	23.503	1.00	0.00	O
ATOM	750	N	THR	A	69	41.131	37.424	23.616	1.00	0.00	N
ATOM	751	H	THR	A	69	40.458	38.082	23.954	1.00	0.00	H
ATOM	752	CA	THR	A	69	40.644	36.195	22.985	1.00	0.00	C
ATOM	753	HA	THR	A	69	41.441	35.619	22.805	1.00	0.00	H

ATOM	754	CB	THR	A	69	39.700	35.428	23.925	1.00	0.00	C
ATOM	755	HB	THR	A	69	38.868	35.970	24.041	1.00	0.00	H
ATOM	756	CG2	THR	A	69	39.408	34.021	23.414	1.00	0.00	C
ATOM	757	HG21	THR	A	69	38.793	33.556	24.051	1.00	0.00	H
ATOM	758	HG22	THR	A	69	38.976	34.076	22.514	1.00	0.00	H
ATOM	759	HG23	THR	A	69	40.263	33.508	23.343	1.00	0.00	H
ATOM	760	OG1	THR	A	69	40.272	35.270	25.205	1.00	0.00	O
ATOM	761	HG1	THR	A	69	39.638	34.769	25.794	1.00	0.00	H
ATOM	762	C	THR	A	69	39.974	36.547	21.668	1.00	0.00	C
ATOM	763	O	THR	A	69	40.399	36.074	20.621	1.00	0.00	O
ATOM	764	N	GLU	A	70	39.044	37.502	21.699	1.00	0.00	N
ATOM	765	H	GLU	A	70	38.920	38.008	22.553	1.00	0.00	H
ATOM	766	CA	GLU	A	70	38.193	37.857	20.561	1.00	0.00	C
ATOM	767	HA	GLU	A	70	38.455	37.252	19.809	1.00	0.00	H
ATOM	768	CB	GLU	A	70	36.725	37.629	20.948	1.00	0.00	C
ATOM	769	HB1	GLU	A	70	36.152	37.866	20.164	1.00	0.00	H
ATOM	770	HB2	GLU	A	70	36.504	38.230	21.716	1.00	0.00	H
ATOM	771	CG	GLU	A	70	36.415	36.179	21.358	1.00	0.00	C
ATOM	772	HG1	GLU	A	70	35.457	36.121	21.640	1.00	0.00	H
ATOM	773	HG2	GLU	A	70	37.005	35.927	22.125	1.00	0.00	H
ATOM	774	CD	GLU	A	70	36.648	35.186	20.218	1.00	0.00	C
ATOM	775	OE1	GLU	A	70	35.662	34.920	19.507	1.00	0.00	O
ATOM	776	OE2	GLU	A	70	37.792	34.719	20.040	1.00	0.00	O
ATOM	777	C	GLU	A	70	38.437	39.301	20.105	1.00	0.00	C
ATOM	778	O	GLU	A	70	38.757	40.169	20.917	1.00	0.00	O
ATOM	779	N	TRP	A	71	38.249	39.598	18.811	1.00	0.00	N
ATOM	780	H	TRP	A	71	37.787	38.936	18.221	1.00	0.00	H
ATOM	781	CA	TRP	A	71	38.707	40.879	18.234	1.00	0.00	C
ATOM	782	HA	TRP	A	71	39.668	40.924	18.505	1.00	0.00	H
ATOM	783	CB	TRP	A	71	38.580	40.838	16.700	1.00	0.00	C
ATOM	784	HB1	TRP	A	71	38.712	39.892	16.405	1.00	0.00	H
ATOM	785	HB2	TRP	A	71	39.303	41.411	16.315	1.00	0.00	H
ATOM	786	CG	TRP	A	71	37.285	41.306	16.095	1.00	0.00	C
ATOM	787	CD1	TRP	A	71	36.111	40.632	16.082	1.00	0.00	C
ATOM	788	HD1	TRP	A	71	35.959	39.732	16.491	1.00	0.00	H
ATOM	789	NE1	TRP	A	71	35.153	41.376	15.418	1.00	0.00	N
ATOM	790	HE1	TRP	A	71	34.216	41.068	15.253	1.00	0.00	H
ATOM	791	CE2	TRP	A	71	35.652	42.595	15.013	1.00	0.00	C
ATOM	792	CZ2	TRP	A	71	35.082	43.695	14.359	1.00	0.00	C
ATOM	793	HZ2	TRP	A	71	34.108	43.697	14.131	1.00	0.00	H
ATOM	794	CH2	TRP	A	71	35.891	44.795	14.027	1.00	0.00	C
ATOM	795	HH2	TRP	A	71	35.497	45.582	13.553	1.00	0.00	H
ATOM	796	CZ3	TRP	A	71	37.256	44.785	14.364	1.00	0.00	C
ATOM	797	HZ3	TRP	A	71	37.835	45.562	14.116	1.00	0.00	H
ATOM	798	CE3	TRP	A	71	37.813	43.686	15.050	1.00	0.00	C
ATOM	799	HE3	TRP	A	71	38.782	43.700	15.296	1.00	0.00	H
ATOM	800	CD2	TRP	A	71	37.026	42.565	15.396	1.00	0.00	C
ATOM	801	C	TRP	A	71	38.055	42.143	18.842	1.00	0.00	C
ATOM	802	O	TRP	A	71	38.652	43.222	18.819	1.00	0.00	O
ATOM	803	N	ASN	A	72	36.869	41.992	19.449	1.00	0.00	N
ATOM	804	H	ASN	A	72	36.466	41.077	19.452	1.00	0.00	H
ATOM	805	CA	ASN	A	72	36.116	43.065	20.109	1.00	0.00	C
ATOM	806	HA	ASN	A	72	36.419	43.904	19.657	1.00	0.00	H
ATOM	807	CB	ASN	A	72	34.645	42.822	19.903	1.00	0.00	C

ATOM	808	HB1	ASN	A	72	34.134	43.470	20.468	1.00	0.00	H
ATOM	809	HB2	ASN	A	72	34.434	41.889	20.194	1.00	0.00	H
ATOM	810	CG	ASN	A	72	34.246	43.002	18.427	1.00	0.00	C
ATOM	811	OD1	ASN	A	72	34.844	43.792	17.712	1.00	0.00	O
ATOM	812	ND2	ASN	A	72	33.138	42.433	18.026	1.00	0.00	N
ATOM	813	HD21	ASN	A	72	32.585	41.905	18.671	1.00	0.00	H
ATOM	814	HD22	ASN	A	72	32.844	42.526	17.075	1.00	0.00	H
ATOM	815	C	ASN	A	72	36.406	43.196	21.614	1.00	0.00	C
ATOM	816	O	ASN	A	72	35.901	44.178	22.266	1.00	0.00	O
ATOM	817	N	ASP	A	73	37.218	42.299	22.282	1.00	0.00	N
ATOM	818	H	ASP	A	73	37.525	41.504	21.760	1.00	0.00	H
ATOM	819	CA	ASP	A	73	37.672	42.382	23.648	1.00	0.00	C
ATOM	820	HA	ASP	A	73	36.838	42.096	24.119	1.00	0.00	H
ATOM	821	CB	ASP	A	73	38.807	41.435	23.946	1.00	0.00	C
ATOM	822	HB1	ASP	A	73	39.250	41.710	24.800	1.00	0.00	H
ATOM	823	HB2	ASP	A	73	39.471	41.472	23.199	1.00	0.00	H
ATOM	824	CG	ASP	A	73	38.274	40.019	24.083	1.00	0.00	C
ATOM	825	OD1	ASP	A	73	37.044	39.772	24.201	1.00	0.00	O
ATOM	826	OD2	ASP	A	73	39.134	39.137	24.273	1.00	0.00	O
ATOM	827	C	ASP	A	73	38.029	43.768	24.130	1.00	0.00	C
ATOM	828	O	ASP	A	73	37.454	44.247	25.078	1.00	0.00	O
ATOM	829	N	GLU	A	74	39.154	44.373	23.686	1.00	0.00	N
ATOM	830	H	GLU	A	74	39.622	43.988	22.891	1.00	0.00	H
ATOM	831	CA	GLU	A	74	39.710	45.554	24.312	1.00	0.00	C
ATOM	832	HA	GLU	A	74	39.826	45.217	25.247	1.00	0.00	H
ATOM	833	CB	GLU	A	74	41.124	45.878	23.970	1.00	0.00	C
ATOM	834	HB1	GLU	A	74	41.693	45.104	24.248	1.00	0.00	H
ATOM	835	HB2	GLU	A	74	41.388	46.690	24.490	1.00	0.00	H
ATOM	836	CG	GLU	A	74	41.315	46.145	22.457	1.00	0.00	C
ATOM	837	HG1	GLU	A	74	40.586	46.758	22.152	1.00	0.00	H
ATOM	838	HG2	GLU	A	74	41.241	45.273	21.973	1.00	0.00	H
ATOM	839	CD	GLU	A	74	42.617	46.770	22.037	1.00	0.00	C
ATOM	840	OE1	GLU	A	74	43.004	47.798	22.611	1.00	0.00	O
ATOM	841	OE2	GLU	A	74	43.264	46.279	21.038	1.00	0.00	O
ATOM	842	C	GLU	A	74	38.714	46.813	24.164	1.00	0.00	C
ATOM	843	O	GLU	A	74	38.852	47.843	24.781	1.00	0.00	O
ATOM	844	N	THR	A	75	37.721	46.648	23.268	1.00	0.00	N
ATOM	845	H	THR	A	75	37.829	45.927	22.584	1.00	0.00	H
ATOM	846	CA	THR	A	75	36.493	47.450	23.223	1.00	0.00	C
ATOM	847	HA	THR	A	75	36.789	48.389	23.400	1.00	0.00	H
ATOM	848	CB	THR	A	75	35.865	47.452	21.821	1.00	0.00	C
ATOM	849	HB	THR	A	75	35.455	46.557	21.648	1.00	0.00	H
ATOM	850	CG2	THR	A	75	34.870	48.538	21.679	1.00	0.00	C
ATOM	851	HG21	THR	A	75	34.479	48.514	20.759	1.00	0.00	H
ATOM	852	HG22	THR	A	75	34.142	48.413	22.353	1.00	0.00	H
ATOM	853	HG23	THR	A	75	35.316	49.420	21.829	1.00	0.00	H
ATOM	854	OG1	THR	A	75	36.773	47.549	20.789	1.00	0.00	O
ATOM	855	HG1	THR	A	75	36.286	47.544	19.915	1.00	0.00	H
ATOM	856	C	THR	A	75	35.514	47.046	24.353	1.00	0.00	C
ATOM	857	O	THR	A	75	35.005	47.866	25.065	1.00	0.00	O
ATOM	858	N	LEU	A	76	35.123	45.806	24.385	1.00	0.00	N
ATOM	859	H	LEU	A	76	35.588	45.132	23.811	1.00	0.00	H
ATOM	860	CA	LEU	A	76	34.006	45.370	25.251	1.00	0.00	C
ATOM	861	HA	LEU	A	76	33.333	46.101	25.134	1.00	0.00	H

ATOM	862	CB	LEU	A	76	33.441	44.029	24.789	1.00	0.00	C
ATOM	863	HB1	LEU	A	76	32.708	43.782	25.423	1.00	0.00	H
ATOM	864	HB2	LEU	A	76	34.179	43.358	24.855	1.00	0.00	H
ATOM	865	CG	LEU	A	76	32.869	43.939	23.374	1.00	0.00	C
ATOM	866	HG	LEU	A	76	33.659	44.084	22.779	1.00	0.00	H
ATOM	867	CD1	LEU	A	76	32.117	42.550	23.185	1.00	0.00	C
ATOM	868	HD11	LEU	A	76	31.745	42.496	22.258	1.00	0.00	H
ATOM	869	HD12	LEU	A	76	32.762	41.799	23.330	1.00	0.00	H
ATOM	870	HD13	LEU	A	76	31.372	42.482	23.848	1.00	0.00	H
ATOM	871	CD2	LEU	A	76	31.868	45.021	23.037	1.00	0.00	C
ATOM	872	HD21	LEU	A	76	31.542	44.894	22.100	1.00	0.00	H
ATOM	873	HD22	LEU	A	76	31.095	44.968	23.669	1.00	0.00	H
ATOM	874	HD23	LEU	A	76	32.305	45.917	23.119	1.00	0.00	H
ATOM	875	C	LEU	A	76	34.402	45.245	26.744	1.00	0.00	C
ATOM	876	O	LEU	A	76	33.458	45.371	27.575	1.00	0.00	O
ATOM	877	N	TYR	A	77	35.657	44.972	27.141	1.00	0.00	N
ATOM	878	H	TYR	A	77	36.284	44.657	26.429	1.00	0.00	H
ATOM	879	CA	TYR	A	77	36.236	45.084	28.535	1.00	0.00	C
ATOM	880	HA	TYR	A	77	35.871	44.360	29.120	1.00	0.00	H
ATOM	881	CB	TYR	A	77	37.730	44.858	28.522	1.00	0.00	C
ATOM	882	HB1	TYR	A	77	38.028	44.985	29.468	1.00	0.00	H
ATOM	883	HB2	TYR	A	77	38.107	45.589	27.953	1.00	0.00	H
ATOM	884	CG	TYR	A	77	38.402	43.587	28.050	1.00	0.00	C
ATOM	885	CD1	TYR	A	77	37.761	42.358	28.241	1.00	0.00	C
ATOM	886	HD1	TYR	A	77	36.812	42.333	28.556	1.00	0.00	H
ATOM	887	CE1	TYR	A	77	38.450	41.155	27.990	1.00	0.00	C
ATOM	888	HE1	TYR	A	77	37.950	40.290	28.040	1.00	0.00	H
ATOM	889	CZ	TYR	A	77	39.820	41.134	27.671	1.00	0.00	C
ATOM	890	OH	TYR	A	77	40.427	40.000	27.205	1.00	0.00	O
ATOM	891	HH	TYR	A	77	41.395	40.185	27.037	1.00	0.00	H
ATOM	892	CE2	TYR	A	77	40.490	42.377	27.504	1.00	0.00	C
ATOM	893	HE2	TYR	A	77	41.455	42.399	27.244	1.00	0.00	H
ATOM	894	CD2	TYR	A	77	39.779	43.582	27.709	1.00	0.00	C
ATOM	895	HD2	TYR	A	77	40.259	44.454	27.611	1.00	0.00	H
ATOM	896	C	TYR	A	77	35.844	46.460	29.103	1.00	0.00	C
ATOM	897	O	TYR	A	77	35.376	46.601	30.257	1.00	0.00	O
ATOM	898	N	GLN	A	78	36.024	47.555	28.295	1.00	0.00	N
ATOM	899	H	GLN	A	78	36.349	47.443	27.356	1.00	0.00	H
ATOM	900	CA	GLN	A	78	35.742	48.851	28.810	1.00	0.00	C
ATOM	901	HA	GLN	A	78	36.168	48.835	29.714	1.00	0.00	H
ATOM	902	CB	GLN	A	78	36.332	49.855	27.804	1.00	0.00	C
ATOM	903	HB1	GLN	A	78	36.343	50.747	28.257	1.00	0.00	H
ATOM	904	HB2	GLN	A	78	35.707	49.885	27.024	1.00	0.00	H
ATOM	905	CG	GLN	A	78	37.737	49.635	27.233	1.00	0.00	C
ATOM	906	HG1	GLN	A	78	37.785	50.043	26.321	1.00	0.00	H
ATOM	907	HG2	GLN	A	78	37.912	48.652	27.169	1.00	0.00	H
ATOM	908	CD	GLN	A	78	38.808	50.279	28.129	1.00	0.00	C
ATOM	909	OE1	GLN	A	78	39.712	51.081	27.609	1.00	0.00	O
ATOM	910	NE2	GLN	A	78	38.779	50.289	29.372	1.00	0.00	N
ATOM	911	HE21	GLN	A	78	38.019	49.857	29.858	1.00	0.00	H
ATOM	912	HE22	GLN	A	78	39.515	50.729	29.886	1.00	0.00	H
ATOM	913	C	GLN	A	78	34.196	49.168	28.901	1.00	0.00	C
ATOM	914	O	GLN	A	78	33.766	49.874	29.835	1.00	0.00	O
ATOM	915	N	GLU	A	79	33.366	48.678	27.943	1.00	0.00	N

ATOM	916	H	GLU	A	79	33.771	48.237	27.142	1.00	0.00	H
ATOM	917	CA	GLU	A	79	31.905	48.766	28.029	1.00	0.00	C
ATOM	918	HA	GLU	A	79	31.658	49.714	28.229	1.00	0.00	H
ATOM	919	CB	GLU	A	79	31.320	48.329	26.656	1.00	0.00	C
ATOM	920	HB1	GLU	A	79	30.321	48.318	26.706	1.00	0.00	H
ATOM	921	HB2	GLU	A	79	31.652	47.414	26.426	1.00	0.00	H
ATOM	922	CG	GLU	A	79	31.670	49.191	25.536	1.00	0.00	C
ATOM	923	HG1	GLU	A	79	31.560	48.654	24.700	1.00	0.00	H
ATOM	924	HG2	GLU	A	79	32.631	49.447	25.644	1.00	0.00	H
ATOM	925	CD	GLU	A	79	30.808	50.454	25.453	1.00	0.00	C
ATOM	926	OE1	GLU	A	79	30.431	51.020	26.506	1.00	0.00	O
ATOM	927	OE2	GLU	A	79	30.456	50.864	24.294	1.00	0.00	O
ATOM	928	C	GLU	A	79	31.366	47.995	29.214	1.00	0.00	C
ATOM	929	O	GLU	A	79	30.442	48.462	29.857	1.00	0.00	O
ATOM	930	N	PHE	A	80	31.872	46.781	29.455	1.00	0.00	N
ATOM	931	H	PHE	A	80	32.478	46.401	28.756	1.00	0.00	H
ATOM	932	CA	PHE	A	80	31.626	45.939	30.655	1.00	0.00	C
ATOM	933	HA	PHE	A	80	30.658	45.695	30.719	1.00	0.00	H
ATOM	934	CB	PHE	A	80	32.390	44.631	30.481	1.00	0.00	C
ATOM	935	HB1	PHE	A	80	33.357	44.866	30.378	1.00	0.00	H
ATOM	936	HB2	PHE	A	80	32.059	44.206	29.638	1.00	0.00	H
ATOM	937	CG	PHE	A	80	32.319	43.572	31.550	1.00	0.00	C
ATOM	938	CD1	PHE	A	80	31.144	42.822	31.687	1.00	0.00	C
ATOM	939	HD1	PHE	A	80	30.374	42.922	31.056	1.00	0.00	H
ATOM	940	CE1	PHE	A	80	31.111	41.875	32.819	1.00	0.00	C
ATOM	941	HE1	PHE	A	80	30.303	41.304	32.965	1.00	0.00	H
ATOM	942	CZ	PHE	A	80	32.189	41.773	33.678	1.00	0.00	C
ATOM	943	HZ	PHE	A	80	32.137	41.121	34.435	1.00	0.00	H
ATOM	944	CE2	PHE	A	80	33.350	42.542	33.535	1.00	0.00	C
ATOM	945	HE2	PHE	A	80	34.125	42.452	34.160	1.00	0.00	H
ATOM	946	CD2	PHE	A	80	33.361	43.451	32.458	1.00	0.00	C
ATOM	947	HD2	PHE	A	80	34.161	44.039	32.342	1.00	0.00	H
ATOM	948	C	PHE	A	80	31.973	46.747	31.939	1.00	0.00	C
ATOM	949	O	PHE	A	80	31.171	46.871	32.891	1.00	0.00	O
ATOM	950	N	ASN	A	81	33.215	47.232	32.069	1.00	0.00	N
ATOM	951	H	ASN	A	81	33.863	47.076	31.323	1.00	0.00	H
ATOM	952	CA	ASN	A	81	33.690	47.996	33.270	1.00	0.00	C
ATOM	953	HA	ASN	A	81	33.313	47.487	34.043	1.00	0.00	H
ATOM	954	CB	ASN	A	81	35.281	48.055	33.270	1.00	0.00	C
ATOM	955	HB1	ASN	A	81	35.619	48.603	34.035	1.00	0.00	H
ATOM	956	HB2	ASN	A	81	35.625	48.434	32.411	1.00	0.00	H
ATOM	957	CG	ASN	A	81	35.775	46.621	33.421	1.00	0.00	C
ATOM	958	OD1	ASN	A	81	35.239	45.692	34.038	1.00	0.00	O
ATOM	959	ND2	ASN	A	81	36.950	46.383	32.836	1.00	0.00	N
ATOM	960	HD21	ASN	A	81	37.425	47.118	32.352	1.00	0.00	H
ATOM	961	HD22	ASN	A	81	37.355	45.470	32.881	1.00	0.00	H
ATOM	962	C	ASN	A	81	33.113	49.429	33.366	1.00	0.00	C
ATOM	963	O	ASN	A	81	33.040	49.999	34.464	1.00	0.00	O
ATOM	964	N	GLY	A	82	32.602	49.958	32.234	1.00	0.00	N
ATOM	965	H	GLY	A	82	32.797	49.501	31.366	1.00	0.00	H
ATOM	966	CA	GLY	A	82	31.807	51.123	32.213	1.00	0.00	C
ATOM	967	HA1	GLY	A	82	31.790	51.407	31.254	1.00	0.00	H
ATOM	968	HA2	GLY	A	82	32.318	51.794	32.751	1.00	0.00	H
ATOM	969	C	GLY	A	82	30.365	51.036	32.744	1.00	0.00	C

ATOM	970	O	GLY	A	82	29.895	52.102	33.151	1.00	0.00	O
ATOM	971	N	LEU	A	83	29.769	49.836	32.935	1.00	0.00	N
ATOM	972	H	LEU	A	83	30.169	49.045	32.471	1.00	0.00	H
ATOM	973	CA	LEU	A	83	28.596	49.604	33.759	1.00	0.00	C
ATOM	974	HA	LEU	A	83	27.835	50.039	33.277	1.00	0.00	H
ATOM	975	CB	LEU	A	83	28.370	48.111	33.857	1.00	0.00	C
ATOM	976	HB1	LEU	A	83	29.096	47.720	34.422	1.00	0.00	H
ATOM	977	HB2	LEU	A	83	28.421	47.725	32.936	1.00	0.00	H
ATOM	978	CG	LEU	A	83	26.975	47.786	34.490	1.00	0.00	C
ATOM	979	HG	LEU	A	83	27.005	48.250	35.376	1.00	0.00	H
ATOM	980	CD1	LEU	A	83	25.739	48.258	33.763	1.00	0.00	C
ATOM	981	HD11	LEU	A	83	24.925	47.984	34.276	1.00	0.00	H
ATOM	982	HD12	LEU	A	83	25.761	49.254	33.681	1.00	0.00	H
ATOM	983	HD13	LEU	A	83	25.712	47.849	32.851	1.00	0.00	H
ATOM	984	CD2	LEU	A	83	26.773	46.323	34.578	1.00	0.00	C
ATOM	985	HD21	LEU	A	83	25.879	46.134	34.984	1.00	0.00	H
ATOM	986	HD22	LEU	A	83	26.814	45.925	33.661	1.00	0.00	H
ATOM	987	HD23	LEU	A	83	27.490	45.921	35.148	1.00	0.00	H
ATOM	988	C	LEU	A	83	28.633	50.303	35.108	1.00	0.00	C
ATOM	989	O	LEU	A	83	27.558	50.887	35.450	1.00	0.00	O
ATOM	990	N	LYS	A	84	29.788	50.340	35.832	1.00	0.00	N
ATOM	991	H	LYS	A	84	30.633	50.020	35.403	1.00	0.00	H
ATOM	992	CA	LYS	A	84	29.843	50.820	37.188	1.00	0.00	C
ATOM	993	HA	LYS	A	84	29.058	50.413	37.656	1.00	0.00	H
ATOM	994	CB	LYS	A	84	31.239	50.369	37.721	1.00	0.00	C
ATOM	995	HB1	LYS	A	84	31.326	50.688	38.665	1.00	0.00	H
ATOM	996	HB2	LYS	A	84	31.940	50.804	37.156	1.00	0.00	H
ATOM	997	CG	LYS	A	84	31.510	48.841	37.714	1.00	0.00	C
ATOM	998	HG1	LYS	A	84	31.508	48.500	36.774	1.00	0.00	H
ATOM	999	HG2	LYS	A	84	30.804	48.369	38.241	1.00	0.00	H
ATOM	1000	CD	LYS	A	84	32.858	48.549	38.335	1.00	0.00	C
ATOM	1001	HD1	LYS	A	84	32.856	47.605	38.666	1.00	0.00	H
ATOM	1002	HD2	LYS	A	84	32.993	49.172	39.105	1.00	0.00	H
ATOM	1003	CE	LYS	A	84	34.019	48.710	37.399	1.00	0.00	C
ATOM	1004	HE1	LYS	A	84	34.061	49.658	37.082	1.00	0.00	H
ATOM	1005	HE2	LYS	A	84	33.900	48.101	36.615	1.00	0.00	H
ATOM	1006	NZ	LYS	A	84	35.290	48.388	38.033	1.00	0.00	N
ATOM	1007	HZ1	LYS	A	84	36.032	48.509	37.373	1.00	0.00	H
ATOM	1008	HZ2	LYS	A	84	35.436	48.995	38.814	1.00	0.00	H
ATOM	1009	HZ3	LYS	A	84	35.275	47.439	38.347	1.00	0.00	H
ATOM	1010	C	LYS	A	84	29.642	52.342	37.331	1.00	0.00	C
ATOM	1011	O	LYS	A	84	29.454	52.812	38.450	1.00	0.00	O
ATOM	1012	N	LYS	A	85	29.715	53.162	36.253	1.00	0.00	N
ATOM	1013	H	LYS	A	85	30.070	52.799	35.392	1.00	0.00	H
ATOM	1014	CA	LYS	A	85	29.281	54.593	36.311	1.00	0.00	C
ATOM	1015	HA	LYS	A	85	29.836	55.116	36.957	1.00	0.00	H
ATOM	1016	CB	LYS	A	85	29.573	55.212	34.969	1.00	0.00	C
ATOM	1017	HB1	LYS	A	85	29.071	54.697	34.274	1.00	0.00	H
ATOM	1018	HB2	LYS	A	85	30.555	55.134	34.798	1.00	0.00	H
ATOM	1019	CG	LYS	A	85	29.140	56.726	34.935	1.00	0.00	C
ATOM	1020	HG1	LYS	A	85	29.479	57.181	35.759	1.00	0.00	H
ATOM	1021	HG2	LYS	A	85	28.142	56.779	34.911	1.00	0.00	H
ATOM	1022	CD	LYS	A	85	29.713	57.465	33.681	1.00	0.00	C
ATOM	1023	HD1	LYS	A	85	29.243	58.343	33.593	1.00	0.00	H

ATOM	1024	HD2	LYS	A	85	29.526	56.904	32.874	1.00	0.00	H
ATOM	1025	CE	LYS	A	85	31.206	57.759	33.681	1.00	0.00	C
ATOM	1026	HE1	LYS	A	85	31.712	56.900	33.760	1.00	0.00	H
ATOM	1027	HE2	LYS	A	85	31.426	58.348	34.458	1.00	0.00	H
ATOM	1028	NZ	LYS	A	85	31.679	58.432	32.478	1.00	0.00	N
ATOM	1029	HZ1	LYS	A	85	32.664	58.591	32.550	1.00	0.00	H
ATOM	1030	HZ2	LYS	A	85	31.492	57.859	31.680	1.00	0.00	H
ATOM	1031	HZ3	LYS	A	85	31.206	59.308	32.378	1.00	0.00	H
ATOM	1032	C	LYS	A	85	27.810	54.703	36.844	1.00	0.00	C
ATOM	1033	O	LYS	A	85	27.528	55.672	37.535	1.00	0.00	O
ATOM	1034	N	MET	A	86	26.875	53.833	36.415	1.00	0.00	N
ATOM	1035	H	MET	A	86	27.165	53.124	35.772	1.00	0.00	H
ATOM	1036	CA	MET	A	86	25.496	53.844	36.808	1.00	0.00	C
ATOM	1037	HA	MET	A	86	25.325	54.829	36.795	1.00	0.00	H
ATOM	1038	CB	MET	A	86	24.656	53.068	35.779	1.00	0.00	C
ATOM	1039	HB1	MET	A	86	23.686	53.236	35.957	1.00	0.00	H
ATOM	1040	HB2	MET	A	86	24.847	52.091	35.873	1.00	0.00	H
ATOM	1041	CG	MET	A	86	24.945	53.465	34.356	1.00	0.00	C
ATOM	1042	HG1	MET	A	86	25.944	53.441	34.311	1.00	0.00	H
ATOM	1043	HG2	MET	A	86	24.633	54.414	34.316	1.00	0.00	H
ATOM	1044	SD	MET	A	86	24.207	52.478	33.060	1.00	0.00	S
ATOM	1045	CE	MET	A	86	22.421	52.646	33.431	1.00	0.00	C
ATOM	1046	HE1	MET	A	86	21.891	52.127	32.760	1.00	0.00	H
ATOM	1047	HE2	MET	A	86	22.236	52.292	34.348	1.00	0.00	H
ATOM	1048	HE3	MET	A	86	22.161	53.611	33.387	1.00	0.00	H
ATOM	1049	C	MET	A	86	25.197	53.248	38.248	1.00	0.00	C
ATOM	1050	O	MET	A	86	24.031	53.292	38.697	1.00	0.00	O
ATOM	1051	N	ASN	A	87	26.225	52.648	38.893	1.00	0.00	N
ATOM	1052	H	ASN	A	87	27.089	52.586	38.394	1.00	0.00	H
ATOM	1053	CA	ASN	A	87	26.207	52.073	40.259	1.00	0.00	C
ATOM	1054	HA	ASN	A	87	25.761	52.784	40.803	1.00	0.00	H
ATOM	1055	CB	ASN	A	87	25.337	50.789	40.405	1.00	0.00	C
ATOM	1056	HB1	ASN	A	87	25.572	50.140	39.681	1.00	0.00	H
ATOM	1057	HB2	ASN	A	87	24.369	51.030	40.336	1.00	0.00	H
ATOM	1058	CG	ASN	A	87	25.561	50.114	41.733	1.00	0.00	C
ATOM	1059	OD1	ASN	A	87	25.831	50.742	42.709	1.00	0.00	O
ATOM	1060	ND2	ASN	A	87	25.413	48.831	41.921	1.00	0.00	N
ATOM	1061	HD21	ASN	A	87	25.137	48.243	41.161	1.00	0.00	H
ATOM	1062	HD22	ASN	A	87	25.575	48.436	42.825	1.00	0.00	H
ATOM	1063	C	ASN	A	87	27.676	51.797	40.726	1.00	0.00	C
ATOM	1064	O	ASN	A	87	28.227	50.731	40.362	1.00	0.00	O
ATOM	1065	N	PRO	A	88	28.331	52.690	41.526	1.00	0.00	N
ATOM	1066	CD	PRO	A	88	27.753	53.933	42.095	1.00	0.00	C
ATOM	1067	HD1	PRO	A	88	26.895	53.736	42.569	1.00	0.00	H
ATOM	1068	HD2	PRO	A	88	27.588	54.605	41.373	1.00	0.00	H
ATOM	1069	CG	PRO	A	88	28.742	54.448	43.043	1.00	0.00	C
ATOM	1070	HG1	PRO	A	88	28.539	54.115	43.964	1.00	0.00	H
ATOM	1071	HG2	PRO	A	88	28.731	55.448	43.040	1.00	0.00	H
ATOM	1072	CB	PRO	A	88	30.086	53.859	42.473	1.00	0.00	C
ATOM	1073	HB1	PRO	A	88	30.763	53.735	43.199	1.00	0.00	H
ATOM	1074	HB2	PRO	A	88	30.466	54.449	41.760	1.00	0.00	H
ATOM	1075	CA	PRO	A	88	29.719	52.516	41.882	1.00	0.00	C
ATOM	1076	HA	PRO	A	88	30.277	52.299	41.081	1.00	0.00	H
ATOM	1077	C	PRO	A	88	29.973	51.297	42.747	1.00	0.00	C

ATOM	1078	O	PRO	A	88	31.157	50.924	42.843	1.00	0.00	O
ATOM	1079	N	LYS	A	89	28.913	50.611	43.280	1.00	0.00	N
ATOM	1080	H	LYS	A	89	27.982	50.900	43.057	1.00	0.00	H
ATOM	1081	CA	LYS	A	89	29.115	49.452	44.178	1.00	0.00	C
ATOM	1082	HA	LYS	A	89	29.997	49.544	44.640	1.00	0.00	H
ATOM	1083	CB	LYS	A	89	27.861	49.427	45.155	1.00	0.00	C
ATOM	1084	HB1	LYS	A	89	27.086	49.032	44.662	1.00	0.00	H
ATOM	1085	HB2	LYS	A	89	27.646	50.368	45.415	1.00	0.00	H
ATOM	1086	CG	LYS	A	89	27.985	48.642	46.457	1.00	0.00	C
ATOM	1087	HG1	LYS	A	89	28.457	47.782	46.262	1.00	0.00	H
ATOM	1088	HG2	LYS	A	89	27.064	48.448	46.796	1.00	0.00	H
ATOM	1089	CD	LYS	A	89	28.769	49.407	47.547	1.00	0.00	C
ATOM	1090	HD1	LYS	A	89	28.283	50.234	47.829	1.00	0.00	H
ATOM	1091	HD2	LYS	A	89	29.685	49.651	47.229	1.00	0.00	H
ATOM	1092	CE	LYS	A	89	28.831	48.372	48.698	1.00	0.00	C
ATOM	1093	HE1	LYS	A	89	29.275	47.539	48.368	1.00	0.00	H
ATOM	1094	HE2	LYS	A	89	27.902	48.158	48.998	1.00	0.00	H
ATOM	1095	NZ	LYS	A	89	29.563	48.783	49.890	1.00	0.00	N
ATOM	1096	HZ1	LYS	A	89	29.541	48.046	50.565	1.00	0.00	H
ATOM	1097	HZ2	LYS	A	89	30.512	48.983	49.646	1.00	0.00	H
ATOM	1098	HZ3	LYS	A	89	29.138	49.602	50.276	1.00	0.00	H
ATOM	1099	C	LYS	A	89	29.224	48.178	43.393	1.00	0.00	C
ATOM	1100	O	LYS	A	89	29.740	47.239	43.937	1.00	0.00	O
ATOM	1101	N	LEU	A	90	28.848	48.162	42.151	1.00	0.00	N
ATOM	1102	H	LEU	A	90	28.472	48.981	41.717	1.00	0.00	H
ATOM	1103	CA	LEU	A	90	28.993	46.883	41.388	1.00	0.00	C
ATOM	1104	HA	LEU	A	90	28.542	46.213	41.977	1.00	0.00	H
ATOM	1105	CB	LEU	A	90	28.251	46.949	39.991	1.00	0.00	C
ATOM	1106	HB1	LEU	A	90	28.647	47.701	39.464	1.00	0.00	H
ATOM	1107	HB2	LEU	A	90	27.283	47.135	40.161	1.00	0.00	H
ATOM	1108	CG	LEU	A	90	28.331	45.667	39.107	1.00	0.00	C
ATOM	1109	HG	LEU	A	90	29.260	45.363	38.895	1.00	0.00	H
ATOM	1110	CD1	LEU	A	90	27.644	44.529	39.873	1.00	0.00	C
ATOM	1111	HD11	LEU	A	90	27.680	43.691	39.328	1.00	0.00	H
ATOM	1112	HD12	LEU	A	90	28.114	44.381	40.743	1.00	0.00	H
ATOM	1113	HD13	LEU	A	90	26.690	44.773	40.047	1.00	0.00	H
ATOM	1114	CD2	LEU	A	90	27.693	45.983	37.777	1.00	0.00	C
ATOM	1115	HD21	LEU	A	90	27.731	45.175	37.189	1.00	0.00	H
ATOM	1116	HD22	LEU	A	90	26.739	46.247	37.919	1.00	0.00	H
ATOM	1117	HD23	LEU	A	90	28.186	46.736	37.341	1.00	0.00	H
ATOM	1118	C	LEU	A	90	30.542	46.521	41.158	1.00	0.00	C
ATOM	1119	O	LEU	A	90	31.388	47.329	40.851	1.00	0.00	O
ATOM	1120	N	LYS	A	91	30.936	45.257	41.363	1.00	0.00	N
ATOM	1121	H	LYS	A	91	30.286	44.616	41.772	1.00	0.00	H
ATOM	1122	CA	LYS	A	91	32.321	44.751	41.005	1.00	0.00	C
ATOM	1123	HA	LYS	A	91	32.883	45.578	40.992	1.00	0.00	H
ATOM	1124	CB	LYS	A	91	32.946	43.824	42.042	1.00	0.00	C
ATOM	1125	HB1	LYS	A	91	33.693	43.335	41.591	1.00	0.00	H
ATOM	1126	HB2	LYS	A	91	32.241	43.172	42.321	1.00	0.00	H
ATOM	1127	CG	LYS	A	91	33.512	44.515	43.322	1.00	0.00	C
ATOM	1128	HG1	LYS	A	91	34.118	45.254	43.029	1.00	0.00	H
ATOM	1129	HG2	LYS	A	91	34.039	43.834	43.831	1.00	0.00	H
ATOM	1130	CD	LYS	A	91	32.560	45.128	44.313	1.00	0.00	C
ATOM	1131	HD1	LYS	A	91	32.161	45.944	43.894	1.00	0.00	H

ATOM	1132	HD2	LYS	A	91	33.084	45.391	45.123	1.00	0.00	H
ATOM	1133	CE	LYS	A	91	31.425	44.166	44.733	1.00	0.00	C
ATOM	1134	HE1	LYS	A	91	31.769	43.519	45.413	1.00	0.00	H
ATOM	1135	HE2	LYS	A	91	31.097	43.665	43.932	1.00	0.00	H
ATOM	1136	NZ	LYS	A	91	30.268	44.852	45.326	1.00	0.00	N
ATOM	1137	HZ1	LYS	A	91	29.572	44.178	45.575	1.00	0.00	H
ATOM	1138	HZ2	LYS	A	91	30.558	45.349	46.144	1.00	0.00	H
ATOM	1139	HZ3	LYS	A	91	29.886	45.496	44.663	1.00	0.00	H
ATOM	1140	C	LYS	A	91	32.345	44.089	39.627	1.00	0.00	C
ATOM	1141	O	LYS	A	91	31.340	43.433	39.297	1.00	0.00	O
ATOM	1142	N	THR	A	92	33.411	44.146	38.935	1.00	0.00	N
ATOM	1143	H	THR	A	92	34.217	44.597	39.320	1.00	0.00	H
ATOM	1144	CA	THR	A	92	33.496	43.538	37.516	1.00	0.00	C
ATOM	1145	HA	THR	A	92	32.809	42.821	37.398	1.00	0.00	H
ATOM	1146	CB	THR	A	92	33.256	44.680	36.489	1.00	0.00	C
ATOM	1147	HB	THR	A	92	33.471	44.261	35.607	1.00	0.00	H
ATOM	1148	CG2	THR	A	92	31.826	45.256	36.634	1.00	0.00	C
ATOM	1149	HG21	THR	A	92	31.692	45.988	35.966	1.00	0.00	H
ATOM	1150	HG22	THR	A	92	31.157	44.531	36.473	1.00	0.00	H
ATOM	1151	HG23	THR	A	92	31.706	45.621	37.557	1.00	0.00	H
ATOM	1152	OG1	THR	A	92	34.155	45.813	36.512	1.00	0.00	O
ATOM	1153	HG1	THR	A	92	33.886	46.466	35.804	1.00	0.00	H
ATOM	1154	C	THR	A	92	34.903	42.917	37.310	1.00	0.00	C
ATOM	1155	O	THR	A	92	35.856	43.601	37.041	1.00	0.00	O
ATOM	1156	N	LEU	A	93	35.008	41.582	37.384	1.00	0.00	N
ATOM	1157	H	LEU	A	93	34.159	41.088	37.573	1.00	0.00	H
ATOM	1158	CA	LEU	A	93	36.217	40.748	37.222	1.00	0.00	C
ATOM	1159	HA	LEU	A	93	36.936	41.401	37.459	1.00	0.00	H
ATOM	1160	CB	LEU	A	93	36.178	39.526	38.160	1.00	0.00	C
ATOM	1161	HB1	LEU	A	93	37.052	39.050	38.068	1.00	0.00	H
ATOM	1162	HB2	LEU	A	93	35.439	38.930	37.845	1.00	0.00	H
ATOM	1163	CG	LEU	A	93	35.945	39.839	39.680	1.00	0.00	C
ATOM	1164	HG	LEU	A	93	36.459	40.668	39.899	1.00	0.00	H
ATOM	1165	CD1	LEU	A	93	34.410	39.978	40.022	1.00	0.00	C
ATOM	1166	HD11	LEU	A	93	34.302	40.177	40.996	1.00	0.00	H
ATOM	1167	HD12	LEU	A	93	34.015	40.723	39.484	1.00	0.00	H
ATOM	1168	HD13	LEU	A	93	33.941	39.123	39.802	1.00	0.00	H
ATOM	1169	CD2	LEU	A	93	36.441	38.712	40.514	1.00	0.00	C
ATOM	1170	HD21	LEU	A	93	36.289	38.920	41.480	1.00	0.00	H
ATOM	1171	HD22	LEU	A	93	35.949	37.876	40.271	1.00	0.00	H
ATOM	1172	HD23	LEU	A	93	37.419	38.582	40.351	1.00	0.00	H
ATOM	1173	C	LEU	A	93	36.481	40.242	35.798	1.00	0.00	C
ATOM	1174	O	LEU	A	93	35.575	40.263	34.914	1.00	0.00	O
ATOM	1175	N	LEU	A	94	37.637	39.660	35.554	1.00	0.00	N
ATOM	1176	H	LEU	A	94	38.312	39.673	36.291	1.00	0.00	H
ATOM	1177	CA	LEU	A	94	38.038	38.985	34.291	1.00	0.00	C
ATOM	1178	HA	LEU	A	94	37.290	39.089	33.636	1.00	0.00	H
ATOM	1179	CB	LEU	A	94	39.449	39.598	33.882	1.00	0.00	C
ATOM	1180	HB1	LEU	A	94	40.129	39.364	34.576	1.00	0.00	H
ATOM	1181	HB2	LEU	A	94	39.374	40.593	33.811	1.00	0.00	H
ATOM	1182	CG	LEU	A	94	40.034	39.173	32.622	1.00	0.00	C
ATOM	1183	HG	LEU	A	94	40.123	38.178	32.665	1.00	0.00	H
ATOM	1184	CD1	LEU	A	94	39.098	39.570	31.459	1.00	0.00	C
ATOM	1185	HD11	LEU	A	94	39.501	39.277	30.592	1.00	0.00	H

ATOM	1186	HD12	LEU	A	94	38.210	39.127	31.580	1.00	0.00	H
ATOM	1187	HD13	LEU	A	94	38.977	40.563	31.452	1.00	0.00	H
ATOM	1188	CD2	LEU	A	94	41.424	39.814	32.462	1.00	0.00	C
ATOM	1189	HD21	LEU	A	94	41.828	39.522	31.595	1.00	0.00	H
ATOM	1190	HD22	LEU	A	94	41.335	40.810	32.470	1.00	0.00	H
ATOM	1191	HD23	LEU	A	94	42.013	39.526	33.217	1.00	0.00	H
ATOM	1192	C	LEU	A	94	38.304	37.519	34.555	1.00	0.00	C
ATOM	1193	O	LEU	A	94	39.222	37.145	35.342	1.00	0.00	O
ATOM	1194	N	ALA	A	95	37.574	36.634	33.881	1.00	0.00	N
ATOM	1195	H	ALA	A	95	36.780	36.956	33.366	1.00	0.00	H
ATOM	1196	CA	ALA	A	95	37.892	35.212	33.868	1.00	0.00	C
ATOM	1197	HA	ALA	A	95	38.198	34.993	34.795	1.00	0.00	H
ATOM	1198	CB	ALA	A	95	36.647	34.376	33.548	1.00	0.00	C
ATOM	1199	HB1	ALA	A	95	36.889	33.406	33.545	1.00	0.00	H
ATOM	1200	HB2	ALA	A	95	35.946	34.542	34.242	1.00	0.00	H
ATOM	1201	HB3	ALA	A	95	36.294	34.635	32.649	1.00	0.00	H
ATOM	1202	C	ALA	A	95	39.030	34.930	32.887	1.00	0.00	C
ATOM	1203	O	ALA	A	95	39.102	35.523	31.805	1.00	0.00	O
ATOM	1204	N	ILE	A	96	39.861	33.956	33.246	1.00	0.00	N
ATOM	1205	H	ILE	A	96	39.770	33.583	34.169	1.00	0.00	H
ATOM	1206	CA	ILE	A	96	40.896	33.395	32.384	1.00	0.00	C
ATOM	1207	HA	ILE	A	96	41.029	34.027	31.620	1.00	0.00	H
ATOM	1208	CB	ILE	A	96	42.227	33.292	33.154	1.00	0.00	C
ATOM	1209	HB	ILE	A	96	42.068	32.708	33.950	1.00	0.00	H
ATOM	1210	CG2	ILE	A	96	43.307	32.679	32.237	1.00	0.00	C
ATOM	1211	HG21	ILE	A	96	44.170	32.612	32.737	1.00	0.00	H
ATOM	1212	HG22	ILE	A	96	43.019	31.767	31.946	1.00	0.00	H
ATOM	1213	HG23	ILE	A	96	43.431	33.261	31.433	1.00	0.00	H
ATOM	1214	CG1	ILE	A	96	42.693	34.594	33.761	1.00	0.00	C
ATOM	1215	HG11	ILE	A	96	43.605	34.423	34.134	1.00	0.00	H
ATOM	1216	HG12	ILE	A	96	42.061	34.801	34.508	1.00	0.00	H
ATOM	1217	CD	ILE	A	96	42.783	35.847	32.864	1.00	0.00	C
ATOM	1218	HD1	ILE	A	96	43.105	36.623	33.407	1.00	0.00	H
ATOM	1219	HD2	ILE	A	96	43.423	35.676	32.115	1.00	0.00	H
ATOM	1220	HD3	ILE	A	96	41.879	36.054	32.489	1.00	0.00	H
ATOM	1221	C	ILE	A	96	40.413	32.048	31.826	1.00	0.00	C
ATOM	1222	O	ILE	A	96	39.949	31.190	32.575	1.00	0.00	O
ATOM	1223	N	GLY	A	97	40.489	31.877	30.506	1.00	0.00	N
ATOM	1224	H	GLY	A	97	40.942	32.590	29.971	1.00	0.00	H
ATOM	1225	CA	GLY	A	97	39.956	30.718	29.783	1.00	0.00	C
ATOM	1226	HA1	GLY	A	97	39.050	30.511	30.152	1.00	0.00	H
ATOM	1227	HA2	GLY	A	97	40.567	29.943	29.944	1.00	0.00	H
ATOM	1228	C	GLY	A	97	39.857	31.001	28.282	1.00	0.00	C
ATOM	1229	O	GLY	A	97	40.593	31.840	27.766	1.00	0.00	O
ATOM	1230	N	GLY	A	98	38.927	30.355	27.572	1.00	0.00	N
ATOM	1231	H	GLY	A	98	38.312	29.724	28.045	1.00	0.00	H
ATOM	1232	CA	GLY	A	98	38.776	30.538	26.125	1.00	0.00	C
ATOM	1233	HA1	GLY	A	98	38.785	31.522	25.945	1.00	0.00	H
ATOM	1234	HA2	GLY	A	98	37.887	30.160	25.867	1.00	0.00	H
ATOM	1235	C	GLY	A	98	39.874	29.861	25.299	1.00	0.00	C
ATOM	1236	O	GLY	A	98	40.866	29.370	25.837	1.00	0.00	O
ATOM	1237	N	TRP	A	99	39.723	29.857	23.971	1.00	0.00	N
ATOM	1238	H	TRP	A	99	38.957	30.367	23.580	1.00	0.00	H
ATOM	1239	CA	TRP	A	99	40.628	29.140	23.052	1.00	0.00	C

ATOM	1240	HA	TRP	A	99	40.578	28.192	23.367	1.00	0.00	H
ATOM	1241	CB	TRP	A	99	40.115	29.306	21.611	1.00	0.00	C
ATOM	1242	HB1	TRP	A	99	39.140	29.082	21.596	1.00	0.00	H
ATOM	1243	HB2	TRP	A	99	40.613	28.667	21.024	1.00	0.00	H
ATOM	1244	CG	TRP	A	99	40.268	30.676	21.011	1.00	0.00	C
ATOM	1245	CD1	TRP	A	99	39.298	31.614	20.914	1.00	0.00	C
ATOM	1246	HD1	TRP	A	99	38.353	31.496	21.218	1.00	0.00	H
ATOM	1247	NE1	TRP	A	99	39.808	32.759	20.332	1.00	0.00	N
ATOM	1248	HE1	TRP	A	99	39.272	33.583	20.147	1.00	0.00	H
ATOM	1249	CE2	TRP	A	99	41.149	32.624	20.038	1.00	0.00	C
ATOM	1250	CZ2	TRP	A	99	42.113	33.489	19.497	1.00	0.00	C
ATOM	1251	HZ2	TRP	A	99	41.868	34.427	19.253	1.00	0.00	H
ATOM	1252	CH2	TRP	A	99	43.425	33.026	19.301	1.00	0.00	C
ATOM	1253	HH2	TRP	A	99	44.122	33.637	18.926	1.00	0.00	H
ATOM	1254	CZ3	TRP	A	99	43.756	31.701	19.634	1.00	0.00	C
ATOM	1255	HZ3	TRP	A	99	44.686	31.367	19.481	1.00	0.00	H
ATOM	1256	CE3	TRP	A	99	42.785	30.841	20.182	1.00	0.00	C
ATOM	1257	HE3	TRP	A	99	43.035	29.900	20.409	1.00	0.00	H
ATOM	1258	CD2	TRP	A	99	41.462	31.283	20.417	1.00	0.00	C
ATOM	1259	C	TRP	A	99	42.114	29.548	23.145	1.00	0.00	C
ATOM	1260	O	TRP	A	99	42.992	28.770	22.777	1.00	0.00	O
ATOM	1261	N	ASN	A	100	42.411	30.760	23.630	1.00	0.00	N
ATOM	1262	H	ASN	A	100	41.657	31.340	23.939	1.00	0.00	H
ATOM	1263	CA	ASN	A	100	43.774	31.288	23.735	1.00	0.00	C
ATOM	1264	HA	ASN	A	100	44.353	30.778	23.099	1.00	0.00	H
ATOM	1265	CB	ASN	A	100	43.734	32.766	23.277	1.00	0.00	C
ATOM	1266	HB1	ASN	A	100	43.376	33.317	24.031	1.00	0.00	H
ATOM	1267	HB2	ASN	A	100	43.118	32.834	22.492	1.00	0.00	H
ATOM	1268	CG	ASN	A	100	45.079	33.364	22.863	1.00	0.00	C
ATOM	1269	OD1	ASN	A	100	45.916	32.725	22.231	1.00	0.00	O
ATOM	1270	ND2	ASN	A	100	45.314	34.527	23.345	1.00	0.00	N
ATOM	1271	HD21	ASN	A	100	44.641	34.967	23.939	1.00	0.00	H
ATOM	1272	HD22	ASN	A	100	46.170	34.995	23.127	1.00	0.00	H
ATOM	1273	C	ASN	A	100	44.426	30.999	25.122	1.00	0.00	C
ATOM	1274	O	ASN	A	100	45.601	31.288	25.341	1.00	0.00	O
ATOM	1275	N	PHE	A	101	43.741	30.349	26.062	1.00	0.00	N
ATOM	1276	H	PHE	A	101	42.746	30.297	25.972	1.00	0.00	H
ATOM	1277	CA	PHE	A	101	44.370	29.702	27.227	1.00	0.00	C
ATOM	1278	HA	PHE	A	101	45.230	30.176	27.415	1.00	0.00	H
ATOM	1279	CB	PHE	A	101	43.421	29.830	28.424	1.00	0.00	C
ATOM	1280	HB1	PHE	A	101	42.612	29.269	28.247	1.00	0.00	H
ATOM	1281	HB2	PHE	A	101	43.150	30.789	28.511	1.00	0.00	H
ATOM	1282	CG	PHE	A	101	44.011	29.391	29.749	1.00	0.00	C
ATOM	1283	CD1	PHE	A	101	43.465	28.325	30.476	1.00	0.00	C
ATOM	1284	HD1	PHE	A	101	42.694	27.803	30.111	1.00	0.00	H
ATOM	1285	CE1	PHE	A	101	44.017	28.003	31.723	1.00	0.00	C
ATOM	1286	HE1	PHE	A	101	43.675	27.208	32.224	1.00	0.00	H
ATOM	1287	CZ	PHE	A	101	45.046	28.785	32.279	1.00	0.00	C
ATOM	1288	HZ	PHE	A	101	45.417	28.518	33.168	1.00	0.00	H
ATOM	1289	CE2	PHE	A	101	45.562	29.887	31.667	1.00	0.00	C
ATOM	1290	HE2	PHE	A	101	46.218	30.484	32.129	1.00	0.00	H
ATOM	1291	CD2	PHE	A	101	45.114	30.153	30.313	1.00	0.00	C
ATOM	1292	HD2	PHE	A	101	45.564	30.860	29.767	1.00	0.00	H
ATOM	1293	C	PHE	A	101	44.691	28.235	26.921	1.00	0.00	C

ATOM	1294	O	PHE	A	101	44.202	27.647	25.962	1.00	0.00	O
ATOM	1295	N	GLY	A	102	45.401	27.603	27.847	1.00	0.00	N
ATOM	1296	H	GLY	A	102	45.939	28.149	28.489	1.00	0.00	H
ATOM	1297	CA	GLY	A	102	45.433	26.152	27.972	1.00	0.00	C
ATOM	1298	HA1	GLY	A	102	45.545	25.760	27.059	1.00	0.00	H
ATOM	1299	HA2	GLY	A	102	44.562	25.850	28.360	1.00	0.00	H
ATOM	1300	C	GLY	A	102	46.550	25.693	28.848	1.00	0.00	C
ATOM	1301	O	GLY	A	102	47.540	26.311	29.279	1.00	0.00	O
ATOM	1302	N	THR	A	103	46.413	24.413	29.241	1.00	0.00	N
ATOM	1303	H	THR	A	103	45.637	23.899	28.876	1.00	0.00	H
ATOM	1304	CA	THR	A	103	47.320	23.710	30.170	1.00	0.00	C
ATOM	1305	HA	THR	A	103	47.340	24.252	31.010	1.00	0.00	H
ATOM	1306	CB	THR	A	103	46.742	22.326	30.463	1.00	0.00	C
ATOM	1307	HB	THR	A	103	45.759	22.437	30.609	1.00	0.00	H
ATOM	1308	CG2	THR	A	103	46.852	21.303	29.382	1.00	0.00	C
ATOM	1309	HG21	THR	A	103	46.441	20.446	29.691	1.00	0.00	H
ATOM	1310	HG22	THR	A	103	46.373	21.627	28.566	1.00	0.00	H
ATOM	1311	HG23	THR	A	103	47.816	21.151	29.164	1.00	0.00	H
ATOM	1312	OG1	THR	A	103	47.304	21.824	31.651	1.00	0.00	O
ATOM	1313	HG1	THR	A	103	46.925	20.918	31.842	1.00	0.00	H
ATOM	1314	C	THR	A	103	48.777	23.650	29.658	1.00	0.00	C
ATOM	1315	O	THR	A	103	49.668	23.782	30.520	1.00	0.00	O
ATOM	1316	N	GLN	A	104	49.076	23.608	28.361	1.00	0.00	N
ATOM	1317	H	GLN	A	104	48.338	23.553	27.689	1.00	0.00	H
ATOM	1318	CA	GLN	A	104	50.461	23.641	27.903	1.00	0.00	C
ATOM	1319	HA	GLN	A	104	50.993	23.078	28.536	1.00	0.00	H
ATOM	1320	CB	GLN	A	104	50.519	22.949	26.510	1.00	0.00	C
ATOM	1321	HB1	GLN	A	104	50.005	22.094	26.576	1.00	0.00	H
ATOM	1322	HB2	GLN	A	104	51.478	22.747	26.313	1.00	0.00	H
ATOM	1323	CG	GLN	A	104	49.916	23.827	25.312	1.00	0.00	C
ATOM	1324	HG1	GLN	A	104	50.097	23.402	24.425	1.00	0.00	H
ATOM	1325	HG2	GLN	A	104	50.289	24.755	25.320	1.00	0.00	H
ATOM	1326	CD	GLN	A	104	48.413	23.888	25.542	1.00	0.00	C
ATOM	1327	OE1	GLN	A	104	47.839	25.020	25.730	1.00	0.00	O
ATOM	1328	NE2	GLN	A	104	47.703	22.782	25.736	1.00	0.00	N
ATOM	1329	HE21	GLN	A	104	48.156	21.890	25.732	1.00	0.00	H
ATOM	1330	HE22	GLN	A	104	46.716	22.841	25.886	1.00	0.00	H
ATOM	1331	C	GLN	A	104	51.128	25.064	28.053	1.00	0.00	C
ATOM	1332	O	GLN	A	104	52.231	25.125	28.625	1.00	0.00	O
ATOM	1333	N	LYS	A	105	50.382	26.205	27.774	1.00	0.00	N
ATOM	1334	H	LYS	A	105	49.501	26.077	27.319	1.00	0.00	H
ATOM	1335	CA	LYS	A	105	50.780	27.578	28.091	1.00	0.00	C
ATOM	1336	HA	LYS	A	105	51.696	27.574	27.690	1.00	0.00	H
ATOM	1337	CB	LYS	A	105	49.849	28.648	27.469	1.00	0.00	C
ATOM	1338	HB1	LYS	A	105	50.213	29.550	27.702	1.00	0.00	H
ATOM	1339	HB2	LYS	A	105	48.940	28.541	27.872	1.00	0.00	H
ATOM	1340	CG	LYS	A	105	49.668	28.623	25.971	1.00	0.00	C
ATOM	1341	HG1	LYS	A	105	49.152	27.795	25.753	1.00	0.00	H
ATOM	1342	HG2	LYS	A	105	50.581	28.562	25.568	1.00	0.00	H
ATOM	1343	CD	LYS	A	105	48.940	29.824	25.330	1.00	0.00	C
ATOM	1344	HD1	LYS	A	105	49.501	30.646	25.426	1.00	0.00	H
ATOM	1345	HD2	LYS	A	105	48.061	29.965	25.786	1.00	0.00	H
ATOM	1346	CE	LYS	A	105	48.698	29.552	23.846	1.00	0.00	C
ATOM	1347	HE1	LYS	A	105	48.307	28.637	23.740	1.00	0.00	H

ATOM	1348	HE2	LYS	A	105	49.569	29.601	23.358	1.00	0.00	H
ATOM	1349	NZ	LYS	A	105	47.740	30.569	23.246	1.00	0.00	N
ATOM	1350	HZ1	LYS	A	105	47.602	30.365	22.277	1.00	0.00	H
ATOM	1351	HZ2	LYS	A	105	46.863	30.524	23.724	1.00	0.00	H
ATOM	1352	HZ3	LYS	A	105	48.125	31.487	23.342	1.00	0.00	H
ATOM	1353	C	LYS	A	105	50.836	27.887	29.587	1.00	0.00	C
ATOM	1354	O	LYS	A	105	51.803	28.514	30.013	1.00	0.00	O
ATOM	1355	N	PHE	A	106	49.917	27.405	30.412	1.00	0.00	N
ATOM	1356	H	PHE	A	106	49.142	26.914	30.013	1.00	0.00	H
ATOM	1357	CA	PHE	A	106	49.963	27.546	31.882	1.00	0.00	C
ATOM	1358	HA	PHE	A	106	50.179	28.501	32.087	1.00	0.00	H
ATOM	1359	CB	PHE	A	106	48.593	27.219	32.524	1.00	0.00	C
ATOM	1360	HB1	PHE	A	106	48.430	26.233	32.489	1.00	0.00	H
ATOM	1361	HB2	PHE	A	106	47.866	27.695	32.029	1.00	0.00	H
ATOM	1362	CG	PHE	A	106	48.515	27.633	33.935	1.00	0.00	C
ATOM	1363	CD1	PHE	A	106	47.938	28.926	34.239	1.00	0.00	C
ATOM	1364	HD1	PHE	A	106	47.663	29.555	33.512	1.00	0.00	H
ATOM	1365	CE1	PHE	A	106	47.781	29.259	35.579	1.00	0.00	C
ATOM	1366	HE1	PHE	A	106	47.267	30.080	35.826	1.00	0.00	H
ATOM	1367	CZ	PHE	A	106	48.331	28.463	36.586	1.00	0.00	C
ATOM	1368	HZ	PHE	A	106	48.312	28.794	37.529	1.00	0.00	H
ATOM	1369	CE2	PHE	A	106	48.903	27.228	36.320	1.00	0.00	C
ATOM	1370	HE2	PHE	A	106	49.178	26.614	37.060	1.00	0.00	H
ATOM	1371	CD2	PHE	A	106	49.089	26.857	34.919	1.00	0.00	C
ATOM	1372	HD2	PHE	A	106	49.627	26.049	34.677	1.00	0.00	H
ATOM	1373	C	PHE	A	106	51.087	26.722	32.433	1.00	0.00	C
ATOM	1374	O	PHE	A	106	51.741	27.200	33.422	1.00	0.00	O
ATOM	1375	N	THR	A	107	51.490	25.655	31.709	1.00	0.00	N
ATOM	1376	H	THR	A	107	50.944	25.321	30.941	1.00	0.00	H
ATOM	1377	CA	THR	A	107	52.781	24.983	32.088	1.00	0.00	C
ATOM	1378	HA	THR	A	107	52.855	25.078	33.081	1.00	0.00	H
ATOM	1379	CB	THR	A	107	52.816	23.445	31.763	1.00	0.00	C
ATOM	1380	HB	THR	A	107	52.965	23.365	30.777	1.00	0.00	H
ATOM	1381	CG2	THR	A	107	53.830	22.640	32.512	1.00	0.00	C
ATOM	1382	HG21	THR	A	107	53.768	21.682	32.232	1.00	0.00	H
ATOM	1383	HG22	THR	A	107	54.746	22.987	32.311	1.00	0.00	H
ATOM	1384	HG23	THR	A	107	53.654	22.713	33.494	1.00	0.00	H
ATOM	1385	OG1	THR	A	107	51.609	22.834	32.194	1.00	0.00	O
ATOM	1386	HG1	THR	A	107	51.637	21.857	31.985	1.00	0.00	H
ATOM	1387	C	THR	A	107	53.946	25.732	31.564	1.00	0.00	C
ATOM	1388	O	THR	A	107	54.931	25.649	32.267	1.00	0.00	O
ATOM	1389	N	ASP	A	108	53.906	26.416	30.406	1.00	0.00	N
ATOM	1390	H	ASP	A	108	53.086	26.411	29.833	1.00	0.00	H
ATOM	1391	CA	ASP	A	108	55.107	27.173	30.006	1.00	0.00	C
ATOM	1392	HA	ASP	A	108	55.771	26.425	30.005	1.00	0.00	H
ATOM	1393	CB	ASP	A	108	55.007	27.847	28.646	1.00	0.00	C
ATOM	1394	HB1	ASP	A	108	55.804	28.443	28.546	1.00	0.00	H
ATOM	1395	HB2	ASP	A	108	54.174	28.401	28.644	1.00	0.00	H
ATOM	1396	CG	ASP	A	108	54.959	26.863	27.433	1.00	0.00	C
ATOM	1397	OD1	ASP	A	108	54.506	27.336	26.397	1.00	0.00	O
ATOM	1398	OD2	ASP	A	108	55.416	25.728	27.487	1.00	0.00	O
ATOM	1399	C	ASP	A	108	55.468	28.315	30.968	1.00	0.00	C
ATOM	1400	O	ASP	A	108	56.639	28.650	31.139	1.00	0.00	O
ATOM	1401	N	MET	A	109	54.383	28.844	31.623	1.00	0.00	N

```
ATOM   1402  H   MET A 109      53.487  28.412  31.515  1.00  0.00           H
ATOM   1403  CA  MET A 109      54.508  30.007  32.462  1.00  0.00           C
ATOM   1404  HA  MET A 109      55.303  30.502  32.111  1.00  0.00           H
ATOM   1405  CB  MET A 109      53.205  30.851  32.304  1.00  0.00           C
ATOM   1406  HB1 MET A 109      52.423  30.236  32.403  1.00  0.00           H
ATOM   1407  HB2 MET A 109      53.203  31.249  31.387  1.00  0.00           H
ATOM   1408  CG  MET A 109      53.019  32.005  33.312  1.00  0.00           C
ATOM   1409  HG1 MET A 109      52.352  32.596  32.858  1.00  0.00           H
ATOM   1410  HG2 MET A 109      53.914  32.451  33.312  1.00  0.00           H
ATOM   1411  SD  MET A 109      52.478  31.546  35.026  1.00  0.00           S
ATOM   1412  CE  MET A 109      50.981  30.580  34.790  1.00  0.00           C
ATOM   1413  HE1 MET A 109      50.626  30.293  35.680  1.00  0.00           H
ATOM   1414  HE2 MET A 109      50.295  31.137  34.321  1.00  0.00           H
ATOM   1415  HE3 MET A 109      51.189  29.773  34.238  1.00  0.00           H
ATOM   1416  C   MET A 109      54.939  29.705  33.899  1.00  0.00           C
ATOM   1417  O   MET A 109      55.692  30.413  34.544  1.00  0.00           O
ATOM   1418  N   VAL A 110      54.309  28.676  34.424  1.00  0.00           N
ATOM   1419  H   VAL A 110      53.865  28.015  33.819  1.00  0.00           H
ATOM   1420  CA  VAL A 110      54.242  28.476  35.860  1.00  0.00           C
ATOM   1421  HA  VAL A 110      54.300  29.385  36.273  1.00  0.00           H
ATOM   1422  CB  VAL A 110      52.853  27.838  36.203  1.00  0.00           C
ATOM   1423  HB  VAL A 110      52.244  28.069  35.445  1.00  0.00           H
ATOM   1424  CG1 VAL A 110      52.770  26.281  36.249  1.00  0.00           C
ATOM   1425 HG11 VAL A 110      51.837  26.004  36.477  1.00  0.00           H
ATOM   1426 HG12 VAL A 110      53.020  25.907  35.356  1.00  0.00           H
ATOM   1427 HG13 VAL A 110      53.400  25.934  36.944  1.00  0.00           H
ATOM   1428  CG2 VAL A 110      52.442  28.417  37.614  1.00  0.00           C
ATOM   1429 HG21 VAL A 110      51.556  28.040  37.884  1.00  0.00           H
ATOM   1430 HG22 VAL A 110      53.132  28.164  38.292  1.00  0.00           H
ATOM   1431 HG23 VAL A 110      52.378  29.413  37.560  1.00  0.00           H
ATOM   1432  C   VAL A 110      55.489  27.747  36.423  1.00  0.00           C
ATOM   1433  O   VAL A 110      55.846  27.947  37.611  1.00  0.00           O
ATOM   1434  N   ALA A 111      56.228  26.970  35.652  1.00  0.00           N
ATOM   1435  H   ALA A 111      55.926  26.870  34.704  1.00  0.00           H
ATOM   1436  CA  ALA A 111      57.446  26.235  36.031  1.00  0.00           C
ATOM   1437  HA  ALA A 111      57.311  26.078  37.009  1.00  0.00           H
ATOM   1438  CB  ALA A 111      57.452  24.994  35.149  1.00  0.00           C
ATOM   1439  HB1 ALA A 111      58.260  24.443  35.355  1.00  0.00           H
ATOM   1440  HB2 ALA A 111      56.628  24.456  35.325  1.00  0.00           H
ATOM   1441  HB3 ALA A 111      57.472  25.268  34.188  1.00  0.00           H
ATOM   1442  C   ALA A 111      58.829  26.940  36.056  1.00  0.00           C
ATOM   1443  O   ALA A 111      59.805  26.260  36.342  1.00  0.00           O
ATOM   1444  N   THR A 112      58.891  28.232  35.682  1.00  0.00           N
ATOM   1445  H   THR A 112      58.034  28.710  35.488  1.00  0.00           H
ATOM   1446  CA  THR A 112      60.139  28.972  35.544  1.00  0.00           C
ATOM   1447  HA  THR A 112      60.819  28.440  36.049  1.00  0.00           H
ATOM   1448  CB  THR A 112      60.565  29.135  34.071  1.00  0.00           C
ATOM   1449  HB  THR A 112      59.831  29.533  33.521  1.00  0.00           H
ATOM   1450  CG2 THR A 112      61.814  29.951  33.873  1.00  0.00           C
ATOM   1451 HG21 THR A 112      62.022  30.010  32.897  1.00  0.00           H
ATOM   1452 HG22 THR A 112      61.673  30.871  34.240  1.00  0.00           H
ATOM   1453 HG23 THR A 112      62.576  29.515  34.351  1.00  0.00           H
ATOM   1454  OG1 THR A 112      60.912  27.848  33.672  1.00  0.00           O
ATOM   1455  HG1 THR A 112      61.204  27.863  32.716  1.00  0.00           H
```

ATOM	1456	C	THR	A	112	59.901	30.378	36.091	1.00	0.00	C
ATOM	1457	O	THR	A	112	59.131	31.143	35.556	1.00	0.00	O
ATOM	1458	N	ALA	A	113	60.668	30.729	37.125	1.00	0.00	N
ATOM	1459	H	ALA	A	113	61.386	30.087	37.394	1.00	0.00	H
ATOM	1460	CA	ALA	A	113	60.557	31.988	37.918	1.00	0.00	C
ATOM	1461	HA	ALA	A	113	59.757	31.918	38.514	1.00	0.00	H
ATOM	1462	CB	ALA	A	113	61.844	32.165	38.710	1.00	0.00	C
ATOM	1463	HB1	ALA	A	113	61.790	33.005	39.250	1.00	0.00	H
ATOM	1464	HB2	ALA	A	113	61.969	31.384	39.321	1.00	0.00	H
ATOM	1465	HB3	ALA	A	113	62.618	32.223	38.080	1.00	0.00	H
ATOM	1466	C	ALA	A	113	60.362	33.175	37.023	1.00	0.00	C
ATOM	1467	O	ALA	A	113	59.386	33.872	37.224	1.00	0.00	O
ATOM	1468	N	ASN	A	114	61.242	33.512	36.044	1.00	0.00	N
ATOM	1469	H	ASN	A	114	62.050	32.938	35.910	1.00	0.00	H
ATOM	1470	CA	ASN	A	114	61.064	34.667	35.183	1.00	0.00	C
ATOM	1471	HA	ASN	A	114	61.063	35.406	35.856	1.00	0.00	H
ATOM	1472	CB	ASN	A	114	62.231	34.824	34.180	1.00	0.00	C
ATOM	1473	HB1	ASN	A	114	62.184	34.093	33.499	1.00	0.00	H
ATOM	1474	HB2	ASN	A	114	63.100	34.766	34.671	1.00	0.00	H
ATOM	1475	CG	ASN	A	114	62.147	36.180	33.467	1.00	0.00	C
ATOM	1476	OD1	ASN	A	114	62.289	37.300	34.191	1.00	0.00	O
ATOM	1477	ND2	ASN	A	114	62.088	36.316	32.232	1.00	0.00	N
ATOM	1478	HD21	ASN	A	114	62.094	35.510	31.639	1.00	0.00	H
ATOM	1479	HD22	ASN	A	114	62.035	37.231	31.832	1.00	0.00	H
ATOM	1480	C	ASN	A	114	59.720	34.575	34.332	1.00	0.00	C
ATOM	1481	O	ASN	A	114	59.100	35.604	34.072	1.00	0.00	O
ATOM	1482	N	ASN	A	115	59.241	33.446	33.858	1.00	0.00	N
ATOM	1483	H	ASN	A	115	59.760	32.613	34.049	1.00	0.00	H
ATOM	1484	CA	ASN	A	115	58.014	33.304	33.075	1.00	0.00	C
ATOM	1485	HA	ASN	A	115	58.034	33.985	32.343	1.00	0.00	H
ATOM	1486	CB	ASN	A	115	57.908	31.930	32.420	1.00	0.00	C
ATOM	1487	HB1	ASN	A	115	57.121	31.965	31.804	1.00	0.00	H
ATOM	1488	HB2	ASN	A	115	57.729	31.275	33.154	1.00	0.00	H
ATOM	1489	CG	ASN	A	115	59.136	31.413	31.602	1.00	0.00	C
ATOM	1490	OD1	ASN	A	115	60.287	31.874	31.675	1.00	0.00	O
ATOM	1491	ND2	ASN	A	115	58.886	30.347	30.905	1.00	0.00	N
ATOM	1492	HD21	ASN	A	115	57.974	29.937	30.927	1.00	0.00	H
ATOM	1493	HD22	ASN	A	115	59.605	29.935	30.346	1.00	0.00	H
ATOM	1494	C	ASN	A	115	56.862	33.502	34.057	1.00	0.00	C
ATOM	1495	O	ASN	A	115	55.933	34.194	33.780	1.00	0.00	O
ATOM	1496	N	ARG	A	116	56.920	32.893	35.257	1.00	0.00	N
ATOM	1497	H	ARG	A	116	57.727	32.349	35.487	1.00	0.00	H
ATOM	1498	CA	ARG	A	116	55.851	33.004	36.220	1.00	0.00	C
ATOM	1499	HA	ARG	A	116	55.022	32.684	35.762	1.00	0.00	H
ATOM	1500	CB	ARG	A	116	56.187	32.159	37.398	1.00	0.00	C
ATOM	1501	HB1	ARG	A	116	56.867	32.649	37.943	1.00	0.00	H
ATOM	1502	HB2	ARG	A	116	56.583	31.305	37.060	1.00	0.00	H
ATOM	1503	CG	ARG	A	116	55.036	31.816	38.290	1.00	0.00	C
ATOM	1504	HG1	ARG	A	116	54.367	31.300	37.755	1.00	0.00	H
ATOM	1505	HG2	ARG	A	116	54.622	32.668	38.610	1.00	0.00	H
ATOM	1506	CD	ARG	A	116	55.430	30.994	39.488	1.00	0.00	C
ATOM	1507	HD1	ARG	A	116	56.013	30.237	39.195	1.00	0.00	H
ATOM	1508	HD2	ARG	A	116	54.609	30.631	39.928	1.00	0.00	H
ATOM	1509	NE	ARG	A	116	56.159	31.749	40.479	1.00	0.00	N

ATOM	1510	HE	ARG	A	116	55.708	32.583	40.796	1.00	0.00	H
ATOM	1511	CZ	ARG	A	116	57.305	31.507	41.016	1.00	0.00	C
ATOM	1512	NH1	ARG	A	116	57.867	30.378	40.765	1.00	0.00	N
ATOM	1513	HH11	ARG	A	116	57.414	29.716	40.167	1.00	0.00	H
ATOM	1514	HH12	ARG	A	116	58.757	30.162	41.167	1.00	0.00	H
ATOM	1515	NH2	ARG	A	116	57.910	32.523	41.652	1.00	0.00	N
ATOM	1516	HH21	ARG	A	116	57.470	33.420	41.694	1.00	0.00	H
ATOM	1517	HH22	ARG	A	116	58.801	32.383	42.084	1.00	0.00	H
ATOM	1518	C	ARG	A	116	55.754	34.465	36.728	1.00	0.00	C
ATOM	1519	O	ARG	A	116	54.618	34.906	36.873	1.00	0.00	O
ATOM	1520	N	GLN	A	117	56.853	35.218	36.805	1.00	0.00	N
ATOM	1521	H	GLN	A	117	57.738	34.862	36.506	1.00	0.00	H
ATOM	1522	CA	GLN	A	117	56.735	36.566	37.332	1.00	0.00	C
ATOM	1523	HA	GLN	A	117	55.864	36.594	37.823	1.00	0.00	H
ATOM	1524	CB	GLN	A	117	57.983	36.770	38.199	1.00	0.00	C
ATOM	1525	HB1	GLN	A	117	58.132	37.754	38.291	1.00	0.00	H
ATOM	1526	HB2	GLN	A	117	58.758	36.359	37.719	1.00	0.00	H
ATOM	1527	CG	GLN	A	117	57.854	36.120	39.644	1.00	0.00	C
ATOM	1528	HG1	GLN	A	117	58.776	35.955	39.995	1.00	0.00	H
ATOM	1529	HG2	GLN	A	117	57.371	35.249	39.552	1.00	0.00	H
ATOM	1530	CD	GLN	A	117	57.093	36.961	40.715	1.00	0.00	C
ATOM	1531	OE1	GLN	A	117	57.189	38.204	40.770	1.00	0.00	O
ATOM	1532	NE2	GLN	A	117	56.223	36.372	41.532	1.00	0.00	N
ATOM	1533	HE21	GLN	A	117	56.056	35.389	41.460	1.00	0.00	H
ATOM	1534	HE22	GLN	A	117	55.736	36.913	42.218	1.00	0.00	H
ATOM	1535	C	GLN	A	117	56.595	37.671	36.301	1.00	0.00	C
ATOM	1536	O	GLN	A	117	56.093	38.719	36.596	1.00	0.00	O
ATOM	1537	N	THR	A	118	56.797	37.359	35.012	1.00	0.00	N
ATOM	1538	H	THR	A	118	57.345	36.548	34.808	1.00	0.00	H
ATOM	1539	CA	THR	A	118	56.265	38.132	33.880	1.00	0.00	C
ATOM	1540	HA	THR	A	118	56.629	39.060	33.956	1.00	0.00	H
ATOM	1541	CB	THR	A	118	56.824	37.548	32.607	1.00	0.00	C
ATOM	1542	HB	THR	A	118	56.677	36.560	32.648	1.00	0.00	H
ATOM	1543	CG2	THR	A	118	56.156	38.036	31.324	1.00	0.00	C
ATOM	1544	HG21	THR	A	118	56.589	37.599	30.536	1.00	0.00	H
ATOM	1545	HG22	THR	A	118	55.184	37.801	31.344	1.00	0.00	H
ATOM	1546	HG23	THR	A	118	56.257	39.028	31.252	1.00	0.00	H
ATOM	1547	OG1	THR	A	118	58.246	37.791	32.562	1.00	0.00	O
ATOM	1548	HG1	THR	A	118	58.623	37.406	31.720	1.00	0.00	H
ATOM	1549	C	THR	A	118	54.667	38.194	33.915	1.00	0.00	C
ATOM	1550	O	THR	A	118	54.079	39.281	33.804	1.00	0.00	O
ATOM	1551	N	PHE	A	119	54.038	37.076	34.250	1.00	0.00	N
ATOM	1552	H	PHE	A	119	54.594	36.263	34.421	1.00	0.00	H
ATOM	1553	CA	PHE	A	119	52.582	36.948	34.388	1.00	0.00	C
ATOM	1554	HA	PHE	A	119	52.263	37.248	33.489	1.00	0.00	H
ATOM	1555	CB	PHE	A	119	52.192	35.480	34.734	1.00	0.00	C
ATOM	1556	HB1	PHE	A	119	52.223	35.380	35.728	1.00	0.00	H
ATOM	1557	HB2	PHE	A	119	52.870	34.875	34.317	1.00	0.00	H
ATOM	1558	CG	PHE	A	119	50.836	34.973	34.293	1.00	0.00	C
ATOM	1559	CD1	PHE	A	119	50.555	34.876	32.948	1.00	0.00	C
ATOM	1560	HD1	PHE	A	119	51.255	35.122	32.278	1.00	0.00	H
ATOM	1561	CE1	PHE	A	119	49.316	34.442	32.515	1.00	0.00	C
ATOM	1562	HE1	PHE	A	119	49.155	34.358	31.532	1.00	0.00	H
ATOM	1563	CZ	PHE	A	119	48.240	34.103	33.427	1.00	0.00	C

ATOM	1564	HZ	PHE	A	119	47.364	33.744	33.107	1.00	0.00	H
ATOM	1565	CE2	PHE	A	119	48.509	34.312	34.787	1.00	0.00	C
ATOM	1566	HE2	PHE	A	119	47.793	34.143	35.464	1.00	0.00	H
ATOM	1567	CD2	PHE	A	119	49.783	34.760	35.212	1.00	0.00	C
ATOM	1568	HD2	PHE	A	119	49.942	34.930	36.185	1.00	0.00	H
ATOM	1569	C	PHE	A	119	51.970	37.861	35.462	1.00	0.00	C
ATOM	1570	O	PHE	A	119	50.990	38.541	35.153	1.00	0.00	O
ATOM	1571	N	VAL	A	120	52.356	37.783	36.759	1.00	0.00	N
ATOM	1572	H	VAL	A	120	53.091	37.143	36.983	1.00	0.00	H
ATOM	1573	CA	VAL	A	120	51.783	38.560	37.857	1.00	0.00	C
ATOM	1574	HA	VAL	A	120	50.787	38.487	37.806	1.00	0.00	H
ATOM	1575	CB	VAL	A	120	52.246	37.913	39.188	1.00	0.00	C
ATOM	1576	HB	VAL	A	120	51.717	38.381	39.896	1.00	0.00	H
ATOM	1577	CG1	VAL	A	120	52.090	36.394	39.025	1.00	0.00	C
ATOM	1578	HG11	VAL	A	120	52.380	35.937	39.866	1.00	0.00	H
ATOM	1579	HG12	VAL	A	120	51.132	36.175	38.840	1.00	0.00	H
ATOM	1580	HG13	VAL	A	120	52.657	36.081	38.263	1.00	0.00	H
ATOM	1581	CG2	VAL	A	120	53.630	38.109	39.658	1.00	0.00	C
ATOM	1582	HG21	VAL	A	120	53.759	37.628	40.525	1.00	0.00	H
ATOM	1583	HG22	VAL	A	120	54.267	37.747	38.978	1.00	0.00	H
ATOM	1584	HG23	VAL	A	120	53.802	39.085	39.790	1.00	0.00	H
ATOM	1585	C	VAL	A	120	52.024	40.078	37.690	1.00	0.00	C
ATOM	1586	O	VAL	A	120	51.121	40.853	37.999	1.00	0.00	O
ATOM	1587	N	ASN	A	121	53.194	40.452	37.161	1.00	0.00	N
ATOM	1588	H	ASN	A	121	53.891	39.749	37.020	1.00	0.00	H
ATOM	1589	CA	ASN	A	121	53.506	41.786	36.788	1.00	0.00	C
ATOM	1590	HA	ASN	A	121	53.243	42.288	37.612	1.00	0.00	H
ATOM	1591	CB	ASN	A	121	54.985	41.906	36.492	1.00	0.00	C
ATOM	1592	HB1	ASN	A	121	55.177	42.767	36.021	1.00	0.00	H
ATOM	1593	HB2	ASN	A	121	55.290	41.141	35.925	1.00	0.00	H
ATOM	1594	CG	ASN	A	121	55.679	41.875	37.833	1.00	0.00	C
ATOM	1595	OD1	ASN	A	121	55.435	42.706	38.708	1.00	0.00	O
ATOM	1596	ND2	ASN	A	121	56.536	40.951	38.094	1.00	0.00	N
ATOM	1597	HD21	ASN	A	121	56.743	40.253	37.409	1.00	0.00	H
ATOM	1598	HD22	ASN	A	121	56.995	40.931	38.982	1.00	0.00	H
ATOM	1599	C	ASN	A	121	52.703	42.304	35.570	1.00	0.00	C
ATOM	1600	O	ASN	A	121	52.325	43.484	35.586	1.00	0.00	O
ATOM	1601	N	SER	A	122	52.307	41.364	34.714	1.00	0.00	N
ATOM	1602	H	SER	A	122	52.657	40.436	34.842	1.00	0.00	H
ATOM	1603	CA	SER	A	122	51.401	41.603	33.609	1.00	0.00	C
ATOM	1604	HA	SER	A	122	51.727	42.444	33.177	1.00	0.00	H
ATOM	1605	CB	SER	A	122	51.489	40.405	32.650	1.00	0.00	C
ATOM	1606	HB1	SER	A	122	51.427	39.537	33.143	1.00	0.00	H
ATOM	1607	HB2	SER	A	122	52.334	40.426	32.116	1.00	0.00	H
ATOM	1608	OG	SER	A	122	50.379	40.502	31.754	1.00	0.00	O
ATOM	1609	HG	SER	A	122	50.402	39.736	31.111	1.00	0.00	H
ATOM	1610	C	SER	A	122	49.915	41.772	34.115	1.00	0.00	C
ATOM	1611	O	SER	A	122	49.255	42.795	33.826	1.00	0.00	O
ATOM	1612	N	ALA	A	123	49.458	40.882	34.987	1.00	0.00	N
ATOM	1613	H	ALA	A	123	50.056	40.129	35.263	1.00	0.00	H
ATOM	1614	CA	ALA	A	123	48.084	40.961	35.573	1.00	0.00	C
ATOM	1615	HA	ALA	A	123	47.442	40.883	34.810	1.00	0.00	H
ATOM	1616	CB	ALA	A	123	48.023	39.845	36.625	1.00	0.00	C
ATOM	1617	HB1	ALA	A	123	47.122	39.844	37.058	1.00	0.00	H

ATOM	1618	HB2	ALA	A	123	48.182	38.962	36.184	1.00	0.00	H
ATOM	1619	HB3	ALA	A	123	48.726	40.002	37.319	1.00	0.00	H
ATOM	1620	C	ALA	A	123	47.700	42.269	36.210	1.00	0.00	C
ATOM	1621	O	ALA	A	123	46.694	42.901	35.879	1.00	0.00	O
ATOM	1622	N	ILE	A	124	48.544	42.817	37.051	1.00	0.00	N
ATOM	1623	H	ILE	A	124	49.417	42.366	37.236	1.00	0.00	H
ATOM	1624	CA	ILE	A	124	48.230	44.075	37.721	1.00	0.00	C
ATOM	1625	HA	ILE	A	124	47.345	43.861	38.135	1.00	0.00	H
ATOM	1626	CB	ILE	A	124	49.138	44.450	38.856	1.00	0.00	C
ATOM	1627	HB	ILE	A	124	48.927	45.351	39.235	1.00	0.00	H
ATOM	1628	CG2	ILE	A	124	48.878	43.506	40.002	1.00	0.00	C
ATOM	1629	HG21	ILE	A	124	49.475	43.741	40.769	1.00	0.00	H
ATOM	1630	HG22	ILE	A	124	47.923	43.583	40.287	1.00	0.00	H
ATOM	1631	HG23	ILE	A	124	49.064	42.568	39.710	1.00	0.00	H
ATOM	1632	CG1	ILE	A	124	50.576	44.615	38.359	1.00	0.00	C
ATOM	1633	HG11	ILE	A	124	50.556	45.185	37.538	1.00	0.00	H
ATOM	1634	HG12	ILE	A	124	50.929	43.709	38.127	1.00	0.00	H
ATOM	1635	CD	ILE	A	124	51.537	45.262	39.373	1.00	0.00	C
ATOM	1636	HD1	ILE	A	124	52.449	45.332	38.970	1.00	0.00	H
ATOM	1637	HD2	ILE	A	124	51.206	46.176	39.609	1.00	0.00	H
ATOM	1638	HD3	ILE	A	124	51.579	44.699	40.198	1.00	0.00	H
ATOM	1639	C	ILE	A	124	48.195	45.245	36.700	1.00	0.00	C
ATOM	1640	O	ILE	A	124	47.594	46.259	36.958	1.00	0.00	O
ATOM	1641	N	ARG	A	125	48.874	45.143	35.573	1.00	0.00	N
ATOM	1642	H	ARG	A	125	49.449	44.336	35.437	1.00	0.00	H
ATOM	1643	CA	ARG	A	125	48.828	46.160	34.501	1.00	0.00	C
ATOM	1644	HA	ARG	A	125	48.813	47.074	34.907	1.00	0.00	H
ATOM	1645	CB	ARG	A	125	50.115	46.055	33.657	1.00	0.00	C
ATOM	1646	HB1	ARG	A	125	49.971	46.517	32.782	1.00	0.00	H
ATOM	1647	HB2	ARG	A	125	50.322	45.090	33.495	1.00	0.00	H
ATOM	1648	CG	ARG	A	125	51.309	46.688	34.335	1.00	0.00	C
ATOM	1649	HG1	ARG	A	125	51.642	46.076	35.052	1.00	0.00	H
ATOM	1650	HG2	ARG	A	125	51.032	47.559	34.742	1.00	0.00	H
ATOM	1651	CD	ARG	A	125	52.416	46.915	33.246	1.00	0.00	C
ATOM	1652	HD1	ARG	A	125	53.123	47.520	33.613	1.00	0.00	H
ATOM	1653	HD2	ARG	A	125	52.005	47.335	32.437	1.00	0.00	H
ATOM	1654	NE	ARG	A	125	53.087	45.631	32.809	1.00	0.00	N
ATOM	1655	HE	ARG	A	125	52.695	45.112	32.049	1.00	0.00	H
ATOM	1656	CZ	ARG	A	125	54.165	45.180	33.396	1.00	0.00	C
ATOM	1657	NH1	ARG	A	125	54.726	45.706	34.445	1.00	0.00	N
ATOM	1658	HH11	ARG	A	125	54.331	46.522	34.867	1.00	0.00	H
ATOM	1659	HH12	ARG	A	125	55.552	45.294	34.830	1.00	0.00	H
ATOM	1660	NH2	ARG	A	125	54.718	44.135	32.865	1.00	0.00	N
ATOM	1661	HH21	ARG	A	125	54.318	43.718	32.049	1.00	0.00	H
ATOM	1662	HH22	ARG	A	125	55.545	43.746	33.271	1.00	0.00	H
ATOM	1663	C	ARG	A	125	47.516	46.014	33.709	1.00	0.00	C
ATOM	1664	O	ARG	A	125	46.861	46.984	33.385	1.00	0.00	O
ATOM	1665	N	PHE	A	126	47.222	44.781	33.258	1.00	0.00	N
ATOM	1666	H	PHE	A	126	47.831	44.045	33.554	1.00	0.00	H
ATOM	1667	CA	PHE	A	126	46.096	44.386	32.371	1.00	0.00	C
ATOM	1668	HA	PHE	A	126	46.233	44.828	31.485	1.00	0.00	H
ATOM	1669	CB	PHE	A	126	46.040	42.870	32.094	1.00	0.00	C
ATOM	1670	HB1	PHE	A	126	45.571	42.453	32.872	1.00	0.00	H
ATOM	1671	HB2	PHE	A	126	46.986	42.548	32.064	1.00	0.00	H

ATOM	1672	CG	PHE	A	126	45.308	42.402	30.757	1.00	0.00	C
ATOM	1673	CD1	PHE	A	126	43.938	41.955	30.776	1.00	0.00	C
ATOM	1674	HD1	PHE	A	126	43.414	41.993	31.627	1.00	0.00	H
ATOM	1675	CE1	PHE	A	126	43.340	41.471	29.616	1.00	0.00	C
ATOM	1676	HE1	PHE	A	126	42.367	41.242	29.606	1.00	0.00	H
ATOM	1677	CZ	PHE	A	126	44.138	41.304	28.433	1.00	0.00	C
ATOM	1678	HZ	PHE	A	126	43.712	40.954	27.599	1.00	0.00	H
ATOM	1679	CE2	PHE	A	126	45.530	41.629	28.427	1.00	0.00	C
ATOM	1680	HE2	PHE	A	126	46.099	41.471	27.620	1.00	0.00	H
ATOM	1681	CD2	PHE	A	126	46.076	42.191	29.628	1.00	0.00	C
ATOM	1682	HD2	PHE	A	126	47.045	42.438	29.649	1.00	0.00	H
ATOM	1683	C	PHE	A	126	44.831	44.878	33.022	1.00	0.00	C
ATOM	1684	O	PHE	A	126	44.159	45.699	32.472	1.00	0.00	O
ATOM	1685	N	LEU	A	127	44.500	44.415	34.269	1.00	0.00	N
ATOM	1686	H	LEU	A	127	45.165	43.840	34.745	1.00	0.00	H
ATOM	1687	CA	LEU	A	127	43.244	44.705	34.946	1.00	0.00	C
ATOM	1688	HA	LEU	A	127	42.555	44.534	34.241	1.00	0.00	H
ATOM	1689	CB	LEU	A	127	43.133	43.770	36.139	1.00	0.00	C
ATOM	1690	HB1	LEU	A	127	42.212	43.902	36.506	1.00	0.00	H
ATOM	1691	HB2	LEU	A	127	43.806	44.093	36.804	1.00	0.00	H
ATOM	1692	CG	LEU	A	127	43.347	42.237	35.977	1.00	0.00	C
ATOM	1693	HG	LEU	A	127	44.213	42.040	35.516	1.00	0.00	H
ATOM	1694	CD1	LEU	A	127	43.265	41.653	37.397	1.00	0.00	C
ATOM	1695	HD11	LEU	A	127	43.396	40.662	37.358	1.00	0.00	H
ATOM	1696	HD12	LEU	A	127	43.978	42.061	37.967	1.00	0.00	H
ATOM	1697	HD13	LEU	A	127	42.368	41.855	37.789	1.00	0.00	H
ATOM	1698	CD2	LEU	A	127	42.273	41.599	35.124	1.00	0.00	C
ATOM	1699	HD21	LEU	A	127	42.448	40.618	35.046	1.00	0.00	H
ATOM	1700	HD22	LEU	A	127	41.379	41.745	35.548	1.00	0.00	H
ATOM	1701	HD23	LEU	A	127	42.281	42.012	34.213	1.00	0.00	H
ATOM	1702	C	LEU	A	127	42.955	46.142	35.306	1.00	0.00	C
ATOM	1703	O	LEU	A	127	41.900	46.701	34.912	1.00	0.00	O
ATOM	1704	N	ARG	A	128	43.975	46.934	35.596	1.00	0.00	N
ATOM	1705	H	ARG	A	128	44.865	46.484	35.674	1.00	0.00	H
ATOM	1706	CA	ARG	A	128	43.974	48.342	35.812	1.00	0.00	C
ATOM	1707	HA	ARG	A	128	43.164	48.364	36.398	1.00	0.00	H
ATOM	1708	CB	ARG	A	128	45.198	48.883	36.659	1.00	0.00	C
ATOM	1709	HB1	ARG	A	128	45.179	49.883	36.678	1.00	0.00	H
ATOM	1710	HB2	ARG	A	128	46.056	48.573	36.248	1.00	0.00	H
ATOM	1711	CG	ARG	A	128	45.154	48.381	38.098	1.00	0.00	C
ATOM	1712	HG1	ARG	A	128	46.031	48.580	38.535	1.00	0.00	H
ATOM	1713	HG2	ARG	A	128	45.003	47.393	38.089	1.00	0.00	H
ATOM	1714	CD	ARG	A	128	44.060	49.021	38.918	1.00	0.00	C
ATOM	1715	HD1	ARG	A	128	43.160	48.735	38.590	1.00	0.00	H
ATOM	1716	HD2	ARG	A	128	44.127	50.018	38.880	1.00	0.00	H
ATOM	1717	NE	ARG	A	128	44.251	48.564	40.302	1.00	0.00	N
ATOM	1718	HE	ARG	A	128	45.190	48.397	40.603	1.00	0.00	H
ATOM	1719	CZ	ARG	A	128	43.287	48.355	41.182	1.00	0.00	C
ATOM	1720	NH1	ARG	A	128	42.040	48.527	40.902	1.00	0.00	N
ATOM	1721	HH11	ARG	A	128	41.770	48.830	39.988	1.00	0.00	H
ATOM	1722	HH12	ARG	A	128	41.343	48.357	41.599	1.00	0.00	H
ATOM	1723	NH2	ARG	A	128	43.611	48.038	42.425	1.00	0.00	N
ATOM	1724	HH21	ARG	A	128	44.573	47.960	42.686	1.00	0.00	H
ATOM	1725	HH22	ARG	A	128	42.894	47.877	43.103	1.00	0.00	H

ATOM	1726	C	ARG	A	128	43.741	49.182	34.464	1.00	0.00	C
ATOM	1727	O	ARG	A	128	43.057	50.183	34.466	1.00	0.00	O
ATOM	1728	N	LYS	A	129	44.449	48.803	33.382	1.00	0.00	N
ATOM	1729	H	LYS	A	129	45.104	48.051	33.449	1.00	0.00	H
ATOM	1730	CA	LYS	A	129	44.252	49.509	32.086	1.00	0.00	C
ATOM	1731	HA	LYS	A	129	44.419	50.485	32.223	1.00	0.00	H
ATOM	1732	CB	LYS	A	129	45.266	48.888	31.129	1.00	0.00	C
ATOM	1733	HB1	LYS	A	129	45.053	47.914	31.051	1.00	0.00	H
ATOM	1734	HB2	LYS	A	129	46.174	49.000	31.534	1.00	0.00	H
ATOM	1735	CG	LYS	A	129	45.287	49.499	29.705	1.00	0.00	C
ATOM	1736	HG1	LYS	A	129	45.449	50.483	29.783	1.00	0.00	H
ATOM	1737	HG2	LYS	A	129	44.397	49.341	29.278	1.00	0.00	H
ATOM	1738	CD	LYS	A	129	46.340	48.940	28.775	1.00	0.00	C
ATOM	1739	HD1	LYS	A	129	46.118	47.976	28.627	1.00	0.00	H
ATOM	1740	HD2	LYS	A	129	47.217	49.011	29.250	1.00	0.00	H
ATOM	1741	CE	LYS	A	129	46.438	49.683	27.368	1.00	0.00	C
ATOM	1742	HE1	LYS	A	129	47.298	49.439	26.920	1.00	0.00	H
ATOM	1743	HE2	LYS	A	129	46.405	50.672	27.510	1.00	0.00	H
ATOM	1744	NZ	LYS	A	129	45.360	49.367	26.396	1.00	0.00	N
ATOM	1745	HZ1	LYS	A	129	45.512	49.880	25.551	1.00	0.00	H
ATOM	1746	HZ2	LYS	A	129	45.368	48.387	26.197	1.00	0.00	H
ATOM	1747	HZ3	LYS	A	129	44.475	49.621	26.786	1.00	0.00	H
ATOM	1748	C	LYS	A	129	42.806	49.464	31.554	1.00	0.00	C
ATOM	1749	O	LYS	A	129	42.350	50.468	30.956	1.00	0.00	O
ATOM	1750	N	TYR	A	130	42.055	48.363	31.849	1.00	0.00	N
ATOM	1751	H	TYR	A	130	42.485	47.648	32.401	1.00	0.00	H
ATOM	1752	CA	TYR	A	130	40.677	48.143	31.426	1.00	0.00	C
ATOM	1753	HA	TYR	A	130	40.643	48.762	30.642	1.00	0.00	H
ATOM	1754	CB	TYR	A	130	40.404	46.712	30.903	1.00	0.00	C
ATOM	1755	HB1	TYR	A	130	39.433	46.646	30.673	1.00	0.00	H
ATOM	1756	HB2	TYR	A	130	40.617	46.068	31.638	1.00	0.00	H
ATOM	1757	CG	TYR	A	130	41.249	46.328	29.636	1.00	0.00	C
ATOM	1758	CD1	TYR	A	130	41.100	47.020	28.424	1.00	0.00	C
ATOM	1759	HD1	TYR	A	130	40.438	47.764	28.334	1.00	0.00	H
ATOM	1760	CE1	TYR	A	130	41.913	46.634	27.334	1.00	0.00	C
ATOM	1761	HE1	TYR	A	130	41.771	47.094	26.457	1.00	0.00	H
ATOM	1762	CZ	TYR	A	130	42.900	45.652	27.406	1.00	0.00	C
ATOM	1763	OH	TYR	A	130	43.568	45.209	26.296	1.00	0.00	O
ATOM	1764	HH	TYR	A	130	44.230	44.509	26.563	1.00	0.00	H
ATOM	1765	CE2	TYR	A	130	43.072	45.010	28.648	1.00	0.00	C
ATOM	1766	HE2	TYR	A	130	43.771	44.304	28.758	1.00	0.00	H
ATOM	1767	CD2	TYR	A	130	42.250	45.372	29.733	1.00	0.00	C
ATOM	1768	HD2	TYR	A	130	42.394	44.920	30.613	1.00	0.00	H
ATOM	1769	C	TYR	A	130	39.611	48.637	32.434	1.00	0.00	C
ATOM	1770	O	TYR	A	130	38.451	48.914	32.114	1.00	0.00	O
ATOM	1771	N	SER	A	131	40.028	48.740	33.663	1.00	0.00	N
ATOM	1772	H	SER	A	131	41.001	48.573	33.821	1.00	0.00	H
ATOM	1773	CA	SER	A	131	39.195	49.086	34.876	1.00	0.00	C
ATOM	1774	HA	SER	A	131	39.867	49.370	35.560	1.00	0.00	H
ATOM	1775	CB	SER	A	131	38.351	50.318	34.666	1.00	0.00	C
ATOM	1776	HB1	SER	A	131	38.001	50.664	35.536	1.00	0.00	H
ATOM	1777	HB2	SER	A	131	37.586	50.127	34.051	1.00	0.00	H
ATOM	1778	OG	SER	A	131	39.163	51.320	34.077	1.00	0.00	O
ATOM	1779	HG	SER	A	131	38.619	52.146	33.930	1.00	0.00	H

ATOM	1780	C	SER	A	131	38.450	47.880	35.530	1.00	0.00	C
ATOM	1781	O	SER	A	131	37.352	47.987	36.122	1.00	0.00	O
ATOM	1782	N	PHE	A	132	39.064	46.653	35.490	1.00	0.00	N
ATOM	1783	H	PHE	A	132	39.870	46.564	34.904	1.00	0.00	H
ATOM	1784	CA	PHE	A	132	38.644	45.470	36.226	1.00	0.00	C
ATOM	1785	HA	PHE	A	132	37.666	45.611	36.071	1.00	0.00	H
ATOM	1786	CB	PHE	A	132	39.188	44.177	35.624	1.00	0.00	C
ATOM	1787	HB1	PHE	A	132	38.941	43.431	36.243	1.00	0.00	H
ATOM	1788	HB2	PHE	A	132	40.184	44.261	35.584	1.00	0.00	H
ATOM	1789	CG	PHE	A	132	38.679	43.815	34.207	1.00	0.00	C
ATOM	1790	CD1	PHE	A	132	37.376	43.283	34.032	1.00	0.00	C
ATOM	1791	HD1	PHE	A	132	36.768	43.195	34.821	1.00	0.00	H
ATOM	1792	CE1	PHE	A	132	36.925	42.873	32.744	1.00	0.00	C
ATOM	1793	HE1	PHE	A	132	36.005	42.504	32.615	1.00	0.00	H
ATOM	1794	CZ	PHE	A	132	37.786	42.999	31.690	1.00	0.00	C
ATOM	1795	HZ	PHE	A	132	37.446	42.858	30.760	1.00	0.00	H
ATOM	1796	CE2	PHE	A	132	39.152	43.326	31.887	1.00	0.00	C
ATOM	1797	HE2	PHE	A	132	39.798	43.259	31.127	1.00	0.00	H
ATOM	1798	CD2	PHE	A	132	39.587	43.737	33.132	1.00	0.00	C
ATOM	1799	HD2	PHE	A	132	40.547	43.979	33.271	1.00	0.00	H
ATOM	1800	C	PHE	A	132	38.897	45.404	37.757	1.00	0.00	C
ATOM	1801	O	PHE	A	132	39.958	45.871	38.152	1.00	0.00	O
ATOM	1802	N	ASP	A	133	37.939	44.892	38.491	1.00	0.00	N
ATOM	1803	H	ASP	A	133	37.094	44.680	37.999	1.00	0.00	H
ATOM	1804	CA	ASP	A	133	37.919	44.582	39.929	1.00	0.00	C
ATOM	1805	HA	ASP	A	133	38.608	45.222	40.268	1.00	0.00	H
ATOM	1806	CB	ASP	A	133	36.505	44.865	40.521	1.00	0.00	C
ATOM	1807	HB1	ASP	A	133	36.461	44.549	41.469	1.00	0.00	H
ATOM	1808	HB2	ASP	A	133	35.807	44.396	39.980	1.00	0.00	H
ATOM	1809	CG	ASP	A	133	36.206	46.326	40.502	1.00	0.00	C
ATOM	1810	OD1	ASP	A	133	37.097	47.167	40.727	1.00	0.00	O
ATOM	1811	OD2	ASP	A	133	34.993	46.599	40.389	1.00	0.00	O
ATOM	1812	C	ASP	A	133	38.446	43.184	40.349	1.00	0.00	C
ATOM	1813	O	ASP	A	133	38.745	42.999	41.549	1.00	0.00	O
ATOM	1814	N	GLY	A	134	38.774	42.203	39.465	1.00	0.00	N
ATOM	1815	H	GLY	A	134	38.562	42.323	38.495	1.00	0.00	H
ATOM	1816	CA	GLY	A	134	39.459	40.925	39.929	1.00	0.00	C
ATOM	1817	HA1	GLY	A	134	38.820	40.397	40.488	1.00	0.00	H
ATOM	1818	HA2	GLY	A	134	40.261	41.165	40.476	1.00	0.00	H
ATOM	1819	C	GLY	A	134	39.911	40.060	38.804	1.00	0.00	C
ATOM	1820	O	GLY	A	134	39.733	40.399	37.614	1.00	0.00	O
ATOM	1821	N	LEU	A	135	40.480	38.896	39.085	1.00	0.00	N
ATOM	1822	H	LEU	A	135	40.671	38.751	40.056	1.00	0.00	H
ATOM	1823	CA	LEU	A	135	40.867	37.813	38.235	1.00	0.00	C
ATOM	1824	HA	LEU	A	135	40.568	38.038	37.308	1.00	0.00	H
ATOM	1825	CB	LEU	A	135	42.421	37.652	38.444	1.00	0.00	C
ATOM	1826	HB1	LEU	A	135	42.566	37.224	39.336	1.00	0.00	H
ATOM	1827	HB2	LEU	A	135	42.825	38.567	38.443	1.00	0.00	H
ATOM	1828	CG	LEU	A	135	43.239	36.839	37.460	1.00	0.00	C
ATOM	1829	HG	LEU	A	135	42.783	35.962	37.310	1.00	0.00	H
ATOM	1830	CD1	LEU	A	135	43.323	37.472	36.026	1.00	0.00	C
ATOM	1831	HD11	LEU	A	135	43.875	36.885	35.434	1.00	0.00	H
ATOM	1832	HD12	LEU	A	135	42.402	37.558	35.645	1.00	0.00	H
ATOM	1833	HD13	LEU	A	135	43.745	38.377	36.085	1.00	0.00	H

```
ATOM   1834  CD2 LEU A 135      44.611  36.620  38.120  1.00  0.00           C
ATOM   1835 HD21 LEU A 135      45.194  36.085  37.508  1.00  0.00           H
ATOM   1836 HD22 LEU A 135      45.040  37.505  38.299  1.00  0.00           H
ATOM   1837 HD23 LEU A 135      44.491  36.127  38.982  1.00  0.00           H
ATOM   1838  C   LEU A 135      40.179  36.530  38.664  1.00  0.00           C
ATOM   1839  O   LEU A 135      40.213  36.239  39.831  1.00  0.00           O
ATOM   1840  N   ASP A 136      39.632  35.720  37.768  1.00  0.00           N
ATOM   1841  H   ASP A 136      39.653  36.012  36.812  1.00  0.00           H
ATOM   1842  CA  ASP A 136      39.000  34.432  38.064  1.00  0.00           C
ATOM   1843  HA  ASP A 136      39.132  34.254  39.039  1.00  0.00           H
ATOM   1844  CB  ASP A 136      37.491  34.510  37.810  1.00  0.00           C
ATOM   1845  HB1 ASP A 136      37.337  34.673  36.835  1.00  0.00           H
ATOM   1846  HB2 ASP A 136      37.115  35.271  38.339  1.00  0.00           H
ATOM   1847  CG  ASP A 136      36.762  33.226  38.213  1.00  0.00           C
ATOM   1848  OD1 ASP A 136      35.881  32.785  37.448  1.00  0.00           O
ATOM   1849  OD2 ASP A 136      37.027  32.728  39.332  1.00  0.00           O
ATOM   1850  C   ASP A 136      39.698  33.308  37.287  1.00  0.00           C
ATOM   1851  O   ASP A 136      39.903  33.411  36.076  1.00  0.00           O
ATOM   1852  N   LEU A 137      40.164  32.297  38.018  1.00  0.00           N
ATOM   1853  H   LEU A 137      39.824  32.213  38.955  1.00  0.00           H
ATOM   1854  CA  LEU A 137      41.134  31.297  37.563  1.00  0.00           C
ATOM   1855  HA  LEU A 137      41.410  31.587  36.647  1.00  0.00           H
ATOM   1856  CB  LEU A 137      42.292  31.275  38.576  1.00  0.00           C
ATOM   1857  HB1 LEU A 137      42.830  30.449  38.407  1.00  0.00           H
ATOM   1858  HB2 LEU A 137      41.895  31.233  39.493  1.00  0.00           H
ATOM   1859  CG  LEU A 137      43.254  32.454  38.554  1.00  0.00           C
ATOM   1860  HG  LEU A 137      42.739  33.262  38.841  1.00  0.00           H
ATOM   1861  CD1 LEU A 137      44.395  32.058  39.474  1.00  0.00           C
ATOM   1862 HD11 LEU A 137      45.069  32.796  39.505  1.00  0.00           H
ATOM   1863 HD12 LEU A 137      44.039  31.892  40.394  1.00  0.00           H
ATOM   1864 HD13 LEU A 137      44.828  31.225  39.129  1.00  0.00           H
ATOM   1865  CD2 LEU A 137      43.876  32.773  37.165  1.00  0.00           C
ATOM   1866 HD21 LEU A 137      44.491  33.557  37.247  1.00  0.00           H
ATOM   1867 HD22 LEU A 137      44.390  31.979  36.840  1.00  0.00           H
ATOM   1868 HD23 LEU A 137      43.147  32.987  36.515  1.00  0.00           H
ATOM   1869  C   LEU A 137      40.539  29.891  37.446  1.00  0.00           C
ATOM   1870  O   LEU A 137      39.811  29.463  38.335  1.00  0.00           O
ATOM   1871  N   ASP A 138      40.992  29.131  36.447  1.00  0.00           N
ATOM   1872  H   ASP A 138      41.552  29.576  35.748  1.00  0.00           H
ATOM   1873  CA  ASP A 138      40.734  27.695  36.292  1.00  0.00           C
ATOM   1874  HA  ASP A 138      40.664  27.300  37.208  1.00  0.00           H
ATOM   1875  CB  ASP A 138      39.407  27.506  35.547  1.00  0.00           C
ATOM   1876  HB1 ASP A 138      39.557  27.717  34.581  1.00  0.00           H
ATOM   1877  HB2 ASP A 138      38.740  28.145  35.928  1.00  0.00           H
ATOM   1878  CG  ASP A 138      38.824  26.094  35.642  1.00  0.00           C
ATOM   1879  OD1 ASP A 138      37.772  25.907  34.989  1.00  0.00           O
ATOM   1880  OD2 ASP A 138      39.378  25.258  36.398  1.00  0.00           O
ATOM   1881  C   ASP A 138      41.905  27.024  35.545  1.00  0.00           C
ATOM   1882  O   ASP A 138      42.635  27.691  34.804  1.00  0.00           O
ATOM   1883  N   TRP A 139      42.143  25.732  35.778  1.00  0.00           N
ATOM   1884  H   TRP A 139      41.531  25.239  36.396  1.00  0.00           H
ATOM   1885  CA  TRP A 139      43.262  25.000  35.169  1.00  0.00           C
ATOM   1886  HA  TRP A 139      43.279  25.311  34.219  1.00  0.00           H
ATOM   1887  CB  TRP A 139      44.580  25.330  35.915  1.00  0.00           C
```

ATOM	1888	HB1	TRP	A	139	44.474	25.059	36.872	1.00	0.00	H
ATOM	1889	HB2	TRP	A	139	44.730	26.317	35.863	1.00	0.00	H
ATOM	1890	CG	TRP	A	139	45.838	24.686	35.431	1.00	0.00	C
ATOM	1891	CD1	TRP	A	139	45.989	24.435	34.138	1.00	0.00	C
ATOM	1892	HD1	TRP	A	139	45.326	24.667	33.426	1.00	0.00	H
ATOM	1893	NE1	TRP	A	139	47.207	23.802	33.919	1.00	0.00	N
ATOM	1894	HE1	TRP	A	139	47.560	23.539	33.021	1.00	0.00	H
ATOM	1895	CE2	TRP	A	139	47.870	23.584	35.134	1.00	0.00	C
ATOM	1896	CZ2	TRP	A	139	49.215	23.169	35.444	1.00	0.00	C
ATOM	1897	HZ2	TRP	A	139	49.825	22.805	34.740	1.00	0.00	H
ATOM	1898	CH2	TRP	A	139	49.641	23.303	36.818	1.00	0.00	C
ATOM	1899	HH2	TRP	A	139	50.557	23.007	37.089	1.00	0.00	H
ATOM	1900	CZ3	TRP	A	139	48.752	23.860	37.791	1.00	0.00	C
ATOM	1901	HZ3	TRP	A	139	49.064	24.005	38.730	1.00	0.00	H
ATOM	1902	CE3	TRP	A	139	47.483	24.192	37.437	1.00	0.00	C
ATOM	1903	HE3	TRP	A	139	46.858	24.478	38.164	1.00	0.00	H
ATOM	1904	CD2	TRP	A	139	46.994	24.163	36.145	1.00	0.00	C
ATOM	1905	C	TRP	A	139	43.038	23.489	35.184	1.00	0.00	C
ATOM	1906	O	TRP	A	139	42.843	22.920	36.261	1.00	0.00	O
ATOM	1907	N	GLU	A	140	43.240	22.812	34.043	1.00	0.00	N
ATOM	1908	H	GLU	A	140	43.421	23.304	33.191	1.00	0.00	H
ATOM	1909	CA	GLU	A	140	43.193	21.340	34.054	1.00	0.00	C
ATOM	1910	HA	GLU	A	140	42.794	21.113	34.942	1.00	0.00	H
ATOM	1911	CB	GLU	A	140	42.364	20.869	32.838	1.00	0.00	C
ATOM	1912	HB1	GLU	A	140	42.451	19.875	32.770	1.00	0.00	H
ATOM	1913	HB2	GLU	A	140	42.748	21.294	32.018	1.00	0.00	H
ATOM	1914	CG	GLU	A	140	40.866	21.206	32.882	1.00	0.00	C
ATOM	1915	HG1	GLU	A	140	40.550	21.109	33.826	1.00	0.00	H
ATOM	1916	HG2	GLU	A	140	40.381	20.554	32.299	1.00	0.00	H
ATOM	1917	CD	GLU	A	140	40.525	22.625	32.404	1.00	0.00	C
ATOM	1918	OE1	GLU	A	140	41.275	23.172	31.562	1.00	0.00	O
ATOM	1919	OE2	GLU	A	140	39.486	23.139	32.860	1.00	0.00	O
ATOM	1920	C	GLU	A	140	44.565	20.675	33.967	1.00	0.00	C
ATOM	1921	O	GLU	A	140	45.155	20.605	32.863	1.00	0.00	O
ATOM	1922	N	TYR	A	141	45.179	20.191	35.058	1.00	0.00	N
ATOM	1923	H	TYR	A	141	46.113	19.864	34.916	1.00	0.00	H
ATOM	1924	CA	TYR	A	141	44.640	20.068	36.513	1.00	0.00	C
ATOM	1925	HA	TYR	A	141	43.969	20.787	36.696	1.00	0.00	H
ATOM	1926	CB	TYR	A	141	43.939	18.689	36.745	1.00	0.00	C
ATOM	1927	HB1	TYR	A	141	43.670	18.612	37.705	1.00	0.00	H
ATOM	1928	HB2	TYR	A	141	44.575	17.952	36.517	1.00	0.00	H
ATOM	1929	CG	TYR	A	141	42.720	18.456	35.946	1.00	0.00	C
ATOM	1930	CD1	TYR	A	141	41.475	18.875	36.467	1.00	0.00	C
ATOM	1931	HD1	TYR	A	141	41.436	19.318	37.363	1.00	0.00	H
ATOM	1932	CE1	TYR	A	141	40.289	18.674	35.735	1.00	0.00	C
ATOM	1933	HE1	TYR	A	141	39.412	18.979	36.106	1.00	0.00	H
ATOM	1934	CZ	TYR	A	141	40.344	18.040	34.476	1.00	0.00	C
ATOM	1935	OH	TYR	A	141	39.217	17.843	33.747	1.00	0.00	O
ATOM	1936	HH	TYR	A	141	39.450	17.383	32.890	1.00	0.00	H
ATOM	1937	CE2	TYR	A	141	41.586	17.621	33.964	1.00	0.00	C
ATOM	1938	HE2	TYR	A	141	41.628	17.147	33.085	1.00	0.00	H
ATOM	1939	CD2	TYR	A	141	42.769	17.867	34.685	1.00	0.00	C
ATOM	1940	HD2	TYR	A	141	43.652	17.617	34.289	1.00	0.00	H
ATOM	1941	C	TYR	A	141	45.815	20.238	37.459	1.00	0.00	C

ATOM	1942	O	TYR	A	141	46.825	19.512	37.362	1.00	0.00	O
ATOM	1943	N	PRO	A	142	45.596	20.871	38.640	1.00	0.00	N
ATOM	1944	CD	PRO	A	142	44.573	21.817	38.893	1.00	0.00	C
ATOM	1945	HD1	PRO	A	142	43.693	21.355	38.779	1.00	0.00	H
ATOM	1946	HD2	PRO	A	142	44.656	22.547	38.215	1.00	0.00	H
ATOM	1947	CG	PRO	A	142	44.716	22.370	40.305	1.00	0.00	C
ATOM	1948	HG1	PRO	A	142	44.243	21.798	40.976	1.00	0.00	H
ATOM	1949	HG2	PRO	A	142	44.379	23.309	40.370	1.00	0.00	H
ATOM	1950	CB	PRO	A	142	46.217	22.307	40.483	1.00	0.00	C
ATOM	1951	HB1	PRO	A	142	46.461	22.252	41.451	1.00	0.00	H
ATOM	1952	HB2	PRO	A	142	46.660	23.103	40.072	1.00	0.00	H
ATOM	1953	CA	PRO	A	142	46.548	20.982	39.713	1.00	0.00	C
ATOM	1954	HA	PRO	A	142	47.447	20.970	39.276	1.00	0.00	H
ATOM	1955	C	PRO	A	142	46.559	19.785	40.634	1.00	0.00	C
ATOM	1956	O	PRO	A	142	45.909	19.654	41.687	1.00	0.00	O
ATOM	1957	N	GLY	A	143	47.351	18.741	40.165	1.00	0.00	N
ATOM	1958	H	GLY	A	143	47.662	18.779	39.215	1.00	0.00	H
ATOM	1959	CA	GLY	A	143	47.747	17.620	40.955	1.00	0.00	C
ATOM	1960	HA1	GLY	A	143	48.063	17.975	41.835	1.00	0.00	H
ATOM	1961	HA2	GLY	A	143	48.508	17.178	40.481	1.00	0.00	H
ATOM	1962	C	GLY	A	143	46.553	16.628	41.146	1.00	0.00	C
ATOM	1963	O	GLY	A	143	46.809	15.562	41.771	1.00	0.00	O
ATOM	1964	N	SER	A	144	45.394	16.787	40.491	1.00	0.00	N
ATOM	1965	H	SER	A	144	45.309	17.559	39.861	1.00	0.00	H
ATOM	1966	CA	SER	A	144	44.255	15.898	40.647	1.00	0.00	C
ATOM	1967	HA	SER	A	144	44.439	15.430	41.511	1.00	0.00	H
ATOM	1968	CB	SER	A	144	42.981	16.679	40.749	1.00	0.00	C
ATOM	1969	HB1	SER	A	144	43.014	17.320	41.515	1.00	0.00	H
ATOM	1970	HB2	SER	A	144	42.195	16.071	40.857	1.00	0.00	H
ATOM	1971	OG	SER	A	144	42.774	17.413	39.614	1.00	0.00	O
ATOM	1972	HG	SER	A	144	41.920	17.927	39.700	1.00	0.00	H
ATOM	1973	C	SER	A	144	44.120	14.913	39.436	1.00	0.00	C
ATOM	1974	O	SER	A	144	44.765	15.145	38.368	1.00	0.00	O
ATOM	1975	N	GLN	A	145	43.266	13.839	39.494	1.00	0.00	N
ATOM	1976	H	GLN	A	145	42.786	13.688	40.358	1.00	0.00	H
ATOM	1977	CA	GLN	A	145	42.992	12.892	38.407	1.00	0.00	C
ATOM	1978	HA	GLN	A	145	42.621	12.141	38.954	1.00	0.00	H
ATOM	1979	CB	GLN	A	145	41.887	13.404	37.447	1.00	0.00	C
ATOM	1980	HB1	GLN	A	145	41.085	13.611	38.008	1.00	0.00	H
ATOM	1981	HB2	GLN	A	145	41.672	12.654	36.822	1.00	0.00	H
ATOM	1982	CG	GLN	A	145	42.148	14.646	36.574	1.00	0.00	C
ATOM	1983	HG1	GLN	A	145	42.435	15.394	37.172	1.00	0.00	H
ATOM	1984	HG2	GLN	A	145	41.293	14.893	36.119	1.00	0.00	H
ATOM	1985	CD	GLN	A	145	43.199	14.504	35.502	1.00	0.00	C
ATOM	1986	OE1	GLN	A	145	42.906	14.095	34.392	1.00	0.00	O
ATOM	1987	NE2	GLN	A	145	44.468	14.822	35.720	1.00	0.00	N
ATOM	1988	HE21	GLN	A	145	44.750	15.167	36.615	1.00	0.00	H
ATOM	1989	HE22	GLN	A	145	45.142	14.717	34.989	1.00	0.00	H
ATOM	1990	C	GLN	A	145	44.194	12.184	37.733	1.00	0.00	C
ATOM	1991	O	GLN	A	145	44.034	11.804	36.586	1.00	0.00	O
ATOM	1992	N	GLY	A	146	45.356	12.204	38.397	1.00	0.00	N
ATOM	1993	H	GLY	A	146	45.374	12.616	39.308	1.00	0.00	H
ATOM	1994	CA	GLY	A	146	46.635	11.640	37.845	1.00	0.00	C
ATOM	1995	HA1	GLY	A	146	46.443	11.260	36.940	1.00	0.00	H

ATOM	1996	HA2	GLY	A	146	46.942	10.909	38.455	1.00	0.00	H
ATOM	1997	C	GLY	A	146	47.748	12.687	37.721	1.00	0.00	C
ATOM	1998	O	GLY	A	146	48.942	12.306	37.920	1.00	0.00	O
ATOM	1999	N	SER	A	147	47.445	13.940	37.357	1.00	0.00	N
ATOM	2000	H	SER	A	147	46.507	14.139	37.074	1.00	0.00	H
ATOM	2001	CA	SER	A	147	48.420	15.033	37.353	1.00	0.00	C
ATOM	2002	HA	SER	A	147	48.962	14.907	36.522	1.00	0.00	H
ATOM	2003	CB	SER	A	147	47.663	16.345	37.324	1.00	0.00	C
ATOM	2004	HB1	SER	A	147	47.260	16.531	38.220	1.00	0.00	H
ATOM	2005	HB2	SER	A	147	46.940	16.311	36.634	1.00	0.00	H
ATOM	2006	OG	SER	A	147	48.423	17.451	37.017	1.00	0.00	O
ATOM	2007	HG	SER	A	147	47.843	18.266	37.021	1.00	0.00	H
ATOM	2008	C	SER	A	147	49.407	14.987	38.519	1.00	0.00	C
ATOM	2009	O	SER	A	147	48.920	14.681	39.600	1.00	0.00	O
ATOM	2010	N	PRO	A	148	50.723	15.135	38.447	1.00	0.00	N
ATOM	2011	CD	PRO	A	148	51.419	15.363	37.203	1.00	0.00	C
ATOM	2012	HD1	PRO	A	148	50.886	15.972	36.616	1.00	0.00	H
ATOM	2013	HD2	PRO	A	148	51.574	14.496	36.730	1.00	0.00	H
ATOM	2014	CG	PRO	A	148	52.710	15.997	37.627	1.00	0.00	C
ATOM	2015	HG1	PRO	A	148	52.586	16.977	37.784	1.00	0.00	H
ATOM	2016	HG2	PRO	A	148	53.415	15.858	36.932	1.00	0.00	H
ATOM	2017	CB	PRO	A	148	53.027	15.222	38.953	1.00	0.00	C
ATOM	2018	HB1	PRO	A	148	53.642	15.741	39.547	1.00	0.00	H
ATOM	2019	HB2	PRO	A	148	53.421	14.321	38.770	1.00	0.00	H
ATOM	2020	CA	PRO	A	148	51.642	15.092	39.585	1.00	0.00	C
ATOM	2021	HA	PRO	A	148	51.460	14.244	40.083	1.00	0.00	H
ATOM	2022	C	PRO	A	148	51.455	16.216	40.593	1.00	0.00	C
ATOM	2023	O	PRO	A	148	51.144	17.362	40.281	1.00	0.00	O
ATOM	2024	N	ALA	A	149	52.013	15.996	41.757	1.00	0.00	N
ATOM	2025	H	ALA	A	149	52.475	15.118	41.882	1.00	0.00	H
ATOM	2026	CA	ALA	A	149	52.021	16.895	42.861	1.00	0.00	C
ATOM	2027	HA	ALA	A	149	51.065	17.178	42.934	1.00	0.00	H
ATOM	2028	CB	ALA	A	149	52.375	16.174	44.142	1.00	0.00	C
ATOM	2029	HB1	ALA	A	149	52.374	16.824	44.902	1.00	0.00	H
ATOM	2030	HB2	ALA	A	149	51.702	15.456	44.317	1.00	0.00	H
ATOM	2031	HB3	ALA	A	149	53.283	15.765	44.054	1.00	0.00	H
ATOM	2032	C	ALA	A	149	52.832	18.173	42.596	1.00	0.00	C
ATOM	2033	O	ALA	A	149	52.646	19.167	43.331	1.00	0.00	O
ATOM	2034	N	VAL	A	150	53.695	18.204	41.569	1.00	0.00	N
ATOM	2035	H	VAL	A	150	53.922	17.332	41.135	1.00	0.00	H
ATOM	2036	CA	VAL	A	150	54.316	19.376	41.045	1.00	0.00	C
ATOM	2037	HA	VAL	A	150	54.683	19.791	41.878	1.00	0.00	H
ATOM	2038	CB	VAL	A	150	55.514	19.000	40.068	1.00	0.00	C
ATOM	2039	HB	VAL	A	150	56.012	18.187	40.368	1.00	0.00	H
ATOM	2040	CG1	VAL	A	150	55.050	18.593	38.712	1.00	0.00	C
ATOM	2041	HG11	VAL	A	150	55.841	18.369	38.142	1.00	0.00	H
ATOM	2042	HG12	VAL	A	150	54.457	17.792	38.789	1.00	0.00	H
ATOM	2043	HG13	VAL	A	150	54.541	19.345	38.294	1.00	0.00	H
ATOM	2044	CG2	VAL	A	150	56.448	20.220	40.008	1.00	0.00	C
ATOM	2045	HG21	VAL	A	150	57.217	20.021	39.401	1.00	0.00	H
ATOM	2046	HG22	VAL	A	150	55.943	21.010	39.659	1.00	0.00	H
ATOM	2047	HG23	VAL	A	150	56.791	20.422	40.925	1.00	0.00	H
ATOM	2048	C	VAL	A	150	53.297	20.424	40.494	1.00	0.00	C
ATOM	2049	O	VAL	A	150	53.475	21.606	40.672	1.00	0.00	O

ATOM	2050	N	ASP	A	151	52.253	19.976	39.833	1.00	0.00	N
ATOM	2051	H	ASP	A	151	52.205	19.002	39.612	1.00	0.00	H
ATOM	2052	CA	ASP	A	151	51.172	20.839	39.413	1.00	0.00	C
ATOM	2053	HA	ASP	A	151	51.575	21.608	38.917	1.00	0.00	H
ATOM	2054	CB	ASP	A	151	50.358	20.126	38.417	1.00	0.00	C
ATOM	2055	HB1	ASP	A	151	49.466	20.576	38.371	1.00	0.00	H
ATOM	2056	HB2	ASP	A	151	50.241	19.185	38.733	1.00	0.00	H
ATOM	2057	CG	ASP	A	151	50.964	20.105	37.041	1.00	0.00	C
ATOM	2058	OD1	ASP	A	151	50.294	19.542	36.127	1.00	0.00	O
ATOM	2059	OD2	ASP	A	151	52.118	20.639	36.824	1.00	0.00	O
ATOM	2060	C	ASP	A	151	50.352	21.358	40.670	1.00	0.00	C
ATOM	2061	O	ASP	A	151	49.682	22.450	40.616	1.00	0.00	O
ATOM	2062	N	LYS	A	152	50.259	20.603	41.769	1.00	0.00	N
ATOM	2063	H	LYS	A	152	50.646	19.681	41.746	1.00	0.00	H
ATOM	2064	CA	LYS	A	152	49.628	21.053	42.987	1.00	0.00	C
ATOM	2065	HA	LYS	A	152	48.716	21.355	42.708	1.00	0.00	H
ATOM	2066	CB	LYS	A	152	49.273	19.954	43.963	1.00	0.00	C
ATOM	2067	HB1	LYS	A	152	50.115	19.508	44.266	1.00	0.00	H
ATOM	2068	HB2	LYS	A	152	48.691	19.286	43.498	1.00	0.00	H
ATOM	2069	CG	LYS	A	152	48.540	20.470	45.173	1.00	0.00	C
ATOM	2070	HG1	LYS	A	152	47.682	20.892	44.879	1.00	0.00	H
ATOM	2071	HG2	LYS	A	152	49.108	21.153	45.632	1.00	0.00	H
ATOM	2072	CD	LYS	A	152	48.223	19.398	46.146	1.00	0.00	C
ATOM	2073	HD1	LYS	A	152	47.720	18.692	45.648	1.00	0.00	H
ATOM	2074	HD2	LYS	A	152	47.629	19.802	46.841	1.00	0.00	H
ATOM	2075	CE	LYS	A	152	49.477	18.759	46.837	1.00	0.00	C
ATOM	2076	HE1	LYS	A	152	50.184	19.442	47.022	1.00	0.00	H
ATOM	2077	HE2	LYS	A	152	49.864	18.019	46.287	1.00	0.00	H
ATOM	2078	NZ	LYS	A	152	49.043	18.208	48.091	1.00	0.00	N
ATOM	2079	HZ1	LYS	A	152	49.822	17.791	48.559	1.00	0.00	H
ATOM	2080	HZ2	LYS	A	152	48.662	18.936	48.661	1.00	0.00	H
ATOM	2081	HZ3	LYS	A	152	48.343	17.514	47.925	1.00	0.00	H
ATOM	2082	C	LYS	A	152	50.419	22.248	43.555	1.00	0.00	C
ATOM	2083	O	LYS	A	152	49.885	23.291	43.824	1.00	0.00	O
ATOM	2084	N	GLU	A	153	51.749	22.131	43.617	1.00	0.00	N
ATOM	2085	H	GLU	A	153	52.155	21.268	43.317	1.00	0.00	H
ATOM	2086	CA	GLU	A	153	52.696	23.229	44.117	1.00	0.00	C
ATOM	2087	HA	GLU	A	153	52.246	23.571	44.942	1.00	0.00	H
ATOM	2088	CB	GLU	A	153	54.110	22.664	44.460	1.00	0.00	C
ATOM	2089	HB1	GLU	A	153	54.671	22.630	43.633	1.00	0.00	H
ATOM	2090	HB2	GLU	A	153	54.023	21.744	44.842	1.00	0.00	H
ATOM	2091	CG	GLU	A	153	54.809	23.538	45.475	1.00	0.00	C
ATOM	2092	HG1	GLU	A	153	54.709	24.484	45.167	1.00	0.00	H
ATOM	2093	HG2	GLU	A	153	55.776	23.284	45.465	1.00	0.00	H
ATOM	2094	CD	GLU	A	153	54.298	23.446	46.934	1.00	0.00	C
ATOM	2095	OE1	GLU	A	153	54.600	22.505	47.624	1.00	0.00	O
ATOM	2096	OE2	GLU	A	153	53.633	24.345	47.502	1.00	0.00	O
ATOM	2097	C	GLU	A	153	52.797	24.461	43.233	1.00	0.00	C
ATOM	2098	O	GLU	A	153	52.686	25.563	43.736	1.00	0.00	O
ATOM	2099	N	ARG	A	154	52.679	24.254	41.894	1.00	0.00	N
ATOM	2100	H	ARG	A	154	52.596	23.303	41.596	1.00	0.00	H
ATOM	2101	CA	ARG	A	154	52.660	25.275	40.839	1.00	0.00	C
ATOM	2102	HA	ARG	A	154	53.453	25.871	40.970	1.00	0.00	H
ATOM	2103	CB	ARG	A	154	52.713	24.558	39.511	1.00	0.00	C

ATOM	2104	HB1	ARG	A	154	52.310	25.149	38.812	1.00	0.00	H
ATOM	2105	HB2	ARG	A	154	52.181	23.714	39.582	1.00	0.00	H
ATOM	2106	CG	ARG	A	154	54.170	24.221	39.126	1.00	0.00	C
ATOM	2107	HG1	ARG	A	154	54.632	23.748	39.876	1.00	0.00	H
ATOM	2108	HG2	ARG	A	154	54.675	25.053	38.895	1.00	0.00	H
ATOM	2109	CD	ARG	A	154	54.081	23.310	37.910	1.00	0.00	C
ATOM	2110	HD1	ARG	A	154	53.728	23.834	37.135	1.00	0.00	H
ATOM	2111	HD2	ARG	A	154	53.457	22.555	38.113	1.00	0.00	H
ATOM	2112	NE	ARG	A	154	55.341	22.717	37.484	1.00	0.00	N
ATOM	2113	HE	ARG	A	154	56.162	23.119	37.890	1.00	0.00	H
ATOM	2114	CZ	ARG	A	154	55.559	21.712	36.628	1.00	0.00	C
ATOM	2115	NH1	ARG	A	154	54.608	21.166	35.925	1.00	0.00	N
ATOM	2116	HH11	ARG	A	154	53.668	21.496	36.013	1.00	0.00	H
ATOM	2117	HH12	ARG	A	154	54.818	20.416	35.297	1.00	0.00	H
ATOM	2118	NH2	ARG	A	154	56.812	21.456	36.375	1.00	0.00	N
ATOM	2119	HH21	ARG	A	154	57.531	21.997	36.811	1.00	0.00	H
ATOM	2120	HH22	ARG	A	154	57.054	20.719	35.745	1.00	0.00	H
ATOM	2121	C	ARG	A	154	51.469	26.172	40.927	1.00	0.00	C
ATOM	2122	O	ARG	A	154	51.507	27.382	40.781	1.00	0.00	O
ATOM	2123	N	PHE	A	155	50.275	25.630	41.246	1.00	0.00	N
ATOM	2124	H	PHE	A	155	50.201	24.633	41.276	1.00	0.00	H
ATOM	2125	CA	PHE	A	155	49.046	26.447	41.560	1.00	0.00	C
ATOM	2126	HA	PHE	A	155	48.993	27.195	40.898	1.00	0.00	H
ATOM	2127	CB	PHE	A	155	47.821	25.552	41.397	1.00	0.00	C
ATOM	2128	HB1	PHE	A	155	47.748	24.971	42.208	1.00	0.00	H
ATOM	2129	HB2	PHE	A	155	47.957	24.980	40.588	1.00	0.00	H
ATOM	2130	CG	PHE	A	155	46.510	26.224	41.231	1.00	0.00	C
ATOM	2131	CD1	PHE	A	155	46.298	27.036	40.036	1.00	0.00	C
ATOM	2132	HD1	PHE	A	155	47.024	27.175	39.363	1.00	0.00	H
ATOM	2133	CE1	PHE	A	155	45.000	27.626	39.871	1.00	0.00	C
ATOM	2134	HE1	PHE	A	155	44.772	28.182	39.072	1.00	0.00	H
ATOM	2135	CZ	PHE	A	155	44.074	27.383	40.885	1.00	0.00	C
ATOM	2136	HZ	PHE	A	155	43.198	27.862	40.822	1.00	0.00	H
ATOM	2137	CE2	PHE	A	155	44.257	26.516	42.005	1.00	0.00	C
ATOM	2138	HE2	PHE	A	155	43.502	26.287	42.620	1.00	0.00	H
ATOM	2139	CD2	PHE	A	155	45.545	26.002	42.200	1.00	0.00	C
ATOM	2140	HD2	PHE	A	155	45.765	25.483	43.026	1.00	0.00	H
ATOM	2141	C	PHE	A	155	49.113	27.157	42.924	1.00	0.00	C
ATOM	2142	O	PHE	A	155	48.764	28.341	43.102	1.00	0.00	O
ATOM	2143	N	THR	A	156	49.639	26.431	43.919	1.00	0.00	N
ATOM	2144	H	THR	A	156	49.922	25.487	43.747	1.00	0.00	H
ATOM	2145	CA	THR	A	156	49.807	27.017	45.284	1.00	0.00	C
ATOM	2146	HA	THR	A	156	48.879	27.212	45.603	1.00	0.00	H
ATOM	2147	CB	THR	A	156	50.387	26.009	46.281	1.00	0.00	C
ATOM	2148	HB	THR	A	156	51.308	25.713	46.028	1.00	0.00	H
ATOM	2149	CG2	THR	A	156	50.435	26.577	47.676	1.00	0.00	C
ATOM	2150	HG21	THR	A	156	50.817	25.896	48.300	1.00	0.00	H
ATOM	2151	HG22	THR	A	156	51.010	27.395	47.683	1.00	0.00	H
ATOM	2152	HG23	THR	A	156	49.510	26.817	47.970	1.00	0.00	H
ATOM	2153	OG1	THR	A	156	49.585	24.862	46.249	1.00	0.00	O
ATOM	2154	HG1	THR	A	156	49.944	24.186	46.893	1.00	0.00	H
ATOM	2155	C	THR	A	156	50.690	28.339	45.207	1.00	0.00	C
ATOM	2156	O	THR	A	156	50.369	29.435	45.641	1.00	0.00	O
ATOM	2157	N	THR	A	157	51.811	28.165	44.474	1.00	0.00	N

ATOM	2158	H	THR	A	157	51.958	27.289	44.014	1.00	0.00	H
ATOM	2159	CA	THR	A	157	52.818	29.219	44.331	1.00	0.00	C
ATOM	2160	HA	THR	A	157	52.969	29.518	45.273	1.00	0.00	H
ATOM	2161	CB	THR	A	157	54.079	28.663	43.714	1.00	0.00	C
ATOM	2162	HB	THR	A	157	53.838	28.287	42.820	1.00	0.00	H
ATOM	2163	CG2	THR	A	157	55.220	29.717	43.532	1.00	0.00	C
ATOM	2164	HG21	THR	A	157	56.018	29.277	43.120	1.00	0.00	H
ATOM	2165	HG22	THR	A	157	54.901	30.454	42.936	1.00	0.00	H
ATOM	2166	HG23	THR	A	157	55.471	30.094	44.424	1.00	0.00	H
ATOM	2167	OG1	THR	A	157	54.509	27.537	44.429	1.00	0.00	O
ATOM	2168	HG1	THR	A	157	55.344	27.177	44.013	1.00	0.00	H
ATOM	2169	C	THR	A	157	52.346	30.397	43.444	1.00	0.00	C
ATOM	2170	O	THR	A	157	52.754	31.550	43.717	1.00	0.00	O
ATOM	2171	N	LEU	A	158	51.524	30.199	42.404	1.00	0.00	N
ATOM	2172	H	LEU	A	158	51.276	29.262	42.158	1.00	0.00	H
ATOM	2173	CA	LEU	A	158	50.993	31.269	41.640	1.00	0.00	C
ATOM	2174	HA	LEU	A	158	51.785	31.868	41.525	1.00	0.00	H
ATOM	2175	CB	LEU	A	158	50.456	30.729	40.272	1.00	0.00	C
ATOM	2176	HB1	LEU	A	158	49.587	30.257	40.419	1.00	0.00	H
ATOM	2177	HB2	LEU	A	158	51.120	30.091	39.882	1.00	0.00	H
ATOM	2178	CG	LEU	A	158	50.230	31.874	39.274	1.00	0.00	C
ATOM	2179	HG	LEU	A	158	49.745	32.566	39.809	1.00	0.00	H
ATOM	2180	CD1	LEU	A	158	51.423	32.664	38.842	1.00	0.00	C
ATOM	2181	HD11	LEU	A	158	51.138	33.373	38.197	1.00	0.00	H
ATOM	2182	HD12	LEU	A	158	51.847	33.091	39.641	1.00	0.00	H
ATOM	2183	HD13	LEU	A	158	52.083	32.056	38.400	1.00	0.00	H
ATOM	2184	CD2	LEU	A	158	49.371	31.429	38.090	1.00	0.00	C
ATOM	2185	HD21	LEU	A	158	49.245	32.197	37.462	1.00	0.00	H
ATOM	2186	HD22	LEU	A	158	49.827	30.679	37.611	1.00	0.00	H
ATOM	2187	HD23	LEU	A	158	48.480	31.120	38.422	1.00	0.00	H
ATOM	2188	C	LEU	A	158	49.827	31.992	42.414	1.00	0.00	C
ATOM	2189	O	LEU	A	158	49.780	33.238	42.446	1.00	0.00	O
ATOM	2190	N	VAL	A	159	48.896	31.189	43.039	1.00	0.00	N
ATOM	2191	H	VAL	A	159	48.922	30.202	42.879	1.00	0.00	H
ATOM	2192	CA	VAL	A	159	47.855	31.744	43.943	1.00	0.00	C
ATOM	2193	HA	VAL	A	159	47.190	32.227	43.373	1.00	0.00	H
ATOM	2194	CB	VAL	A	159	47.035	30.674	44.676	1.00	0.00	C
ATOM	2195	HB	VAL	A	159	47.723	30.006	44.958	1.00	0.00	H
ATOM	2196	CG1	VAL	A	159	46.329	31.129	45.937	1.00	0.00	C
ATOM	2197	HG11	VAL	A	159	45.827	30.360	46.334	1.00	0.00	H
ATOM	2198	HG12	VAL	A	159	47.004	31.462	46.595	1.00	0.00	H
ATOM	2199	HG13	VAL	A	159	45.690	31.865	45.714	1.00	0.00	H
ATOM	2200	CG2	VAL	A	159	46.001	30.042	43.771	1.00	0.00	C
ATOM	2201	HG21	VAL	A	159	45.488	29.352	44.282	1.00	0.00	H
ATOM	2202	HG22	VAL	A	159	45.373	30.746	43.439	1.00	0.00	H
ATOM	2203	HG23	VAL	A	159	46.458	29.610	42.994	1.00	0.00	H
ATOM	2204	C	VAL	A	159	48.588	32.676	44.933	1.00	0.00	C
ATOM	2205	O	VAL	A	159	48.078	33.777	45.205	1.00	0.00	O
ATOM	2206	N	GLN	A	160	49.739	32.254	45.461	1.00	0.00	N
ATOM	2207	H	GLN	A	160	50.085	31.345	45.227	1.00	0.00	H
ATOM	2208	CA	GLN	A	160	50.513	33.114	46.390	1.00	0.00	C
ATOM	2209	HA	GLN	A	160	49.857	33.545	47.010	1.00	0.00	H
ATOM	2210	CB	GLN	A	160	51.477	32.159	47.072	1.00	0.00	C
ATOM	2211	HB1	GLN	A	160	52.218	31.963	46.430	1.00	0.00	H

```
ATOM   2212  HB2 GLN A 160      50.980  31.315  47.274  1.00  0.00           H
ATOM   2213  CG  GLN A 160      52.092  32.690  48.382  1.00  0.00           C
ATOM   2214  HG1 GLN A 160      51.350  32.798  49.043  1.00  0.00           H
ATOM   2215  HG2 GLN A 160      52.497  33.583  48.186  1.00  0.00           H
ATOM   2216  CD  GLN A 160      53.167  31.857  49.058  1.00  0.00           C
ATOM   2217  OE1 GLN A 160      53.490  30.781  48.706  1.00  0.00           O
ATOM   2218  NE2 GLN A 160      53.765  32.364  50.130  1.00  0.00           N
ATOM   2219 HE21 GLN A 160      53.503  33.268  50.468  1.00  0.00           H
ATOM   2220 HE22 GLN A 160      54.477  31.842  50.599  1.00  0.00           H
ATOM   2221  C   GLN A 160      51.212  34.345  45.797  1.00  0.00           C
ATOM   2222  O   GLN A 160      51.326  35.379  46.417  1.00  0.00           O
ATOM   2223  N   ASP A 161      51.698  34.224  44.593  1.00  0.00           N
ATOM   2224  H   ASP A 161      51.625  33.330  44.150  1.00  0.00           H
ATOM   2225  CA  ASP A 161      52.333  35.288  43.854  1.00  0.00           C
ATOM   2226  HA  ASP A 161      52.969  35.729  44.487  1.00  0.00           H
ATOM   2227  CB  ASP A 161      53.044  34.639  42.613  1.00  0.00           C
ATOM   2228  HB1 ASP A 161      53.138  35.326  41.892  1.00  0.00           H
ATOM   2229  HB2 ASP A 161      52.489  33.878  42.278  1.00  0.00           H
ATOM   2230  CG  ASP A 161      54.411  34.095  42.871  1.00  0.00           C
ATOM   2231  OD1 ASP A 161      55.122  33.721  41.892  1.00  0.00           O
ATOM   2232  OD2 ASP A 161      54.860  34.052  44.042  1.00  0.00           O
ATOM   2233  C   ASP A 161      51.286  36.327  43.389  1.00  0.00           C
ATOM   2234  O   ASP A 161      51.597  37.544  43.353  1.00  0.00           O
ATOM   2235  N   LEU A 162      50.000  35.938  43.029  1.00  0.00           N
ATOM   2236  H   LEU A 162      49.735  34.975  43.081  1.00  0.00           H
ATOM   2237  CA  LEU A 162      49.068  36.905  42.590  1.00  0.00           C
ATOM   2238  HA  LEU A 162      49.566  37.554  42.015  1.00  0.00           H
ATOM   2239  CB  LEU A 162      47.966  36.218  41.784  1.00  0.00           C
ATOM   2240  HB1 LEU A 162      47.171  36.824  41.757  1.00  0.00           H
ATOM   2241  HB2 LEU A 162      47.721  35.367  42.248  1.00  0.00           H
ATOM   2242  CG  LEU A 162      48.439  35.902  40.316  1.00  0.00           C
ATOM   2243  HG  LEU A 162      49.424  35.727  40.300  1.00  0.00           H
ATOM   2244  CD1 LEU A 162      47.563  34.688  39.813  1.00  0.00           C
ATOM   2245 HD11 LEU A 162      47.829  34.450  38.879  1.00  0.00           H
ATOM   2246 HD12 LEU A 162      47.709  33.901  40.412  1.00  0.00           H
ATOM   2247 HD13 LEU A 162      46.597  34.945  39.829  1.00  0.00           H
ATOM   2248  CD2 LEU A 162      48.412  37.044  39.374  1.00  0.00           C
ATOM   2249 HD21 LEU A 162      48.728  36.742  38.475  1.00  0.00           H
ATOM   2250 HD22 LEU A 162      47.478  37.392  39.299  1.00  0.00           H
ATOM   2251 HD23 LEU A 162      49.012  37.769  39.712  1.00  0.00           H
ATOM   2252  C   LEU A 162      48.493  37.589  43.816  1.00  0.00           C
ATOM   2253  O   LEU A 162      48.113  38.764  43.692  1.00  0.00           O
ATOM   2254  N   ALA A 163      48.340  36.936  44.988  1.00  0.00           N
ATOM   2255  H   ALA A 163      48.517  35.952  45.004  1.00  0.00           H
ATOM   2256  CA  ALA A 163      47.938  37.570  46.212  1.00  0.00           C
ATOM   2257  HA  ALA A 163      47.086  38.083  46.106  1.00  0.00           H
ATOM   2258  CB  ALA A 163      47.681  36.420  47.148  1.00  0.00           C
ATOM   2259  HB1 ALA A 163      47.392  36.773  48.038  1.00  0.00           H
ATOM   2260  HB2 ALA A 163      46.961  35.838  46.771  1.00  0.00           H
ATOM   2261  HB3 ALA A 163      48.519  35.886  47.257  1.00  0.00           H
ATOM   2262  C   ALA A 163      48.931  38.669  46.642  1.00  0.00           C
ATOM   2263  O   ALA A 163      48.430  39.782  47.008  1.00  0.00           O
ATOM   2264  N   ASN A 164      50.230  38.328  46.540  1.00  0.00           N
ATOM   2265  H   ASN A 164      50.470  37.381  46.324  1.00  0.00           H
```

ATOM	2266	CA	ASN	A	164	51.301	39.309	46.737	1.00	0.00	C
ATOM	2267	HA	ASN	A	164	51.106	39.568	47.683	1.00	0.00	H
ATOM	2268	CB	ASN	A	164	52.738	38.696	46.541	1.00	0.00	C
ATOM	2269	HB1	ASN	A	164	52.874	38.494	45.571	1.00	0.00	H
ATOM	2270	HB2	ASN	A	164	52.804	37.850	47.070	1.00	0.00	H
ATOM	2271	CG	ASN	A	164	53.882	39.603	46.984	1.00	0.00	C
ATOM	2272	OD1	ASN	A	164	53.881	40.822	46.846	1.00	0.00	O
ATOM	2273	ND2	ASN	A	164	54.954	39.020	47.458	1.00	0.00	N
ATOM	2274	HD21	ASN	A	164	54.996	38.023	47.517	1.00	0.00	H
ATOM	2275	HD22	ASN	A	164	55.731	39.572	47.761	1.00	0.00	H
ATOM	2276	C	ASN	A	164	51.192	40.563	45.886	1.00	0.00	C
ATOM	2277	O	ASN	A	164	50.739	41.551	46.456	1.00	0.00	O
ATOM	2278	N	ALA	A	165	51.288	40.473	44.519	1.00	0.00	N
ATOM	2279	H	ALA	A	165	51.513	39.568	44.157	1.00	0.00	H
ATOM	2280	CA	ALA	A	165	51.108	41.507	43.544	1.00	0.00	C
ATOM	2281	HA	ALA	A	165	51.883	42.138	43.580	1.00	0.00	H
ATOM	2282	CB	ALA	A	165	50.998	40.743	42.188	1.00	0.00	C
ATOM	2283	HB1	ALA	A	165	50.869	41.400	41.445	1.00	0.00	H
ATOM	2284	HB2	ALA	A	165	51.837	40.221	42.030	1.00	0.00	H
ATOM	2285	HB3	ALA	A	165	50.218	40.118	42.220	1.00	0.00	H
ATOM	2286	C	ALA	A	165	49.882	42.364	43.871	1.00	0.00	C
ATOM	2287	O	ALA	A	165	49.991	43.562	44.014	1.00	0.00	O
ATOM	2288	N	PHE	A	166	48.695	41.781	44.021	1.00	0.00	N
ATOM	2289	H	PHE	A	166	48.665	40.785	43.932	1.00	0.00	H
ATOM	2290	CA	PHE	A	166	47.459	42.443	44.297	1.00	0.00	C
ATOM	2291	HA	PHE	A	166	47.404	43.160	43.602	1.00	0.00	H
ATOM	2292	CB	PHE	A	166	46.280	41.434	44.189	1.00	0.00	C
ATOM	2293	HB1	PHE	A	166	45.481	41.850	44.624	1.00	0.00	H
ATOM	2294	HB2	PHE	A	166	46.539	40.607	44.689	1.00	0.00	H
ATOM	2295	CG	PHE	A	166	45.869	41.009	42.753	1.00	0.00	C
ATOM	2296	CD1	PHE	A	166	46.589	41.297	41.591	1.00	0.00	C
ATOM	2297	HD1	PHE	A	166	47.448	41.796	41.701	1.00	0.00	H
ATOM	2298	CE1	PHE	A	166	46.223	40.956	40.308	1.00	0.00	C
ATOM	2299	HE1	PHE	A	166	46.812	41.144	39.522	1.00	0.00	H
ATOM	2300	CZ	PHE	A	166	45.002	40.342	40.172	1.00	0.00	C
ATOM	2301	HZ	PHE	A	166	44.645	40.186	39.251	1.00	0.00	H
ATOM	2302	CE2	PHE	A	166	44.236	39.925	41.267	1.00	0.00	C
ATOM	2303	HE2	PHE	A	166	43.380	39.426	41.128	1.00	0.00	H
ATOM	2304	CD2	PHE	A	166	44.688	40.218	42.562	1.00	0.00	C
ATOM	2305	HD2	PHE	A	166	44.185	39.874	43.355	1.00	0.00	H
ATOM	2306	C	PHE	A	166	47.474	43.179	45.587	1.00	0.00	C
ATOM	2307	O	PHE	A	166	47.009	44.324	45.566	1.00	0.00	O
ATOM	2308	N	GLN	A	167	47.892	42.619	46.759	1.00	0.00	N
ATOM	2309	H	GLN	A	167	48.025	41.628	46.750	1.00	0.00	H
ATOM	2310	CA	GLN	A	167	48.170	43.298	48.033	1.00	0.00	C
ATOM	2311	HA	GLN	A	167	47.278	43.642	48.327	1.00	0.00	H
ATOM	2312	CB	GLN	A	167	48.788	42.296	49.059	1.00	0.00	C
ATOM	2313	HB1	GLN	A	167	49.517	41.780	48.610	1.00	0.00	H
ATOM	2314	HB2	GLN	A	167	48.075	41.666	49.367	1.00	0.00	H
ATOM	2315	CG	GLN	A	167	49.383	42.867	50.278	1.00	0.00	C
ATOM	2316	HG1	GLN	A	167	50.133	43.466	49.996	1.00	0.00	H
ATOM	2317	HG2	GLN	A	167	49.747	42.110	50.820	1.00	0.00	H
ATOM	2318	CD	GLN	A	167	48.445	43.656	51.139	1.00	0.00	C
ATOM	2319	OE1	GLN	A	167	47.244	43.735	50.925	1.00	0.00	O

ATOM	2320	NE2	GLN	A	167	48.952	44.448	52.070	1.00	0.00	N
ATOM	2321	HE21	GLN	A	167	49.943	44.511	52.188	1.00	0.00	H
ATOM	2322	HE22	GLN	A	167	48.344	44.984	52.656	1.00	0.00	H
ATOM	2323	C	GLN	A	167	49.139	44.474	47.850	1.00	0.00	C
ATOM	2324	O	GLN	A	167	48.832	45.629	48.168	1.00	0.00	O
ATOM	2325	N	GLN	A	168	50.315	44.223	47.244	1.00	0.00	N
ATOM	2326	H	GLN	A	168	50.451	43.299	46.888	1.00	0.00	H
ATOM	2327	CA	GLN	A	168	51.397	45.157	47.059	1.00	0.00	C
ATOM	2328	HA	GLN	A	168	51.449	45.562	47.972	1.00	0.00	H
ATOM	2329	CB	GLN	A	168	52.676	44.373	46.597	1.00	0.00	C
ATOM	2330	HB1	GLN	A	168	52.519	44.039	45.668	1.00	0.00	H
ATOM	2331	HB2	GLN	A	168	52.806	43.596	47.213	1.00	0.00	H
ATOM	2332	CG	GLN	A	168	53.995	45.192	46.587	1.00	0.00	C
ATOM	2333	HG1	GLN	A	168	53.972	45.812	45.803	1.00	0.00	H
ATOM	2334	HG2	GLN	A	168	54.758	44.554	46.485	1.00	0.00	H
ATOM	2335	CD	GLN	A	168	54.274	46.036	47.823	1.00	0.00	C
ATOM	2336	OE1	GLN	A	168	54.091	45.572	48.963	1.00	0.00	O
ATOM	2337	NE2	GLN	A	168	54.625	47.286	47.661	1.00	0.00	N
ATOM	2338	HE21	GLN	A	168	54.705	47.668	46.740	1.00	0.00	H
ATOM	2339	HE22	GLN	A	168	54.813	47.860	48.458	1.00	0.00	H
ATOM	2340	C	GLN	A	168	51.128	46.316	46.077	1.00	0.00	C
ATOM	2341	O	GLN	A	168	51.658	47.417	46.233	1.00	0.00	O
ATOM	2342	N	GLU	A	169	50.154	46.130	45.140	1.00	0.00	N
ATOM	2343	H	GLU	A	169	49.795	45.204	45.024	1.00	0.00	H
ATOM	2344	CA	GLU	A	169	49.591	47.189	44.287	1.00	0.00	C
ATOM	2345	HA	GLU	A	169	50.371	47.793	44.121	1.00	0.00	H
ATOM	2346	CB	GLU	A	169	49.066	46.626	42.920	1.00	0.00	C
ATOM	2347	HB1	GLU	A	169	48.275	46.053	43.134	1.00	0.00	H
ATOM	2348	HB2	GLU	A	169	49.799	46.057	42.547	1.00	0.00	H
ATOM	2349	CG	GLU	A	169	48.620	47.611	41.769	1.00	0.00	C
ATOM	2350	HG1	GLU	A	169	48.578	47.116	40.901	1.00	0.00	H
ATOM	2351	HG2	GLU	A	169	49.279	48.360	41.694	1.00	0.00	H
ATOM	2352	CD	GLU	A	169	47.231	48.209	42.058	1.00	0.00	C
ATOM	2353	OE1	GLU	A	169	46.502	47.771	42.974	1.00	0.00	O
ATOM	2354	OE2	GLU	A	169	46.806	49.002	41.258	1.00	0.00	O
ATOM	2355	C	GLU	A	169	48.673	48.130	44.988	1.00	0.00	C
ATOM	2356	O	GLU	A	169	48.592	49.351	44.844	1.00	0.00	O
ATOM	2357	N	ALA	A	170	47.907	47.568	45.940	1.00	0.00	N
ATOM	2358	H	ALA	A	170	47.902	46.572	46.027	1.00	0.00	H
ATOM	2359	CA	ALA	A	170	47.119	48.322	46.812	1.00	0.00	C
ATOM	2360	HA	ALA	A	170	46.689	48.993	46.208	1.00	0.00	H
ATOM	2361	CB	ALA	A	170	46.008	47.459	47.366	1.00	0.00	C
ATOM	2362	HB1	ALA	A	170	45.445	48.001	47.990	1.00	0.00	H
ATOM	2363	HB2	ALA	A	170	45.442	47.121	46.614	1.00	0.00	H
ATOM	2364	HB3	ALA	A	170	46.403	46.684	47.860	1.00	0.00	H
ATOM	2365	C	ALA	A	170	47.995	49.075	47.908	1.00	0.00	C
ATOM	2366	O	ALA	A	170	47.673	50.228	48.236	1.00	0.00	O
ATOM	2367	N	GLN	A	171	49.147	48.579	48.256	1.00	0.00	N
ATOM	2368	H	GLN	A	171	49.354	47.622	48.053	1.00	0.00	H
ATOM	2369	CA	GLN	A	171	50.129	49.393	48.931	1.00	0.00	C
ATOM	2370	HA	GLN	A	171	49.617	49.900	49.625	1.00	0.00	H
ATOM	2371	CB	GLN	A	171	51.193	48.437	49.536	1.00	0.00	C
ATOM	2372	HB1	GLN	A	171	51.967	48.987	49.851	1.00	0.00	H
ATOM	2373	HB2	GLN	A	171	51.498	47.812	48.818	1.00	0.00	H

ATOM	2374	CG	GLN	A	171	50.648	47.593	50.743	1.00	0.00	C
ATOM	2375	HG1	GLN	A	171	51.394	47.073	51.159	1.00	0.00	H
ATOM	2376	HG2	GLN	A	171	49.939	46.963	50.425	1.00	0.00	H
ATOM	2377	CD	GLN	A	171	50.046	48.509	51.800	1.00	0.00	C
ATOM	2378	OE1	GLN	A	171	48.904	48.905	51.693	1.00	0.00	O
ATOM	2379	NE2	GLN	A	171	50.859	48.900	52.758	1.00	0.00	N
ATOM	2380	HE21	GLN	A	171	51.811	48.595	52.761	1.00	0.00	H
ATOM	2381	HE22	GLN	A	171	50.525	49.503	53.483	1.00	0.00	H
ATOM	2382	C	GLN	A	171	50.697	50.519	48.120	1.00	0.00	C
ATOM	2383	O	GLN	A	171	51.460	51.259	48.634	1.00	0.00	O
ATOM	2384	N	THR	A	172	50.372	50.576	46.778	1.00	0.00	N
ATOM	2385	H	THR	A	172	49.693	49.928	46.431	1.00	0.00	H
ATOM	2386	CA	THR	A	172	50.972	51.545	45.814	1.00	0.00	C
ATOM	2387	HA	THR	A	172	51.627	52.107	46.319	1.00	0.00	H
ATOM	2388	CB	THR	A	172	51.807	50.839	44.759	1.00	0.00	C
ATOM	2389	HB	THR	A	172	51.167	50.211	44.316	1.00	0.00	H
ATOM	2390	CG2	THR	A	172	52.486	51.735	43.747	1.00	0.00	C
ATOM	2391	HG21	THR	A	172	53.006	51.175	43.102	1.00	0.00	H
ATOM	2392	HG22	THR	A	172	51.795	52.260	43.250	1.00	0.00	H
ATOM	2393	HG23	THR	A	172	53.105	52.362	44.219	1.00	0.00	H
ATOM	2394	OG1	THR	A	172	52.822	50.080	45.311	1.00	0.00	O
ATOM	2395	HG1	THR	A	172	53.342	49.637	44.581	1.00	0.00	H
ATOM	2396	C	THR	A	172	49.927	52.526	45.281	1.00	0.00	C
ATOM	2397	O	THR	A	172	49.947	53.713	45.625	1.00	0.00	O
ATOM	2398	N	SER	A	173	48.982	52.004	44.446	1.00	0.00	N
ATOM	2399	H	SER	A	173	48.981	51.019	44.272	1.00	0.00	H
ATOM	2400	CA	SER	A	173	48.000	52.815	43.816	1.00	0.00	C
ATOM	2401	HA	SER	A	173	48.529	53.539	43.373	1.00	0.00	H
ATOM	2402	CB	SER	A	173	47.182	51.882	42.876	1.00	0.00	C
ATOM	2403	HB1	SER	A	173	47.790	51.296	42.341	1.00	0.00	H
ATOM	2404	HB2	SER	A	173	46.606	52.417	42.258	1.00	0.00	H
ATOM	2405	OG	SER	A	173	46.319	51.020	43.546	1.00	0.00	O
ATOM	2406	HG	SER	A	173	45.830	50.455	42.882	1.00	0.00	H
ATOM	2407	C	SER	A	173	47.004	53.421	44.822	1.00	0.00	C
ATOM	2408	O	SER	A	173	46.429	54.492	44.608	1.00	0.00	O
ATOM	2409	N	GLY	A	174	46.725	52.737	45.953	1.00	0.00	N
ATOM	2410	H	GLY	A	174	47.214	51.872	46.068	1.00	0.00	H
ATOM	2411	CA	GLY	A	174	45.824	53.084	47.008	1.00	0.00	C
ATOM	2412	HA1	GLY	A	174	46.010	54.039	47.240	1.00	0.00	H
ATOM	2413	HA2	GLY	A	174	46.057	52.500	47.786	1.00	0.00	H
ATOM	2414	C	GLY	A	174	44.321	52.898	46.611	1.00	0.00	C
ATOM	2415	O	GLY	A	174	43.412	53.208	47.443	1.00	0.00	O
ATOM	2416	N	LYS	A	175	44.113	52.202	45.458	1.00	0.00	N
ATOM	2417	H	LYS	A	175	44.924	51.907	44.952	1.00	0.00	H
ATOM	2418	CA	LYS	A	175	42.858	51.860	44.913	1.00	0.00	C
ATOM	2419	HA	LYS	A	175	42.309	52.690	45.009	1.00	0.00	H
ATOM	2420	CB	LYS	A	175	42.990	51.620	43.421	1.00	0.00	C
ATOM	2421	HB1	LYS	A	175	42.105	51.276	43.108	1.00	0.00	H
ATOM	2422	HB2	LYS	A	175	43.683	50.909	43.306	1.00	0.00	H
ATOM	2423	CG	LYS	A	175	43.390	52.855	42.527	1.00	0.00	C
ATOM	2424	HG1	LYS	A	175	44.289	53.196	42.800	1.00	0.00	H
ATOM	2425	HG2	LYS	A	175	42.712	53.583	42.628	1.00	0.00	H
ATOM	2426	CD	LYS	A	175	43.453	52.464	41.064	1.00	0.00	C
ATOM	2427	HD1	LYS	A	175	42.736	51.790	40.886	1.00	0.00	H

ATOM	2428	HD2	LYS	A	175	44.348	52.055	40.884	1.00	0.00	H
ATOM	2429	CE	LYS	A	175	43.253	53.672	40.130	1.00	0.00	C
ATOM	2430	HE1	LYS	A	175	43.983	54.341	40.272	1.00	0.00	H
ATOM	2431	HE2	LYS	A	175	42.368	54.102	40.308	1.00	0.00	H
ATOM	2432	NZ	LYS	A	175	43.282	53.262	38.725	1.00	0.00	N
ATOM	2433	HZ1	LYS	A	175	43.150	54.063	38.141	1.00	0.00	H
ATOM	2434	HZ2	LYS	A	175	44.165	52.841	38.519	1.00	0.00	H
ATOM	2435	HZ3	LYS	A	175	42.550	52.602	38.555	1.00	0.00	H
ATOM	2436	C	LYS	A	175	42.167	50.705	45.738	1.00	0.00	C
ATOM	2437	O	LYS	A	175	42.688	50.167	46.676	1.00	0.00	O
ATOM	2438	N	GLU	A	176	40.986	50.253	45.247	1.00	0.00	N
ATOM	2439	H	GLU	A	176	40.506	50.761	44.532	1.00	0.00	H
ATOM	2440	CA	GLU	A	176	40.459	49.084	45.749	1.00	0.00	C
ATOM	2441	HA	GLU	A	176	40.613	49.349	46.701	1.00	0.00	H
ATOM	2442	CB	GLU	A	176	38.964	48.889	45.377	1.00	0.00	C
ATOM	2443	HB1	GLU	A	176	38.740	47.914	45.373	1.00	0.00	H
ATOM	2444	HB2	GLU	A	176	38.787	49.275	44.472	1.00	0.00	H
ATOM	2445	CG	GLU	A	176	38.071	49.578	46.371	1.00	0.00	C
ATOM	2446	HG1	GLU	A	176	38.450	49.463	47.289	1.00	0.00	H
ATOM	2447	HG2	GLU	A	176	37.159	49.169	46.333	1.00	0.00	H
ATOM	2448	CD	GLU	A	176	37.979	51.056	46.040	1.00	0.00	C
ATOM	2449	OE1	GLU	A	176	38.405	51.936	46.787	1.00	0.00	O
ATOM	2450	OE2	GLU	A	176	37.469	51.350	44.926	1.00	0.00	O
ATOM	2451	C	GLU	A	176	41.240	47.789	45.469	1.00	0.00	C
ATOM	2452	O	GLU	A	176	41.667	47.598	44.361	1.00	0.00	O
ATOM	2453	N	ARG	A	177	41.394	46.787	46.411	1.00	0.00	N
ATOM	2454	H	ARG	A	177	40.869	46.866	47.259	1.00	0.00	H
ATOM	2455	CA	ARG	A	177	42.242	45.652	46.267	1.00	0.00	C
ATOM	2456	HA	ARG	A	177	43.074	46.128	45.984	1.00	0.00	H
ATOM	2457	CB	ARG	A	177	42.509	44.823	47.541	1.00	0.00	C
ATOM	2458	HB1	ARG	A	177	41.876	44.049	47.524	1.00	0.00	H
ATOM	2459	HB2	ARG	A	177	42.296	45.411	48.322	1.00	0.00	H
ATOM	2460	CG	ARG	A	177	43.973	44.257	47.735	1.00	0.00	C
ATOM	2461	HG1	ARG	A	177	44.255	44.436	48.678	1.00	0.00	H
ATOM	2462	HG2	ARG	A	177	44.581	44.743	47.107	1.00	0.00	H
ATOM	2463	CD	ARG	A	177	44.242	42.799	47.506	1.00	0.00	C
ATOM	2464	HD1	ARG	A	177	45.233	42.665	47.499	1.00	0.00	H
ATOM	2465	HD2	ARG	A	177	43.865	42.551	46.613	1.00	0.00	H
ATOM	2466	NE	ARG	A	177	43.701	41.845	48.464	1.00	0.00	N
ATOM	2467	HE	ARG	A	177	44.116	41.863	49.373	1.00	0.00	H
ATOM	2468	CZ	ARG	A	177	42.708	40.933	48.290	1.00	0.00	C
ATOM	2469	NH1	ARG	A	177	42.150	40.698	47.066	1.00	0.00	N
ATOM	2470	HH11	ARG	A	177	42.471	41.204	46.265	1.00	0.00	H
ATOM	2471	HH12	ARG	A	177	41.421	40.020	46.971	1.00	0.00	H
ATOM	2472	NH2	ARG	A	177	42.194	40.216	49.250	1.00	0.00	N
ATOM	2473	HH21	ARG	A	177	42.530	40.320	50.186	1.00	0.00	H
ATOM	2474	HH22	ARG	A	177	41.464	39.562	49.050	1.00	0.00	H
ATOM	2475	C	ARG	A	177	41.710	44.709	45.228	1.00	0.00	C
ATOM	2476	O	ARG	A	177	40.515	44.295	45.216	1.00	0.00	O
ATOM	2477	N	LEU	A	178	42.531	44.395	44.264	1.00	0.00	N
ATOM	2478	H	LEU	A	178	43.468	44.743	44.300	1.00	0.00	H
ATOM	2479	CA	LEU	A	178	42.138	43.554	43.132	1.00	0.00	C
ATOM	2480	HA	LEU	A	178	41.344	44.000	42.719	1.00	0.00	H
ATOM	2481	CB	LEU	A	178	43.227	43.435	42.038	1.00	0.00	C

ATOM	2482	HB1	LEU	A	178	42.937	42.762	41.357	1.00	0.00	H
ATOM	2483	HB2	LEU	A	178	44.084	43.137	42.458	1.00	0.00	H
ATOM	2484	CG	LEU	A	178	43.474	44.757	41.331	1.00	0.00	C
ATOM	2485	HG	LEU	A	178	43.465	45.528	41.968	1.00	0.00	H
ATOM	2486	CD1	LEU	A	178	44.799	44.615	40.672	1.00	0.00	C
ATOM	2487	HD11	LEU	A	178	45.021	45.460	40.185	1.00	0.00	H
ATOM	2488	HD12	LEU	A	178	45.498	44.435	41.364	1.00	0.00	H
ATOM	2489	HD13	LEU	A	178	44.769	43.854	40.023	1.00	0.00	H
ATOM	2490	CD2	LEU	A	178	42.409	45.046	40.311	1.00	0.00	C
ATOM	2491	HD21	LEU	A	178	42.602	45.921	39.866	1.00	0.00	H
ATOM	2492	HD22	LEU	A	178	42.398	44.318	39.625	1.00	0.00	H
ATOM	2493	HD23	LEU	A	178	41.518	45.091	40.763	1.00	0.00	H
ATOM	2494	C	LEU	A	178	41.789	42.195	43.683	1.00	0.00	C
ATOM	2495	O	LEU	A	178	42.600	41.503	44.361	1.00	0.00	O
ATOM	2496	N	LEU	A	179	40.573	41.726	43.433	1.00	0.00	N
ATOM	2497	H	LEU	A	179	39.943	42.289	42.899	1.00	0.00	H
ATOM	2498	CA	LEU	A	179	40.110	40.404	43.912	1.00	0.00	C
ATOM	2499	HA	LEU	A	179	40.453	40.398	44.851	1.00	0.00	H
ATOM	2500	CB	LEU	A	179	38.563	40.252	43.790	1.00	0.00	C
ATOM	2501	HB1	LEU	A	179	38.334	39.307	44.023	1.00	0.00	H
ATOM	2502	HB2	LEU	A	179	38.318	40.429	42.837	1.00	0.00	H
ATOM	2503	CG	LEU	A	179	37.685	41.185	44.685	1.00	0.00	C
ATOM	2504	HG	LEU	A	179	37.974	42.105	44.419	1.00	0.00	H
ATOM	2505	CD1	LEU	A	179	36.194	41.119	44.399	1.00	0.00	C
ATOM	2506	HD11	LEU	A	179	35.709	41.743	45.012	1.00	0.00	H
ATOM	2507	HD12	LEU	A	179	36.024	41.385	43.450	1.00	0.00	H
ATOM	2508	HD13	LEU	A	179	35.867	40.185	44.547	1.00	0.00	H
ATOM	2509	CD2	LEU	A	179	37.929	41.011	46.205	1.00	0.00	C
ATOM	2510	HD21	LEU	A	179	37.336	41.637	46.712	1.00	0.00	H
ATOM	2511	HD22	LEU	A	179	37.723	40.069	46.470	1.00	0.00	H
ATOM	2512	HD23	LEU	A	179	38.885	41.215	46.415	1.00	0.00	H
ATOM	2513	C	LEU	A	179	40.686	39.243	43.123	1.00	0.00	C
ATOM	2514	O	LEU	A	179	40.820	39.122	41.910	1.00	0.00	O
ATOM	2515	N	LEU	A	180	40.883	38.157	43.882	1.00	0.00	N
ATOM	2516	H	LEU	A	180	40.659	38.203	44.855	1.00	0.00	H
ATOM	2517	CA	LEU	A	180	41.416	36.902	43.338	1.00	0.00	C
ATOM	2518	HA	LEU	A	180	41.475	36.988	42.343	1.00	0.00	H
ATOM	2519	CB	LEU	A	180	42.793	36.635	43.929	1.00	0.00	C
ATOM	2520	HB1	LEU	A	180	42.685	36.547	44.919	1.00	0.00	H
ATOM	2521	HB2	LEU	A	180	43.368	37.428	43.726	1.00	0.00	H
ATOM	2522	CG	LEU	A	180	43.524	35.393	43.431	1.00	0.00	C
ATOM	2523	HG	LEU	A	180	42.894	34.630	43.574	1.00	0.00	H
ATOM	2524	CD1	LEU	A	180	43.933	35.424	41.956	1.00	0.00	C
ATOM	2525	HD11	LEU	A	180	44.403	34.573	41.722	1.00	0.00	H
ATOM	2526	HD12	LEU	A	180	43.117	35.522	41.386	1.00	0.00	H
ATOM	2527	HD13	LEU	A	180	44.546	36.198	41.796	1.00	0.00	H
ATOM	2528	CD2	LEU	A	180	44.788	35.260	44.194	1.00	0.00	C
ATOM	2529	HD21	LEU	A	180	45.283	34.449	43.881	1.00	0.00	H
ATOM	2530	HD22	LEU	A	180	45.353	36.072	44.048	1.00	0.00	H
ATOM	2531	HD23	LEU	A	180	44.581	35.169	45.168	1.00	0.00	H
ATOM	2532	C	LEU	A	180	40.484	35.786	43.680	1.00	0.00	C
ATOM	2533	O	LEU	A	180	40.176	35.377	44.837	1.00	0.00	O
ATOM	2534	N	SER	A	181	39.918	35.211	42.590	1.00	0.00	N
ATOM	2535	H	SER	A	181	40.245	35.535	41.702	1.00	0.00	H

ATOM	2536	CA	SER	A	181	38.907	34.194	42.557	1.00	0.00	C
ATOM	2537	HA	SER	A	181	38.781	33.886	43.500	1.00	0.00	H
ATOM	2538	CB	SER	A	181	37.675	34.845	41.829	1.00	0.00	C
ATOM	2539	HB1	SER	A	181	37.967	35.257	40.966	1.00	0.00	H
ATOM	2540	HB2	SER	A	181	37.265	35.547	42.412	1.00	0.00	H
ATOM	2541	OG	SER	A	181	36.598	34.037	41.468	1.00	0.00	O
ATOM	2542	HG	SER	A	181	35.900	34.594	41.018	1.00	0.00	H
ATOM	2543	C	SER	A	181	39.329	33.019	41.745	1.00	0.00	C
ATOM	2544	O	SER	A	181	40.187	33.190	40.916	1.00	0.00	O
ATOM	2545	N	ALA	A	182	38.661	31.831	42.061	1.00	0.00	N
ATOM	2546	H	ALA	A	182	37.946	31.818	42.760	1.00	0.00	H
ATOM	2547	CA	ALA	A	182	39.029	30.610	41.349	1.00	0.00	C
ATOM	2548	HA	ALA	A	182	39.296	30.896	40.429	1.00	0.00	H
ATOM	2549	CB	ALA	A	182	40.276	29.922	41.971	1.00	0.00	C
ATOM	2550	HB1	ALA	A	182	40.492	29.093	41.454	1.00	0.00	H
ATOM	2551	HB2	ALA	A	182	41.054	30.549	41.936	1.00	0.00	H
ATOM	2552	HB3	ALA	A	182	40.084	29.681	42.922	1.00	0.00	H
ATOM	2553	C	ALA	A	182	37.804	29.706	41.209	1.00	0.00	C
ATOM	2554	O	ALA	A	182	37.129	29.374	42.186	1.00	0.00	O
ATOM	2555	N	ALA	A	183	37.511	29.344	39.969	1.00	0.00	N
ATOM	2556	H	ALA	A	183	38.059	29.740	39.232	1.00	0.00	H
ATOM	2557	CA	ALA	A	183	36.462	28.422	39.596	1.00	0.00	C
ATOM	2558	HA	ALA	A	183	35.713	28.497	40.255	1.00	0.00	H
ATOM	2559	CB	ALA	A	183	35.968	28.830	38.215	1.00	0.00	C
ATOM	2560	HB1	ALA	A	183	35.239	28.210	37.925	1.00	0.00	H
ATOM	2561	HB2	ALA	A	183	35.616	29.765	38.250	1.00	0.00	H
ATOM	2562	HB3	ALA	A	183	36.725	28.782	37.563	1.00	0.00	H
ATOM	2563	C	ALA	A	183	36.983	26.984	39.657	1.00	0.00	C
ATOM	2564	O	ALA	A	183	38.171	26.733	39.454	1.00	0.00	O
ATOM	2565	N	VAL	A	184	36.120	26.045	40.054	1.00	0.00	N
ATOM	2566	H	VAL	A	184	35.150	26.274	40.129	1.00	0.00	H
ATOM	2567	CA	VAL	A	184	36.562	24.684	40.383	1.00	0.00	C
ATOM	2568	HA	VAL	A	184	37.364	24.560	39.799	1.00	0.00	H
ATOM	2569	CB	VAL	A	184	36.941	24.583	41.870	1.00	0.00	C
ATOM	2570	HB	VAL	A	184	37.235	23.628	41.907	1.00	0.00	H
ATOM	2571	CG1	VAL	A	184	38.085	25.520	42.340	1.00	0.00	C
ATOM	2572	HG11	VAL	A	184	38.253	25.378	43.315	1.00	0.00	H
ATOM	2573	HG12	VAL	A	184	38.917	25.314	41.825	1.00	0.00	H
ATOM	2574	HG13	VAL	A	184	37.822	26.472	42.183	1.00	0.00	H
ATOM	2575	CG2	VAL	A	184	35.794	24.786	42.839	1.00	0.00	C
ATOM	2576	HG21	VAL	A	184	36.131	24.703	43.777	1.00	0.00	H
ATOM	2577	HG22	VAL	A	184	35.401	25.696	42.705	1.00	0.00	H
ATOM	2578	HG23	VAL	A	184	35.092	24.093	42.675	1.00	0.00	H
ATOM	2579	C	VAL	A	184	35.534	23.593	40.019	1.00	0.00	C
ATOM	2580	O	VAL	A	184	34.340	23.821	40.203	1.00	0.00	O
ATOM	2581	N	PRO	A	185	35.945	22.396	39.552	1.00	0.00	N
ATOM	2582	CD	PRO	A	185	37.321	21.978	39.343	1.00	0.00	C
ATOM	2583	HD1	PRO	A	185	37.847	22.145	40.177	1.00	0.00	H
ATOM	2584	HD2	PRO	A	185	37.715	22.504	38.589	1.00	0.00	H
ATOM	2585	CG	PRO	A	185	37.272	20.487	39.010	1.00	0.00	C
ATOM	2586	HG1	PRO	A	185	37.340	19.930	39.838	1.00	0.00	H
ATOM	2587	HG2	PRO	A	185	38.004	20.235	38.377	1.00	0.00	H
ATOM	2588	CB	PRO	A	185	35.900	20.334	38.354	1.00	0.00	C
ATOM	2589	HB1	PRO	A	185	35.559	19.399	38.449	1.00	0.00	H

```
ATOM   2590  HB2 PRO A 185      35.936  20.579  37.385  1.00  0.00           H
ATOM   2591  CA  PRO A 185      35.033  21.322  39.142  1.00  0.00           C
ATOM   2592  HA  PRO A 185      34.302  21.725  38.591  1.00  0.00           H
ATOM   2593  C   PRO A 185      34.328  20.644  40.329  1.00  0.00           C
ATOM   2594  O   PRO A 185      34.827  20.634  41.456  1.00  0.00           O
ATOM   2595  N   ALA A 186      33.170  20.031  40.064  1.00  0.00           N
ATOM   2596  H   ALA A 186      32.885  19.944  39.109  1.00  0.00           H
ATOM   2597  CA  ALA A 186      32.297  19.480  41.105  1.00  0.00           C
ATOM   2598  HA  ALA A 186      32.690  19.798  41.968  1.00  0.00           H
ATOM   2599  CB  ALA A 186      30.893  20.056  40.878  1.00  0.00           C
ATOM   2600  HB1 ALA A 186      30.270  19.699  41.574  1.00  0.00           H
ATOM   2601  HB2 ALA A 186      30.928  21.053  40.942  1.00  0.00           H
ATOM   2602  HB3 ALA A 186      30.567  19.791  39.971  1.00  0.00           H
ATOM   2603  C   ALA A 186      32.320  17.942  41.258  1.00  0.00           C
ATOM   2604  O   ALA A 186      32.001  17.452  42.340  1.00  0.00           O
ATOM   2605  N   GLY A 187      32.712  17.166  40.239  1.00  0.00           N
ATOM   2606  H   GLY A 187      32.961  17.604  39.375  1.00  0.00           H
ATOM   2607  CA  GLY A 187      32.794  15.700  40.331  1.00  0.00           C
ATOM   2608  HA1 GLY A 187      32.856  15.344  39.399  1.00  0.00           H
ATOM   2609  HA2 GLY A 187      31.948  15.378  40.755  1.00  0.00           H
ATOM   2610  C   GLY A 187      33.996  15.203  41.149  1.00  0.00           C
ATOM   2611  O   GLY A 187      35.124  15.643  40.930  1.00  0.00           O
ATOM   2612  N   GLN A 188      33.790  14.205  42.020  1.00  0.00           N
ATOM   2613  H   GLN A 188      32.888  13.774  42.035  1.00  0.00           H
ATOM   2614  CA  GLN A 188      34.816  13.702  42.960  1.00  0.00           C
ATOM   2615  HA  GLN A 188      35.144  14.513  43.444  1.00  0.00           H
ATOM   2616  CB  GLN A 188      34.151  12.701  43.929  1.00  0.00           C
ATOM   2617  HB1 GLN A 188      33.806  11.925  43.401  1.00  0.00           H
ATOM   2618  HB2 GLN A 188      33.389  13.157  44.388  1.00  0.00           H
ATOM   2619  CG  GLN A 188      35.075  12.159  44.997  1.00  0.00           C
ATOM   2620  HG1 GLN A 188      34.548  12.059  45.841  1.00  0.00           H
ATOM   2621  HG2 GLN A 188      35.808  12.824  45.139  1.00  0.00           H
ATOM   2622  CD  GLN A 188      35.699  10.796  44.632  1.00  0.00           C
ATOM   2623  OE1 GLN A 188      35.801  10.429  43.510  1.00  0.00           O
ATOM   2624  NE2 GLN A 188      36.208  10.036  45.537  1.00  0.00           N
ATOM   2625 HE21 GLN A 188      36.203  10.326  46.494  1.00  0.00           H
ATOM   2626 HE22 GLN A 188      36.608   9.156  45.283  1.00  0.00           H
ATOM   2627  C   GLN A 188      36.067  13.111  42.281  1.00  0.00           C
ATOM   2628  O   GLN A 188      37.171  13.253  42.806  1.00  0.00           O
ATOM   2629  N   THR A 189      35.929  12.592  41.057  1.00  0.00           N
ATOM   2630  H   THR A 189      35.014  12.575  40.654  1.00  0.00           H
ATOM   2631  CA  THR A 189      37.045  12.044  40.267  1.00  0.00           C
ATOM   2632  HA  THR A 189      37.501  11.422  40.904  1.00  0.00           H
ATOM   2633  CB  THR A 189      36.503  11.295  39.040  1.00  0.00           C
ATOM   2634  HB  THR A 189      35.853  10.610  39.369  1.00  0.00           H
ATOM   2635  CG2 THR A 189      35.841  12.206  38.000  1.00  0.00           C
ATOM   2636 HG21 THR A 189      35.513  11.654  37.233  1.00  0.00           H
ATOM   2637 HG22 THR A 189      35.070  12.685  38.419  1.00  0.00           H
ATOM   2638 HG23 THR A 189      36.508  12.874  37.669  1.00  0.00           H
ATOM   2639  OG1 THR A 189      37.535  10.596  38.392  1.00  0.00           O
ATOM   2640  HG1 THR A 189      37.164  10.115  37.597  1.00  0.00           H
ATOM   2641  C   THR A 189      38.083  13.091  39.838  1.00  0.00           C
ATOM   2642  O   THR A 189      39.178  12.717  39.427  1.00  0.00           O
ATOM   2643  N   TYR A 190      37.764  14.388  39.919  1.00  0.00           N
```

ATOM	2644	H	TYR	A	190	36.832	14.616	40.200	1.00	0.00	H
ATOM	2645	CA	TYR	A	190	38.675	15.502	39.628	1.00	0.00	C
ATOM	2646	HA	TYR	A	190	39.416	15.102	39.088	1.00	0.00	H
ATOM	2647	CB	TYR	A	190	37.913	16.585	38.842	1.00	0.00	C
ATOM	2648	HB1	TYR	A	190	38.541	17.342	38.660	1.00	0.00	H
ATOM	2649	HB2	TYR	A	190	37.155	16.910	39.408	1.00	0.00	H
ATOM	2650	CG	TYR	A	190	37.334	16.142	37.514	1.00	0.00	C
ATOM	2651	CD1	TYR	A	190	38.199	15.875	36.438	1.00	0.00	C
ATOM	2652	HD1	TYR	A	190	39.188	15.963	36.559	1.00	0.00	H
ATOM	2653	CE1	TYR	A	190	37.676	15.484	35.193	1.00	0.00	C
ATOM	2654	HE1	TYR	A	190	38.295	15.293	34.431	1.00	0.00	H
ATOM	2655	CZ	TYR	A	190	36.283	15.359	35.012	1.00	0.00	C
ATOM	2656	OH	TYR	A	190	35.820	14.976	33.792	1.00	0.00	O
ATOM	2657	HH	TYR	A	190	34.821	14.931	33.814	1.00	0.00	H
ATOM	2658	CE2	TYR	A	190	35.413	15.633	36.092	1.00	0.00	C
ATOM	2659	HE2	TYR	A	190	34.424	15.547	35.972	1.00	0.00	H
ATOM	2660	CD2	TYR	A	190	35.940	16.027	37.339	1.00	0.00	C
ATOM	2661	HD2	TYR	A	190	35.323	16.225	38.101	1.00	0.00	H
ATOM	2662	C	TYR	A	190	39.274	16.112	40.905	1.00	0.00	C
ATOM	2663	O	TYR	A	190	39.977	17.111	40.883	1.00	0.00	O
ATOM	2664	N	VAL	A	191	38.895	15.662	42.113	1.00	0.00	N
ATOM	2665	H	VAL	A	191	38.539	14.731	42.189	1.00	0.00	H
ATOM	2666	CA	VAL	A	191	38.982	16.472	43.306	1.00	0.00	C
ATOM	2667	HA	VAL	A	191	39.657	17.163	43.049	1.00	0.00	H
ATOM	2668	CB	VAL	A	191	37.619	17.222	43.628	1.00	0.00	C
ATOM	2669	HB	VAL	A	191	36.875	16.554	43.603	1.00	0.00	H
ATOM	2670	CG1	VAL	A	191	37.708	17.894	45.041	1.00	0.00	C
ATOM	2671	HG11	VAL	A	191	36.849	18.365	45.241	1.00	0.00	H
ATOM	2672	HG12	VAL	A	191	37.872	17.192	45.734	1.00	0.00	H
ATOM	2673	HG13	VAL	A	191	38.460	18.553	45.050	1.00	0.00	H
ATOM	2674	CG2	VAL	A	191	37.192	18.221	42.571	1.00	0.00	C
ATOM	2675	HG21	VAL	A	191	36.331	18.648	42.845	1.00	0.00	H
ATOM	2676	HG22	VAL	A	191	37.896	18.924	42.473	1.00	0.00	H
ATOM	2677	HG23	VAL	A	191	37.067	17.750	41.698	1.00	0.00	H
ATOM	2678	C	VAL	A	191	39.492	15.570	44.535	1.00	0.00	C
ATOM	2679	O	VAL	A	191	40.008	16.085	45.471	1.00	0.00	O
ATOM	2680	N	ASP	A	192	39.450	14.223	44.481	1.00	0.00	N
ATOM	2681	H	ASP	A	192	39.023	13.773	43.697	1.00	0.00	H
ATOM	2682	CA	ASP	A	192	40.018	13.417	45.550	1.00	0.00	C
ATOM	2683	HA	ASP	A	192	39.542	13.825	46.329	1.00	0.00	H
ATOM	2684	CB	ASP	A	192	39.679	11.901	45.403	1.00	0.00	C
ATOM	2685	HB1	ASP	A	192	38.683	11.811	45.387	1.00	0.00	H
ATOM	2686	HB2	ASP	A	192	40.043	11.428	46.205	1.00	0.00	H
ATOM	2687	CG	ASP	A	192	40.238	11.185	44.139	1.00	0.00	C
ATOM	2688	OD1	ASP	A	192	39.982	10.018	43.948	1.00	0.00	O
ATOM	2689	OD2	ASP	A	192	41.242	11.643	43.592	1.00	0.00	O
ATOM	2690	C	ASP	A	192	41.561	13.582	45.726	1.00	0.00	C
ATOM	2691	O	ASP	A	192	42.131	13.112	46.723	1.00	0.00	O
ATOM	2692	N	ALA	A	193	42.302	14.190	44.820	1.00	0.00	N
ATOM	2693	H	ALA	A	193	41.824	14.500	43.998	1.00	0.00	H
ATOM	2694	CA	ALA	A	193	43.745	14.468	44.870	1.00	0.00	C
ATOM	2695	HA	ALA	A	193	44.163	14.174	45.730	1.00	0.00	H
ATOM	2696	CB	ALA	A	193	44.461	13.614	43.755	1.00	0.00	C
ATOM	2697	HB1	ALA	A	193	45.444	13.794	43.778	1.00	0.00	H

ATOM	2698	HB2	ALA	A	193	44.296	12.642	43.922	1.00	0.00	H
ATOM	2699	HB3	ALA	A	193	44.097	13.865	42.858	1.00	0.00	H
ATOM	2700	C	ALA	A	193	43.832	16.050	44.717	1.00	0.00	C
ATOM	2701	O	ALA	A	193	42.758	16.605	44.502	1.00	0.00	O
ATOM	2702	N	GLY	A	194	45.051	16.653	44.830	1.00	0.00	N
ATOM	2703	H	GLY	A	194	45.789	16.176	45.308	1.00	0.00	H
ATOM	2704	CA	GLY	A	194	45.299	17.954	44.280	1.00	0.00	C
ATOM	2705	HA1	GLY	A	194	44.933	17.929	43.350	1.00	0.00	H
ATOM	2706	HA2	GLY	A	194	46.294	18.048	44.245	1.00	0.00	H
ATOM	2707	C	GLY	A	194	44.732	19.166	44.981	1.00	0.00	C
ATOM	2708	O	GLY	A	194	44.232	18.949	46.090	1.00	0.00	O
ATOM	2709	N	TYR	A	195	44.826	20.368	44.431	1.00	0.00	N
ATOM	2710	H	TYR	A	195	45.267	20.421	43.535	1.00	0.00	H
ATOM	2711	CA	TYR	A	195	44.340	21.647	45.016	1.00	0.00	C
ATOM	2712	HA	TYR	A	195	44.961	22.253	44.518	1.00	0.00	H
ATOM	2713	CB	TYR	A	195	42.865	21.984	44.720	1.00	0.00	C
ATOM	2714	HB1	TYR	A	195	42.680	22.862	45.162	1.00	0.00	H
ATOM	2715	HB2	TYR	A	195	42.317	21.269	45.155	1.00	0.00	H
ATOM	2716	CG	TYR	A	195	42.330	22.112	43.299	1.00	0.00	C
ATOM	2717	CD1	TYR	A	195	41.985	20.949	42.580	1.00	0.00	C
ATOM	2718	HD1	TYR	A	195	42.258	20.061	42.950	1.00	0.00	H
ATOM	2719	CE1	TYR	A	195	41.282	20.980	41.380	1.00	0.00	C
ATOM	2720	HE1	TYR	A	195	41.011	20.132	40.925	1.00	0.00	H
ATOM	2721	CZ	TYR	A	195	40.962	22.224	40.823	1.00	0.00	C
ATOM	2722	OH	TYR	A	195	40.396	22.260	39.589	1.00	0.00	O
ATOM	2723	HH	TYR	A	195	40.227	23.210	39.328	1.00	0.00	H
ATOM	2724	CE2	TYR	A	195	41.254	23.380	41.511	1.00	0.00	C
ATOM	2725	HE2	TYR	A	195	40.964	24.258	41.131	1.00	0.00	H
ATOM	2726	CD2	TYR	A	195	41.922	23.360	42.686	1.00	0.00	C
ATOM	2727	HD2	TYR	A	195	42.139	24.224	43.141	1.00	0.00	H
ATOM	2728	C	TYR	A	195	44.538	21.817	46.532	1.00	0.00	C
ATOM	2729	O	TYR	A	195	43.783	21.412	47.381	1.00	0.00	O
ATOM	2730	N	GLU	A	196	45.706	22.336	46.991	1.00	0.00	N
ATOM	2731	H	GLU	A	196	46.365	22.618	46.294	1.00	0.00	H
ATOM	2732	CA	GLU	A	196	46.112	22.530	48.367	1.00	0.00	C
ATOM	2733	HA	GLU	A	196	45.932	21.634	48.774	1.00	0.00	H
ATOM	2734	CB	GLU	A	196	47.619	22.688	48.552	1.00	0.00	C
ATOM	2735	HB1	GLU	A	196	47.866	23.605	48.238	1.00	0.00	H
ATOM	2736	HB2	GLU	A	196	48.066	22.006	47.974	1.00	0.00	H
ATOM	2737	CG	GLU	A	196	48.157	22.519	49.984	1.00	0.00	C
ATOM	2738	HG1	GLU	A	196	47.506	22.904	50.638	1.00	0.00	H
ATOM	2739	HG2	GLU	A	196	49.036	22.988	50.073	1.00	0.00	H
ATOM	2740	CD	GLU	A	196	48.350	21.058	50.290	1.00	0.00	C
ATOM	2741	OE1	GLU	A	196	47.802	20.163	49.583	1.00	0.00	O
ATOM	2742	OE2	GLU	A	196	48.832	20.705	51.376	1.00	0.00	O
ATOM	2743	C	GLU	A	196	45.198	23.498	49.135	1.00	0.00	C
ATOM	2744	O	GLU	A	196	45.665	24.571	49.584	1.00	0.00	O
ATOM	2745	N	VAL	A	197	43.929	23.199	49.363	1.00	0.00	N
ATOM	2746	H	VAL	A	197	43.605	22.336	48.976	1.00	0.00	H
ATOM	2747	CA	VAL	A	197	42.932	23.986	50.121	1.00	0.00	C
ATOM	2748	HA	VAL	A	197	42.899	24.779	49.513	1.00	0.00	H
ATOM	2749	CB	VAL	A	197	41.532	23.403	50.131	1.00	0.00	C
ATOM	2750	HB	VAL	A	197	41.735	22.529	50.572	1.00	0.00	H
ATOM	2751	CG1	VAL	A	197	40.498	24.283	50.938	1.00	0.00	C

ATOM	2752	HG11	VAL	A	197	39.597	23.850	50.909	1.00	0.00	H
ATOM	2753	HG12	VAL	A	197	40.799	24.363	51.888	1.00	0.00	H
ATOM	2754	HG13	VAL	A	197	40.443	25.193	50.528	1.00	0.00	H
ATOM	2755	CG2	VAL	A	197	40.896	23.100	48.748	1.00	0.00	C
ATOM	2756	HG21	VAL	A	197	39.980	22.722	48.878	1.00	0.00	H
ATOM	2757	HG22	VAL	A	197	40.835	23.945	48.217	1.00	0.00	H
ATOM	2758	HG23	VAL	A	197	41.463	22.438	48.258	1.00	0.00	H
ATOM	2759	C	VAL	A	197	43.368	24.569	51.473	1.00	0.00	C
ATOM	2760	O	VAL	A	197	43.155	25.802	51.666	1.00	0.00	O
ATOM	2761	N	ASP	A	198	44.087	23.814	52.233	1.00	0.00	N
ATOM	2762	H	ASP	A	198	44.174	22.843	52.012	1.00	0.00	H
ATOM	2763	CA	ASP	A	198	44.782	24.344	53.417	1.00	0.00	C
ATOM	2764	HA	ASP	A	198	43.994	24.610	53.972	1.00	0.00	H
ATOM	2765	CB	ASP	A	198	45.743	23.236	54.070	1.00	0.00	C
ATOM	2766	HB1	ASP	A	198	46.140	23.577	54.922	1.00	0.00	H
ATOM	2767	HB2	ASP	A	198	46.476	22.993	53.435	1.00	0.00	H
ATOM	2768	CG	ASP	A	198	44.941	21.975	54.384	1.00	0.00	C
ATOM	2769	OD1	ASP	A	198	45.599	20.971	54.622	1.00	0.00	O
ATOM	2770	OD2	ASP	A	198	43.707	21.987	54.452	1.00	0.00	O
ATOM	2771	C	ASP	A	198	45.526	25.641	53.237	1.00	0.00	C
ATOM	2772	O	ASP	A	198	45.635	26.408	54.201	1.00	0.00	O
ATOM	2773	N	LYS	A	199	46.224	25.843	52.052	1.00	0.00	N
ATOM	2774	H	LYS	A	199	46.145	25.145	51.340	1.00	0.00	H
ATOM	2775	CA	LYS	A	199	47.060	26.997	51.777	1.00	0.00	C
ATOM	2776	HA	LYS	A	199	47.222	27.349	52.699	1.00	0.00	H
ATOM	2777	CB	LYS	A	199	48.375	26.582	51.061	1.00	0.00	C
ATOM	2778	HB1	LYS	A	199	48.886	27.386	50.757	1.00	0.00	H
ATOM	2779	HB2	LYS	A	199	48.184	25.992	50.276	1.00	0.00	H
ATOM	2780	CG	LYS	A	199	49.162	25.813	52.142	1.00	0.00	C
ATOM	2781	HG1	LYS	A	199	48.671	24.971	52.364	1.00	0.00	H
ATOM	2782	HG2	LYS	A	199	49.233	26.383	52.960	1.00	0.00	H
ATOM	2783	CD	LYS	A	199	50.561	25.440	51.707	1.00	0.00	C
ATOM	2784	HD1	LYS	A	199	50.497	24.977	50.823	1.00	0.00	H
ATOM	2785	HD2	LYS	A	199	50.938	24.811	52.386	1.00	0.00	H
ATOM	2786	CE	LYS	A	199	51.504	26.658	51.570	1.00	0.00	C
ATOM	2787	HE1	LYS	A	199	51.231	27.198	50.774	1.00	0.00	H
ATOM	2788	HE2	LYS	A	199	52.443	26.336	51.448	1.00	0.00	H
ATOM	2789	NZ	LYS	A	199	51.508	27.569	52.727	1.00	0.00	N
ATOM	2790	HZ1	LYS	A	199	52.141	28.324	52.555	1.00	0.00	H
ATOM	2791	HZ2	LYS	A	199	50.587	27.932	52.869	1.00	0.00	H
ATOM	2792	HZ3	LYS	A	199	51.799	27.070	53.543	1.00	0.00	H
ATOM	2793	C	LYS	A	199	46.356	28.078	50.938	1.00	0.00	C
ATOM	2794	O	LYS	A	199	46.216	29.207	51.351	1.00	0.00	O
ATOM	2795	N	ILE	A	200	45.728	27.637	49.815	1.00	0.00	N
ATOM	2796	H	ILE	A	200	45.688	26.651	49.652	1.00	0.00	H
ATOM	2797	CA	ILE	A	200	45.112	28.521	48.840	1.00	0.00	C
ATOM	2798	HA	ILE	A	200	45.755	29.252	48.611	1.00	0.00	H
ATOM	2799	CB	ILE	A	200	44.858	27.713	47.564	1.00	0.00	C
ATOM	2800	HB	ILE	A	200	44.499	28.405	46.938	1.00	0.00	H
ATOM	2801	CG2	ILE	A	200	46.198	27.039	47.049	1.00	0.00	C
ATOM	2802	HG21	ILE	A	200	46.010	26.517	46.217	1.00	0.00	H
ATOM	2803	HG22	ILE	A	200	46.874	27.749	46.852	1.00	0.00	H
ATOM	2804	HG23	ILE	A	200	46.555	26.426	47.754	1.00	0.00	H
ATOM	2805	CG1	ILE	A	200	43.743	26.655	47.638	1.00	0.00	C

```
ATOM   2806 HG11 ILE A 200      42.893  27.134  47.857  1.00  0.00           H
ATOM   2807 HG12 ILE A 200      43.975  26.030  48.383  1.00  0.00           H
ATOM   2808  CD  ILE A 200      43.482  25.800  46.380  1.00  0.00           C
ATOM   2809  HD1 ILE A 200      42.738  25.157  46.562  1.00  0.00           H
ATOM   2810  HD2 ILE A 200      43.228  26.396  45.618  1.00  0.00           H
ATOM   2811  HD3 ILE A 200      44.311  25.292  46.144  1.00  0.00           H
ATOM   2812  C   ILE A 200      43.914  29.262  49.439  1.00  0.00           C
ATOM   2813  O   ILE A 200      43.724  30.413  49.123  1.00  0.00           O
ATOM   2814  N   ALA A 201      43.161  28.729  50.337  1.00  0.00           N
ATOM   2815  H   ALA A 201      43.372  27.803  50.651  1.00  0.00           H
ATOM   2816  CA  ALA A 201      41.979  29.440  50.932  1.00  0.00           C
ATOM   2817  HA  ALA A 201      41.524  29.830  50.131  1.00  0.00           H
ATOM   2818  CB  ALA A 201      41.032  28.465  51.639  1.00  0.00           C
ATOM   2819  HB1 ALA A 201      40.259  28.970  52.022  1.00  0.00           H
ATOM   2820  HB2 ALA A 201      40.696  27.790  50.982  1.00  0.00           H
ATOM   2821  HB3 ALA A 201      41.523  27.998  52.375  1.00  0.00           H
ATOM   2822  C   ALA A 201      42.303  30.647  51.804  1.00  0.00           C
ATOM   2823  O   ALA A 201      41.394  31.479  51.980  1.00  0.00           O
ATOM   2824  N   GLN A 202      43.580  30.880  52.203  1.00  0.00           N
ATOM   2825  H   GLN A 202      44.214  30.115  52.095  1.00  0.00           H
ATOM   2826  CA  GLN A 202      44.164  32.130  52.783  1.00  0.00           C
ATOM   2827  HA  GLN A 202      43.557  32.467  53.502  1.00  0.00           H
ATOM   2828  CB  GLN A 202      45.625  31.677  53.163  1.00  0.00           C
ATOM   2829  HB1 GLN A 202      46.157  31.605  52.319  1.00  0.00           H
ATOM   2830  HB2 GLN A 202      45.568  30.779  53.599  1.00  0.00           H
ATOM   2831  CG  GLN A 202      46.422  32.580  54.112  1.00  0.00           C
ATOM   2832  HG1 GLN A 202      45.900  32.697  54.957  1.00  0.00           H
ATOM   2833  HG2 GLN A 202      46.552  33.470  53.674  1.00  0.00           H
ATOM   2834  CD  GLN A 202      47.800  31.989  54.457  1.00  0.00           C
ATOM   2835  OE1 GLN A 202      48.002  30.790  54.888  1.00  0.00           O
ATOM   2836  NE2 GLN A 202      48.769  32.545  54.095  1.00  0.00           N
ATOM   2837 HE21 GLN A 202      48.692  33.392  53.569  1.00  0.00           H
ATOM   2838 HE22 GLN A 202      49.671  32.171  54.313  1.00  0.00           H
ATOM   2839  C   GLN A 202      44.299  33.271  51.785  1.00  0.00           C
ATOM   2840  O   GLN A 202      44.339  34.406  52.246  1.00  0.00           O
ATOM   2841  N   ASN A 203      44.512  32.965  50.518  1.00  0.00           N
ATOM   2842  H   ASN A 203      44.403  32.010  50.244  1.00  0.00           H
ATOM   2843  CA  ASN A 203      44.882  33.892  49.530  1.00  0.00           C
ATOM   2844  HA  ASN A 203      45.012  34.728  50.063  1.00  0.00           H
ATOM   2845  CB  ASN A 203      46.115  33.439  48.854  1.00  0.00           C
ATOM   2846  HB1 ASN A 203      46.155  33.908  47.972  1.00  0.00           H
ATOM   2847  HB2 ASN A 203      46.023  32.455  48.705  1.00  0.00           H
ATOM   2848  CG  ASN A 203      47.418  33.670  49.547  1.00  0.00           C
ATOM   2849  OD1 ASN A 203      47.643  34.673  50.222  1.00  0.00           O
ATOM   2850  ND2 ASN A 203      48.333  32.749  49.384  1.00  0.00           N
ATOM   2851 HD21 ASN A 203      48.139  31.946  48.820  1.00  0.00           H
ATOM   2852 HD22 ASN A 203      49.226  32.848  49.823  1.00  0.00           H
ATOM   2853  C   ASN A 203      43.823  34.007  48.411  1.00  0.00           C
ATOM   2854  O   ASN A 203      43.638  35.081  47.815  1.00  0.00           O
ATOM   2855  N   LEU A 204      43.035  32.849  48.181  1.00  0.00           N
ATOM   2856  H   LEU A 204      43.306  31.958  48.545  1.00  0.00           H
ATOM   2857  CA  LEU A 204      41.809  33.005  47.390  1.00  0.00           C
ATOM   2858  HA  LEU A 204      42.023  33.585  46.604  1.00  0.00           H
ATOM   2859  CB  LEU A 204      41.380  31.617  46.950  1.00  0.00           C
```

```
ATOM   2860  HB1 LEU A 204      40.493  31.709  46.498  1.00  0.00           H
ATOM   2861  HB2 LEU A 204      41.278  31.061  47.775  1.00  0.00           H
ATOM   2862  CG  LEU A 204      42.355  30.888  45.981  1.00  0.00           C
ATOM   2863  HG  LEU A 204      43.250  30.922  46.425  1.00  0.00           H
ATOM   2864  CD1 LEU A 204      41.883  29.439  45.768  1.00  0.00           C
ATOM   2865 HD11 LEU A 204      42.511  28.972  45.145  1.00  0.00           H
ATOM   2866 HD12 LEU A 204      41.868  28.961  46.646  1.00  0.00           H
ATOM   2867 HD13 LEU A 204      40.964  29.442  45.374  1.00  0.00           H
ATOM   2868  CD2 LEU A 204      42.509  31.559  44.583  1.00  0.00           C
ATOM   2869 HD21 LEU A 204      43.150  31.029  44.028  1.00  0.00           H
ATOM   2870 HD22 LEU A 204      41.619  31.589  44.128  1.00  0.00           H
ATOM   2871 HD23 LEU A 204      42.857  32.490  44.696  1.00  0.00           H
ATOM   2872  C   LEU A 204      40.701  33.708  48.179  1.00  0.00           C
ATOM   2873  O   LEU A 204      40.411  33.394  49.321  1.00  0.00           O
ATOM   2874  N   ASP A 205      40.034  34.658  47.556  1.00  0.00           N
ATOM   2875  H   ASP A 205      40.173  34.786  46.574  1.00  0.00           H
ATOM   2876  CA  ASP A 205      39.096  35.531  48.262  1.00  0.00           C
ATOM   2877  HA  ASP A 205      39.430  35.703  49.189  1.00  0.00           H
ATOM   2878  CB  ASP A 205      38.990  36.828  47.504  1.00  0.00           C
ATOM   2879  HB1 ASP A 205      38.078  37.211  47.651  1.00  0.00           H
ATOM   2880  HB2 ASP A 205      39.120  36.641  46.530  1.00  0.00           H
ATOM   2881  CG  ASP A 205      39.989  37.849  47.913  1.00  0.00           C
ATOM   2882  OD1 ASP A 205      40.302  38.023  49.094  1.00  0.00           O
ATOM   2883  OD2 ASP A 205      40.610  38.425  46.990  1.00  0.00           O
ATOM   2884  C   ASP A 205      37.712  34.822  48.192  1.00  0.00           C
ATOM   2885  O   ASP A 205      37.068  34.808  49.234  1.00  0.00           O
ATOM   2886  N   PHE A 206      37.388  34.140  47.082  1.00  0.00           N
ATOM   2887  H   PHE A 206      37.977  34.276  46.286  1.00  0.00           H
ATOM   2888  CA  PHE A 206      36.234  33.194  46.909  1.00  0.00           C
ATOM   2889  HA  PHE A 206      36.157  32.802  47.826  1.00  0.00           H
ATOM   2890  CB  PHE A 206      34.894  33.870  46.585  1.00  0.00           C
ATOM   2891  HB1 PHE A 206      34.556  34.262  47.441  1.00  0.00           H
ATOM   2892  HB2 PHE A 206      34.274  33.147  46.281  1.00  0.00           H
ATOM   2893  CG  PHE A 206      34.760  34.963  45.567  1.00  0.00           C
ATOM   2894  CD1 PHE A 206      34.433  34.619  44.247  1.00  0.00           C
ATOM   2895  HD1 PHE A 206      34.308  33.666  43.970  1.00  0.00           H
ATOM   2896  CE1 PHE A 206      34.288  35.692  43.319  1.00  0.00           C
ATOM   2897  HE1 PHE A 206      34.157  35.482  42.350  1.00  0.00           H
ATOM   2898  CZ  PHE A 206      34.324  37.053  43.734  1.00  0.00           C
ATOM   2899  HZ  PHE A 206      34.227  37.779  43.054  1.00  0.00           H
ATOM   2900  CE2 PHE A 206      34.491  37.383  45.065  1.00  0.00           C
ATOM   2901  HE2 PHE A 206      34.468  38.330  45.386  1.00  0.00           H
ATOM   2902  CD2 PHE A 206      34.701  36.274  45.960  1.00  0.00           C
ATOM   2903  HD2 PHE A 206      34.811  36.475  46.933  1.00  0.00           H
ATOM   2904  C   PHE A 206      36.521  32.087  45.854  1.00  0.00           C
ATOM   2905  O   PHE A 206      37.325  32.203  44.891  1.00  0.00           O
ATOM   2906  N   VAL A 207      35.779  30.963  46.065  1.00  0.00           N
ATOM   2907  H   VAL A 207      35.140  30.986  46.834  1.00  0.00           H
ATOM   2908  CA  VAL A 207      35.821  29.693  45.263  1.00  0.00           C
ATOM   2909  HA  VAL A 207      36.530  29.766  44.561  1.00  0.00           H
ATOM   2910  CB  VAL A 207      36.155  28.542  46.212  1.00  0.00           C
ATOM   2911  HB  VAL A 207      35.404  28.605  46.869  1.00  0.00           H
ATOM   2912  CG1 VAL A 207      36.241  27.227  45.466  1.00  0.00           C
ATOM   2913 HG11 VAL A 207      36.460  26.493  46.109  1.00  0.00           H
```

```
ATOM   2914 HG12 VAL A 207      35.363  27.035  45.027  1.00  0.00           H
ATOM   2915 HG13 VAL A 207      36.956  27.285  44.770  1.00  0.00           H
ATOM   2916  CG2 VAL A 207      37.579  28.607  46.879  1.00  0.00           C
ATOM   2917 HG21 VAL A 207      37.703  27.816  47.478  1.00  0.00           H
ATOM   2918 HG22 VAL A 207      38.281  28.599  46.167  1.00  0.00           H
ATOM   2919 HG23 VAL A 207      37.658  29.447  47.415  1.00  0.00           H
ATOM   2920  C   VAL A 207      34.475  29.465  44.556  1.00  0.00           C
ATOM   2921  O   VAL A 207      33.478  29.051  45.215  1.00  0.00           O
ATOM   2922  N   ASN A 208      34.457  29.580  43.230  1.00  0.00           N
ATOM   2923  H   ASN A 208      35.304  29.842  42.768  1.00  0.00           H
ATOM   2924  CA  ASN A 208      33.270  29.345  42.410  1.00  0.00           C
ATOM   2925  HA  ASN A 208      32.468  29.561  42.967  1.00  0.00           H
ATOM   2926  CB  ASN A 208      33.310  30.298  41.198  1.00  0.00           C
ATOM   2927  HB1 ASN A 208      32.492  30.148  40.642  1.00  0.00           H
ATOM   2928  HB2 ASN A 208      34.124  30.095  40.654  1.00  0.00           H
ATOM   2929  CG  ASN A 208      33.355  31.764  41.584  1.00  0.00           C
ATOM   2930  OD1 ASN A 208      32.660  32.216  42.481  1.00  0.00           O
ATOM   2931  ND2 ASN A 208      34.185  32.531  40.922  1.00  0.00           N
ATOM   2932 HD21 ASN A 208      34.754  32.143  40.197  1.00  0.00           H
ATOM   2933 HD22 ASN A 208      34.249  33.505  41.141  1.00  0.00           H
ATOM   2934  C   ASN A 208      33.162  27.856  42.025  1.00  0.00           C
ATOM   2935  O   ASN A 208      33.614  27.442  40.956  1.00  0.00           O
ATOM   2936  N   LEU A 209      32.561  27.031  42.889  1.00  0.00           N
ATOM   2937  H   LEU A 209      32.324  27.383  43.794  1.00  0.00           H
ATOM   2938  CA  LEU A 209      32.232  25.640  42.578  1.00  0.00           C
ATOM   2939  HA  LEU A 209      33.088  25.154  42.403  1.00  0.00           H
ATOM   2940  CB  LEU A 209      31.540  25.039  43.778  1.00  0.00           C
ATOM   2941  HB1 LEU A 209      30.766  25.634  43.995  1.00  0.00           H
ATOM   2942  HB2 LEU A 209      32.197  25.059  44.532  1.00  0.00           H
ATOM   2943  CG  LEU A 209      31.014  23.564  43.599  1.00  0.00           C
ATOM   2944  HG  LEU A 209      30.410  23.614  42.803  1.00  0.00           H
ATOM   2945  CD1 LEU A 209      32.107  22.549  43.321  1.00  0.00           C
ATOM   2946 HD11 LEU A 209      31.701  21.641  43.220  1.00  0.00           H
ATOM   2947 HD12 LEU A 209      32.585  22.796  42.478  1.00  0.00           H
ATOM   2948 HD13 LEU A 209      32.756  22.541  44.082  1.00  0.00           H
ATOM   2949  CD2 LEU A 209      30.213  23.036  44.776  1.00  0.00           C
ATOM   2950 HD21 LEU A 209      29.917  22.100  44.585  1.00  0.00           H
ATOM   2951 HD22 LEU A 209      30.783  23.044  45.598  1.00  0.00           H
ATOM   2952 HD23 LEU A 209      29.411  23.616  44.920  1.00  0.00           H
ATOM   2953  C   LEU A 209      31.329  25.568  41.326  1.00  0.00           C
ATOM   2954  O   LEU A 209      30.177  26.004  41.339  1.00  0.00           O
ATOM   2955  N   MET A 210      31.830  24.956  40.257  1.00  0.00           N
ATOM   2956  H   MET A 210      32.765  24.611  40.335  1.00  0.00           H
ATOM   2957  CA  MET A 210      31.153  24.736  38.978  1.00  0.00           C
ATOM   2958  HA  MET A 210      30.616  25.558  38.789  1.00  0.00           H
ATOM   2959  CB  MET A 210      32.197  24.544  37.862  1.00  0.00           C
ATOM   2960  HB1 MET A 210      31.722  24.367  37.000  1.00  0.00           H
ATOM   2961  HB2 MET A 210      32.776  23.762  38.090  1.00  0.00           H
ATOM   2962  CG  MET A 210      33.085  25.783  37.691  1.00  0.00           C
ATOM   2963  HG1 MET A 210      33.603  25.840  38.545  1.00  0.00           H
ATOM   2964  HG2 MET A 210      32.442  26.547  37.639  1.00  0.00           H
ATOM   2965  SD  MET A 210      34.172  25.720  36.248  1.00  0.00           S
ATOM   2966  CE  MET A 210      35.497  24.602  36.779  1.00  0.00           C
ATOM   2967  HE1 MET A 210      36.162  24.498  36.039  1.00  0.00           H
```

ATOM	2968	HE2	MET	A	210	35.951	24.982	37.585	1.00	0.00	H
ATOM	2969	HE3	MET	A	210	35.109	23.709	37.006	1.00	0.00	H
ATOM	2970	C	MET	A	210	30.188	23.550	39.081	1.00	0.00	C
ATOM	2971	O	MET	A	210	30.439	22.464	38.559	1.00	0.00	O
ATOM	2972	N	ALA	A	211	29.086	23.737	39.811	1.00	0.00	N
ATOM	2973	H	ALA	A	211	28.947	24.636	40.226	1.00	0.00	H
ATOM	2974	CA	ALA	A	211	28.067	22.709	40.044	1.00	0.00	C
ATOM	2975	HA	ALA	A	211	28.552	21.836	40.099	1.00	0.00	H
ATOM	2976	CB	ALA	A	211	27.400	22.971	41.398	1.00	0.00	C
ATOM	2977	HB1	ALA	A	211	26.703	22.274	41.566	1.00	0.00	H
ATOM	2978	HB2	ALA	A	211	28.089	22.934	42.122	1.00	0.00	H
ATOM	2979	HB3	ALA	A	211	26.973	23.875	41.390	1.00	0.00	H
ATOM	2980	C	ALA	A	211	27.093	22.539	38.852	1.00	0.00	C
ATOM	2981	O	ALA	A	211	25.880	22.377	39.015	1.00	0.00	O
ATOM	2982	N	TYR	A	212	27.649	22.573	37.644	1.00	0.00	N
ATOM	2983	H	TYR	A	212	28.631	22.757	37.615	1.00	0.00	H
ATOM	2984	CA	TYR	A	212	26.993	22.373	36.356	1.00	0.00	C
ATOM	2985	HA	TYR	A	212	26.070	22.051	36.568	1.00	0.00	H
ATOM	2986	CB	TYR	A	212	26.890	23.701	35.578	1.00	0.00	C
ATOM	2987	HB1	TYR	A	212	25.965	24.055	35.714	1.00	0.00	H
ATOM	2988	HB2	TYR	A	212	27.028	23.491	34.610	1.00	0.00	H
ATOM	2989	CG	TYR	A	212	27.846	24.834	35.920	1.00	0.00	C
ATOM	2990	CD1	TYR	A	212	29.133	24.877	35.350	1.00	0.00	C
ATOM	2991	HD1	TYR	A	212	29.468	24.102	34.814	1.00	0.00	H
ATOM	2992	CE1	TYR	A	212	29.950	26.011	35.533	1.00	0.00	C
ATOM	2993	HE1	TYR	A	212	30.865	26.038	35.130	1.00	0.00	H
ATOM	2994	CZ	TYR	A	212	29.478	27.107	36.284	1.00	0.00	C
ATOM	2995	OH	TYR	A	212	30.249	28.217	36.411	1.00	0.00	O
ATOM	2996	HH	TYR	A	212	29.770	28.894	36.970	1.00	0.00	H
ATOM	2997	CE2	TYR	A	212	28.204	27.047	36.891	1.00	0.00	C
ATOM	2998	HE2	TYR	A	212	27.878	27.812	37.447	1.00	0.00	H
ATOM	2999	CD2	TYR	A	212	27.394	25.909	36.713	1.00	0.00	C
ATOM	3000	HD2	TYR	A	212	26.496	25.863	37.150	1.00	0.00	H
ATOM	3001	C	TYR	A	212	27.727	21.294	35.551	1.00	0.00	C
ATOM	3002	O	TYR	A	212	28.785	20.806	35.950	1.00	0.00	O
ATOM	3003	N	ASP	A	213	27.157	20.911	34.409	1.00	0.00	N
ATOM	3004	H	ASP	A	213	26.275	21.318	34.171	1.00	0.00	H
ATOM	3005	CA	ASP	A	213	27.729	19.938	33.479	1.00	0.00	C
ATOM	3006	HA	ASP	A	213	27.023	19.817	32.781	1.00	0.00	H
ATOM	3007	CB	ASP	A	213	28.991	20.518	32.803	1.00	0.00	C
ATOM	3008	HB1	ASP	A	213	29.166	19.995	31.969	1.00	0.00	H
ATOM	3009	HB2	ASP	A	213	29.760	20.409	33.432	1.00	0.00	H
ATOM	3010	CG	ASP	A	213	28.898	22.004	32.421	1.00	0.00	C
ATOM	3011	OD1	ASP	A	213	29.899	22.725	32.639	1.00	0.00	O
ATOM	3012	OD2	ASP	A	213	27.860	22.422	31.842	1.00	0.00	O
ATOM	3013	C	ASP	A	213	27.936	18.539	34.111	1.00	0.00	C
ATOM	3014	O	ASP	A	213	28.745	17.739	33.637	1.00	0.00	O
ATOM	3015	N	PHE	A	214	27.203	18.208	35.187	1.00	0.00	N
ATOM	3016	H	PHE	A	214	26.556	18.885	35.538	1.00	0.00	H
ATOM	3017	CA	PHE	A	214	27.291	16.911	35.885	1.00	0.00	C
ATOM	3018	HA	PHE	A	214	28.247	16.852	36.173	1.00	0.00	H
ATOM	3019	CB	PHE	A	214	26.333	16.893	37.083	1.00	0.00	C
ATOM	3020	HB1	PHE	A	214	26.130	15.940	37.306	1.00	0.00	H
ATOM	3021	HB2	PHE	A	214	25.490	17.360	36.816	1.00	0.00	H

ATOM	3022	CG	PHE	A	214	26.849	17.560	38.343	1.00	0.00	C
ATOM	3023	CD1	PHE	A	214	26.290	18.767	38.788	1.00	0.00	C
ATOM	3024	HD1	PHE	A	214	25.639	19.257	38.208	1.00	0.00	H
ATOM	3025	CE1	PHE	A	214	26.639	19.291	40.044	1.00	0.00	C
ATOM	3026	HE1	PHE	A	214	26.210	20.133	40.371	1.00	0.00	H
ATOM	3027	CZ	PHE	A	214	27.590	18.632	40.840	1.00	0.00	C
ATOM	3028	HZ	PHE	A	214	27.829	18.997	41.740	1.00	0.00	H
ATOM	3029	CE2	PHE	A	214	28.205	17.459	40.371	1.00	0.00	C
ATOM	3030	HE2	PHE	A	214	28.908	17.007	40.921	1.00	0.00	H
ATOM	3031	CD2	PHE	A	214	27.829	16.922	39.125	1.00	0.00	C
ATOM	3032	HD2	PHE	A	214	28.261	16.083	38.794	1.00	0.00	H
ATOM	3033	C	PHE	A	214	26.981	15.715	34.977	1.00	0.00	C
ATOM	3034	O	PHE	A	214	27.577	14.655	35.142	1.00	0.00	O
ATOM	3035	N	HIS	A	215	26.088	15.909	34.005	1.00	0.00	N
ATOM	3036	H	HIS	A	215	25.618	16.792	34.004	1.00	0.00	H
ATOM	3037	CA	HIS	A	215	25.718	14.975	32.939	1.00	0.00	C
ATOM	3038	HA	HIS	A	215	26.503	14.382	32.761	1.00	0.00	H
ATOM	3039	CB	HIS	A	215	24.525	14.126	33.404	1.00	0.00	C
ATOM	3040	HB1	HIS	A	215	24.238	13.548	32.640	1.00	0.00	H
ATOM	3041	HB2	HIS	A	215	23.780	14.746	33.651	1.00	0.00	H
ATOM	3042	CG	HIS	A	215	24.772	13.219	34.591	1.00	0.00	C
ATOM	3043	ND1	HIS	A	215	25.711	12.207	34.661	1.00	0.00	N
ATOM	3044	HD1	HIS	A	215	26.380	11.978	33.954	1.00	0.00	H
ATOM	3045	CE1	HIS	A	215	25.570	11.583	35.846	1.00	0.00	C
ATOM	3046	HE1	HIS	A	215	26.115	10.806	36.161	1.00	0.00	H
ATOM	3047	NE2	HIS	A	215	24.574	12.176	36.542	1.00	0.00	N
ATOM	3048	CD2	HIS	A	215	24.069	13.213	35.769	1.00	0.00	C
ATOM	3049	HD2	HIS	A	215	23.331	13.838	36.023	1.00	0.00	H
ATOM	3050	C	HIS	A	215	25.359	15.782	31.676	1.00	0.00	C
ATOM	3051	O	HIS	A	215	25.107	16.987	31.760	1.00	0.00	O
ATOM	3052	N	GLY	A	216	25.322	15.141	30.509	1.00	0.00	N
ATOM	3053	H	GLY	A	216	25.498	14.157	30.503	1.00	0.00	H
ATOM	3054	CA	GLY	A	216	25.036	15.804	29.232	1.00	0.00	C
ATOM	3055	HA1	GLY	A	216	25.642	16.596	29.155	1.00	0.00	H
ATOM	3056	HA2	GLY	A	216	24.085	16.113	29.253	1.00	0.00	H
ATOM	3057	C	GLY	A	216	25.248	14.877	28.035	1.00	0.00	C
ATOM	3058	O	GLY	A	216	25.397	13.675	28.205	1.00	0.00	O
ATOM	3059	N	SER	A	217	25.282	15.427	26.820	1.00	0.00	N
ATOM	3060	H	SER	A	217	25.417	16.417	26.771	1.00	0.00	H
ATOM	3061	CA	SER	A	217	25.138	14.701	25.537	1.00	0.00	C
ATOM	3062	HA	SER	A	217	24.243	14.255	25.560	1.00	0.00	H
ATOM	3063	CB	SER	A	217	25.322	15.754	24.454	1.00	0.00	C
ATOM	3064	HB1	SER	A	217	25.149	15.365	23.549	1.00	0.00	H
ATOM	3065	HB2	SER	A	217	26.247	16.133	24.482	1.00	0.00	H
ATOM	3066	OG	SER	A	217	24.411	16.821	24.648	1.00	0.00	O
ATOM	3067	HG	SER	A	217	24.542	17.506	23.931	1.00	0.00	H
ATOM	3068	C	SER	A	217	26.056	13.543	25.305	1.00	0.00	C
ATOM	3069	O	SER	A	217	25.812	12.727	24.420	1.00	0.00	O
ATOM	3070	N	TRP	A	218	27.194	13.509	26.007	1.00	0.00	N
ATOM	3071	H	TRP	A	218	27.345	14.257	26.654	1.00	0.00	H
ATOM	3072	CA	TRP	A	218	28.245	12.486	25.925	1.00	0.00	C
ATOM	3073	HA	TRP	A	218	28.333	12.273	24.952	1.00	0.00	H
ATOM	3074	CB	TRP	A	218	29.516	13.089	26.546	1.00	0.00	C
ATOM	3075	HB1	TRP	A	218	29.788	13.887	26.009	1.00	0.00	H

ATOM	3076	HB2	TRP	A	218	30.244	12.404	26.517	1.00	0.00	H
ATOM	3077	CG	TRP	A	218	29.358	13.530	27.976	1.00	0.00	C
ATOM	3078	CD1	TRP	A	218	29.470	12.725	29.056	1.00	0.00	C
ATOM	3079	HD1	TRP	A	218	29.736	11.761	29.028	1.00	0.00	H
ATOM	3080	NE1	TRP	A	218	29.163	13.429	30.204	1.00	0.00	N
ATOM	3081	HE1	TRP	A	218	29.182	13.041	31.126	1.00	0.00	H
ATOM	3082	CE2	TRP	A	218	28.828	14.733	29.917	1.00	0.00	C
ATOM	3083	CZ2	TRP	A	218	28.435	15.818	30.713	1.00	0.00	C
ATOM	3084	HZ2	TRP	A	218	28.322	15.706	31.700	1.00	0.00	H
ATOM	3085	CH2	TRP	A	218	28.201	17.065	30.108	1.00	0.00	C
ATOM	3086	HH2	TRP	A	218	27.935	17.851	30.666	1.00	0.00	H
ATOM	3087	CZ3	TRP	A	218	28.342	17.205	28.715	1.00	0.00	C
ATOM	3088	HZ3	TRP	A	218	28.178	18.093	28.286	1.00	0.00	H
ATOM	3089	CE3	TRP	A	218	28.713	16.102	27.920	1.00	0.00	C
ATOM	3090	HE3	TRP	A	218	28.794	16.215	26.930	1.00	0.00	H
ATOM	3091	CD2	TRP	A	218	28.972	14.839	28.500	1.00	0.00	C
ATOM	3092	C	TRP	A	218	27.896	11.171	26.645	1.00	0.00	C
ATOM	3093	O	TRP	A	218	28.541	10.151	26.408	1.00	0.00	O
ATOM	3094	N	GLU	A	219	26.941	11.205	27.575	1.00	0.00	N
ATOM	3095	H	GLU	A	219	26.453	12.067	27.715	1.00	0.00	H
ATOM	3096	CA	GLU	A	219	26.562	10.060	28.404	1.00	0.00	C
ATOM	3097	HA	GLU	A	219	27.426	9.691	28.747	1.00	0.00	H
ATOM	3098	CB	GLU	A	219	25.644	10.528	29.537	1.00	0.00	C
ATOM	3099	HB1	GLU	A	219	25.430	9.733	30.105	1.00	0.00	H
ATOM	3100	HB2	GLU	A	219	24.804	10.879	29.124	1.00	0.00	H
ATOM	3101	CG	GLU	A	219	26.204	11.617	30.452	1.00	0.00	C
ATOM	3102	HG1	GLU	A	219	25.446	12.005	30.976	1.00	0.00	H
ATOM	3103	HG2	GLU	A	219	26.611	12.328	29.879	1.00	0.00	H
ATOM	3104	CD	GLU	A	219	27.263	11.145	31.437	1.00	0.00	C
ATOM	3105	OE1	GLU	A	219	27.327	11.810	32.493	1.00	0.00	O
ATOM	3106	OE2	GLU	A	219	28.041	10.208	31.155	1.00	0.00	O
ATOM	3107	C	GLU	A	219	25.813	8.998	27.589	1.00	0.00	C
ATOM	3108	O	GLU	A	219	25.122	9.317	26.612	1.00	0.00	O
ATOM	3109	N	LYS	A	220	25.946	7.737	27.932	1.00	0.00	N
ATOM	3110	H	LYS	A	220	26.641	7.476	28.602	1.00	0.00	H
ATOM	3111	CA	LYS	A	220	25.070	6.692	27.331	1.00	0.00	C
ATOM	3112	HA	LYS	A	220	25.219	6.729	26.343	1.00	0.00	H
ATOM	3113	CB	LYS	A	220	25.456	5.311	27.877	1.00	0.00	C
ATOM	3114	HB1	LYS	A	220	24.735	4.659	27.641	1.00	0.00	H
ATOM	3115	HB2	LYS	A	220	25.538	5.371	28.872	1.00	0.00	H
ATOM	3116	CG	LYS	A	220	26.796	4.814	27.290	1.00	0.00	C
ATOM	3117	HG1	LYS	A	220	27.533	5.422	27.585	1.00	0.00	H
ATOM	3118	HG2	LYS	A	220	26.742	4.816	26.291	1.00	0.00	H
ATOM	3119	CD	LYS	A	220	27.145	3.432	27.723	1.00	0.00	C
ATOM	3120	HD1	LYS	A	220	26.418	2.804	27.446	1.00	0.00	H
ATOM	3121	HD2	LYS	A	220	27.243	3.411	28.718	1.00	0.00	H
ATOM	3122	CE	LYS	A	220	28.466	3.053	27.047	1.00	0.00	C
ATOM	3123	HE1	LYS	A	220	29.168	3.714	27.312	1.00	0.00	H
ATOM	3124	HE2	LYS	A	220	28.342	3.078	26.055	1.00	0.00	H
ATOM	3125	NZ	LYS	A	220	29.024	1.769	27.330	1.00	0.00	N
ATOM	3126	HZ1	LYS	A	220	29.879	1.658	26.824	1.00	0.00	H
ATOM	3127	HZ2	LYS	A	220	29.205	1.693	28.311	1.00	0.00	H
ATOM	3128	HZ3	LYS	A	220	28.379	1.057	27.053	1.00	0.00	H
ATOM	3129	C	LYS	A	220	23.597	6.976	27.587	1.00	0.00	C

ATOM	3130	O	LYS	A	220	22.820	6.630	26.667	1.00	0.00	O
ATOM	3131	N	VAL	A	221	23.269	7.555	28.750	1.00	0.00	N
ATOM	3132	H	VAL	A	221	23.985	7.748	29.421	1.00	0.00	H
ATOM	3133	CA	VAL	A	221	21.873	7.913	29.060	1.00	0.00	C
ATOM	3134	HA	VAL	A	221	21.419	7.836	28.172	1.00	0.00	H
ATOM	3135	CB	VAL	A	221	21.362	6.959	30.204	1.00	0.00	C
ATOM	3136	HB	VAL	A	221	20.428	7.276	30.369	1.00	0.00	H
ATOM	3137	CG1	VAL	A	221	21.454	5.463	29.801	1.00	0.00	C
ATOM	3138	HG11	VAL	A	221	21.121	4.894	30.553	1.00	0.00	H
ATOM	3139	HG12	VAL	A	221	20.895	5.302	28.988	1.00	0.00	H
ATOM	3140	HG13	VAL	A	221	22.406	5.229	29.602	1.00	0.00	H
ATOM	3141	CG2	VAL	A	221	22.154	7.028	31.504	1.00	0.00	C
ATOM	3142	HG21	VAL	A	221	21.760	6.393	32.168	1.00	0.00	H
ATOM	3143	HG22	VAL	A	221	23.106	6.778	31.328	1.00	0.00	H
ATOM	3144	HG23	VAL	A	221	22.115	7.958	31.869	1.00	0.00	H
ATOM	3145	C	VAL	A	221	21.634	9.334	29.488	1.00	0.00	C
ATOM	3146	O	VAL	A	221	22.639	9.986	29.911	1.00	0.00	O
ATOM	3147	N	THR	A	222	20.352	9.788	29.449	1.00	0.00	N
ATOM	3148	H	THR	A	222	19.632	9.166	29.141	1.00	0.00	H
ATOM	3149	CA	THR	A	222	19.973	11.191	29.851	1.00	0.00	C
ATOM	3150	HA	THR	A	222	20.533	11.816	29.307	1.00	0.00	H
ATOM	3151	CB	THR	A	222	18.446	11.406	29.566	1.00	0.00	C
ATOM	3152	HB	THR	A	222	18.231	12.317	29.918	1.00	0.00	H
ATOM	3153	CG2	THR	A	222	18.032	11.269	28.119	1.00	0.00	C
ATOM	3154	HG21	THR	A	222	17.047	11.422	28.038	1.00	0.00	H
ATOM	3155	HG22	THR	A	222	18.521	11.944	27.567	1.00	0.00	H
ATOM	3156	HG23	THR	A	222	18.254	10.350	27.794	1.00	0.00	H
ATOM	3157	OG1	THR	A	222	17.629	10.505	30.244	1.00	0.00	O
ATOM	3158	HG1	THR	A	222	16.672	10.692	30.023	1.00	0.00	H
ATOM	3159	C	THR	A	222	20.281	11.442	31.354	1.00	0.00	C
ATOM	3160	O	THR	A	222	19.933	10.649	32.230	1.00	0.00	O
ATOM	3161	N	GLY	A	223	20.872	12.601	31.674	1.00	0.00	N
ATOM	3162	H	GLY	A	223	21.178	13.193	30.928	1.00	0.00	H
ATOM	3163	CA	GLY	A	223	21.098	13.054	33.047	1.00	0.00	C
ATOM	3164	HA1	GLY	A	223	21.963	12.665	33.364	1.00	0.00	H
ATOM	3165	HA2	GLY	A	223	20.350	12.710	33.614	1.00	0.00	H
ATOM	3166	C	GLY	A	223	21.159	14.541	33.174	1.00	0.00	C
ATOM	3167	O	GLY	A	223	21.490	15.246	32.234	1.00	0.00	O
ATOM	3168	N	HIS	A	224	20.784	15.026	34.315	1.00	0.00	N
ATOM	3169	H	HIS	A	224	20.689	14.396	35.086	1.00	0.00	H
ATOM	3170	CA	HIS	A	224	20.490	16.446	34.544	1.00	0.00	C
ATOM	3171	HA	HIS	A	224	19.926	16.793	33.795	1.00	0.00	H
ATOM	3172	CB	HIS	A	224	19.701	16.579	35.840	1.00	0.00	C
ATOM	3173	HB1	HIS	A	224	20.303	16.446	36.627	1.00	0.00	H
ATOM	3174	HB2	HIS	A	224	18.963	15.904	35.867	1.00	0.00	H
ATOM	3175	CG	HIS	A	224	19.119	17.947	35.890	1.00	0.00	C
ATOM	3176	ND1	HIS	A	224	19.748	19.072	36.434	1.00	0.00	N
ATOM	3177	CE1	HIS	A	224	18.907	20.083	36.335	1.00	0.00	C
ATOM	3178	HE1	HIS	A	224	19.091	21.018	36.638	1.00	0.00	H
ATOM	3179	NE2	HIS	A	224	17.787	19.673	35.776	1.00	0.00	N
ATOM	3180	HE2	HIS	A	224	16.984	20.241	35.594	1.00	0.00	H
ATOM	3181	CD2	HIS	A	224	17.906	18.325	35.483	1.00	0.00	C
ATOM	3182	HD2	HIS	A	224	17.215	17.746	35.050	1.00	0.00	H
ATOM	3183	C	HIS	A	224	21.806	17.228	34.568	1.00	0.00	C

ATOM	3184	O	HIS	A	224	22.836	16.749	35.046	1.00	0.00	O
ATOM	3185	N	ASN	A	225	21.721	18.520	34.111	1.00	0.00	N
ATOM	3186	H	ASN	A	225	20.841	18.843	33.764	1.00	0.00	H
ATOM	3187	CA	ASN	A	225	22.850	19.446	34.106	1.00	0.00	C
ATOM	3188	HA	ASN	A	225	23.528	19.053	33.485	1.00	0.00	H
ATOM	3189	CB	ASN	A	225	22.299	20.823	33.664	1.00	0.00	C
ATOM	3190	HB1	ASN	A	225	21.682	21.162	34.374	1.00	0.00	H
ATOM	3191	HB2	ASN	A	225	21.792	20.704	32.810	1.00	0.00	H
ATOM	3192	CG	ASN	A	225	23.368	21.883	33.429	1.00	0.00	C
ATOM	3193	OD1	ASN	A	225	24.549	21.608	33.296	1.00	0.00	O
ATOM	3194	ND2	ASN	A	225	22.970	23.113	33.205	1.00	0.00	N
ATOM	3195	HD21	ASN	A	225	21.993	23.328	33.193	1.00	0.00	H
ATOM	3196	HD22	ASN	A	225	23.643	23.835	33.047	1.00	0.00	H
ATOM	3197	C	ASN	A	225	23.425	19.635	35.556	1.00	0.00	C
ATOM	3198	O	ASN	A	225	24.616	19.887	35.726	1.00	0.00	O
ATOM	3199	N	SER	A	226	22.568	19.631	36.598	1.00	0.00	N
ATOM	3200	H	SER	A	226	21.722	19.113	36.474	1.00	0.00	H
ATOM	3201	CA	SER	A	226	22.738	20.304	37.899	1.00	0.00	C
ATOM	3202	HA	SER	A	226	23.645	20.037	38.224	1.00	0.00	H
ATOM	3203	CB	SER	A	226	22.698	21.837	37.665	1.00	0.00	C
ATOM	3204	HB1	SER	A	226	21.764	22.156	37.501	1.00	0.00	H
ATOM	3205	HB2	SER	A	226	23.281	22.103	36.897	1.00	0.00	H
ATOM	3206	OG	SER	A	226	23.203	22.461	38.879	1.00	0.00	O
ATOM	3207	HG	SER	A	226	23.195	23.455	38.771	1.00	0.00	H
ATOM	3208	C	SER	A	226	21.729	19.803	38.997	1.00	0.00	C
ATOM	3209	O	SER	A	226	21.076	20.609	39.689	1.00	0.00	O
ATOM	3210	N	PRO	A	227	21.585	18.453	39.228	1.00	0.00	N
ATOM	3211	CD	PRO	A	227	22.349	17.352	38.646	1.00	0.00	C
ATOM	3212	HD1	PRO	A	227	23.323	17.553	38.749	1.00	0.00	H
ATOM	3213	HD2	PRO	A	227	22.122	17.288	37.674	1.00	0.00	H
ATOM	3214	CG	PRO	A	227	21.975	16.043	39.387	1.00	0.00	C
ATOM	3215	HG1	PRO	A	227	22.616	15.829	40.124	1.00	0.00	H
ATOM	3216	HG2	PRO	A	227	21.909	15.262	38.766	1.00	0.00	H
ATOM	3217	CB	PRO	A	227	20.612	16.446	39.928	1.00	0.00	C
ATOM	3218	HB1	PRO	A	227	20.402	15.924	40.755	1.00	0.00	H
ATOM	3219	HB2	PRO	A	227	19.906	16.279	39.240	1.00	0.00	H
ATOM	3220	CA	PRO	A	227	20.648	17.902	40.257	1.00	0.00	C
ATOM	3221	HA	PRO	A	227	19.745	18.328	40.198	1.00	0.00	H
ATOM	3222	C	PRO	A	227	21.102	18.167	41.704	1.00	0.00	C
ATOM	3223	O	PRO	A	227	22.304	18.247	42.046	1.00	0.00	O
ATOM	3224	N	LEU	A	228	20.227	18.529	42.606	1.00	0.00	N
ATOM	3225	H	LEU	A	228	19.268	18.507	42.322	1.00	0.00	H
ATOM	3226	CA	LEU	A	228	20.487	18.974	44.017	1.00	0.00	C
ATOM	3227	HA	LEU	A	228	21.220	19.651	43.953	1.00	0.00	H
ATOM	3228	CB	LEU	A	228	19.304	19.749	44.640	1.00	0.00	C
ATOM	3229	HB1	LEU	A	228	18.516	19.136	44.694	1.00	0.00	H
ATOM	3230	HB2	LEU	A	228	19.084	20.524	44.047	1.00	0.00	H
ATOM	3231	CG	LEU	A	228	19.599	20.287	46.053	1.00	0.00	C
ATOM	3232	HG	LEU	A	228	19.725	19.460	46.601	1.00	0.00	H
ATOM	3233	CD1	LEU	A	228	20.875	21.117	46.183	1.00	0.00	C
ATOM	3234	HD11	LEU	A	228	20.981	21.420	47.130	1.00	0.00	H
ATOM	3235	HD12	LEU	A	228	21.663	20.560	45.921	1.00	0.00	H
ATOM	3236	HD13	LEU	A	228	20.817	21.914	45.582	1.00	0.00	H
ATOM	3237	CD2	LEU	A	228	18.434	21.205	46.535	1.00	0.00	C

ATOM	3238	HD21	LEU	A	228	18.637	21.547	47.452	1.00	0.00	H
ATOM	3239	HD22	LEU	A	228	18.335	21.976	45.906	1.00	0.00	H
ATOM	3240	HD23	LEU	A	228	17.583	20.680	46.556	1.00	0.00	H
ATOM	3241	C	LEU	A	228	20.971	17.759	44.889	1.00	0.00	C
ATOM	3242	O	LEU	A	228	21.797	17.945	45.782	1.00	0.00	O
ATOM	3243	N	TYR	A	229	20.527	16.549	44.485	1.00	0.00	N
ATOM	3244	H	TYR	A	229	20.014	16.506	43.628	1.00	0.00	H
ATOM	3245	CA	TYR	A	229	20.743	15.285	45.216	1.00	0.00	C
ATOM	3246	HA	TYR	A	229	21.594	15.398	45.730	1.00	0.00	H
ATOM	3247	CB	TYR	A	229	19.630	15.069	46.236	1.00	0.00	C
ATOM	3248	HB1	TYR	A	229	19.746	14.158	46.631	1.00	0.00	H
ATOM	3249	HB2	TYR	A	229	18.756	15.113	45.752	1.00	0.00	H
ATOM	3250	CG	TYR	A	229	19.538	16.021	47.375	1.00	0.00	C
ATOM	3251	CD1	TYR	A	229	20.427	16.000	48.481	1.00	0.00	C
ATOM	3252	HD1	TYR	A	229	21.222	15.393	48.471	1.00	0.00	H
ATOM	3253	CE1	TYR	A	229	20.199	16.808	49.559	1.00	0.00	C
ATOM	3254	HE1	TYR	A	229	20.844	16.814	50.323	1.00	0.00	H
ATOM	3255	CZ	TYR	A	229	19.043	17.644	49.597	1.00	0.00	C
ATOM	3256	OH	TYR	A	229	18.785	18.490	50.662	1.00	0.00	O
ATOM	3257	HH	TYR	A	229	17.932	18.985	50.495	1.00	0.00	H
ATOM	3258	CE2	TYR	A	229	18.232	17.772	48.451	1.00	0.00	C
ATOM	3259	HE2	TYR	A	229	17.487	18.439	48.418	1.00	0.00	H
ATOM	3260	CD2	TYR	A	229	18.501	16.939	47.363	1.00	0.00	C
ATOM	3261	HD2	TYR	A	229	17.926	17.008	46.548	1.00	0.00	H
ATOM	3262	C	TYR	A	229	20.865	14.077	44.198	1.00	0.00	C
ATOM	3263	O	TYR	A	229	20.530	14.177	43.058	1.00	0.00	O
ATOM	3264	N	LYS	A	230	21.235	12.939	44.653	1.00	0.00	N
ATOM	3265	H	LYS	A	230	21.402	12.899	45.638	1.00	0.00	H
ATOM	3266	CA	LYS	A	230	21.449	11.669	43.910	1.00	0.00	C
ATOM	3267	HA	LYS	A	230	21.961	11.917	43.088	1.00	0.00	H
ATOM	3268	CB	LYS	A	230	22.236	10.656	44.777	1.00	0.00	C
ATOM	3269	HB1	LYS	A	230	23.036	11.129	45.146	1.00	0.00	H
ATOM	3270	HB2	LYS	A	230	22.535	9.912	44.179	1.00	0.00	H
ATOM	3271	CG	LYS	A	230	21.445	10.032	45.972	1.00	0.00	C
ATOM	3272	HG1	LYS	A	230	20.778	9.366	45.636	1.00	0.00	H
ATOM	3273	HG2	LYS	A	230	20.972	10.748	46.486	1.00	0.00	H
ATOM	3274	CD	LYS	A	230	22.494	9.327	46.880	1.00	0.00	C
ATOM	3275	HD1	LYS	A	230	23.123	10.000	47.268	1.00	0.00	H
ATOM	3276	HD2	LYS	A	230	23.011	8.654	46.352	1.00	0.00	H
ATOM	3277	CE	LYS	A	230	21.751	8.622	48.007	1.00	0.00	C
ATOM	3278	HE1	LYS	A	230	21.004	8.089	47.610	1.00	0.00	H
ATOM	3279	HE2	LYS	A	230	21.376	9.315	48.622	1.00	0.00	H
ATOM	3280	NZ	LYS	A	230	22.576	7.693	48.829	1.00	0.00	N
ATOM	3281	HZ1	LYS	A	230	22.006	7.280	49.539	1.00	0.00	H
ATOM	3282	HZ2	LYS	A	230	22.952	6.975	48.244	1.00	0.00	H
ATOM	3283	HZ3	LYS	A	230	23.324	8.201	49.256	1.00	0.00	H
ATOM	3284	C	LYS	A	230	20.102	11.037	43.542	1.00	0.00	C
ATOM	3285	O	LYS	A	230	19.149	11.183	44.273	1.00	0.00	O
ATOM	3286	N	ARG	A	231	20.062	10.254	42.510	1.00	0.00	N
ATOM	3287	H	ARG	A	231	20.875	10.242	41.928	1.00	0.00	H
ATOM	3288	CA	ARG	A	231	18.899	9.341	42.084	1.00	0.00	C
ATOM	3289	HA	ARG	A	231	18.060	9.781	42.404	1.00	0.00	H
ATOM	3290	CB	ARG	A	231	18.888	9.208	40.557	1.00	0.00	C
ATOM	3291	HB1	ARG	A	231	19.373	8.367	40.318	1.00	0.00	H

ATOM	3292	HB2	ARG	A	231	19.376	9.994	40.177	1.00	0.00	H
ATOM	3293	CG	ARG	A	231	17.460	9.156	39.931	1.00	0.00	C
ATOM	3294	HG1	ARG	A	231	16.881	9.877	40.311	1.00	0.00	H
ATOM	3295	HG2	ARG	A	231	17.034	8.266	40.094	1.00	0.00	H
ATOM	3296	CD	ARG	A	231	17.643	9.375	38.434	1.00	0.00	C
ATOM	3297	HD1	ARG	A	231	18.158	10.216	38.270	1.00	0.00	H
ATOM	3298	HD2	ARG	A	231	16.752	9.437	37.984	1.00	0.00	H
ATOM	3299	NE	ARG	A	231	18.337	8.365	37.774	1.00	0.00	N
ATOM	3300	HE	ARG	A	231	19.327	8.487	37.699	1.00	0.00	H
ATOM	3301	CZ	ARG	A	231	17.844	7.263	37.234	1.00	0.00	C
ATOM	3302	NH1	ARG	A	231	16.611	7.013	37.263	1.00	0.00	N
ATOM	3303	HH11	ARG	A	231	15.981	7.654	37.701	1.00	0.00	H
ATOM	3304	HH12	ARG	A	231	16.261	6.173	36.848	1.00	0.00	H
ATOM	3305	NH2	ARG	A	231	18.616	6.321	36.857	1.00	0.00	N
ATOM	3306	HH21	ARG	A	231	19.606	6.411	36.968	1.00	0.00	H
ATOM	3307	HH22	ARG	A	231	18.232	5.492	36.450	1.00	0.00	H
ATOM	3308	C	ARG	A	231	18.792	8.013	42.786	1.00	0.00	C
ATOM	3309	O	ARG	A	231	19.845	7.557	43.287	1.00	0.00	O
ATOM	3310	N	GLN	A	232	17.635	7.374	43.014	1.00	0.00	N
ATOM	3311	H	GLN	A	232	16.809	7.692	42.548	1.00	0.00	H
ATOM	3312	CA	GLN	A	232	17.536	6.215	43.930	1.00	0.00	C
ATOM	3313	HA	GLN	A	232	17.871	6.630	44.776	1.00	0.00	H
ATOM	3314	CB	GLN	A	232	16.072	5.784	44.077	1.00	0.00	C
ATOM	3315	HB1	GLN	A	232	15.758	5.406	43.206	1.00	0.00	H
ATOM	3316	HB2	GLN	A	232	15.521	6.585	44.310	1.00	0.00	H
ATOM	3317	CG	GLN	A	232	15.875	4.733	45.158	1.00	0.00	C
ATOM	3318	HG1	GLN	A	232	14.948	4.831	45.521	1.00	0.00	H
ATOM	3319	HG2	GLN	A	232	16.538	4.909	45.886	1.00	0.00	H
ATOM	3320	CD	GLN	A	232	16.068	3.277	44.627	1.00	0.00	C
ATOM	3321	OE1	GLN	A	232	16.147	3.014	43.421	1.00	0.00	O
ATOM	3322	NE2	GLN	A	232	15.930	2.303	45.538	1.00	0.00	N
ATOM	3323	HE21	GLN	A	232	15.717	2.533	46.488	1.00	0.00	H
ATOM	3324	HE22	GLN	A	232	16.040	1.346	45.268	1.00	0.00	H
ATOM	3325	C	GLN	A	232	18.499	4.989	43.684	1.00	0.00	C
ATOM	3326	O	GLN	A	232	18.763	4.248	44.600	1.00	0.00	O
ATOM	3327	N	GLU	A	233	18.885	4.756	42.448	1.00	0.00	N
ATOM	3328	H	GLU	A	233	18.479	5.315	41.725	1.00	0.00	H
ATOM	3329	CA	GLU	A	233	19.866	3.738	42.049	1.00	0.00	C
ATOM	3330	HA	GLU	A	233	19.764	2.934	42.634	1.00	0.00	H
ATOM	3331	CB	GLU	A	233	19.570	3.385	40.534	1.00	0.00	C
ATOM	3332	HB1	GLU	A	233	18.583	3.437	40.382	1.00	0.00	H
ATOM	3333	HB2	GLU	A	233	19.885	2.453	40.357	1.00	0.00	H
ATOM	3334	CG	GLU	A	233	20.251	4.312	39.497	1.00	0.00	C
ATOM	3335	HG1	GLU	A	233	21.215	4.413	39.743	1.00	0.00	H
ATOM	3336	HG2	GLU	A	233	19.804	5.206	39.525	1.00	0.00	H
ATOM	3337	CD	GLU	A	233	20.183	3.797	38.064	1.00	0.00	C
ATOM	3338	OE1	GLU	A	233	21.056	4.039	37.226	1.00	0.00	O
ATOM	3339	OE2	GLU	A	233	19.168	3.143	37.813	1.00	0.00	O
ATOM	3340	C	GLU	A	233	21.289	4.225	42.327	1.00	0.00	C
ATOM	3341	O	GLU	A	233	22.188	3.353	42.382	1.00	0.00	O
ATOM	3342	N	GLU	A	234	21.597	5.513	42.289	1.00	0.00	N
ATOM	3343	H	GLU	A	234	20.901	6.227	42.370	1.00	0.00	H
ATOM	3344	CA	GLU	A	234	23.076	5.842	42.115	1.00	0.00	C
ATOM	3345	HA	GLU	A	234	23.342	5.338	41.293	1.00	0.00	H

ATOM	3346	CB	GLU	A	234	23.233	7.385	41.966	1.00	0.00	C
ATOM	3347	HB1	GLU	A	234	24.195	7.635	41.858	1.00	0.00	H
ATOM	3348	HB2	GLU	A	234	22.852	7.859	42.760	1.00	0.00	H
ATOM	3349	CG	GLU	A	234	22.470	7.782	40.731	1.00	0.00	C
ATOM	3350	HG1	GLU	A	234	21.509	7.551	40.881	1.00	0.00	H
ATOM	3351	HG2	GLU	A	234	22.827	7.244	39.968	1.00	0.00	H
ATOM	3352	CD	GLU	A	234	22.594	9.308	40.396	1.00	0.00	C
ATOM	3353	OE1	GLU	A	234	22.935	9.555	39.217	1.00	0.00	O
ATOM	3354	OE2	GLU	A	234	22.604	10.241	41.226	1.00	0.00	O
ATOM	3355	C	GLU	A	234	24.007	5.370	43.300	1.00	0.00	C
ATOM	3356	O	GLU	A	234	23.625	5.681	44.456	1.00	0.00	O
ATOM	3357	N	SER	A	235	25.170	4.778	42.854	1.00	0.00	N
ATOM	3358	H	SER	A	235	25.377	4.831	41.877	1.00	0.00	H
ATOM	3359	CA	SER	A	235	26.135	4.067	43.730	1.00	0.00	C
ATOM	3360	HA	SER	A	235	26.062	4.422	44.662	1.00	0.00	H
ATOM	3361	CB	SER	A	235	25.750	2.553	43.778	1.00	0.00	C
ATOM	3362	HB1	SER	A	235	26.071	2.071	42.963	1.00	0.00	H
ATOM	3363	HB2	SER	A	235	24.761	2.433	43.869	1.00	0.00	H
ATOM	3364	OG	SER	A	235	26.314	1.895	44.856	1.00	0.00	O
ATOM	3365	HG	SER	A	235	26.038	0.934	44.841	1.00	0.00	H
ATOM	3366	C	SER	A	235	27.584	4.387	43.288	1.00	0.00	C
ATOM	3367	O	SER	A	235	27.839	4.808	42.166	1.00	0.00	O
ATOM	3368	N	GLY	A	236	28.575	4.146	44.220	1.00	0.00	N
ATOM	3369	H	GLY	A	236	28.318	3.718	45.086	1.00	0.00	H
ATOM	3370	CA	GLY	A	236	29.980	4.485	44.005	1.00	0.00	C
ATOM	3371	HA1	GLY	A	236	30.365	3.793	43.395	1.00	0.00	H
ATOM	3372	HA2	GLY	A	236	30.438	4.439	44.893	1.00	0.00	H
ATOM	3373	C	GLY	A	236	30.220	5.844	43.407	1.00	0.00	C
ATOM	3374	O	GLY	A	236	29.901	6.900	43.989	1.00	0.00	O
ATOM	3375	N	ALA	A	237	30.937	5.917	42.293	1.00	0.00	N
ATOM	3376	H	ALA	A	237	31.262	5.064	41.884	1.00	0.00	H
ATOM	3377	CA	ALA	A	237	31.279	7.174	41.630	1.00	0.00	C
ATOM	3378	HA	ALA	A	237	31.684	7.755	42.336	1.00	0.00	H
ATOM	3379	CB	ALA	A	237	32.288	6.875	40.515	1.00	0.00	C
ATOM	3380	HB1	ALA	A	237	32.533	7.726	40.050	1.00	0.00	H
ATOM	3381	HB2	ALA	A	237	33.109	6.463	40.910	1.00	0.00	H
ATOM	3382	HB3	ALA	A	237	31.880	6.242	39.857	1.00	0.00	H
ATOM	3383	C	ALA	A	237	30.041	7.912	41.087	1.00	0.00	C
ATOM	3384	O	ALA	A	237	30.039	9.145	41.063	1.00	0.00	O
ATOM	3385	N	ALA	A	238	28.966	7.189	40.737	1.00	0.00	N
ATOM	3386	H	ALA	A	238	29.000	6.196	40.853	1.00	0.00	H
ATOM	3387	CA	ALA	A	238	27.747	7.786	40.194	1.00	0.00	C
ATOM	3388	HA	ALA	A	238	28.038	8.312	39.395	1.00	0.00	H
ATOM	3389	CB	ALA	A	238	26.769	6.685	39.761	1.00	0.00	C
ATOM	3390	HB1	ALA	A	238	25.939	7.103	39.391	1.00	0.00	H
ATOM	3391	HB2	ALA	A	238	27.197	6.116	39.059	1.00	0.00	H
ATOM	3392	HB3	ALA	A	238	26.534	6.119	40.551	1.00	0.00	H
ATOM	3393	C	ALA	A	238	27.101	8.752	41.195	1.00	0.00	C
ATOM	3394	O	ALA	A	238	26.808	9.890	40.828	1.00	0.00	O
ATOM	3395	N	ALA	A	239	27.031	8.388	42.489	1.00	0.00	N
ATOM	3396	H	ALA	A	239	27.410	7.511	42.784	1.00	0.00	H
ATOM	3397	CA	ALA	A	239	26.400	9.278	43.464	1.00	0.00	C
ATOM	3398	HA	ALA	A	239	25.590	9.540	42.939	1.00	0.00	H
ATOM	3399	CB	ALA	A	239	26.003	8.579	44.790	1.00	0.00	C

ATOM	3400	HB1	ALA	A	239	25.580	9.245	45.404	1.00	0.00	H
ATOM	3401	HB2	ALA	A	239	25.353	7.843	44.598	1.00	0.00	H
ATOM	3402	HB3	ALA	A	239	26.820	8.198	45.223	1.00	0.00	H
ATOM	3403	C	ALA	A	239	27.228	10.524	43.802	1.00	0.00	C
ATOM	3404	O	ALA	A	239	26.790	11.317	44.631	1.00	0.00	O
ATOM	3405	N	SER	A	240	28.418	10.733	43.220	1.00	0.00	N
ATOM	3406	H	SER	A	240	28.796	10.022	42.626	1.00	0.00	H
ATOM	3407	CA	SER	A	240	29.190	11.973	43.424	1.00	0.00	C
ATOM	3408	HA	SER	A	240	29.035	12.186	44.389	1.00	0.00	H
ATOM	3409	CB	SER	A	240	30.684	11.739	43.169	1.00	0.00	C
ATOM	3410	HB1	SER	A	240	30.989	10.908	43.635	1.00	0.00	H
ATOM	3411	HB2	SER	A	240	31.217	12.519	43.496	1.00	0.00	H
ATOM	3412	OG	SER	A	240	30.997	11.576	41.797	1.00	0.00	O
ATOM	3413	HG	SER	A	240	31.981	11.428	41.695	1.00	0.00	H
ATOM	3414	C	SER	A	240	28.675	13.163	42.598	1.00	0.00	C
ATOM	3415	O	SER	A	240	29.088	14.301	42.817	1.00	0.00	O
ATOM	3416	N	LEU	A	241	27.789	12.909	41.630	1.00	0.00	N
ATOM	3417	H	LEU	A	241	27.296	12.040	41.666	1.00	0.00	H
ATOM	3418	CA	LEU	A	241	27.491	13.817	40.520	1.00	0.00	C
ATOM	3419	HA	LEU	A	241	28.235	14.484	40.487	1.00	0.00	H
ATOM	3420	CB	LEU	A	241	27.461	12.995	39.226	1.00	0.00	C
ATOM	3421	HB1	LEU	A	241	27.187	13.593	38.473	1.00	0.00	H
ATOM	3422	HB2	LEU	A	241	26.790	12.261	39.329	1.00	0.00	H
ATOM	3423	CG	LEU	A	241	28.829	12.374	38.894	1.00	0.00	C
ATOM	3424	HG	LEU	A	241	29.249	12.010	39.726	1.00	0.00	H
ATOM	3425	CD1	LEU	A	241	28.599	11.259	37.891	1.00	0.00	C
ATOM	3426	HD11	LEU	A	241	29.474	10.836	37.656	1.00	0.00	H
ATOM	3427	HD12	LEU	A	241	27.993	10.571	38.290	1.00	0.00	H
ATOM	3428	HD13	LEU	A	241	28.178	11.635	37.065	1.00	0.00	H
ATOM	3429	CD2	LEU	A	241	29.798	13.413	38.319	1.00	0.00	C
ATOM	3430	HD21	LEU	A	241	30.674	12.975	38.116	1.00	0.00	H
ATOM	3431	HD22	LEU	A	241	29.415	13.798	37.479	1.00	0.00	H
ATOM	3432	HD23	LEU	A	241	29.938	14.144	38.987	1.00	0.00	H
ATOM	3433	C	LEU	A	241	26.226	14.653	40.748	1.00	0.00	C
ATOM	3434	O	LEU	A	241	25.292	14.642	39.945	1.00	0.00	O
ATOM	3435	N	ASN	A	242	26.179	15.330	41.893	1.00	0.00	N
ATOM	3436	H	ASN	A	242	26.958	15.251	42.515	1.00	0.00	H
ATOM	3437	CA	ASN	A	242	25.065	16.180	42.301	1.00	0.00	C
ATOM	3438	HA	ASN	A	242	24.754	16.663	41.482	1.00	0.00	H
ATOM	3439	CB	ASN	A	242	23.964	15.286	42.851	1.00	0.00	C
ATOM	3440	HB1	ASN	A	242	23.742	14.607	42.151	1.00	0.00	H
ATOM	3441	HB2	ASN	A	242	23.163	15.859	43.026	1.00	0.00	H
ATOM	3442	CG	ASN	A	242	24.292	14.540	44.134	1.00	0.00	C
ATOM	3443	OD1	ASN	A	242	24.178	15.055	45.262	1.00	0.00	O
ATOM	3444	ND2	ASN	A	242	24.672	13.310	43.973	1.00	0.00	N
ATOM	3445	HD21	ASN	A	242	24.731	12.921	43.054	1.00	0.00	H
ATOM	3446	HD22	ASN	A	242	24.906	12.751	44.768	1.00	0.00	H
ATOM	3447	C	ASN	A	242	25.492	17.241	43.317	1.00	0.00	C
ATOM	3448	O	ASN	A	242	26.561	17.101	43.916	1.00	0.00	O
ATOM	3449	N	VAL	A	243	24.764	18.361	43.394	1.00	0.00	N
ATOM	3450	H	VAL	A	243	23.890	18.416	42.912	1.00	0.00	H
ATOM	3451	CA	VAL	A	243	25.244	19.534	44.195	1.00	0.00	C
ATOM	3452	HA	VAL	A	243	26.083	19.859	43.759	1.00	0.00	H
ATOM	3453	CB	VAL	A	243	24.252	20.719	44.118	1.00	0.00	C

ATOM	3454	HB	VAL	A	243	23.407	20.226	44.328	1.00	0.00	H
ATOM	3455	CG1	VAL	A	243	24.578	21.808	45.097	1.00	0.00	C
ATOM	3456	HG11	VAL	A	243	23.911	22.547	45.008	1.00	0.00	H
ATOM	3457	HG12	VAL	A	243	24.546	21.441	46.027	1.00	0.00	H
ATOM	3458	HG13	VAL	A	243	25.494	22.162	44.909	1.00	0.00	H
ATOM	3459	CG2	VAL	A	243	24.064	21.474	42.781	1.00	0.00	C
ATOM	3460	HG21	VAL	A	243	23.393	22.206	42.903	1.00	0.00	H
ATOM	3461	HG22	VAL	A	243	24.938	21.867	42.494	1.00	0.00	H
ATOM	3462	HG23	VAL	A	243	23.740	20.837	42.081	1.00	0.00	H
ATOM	3463	C	VAL	A	243	25.555	19.073	45.676	1.00	0.00	C
ATOM	3464	O	VAL	A	243	26.592	19.396	46.232	1.00	0.00	O
ATOM	3465	N	ASP	A	244	24.614	18.315	46.312	1.00	0.00	N
ATOM	3466	H	ASP	A	244	23.756	18.112	45.840	1.00	0.00	H
ATOM	3467	CA	ASP	A	244	24.827	17.771	47.711	1.00	0.00	C
ATOM	3468	HA	ASP	A	244	24.776	18.608	48.256	1.00	0.00	H
ATOM	3469	CB	ASP	A	244	23.713	16.930	48.235	1.00	0.00	C
ATOM	3470	HB1	ASP	A	244	23.667	16.112	47.662	1.00	0.00	H
ATOM	3471	HB2	ASP	A	244	22.871	17.458	48.130	1.00	0.00	H
ATOM	3472	CG	ASP	A	244	23.838	16.493	49.705	1.00	0.00	C
ATOM	3473	OD1	ASP	A	244	23.667	15.277	50.054	1.00	0.00	O
ATOM	3474	OD2	ASP	A	244	24.226	17.338	50.545	1.00	0.00	O
ATOM	3475	C	ASP	A	244	26.215	17.060	47.929	1.00	0.00	C
ATOM	3476	O	ASP	A	244	26.978	17.461	48.799	1.00	0.00	O
ATOM	3477	N	ALA	A	245	26.446	16.124	47.071	1.00	0.00	N
ATOM	3478	H	ALA	A	245	25.736	15.954	46.388	1.00	0.00	H
ATOM	3479	CA	ALA	A	245	27.599	15.318	47.001	1.00	0.00	C
ATOM	3480	HA	ALA	A	245	27.547	14.937	47.924	1.00	0.00	H
ATOM	3481	CB	ALA	A	245	27.511	14.102	46.092	1.00	0.00	C
ATOM	3482	HB1	ALA	A	245	28.378	13.604	46.117	1.00	0.00	H
ATOM	3483	HB2	ALA	A	245	26.774	13.504	46.406	1.00	0.00	H
ATOM	3484	HB3	ALA	A	245	27.324	14.399	45.156	1.00	0.00	H
ATOM	3485	C	ALA	A	245	28.906	16.211	46.671	1.00	0.00	C
ATOM	3486	O	ALA	A	245	29.930	15.935	47.259	1.00	0.00	O
ATOM	3487	N	ALA	A	246	28.844	17.169	45.777	1.00	0.00	N
ATOM	3488	H	ALA	A	246	27.982	17.282	45.283	1.00	0.00	H
ATOM	3489	CA	ALA	A	246	29.932	18.082	45.447	1.00	0.00	C
ATOM	3490	HA	ALA	A	246	30.730	17.496	45.307	1.00	0.00	H
ATOM	3491	CB	ALA	A	246	29.548	18.834	44.174	1.00	0.00	C
ATOM	3492	HB1	ALA	A	246	30.281	19.468	43.927	1.00	0.00	H
ATOM	3493	HB2	ALA	A	246	29.408	18.180	43.430	1.00	0.00	H
ATOM	3494	HB3	ALA	A	246	28.703	19.346	44.331	1.00	0.00	H
ATOM	3495	C	ALA	A	246	30.296	19.056	46.585	1.00	0.00	C
ATOM	3496	O	ALA	A	246	31.483	19.221	46.794	1.00	0.00	O
ATOM	3497	N	VAL	A	247	29.311	19.626	47.239	1.00	0.00	N
ATOM	3498	H	VAL	A	247	28.397	19.492	46.855	1.00	0.00	H
ATOM	3499	CA	VAL	A	247	29.382	20.456	48.494	1.00	0.00	C
ATOM	3500	HA	VAL	A	247	29.989	21.246	48.412	1.00	0.00	H
ATOM	3501	CB	VAL	A	247	27.954	21.093	48.797	1.00	0.00	C
ATOM	3502	HB	VAL	A	247	27.235	20.400	48.839	1.00	0.00	H
ATOM	3503	CG1	VAL	A	247	28.031	21.773	50.197	1.00	0.00	C
ATOM	3504	HG11	VAL	A	247	27.146	22.184	50.417	1.00	0.00	H
ATOM	3505	HG12	VAL	A	247	28.261	21.088	50.888	1.00	0.00	H
ATOM	3506	HG13	VAL	A	247	28.735	22.483	50.184	1.00	0.00	H
ATOM	3507	CG2	VAL	A	247	27.490	22.053	47.639	1.00	0.00	C

```
ATOM   3508 HG21 VAL A 247      26.593  22.434  47.861  1.00  0.00           H
ATOM   3509 HG22 VAL A 247      28.152  22.796  47.537  1.00  0.00           H
ATOM   3510 HG23 VAL A 247      27.431  21.539  46.783  1.00  0.00           H
ATOM   3511  C   VAL A 247      30.015  19.522  49.563  1.00  0.00           C
ATOM   3512  O   VAL A 247      31.023  19.860  50.171  1.00  0.00           O
ATOM   3513  N   GLN A 248      29.522  18.320  49.769  1.00  0.00           N
ATOM   3514  H   GLN A 248      28.723  18.043  49.235  1.00  0.00           H
ATOM   3515  CA  GLN A 248      30.104  17.304  50.790  1.00  0.00           C
ATOM   3516  HA  GLN A 248      30.015  17.627  51.732  1.00  0.00           H
ATOM   3517  CB  GLN A 248      29.384  15.962  50.590  1.00  0.00           C
ATOM   3518  HB1 GLN A 248      29.660  15.564  49.715  1.00  0.00           H
ATOM   3519  HB2 GLN A 248      28.395  16.112  50.592  1.00  0.00           H
ATOM   3520  CG  GLN A 248      29.780  15.008  51.757  1.00  0.00           C
ATOM   3521  HG1 GLN A 248      29.408  15.351  52.620  1.00  0.00           H
ATOM   3522  HG2 GLN A 248      30.776  14.945  51.824  1.00  0.00           H
ATOM   3523  CD  GLN A 248      29.159  13.598  51.408  1.00  0.00           C
ATOM   3524  OE1 GLN A 248      27.888  13.522  51.151  1.00  0.00           O
ATOM   3525  NE2 GLN A 248      29.859  12.576  51.373  1.00  0.00           N
ATOM   3526 HE21 GLN A 248      30.838  12.634  51.568  1.00  0.00           H
ATOM   3527 HE22 GLN A 248      29.445  11.694  51.150  1.00  0.00           H
ATOM   3528  C   GLN A 248      31.547  17.117  50.515  1.00  0.00           C
ATOM   3529  O   GLN A 248      32.381  17.084  51.398  1.00  0.00           O
ATOM   3530  N   GLN A 249      31.877  16.948  49.234  1.00  0.00           N
ATOM   3531  H   GLN A 249      31.154  17.001  48.545  1.00  0.00           H
ATOM   3532  CA  GLN A 249      33.283  16.682  48.776  1.00  0.00           C
ATOM   3533  HA  GLN A 249      33.620  15.909  49.314  1.00  0.00           H
ATOM   3534  CB  GLN A 249      33.312  16.341  47.259  1.00  0.00           C
ATOM   3535  HB1 GLN A 249      32.952  17.133  46.765  1.00  0.00           H
ATOM   3536  HB2 GLN A 249      32.710  15.556  47.115  1.00  0.00           H
ATOM   3537  CG  GLN A 249      34.676  15.991  46.626  1.00  0.00           C
ATOM   3538  HG1 GLN A 249      35.267  16.792  46.723  1.00  0.00           H
ATOM   3539  HG2 GLN A 249      34.519  15.808  45.655  1.00  0.00           H
ATOM   3540  CD  GLN A 249      35.434  14.779  47.212  1.00  0.00           C
ATOM   3541  OE1 GLN A 249      36.683  14.761  47.069  1.00  0.00           O
ATOM   3542  NE2 GLN A 249      34.907  13.962  47.951  1.00  0.00           N
ATOM   3543 HE21 GLN A 249      33.944  14.062  48.201  1.00  0.00           H
ATOM   3544 HE22 GLN A 249      35.439  13.193  48.306  1.00  0.00           H
ATOM   3545  C   GLN A 249      34.194  17.825  49.092  1.00  0.00           C
ATOM   3546  O   GLN A 249      35.263  17.709  49.660  1.00  0.00           O
ATOM   3547  N   TRP A 250      33.841  18.970  48.623  1.00  0.00           N
ATOM   3548  H   TRP A 250      32.993  19.016  48.095  1.00  0.00           H
ATOM   3549  CA  TRP A 250      34.620  20.251  48.816  1.00  0.00           C
ATOM   3550  HA  TRP A 250      35.554  19.929  48.660  1.00  0.00           H
ATOM   3551  CB  TRP A 250      34.220  21.379  47.785  1.00  0.00           C
ATOM   3552  HB1 TRP A 250      34.460  22.279  48.149  1.00  0.00           H
ATOM   3553  HB2 TRP A 250      33.238  21.345  47.600  1.00  0.00           H
ATOM   3554  CG  TRP A 250      34.898  21.250  46.494  1.00  0.00           C
ATOM   3555  CD1 TRP A 250      34.330  20.647  45.452  1.00  0.00           C
ATOM   3556  HD1 TRP A 250      33.456  20.162  45.476  1.00  0.00           H
ATOM   3557  NE1 TRP A 250      35.108  20.786  44.357  1.00  0.00           N
ATOM   3558  HE1 TRP A 250      34.832  20.510  43.436  1.00  0.00           H
ATOM   3559  CE2 TRP A 250      36.374  21.374  44.669  1.00  0.00           C
ATOM   3560  CZ2 TRP A 250      37.531  21.641  43.894  1.00  0.00           C
ATOM   3561  HZ2 TRP A 250      37.566  21.435  42.916  1.00  0.00           H
```

ATOM	3562	CH2	TRP	A	250	38.603	22.198	44.554	1.00	0.00	C
ATOM	3563	HH2	TRP	A	250	39.457	22.352	44.056	1.00	0.00	H
ATOM	3564	CZ3	TRP	A	250	38.525	22.557	45.919	1.00	0.00	C
ATOM	3565	HZ3	TRP	A	250	39.286	23.033	46.359	1.00	0.00	H
ATOM	3566	CE3	TRP	A	250	37.355	22.236	46.661	1.00	0.00	C
ATOM	3567	HE3	TRP	A	250	37.310	22.480	47.630	1.00	0.00	H
ATOM	3568	CD2	TRP	A	250	36.280	21.596	46.076	1.00	0.00	C
ATOM	3569	C	TRP	A	250	34.672	20.793	50.228	1.00	0.00	C
ATOM	3570	O	TRP	A	250	35.590	21.456	50.664	1.00	0.00	O
ATOM	3571	N	LEU	A	251	33.772	20.390	51.092	1.00	0.00	N
ATOM	3572	H	LEU	A	251	32.988	19.868	50.756	1.00	0.00	H
ATOM	3573	CA	LEU	A	251	33.864	20.672	52.540	1.00	0.00	C
ATOM	3574	HA	LEU	A	251	34.460	21.471	52.626	1.00	0.00	H
ATOM	3575	CB	LEU	A	251	32.506	21.044	53.099	1.00	0.00	C
ATOM	3576	HB1	LEU	A	251	32.631	21.121	54.088	1.00	0.00	H
ATOM	3577	HB2	LEU	A	251	31.908	20.268	52.898	1.00	0.00	H
ATOM	3578	CG	LEU	A	251	31.770	22.336	52.621	1.00	0.00	C
ATOM	3579	HG	LEU	A	251	31.639	22.201	51.639	1.00	0.00	H
ATOM	3580	CD1	LEU	A	251	30.484	22.530	53.440	1.00	0.00	C
ATOM	3581	HD11	LEU	A	251	30.015	23.358	53.132	1.00	0.00	H
ATOM	3582	HD12	LEU	A	251	29.885	21.740	53.312	1.00	0.00	H
ATOM	3583	HD13	LEU	A	251	30.716	22.619	54.409	1.00	0.00	H
ATOM	3584	CD2	LEU	A	251	32.511	23.631	52.755	1.00	0.00	C
ATOM	3585	HD21	LEU	A	251	31.939	24.379	52.418	1.00	0.00	H
ATOM	3586	HD22	LEU	A	251	32.734	23.789	53.717	1.00	0.00	H
ATOM	3587	HD23	LEU	A	251	33.355	23.589	52.220	1.00	0.00	H
ATOM	3588	C	LEU	A	251	34.597	19.621	53.325	1.00	0.00	C
ATOM	3589	O	LEU	A	251	35.383	19.984	54.198	1.00	0.00	O
ATOM	3590	N	GLN	A	252	34.533	18.380	52.971	1.00	0.00	N
ATOM	3591	H	GLN	A	252	33.872	18.113	52.270	1.00	0.00	H
ATOM	3592	CA	GLN	A	252	35.370	17.377	53.543	1.00	0.00	C
ATOM	3593	HA	GLN	A	252	35.394	17.664	54.501	1.00	0.00	H
ATOM	3594	CB	GLN	A	252	34.730	15.960	53.375	1.00	0.00	C
ATOM	3595	HB1	GLN	A	252	35.390	15.278	53.689	1.00	0.00	H
ATOM	3596	HB2	GLN	A	252	34.537	15.815	52.404	1.00	0.00	H
ATOM	3597	CG	GLN	A	252	33.404	15.757	54.170	1.00	0.00	C
ATOM	3598	HG1	GLN	A	252	32.933	14.954	53.805	1.00	0.00	H
ATOM	3599	HG2	GLN	A	252	32.830	16.566	54.044	1.00	0.00	H
ATOM	3600	CD	GLN	A	252	33.648	15.545	55.704	1.00	0.00	C
ATOM	3601	OE1	GLN	A	252	34.685	15.822	56.308	1.00	0.00	O
ATOM	3602	NE2	GLN	A	252	32.657	15.080	56.423	1.00	0.00	N
ATOM	3603	HE21	GLN	A	252	31.780	14.872	55.990	1.00	0.00	H
ATOM	3604	HE22	GLN	A	252	32.778	14.933	57.405	1.00	0.00	H
ATOM	3605	C	GLN	A	252	36.816	17.420	53.047	1.00	0.00	C
ATOM	3606	O	GLN	A	252	37.747	16.921	53.734	1.00	0.00	O
ATOM	3607	N	LYS	A	253	37.069	18.124	51.970	1.00	0.00	N
ATOM	3608	H	LYS	A	253	36.297	18.441	51.420	1.00	0.00	H
ATOM	3609	CA	LYS	A	253	38.448	18.477	51.522	1.00	0.00	C
ATOM	3610	HA	LYS	A	253	39.067	17.757	51.835	1.00	0.00	H
ATOM	3611	CB	LYS	A	253	38.468	18.559	49.963	1.00	0.00	C
ATOM	3612	HB1	LYS	A	253	37.908	19.338	49.679	1.00	0.00	H
ATOM	3613	HB2	LYS	A	253	38.076	17.715	49.596	1.00	0.00	H
ATOM	3614	CG	LYS	A	253	39.891	18.737	49.365	1.00	0.00	C
ATOM	3615	HG1	LYS	A	253	40.452	17.934	49.568	1.00	0.00	H

ATOM	3616	HG2	LYS	A	253	40.325	19.553	49.748	1.00	0.00	H
ATOM	3617	CD	LYS	A	253	39.797	18.895	47.873	1.00	0.00	C
ATOM	3618	HD1	LYS	A	253	39.237	19.697	47.668	1.00	0.00	H
ATOM	3619	HD2	LYS	A	253	39.364	18.079	47.489	1.00	0.00	H
ATOM	3620	CE	LYS	A	253	41.142	19.072	47.235	1.00	0.00	C
ATOM	3621	HE1	LYS	A	253	41.719	19.627	47.834	1.00	0.00	H
ATOM	3622	HE2	LYS	A	253	41.030	19.537	46.357	1.00	0.00	H
ATOM	3623	NZ	LYS	A	253	41.838	17.828	46.986	1.00	0.00	N
ATOM	3624	HZ1	LYS	A	253	42.723	18.020	46.562	1.00	0.00	H
ATOM	3625	HZ2	LYS	A	253	41.981	17.347	47.851	1.00	0.00	H
ATOM	3626	HZ3	LYS	A	253	41.291	17.257	46.373	1.00	0.00	H
ATOM	3627	C	LYS	A	253	38.899	19.804	52.174	1.00	0.00	C
ATOM	3628	O	LYS	A	253	40.042	20.254	52.060	1.00	0.00	O
ATOM	3629	N	GLY	A	254	37.949	20.432	52.936	1.00	0.00	N
ATOM	3630	H	GLY	A	254	36.981	20.232	52.786	1.00	0.00	H
ATOM	3631	CA	GLY	A	254	38.342	21.393	53.967	1.00	0.00	C
ATOM	3632	HA1	GLY	A	254	39.314	21.240	54.144	1.00	0.00	H
ATOM	3633	HA2	GLY	A	254	37.814	21.165	54.785	1.00	0.00	H
ATOM	3634	C	GLY	A	254	38.119	22.883	53.620	1.00	0.00	C
ATOM	3635	O	GLY	A	254	38.516	23.739	54.433	1.00	0.00	O
ATOM	3636	N	THR	A	255	37.479	23.168	52.483	1.00	0.00	N
ATOM	3637	H	THR	A	255	37.254	22.411	51.869	1.00	0.00	H
ATOM	3638	CA	THR	A	255	37.099	24.477	52.092	1.00	0.00	C
ATOM	3639	HA	THR	A	255	38.010	24.859	51.935	1.00	0.00	H
ATOM	3640	CB	THR	A	255	36.404	24.561	50.770	1.00	0.00	C
ATOM	3641	HB	THR	A	255	35.446	24.380	50.994	1.00	0.00	H
ATOM	3642	CG2	THR	A	255	36.630	25.881	50.044	1.00	0.00	C
ATOM	3643	HG21	THR	A	255	36.140	25.870	49.172	1.00	0.00	H
ATOM	3644	HG22	THR	A	255	36.290	26.634	50.608	1.00	0.00	H
ATOM	3645	HG23	THR	A	255	37.608	26.006	49.875	1.00	0.00	H
ATOM	3646	OG1	THR	A	255	36.814	23.577	49.863	1.00	0.00	O
ATOM	3647	HG1	THR	A	255	36.315	23.687	49.004	1.00	0.00	H
ATOM	3648	C	THR	A	255	36.298	25.234	53.216	1.00	0.00	C
ATOM	3649	O	THR	A	255	35.283	24.720	53.599	1.00	0.00	O
ATOM	3650	N	PRO	A	256	36.533	26.470	53.663	1.00	0.00	N
ATOM	3651	CD	PRO	A	256	37.827	27.237	53.544	1.00	0.00	C
ATOM	3652	HD1	PRO	A	256	37.871	27.761	52.693	1.00	0.00	H
ATOM	3653	HD2	PRO	A	256	38.622	26.634	53.606	1.00	0.00	H
ATOM	3654	CG	PRO	A	256	37.790	28.130	54.677	1.00	0.00	C
ATOM	3655	HG1	PRO	A	256	38.423	28.891	54.539	1.00	0.00	H
ATOM	3656	HG2	PRO	A	256	38.036	27.639	55.513	1.00	0.00	H
ATOM	3657	CB	PRO	A	256	36.364	28.595	54.707	1.00	0.00	C
ATOM	3658	HB1	PRO	A	256	36.174	29.249	53.975	1.00	0.00	H
ATOM	3659	HB2	PRO	A	256	36.128	29.004	55.588	1.00	0.00	H
ATOM	3660	CA	PRO	A	256	35.673	27.227	54.480	1.00	0.00	C
ATOM	3661	HA	PRO	A	256	35.560	26.622	55.268	1.00	0.00	H
ATOM	3662	C	PRO	A	256	34.302	27.578	53.818	1.00	0.00	C
ATOM	3663	O	PRO	A	256	34.187	27.916	52.620	1.00	0.00	O
ATOM	3664	N	ALA	A	257	33.274	27.456	54.600	1.00	0.00	N
ATOM	3665	H	ALA	A	257	33.369	27.345	55.589	1.00	0.00	H
ATOM	3666	CA	ALA	A	257	31.975	27.491	53.948	1.00	0.00	C
ATOM	3667	HA	ALA	A	257	32.042	26.904	53.142	1.00	0.00	H
ATOM	3668	CB	ALA	A	257	30.919	26.923	54.948	1.00	0.00	C
ATOM	3669	HB1	ALA	A	257	30.015	26.937	54.521	1.00	0.00	H

ATOM	3670	HB2	ALA	A	257	31.160	25.983	55.189	1.00	0.00	H
ATOM	3671	HB3	ALA	A	257	30.906	27.486	55.775	1.00	0.00	H
ATOM	3672	C	ALA	A	257	31.639	28.912	53.419	1.00	0.00	C
ATOM	3673	O	ALA	A	257	31.127	29.053	52.294	1.00	0.00	O
ATOM	3674	N	SER	A	258	32.106	29.891	54.208	1.00	0.00	N
ATOM	3675	H	SER	A	258	32.579	29.615	55.045	1.00	0.00	H
ATOM	3676	CA	SER	A	258	31.984	31.327	53.953	1.00	0.00	C
ATOM	3677	HA	SER	A	258	31.011	31.414	53.739	1.00	0.00	H
ATOM	3678	CB	SER	A	258	32.460	32.082	55.204	1.00	0.00	C
ATOM	3679	HB1	SER	A	258	31.967	31.792	56.024	1.00	0.00	H
ATOM	3680	HB2	SER	A	258	32.374	33.072	55.096	1.00	0.00	H
ATOM	3681	OG	SER	A	258	33.812	31.777	55.377	1.00	0.00	O
ATOM	3682	HG	SER	A	258	34.160	32.252	56.185	1.00	0.00	H
ATOM	3683	C	SER	A	258	32.791	31.883	52.756	1.00	0.00	C
ATOM	3684	O	SER	A	258	32.445	33.006	52.298	1.00	0.00	O
ATOM	3685	N	LYS	A	259	33.675	31.067	52.210	1.00	0.00	N
ATOM	3686	H	LYS	A	259	33.893	30.210	52.677	1.00	0.00	H
ATOM	3687	CA	LYS	A	259	34.362	31.379	50.918	1.00	0.00	C
ATOM	3688	HA	LYS	A	259	34.093	32.322	50.720	1.00	0.00	H
ATOM	3689	CB	LYS	A	259	35.877	31.278	51.065	1.00	0.00	C
ATOM	3690	HB1	LYS	A	259	36.290	31.141	50.165	1.00	0.00	H
ATOM	3691	HB2	LYS	A	259	36.103	30.504	51.657	1.00	0.00	H
ATOM	3692	CG	LYS	A	259	36.402	32.590	51.691	1.00	0.00	C
ATOM	3693	HG1	LYS	A	259	36.013	32.685	52.607	1.00	0.00	H
ATOM	3694	HG2	LYS	A	259	36.105	33.357	51.122	1.00	0.00	H
ATOM	3695	CD	LYS	A	259	37.918	32.656	51.815	1.00	0.00	C
ATOM	3696	HD1	LYS	A	259	38.293	32.368	50.934	1.00	0.00	H
ATOM	3697	HD2	LYS	A	259	38.180	31.997	52.520	1.00	0.00	H
ATOM	3698	CE	LYS	A	259	38.573	34.001	52.176	1.00	0.00	C
ATOM	3699	HE1	LYS	A	259	38.245	34.274	53.080	1.00	0.00	H
ATOM	3700	HE2	LYS	A	259	38.285	34.678	51.499	1.00	0.00	H
ATOM	3701	NZ	LYS	A	259	40.085	34.025	52.221	1.00	0.00	N
ATOM	3702	HZ1	LYS	A	259	40.397	34.944	52.464	1.00	0.00	H
ATOM	3703	HZ2	LYS	A	259	40.409	33.371	52.905	1.00	0.00	H
ATOM	3704	HZ3	LYS	A	259	40.449	33.776	51.324	1.00	0.00	H
ATOM	3705	C	LYS	A	259	33.905	30.491	49.753	1.00	0.00	C
ATOM	3706	O	LYS	A	259	34.193	30.896	48.616	1.00	0.00	O
ATOM	3707	N	LEU	A	260	33.289	29.352	49.953	1.00	0.00	N
ATOM	3708	H	LEU	A	260	33.166	29.044	50.896	1.00	0.00	H
ATOM	3709	CA	LEU	A	260	32.761	28.489	48.862	1.00	0.00	C
ATOM	3710	HA	LEU	A	260	33.365	28.490	48.065	1.00	0.00	H
ATOM	3711	CB	LEU	A	260	32.611	27.022	49.479	1.00	0.00	C
ATOM	3712	HB1	LEU	A	260	31.950	27.067	50.228	1.00	0.00	H
ATOM	3713	HB2	LEU	A	260	33.502	26.744	49.838	1.00	0.00	H
ATOM	3714	CG	LEU	A	260	32.117	25.903	48.440	1.00	0.00	C
ATOM	3715	HG	LEU	A	260	31.342	26.243	47.907	1.00	0.00	H
ATOM	3716	CD1	LEU	A	260	33.116	25.470	47.365	1.00	0.00	C
ATOM	3717	HD11	LEU	A	260	32.697	24.774	46.782	1.00	0.00	H
ATOM	3718	HD12	LEU	A	260	33.371	26.261	46.809	1.00	0.00	H
ATOM	3719	HD13	LEU	A	260	33.932	25.092	47.802	1.00	0.00	H
ATOM	3720	CD2	LEU	A	260	31.874	24.669	49.336	1.00	0.00	C
ATOM	3721	HD21	LEU	A	260	31.558	23.905	48.773	1.00	0.00	H
ATOM	3722	HD22	LEU	A	260	32.727	24.416	49.793	1.00	0.00	H
ATOM	3723	HD23	LEU	A	260	31.180	24.887	50.022	1.00	0.00	H

ATOM	3724	C	LEU	A	260	31.452	29.091	48.382	1.00	0.00	C
ATOM	3725	O	LEU	A	260	30.547	29.491	49.112	1.00	0.00	O
ATOM	3726	N	ILE	A	261	31.326	29.297	47.017	1.00	0.00	N
ATOM	3727	H	ILE	A	261	32.109	28.996	46.473	1.00	0.00	H
ATOM	3728	CA	ILE	A	261	30.194	29.904	46.220	1.00	0.00	C
ATOM	3729	HA	ILE	A	261	29.416	30.211	46.768	1.00	0.00	H
ATOM	3730	CB	ILE	A	261	30.782	31.148	45.502	1.00	0.00	C
ATOM	3731	HB	ILE	A	261	31.397	30.737	44.829	1.00	0.00	H
ATOM	3732	CG2	ILE	A	261	29.757	31.993	44.753	1.00	0.00	C
ATOM	3733	HG21	ILE	A	261	30.216	32.770	44.322	1.00	0.00	H
ATOM	3734	HG22	ILE	A	261	29.315	31.435	44.051	1.00	0.00	H
ATOM	3735	HG23	ILE	A	261	29.068	32.328	45.395	1.00	0.00	H
ATOM	3736	CG1	ILE	A	261	31.599	32.079	46.405	1.00	0.00	C
ATOM	3737	HG11	ILE	A	261	32.061	32.743	45.816	1.00	0.00	H
ATOM	3738	HG12	ILE	A	261	32.281	31.520	46.877	1.00	0.00	H
ATOM	3739	CD	ILE	A	261	30.805	32.862	47.473	1.00	0.00	C
ATOM	3740	HD1	ILE	A	261	31.432	33.434	48.002	1.00	0.00	H
ATOM	3741	HD2	ILE	A	261	30.124	33.441	47.024	1.00	0.00	H
ATOM	3742	HD3	ILE	A	261	30.345	32.218	48.085	1.00	0.00	H
ATOM	3743	C	ILE	A	261	29.675	28.793	45.296	1.00	0.00	C
ATOM	3744	O	ILE	A	261	30.463	28.042	44.701	1.00	0.00	O
ATOM	3745	N	LEU	A	262	28.348	28.712	45.133	1.00	0.00	N
ATOM	3746	H	LEU	A	262	27.784	29.417	45.563	1.00	0.00	H
ATOM	3747	CA	LEU	A	262	27.664	27.665	44.370	1.00	0.00	C
ATOM	3748	HA	LEU	A	262	28.354	26.967	44.179	1.00	0.00	H
ATOM	3749	CB	LEU	A	262	26.515	27.114	45.286	1.00	0.00	C
ATOM	3750	HB1	LEU	A	262	25.964	27.886	45.604	1.00	0.00	H
ATOM	3751	HB2	LEU	A	262	26.928	26.653	46.071	1.00	0.00	H
ATOM	3752	CG	LEU	A	262	25.559	26.128	44.655	1.00	0.00	C
ATOM	3753	HG	LEU	A	262	25.297	26.571	43.797	1.00	0.00	H
ATOM	3754	CD1	LEU	A	262	26.250	24.788	44.473	1.00	0.00	C
ATOM	3755	HD11	LEU	A	262	25.614	24.139	44.055	1.00	0.00	H
ATOM	3756	HD12	LEU	A	262	27.047	24.902	43.880	1.00	0.00	H
ATOM	3757	HD13	LEU	A	262	26.544	24.441	45.364	1.00	0.00	H
ATOM	3758	CD2	LEU	A	262	24.324	25.785	45.557	1.00	0.00	C
ATOM	3759	HD21	LEU	A	262	23.735	25.131	45.082	1.00	0.00	H
ATOM	3760	HD22	LEU	A	262	24.640	25.385	46.417	1.00	0.00	H
ATOM	3761	HD23	LEU	A	262	23.808	26.620	45.748	1.00	0.00	H
ATOM	3762	C	LEU	A	262	27.208	28.101	42.946	1.00	0.00	C
ATOM	3763	O	LEU	A	262	26.269	28.874	42.746	1.00	0.00	O
ATOM	3764	N	GLY	A	263	27.770	27.413	41.925	1.00	0.00	N
ATOM	3765	H	GLY	A	263	28.471	26.732	42.136	1.00	0.00	H
ATOM	3766	CA	GLY	A	263	27.393	27.624	40.510	1.00	0.00	C
ATOM	3767	HA1	GLY	A	263	28.071	27.162	39.938	1.00	0.00	H
ATOM	3768	HA2	GLY	A	263	27.417	28.607	40.327	1.00	0.00	H
ATOM	3769	C	GLY	A	263	26.017	27.093	40.170	1.00	0.00	C
ATOM	3770	O	GLY	A	263	25.732	25.911	40.146	1.00	0.00	O
ATOM	3771	N	MET	A	264	25.135	28.071	39.988	1.00	0.00	N
ATOM	3772	H	MET	A	264	25.493	28.989	40.158	1.00	0.00	H
ATOM	3773	CA	MET	A	264	23.776	28.034	39.595	1.00	0.00	C
ATOM	3774	HA	MET	A	264	23.454	27.113	39.813	1.00	0.00	H
ATOM	3775	CB	MET	A	264	23.023	29.102	40.420	1.00	0.00	C
ATOM	3776	HB1	MET	A	264	22.084	29.192	40.089	1.00	0.00	H
ATOM	3777	HB2	MET	A	264	23.488	29.984	40.346	1.00	0.00	H

ATOM	3778	CG	MET	A	264	22.996	28.681	41.864	1.00	0.00	C
ATOM	3779	HG1	MET	A	264	23.946	28.787	42.158	1.00	0.00	H
ATOM	3780	HG2	MET	A	264	22.767	27.709	41.822	1.00	0.00	H
ATOM	3781	SD	MET	A	264	21.892	29.555	42.932	1.00	0.00	S
ATOM	3782	CE	MET	A	264	20.343	28.727	42.500	1.00	0.00	C
ATOM	3783	HE1	MET	A	264	19.595	29.119	43.035	1.00	0.00	H
ATOM	3784	HE2	MET	A	264	20.158	28.854	41.526	1.00	0.00	H
ATOM	3785	HE3	MET	A	264	20.420	27.750	42.700	1.00	0.00	H
ATOM	3786	C	MET	A	264	23.647	28.296	38.053	1.00	0.00	C
ATOM	3787	O	MET	A	264	23.913	29.383	37.482	1.00	0.00	O
ATOM	3788	N	PRO	A	265	23.370	27.284	37.260	1.00	0.00	N
ATOM	3789	CD	PRO	A	265	23.286	25.993	37.716	1.00	0.00	C
ATOM	3790	HD1	PRO	A	265	22.468	25.929	38.288	1.00	0.00	H
ATOM	3791	HD2	PRO	A	265	24.097	25.818	38.274	1.00	0.00	H
ATOM	3792	CG	PRO	A	265	23.212	25.055	36.502	1.00	0.00	C
ATOM	3793	HG1	PRO	A	265	22.321	24.603	36.460	1.00	0.00	H
ATOM	3794	HG2	PRO	A	265	23.934	24.365	36.547	1.00	0.00	H
ATOM	3795	CB	PRO	A	265	23.411	25.960	35.283	1.00	0.00	C
ATOM	3796	HB1	PRO	A	265	22.744	25.744	34.570	1.00	0.00	H
ATOM	3797	HB2	PRO	A	265	24.336	25.860	34.916	1.00	0.00	H
ATOM	3798	CA	PRO	A	265	23.191	27.378	35.816	1.00	0.00	C
ATOM	3799	HA	PRO	A	265	23.867	28.014	35.444	1.00	0.00	H
ATOM	3800	C	PRO	A	265	21.824	27.980	35.437	1.00	0.00	C
ATOM	3801	O	PRO	A	265	20.788	27.497	35.831	1.00	0.00	O
ATOM	3802	N	THR	A	266	21.839	28.973	34.526	1.00	0.00	N
ATOM	3803	H	THR	A	266	22.724	29.375	34.292	1.00	0.00	H
ATOM	3804	CA	THR	A	266	20.633	29.515	33.843	1.00	0.00	C
ATOM	3805	HA	THR	A	266	19.840	29.421	34.445	1.00	0.00	H
ATOM	3806	CB	THR	A	266	20.742	31.006	33.596	1.00	0.00	C
ATOM	3807	HB	THR	A	266	20.051	31.304	32.938	1.00	0.00	H
ATOM	3808	CG2	THR	A	266	20.585	31.876	34.836	1.00	0.00	C
ATOM	3809	HG21	THR	A	266	20.671	32.839	34.581	1.00	0.00	H
ATOM	3810	HG22	THR	A	266	19.685	31.718	35.242	1.00	0.00	H
ATOM	3811	HG23	THR	A	266	21.296	31.643	35.499	1.00	0.00	H
ATOM	3812	OG1	THR	A	266	21.970	31.213	33.025	1.00	0.00	O
ATOM	3813	HG1	THR	A	266	22.092	32.188	32.841	1.00	0.00	H
ATOM	3814	C	THR	A	266	20.406	28.695	32.582	1.00	0.00	C
ATOM	3815	O	THR	A	266	19.855	29.172	31.555	1.00	0.00	O
ATOM	3816	N	TYR	A	267	20.799	27.415	32.556	1.00	0.00	N
ATOM	3817	H	TYR	A	267	21.203	26.996	33.369	1.00	0.00	H
ATOM	3818	CA	TYR	A	267	20.633	26.638	31.334	1.00	0.00	C
ATOM	3819	HA	TYR	A	267	19.780	27.025	30.983	1.00	0.00	H
ATOM	3820	CB	TYR	A	267	21.843	26.844	30.396	1.00	0.00	C
ATOM	3821	HB1	TYR	A	267	21.956	27.825	30.241	1.00	0.00	H
ATOM	3822	HB2	TYR	A	267	21.647	26.389	29.527	1.00	0.00	H
ATOM	3823	CG	TYR	A	267	23.171	26.299	30.918	1.00	0.00	C
ATOM	3824	CD1	TYR	A	267	23.949	27.078	31.795	1.00	0.00	C
ATOM	3825	HD1	TYR	A	267	23.621	27.977	32.084	1.00	0.00	H
ATOM	3826	CE1	TYR	A	267	25.186	26.599	32.268	1.00	0.00	C
ATOM	3827	HE1	TYR	A	267	25.722	27.156	32.902	1.00	0.00	H
ATOM	3828	CZ	TYR	A	267	25.677	25.343	31.858	1.00	0.00	C
ATOM	3829	OH	TYR	A	267	26.887	24.928	32.319	1.00	0.00	O
ATOM	3830	HH	TYR	A	267	27.096	24.025	31.942	1.00	0.00	H
ATOM	3831	CE2	TYR	A	267	24.896	24.554	30.984	1.00	0.00	C

ATOM	3832	HE2	TYR	A	267	25.226	23.656	30.694	1.00	0.00	H
ATOM	3833	CD2	TYR	A	267	23.650	25.032	30.519	1.00	0.00	C
ATOM	3834	HD2	TYR	A	267	23.102	24.468	29.901	1.00	0.00	H
ATOM	3835	C	TYR	A	267	20.311	25.177	31.484	1.00	0.00	C
ATOM	3836	O	TYR	A	267	20.465	24.589	32.532	1.00	0.00	O
ATOM	3837	N	GLY	A	268	19.850	24.554	30.417	1.00	0.00	N
ATOM	3838	H	GLY	A	268	19.509	25.092	29.646	1.00	0.00	H
ATOM	3839	CA	GLY	A	268	19.825	23.081	30.334	1.00	0.00	C
ATOM	3840	HA1	GLY	A	268	18.915	22.829	30.006	1.00	0.00	H
ATOM	3841	HA2	GLY	A	268	19.949	22.741	31.266	1.00	0.00	H
ATOM	3842	C	GLY	A	268	20.851	22.399	29.444	1.00	0.00	C
ATOM	3843	O	GLY	A	268	21.382	23.043	28.544	1.00	0.00	O
ATOM	3844	N	ARG	A	269	21.029	21.072	29.584	1.00	0.00	N
ATOM	3845	H	ARG	A	269	20.621	20.613	30.373	1.00	0.00	H
ATOM	3846	CA	ARG	A	269	21.799	20.272	28.622	1.00	0.00	C
ATOM	3847	HA	ARG	A	269	22.066	20.888	27.880	1.00	0.00	H
ATOM	3848	CB	ARG	A	269	23.070	19.768	29.307	1.00	0.00	C
ATOM	3849	HB1	ARG	A	269	23.606	19.243	28.646	1.00	0.00	H
ATOM	3850	HB2	ARG	A	269	22.812	19.177	30.072	1.00	0.00	H
ATOM	3851	CG	ARG	A	269	23.923	20.940	29.836	1.00	0.00	C
ATOM	3852	HG1	ARG	A	269	23.578	21.205	30.736	1.00	0.00	H
ATOM	3853	HG2	ARG	A	269	23.834	21.710	29.205	1.00	0.00	H
ATOM	3854	CD	ARG	A	269	25.408	20.604	29.973	1.00	0.00	C
ATOM	3855	HD1	ARG	A	269	25.519	19.678	30.333	1.00	0.00	H
ATOM	3856	HD2	ARG	A	269	25.852	21.256	30.588	1.00	0.00	H
ATOM	3857	NE	ARG	A	269	26.064	20.678	28.653	1.00	0.00	N
ATOM	3858	HE	ARG	A	269	25.632	20.164	27.912	1.00	0.00	H
ATOM	3859	CZ	ARG	A	269	27.161	21.349	28.342	1.00	0.00	C
ATOM	3860	NH1	ARG	A	269	27.637	21.283	27.134	1.00	0.00	N
ATOM	3861	HH11	ARG	A	269	27.174	20.727	26.444	1.00	0.00	H
ATOM	3862	HH12	ARG	A	269	28.466	21.789	26.895	1.00	0.00	H
ATOM	3863	NH2	ARG	A	269	27.837	22.084	29.169	1.00	0.00	N
ATOM	3864	HH21	ARG	A	269	27.537	22.173	30.119	1.00	0.00	H
ATOM	3865	HH22	ARG	A	269	28.658	22.561	28.857	1.00	0.00	H
ATOM	3866	C	ARG	A	269	20.955	19.185	27.928	1.00	0.00	C
ATOM	3867	O	ARG	A	269	20.164	18.511	28.583	1.00	0.00	O
ATOM	3868	N	SER	A	270	21.129	19.042	26.630	1.00	0.00	N
ATOM	3869	H	SER	A	270	21.974	19.394	26.226	1.00	0.00	H
ATOM	3870	CA	SER	A	270	20.116	18.370	25.706	1.00	0.00	C
ATOM	3871	HA	SER	A	270	19.327	18.234	26.305	1.00	0.00	H
ATOM	3872	CB	SER	A	270	19.783	19.273	24.411	1.00	0.00	C
ATOM	3873	HB1	SER	A	270	19.649	20.234	24.654	1.00	0.00	H
ATOM	3874	HB2	SER	A	270	18.977	18.937	23.923	1.00	0.00	H
ATOM	3875	OG	SER	A	270	20.914	19.202	23.498	1.00	0.00	O
ATOM	3876	HG	SER	A	270	20.724	19.757	22.688	1.00	0.00	H
ATOM	3877	C	SER	A	270	20.535	16.984	25.243	1.00	0.00	C
ATOM	3878	O	SER	A	270	21.721	16.619	25.192	1.00	0.00	O
ATOM	3879	N	PHE	A	271	19.591	16.171	24.840	1.00	0.00	N
ATOM	3880	H	PHE	A	271	18.651	16.494	24.952	1.00	0.00	H
ATOM	3881	CA	PHE	A	271	19.757	14.814	24.231	1.00	0.00	C
ATOM	3882	HA	PHE	A	271	20.641	14.938	23.779	1.00	0.00	H
ATOM	3883	CB	PHE	A	271	19.739	13.636	25.277	1.00	0.00	C
ATOM	3884	HB1	PHE	A	271	19.971	12.786	24.805	1.00	0.00	H
ATOM	3885	HB2	PHE	A	271	18.818	13.563	25.661	1.00	0.00	H

ATOM	3886	CG	PHE	A	271	20.713	13.788	26.451	1.00	0.00	C
ATOM	3887	CD1	PHE	A	271	20.299	14.580	27.480	1.00	0.00	C
ATOM	3888	HD1	PHE	A	271	19.487	15.153	27.370	1.00	0.00	H
ATOM	3889	CE1	PHE	A	271	21.010	14.598	28.704	1.00	0.00	C
ATOM	3890	HE1	PHE	A	271	20.676	15.143	29.473	1.00	0.00	H
ATOM	3891	CZ	PHE	A	271	22.177	13.852	28.830	1.00	0.00	C
ATOM	3892	HZ	PHE	A	271	22.770	13.975	29.625	1.00	0.00	H
ATOM	3893	CE2	PHE	A	271	22.517	12.920	27.826	1.00	0.00	C
ATOM	3894	HE2	PHE	A	271	23.243	12.249	27.975	1.00	0.00	H
ATOM	3895	CD2	PHE	A	271	21.827	12.957	26.641	1.00	0.00	C
ATOM	3896	HD2	PHE	A	271	22.128	12.372	25.888	1.00	0.00	H
ATOM	3897	C	PHE	A	271	18.662	14.456	23.230	1.00	0.00	C
ATOM	3898	O	PHE	A	271	17.535	14.817	23.462	1.00	0.00	O
ATOM	3899	N	THR	A	272	18.943	13.720	22.197	1.00	0.00	N
ATOM	3900	H	THR	A	272	19.924	13.640	22.020	1.00	0.00	H
ATOM	3901	CA	THR	A	272	18.126	12.994	21.253	1.00	0.00	C
ATOM	3902	HA	THR	A	272	17.186	13.335	21.230	1.00	0.00	H
ATOM	3903	CB	THR	A	272	18.630	13.239	19.789	1.00	0.00	C
ATOM	3904	HB	THR	A	272	18.522	14.171	19.444	1.00	0.00	H
ATOM	3905	CG2	THR	A	272	20.100	12.963	19.564	1.00	0.00	C
ATOM	3906	HG21	THR	A	272	20.331	13.145	18.608	1.00	0.00	H
ATOM	3907	HG22	THR	A	272	20.645	13.555	20.157	1.00	0.00	H
ATOM	3908	HG23	THR	A	272	20.296	12.006	19.778	1.00	0.00	H
ATOM	3909	OG1	THR	A	272	17.822	12.393	19.056	1.00	0.00	O
ATOM	3910	HG1	THR	A	272	18.057	12.465	18.087	1.00	0.00	H
ATOM	3911	C	THR	A	272	17.966	11.602	21.802	1.00	0.00	C
ATOM	3912	O	THR	A	272	19.007	10.955	22.090	1.00	0.00	O
ATOM	3913	N	LEU	A	273	16.753	11.096	22.058	1.00	0.00	N
ATOM	3914	H	LEU	A	273	15.957	11.668	21.862	1.00	0.00	H
ATOM	3915	CA	LEU	A	273	16.518	9.765	22.604	1.00	0.00	C
ATOM	3916	HA	LEU	A	273	17.250	9.679	23.280	1.00	0.00	H
ATOM	3917	CB	LEU	A	273	15.032	9.768	23.152	1.00	0.00	C
ATOM	3918	HB1	LEU	A	273	14.800	8.839	23.439	1.00	0.00	H
ATOM	3919	HB2	LEU	A	273	14.423	10.050	22.411	1.00	0.00	H
ATOM	3920	CG	LEU	A	273	14.805	10.740	24.380	1.00	0.00	C
ATOM	3921	HG	LEU	A	273	15.245	11.625	24.229	1.00	0.00	H
ATOM	3922	CD1	LEU	A	273	13.255	10.796	24.614	1.00	0.00	C
ATOM	3923	HD11	LEU	A	273	13.056	11.401	25.385	1.00	0.00	H
ATOM	3924	HD12	LEU	A	273	12.806	11.145	23.791	1.00	0.00	H
ATOM	3925	HD13	LEU	A	273	12.915	9.877	24.815	1.00	0.00	H
ATOM	3926	CD2	LEU	A	273	15.339	10.068	25.644	1.00	0.00	C
ATOM	3927	HD21	LEU	A	273	15.201	10.674	26.427	1.00	0.00	H
ATOM	3928	HD22	LEU	A	273	14.850	9.209	25.797	1.00	0.00	H
ATOM	3929	HD23	LEU	A	273	16.315	9.881	25.535	1.00	0.00	H
ATOM	3930	C	LEU	A	273	16.622	8.566	21.543	1.00	0.00	C
ATOM	3931	O	LEU	A	273	16.101	8.623	20.372	1.00	0.00	O
ATOM	3932	N	ALA	A	274	17.246	7.459	21.975	1.00	0.00	N
ATOM	3933	H	ALA	A	274	17.677	7.475	22.877	1.00	0.00	H
ATOM	3934	CA	ALA	A	274	17.328	6.176	21.152	1.00	0.00	C
ATOM	3935	HA	ALA	A	274	17.930	6.324	20.367	1.00	0.00	H
ATOM	3936	CB	ALA	A	274	17.920	5.141	21.963	1.00	0.00	C
ATOM	3937	HB1	ALA	A	274	17.979	4.295	21.433	1.00	0.00	H
ATOM	3938	HB2	ALA	A	274	18.837	5.422	22.245	1.00	0.00	H
ATOM	3939	HB3	ALA	A	274	17.355	4.988	22.773	1.00	0.00	H

ATOM	3940	C	ALA	A	274	15.911	5.705	20.700	1.00	0.00	C
ATOM	3941	O	ALA	A	274	15.745	5.167	19.651	1.00	0.00	O
ATOM	3942	N	SER	A	275	14.872	5.869	21.574	1.00	0.00	N
ATOM	3943	H	SER	A	275	15.105	5.970	22.541	1.00	0.00	H
ATOM	3944	CA	SER	A	275	13.473	5.910	21.231	1.00	0.00	C
ATOM	3945	HA	SER	A	275	13.389	6.217	20.283	1.00	0.00	H
ATOM	3946	CB	SER	A	275	12.885	4.509	21.313	1.00	0.00	C
ATOM	3947	HB1	SER	A	275	13.063	4.092	22.204	1.00	0.00	H
ATOM	3948	HB2	SER	A	275	13.247	3.920	20.590	1.00	0.00	H
ATOM	3949	OG	SER	A	275	11.489	4.613	21.143	1.00	0.00	O
ATOM	3950	HG	SER	A	275	11.078	3.703	21.193	1.00	0.00	H
ATOM	3951	C	SER	A	275	12.780	6.919	22.145	1.00	0.00	C
ATOM	3952	O	SER	A	275	13.157	6.993	23.313	1.00	0.00	O
ATOM	3953	N	SER	A	276	11.816	7.630	21.548	1.00	0.00	N
ATOM	3954	H	SER	A	276	11.678	7.456	20.573	1.00	0.00	H
ATOM	3955	CA	SER	A	276	10.937	8.637	22.159	1.00	0.00	C
ATOM	3956	HA	SER	A	276	11.475	9.215	22.772	1.00	0.00	H
ATOM	3957	CB	SER	A	276	10.321	9.524	21.018	1.00	0.00	C
ATOM	3958	HB1	SER	A	276	9.626	10.153	21.367	1.00	0.00	H
ATOM	3959	HB2	SER	A	276	9.930	8.969	20.284	1.00	0.00	H
ATOM	3960	OG	SER	A	276	11.329	10.300	20.453	1.00	0.00	O
ATOM	3961	HG	SER	A	276	10.944	10.868	19.725	1.00	0.00	H
ATOM	3962	C	SER	A	276	9.924	7.951	23.057	1.00	0.00	C
ATOM	3963	O	SER	A	276	9.180	8.562	23.805	1.00	0.00	O
ATOM	3964	N	SER	A	277	9.843	6.591	22.949	1.00	0.00	N
ATOM	3965	H	SER	A	277	10.381	6.122	22.249	1.00	0.00	H
ATOM	3966	CA	SER	A	277	8.945	5.770	23.878	1.00	0.00	C
ATOM	3967	HA	SER	A	277	8.015	6.117	23.752	1.00	0.00	H
ATOM	3968	CB	SER	A	277	9.186	4.297	23.491	1.00	0.00	C
ATOM	3969	HB1	SER	A	277	8.885	4.103	22.557	1.00	0.00	H
ATOM	3970	HB2	SER	A	277	8.736	3.667	24.124	1.00	0.00	H
ATOM	3971	OG	SER	A	277	10.573	4.081	23.563	1.00	0.00	O
ATOM	3972	HG	SER	A	277	10.776	3.133	23.318	1.00	0.00	H
ATOM	3973	C	SER	A	277	9.214	5.897	25.385	1.00	0.00	C
ATOM	3974	O	SER	A	277	8.375	5.476	26.199	1.00	0.00	O
ATOM	3975	N	ASP	A	278	10.354	6.395	25.859	1.00	0.00	N
ATOM	3976	H	ASP	A	278	11.006	6.732	25.180	1.00	0.00	H
ATOM	3977	CA	ASP	A	278	10.764	6.506	27.264	1.00	0.00	C
ATOM	3978	HA	ASP	A	278	9.937	6.582	27.820	1.00	0.00	H
ATOM	3979	CB	ASP	A	278	11.522	5.254	27.686	1.00	0.00	C
ATOM	3980	HB1	ASP	A	278	12.420	5.231	27.246	1.00	0.00	H
ATOM	3981	HB2	ASP	A	278	11.005	4.437	27.431	1.00	0.00	H
ATOM	3982	CG	ASP	A	278	11.688	5.308	29.190	1.00	0.00	C
ATOM	3983	OD1	ASP	A	278	10.888	5.925	29.956	1.00	0.00	O
ATOM	3984	OD2	ASP	A	278	12.501	4.546	29.710	1.00	0.00	O
ATOM	3985	C	ASP	A	278	11.503	7.769	27.516	1.00	0.00	C
ATOM	3986	O	ASP	A	278	12.707	7.941	27.289	1.00	0.00	O
ATOM	3987	N	THR	A	279	10.769	8.755	28.045	1.00	0.00	N
ATOM	3988	H	THR	A	279	9.835	8.525	28.317	1.00	0.00	H
ATOM	3989	CA	THR	A	279	11.202	10.148	28.266	1.00	0.00	C
ATOM	3990	HA	THR	A	279	11.966	10.282	27.634	1.00	0.00	H
ATOM	3991	CB	THR	A	279	10.142	11.204	27.899	1.00	0.00	C
ATOM	3992	HB	THR	A	279	10.499	12.104	28.151	1.00	0.00	H
ATOM	3993	CG2	THR	A	279	9.712	11.107	26.444	1.00	0.00	C

ATOM	3994	HG21	THR	A	279	9.026	11.809	26.252	1.00	0.00	H
ATOM	3995	HG22	THR	A	279	10.506	11.246	25.852	1.00	0.00	H
ATOM	3996	HG23	THR	A	279	9.321	10.203	26.271	1.00	0.00	H
ATOM	3997	OG1	THR	A	279	8.990	11.040	28.618	1.00	0.00	O
ATOM	3998	HG1	THR	A	279	8.329	11.742	28.353	1.00	0.00	H
ATOM	3999	C	THR	A	279	11.672	10.305	29.700	1.00	0.00	C
ATOM	4000	O	THR	A	279	11.984	11.424	30.038	1.00	0.00	O
ATOM	4001	N	ARG	A	280	11.670	9.235	30.607	1.00	0.00	N
ATOM	4002	H	ARG	A	280	11.302	8.352	30.316	1.00	0.00	H
ATOM	4003	CA	ARG	A	280	12.199	9.378	31.986	1.00	0.00	C
ATOM	4004	HA	ARG	A	280	11.622	10.097	32.374	1.00	0.00	H
ATOM	4005	CB	ARG	A	280	11.999	8.090	32.799	1.00	0.00	C
ATOM	4006	HB1	ARG	A	280	12.370	8.269	33.710	1.00	0.00	H
ATOM	4007	HB2	ARG	A	280	12.545	7.383	32.350	1.00	0.00	H
ATOM	4008	CG	ARG	A	280	10.531	7.533	32.973	1.00	0.00	C
ATOM	4009	HG1	ARG	A	280	10.097	7.482	32.073	1.00	0.00	H
ATOM	4010	HG2	ARG	A	280	10.016	8.161	33.556	1.00	0.00	H
ATOM	4011	CD	ARG	A	280	10.515	6.094	33.631	1.00	0.00	C
ATOM	4012	HD1	ARG	A	280	9.582	5.771	33.793	1.00	0.00	H
ATOM	4013	HD2	ARG	A	280	11.028	6.074	34.489	1.00	0.00	H
ATOM	4014	NE	ARG	A	280	11.161	5.197	32.679	1.00	0.00	N
ATOM	4015	HE	ARG	A	280	11.387	5.577	31.782	1.00	0.00	H
ATOM	4016	CZ	ARG	A	280	11.480	3.914	32.891	1.00	0.00	C
ATOM	4017	NH1	ARG	A	280	11.960	3.287	31.933	1.00	0.00	N
ATOM	4018	HH11	ARG	A	280	12.092	3.743	31.053	1.00	0.00	H
ATOM	4019	HH12	ARG	A	280	12.214	2.326	32.041	1.00	0.00	H
ATOM	4020	NH2	ARG	A	280	11.260	3.251	34.037	1.00	0.00	N
ATOM	4021	HH21	ARG	A	280	10.829	3.717	34.809	1.00	0.00	H
ATOM	4022	HH22	ARG	A	280	11.527	2.291	34.118	1.00	0.00	H
ATOM	4023	C	ARG	A	280	13.711	9.834	32.026	1.00	0.00	C
ATOM	4024	O	ARG	A	280	14.350	9.928	30.991	1.00	0.00	O
ATOM	4025	N	VAL	A	281	14.287	10.223	33.162	1.00	0.00	N
ATOM	4026	H	VAL	A	281	13.713	10.465	33.944	1.00	0.00	H
ATOM	4027	CA	VAL	A	281	15.765	10.305	33.292	1.00	0.00	C
ATOM	4028	HA	VAL	A	281	16.171	10.791	32.518	1.00	0.00	H
ATOM	4029	CB	VAL	A	281	16.116	11.099	34.481	1.00	0.00	C
ATOM	4030	HB	VAL	A	281	15.616	11.942	34.282	1.00	0.00	H
ATOM	4031	CG1	VAL	A	281	15.644	10.586	35.847	1.00	0.00	C
ATOM	4032	HG11	VAL	A	281	15.948	11.215	36.563	1.00	0.00	H
ATOM	4033	HG12	VAL	A	281	14.646	10.530	35.855	1.00	0.00	H
ATOM	4034	HG13	VAL	A	281	16.031	9.679	36.014	1.00	0.00	H
ATOM	4035	CG2	VAL	A	281	17.614	11.268	34.573	1.00	0.00	C
ATOM	4036	HG21	VAL	A	281	17.839	11.808	35.384	1.00	0.00	H
ATOM	4037	HG22	VAL	A	281	18.047	10.369	34.640	1.00	0.00	H
ATOM	4038	HG23	VAL	A	281	17.947	11.738	33.756	1.00	0.00	H
ATOM	4039	C	VAL	A	281	16.327	8.917	33.310	1.00	0.00	C
ATOM	4040	O	VAL	A	281	15.592	8.006	33.534	1.00	0.00	O
ATOM	4041	N	GLY	A	282	17.671	8.762	32.918	1.00	0.00	N
ATOM	4042	H	GLY	A	282	18.232	9.554	32.677	1.00	0.00	H
ATOM	4043	CA	GLY	A	282	18.228	7.391	32.874	1.00	0.00	C
ATOM	4044	HA1	GLY	A	282	17.909	6.922	33.698	1.00	0.00	H
ATOM	4045	HA2	GLY	A	282	19.223	7.481	32.906	1.00	0.00	H
ATOM	4046	C	GLY	A	282	17.825	6.558	31.617	1.00	0.00	C
ATOM	4047	O	GLY	A	282	18.176	5.399	31.449	1.00	0.00	O

ATOM	4048	N	ALA	A	283	17.166	7.257	30.665	1.00	0.00	N
ATOM	4049	H	ALA	A	283	16.942	8.204	30.895	1.00	0.00	H
ATOM	4050	CA	ALA	A	283	16.733	6.797	29.322	1.00	0.00	C
ATOM	4051	HA	ALA	A	283	16.464	5.834	29.309	1.00	0.00	H
ATOM	4052	CB	ALA	A	283	15.417	7.513	28.959	1.00	0.00	C
ATOM	4053	HB1	ALA	A	283	15.112	7.213	28.055	1.00	0.00	H
ATOM	4054	HB2	ALA	A	283	14.718	7.289	29.638	1.00	0.00	H
ATOM	4055	HB3	ALA	A	283	15.567	8.502	28.949	1.00	0.00	H
ATOM	4056	C	ALA	A	283	17.960	6.854	28.359	1.00	0.00	C
ATOM	4057	O	ALA	A	283	18.778	7.764	28.494	1.00	0.00	O
ATOM	4058	N	PRO	A	284	18.185	5.926	27.439	1.00	0.00	N
ATOM	4059	CD	PRO	A	284	17.129	4.831	27.261	1.00	0.00	C
ATOM	4060	HD1	PRO	A	284	16.209	5.219	27.198	1.00	0.00	H
ATOM	4061	HD2	PRO	A	284	17.161	4.169	28.010	1.00	0.00	H
ATOM	4062	CG	PRO	A	284	17.559	4.196	25.935	1.00	0.00	C
ATOM	4063	HG1	PRO	A	284	17.097	4.631	25.163	1.00	0.00	H
ATOM	4064	HG2	PRO	A	284	17.367	3.215	25.929	1.00	0.00	H
ATOM	4065	CB	PRO	A	284	19.029	4.428	25.846	1.00	0.00	C
ATOM	4066	HB1	PRO	A	284	19.321	4.467	24.890	1.00	0.00	H
ATOM	4067	HB2	PRO	A	284	19.528	3.701	26.317	1.00	0.00	H
ATOM	4068	CA	PRO	A	284	19.272	5.727	26.508	1.00	0.00	C
ATOM	4069	HA	PRO	A	284	20.140	5.729	27.005	1.00	0.00	H
ATOM	4070	C	PRO	A	284	19.203	6.823	25.438	1.00	0.00	C
ATOM	4071	O	PRO	A	284	18.155	7.083	24.803	1.00	0.00	O
ATOM	4072	N	ALA	A	285	20.361	7.438	25.130	1.00	0.00	N
ATOM	4073	H	ALA	A	285	21.203	7.113	25.561	1.00	0.00	H
ATOM	4074	CA	ALA	A	285	20.443	8.533	24.217	1.00	0.00	C
ATOM	4075	HA	ALA	A	285	19.639	8.614	23.628	1.00	0.00	H
ATOM	4076	CB	ALA	A	285	20.511	9.776	25.106	1.00	0.00	C
ATOM	4077	HB1	ALA	A	285	20.571	10.593	24.533	1.00	0.00	H
ATOM	4078	HB2	ALA	A	285	19.689	9.826	25.673	1.00	0.00	H
ATOM	4079	HB3	ALA	A	285	21.318	9.722	25.694	1.00	0.00	H
ATOM	4080	C	ALA	A	285	21.652	8.265	23.290	1.00	0.00	C
ATOM	4081	O	ALA	A	285	22.500	7.399	23.471	1.00	0.00	O
ATOM	4082	N	THR	A	286	21.618	9.018	22.162	1.00	0.00	N
ATOM	4083	H	THR	A	286	20.872	9.684	22.133	1.00	0.00	H
ATOM	4084	CA	THR	A	286	22.497	9.027	20.958	1.00	0.00	C
ATOM	4085	HA	THR	A	286	23.261	8.472	21.287	1.00	0.00	H
ATOM	4086	CB	THR	A	286	21.874	8.338	19.752	1.00	0.00	C
ATOM	4087	HB	THR	A	286	22.381	8.594	18.929	1.00	0.00	H
ATOM	4088	CG2	THR	A	286	21.811	6.800	20.035	1.00	0.00	C
ATOM	4089	HG21	THR	A	286	21.403	6.333	19.250	1.00	0.00	H
ATOM	4090	HG22	THR	A	286	22.736	6.451	20.187	1.00	0.00	H
ATOM	4091	HG23	THR	A	286	21.254	6.633	20.848	1.00	0.00	H
ATOM	4092	OG1	THR	A	286	20.581	8.817	19.454	1.00	0.00	O
ATOM	4093	HG1	THR	A	286	20.221	8.332	18.657	1.00	0.00	H
ATOM	4094	C	THR	A	286	23.033	10.427	20.544	1.00	0.00	C
ATOM	4095	O	THR	A	286	22.873	10.909	19.384	1.00	0.00	O
ATOM	4096	N	GLY	A	287	23.586	11.167	21.475	1.00	0.00	N
ATOM	4097	H	GLY	A	287	23.703	10.761	22.381	1.00	0.00	H
ATOM	4098	CA	GLY	A	287	24.052	12.566	21.281	1.00	0.00	C
ATOM	4099	HA1	GLY	A	287	24.346	12.675	20.331	1.00	0.00	H
ATOM	4100	HA2	GLY	A	287	24.827	12.731	21.891	1.00	0.00	H
ATOM	4101	C	GLY	A	287	22.918	13.556	21.595	1.00	0.00	C

ATOM	4102	O	GLY	A	287	22.043	13.254	22.362	1.00	0.00	O
ATOM	4103	N	SER	A	288	23.025	14.768	21.005	1.00	0.00	N
ATOM	4104	H	SER	A	288	23.649	14.839	20.227	1.00	0.00	H
ATOM	4105	CA	SER	A	288	22.317	15.980	21.396	1.00	0.00	C
ATOM	4106	HA	SER	A	288	21.931	15.685	22.270	1.00	0.00	H
ATOM	4107	CB	SER	A	288	23.384	17.142	21.334	1.00	0.00	C
ATOM	4108	HB1	SER	A	288	24.216	16.898	21.832	1.00	0.00	H
ATOM	4109	HB2	SER	A	288	23.014	17.995	21.701	1.00	0.00	H
ATOM	4110	OG	SER	A	288	23.760	17.392	19.978	1.00	0.00	O
ATOM	4111	HG	SER	A	288	24.435	18.129	19.950	1.00	0.00	H
ATOM	4112	C	SER	A	288	21.075	16.437	20.643	1.00	0.00	C
ATOM	4113	O	SER	A	288	20.032	16.904	21.213	1.00	0.00	O
ATOM	4114	N	GLY	A	289	21.156	16.151	19.372	1.00	0.00	N
ATOM	4115	H	GLY	A	289	21.974	15.677	19.045	1.00	0.00	H
ATOM	4116	CA	GLY	A	289	20.067	16.502	18.354	1.00	0.00	C
ATOM	4117	HA1	GLY	A	289	19.220	16.705	18.846	1.00	0.00	H
ATOM	4118	HA2	GLY	A	289	19.923	15.716	17.753	1.00	0.00	H
ATOM	4119	C	GLY	A	289	20.410	17.696	17.493	1.00	0.00	C
ATOM	4120	O	GLY	A	289	21.606	18.056	17.368	1.00	0.00	O
ATOM	4121	N	THR	A	290	19.403	18.276	16.861	1.00	0.00	N
ATOM	4122	H	THR	A	290	18.550	17.758	16.799	1.00	0.00	H
ATOM	4123	CA	THR	A	290	19.417	19.608	16.241	1.00	0.00	C
ATOM	4124	HA	THR	A	290	20.189	19.549	15.608	1.00	0.00	H
ATOM	4125	CB	THR	A	290	18.129	19.958	15.448	1.00	0.00	C
ATOM	4126	HB	THR	A	290	17.346	19.802	16.050	1.00	0.00	H
ATOM	4127	CG2	THR	A	290	18.054	21.363	14.845	1.00	0.00	C
ATOM	4128	HG21	THR	A	290	17.186	21.472	14.361	1.00	0.00	H
ATOM	4129	HG22	THR	A	290	18.116	22.043	15.576	1.00	0.00	H
ATOM	4130	HG23	THR	A	290	18.811	21.492	14.204	1.00	0.00	H
ATOM	4131	OG1	THR	A	290	18.180	19.154	14.287	1.00	0.00	O
ATOM	4132	HG1	THR	A	290	17.372	19.330	13.724	1.00	0.00	H
ATOM	4133	C	THR	A	290	19.670	20.670	17.274	1.00	0.00	C
ATOM	4134	O	THR	A	290	18.944	20.743	18.221	1.00	0.00	O
ATOM	4135	N	PRO	A	291	20.750	21.477	17.226	1.00	0.00	N
ATOM	4136	CD	PRO	A	291	21.731	21.536	16.167	1.00	0.00	C
ATOM	4137	HD1	PRO	A	291	21.271	21.823	15.327	1.00	0.00	H
ATOM	4138	HD2	PRO	A	291	22.114	20.621	16.041	1.00	0.00	H
ATOM	4139	CG	PRO	A	291	22.825	22.514	16.521	1.00	0.00	C
ATOM	4140	HG1	PRO	A	291	22.746	23.345	15.971	1.00	0.00	H
ATOM	4141	HG2	PRO	A	291	23.725	22.103	16.377	1.00	0.00	H
ATOM	4142	CB	PRO	A	291	22.553	22.793	18.030	1.00	0.00	C
ATOM	4143	HB1	PRO	A	291	22.738	23.752	18.244	1.00	0.00	H
ATOM	4144	HB2	PRO	A	291	23.129	22.207	18.600	1.00	0.00	H
ATOM	4145	CA	PRO	A	291	21.045	22.466	18.267	1.00	0.00	C
ATOM	4146	HA	PRO	A	291	20.853	22.052	19.157	1.00	0.00	H
ATOM	4147	C	PRO	A	291	20.140	23.791	18.121	1.00	0.00	C
ATOM	4148	O	PRO	A	291	19.702	24.144	17.057	1.00	0.00	O
ATOM	4149	N	GLY	A	292	19.834	24.442	19.265	1.00	0.00	N
ATOM	4150	H	GLY	A	292	20.383	24.230	20.074	1.00	0.00	H
ATOM	4151	CA	GLY	A	292	18.782	25.416	19.417	1.00	0.00	C
ATOM	4152	HA1	GLY	A	292	18.693	25.640	20.387	1.00	0.00	H
ATOM	4153	HA2	GLY	A	292	17.927	25.016	19.087	1.00	0.00	H
ATOM	4154	C	GLY	A	292	19.193	26.663	18.576	1.00	0.00	C
ATOM	4155	O	GLY	A	292	20.379	27.101	18.676	1.00	0.00	O

ATOM	4156	N	PRO	A	293	18.312	27.256	17.782	1.00	0.00	N
ATOM	4157	CD	PRO	A	293	17.014	26.778	17.478	1.00	0.00	C
ATOM	4158	HD1	PRO	A	293	16.620	26.374	18.304	1.00	0.00	H
ATOM	4159	HD2	PRO	A	293	17.089	26.078	16.768	1.00	0.00	H
ATOM	4160	CG	PRO	A	293	16.207	28.002	16.996	1.00	0.00	C
ATOM	4161	HG1	PRO	A	293	15.928	28.605	17.743	1.00	0.00	H
ATOM	4162	HG2	PRO	A	293	15.406	27.752	16.453	1.00	0.00	H
ATOM	4163	CB	PRO	A	293	17.306	28.603	16.154	1.00	0.00	C
ATOM	4164	HB1	PRO	A	293	17.130	29.571	15.977	1.00	0.00	H
ATOM	4165	HB2	PRO	A	293	17.395	28.116	15.285	1.00	0.00	H
ATOM	4166	CA	PRO	A	293	18.542	28.447	16.956	1.00	0.00	C
ATOM	4167	HA	PRO	A	293	19.340	28.292	16.373	1.00	0.00	H
ATOM	4168	C	PRO	A	293	18.816	29.714	17.803	1.00	0.00	C
ATOM	4169	O	PRO	A	293	19.325	30.644	17.271	1.00	0.00	O
ATOM	4170	N	PHE	A	294	18.513	29.770	19.122	1.00	0.00	N
ATOM	4171	H	PHE	A	294	17.914	29.059	19.489	1.00	0.00	H
ATOM	4172	CA	PHE	A	294	19.005	30.811	20.066	1.00	0.00	C
ATOM	4173	HA	PHE	A	294	19.050	31.645	19.516	1.00	0.00	H
ATOM	4174	CB	PHE	A	294	18.089	31.115	21.279	1.00	0.00	C
ATOM	4175	HB1	PHE	A	294	18.529	31.828	21.825	1.00	0.00	H
ATOM	4176	HB2	PHE	A	294	18.012	30.279	21.822	1.00	0.00	H
ATOM	4177	CG	PHE	A	294	16.652	31.595	20.932	1.00	0.00	C
ATOM	4178	CD1	PHE	A	294	16.301	32.108	19.647	1.00	0.00	C
ATOM	4179	HD1	PHE	A	294	17.002	32.231	18.944	1.00	0.00	H
ATOM	4180	CE1	PHE	A	294	14.988	32.433	19.383	1.00	0.00	C
ATOM	4181	HE1	PHE	A	294	14.726	32.705	18.457	1.00	0.00	H
ATOM	4182	CZ	PHE	A	294	14.034	32.389	20.370	1.00	0.00	C
ATOM	4183	HZ	PHE	A	294	13.080	32.601	20.160	1.00	0.00	H
ATOM	4184	CE2	PHE	A	294	14.404	32.051	21.646	1.00	0.00	C
ATOM	4185	HE2	PHE	A	294	13.708	32.062	22.364	1.00	0.00	H
ATOM	4186	CD2	PHE	A	294	15.694	31.696	21.983	1.00	0.00	C
ATOM	4187	HD2	PHE	A	294	15.949	31.516	22.933	1.00	0.00	H
ATOM	4188	C	PHE	A	294	20.353	30.444	20.525	1.00	0.00	C
ATOM	4189	O	PHE	A	294	21.302	31.202	20.310	1.00	0.00	O
ATOM	4190	N	THR	A	295	20.441	29.313	21.208	1.00	0.00	N
ATOM	4191	H	THR	A	295	19.686	28.657	21.193	1.00	0.00	H
ATOM	4192	CA	THR	A	295	21.687	29.017	22.011	1.00	0.00	C
ATOM	4193	HA	THR	A	295	21.929	29.840	22.524	1.00	0.00	H
ATOM	4194	CB	THR	A	295	21.443	27.877	23.021	1.00	0.00	C
ATOM	4195	HB	THR	A	295	21.338	27.054	22.463	1.00	0.00	H
ATOM	4196	CG2	THR	A	295	22.561	27.719	24.051	1.00	0.00	C
ATOM	4197	HG21	THR	A	295	22.339	26.966	24.671	1.00	0.00	H
ATOM	4198	HG22	THR	A	295	23.421	27.521	23.581	1.00	0.00	H
ATOM	4199	HG23	THR	A	295	22.654	28.566	24.575	1.00	0.00	H
ATOM	4200	OG1	THR	A	295	20.225	28.027	23.769	1.00	0.00	O
ATOM	4201	HG1	THR	A	295	20.126	27.260	24.403	1.00	0.00	H
ATOM	4202	C	THR	A	295	22.850	28.750	21.111	1.00	0.00	C
ATOM	4203	O	THR	A	295	23.951	29.156	21.484	1.00	0.00	O
ATOM	4204	N	LYS	A	296	22.713	28.134	19.933	1.00	0.00	N
ATOM	4205	H	LYS	A	296	21.776	28.027	19.600	1.00	0.00	H
ATOM	4206	CA	LYS	A	296	23.780	27.589	19.059	1.00	0.00	C
ATOM	4207	HA	LYS	A	296	23.232	27.046	18.422	1.00	0.00	H
ATOM	4208	CB	LYS	A	296	24.538	28.726	18.338	1.00	0.00	C
ATOM	4209	HB1	LYS	A	296	25.174	28.309	17.688	1.00	0.00	H

ATOM	4210	HB2	LYS	A	296	25.052	29.239	19.026	1.00	0.00	H
ATOM	4211	CG	LYS	A	296	23.656	29.726	17.563	1.00	0.00	C
ATOM	4212	HG1	LYS	A	296	24.249	30.414	17.144	1.00	0.00	H
ATOM	4213	HG2	LYS	A	296	23.036	30.170	18.210	1.00	0.00	H
ATOM	4214	CD	LYS	A	296	22.814	29.071	16.455	1.00	0.00	C
ATOM	4215	HD1	LYS	A	296	22.272	28.331	16.853	1.00	0.00	H
ATOM	4216	HD2	LYS	A	296	23.427	28.698	15.758	1.00	0.00	H
ATOM	4217	CE	LYS	A	296	21.866	30.078	15.793	1.00	0.00	C
ATOM	4218	HE1	LYS	A	296	21.425	30.629	16.502	1.00	0.00	H
ATOM	4219	HE2	LYS	A	296	21.169	29.582	15.275	1.00	0.00	H
ATOM	4220	NZ	LYS	A	296	22.543	31.007	14.858	1.00	0.00	N
ATOM	4221	HZ1	LYS	A	296	21.870	31.633	14.464	1.00	0.00	H
ATOM	4222	HZ2	LYS	A	296	23.236	31.531	15.353	1.00	0.00	H
ATOM	4223	HZ3	LYS	A	296	22.980	30.484	14.126	1.00	0.00	H
ATOM	4224	C	LYS	A	296	24.757	26.569	19.683	1.00	0.00	C
ATOM	4225	O	LYS	A	296	25.173	25.662	18.968	1.00	0.00	O
ATOM	4226	N	GLU	A	297	25.081	26.647	20.976	1.00	0.00	N
ATOM	4227	H	GLU	A	297	24.665	27.376	21.519	1.00	0.00	H
ATOM	4228	CA	GLU	A	297	26.012	25.728	21.655	1.00	0.00	C
ATOM	4229	HA	GLU	A	297	26.841	25.780	21.099	1.00	0.00	H
ATOM	4230	CB	GLU	A	297	26.310	26.185	23.096	1.00	0.00	C
ATOM	4231	HB1	GLU	A	297	27.089	25.652	23.426	1.00	0.00	H
ATOM	4232	HB2	GLU	A	297	25.504	25.977	23.651	1.00	0.00	H
ATOM	4233	CG	GLU	A	297	26.645	27.667	23.315	1.00	0.00	C
ATOM	4234	HG1	GLU	A	297	26.944	27.782	24.262	1.00	0.00	H
ATOM	4235	HG2	GLU	A	297	25.813	28.200	23.161	1.00	0.00	H
ATOM	4236	CD	GLU	A	297	27.740	28.225	22.402	1.00	0.00	C
ATOM	4237	OE1	GLU	A	297	27.622	29.433	22.076	1.00	0.00	O
ATOM	4238	OE2	GLU	A	297	28.698	27.488	22.086	1.00	0.00	O
ATOM	4239	C	GLU	A	297	25.473	24.279	21.679	1.00	0.00	C
ATOM	4240	O	GLU	A	297	24.441	23.994	22.287	1.00	0.00	O
ATOM	4241	N	GLY	A	298	26.193	23.342	21.051	1.00	0.00	N
ATOM	4242	H	GLY	A	298	27.165	23.520	20.898	1.00	0.00	H
ATOM	4243	CA	GLY	A	298	25.638	22.064	20.572	1.00	0.00	C
ATOM	4244	HA1	GLY	A	298	26.420	21.498	20.311	1.00	0.00	H
ATOM	4245	HA2	GLY	A	298	25.096	22.279	19.759	1.00	0.00	H
ATOM	4246	C	GLY	A	298	24.758	21.261	21.546	1.00	0.00	C
ATOM	4247	O	GLY	A	298	23.623	20.930	21.179	1.00	0.00	O
ATOM	4248	N	GLY	A	299	25.273	20.979	22.748	1.00	0.00	N
ATOM	4249	H	GLY	A	299	26.159	21.384	22.975	1.00	0.00	H
ATOM	4250	CA	GLY	A	299	24.643	20.123	23.762	1.00	0.00	C
ATOM	4251	HA1	GLY	A	299	25.362	19.544	24.146	1.00	0.00	H
ATOM	4252	HA2	GLY	A	299	23.972	19.552	23.289	1.00	0.00	H
ATOM	4253	C	GLY	A	299	23.946	20.863	24.899	1.00	0.00	C
ATOM	4254	O	GLY	A	299	23.951	20.363	26.029	1.00	0.00	O
ATOM	4255	N	MET	A	300	23.455	22.083	24.664	1.00	0.00	N
ATOM	4256	H	MET	A	300	23.569	22.477	23.752	1.00	0.00	H
ATOM	4257	CA	MET	A	300	22.756	22.870	25.685	1.00	0.00	C
ATOM	4258	HA	MET	A	300	22.240	22.180	26.192	1.00	0.00	H
ATOM	4259	CB	MET	A	300	23.763	23.573	26.604	1.00	0.00	C
ATOM	4260	HB1	MET	A	300	24.310	22.876	27.067	1.00	0.00	H
ATOM	4261	HB2	MET	A	300	23.256	24.104	27.283	1.00	0.00	H
ATOM	4262	CG	MET	A	300	24.712	24.515	25.879	1.00	0.00	C
ATOM	4263	HG1	MET	A	300	24.125	25.154	25.381	1.00	0.00	H

ATOM	4264	HG2	MET	A	300	25.210	23.943	25.227	1.00	0.00	H
ATOM	4265	SD	MET	A	300	25.808	25.348	27.044	1.00	0.00	S
ATOM	4266	CE	MET	A	300	24.734	26.713	27.567	1.00	0.00	C
ATOM	4267	HE1	MET	A	300	25.217	27.281	28.234	1.00	0.00	H
ATOM	4268	HE2	MET	A	300	24.489	27.268	26.772	1.00	0.00	H
ATOM	4269	HE3	MET	A	300	23.904	26.343	27.984	1.00	0.00	H
ATOM	4270	C	MET	A	300	21.719	23.849	25.130	1.00	0.00	C
ATOM	4271	O	MET	A	300	21.728	24.191	23.952	1.00	0.00	O
ATOM	4272	N	LEU	A	301	20.781	24.248	25.994	1.00	0.00	N
ATOM	4273	H	LEU	A	301	20.863	23.942	26.942	1.00	0.00	H
ATOM	4274	CA	LEU	A	301	19.644	25.100	25.652	1.00	0.00	C
ATOM	4275	HA	LEU	A	301	19.925	25.550	24.804	1.00	0.00	H
ATOM	4276	CB	LEU	A	301	18.360	24.249	25.458	1.00	0.00	C
ATOM	4277	HB1	LEU	A	301	17.583	24.864	25.324	1.00	0.00	H
ATOM	4278	HB2	LEU	A	301	18.211	23.698	26.279	1.00	0.00	H
ATOM	4279	CG	LEU	A	301	18.463	23.303	24.236	1.00	0.00	C
ATOM	4280	HG	LEU	A	301	19.320	22.798	24.337	1.00	0.00	H
ATOM	4281	CD1	LEU	A	301	17.241	22.422	24.165	1.00	0.00	C
ATOM	4282	HD11	LEU	A	301	17.314	21.814	23.374	1.00	0.00	H
ATOM	4283	HD12	LEU	A	301	17.175	21.876	25.000	1.00	0.00	H
ATOM	4284	HD13	LEU	A	301	16.424	22.992	24.073	1.00	0.00	H
ATOM	4285	CD2	LEU	A	301	18.406	24.052	22.922	1.00	0.00	C
ATOM	4286	HD21	LEU	A	301	18.476	23.403	22.165	1.00	0.00	H
ATOM	4287	HD22	LEU	A	301	17.539	24.547	22.858	1.00	0.00	H
ATOM	4288	HD23	LEU	A	301	19.165	24.701	22.875	1.00	0.00	H
ATOM	4289	C	LEU	A	301	19.348	26.095	26.839	1.00	0.00	C
ATOM	4290	O	LEU	A	301	19.127	25.702	28.004	1.00	0.00	O
ATOM	4291	N	ALA	A	302	19.410	27.387	26.501	1.00	0.00	N
ATOM	4292	H	ALA	A	302	19.644	27.651	25.565	1.00	0.00	H
ATOM	4293	CA	ALA	A	302	19.124	28.451	27.543	1.00	0.00	C
ATOM	4294	HA	ALA	A	302	19.828	28.253	28.225	1.00	0.00	H
ATOM	4295	CB	ALA	A	302	19.244	29.859	26.918	1.00	0.00	C
ATOM	4296	HB1	ALA	A	302	19.053	30.550	27.615	1.00	0.00	H
ATOM	4297	HB2	ALA	A	302	20.170	29.989	26.564	1.00	0.00	H
ATOM	4298	HB3	ALA	A	302	18.586	29.949	26.170	1.00	0.00	H
ATOM	4299	C	ALA	A	302	17.694	28.395	28.219	1.00	0.00	C
ATOM	4300	O	ALA	A	302	16.756	27.870	27.602	1.00	0.00	O
ATOM	4301	N	TYR	A	303	17.609	28.896	29.389	1.00	0.00	N
ATOM	4302	H	TYR	A	303	18.456	29.114	29.875	1.00	0.00	H
ATOM	4303	CA	TYR	A	303	16.338	29.168	30.056	1.00	0.00	C
ATOM	4304	HA	TYR	A	303	16.007	28.233	30.184	1.00	0.00	H
ATOM	4305	CB	TYR	A	303	16.529	29.741	31.445	1.00	0.00	C
ATOM	4306	HB1	TYR	A	303	16.991	30.623	31.352	1.00	0.00	H
ATOM	4307	HB2	TYR	A	303	17.113	29.112	31.959	1.00	0.00	H
ATOM	4308	CG	TYR	A	303	15.221	29.951	32.231	1.00	0.00	C
ATOM	4309	CD1	TYR	A	303	14.625	28.811	32.795	1.00	0.00	C
ATOM	4310	HD1	TYR	A	303	15.094	27.935	32.684	1.00	0.00	H
ATOM	4311	CE1	TYR	A	303	13.415	28.823	33.504	1.00	0.00	C
ATOM	4312	HE1	TYR	A	303	12.981	27.987	33.841	1.00	0.00	H
ATOM	4313	CZ	TYR	A	303	12.852	30.112	33.708	1.00	0.00	C
ATOM	4314	OH	TYR	A	303	11.794	30.165	34.499	1.00	0.00	O
ATOM	4315	HH	TYR	A	303	11.480	31.112	34.573	1.00	0.00	H
ATOM	4316	CE2	TYR	A	303	13.505	31.291	33.311	1.00	0.00	C
ATOM	4317	HE2	TYR	A	303	13.141	32.176	33.601	1.00	0.00	H

ATOM	4318	CD2	TYR	A	303	14.652	31.247	32.520	1.00	0.00	C
ATOM	4319	HD2	TYR	A	303	15.067	32.084	32.165	1.00	0.00	H
ATOM	4320	C	TYR	A	303	15.243	29.902	29.192	1.00	0.00	C
ATOM	4321	O	TYR	A	303	14.143	29.428	28.972	1.00	0.00	O
ATOM	4322	N	TYR	A	304	15.643	30.987	28.517	1.00	0.00	N
ATOM	4323	H	TYR	A	304	16.551	31.357	28.713	1.00	0.00	H
ATOM	4324	CA	TYR	A	304	14.816	31.668	27.498	1.00	0.00	C
ATOM	4325	HA	TYR	A	304	13.923	31.778	27.935	1.00	0.00	H
ATOM	4326	CB	TYR	A	304	15.475	33.059	27.134	1.00	0.00	C
ATOM	4327	HB1	TYR	A	304	15.915	33.394	27.967	1.00	0.00	H
ATOM	4328	HB2	TYR	A	304	14.727	33.673	26.882	1.00	0.00	H
ATOM	4329	CG	TYR	A	304	16.543	33.188	25.998	1.00	0.00	C
ATOM	4330	CD1	TYR	A	304	16.166	33.511	24.662	1.00	0.00	C
ATOM	4331	HD1	TYR	A	304	15.203	33.504	24.393	1.00	0.00	H
ATOM	4332	CE1	TYR	A	304	17.210	33.856	23.681	1.00	0.00	C
ATOM	4333	HE1	TYR	A	304	16.971	34.212	22.778	1.00	0.00	H
ATOM	4334	CZ	TYR	A	304	18.550	33.669	24.054	1.00	0.00	C
ATOM	4335	OH	TYR	A	304	19.650	33.933	23.319	1.00	0.00	O
ATOM	4336	HH	TYR	A	304	20.467	33.699	23.846	1.00	0.00	H
ATOM	4337	CE2	TYR	A	304	18.861	33.314	25.416	1.00	0.00	C
ATOM	4338	HE2	TYR	A	304	19.816	33.272	25.710	1.00	0.00	H
ATOM	4339	CD2	TYR	A	304	17.857	33.032	26.319	1.00	0.00	C
ATOM	4340	HD2	TYR	A	304	18.100	32.705	27.232	1.00	0.00	H
ATOM	4341	C	TYR	A	304	14.492	30.815	26.274	1.00	0.00	C
ATOM	4342	O	TYR	A	304	13.447	30.974	25.647	1.00	0.00	O
ATOM	4343	N	GLU	A	305	15.316	29.856	25.885	1.00	0.00	N
ATOM	4344	H	GLU	A	305	16.172	29.745	26.389	1.00	0.00	H
ATOM	4345	CA	GLU	A	305	15.054	28.925	24.741	1.00	0.00	C
ATOM	4346	HA	GLU	A	305	14.618	29.502	24.051	1.00	0.00	H
ATOM	4347	CB	GLU	A	305	16.376	28.325	24.205	1.00	0.00	C
ATOM	4348	HB1	GLU	A	305	16.801	27.771	24.921	1.00	0.00	H
ATOM	4349	HB2	GLU	A	305	16.995	29.067	23.946	1.00	0.00	H
ATOM	4350	CG	GLU	A	305	16.108	27.439	22.973	1.00	0.00	C
ATOM	4351	HG1	GLU	A	305	15.195	27.663	22.633	1.00	0.00	H
ATOM	4352	HG2	GLU	A	305	16.127	26.486	23.276	1.00	0.00	H
ATOM	4353	CD	GLU	A	305	17.129	27.604	21.795	1.00	0.00	C
ATOM	4354	OE1	GLU	A	305	18.385	27.506	21.942	1.00	0.00	O
ATOM	4355	OE2	GLU	A	305	16.698	27.815	20.647	1.00	0.00	O
ATOM	4356	C	GLU	A	305	14.079	27.788	25.097	1.00	0.00	C
ATOM	4357	O	GLU	A	305	13.235	27.537	24.193	1.00	0.00	O
ATOM	4358	N	VAL	A	306	14.117	27.199	26.270	1.00	0.00	N
ATOM	4359	H	VAL	A	306	14.849	27.432	26.911	1.00	0.00	H
ATOM	4360	CA	VAL	A	306	13.066	26.163	26.679	1.00	0.00	C
ATOM	4361	HA	VAL	A	306	12.902	25.570	25.891	1.00	0.00	H
ATOM	4362	CB	VAL	A	306	13.551	25.238	27.803	1.00	0.00	C
ATOM	4363	HB	VAL	A	306	12.783	24.656	28.069	1.00	0.00	H
ATOM	4364	CG1	VAL	A	306	14.568	24.288	27.358	1.00	0.00	C
ATOM	4365	HG11	VAL	A	306	14.846	23.713	28.127	1.00	0.00	H
ATOM	4366	HG12	VAL	A	306	14.192	23.713	26.631	1.00	0.00	H
ATOM	4367	HG13	VAL	A	306	15.362	24.788	27.011	1.00	0.00	H
ATOM	4368	CG2	VAL	A	306	14.026	26.026	29.024	1.00	0.00	C
ATOM	4369	HG21	VAL	A	306	14.333	25.390	29.732	1.00	0.00	H
ATOM	4370	HG22	VAL	A	306	14.784	26.623	28.761	1.00	0.00	H
ATOM	4371	HG23	VAL	A	306	13.272	26.580	29.377	1.00	0.00	H

ATOM	4372	C	VAL	A	306	11.738	26.803	26.962	1.00	0.00	C
ATOM	4373	O	VAL	A	306	10.669	26.282	26.784	1.00	0.00	O
ATOM	4374	N	CYS	A	307	11.809	28.047	27.449	1.00	0.00	N
ATOM	4375	H	CYS	A	307	12.710	28.411	27.685	1.00	0.00	H
ATOM	4376	CA	CYS	A	307	10.614	28.925	27.661	1.00	0.00	C
ATOM	4377	HA	CYS	A	307	10.018	28.483	28.331	1.00	0.00	H
ATOM	4378	CB	CYS	A	307	10.966	30.284	28.241	1.00	0.00	C
ATOM	4379	HB1	CYS	A	307	10.165	30.829	27.991	1.00	0.00	H
ATOM	4380	HB2	CYS	A	307	11.744	30.555	27.674	1.00	0.00	H
ATOM	4381	SG	CYS	A	307	11.352	30.429	30.037	1.00	0.00	S
ATOM	4382	C	CYS	A	307	9.909	29.068	26.347	1.00	0.00	C
ATOM	4383	O	CYS	A	307	8.733	28.807	26.347	1.00	0.00	O
ATOM	4384	N	SER	A	308	10.702	29.259	25.242	1.00	0.00	N
ATOM	4385	H	SER	A	308	11.696	29.255	25.353	1.00	0.00	H
ATOM	4386	CA	SER	A	308	10.132	29.471	23.889	1.00	0.00	C
ATOM	4387	HA	SER	A	308	9.307	30.029	23.981	1.00	0.00	H
ATOM	4388	CB	SER	A	308	11.263	30.147	23.067	1.00	0.00	C
ATOM	4389	HB1	SER	A	308	11.003	30.262	22.108	1.00	0.00	H
ATOM	4390	HB2	SER	A	308	12.117	29.630	23.121	1.00	0.00	H
ATOM	4391	OG	SER	A	308	11.532	31.406	23.557	1.00	0.00	O
ATOM	4392	HG	SER	A	308	12.261	31.825	23.016	1.00	0.00	H
ATOM	4393	C	SER	A	308	9.722	28.134	23.214	1.00	0.00	C
ATOM	4394	O	SER	A	308	9.015	28.205	22.181	1.00	0.00	O
ATOM	4395	N	TRP	A	309	9.915	26.938	23.831	1.00	0.00	N
ATOM	4396	H	TRP	A	309	10.480	26.961	24.655	1.00	0.00	H
ATOM	4397	CA	TRP	A	309	9.402	25.615	23.446	1.00	0.00	C
ATOM	4398	HA	TRP	A	309	9.444	25.597	22.447	1.00	0.00	H
ATOM	4399	CB	TRP	A	309	10.298	24.402	23.980	1.00	0.00	C
ATOM	4400	HB1	TRP	A	309	9.786	23.549	23.875	1.00	0.00	H
ATOM	4401	HB2	TRP	A	309	10.503	24.551	24.947	1.00	0.00	H
ATOM	4402	CG	TRP	A	309	11.599	24.118	23.375	1.00	0.00	C
ATOM	4403	CD1	TRP	A	309	12.437	24.951	22.794	1.00	0.00	C
ATOM	4404	HD1	TRP	A	309	12.312	25.938	22.696	1.00	0.00	H
ATOM	4405	NE1	TRP	A	309	13.546	24.202	22.332	1.00	0.00	N
ATOM	4406	HE1	TRP	A	309	14.350	24.564	21.861	1.00	0.00	H
ATOM	4407	CE2	TRP	A	309	13.306	22.849	22.660	1.00	0.00	C
ATOM	4408	CZ2	TRP	A	309	14.160	21.761	22.547	1.00	0.00	C
ATOM	4409	HZ2	TRP	A	309	15.027	21.851	22.057	1.00	0.00	H
ATOM	4410	CH2	TRP	A	309	13.797	20.519	23.131	1.00	0.00	C
ATOM	4411	HH2	TRP	A	309	14.368	19.712	22.979	1.00	0.00	H
ATOM	4412	CZ3	TRP	A	309	12.640	20.419	23.921	1.00	0.00	C
ATOM	4413	HZ3	TRP	A	309	12.401	19.560	24.374	1.00	0.00	H
ATOM	4414	CE3	TRP	A	309	11.828	21.556	24.058	1.00	0.00	C
ATOM	4415	HE3	TRP	A	309	10.974	21.485	24.574	1.00	0.00	H
ATOM	4416	CD2	TRP	A	309	12.192	22.819	23.476	1.00	0.00	C
ATOM	4417	C	TRP	A	309	7.931	25.531	24.000	1.00	0.00	C
ATOM	4418	O	TRP	A	309	7.678	24.927	25.048	1.00	0.00	O
ATOM	4419	N	LYS	A	310	7.030	26.275	23.363	1.00	0.00	N
ATOM	4420	H	LYS	A	310	7.343	26.771	22.553	1.00	0.00	H
ATOM	4421	CA	LYS	A	310	5.633	26.434	23.735	1.00	0.00	C
ATOM	4422	HA	LYS	A	310	5.650	26.697	24.700	1.00	0.00	H
ATOM	4423	CB	LYS	A	310	5.014	27.641	22.980	1.00	0.00	C
ATOM	4424	HB1	LYS	A	310	4.924	27.420	22.009	1.00	0.00	H
ATOM	4425	HB2	LYS	A	310	5.603	28.442	23.086	1.00	0.00	H

ATOM	4426	CG	LYS	A	310	3.623	27.950	23.564	1.00	0.00	C
ATOM	4427	HG1	LYS	A	310	3.675	27.891	24.561	1.00	0.00	H
ATOM	4428	HG2	LYS	A	310	2.975	27.269	23.223	1.00	0.00	H
ATOM	4429	CD	LYS	A	310	3.114	29.353	23.179	1.00	0.00	C
ATOM	4430	HD1	LYS	A	310	2.243	29.479	23.653	1.00	0.00	H
ATOM	4431	HD2	LYS	A	310	2.957	29.337	22.192	1.00	0.00	H
ATOM	4432	CE	LYS	A	310	4.023	30.605	23.501	1.00	0.00	C
ATOM	4433	HE1	LYS	A	310	3.562	31.446	23.219	1.00	0.00	H
ATOM	4434	HE2	LYS	A	310	4.897	30.528	23.022	1.00	0.00	H
ATOM	4435	NZ	LYS	A	310	4.344	30.770	24.905	1.00	0.00	N
ATOM	4436	HZ1	LYS	A	310	4.919	31.580	25.023	1.00	0.00	H
ATOM	4437	HZ2	LYS	A	310	3.500	30.881	25.429	1.00	0.00	H
ATOM	4438	HZ3	LYS	A	310	4.835	29.963	25.232	1.00	0.00	H
ATOM	4439	C	LYS	A	310	4.835	25.074	23.717	1.00	0.00	C
ATOM	4440	O	LYS	A	310	5.097	24.238	22.890	1.00	0.00	O
ATOM	4441	N	GLY	A	311	3.854	24.867	24.684	1.00	0.00	N
ATOM	4442	H	GLY	A	311	3.618	25.610	25.311	1.00	0.00	H
ATOM	4443	CA	GLY	A	311	3.128	23.518	24.805	1.00	0.00	C
ATOM	4444	HA1	GLY	A	311	2.715	23.314	23.917	1.00	0.00	H
ATOM	4445	HA2	GLY	A	311	2.408	23.621	25.492	1.00	0.00	H
ATOM	4446	C	GLY	A	311	4.012	22.313	25.212	1.00	0.00	C
ATOM	4447	O	GLY	A	311	3.543	21.138	25.174	1.00	0.00	O
ATOM	4448	N	ALA	A	312	5.314	22.503	25.498	1.00	0.00	N
ATOM	4449	H	ALA	A	312	5.733	23.405	25.391	1.00	0.00	H
ATOM	4450	CA	ALA	A	312	6.076	21.403	25.952	1.00	0.00	C
ATOM	4451	HA	ALA	A	312	5.867	20.775	25.202	1.00	0.00	H
ATOM	4452	CB	ALA	A	312	7.514	21.765	25.949	1.00	0.00	C
ATOM	4453	HB1	ALA	A	312	8.054	20.987	26.270	1.00	0.00	H
ATOM	4454	HB2	ALA	A	312	7.795	22.005	25.020	1.00	0.00	H
ATOM	4455	HB3	ALA	A	312	7.662	22.547	26.555	1.00	0.00	H
ATOM	4456	C	ALA	A	312	5.672	20.758	27.385	1.00	0.00	C
ATOM	4457	O	ALA	A	312	5.218	21.409	28.316	1.00	0.00	O
ATOM	4458	N	THR	A	313	5.930	19.450	27.481	1.00	0.00	N
ATOM	4459	H	THR	A	313	6.307	19.031	26.655	1.00	0.00	H
ATOM	4460	CA	THR	A	313	5.741	18.516	28.637	1.00	0.00	C
ATOM	4461	HA	THR	A	313	4.948	18.831	29.158	1.00	0.00	H
ATOM	4462	CB	THR	A	313	5.478	17.090	28.071	1.00	0.00	C
ATOM	4463	HB	THR	A	313	6.305	16.751	27.623	1.00	0.00	H
ATOM	4464	CG2	THR	A	313	4.914	16.213	29.191	1.00	0.00	C
ATOM	4465	HG21	THR	A	313	4.741	15.293	28.839	1.00	0.00	H
ATOM	4466	HG22	THR	A	313	5.574	16.164	29.941	1.00	0.00	H
ATOM	4467	HG23	THR	A	313	4.059	16.609	29.525	1.00	0.00	H
ATOM	4468	OG1	THR	A	313	4.529	17.053	27.062	1.00	0.00	O
ATOM	4469	HG1	THR	A	313	4.412	16.111	26.748	1.00	0.00	H
ATOM	4470	C	THR	A	313	6.883	18.584	29.658	1.00	0.00	C
ATOM	4471	O	THR	A	313	7.907	17.951	29.505	1.00	0.00	O
ATOM	4472	N	LYS	A	314	6.701	19.383	30.711	1.00	0.00	N
ATOM	4473	H	LYS	A	314	5.847	19.904	30.726	1.00	0.00	H
ATOM	4474	CA	LYS	A	314	7.612	19.591	31.860	1.00	0.00	C
ATOM	4475	HA	LYS	A	314	8.550	19.405	31.569	1.00	0.00	H
ATOM	4476	CB	LYS	A	314	7.520	21.053	32.255	1.00	0.00	C
ATOM	4477	HB1	LYS	A	314	6.598	21.222	32.603	1.00	0.00	H
ATOM	4478	HB2	LYS	A	314	7.673	21.605	31.435	1.00	0.00	H
ATOM	4479	CG	LYS	A	314	8.541	21.471	33.332	1.00	0.00	C

ATOM	4480	HG1	LYS	A	314	9.468	21.392	32.965	1.00	0.00	H
ATOM	4481	HG2	LYS	A	314	8.447	20.877	34.131	1.00	0.00	H
ATOM	4482	CD	LYS	A	314	8.256	22.944	33.732	1.00	0.00	C
ATOM	4483	HD1	LYS	A	314	8.165	23.511	32.913	1.00	0.00	H
ATOM	4484	HD2	LYS	A	314	9.000	23.296	34.300	1.00	0.00	H
ATOM	4485	CE	LYS	A	314	7.009	23.065	34.501	1.00	0.00	C
ATOM	4486	HE1	LYS	A	314	7.143	22.597	35.374	1.00	0.00	H
ATOM	4487	HE2	LYS	A	314	6.286	22.606	33.984	1.00	0.00	H
ATOM	4488	NZ	LYS	A	314	6.616	24.531	34.753	1.00	0.00	N
ATOM	4489	HZ1	LYS	A	314	5.765	24.563	35.277	1.00	0.00	H
ATOM	4490	HZ2	LYS	A	314	7.342	24.987	35.268	1.00	0.00	H
ATOM	4491	HZ3	LYS	A	314	6.485	24.996	33.878	1.00	0.00	H
ATOM	4492	C	LYS	A	314	7.292	18.600	33.025	1.00	0.00	C
ATOM	4493	O	LYS	A	314	6.130	18.444	33.342	1.00	0.00	O
ATOM	4494	N	GLN	A	315	8.314	18.101	33.747	1.00	0.00	N
ATOM	4495	H	GLN	A	315	9.242	18.335	33.456	1.00	0.00	H
ATOM	4496	CA	GLN	A	315	8.173	17.254	34.910	1.00	0.00	C
ATOM	4497	HA	GLN	A	315	7.258	17.400	35.287	1.00	0.00	H
ATOM	4498	CB	GLN	A	315	8.199	15.782	34.602	1.00	0.00	C
ATOM	4499	HB1	GLN	A	315	8.113	15.300	35.474	1.00	0.00	H
ATOM	4500	HB2	GLN	A	315	9.092	15.584	34.198	1.00	0.00	H
ATOM	4501	CG	GLN	A	315	7.135	15.227	33.662	1.00	0.00	C
ATOM	4502	HG1	GLN	A	315	7.478	14.366	33.285	1.00	0.00	H
ATOM	4503	HG2	GLN	A	315	7.006	15.887	32.922	1.00	0.00	H
ATOM	4504	CD	GLN	A	315	5.777	14.948	34.259	1.00	0.00	C
ATOM	4505	OE1	GLN	A	315	5.381	15.538	35.295	1.00	0.00	O
ATOM	4506	NE2	GLN	A	315	4.967	14.073	33.647	1.00	0.00	N
ATOM	4507	HE21	GLN	A	315	5.266	13.617	32.809	1.00	0.00	H
ATOM	4508	HE22	GLN	A	315	4.064	13.876	34.029	1.00	0.00	H
ATOM	4509	C	GLN	A	315	9.272	17.677	35.859	1.00	0.00	C
ATOM	4510	O	GLN	A	315	10.258	18.262	35.492	1.00	0.00	O
ATOM	4511	N	ARG	A	316	9.156	17.301	37.216	1.00	0.00	N
ATOM	4512	H	ARG	A	316	8.240	17.225	37.611	1.00	0.00	H
ATOM	4513	CA	ARG	A	316	10.350	17.007	38.104	1.00	0.00	C
ATOM	4514	HA	ARG	A	316	10.994	17.744	37.897	1.00	0.00	H
ATOM	4515	CB	ARG	A	316	9.951	17.083	39.571	1.00	0.00	C
ATOM	4516	HB1	ARG	A	316	10.379	16.312	40.044	1.00	0.00	H
ATOM	4517	HB2	ARG	A	316	8.956	16.995	39.621	1.00	0.00	H
ATOM	4518	CG	ARG	A	316	10.382	18.416	40.273	1.00	0.00	C
ATOM	4519	HG1	ARG	A	316	11.381	18.405	40.325	1.00	0.00	H
ATOM	4520	HG2	ARG	A	316	10.000	18.393	41.197	1.00	0.00	H
ATOM	4521	CD	ARG	A	316	9.941	19.797	39.595	1.00	0.00	C
ATOM	4522	HD1	ARG	A	316	8.969	19.952	39.774	1.00	0.00	H
ATOM	4523	HD2	ARG	A	316	10.093	19.739	38.608	1.00	0.00	H
ATOM	4524	NE	ARG	A	316	10.664	21.040	40.065	1.00	0.00	N
ATOM	4525	HE	ARG	A	316	11.129	20.922	40.942	1.00	0.00	H
ATOM	4526	CZ	ARG	A	316	10.804	22.226	39.564	1.00	0.00	C
ATOM	4527	NH1	ARG	A	316	10.181	22.572	38.558	1.00	0.00	N
ATOM	4528	HH11	ARG	A	316	9.554	21.932	38.114	1.00	0.00	H
ATOM	4529	HH12	ARG	A	316	10.303	23.492	38.186	1.00	0.00	H
ATOM	4530	NH2	ARG	A	316	11.689	23.031	39.992	1.00	0.00	N
ATOM	4531	HH21	ARG	A	316	12.300	22.754	40.734	1.00	0.00	H
ATOM	4532	HH22	ARG	A	316	11.775	23.941	39.587	1.00	0.00	H
ATOM	4533	C	ARG	A	316	11.053	15.618	37.811	1.00	0.00	C

ATOM	4534	O	ARG	A	316	10.404	14.637	37.409	1.00	0.00	O
ATOM	4535	N	ILE	A	317	12.405	15.591	37.880	1.00	0.00	N
ATOM	4536	H	ILE	A	317	12.963	16.346	37.536	1.00	0.00	H
ATOM	4537	CA	ILE	A	317	12.992	14.436	38.465	1.00	0.00	C
ATOM	4538	HA	ILE	A	317	12.505	13.675	38.037	1.00	0.00	H
ATOM	4539	CB	ILE	A	317	14.441	14.270	38.122	1.00	0.00	C
ATOM	4540	HB	ILE	A	317	14.885	15.042	38.577	1.00	0.00	H
ATOM	4541	CG2	ILE	A	317	14.969	12.925	38.673	1.00	0.00	C
ATOM	4542	HG21	ILE	A	317	15.936	12.825	38.438	1.00	0.00	H
ATOM	4543	HG22	ILE	A	317	14.864	12.908	39.667	1.00	0.00	H
ATOM	4544	HG23	ILE	A	317	14.449	12.172	38.270	1.00	0.00	H
ATOM	4545	CG1	ILE	A	317	14.770	14.420	36.622	1.00	0.00	C
ATOM	4546	HG11	ILE	A	317	14.250	15.191	36.253	1.00	0.00	H
ATOM	4547	HG12	ILE	A	317	14.504	13.581	36.148	1.00	0.00	H
ATOM	4548	CD	ILE	A	317	16.255	14.671	36.362	1.00	0.00	C
ATOM	4549	HD1	ILE	A	317	16.411	14.760	35.378	1.00	0.00	H
ATOM	4550	HD2	ILE	A	317	16.536	15.514	36.822	1.00	0.00	H
ATOM	4551	HD3	ILE	A	317	16.790	13.904	36.716	1.00	0.00	H
ATOM	4552	C	ILE	A	317	12.785	14.471	39.969	1.00	0.00	C
ATOM	4553	O	ILE	A	317	13.316	15.410	40.634	1.00	0.00	O
ATOM	4554	N	GLN	A	318	11.926	13.650	40.529	1.00	0.00	N
ATOM	4555	H	GLN	A	318	11.479	12.967	39.951	1.00	0.00	H
ATOM	4556	CA	GLN	A	318	11.578	13.681	41.991	1.00	0.00	C
ATOM	4557	HA	GLN	A	318	11.289	14.626	42.142	1.00	0.00	H
ATOM	4558	CB	GLN	A	318	10.393	12.689	42.284	1.00	0.00	C
ATOM	4559	HB1	GLN	A	318	10.229	12.674	43.270	1.00	0.00	H
ATOM	4560	HB2	GLN	A	318	10.661	11.776	41.977	1.00	0.00	H
ATOM	4561	CG	GLN	A	318	9.048	13.120	41.535	1.00	0.00	C
ATOM	4562	HG1	GLN	A	318	9.243	13.256	40.564	1.00	0.00	H
ATOM	4563	HG2	GLN	A	318	8.712	13.975	41.931	1.00	0.00	H
ATOM	4564	CD	GLN	A	318	7.906	12.118	41.618	1.00	0.00	C
ATOM	4565	OE1	GLN	A	318	7.802	11.384	42.589	1.00	0.00	O
ATOM	4566	NE2	GLN	A	318	6.995	12.123	40.679	1.00	0.00	N
ATOM	4567	HE21	GLN	A	318	7.057	12.777	39.925	1.00	0.00	H
ATOM	4568	HE22	GLN	A	318	6.236	11.473	40.715	1.00	0.00	H
ATOM	4569	C	GLN	A	318	12.809	13.415	42.954	1.00	0.00	C
ATOM	4570	O	GLN	A	318	12.956	14.195	43.847	1.00	0.00	O
ATOM	4571	N	ASP	A	319	13.604	12.358	42.717	1.00	0.00	N
ATOM	4572	H	ASP	A	319	13.464	11.807	41.894	1.00	0.00	H
ATOM	4573	CA	ASP	A	319	14.709	11.993	43.683	1.00	0.00	C
ATOM	4574	HA	ASP	A	319	14.252	11.939	44.571	1.00	0.00	H
ATOM	4575	CB	ASP	A	319	15.356	10.648	43.183	1.00	0.00	C
ATOM	4576	HB1	ASP	A	319	16.141	10.474	43.777	1.00	0.00	H
ATOM	4577	HB2	ASP	A	319	15.675	10.817	42.251	1.00	0.00	H
ATOM	4578	CG	ASP	A	319	14.628	9.309	43.104	1.00	0.00	C
ATOM	4579	OD1	ASP	A	319	13.697	9.096	43.933	1.00	0.00	O
ATOM	4580	OD2	ASP	A	319	14.993	8.465	42.237	1.00	0.00	O
ATOM	4581	C	ASP	A	319	15.890	13.058	43.746	1.00	0.00	C
ATOM	4582	O	ASP	A	319	16.748	13.011	44.649	1.00	0.00	O
ATOM	4583	N	GLN	A	320	15.849	13.920	42.702	1.00	0.00	N
ATOM	4584	H	GLN	A	320	15.073	13.892	42.072	1.00	0.00	H
ATOM	4585	CA	GLN	A	320	16.926	14.897	42.479	1.00	0.00	C
ATOM	4586	HA	GLN	A	320	17.535	14.798	43.266	1.00	0.00	H
ATOM	4587	CB	GLN	A	320	17.609	14.505	41.167	1.00	0.00	C

ATOM	4588	HB1	GLN	A	320	18.372	15.132	41.008	1.00	0.00	H
ATOM	4589	HB2	GLN	A	320	16.944	14.595	40.426	1.00	0.00	H
ATOM	4590	CG	GLN	A	320	18.151	13.078	41.154	1.00	0.00	C
ATOM	4591	HG1	GLN	A	320	17.401	12.437	40.992	1.00	0.00	H
ATOM	4592	HG2	GLN	A	320	18.579	12.874	42.035	1.00	0.00	H
ATOM	4593	CD	GLN	A	320	19.176	12.919	40.063	1.00	0.00	C
ATOM	4594	OE1	GLN	A	320	18.867	12.566	38.921	1.00	0.00	O
ATOM	4595	NE2	GLN	A	320	20.457	13.136	40.344	1.00	0.00	N
ATOM	4596	HE21	GLN	A	320	20.728	13.401	41.269	1.00	0.00	H
ATOM	4597	HE22	GLN	A	320	21.150	13.034	39.630	1.00	0.00	H
ATOM	4598	C	GLN	A	320	16.523	16.396	42.539	1.00	0.00	C
ATOM	4599	O	GLN	A	320	17.417	17.233	42.453	1.00	0.00	O
ATOM	4600	N	LYS	A	321	15.233	16.615	42.720	1.00	0.00	N
ATOM	4601	H	LYS	A	321	14.672	15.789	42.665	1.00	0.00	H
ATOM	4602	CA	LYS	A	321	14.466	17.830	42.985	1.00	0.00	C
ATOM	4603	HA	LYS	A	321	13.517	17.515	42.966	1.00	0.00	H
ATOM	4604	CB	LYS	A	321	14.841	18.346	44.333	1.00	0.00	C
ATOM	4605	HB1	LYS	A	321	14.414	19.241	44.458	1.00	0.00	H
ATOM	4606	HB2	LYS	A	321	15.836	18.443	44.368	1.00	0.00	H
ATOM	4607	CG	LYS	A	321	14.380	17.399	45.445	1.00	0.00	C
ATOM	4608	HG1	LYS	A	321	14.793	17.687	46.309	1.00	0.00	H
ATOM	4609	HG2	LYS	A	321	14.679	16.470	45.226	1.00	0.00	H
ATOM	4610	CD	LYS	A	321	12.828	17.433	45.570	1.00	0.00	C
ATOM	4611	HD1	LYS	A	321	12.407	17.085	44.732	1.00	0.00	H
ATOM	4612	HD2	LYS	A	321	12.516	18.368	45.737	1.00	0.00	H
ATOM	4613	CE	LYS	A	321	12.418	16.552	46.740	1.00	0.00	C
ATOM	4614	HE1	LYS	A	321	11.553	16.875	47.124	1.00	0.00	H
ATOM	4615	HE2	LYS	A	321	13.125	16.573	47.447	1.00	0.00	H
ATOM	4616	NZ	LYS	A	321	12.242	15.161	46.295	1.00	0.00	N
ATOM	4617	HZ1	LYS	A	321	11.973	14.594	47.073	1.00	0.00	H
ATOM	4618	HZ2	LYS	A	321	11.531	15.123	45.593	1.00	0.00	H
ATOM	4619	HZ3	LYS	A	321	13.103	14.821	45.916	1.00	0.00	H
ATOM	4620	C	LYS	A	321	14.585	18.947	41.927	1.00	0.00	C
ATOM	4621	O	LYS	A	321	14.646	20.145	42.239	1.00	0.00	O
ATOM	4622	N	VAL	A	322	14.724	18.559	40.619	1.00	0.00	N
ATOM	4623	H	VAL	A	322	14.627	17.589	40.394	1.00	0.00	H
ATOM	4624	CA	VAL	A	322	15.017	19.531	39.506	1.00	0.00	C
ATOM	4625	HA	VAL	A	322	14.733	20.406	39.898	1.00	0.00	H
ATOM	4626	CB	VAL	A	322	16.492	19.571	39.081	1.00	0.00	C
ATOM	4627	HB	VAL	A	322	16.558	19.985	38.173	1.00	0.00	H
ATOM	4628	CG1	VAL	A	322	17.279	20.296	40.187	1.00	0.00	C
ATOM	4629	HG11	VAL	A	322	18.246	20.337	39.936	1.00	0.00	H
ATOM	4630	HG12	VAL	A	322	16.923	21.224	40.295	1.00	0.00	H
ATOM	4631	HG13	VAL	A	322	17.179	19.798	41.048	1.00	0.00	H
ATOM	4632	CG2	VAL	A	322	17.075	18.228	38.992	1.00	0.00	C
ATOM	4633	HG21	VAL	A	322	18.033	18.298	38.713	1.00	0.00	H
ATOM	4634	HG22	VAL	A	322	17.019	17.781	39.885	1.00	0.00	H
ATOM	4635	HG23	VAL	A	322	16.570	17.690	38.317	1.00	0.00	H
ATOM	4636	C	VAL	A	322	14.272	19.119	38.215	1.00	0.00	C
ATOM	4637	O	VAL	A	322	13.937	17.923	38.124	1.00	0.00	O
ATOM	4638	N	PRO	A	323	13.922	20.095	37.336	1.00	0.00	N
ATOM	4639	CD	PRO	A	323	14.114	21.565	37.380	1.00	0.00	C
ATOM	4640	HD1	PRO	A	323	15.090	21.779	37.337	1.00	0.00	H
ATOM	4641	HD2	PRO	A	323	13.729	21.930	38.228	1.00	0.00	H

ATOM	4642	CG	PRO	A	323	13.340	22.088	36.109	1.00	0.00	C
ATOM	4643	HG1	PRO	A	323	13.954	22.201	35.328	1.00	0.00	H
ATOM	4644	HG2	PRO	A	323	12.874	22.953	36.295	1.00	0.00	H
ATOM	4645	CB	PRO	A	323	12.322	20.993	35.823	1.00	0.00	C
ATOM	4646	HB1	PRO	A	323	12.078	20.983	34.853	1.00	0.00	H
ATOM	4647	HB2	PRO	A	323	11.498	21.125	36.373	1.00	0.00	H
ATOM	4648	CA	PRO	A	323	12.996	19.704	36.201	1.00	0.00	C
ATOM	4649	HA	PRO	A	323	12.345	19.012	36.512	1.00	0.00	H
ATOM	4650	C	PRO	A	323	13.712	19.053	34.982	1.00	0.00	C
ATOM	4651	O	PRO	A	323	14.929	19.009	34.916	1.00	0.00	O
ATOM	4652	N	TYR	A	324	12.871	18.596	34.040	1.00	0.00	N
ATOM	4653	H	TYR	A	324	11.923	18.439	34.317	1.00	0.00	H
ATOM	4654	CA	TYR	A	324	13.216	18.300	32.619	1.00	0.00	C
ATOM	4655	HA	TYR	A	324	13.928	18.945	32.341	1.00	0.00	H
ATOM	4656	CB	TYR	A	324	13.840	16.923	32.456	1.00	0.00	C
ATOM	4657	HB1	TYR	A	324	14.546	16.798	33.153	1.00	0.00	H
ATOM	4658	HB2	TYR	A	324	14.252	16.855	31.547	1.00	0.00	H
ATOM	4659	CG	TYR	A	324	12.838	15.826	32.601	1.00	0.00	C
ATOM	4660	CD1	TYR	A	324	12.424	15.521	33.890	1.00	0.00	C
ATOM	4661	HD1	TYR	A	324	12.728	16.079	34.662	1.00	0.00	H
ATOM	4662	CE1	TYR	A	324	11.566	14.411	34.104	1.00	0.00	C
ATOM	4663	HE1	TYR	A	324	11.365	14.127	35.041	1.00	0.00	H
ATOM	4664	CZ	TYR	A	324	10.998	13.708	33.050	1.00	0.00	C
ATOM	4665	OH	TYR	A	324	10.071	12.680	33.251	1.00	0.00	O
ATOM	4666	HH	TYR	A	324	9.789	12.308	32.367	1.00	0.00	H
ATOM	4667	CE2	TYR	A	324	11.395	13.991	31.718	1.00	0.00	C
ATOM	4668	HE2	TYR	A	324	11.018	13.494	30.936	1.00	0.00	H
ATOM	4669	CD2	TYR	A	324	12.379	15.043	31.558	1.00	0.00	C
ATOM	4670	HD2	TYR	A	324	12.746	15.212	30.643	1.00	0.00	H
ATOM	4671	C	TYR	A	324	11.984	18.518	31.733	1.00	0.00	C
ATOM	4672	O	TYR	A	324	10.804	18.536	32.163	1.00	0.00	O
ATOM	4673	N	ILE	A	325	12.298	18.805	30.420	1.00	0.00	N
ATOM	4674	H	ILE	A	325	13.268	18.860	30.183	1.00	0.00	H
ATOM	4675	CA	ILE	A	325	11.327	19.037	29.339	1.00	0.00	C
ATOM	4676	HA	ILE	A	325	10.415	18.744	29.626	1.00	0.00	H
ATOM	4677	CB	ILE	A	325	11.283	20.623	29.061	1.00	0.00	C
ATOM	4678	HB	ILE	A	325	12.200	20.882	29.364	1.00	0.00	H
ATOM	4679	CG2	ILE	A	325	11.192	21.132	27.635	1.00	0.00	C
ATOM	4680	HG21	ILE	A	325	11.175	22.132	27.638	1.00	0.00	H
ATOM	4681	HG22	ILE	A	325	11.986	20.814	27.116	1.00	0.00	H
ATOM	4682	HG23	ILE	A	325	10.356	20.785	27.210	1.00	0.00	H
ATOM	4683	CG1	ILE	A	325	10.121	21.358	29.810	1.00	0.00	C
ATOM	4684	HG11	ILE	A	325	10.079	20.997	30.742	1.00	0.00	H
ATOM	4685	HG12	ILE	A	325	9.267	21.149	29.333	1.00	0.00	H
ATOM	4686	CD	ILE	A	325	10.240	22.909	29.909	1.00	0.00	C
ATOM	4687	HD1	ILE	A	325	9.451	23.273	30.403	1.00	0.00	H
ATOM	4688	HD2	ILE	A	325	11.079	23.148	30.398	1.00	0.00	H
ATOM	4689	HD3	ILE	A	325	10.267	23.301	28.989	1.00	0.00	H
ATOM	4690	C	ILE	A	325	11.653	18.086	28.141	1.00	0.00	C
ATOM	4691	O	ILE	A	325	12.811	17.584	28.111	1.00	0.00	O
ATOM	4692	N	PHE	A	326	10.752	17.884	27.247	1.00	0.00	N
ATOM	4693	H	PHE	A	326	9.859	18.314	27.377	1.00	0.00	H
ATOM	4694	CA	PHE	A	326	10.953	17.055	26.038	1.00	0.00	C
ATOM	4695	HA	PHE	A	326	11.897	17.197	25.740	1.00	0.00	H

ATOM	4696	CB	PHE	A	326	10.671	15.521	26.446	1.00	0.00	C
ATOM	4697	HB1	PHE	A	326	9.956	15.473	27.143	1.00	0.00	H
ATOM	4698	HB2	PHE	A	326	11.506	15.095	26.795	1.00	0.00	H
ATOM	4699	CG	PHE	A	326	10.203	14.726	25.224	1.00	0.00	C
ATOM	4700	CD1	PHE	A	326	8.878	14.382	24.931	1.00	0.00	C
ATOM	4701	HD1	PHE	A	326	8.159	14.728	25.534	1.00	0.00	H
ATOM	4702	CE1	PHE	A	326	8.489	13.569	23.826	1.00	0.00	C
ATOM	4703	HE1	PHE	A	326	7.536	13.356	23.609	1.00	0.00	H
ATOM	4704	CZ	PHE	A	326	9.596	13.089	23.060	1.00	0.00	C
ATOM	4705	HZ	PHE	A	326	9.395	12.536	22.252	1.00	0.00	H
ATOM	4706	CE2	PHE	A	326	10.935	13.335	23.356	1.00	0.00	C
ATOM	4707	HE2	PHE	A	326	11.654	12.963	22.769	1.00	0.00	H
ATOM	4708	CD2	PHE	A	326	11.276	14.111	24.486	1.00	0.00	C
ATOM	4709	HD2	PHE	A	326	12.229	14.229	24.764	1.00	0.00	H
ATOM	4710	C	PHE	A	326	10.076	17.556	24.851	1.00	0.00	C
ATOM	4711	O	PHE	A	326	8.960	18.053	25.180	1.00	0.00	O
ATOM	4712	N	ARG	A	327	10.583	17.497	23.589	1.00	0.00	N
ATOM	4713	H	ARG	A	327	11.542	17.232	23.492	1.00	0.00	H
ATOM	4714	CA	ARG	A	327	9.822	17.796	22.332	1.00	0.00	C
ATOM	4715	HA	ARG	A	327	8.846	17.689	22.522	1.00	0.00	H
ATOM	4716	CB	ARG	A	327	10.207	19.250	21.946	1.00	0.00	C
ATOM	4717	HB1	ARG	A	327	11.167	19.277	21.667	1.00	0.00	H
ATOM	4718	HB2	ARG	A	327	10.072	19.850	22.734	1.00	0.00	H
ATOM	4719	CG	ARG	A	327	9.325	19.740	20.777	1.00	0.00	C
ATOM	4720	HG1	ARG	A	327	8.391	19.404	20.898	1.00	0.00	H
ATOM	4721	HG2	ARG	A	327	9.693	19.397	19.913	1.00	0.00	H
ATOM	4722	CD	ARG	A	327	9.328	21.290	20.770	1.00	0.00	C
ATOM	4723	HD1	ARG	A	327	9.229	21.633	21.704	1.00	0.00	H
ATOM	4724	HD2	ARG	A	327	8.573	21.628	20.209	1.00	0.00	H
ATOM	4725	NE	ARG	A	327	10.618	21.814	20.202	1.00	0.00	N
ATOM	4726	HE	ARG	A	327	11.305	21.162	19.880	1.00	0.00	H
ATOM	4727	CZ	ARG	A	327	10.874	23.111	20.114	1.00	0.00	C
ATOM	4728	NH1	ARG	A	327	12.044	23.506	19.670	1.00	0.00	N
ATOM	4729	HH11	ARG	A	327	12.731	22.831	19.402	1.00	0.00	H
ATOM	4730	HH12	ARG	A	327	12.248	24.482	19.599	1.00	0.00	H
ATOM	4731	NH2	ARG	A	327	10.035	24.051	20.432	1.00	0.00	N
ATOM	4732	HH21	ARG	A	327	9.124	23.815	20.770	1.00	0.00	H
ATOM	4733	HH22	ARG	A	327	10.300	25.011	20.339	1.00	0.00	H
ATOM	4734	C	ARG	A	327	10.178	16.805	21.169	1.00	0.00	C
ATOM	4735	O	ARG	A	327	11.154	16.985	20.451	1.00	0.00	O
ATOM	4736	N	ASP	A	328	9.315	15.816	20.978	1.00	0.00	N
ATOM	4737	H	ASP	A	328	8.587	15.738	21.659	1.00	0.00	H
ATOM	4738	CA	ASP	A	328	9.287	14.838	19.918	1.00	0.00	C
ATOM	4739	HA	ASP	A	328	8.523	14.273	20.229	1.00	0.00	H
ATOM	4740	CB	ASP	A	328	8.898	15.547	18.628	1.00	0.00	C
ATOM	4741	HB1	ASP	A	328	9.598	16.246	18.482	1.00	0.00	H
ATOM	4742	HB2	ASP	A	328	8.021	15.989	18.817	1.00	0.00	H
ATOM	4743	CG	ASP	A	328	8.732	14.817	17.284	1.00	0.00	C
ATOM	4744	OD1	ASP	A	328	8.567	15.520	16.292	1.00	0.00	O
ATOM	4745	OD2	ASP	A	328	8.661	13.581	17.137	1.00	0.00	O
ATOM	4746	C	ASP	A	328	10.449	13.743	19.822	1.00	0.00	C
ATOM	4747	O	ASP	A	328	10.150	12.547	19.843	1.00	0.00	O
ATOM	4748	N	ASN	A	329	11.695	14.227	19.959	1.00	0.00	N
ATOM	4749	H	ASN	A	329	11.828	15.216	19.890	1.00	0.00	H

ATOM	4750	CA	ASN	A	329	12.877	13.379	20.206	1.00	0.00	C
ATOM	4751	HA	ASN	A	329	12.563	12.578	20.715	1.00	0.00	H
ATOM	4752	CB	ASN	A	329	13.452	12.932	18.858	1.00	0.00	C
ATOM	4753	HB1	ASN	A	329	12.834	12.254	18.460	1.00	0.00	H
ATOM	4754	HB2	ASN	A	329	14.346	12.514	19.019	1.00	0.00	H
ATOM	4755	CG	ASN	A	329	13.638	14.030	17.845	1.00	0.00	C
ATOM	4756	OD1	ASN	A	329	14.667	14.668	17.847	1.00	0.00	O
ATOM	4757	ND2	ASN	A	329	12.793	14.371	16.909	1.00	0.00	N
ATOM	4758	HD21	ASN	A	329	11.925	13.884	16.818	1.00	0.00	H
ATOM	4759	HD22	ASN	A	329	13.015	15.119	16.284	1.00	0.00	H
ATOM	4760	C	ASN	A	329	13.886	14.002	21.150	1.00	0.00	C
ATOM	4761	O	ASN	A	329	14.673	13.258	21.692	1.00	0.00	O
ATOM	4762	N	GLN	A	330	13.811	15.313	21.378	1.00	0.00	N
ATOM	4763	H	GLN	A	330	13.046	15.842	21.010	1.00	0.00	H
ATOM	4764	CA	GLN	A	330	14.859	15.985	22.175	1.00	0.00	C
ATOM	4765	HA	GLN	A	330	15.661	15.389	22.219	1.00	0.00	H
ATOM	4766	CB	GLN	A	330	15.246	17.262	21.443	1.00	0.00	C
ATOM	4767	HB1	GLN	A	330	14.635	17.996	21.739	1.00	0.00	H
ATOM	4768	HB2	GLN	A	330	15.135	17.112	20.461	1.00	0.00	H
ATOM	4769	CG	GLN	A	330	16.681	17.691	21.708	1.00	0.00	C
ATOM	4770	HG1	GLN	A	330	17.304	16.963	21.422	1.00	0.00	H
ATOM	4771	HG2	GLN	A	330	16.800	17.868	22.685	1.00	0.00	H
ATOM	4772	CD	GLN	A	330	16.982	18.962	20.917	1.00	0.00	C
ATOM	4773	OE1	GLN	A	330	16.133	19.772	20.685	1.00	0.00	O
ATOM	4774	NE2	GLN	A	330	18.253	19.087	20.559	1.00	0.00	N
ATOM	4775	HE21	GLN	A	330	18.915	18.381	20.811	1.00	0.00	H
ATOM	4776	HE22	GLN	A	330	18.549	19.887	20.037	1.00	0.00	H
ATOM	4777	C	GLN	A	330	14.421	16.196	23.644	1.00	0.00	C
ATOM	4778	O	GLN	A	330	13.348	16.774	23.835	1.00	0.00	O
ATOM	4779	N	TRP	A	331	15.329	15.869	24.576	1.00	0.00	N
ATOM	4780	H	TRP	A	331	16.211	15.546	24.234	1.00	0.00	H
ATOM	4781	CA	TRP	A	331	15.191	15.925	25.997	1.00	0.00	C
ATOM	4782	HA	TRP	A	331	14.268	16.233	26.230	1.00	0.00	H
ATOM	4783	CB	TRP	A	331	15.611	14.453	26.463	1.00	0.00	C
ATOM	4784	HB1	TRP	A	331	16.606	14.362	26.414	1.00	0.00	H
ATOM	4785	HB2	TRP	A	331	15.184	13.780	25.859	1.00	0.00	H
ATOM	4786	CG	TRP	A	331	15.213	14.082	27.871	1.00	0.00	C
ATOM	4787	CD1	TRP	A	331	14.154	13.309	28.109	1.00	0.00	C
ATOM	4788	HD1	TRP	A	331	13.524	12.930	27.431	1.00	0.00	H
ATOM	4789	NE1	TRP	A	331	14.098	13.134	29.481	1.00	0.00	N
ATOM	4790	HE1	TRP	A	331	13.380	12.613	29.942	1.00	0.00	H
ATOM	4791	CE2	TRP	A	331	15.122	13.745	30.138	1.00	0.00	C
ATOM	4792	CZ2	TRP	A	331	15.445	13.766	31.478	1.00	0.00	C
ATOM	4793	HZ2	TRP	A	331	14.871	13.273	32.131	1.00	0.00	H
ATOM	4794	CH2	TRP	A	331	16.593	14.485	31.948	1.00	0.00	C
ATOM	4795	HH2	TRP	A	331	16.808	14.558	32.922	1.00	0.00	H
ATOM	4796	CZ3	TRP	A	331	17.394	15.078	30.969	1.00	0.00	C
ATOM	4797	HZ3	TRP	A	331	18.238	15.530	31.258	1.00	0.00	H
ATOM	4798	CE3	TRP	A	331	17.074	15.074	29.584	1.00	0.00	C
ATOM	4799	HE3	TRP	A	331	17.654	15.551	28.924	1.00	0.00	H
ATOM	4800	CD2	TRP	A	331	15.879	14.358	29.147	1.00	0.00	C
ATOM	4801	C	TRP	A	331	16.160	16.944	26.567	1.00	0.00	C
ATOM	4802	O	TRP	A	331	17.294	16.993	26.148	1.00	0.00	O
ATOM	4803	N	VAL	A	332	15.788	17.753	27.602	1.00	0.00	N

ATOM	4804	H	VAL	A	332	14.863	17.624	27.960	1.00	0.00	H
ATOM	4805	CA	VAL	A	332	16.586	18.771	28.232	1.00	0.00	C
ATOM	4806	HA	VAL	A	332	17.530	18.475	28.085	1.00	0.00	H
ATOM	4807	CB	VAL	A	332	16.443	20.138	27.575	1.00	0.00	C
ATOM	4808	HB	VAL	A	332	16.792	19.992	26.649	1.00	0.00	H
ATOM	4809	CG1	VAL	A	332	14.953	20.652	27.449	1.00	0.00	C
ATOM	4810	HG11	VAL	A	332	14.946	21.550	27.010	1.00	0.00	H
ATOM	4811	HG12	VAL	A	332	14.424	20.007	26.897	1.00	0.00	H
ATOM	4812	HG13	VAL	A	332	14.547	20.723	28.360	1.00	0.00	H
ATOM	4813	CG2	VAL	A	332	17.319	21.188	28.295	1.00	0.00	C
ATOM	4814	HG21	VAL	A	332	17.210	22.076	27.848	1.00	0.00	H
ATOM	4815	HG22	VAL	A	332	17.036	21.259	29.251	1.00	0.00	H
ATOM	4816	HG23	VAL	A	332	18.278	20.908	28.251	1.00	0.00	H
ATOM	4817	C	VAL	A	332	16.394	18.777	29.695	1.00	0.00	C
ATOM	4818	O	VAL	A	332	15.281	18.919	30.191	1.00	0.00	O
ATOM	4819	N	GLY	A	333	17.495	18.717	30.411	1.00	0.00	N
ATOM	4820	H	GLY	A	333	18.331	18.578	29.880	1.00	0.00	H
ATOM	4821	CA	GLY	A	333	17.701	18.820	31.865	1.00	0.00	C
ATOM	4822	HA1	GLY	A	333	18.341	18.101	32.135	1.00	0.00	H
ATOM	4823	HA2	GLY	A	333	16.819	18.666	32.311	1.00	0.00	H
ATOM	4824	C	GLY	A	333	18.255	20.181	32.287	1.00	0.00	C
ATOM	4825	O	GLY	A	333	19.448	20.432	32.151	1.00	0.00	O
ATOM	4826	N	PHE	A	334	17.395	21.014	32.919	1.00	0.00	N
ATOM	4827	H	PHE	A	334	16.555	20.614	33.285	1.00	0.00	H
ATOM	4828	CA	PHE	A	334	17.592	22.451	33.112	1.00	0.00	C
ATOM	4829	HA	PHE	A	334	18.582	22.573	33.040	1.00	0.00	H
ATOM	4830	CB	PHE	A	334	16.921	23.254	32.013	1.00	0.00	C
ATOM	4831	HB1	PHE	A	334	17.116	22.772	31.159	1.00	0.00	H
ATOM	4832	HB2	PHE	A	334	17.375	24.145	32.004	1.00	0.00	H
ATOM	4833	CG	PHE	A	334	15.348	23.490	32.112	1.00	0.00	C
ATOM	4834	CD1	PHE	A	334	14.465	22.386	31.831	1.00	0.00	C
ATOM	4835	HD1	PHE	A	334	14.845	21.562	31.412	1.00	0.00	H
ATOM	4836	CE1	PHE	A	334	13.104	22.422	32.120	1.00	0.00	C
ATOM	4837	HE1	PHE	A	334	12.501	21.638	31.975	1.00	0.00	H
ATOM	4838	CZ	PHE	A	334	12.639	23.663	32.638	1.00	0.00	C
ATOM	4839	HZ	PHE	A	334	11.676	23.704	32.906	1.00	0.00	H
ATOM	4840	CE2	PHE	A	334	13.389	24.832	32.812	1.00	0.00	C
ATOM	4841	HE2	PHE	A	334	12.984	25.687	33.137	1.00	0.00	H
ATOM	4842	CD2	PHE	A	334	14.788	24.712	32.487	1.00	0.00	C
ATOM	4843	HD2	PHE	A	334	15.371	25.523	32.531	1.00	0.00	H
ATOM	4844	C	PHE	A	334	17.234	22.934	34.493	1.00	0.00	C
ATOM	4845	O	PHE	A	334	16.470	22.277	35.183	1.00	0.00	O
ATOM	4846	N	ASP	A	335	17.653	24.127	34.938	1.00	0.00	N
ATOM	4847	H	ASP	A	335	18.362	24.603	34.417	1.00	0.00	H
ATOM	4848	CA	ASP	A	335	17.116	24.789	36.177	1.00	0.00	C
ATOM	4849	HA	ASP	A	335	16.662	24.043	36.664	1.00	0.00	H
ATOM	4850	CB	ASP	A	335	18.253	25.421	37.072	1.00	0.00	C
ATOM	4851	HB1	ASP	A	335	17.858	26.087	37.705	1.00	0.00	H
ATOM	4852	HB2	ASP	A	335	18.932	25.872	36.493	1.00	0.00	H
ATOM	4853	CG	ASP	A	335	18.964	24.377	37.887	1.00	0.00	C
ATOM	4854	OD1	ASP	A	335	18.283	23.624	38.655	1.00	0.00	O
ATOM	4855	OD2	ASP	A	335	20.224	24.373	37.987	1.00	0.00	O
ATOM	4856	C	ASP	A	335	16.108	25.913	35.807	1.00	0.00	C
ATOM	4857	O	ASP	A	335	16.182	26.605	34.796	1.00	0.00	O

ATOM	4858	N	ASP	A	336	15.100	25.966	36.722	1.00	0.00	N
ATOM	4859	H	ASP	A	336	15.130	25.309	37.475	1.00	0.00	H
ATOM	4860	CA	ASP	A	336	13.984	26.899	36.695	1.00	0.00	C
ATOM	4861	HA	ASP	A	336	14.239	27.476	35.919	1.00	0.00	H
ATOM	4862	CB	ASP	A	336	12.608	26.174	36.454	1.00	0.00	C
ATOM	4863	HB1	ASP	A	336	12.715	25.557	35.675	1.00	0.00	H
ATOM	4864	HB2	ASP	A	336	11.925	26.871	36.236	1.00	0.00	H
ATOM	4865	CG	ASP	A	336	12.030	25.339	37.595	1.00	0.00	C
ATOM	4866	OD1	ASP	A	336	12.457	25.441	38.759	1.00	0.00	O
ATOM	4867	OD2	ASP	A	336	10.895	24.878	37.360	1.00	0.00	O
ATOM	4868	C	ASP	A	336	13.882	27.737	38.013	1.00	0.00	C
ATOM	4869	O	ASP	A	336	14.693	27.596	38.923	1.00	0.00	O
ATOM	4870	N	VAL	A	337	12.850	28.566	38.069	1.00	0.00	N
ATOM	4871	H	VAL	A	337	12.187	28.544	37.321	1.00	0.00	H
ATOM	4872	CA	VAL	A	337	12.620	29.533	39.187	1.00	0.00	C
ATOM	4873	HA	VAL	A	337	13.509	29.989	39.159	1.00	0.00	H
ATOM	4874	CB	VAL	A	337	11.427	30.568	39.003	1.00	0.00	C
ATOM	4875	HB	VAL	A	337	11.291	31.196	39.769	1.00	0.00	H
ATOM	4876	CG1	VAL	A	337	11.740	31.320	37.745	1.00	0.00	C
ATOM	4877	HG11	VAL	A	337	11.021	31.993	37.571	1.00	0.00	H
ATOM	4878	HG12	VAL	A	337	12.618	31.788	37.846	1.00	0.00	H
ATOM	4879	HG13	VAL	A	337	11.790	30.680	36.978	1.00	0.00	H
ATOM	4880	CG2	VAL	A	337	10.148	29.805	38.800	1.00	0.00	C
ATOM	4881	HG21	VAL	A	337	9.391	30.448	38.683	1.00	0.00	H
ATOM	4882	HG22	VAL	A	337	10.228	29.232	37.984	1.00	0.00	H
ATOM	4883	HG23	VAL	A	337	9.974	29.227	39.597	1.00	0.00	H
ATOM	4884	C	VAL	A	337	12.456	28.887	40.525	1.00	0.00	C
ATOM	4885	O	VAL	A	337	12.997	29.446	41.430	1.00	0.00	O
ATOM	4886	N	GLU	A	338	11.673	27.780	40.647	1.00	0.00	N
ATOM	4887	H	GLU	A	338	11.164	27.487	39.837	1.00	0.00	H
ATOM	4888	CA	GLU	A	338	11.513	26.980	41.868	1.00	0.00	C
ATOM	4889	HA	GLU	A	338	11.205	27.677	42.515	1.00	0.00	H
ATOM	4890	CB	GLU	A	338	10.452	25.902	41.758	1.00	0.00	C
ATOM	4891	HB1	GLU	A	338	10.140	25.723	42.691	1.00	0.00	H
ATOM	4892	HB2	GLU	A	338	10.916	25.092	41.400	1.00	0.00	H
ATOM	4893	CG	GLU	A	338	9.193	26.113	40.899	1.00	0.00	C
ATOM	4894	HG1	GLU	A	338	8.631	25.288	40.964	1.00	0.00	H
ATOM	4895	HG2	GLU	A	338	9.483	26.246	39.951	1.00	0.00	H
ATOM	4896	CD	GLU	A	338	8.307	27.272	41.250	1.00	0.00	C
ATOM	4897	OE1	GLU	A	338	7.371	27.489	40.433	1.00	0.00	O
ATOM	4898	OE2	GLU	A	338	8.597	28.213	42.020	1.00	0.00	O
ATOM	4899	C	GLU	A	338	12.841	26.292	42.302	1.00	0.00	C
ATOM	4900	O	GLU	A	338	13.098	26.291	43.504	1.00	0.00	O
ATOM	4901	N	SER	A	339	13.622	25.775	41.344	1.00	0.00	N
ATOM	4902	H	SER	A	339	13.287	25.860	40.406	1.00	0.00	H
ATOM	4903	CA	SER	A	339	14.875	25.118	41.509	1.00	0.00	C
ATOM	4904	HA	SER	A	339	14.657	24.382	42.149	1.00	0.00	H
ATOM	4905	CB	SER	A	339	15.362	24.523	40.198	1.00	0.00	C
ATOM	4906	HB1	SER	A	339	15.678	25.234	39.570	1.00	0.00	H
ATOM	4907	HB2	SER	A	339	14.648	23.981	39.755	1.00	0.00	H
ATOM	4908	OG	SER	A	339	16.452	23.673	40.511	1.00	0.00	O
ATOM	4909	HG	SER	A	339	16.804	23.259	39.671	1.00	0.00	H
ATOM	4910	C	SER	A	339	15.943	26.076	42.098	1.00	0.00	C
ATOM	4911	O	SER	A	339	16.560	25.714	43.097	1.00	0.00	O

```
ATOM   4912  N   PHE A 340      16.057  27.271  41.485  1.00  0.00           N
ATOM   4913  H   PHE A 340      15.545  27.432  40.641  1.00  0.00           H
ATOM   4914  CA  PHE A 340      16.917  28.357  42.021  1.00  0.00           C
ATOM   4915  HA  PHE A 340      17.878  28.082  42.008  1.00  0.00           H
ATOM   4916  CB  PHE A 340      16.665  29.628  41.083  1.00  0.00           C
ATOM   4917  HB1 PHE A 340      16.695  30.462  41.634  1.00  0.00           H
ATOM   4918  HB2 PHE A 340      15.769  29.549  40.646  1.00  0.00           H
ATOM   4919  CG  PHE A 340      17.698  29.780  39.975  1.00  0.00           C
ATOM   4920  CD1 PHE A 340      18.026  28.728  39.092  1.00  0.00           C
ATOM   4921  HD1 PHE A 340      17.521  27.868  39.168  1.00  0.00           H
ATOM   4922  CE1 PHE A 340      19.021  28.831  38.119  1.00  0.00           C
ATOM   4923  HE1 PHE A 340      19.205  28.079  37.487  1.00  0.00           H
ATOM   4924  CZ  PHE A 340      19.754  30.026  38.058  1.00  0.00           C
ATOM   4925  HZ  PHE A 340      20.549  30.089  37.455  1.00  0.00           H
ATOM   4926  CE2 PHE A 340      19.382  31.133  38.835  1.00  0.00           C
ATOM   4927  HE2 PHE A 340      19.833  32.014  38.691  1.00  0.00           H
ATOM   4928  CD2 PHE A 340      18.370  31.013  39.825  1.00  0.00           C
ATOM   4929  HD2 PHE A 340      18.137  31.791  40.408  1.00  0.00           H
ATOM   4930  C   PHE A 340      16.571  28.589  43.515  1.00  0.00           C
ATOM   4931  O   PHE A 340      17.453  28.580  44.367  1.00  0.00           O
ATOM   4932  N   LYS A 341      15.240  28.746  43.823  1.00  0.00           N
ATOM   4933  H   LYS A 341      14.566  28.754  43.084  1.00  0.00           H
ATOM   4934  CA  LYS A 341      14.782  28.903  45.214  1.00  0.00           C
ATOM   4935  HA  LYS A 341      15.220  29.661  45.698  1.00  0.00           H
ATOM   4936  CB  LYS A 341      13.237  29.236  45.091  1.00  0.00           C
ATOM   4937  HB1 LYS A 341      12.772  28.518  44.573  1.00  0.00           H
ATOM   4938  HB2 LYS A 341      13.112  30.114  44.629  1.00  0.00           H
ATOM   4939  CG  LYS A 341      12.610  29.320  46.491  1.00  0.00           C
ATOM   4940  HG1 LYS A 341      13.271  29.717  47.128  1.00  0.00           H
ATOM   4941  HG2 LYS A 341      12.361  28.402  46.799  1.00  0.00           H
ATOM   4942  CD  LYS A 341      11.377  30.171  46.499  1.00  0.00           C
ATOM   4943  HD1 LYS A 341      10.851  29.919  45.687  1.00  0.00           H
ATOM   4944  HD2 LYS A 341      11.684  31.119  46.416  1.00  0.00           H
ATOM   4945  CE  LYS A 341      10.435  30.097  47.696  1.00  0.00           C
ATOM   4946  HE1 LYS A 341      10.011  29.192  47.728  1.00  0.00           H
ATOM   4947  HE2 LYS A 341       9.725  30.794  47.602  1.00  0.00           H
ATOM   4948  NZ  LYS A 341      11.086  30.315  48.980  1.00  0.00           N
ATOM   4949  HZ1 LYS A 341      10.408  30.252  49.712  1.00  0.00           H
ATOM   4950  HZ2 LYS A 341      11.792  29.620  49.118  1.00  0.00           H
ATOM   4951  HZ3 LYS A 341      11.505  31.223  48.991  1.00  0.00           H
ATOM   4952  C   LYS A 341      15.276  27.701  46.073  1.00  0.00           C
ATOM   4953  O   LYS A 341      15.898  27.967  47.130  1.00  0.00           O
ATOM   4954  N   THR A 342      14.992  26.496  45.609  1.00  0.00           N
ATOM   4955  H   THR A 342      14.556  26.440  44.711  1.00  0.00           H
ATOM   4956  CA  THR A 342      15.266  25.230  46.309  1.00  0.00           C
ATOM   4957  HA  THR A 342      14.694  25.238  47.129  1.00  0.00           H
ATOM   4958  CB  THR A 342      14.923  24.039  45.404  1.00  0.00           C
ATOM   4959  HB  THR A 342      15.556  24.123  44.635  1.00  0.00           H
ATOM   4960  CG2 THR A 342      14.994  22.607  45.962  1.00  0.00           C
ATOM   4961 HG21 THR A 342      14.741  21.957  45.245  1.00  0.00           H
ATOM   4962 HG22 THR A 342      15.925  22.416  46.272  1.00  0.00           H
ATOM   4963 HG23 THR A 342      14.361  22.517  46.731  1.00  0.00           H
ATOM   4964  OG1 THR A 342      13.570  24.156  45.082  1.00  0.00           O
ATOM   4965  HG1 THR A 342      13.301  23.396  44.490  1.00  0.00           H
```

ATOM	4966	C	THR	A	342	16.747	25.143	46.674	1.00	0.00	C
ATOM	4967	O	THR	A	342	17.162	24.875	47.818	1.00	0.00	O
ATOM	4968	N	LYS	A	343	17.601	25.435	45.746	1.00	0.00	N
ATOM	4969	H	LYS	A	343	17.226	25.587	44.831	1.00	0.00	H
ATOM	4970	CA	LYS	A	343	19.085	25.571	45.885	1.00	0.00	C
ATOM	4971	HA	LYS	A	343	19.353	24.716	46.329	1.00	0.00	H
ATOM	4972	CB	LYS	A	343	19.700	25.809	44.466	1.00	0.00	C
ATOM	4973	HB1	LYS	A	343	20.689	25.940	44.531	1.00	0.00	H
ATOM	4974	HB2	LYS	A	343	19.285	26.610	44.035	1.00	0.00	H
ATOM	4975	CG	LYS	A	343	19.392	24.549	43.635	1.00	0.00	C
ATOM	4976	HG1	LYS	A	343	18.399	24.439	43.594	1.00	0.00	H
ATOM	4977	HG2	LYS	A	343	19.795	23.762	44.102	1.00	0.00	H
ATOM	4978	CD	LYS	A	343	19.933	24.580	42.191	1.00	0.00	C
ATOM	4979	HD1	LYS	A	343	20.888	24.877	42.202	1.00	0.00	H
ATOM	4980	HD2	LYS	A	343	19.391	25.222	41.649	1.00	0.00	H
ATOM	4981	CE	LYS	A	343	19.868	23.232	41.527	1.00	0.00	C
ATOM	4982	HE1	LYS	A	343	18.934	23.031	41.233	1.00	0.00	H
ATOM	4983	HE2	LYS	A	343	20.181	22.518	42.153	1.00	0.00	H
ATOM	4984	NZ	LYS	A	343	20.742	23.267	40.362	1.00	0.00	N
ATOM	4985	HZ1	LYS	A	343	20.718	22.380	39.900	1.00	0.00	H
ATOM	4986	HZ2	LYS	A	343	20.430	23.979	39.733	1.00	0.00	H
ATOM	4987	HZ3	LYS	A	343	21.678	23.466	40.653	1.00	0.00	H
ATOM	4988	C	LYS	A	343	19.543	26.781	46.761	1.00	0.00	C
ATOM	4989	O	LYS	A	343	20.487	26.552	47.505	1.00	0.00	O
ATOM	4990	N	VAL	A	344	18.787	27.888	46.792	1.00	0.00	N
ATOM	4991	H	VAL	A	344	17.969	27.956	46.221	1.00	0.00	H
ATOM	4992	CA	VAL	A	344	19.177	29.022	47.688	1.00	0.00	C
ATOM	4993	HA	VAL	A	344	20.155	29.089	47.489	1.00	0.00	H
ATOM	4994	CB	VAL	A	344	18.676	30.467	47.386	1.00	0.00	C
ATOM	4995	HB	VAL	A	344	17.676	30.444	47.397	1.00	0.00	H
ATOM	4996	CG1	VAL	A	344	19.093	31.503	48.388	1.00	0.00	C
ATOM	4997	HG11	VAL	A	344	18.730	32.394	48.117	1.00	0.00	H
ATOM	4998	HG12	VAL	A	344	18.736	31.258	49.289	1.00	0.00	H
ATOM	4999	HG13	VAL	A	344	20.091	31.547	48.426	1.00	0.00	H
ATOM	5000	CG2	VAL	A	344	19.250	30.911	46.065	1.00	0.00	C
ATOM	5001	HG21	VAL	A	344	18.936	31.838	45.860	1.00	0.00	H
ATOM	5002	HG22	VAL	A	344	20.249	30.901	46.115	1.00	0.00	H
ATOM	5003	HG23	VAL	A	344	18.947	30.289	45.344	1.00	0.00	H
ATOM	5004	C	VAL	A	344	18.795	28.624	49.145	1.00	0.00	C
ATOM	5005	O	VAL	A	344	19.476	29.126	50.068	1.00	0.00	O
ATOM	5006	N	SER	A	345	17.734	27.892	49.317	1.00	0.00	N
ATOM	5007	H	SER	A	345	17.157	27.667	48.532	1.00	0.00	H
ATOM	5008	CA	SER	A	345	17.369	27.386	50.665	1.00	0.00	C
ATOM	5009	HA	SER	A	345	17.346	28.227	51.205	1.00	0.00	H
ATOM	5010	CB	SER	A	345	15.954	26.749	50.636	1.00	0.00	C
ATOM	5011	HB1	SER	A	345	15.905	26.029	49.944	1.00	0.00	H
ATOM	5012	HB2	SER	A	345	15.259	27.442	50.445	1.00	0.00	H
ATOM	5013	OG	SER	A	345	15.578	26.150	51.840	1.00	0.00	O
ATOM	5014	HG	SER	A	345	14.661	25.763	51.748	1.00	0.00	H
ATOM	5015	C	SER	A	345	18.450	26.362	51.300	1.00	0.00	C
ATOM	5016	O	SER	A	345	18.882	26.459	52.477	1.00	0.00	O
ATOM	5017	N	TYR	A	346	18.860	25.365	50.511	1.00	0.00	N
ATOM	5018	H	TYR	A	346	18.341	25.205	49.671	1.00	0.00	H
ATOM	5019	CA	TYR	A	346	20.031	24.464	50.774	1.00	0.00	C

ATOM	5020	HA	TYR	A	346	19.777	24.010	51.628	1.00	0.00	H
ATOM	5021	CB	TYR	A	346	20.087	23.367	49.718	1.00	0.00	C
ATOM	5022	HB1	TYR	A	346	20.181	23.783	48.814	1.00	0.00	H
ATOM	5023	HB2	TYR	A	346	19.243	22.831	49.752	1.00	0.00	H
ATOM	5024	CG	TYR	A	346	21.236	22.444	49.930	1.00	0.00	C
ATOM	5025	CD1	TYR	A	346	21.088	21.237	50.619	1.00	0.00	C
ATOM	5026	HD1	TYR	A	346	20.183	21.003	50.974	1.00	0.00	H
ATOM	5027	CE1	TYR	A	346	22.125	20.349	50.837	1.00	0.00	C
ATOM	5028	HE1	TYR	A	346	21.991	19.491	51.334	1.00	0.00	H
ATOM	5029	CZ	TYR	A	346	23.353	20.712	50.332	1.00	0.00	C
ATOM	5030	OH	TYR	A	346	24.403	19.946	50.455	1.00	0.00	O
ATOM	5031	HH	TYR	A	346	25.190	20.387	50.024	1.00	0.00	H
ATOM	5032	CE2	TYR	A	346	23.529	21.861	49.568	1.00	0.00	C
ATOM	5033	HE2	TYR	A	346	24.415	22.036	49.140	1.00	0.00	H
ATOM	5034	CD2	TYR	A	346	22.465	22.793	49.386	1.00	0.00	C
ATOM	5035	HD2	TYR	A	346	22.597	23.653	48.893	1.00	0.00	H
ATOM	5036	C	TYR	A	346	21.459	25.219	51.040	1.00	0.00	C
ATOM	5037	O	TYR	A	346	22.264	24.898	51.925	1.00	0.00	O
ATOM	5038	N	LEU	A	347	21.595	26.307	50.267	1.00	0.00	N
ATOM	5039	H	LEU	A	347	20.880	26.515	49.600	1.00	0.00	H
ATOM	5040	CA	LEU	A	347	22.735	27.191	50.361	1.00	0.00	C
ATOM	5041	HA	LEU	A	347	23.544	26.604	50.317	1.00	0.00	H
ATOM	5042	CB	LEU	A	347	22.719	28.166	49.125	1.00	0.00	C
ATOM	5043	HB1	LEU	A	347	21.894	28.726	49.200	1.00	0.00	H
ATOM	5044	HB2	LEU	A	347	22.664	27.600	48.302	1.00	0.00	H
ATOM	5045	CG	LEU	A	347	23.786	29.149	48.808	1.00	0.00	C
ATOM	5046	HG	LEU	A	347	24.613	28.605	48.950	1.00	0.00	H
ATOM	5047	CD1	LEU	A	347	23.749	29.670	47.348	1.00	0.00	C
ATOM	5048	HD11	LEU	A	347	24.492	30.324	47.209	1.00	0.00	H
ATOM	5049	HD12	LEU	A	347	23.858	28.902	46.717	1.00	0.00	H
ATOM	5050	HD13	LEU	A	347	22.873	30.120	47.177	1.00	0.00	H
ATOM	5051	CD2	LEU	A	347	23.700	30.394	49.746	1.00	0.00	C
ATOM	5052	HD21	LEU	A	347	24.428	31.039	49.512	1.00	0.00	H
ATOM	5053	HD22	LEU	A	347	22.811	30.838	49.630	1.00	0.00	H
ATOM	5054	HD23	LEU	A	347	23.806	30.103	50.697	1.00	0.00	H
ATOM	5055	C	LEU	A	347	22.881	27.833	51.746	1.00	0.00	C
ATOM	5056	O	LEU	A	347	23.995	27.916	52.226	1.00	0.00	O
ATOM	5057	N	LYS	A	348	21.742	28.344	52.221	1.00	0.00	N
ATOM	5058	H	LYS	A	348	20.924	28.311	51.646	1.00	0.00	H
ATOM	5059	CA	LYS	A	348	21.645	28.938	53.521	1.00	0.00	C
ATOM	5060	HA	LYS	A	348	22.470	29.491	53.636	1.00	0.00	H
ATOM	5061	CB	LYS	A	348	20.345	29.777	53.557	1.00	0.00	C
ATOM	5062	HB1	LYS	A	348	20.171	29.995	54.517	1.00	0.00	H
ATOM	5063	HB2	LYS	A	348	19.614	29.189	53.211	1.00	0.00	H
ATOM	5064	CG	LYS	A	348	20.294	31.126	52.746	1.00	0.00	C
ATOM	5065	HG1	LYS	A	348	20.492	30.922	51.787	1.00	0.00	H
ATOM	5066	HG2	LYS	A	348	20.998	31.736	53.110	1.00	0.00	H
ATOM	5067	CD	LYS	A	348	18.906	31.875	52.816	1.00	0.00	C
ATOM	5068	HD1	LYS	A	348	18.990	32.775	52.389	1.00	0.00	H
ATOM	5069	HD2	LYS	A	348	18.633	31.984	53.772	1.00	0.00	H
ATOM	5070	CE	LYS	A	348	17.796	31.140	52.109	1.00	0.00	C
ATOM	5071	HE1	LYS	A	348	17.563	30.317	52.627	1.00	0.00	H
ATOM	5072	HE2	LYS	A	348	18.102	30.882	51.193	1.00	0.00	H
ATOM	5073	NZ	LYS	A	348	16.576	32.019	51.997	1.00	0.00	N

ATOM	5074	HZ1	LYS	A	348	15.848	31.520	51.527	1.00	0.00	H
ATOM	5075	HZ2	LYS	A	348	16.266	32.277	52.912	1.00	0.00	H
ATOM	5076	HZ3	LYS	A	348	16.805	32.843	51.478	1.00	0.00	H
ATOM	5077	C	LYS	A	348	21.702	27.925	54.625	1.00	0.00	C
ATOM	5078	O	LYS	A	348	22.351	28.153	55.650	1.00	0.00	O
ATOM	5079	N	GLN	A	349	21.084	26.699	54.389	1.00	0.00	N
ATOM	5080	H	GLN	A	349	20.524	26.604	53.566	1.00	0.00	H
ATOM	5081	CA	GLN	A	349	21.203	25.579	55.248	1.00	0.00	C
ATOM	5082	HA	GLN	A	349	20.812	26.011	56.061	1.00	0.00	H
ATOM	5083	CB	GLN	A	349	20.332	24.413	54.751	1.00	0.00	C
ATOM	5084	HB1	GLN	A	349	20.673	24.122	53.857	1.00	0.00	H
ATOM	5085	HB2	GLN	A	349	19.391	24.738	54.659	1.00	0.00	H
ATOM	5086	CG	GLN	A	349	20.358	23.197	55.728	1.00	0.00	C
ATOM	5087	HG1	GLN	A	349	19.432	22.819	55.756	1.00	0.00	H
ATOM	5088	HG2	GLN	A	349	20.602	23.548	56.632	1.00	0.00	H
ATOM	5089	CD	GLN	A	349	21.318	22.017	55.429	1.00	0.00	C
ATOM	5090	OE1	GLN	A	349	21.161	21.333	54.400	1.00	0.00	O
ATOM	5091	NE2	GLN	A	349	22.263	21.714	56.300	1.00	0.00	N
ATOM	5092	HE21	GLN	A	349	22.354	22.244	57.143	1.00	0.00	H
ATOM	5093	HE22	GLN	A	349	22.887	20.955	56.116	1.00	0.00	H
ATOM	5094	C	GLN	A	349	22.636	25.135	55.683	1.00	0.00	C
ATOM	5095	O	GLN	A	349	22.907	24.913	56.840	1.00	0.00	O
ATOM	5096	N	LYS	A	350	23.496	24.978	54.671	1.00	0.00	N
ATOM	5097	H	LYS	A	350	23.142	25.063	53.740	1.00	0.00	H
ATOM	5098	CA	LYS	A	350	24.939	24.687	54.839	1.00	0.00	C
ATOM	5099	HA	LYS	A	350	25.101	24.078	55.615	1.00	0.00	H
ATOM	5100	CB	LYS	A	350	25.330	24.153	53.452	1.00	0.00	C
ATOM	5101	HB1	LYS	A	350	26.325	24.220	53.379	1.00	0.00	H
ATOM	5102	HB2	LYS	A	350	24.905	24.752	52.773	1.00	0.00	H
ATOM	5103	CG	LYS	A	350	24.946	22.714	53.088	1.00	0.00	C
ATOM	5104	HG1	LYS	A	350	25.288	22.541	52.164	1.00	0.00	H
ATOM	5105	HG2	LYS	A	350	23.947	22.665	53.089	1.00	0.00	H
ATOM	5106	CD	LYS	A	350	25.506	21.593	54.050	1.00	0.00	C
ATOM	5107	HD1	LYS	A	350	25.057	21.628	54.943	1.00	0.00	H
ATOM	5108	HD2	LYS	A	350	26.495	21.684	54.170	1.00	0.00	H
ATOM	5109	CE	LYS	A	350	25.217	20.274	53.418	1.00	0.00	C
ATOM	5110	HE1	LYS	A	350	25.805	20.167	52.616	1.00	0.00	H
ATOM	5111	HE2	LYS	A	350	24.259	20.258	53.133	1.00	0.00	H
ATOM	5112	NZ	LYS	A	350	25.470	19.135	54.375	1.00	0.00	N
ATOM	5113	HZ1	LYS	A	350	25.267	18.267	53.921	1.00	0.00	H
ATOM	5114	HZ2	LYS	A	350	26.428	19.146	54.660	1.00	0.00	H
ATOM	5115	HZ3	LYS	A	350	24.882	19.237	55.177	1.00	0.00	H
ATOM	5116	C	LYS	A	350	25.729	25.914	55.141	1.00	0.00	C
ATOM	5117	O	LYS	A	350	26.858	25.759	55.754	1.00	0.00	O
ATOM	5118	N	GLY	A	351	25.305	27.160	54.859	1.00	0.00	N
ATOM	5119	H	GLY	A	351	24.392	27.255	54.462	1.00	0.00	H
ATOM	5120	CA	GLY	A	351	26.067	28.385	55.084	1.00	0.00	C
ATOM	5121	HA1	GLY	A	351	26.407	28.370	56.024	1.00	0.00	H
ATOM	5122	HA2	GLY	A	351	25.448	29.161	54.966	1.00	0.00	H
ATOM	5123	C	GLY	A	351	27.233	28.505	54.117	1.00	0.00	C
ATOM	5124	O	GLY	A	351	28.372	28.797	54.594	1.00	0.00	O
ATOM	5125	N	LEU	A	352	27.029	28.149	52.843	1.00	0.00	N
ATOM	5126	H	LEU	A	352	26.232	27.575	52.656	1.00	0.00	H
ATOM	5127	CA	LEU	A	352	27.858	28.518	51.703	1.00	0.00	C

ATOM	5128	HA	LEU	A	352	28.766	28.141	51.884	1.00	0.00	H
ATOM	5129	CB	LEU	A	352	27.466	27.878	50.431	1.00	0.00	C
ATOM	5130	HB1	LEU	A	352	28.257	27.959	49.825	1.00	0.00	H
ATOM	5131	HB2	LEU	A	352	26.711	28.424	50.068	1.00	0.00	H
ATOM	5132	CG	LEU	A	352	27.024	26.375	50.493	1.00	0.00	C
ATOM	5133	HG	LEU	A	352	26.117	26.378	50.914	1.00	0.00	H
ATOM	5134	CD1	LEU	A	352	26.919	25.688	49.136	1.00	0.00	C
ATOM	5135	HD11	LEU	A	352	26.633	24.738	49.264	1.00	0.00	H
ATOM	5136	HD12	LEU	A	352	26.245	26.165	48.572	1.00	0.00	H
ATOM	5137	HD13	LEU	A	352	27.810	25.710	48.682	1.00	0.00	H
ATOM	5138	CD2	LEU	A	352	28.061	25.555	51.275	1.00	0.00	C
ATOM	5139	HD21	LEU	A	352	27.772	24.598	51.310	1.00	0.00	H
ATOM	5140	HD22	LEU	A	352	28.949	25.619	50.819	1.00	0.00	H
ATOM	5141	HD23	LEU	A	352	28.137	25.914	52.205	1.00	0.00	H
ATOM	5142	C	LEU	A	352	27.942	29.999	51.609	1.00	0.00	C
ATOM	5143	O	LEU	A	352	26.988	30.691	51.988	1.00	0.00	O
ATOM	5144	N	GLY	A	353	28.984	30.614	51.046	1.00	0.00	N
ATOM	5145	H	GLY	A	353	29.672	30.034	50.610	1.00	0.00	H
ATOM	5146	CA	GLY	A	353	29.213	32.069	51.008	1.00	0.00	C
ATOM	5147	HA1	GLY	A	353	30.174	32.181	50.754	1.00	0.00	H
ATOM	5148	HA2	GLY	A	353	29.078	32.385	51.947	1.00	0.00	H
ATOM	5149	C	GLY	A	353	28.392	32.979	50.099	1.00	0.00	C
ATOM	5150	O	GLY	A	353	28.566	34.173	50.045	1.00	0.00	O
ATOM	5151	N	GLY	A	354	27.456	32.389	49.315	1.00	0.00	N
ATOM	5152	H	GLY	A	354	27.350	31.405	49.458	1.00	0.00	H
ATOM	5153	CA	GLY	A	354	26.590	32.949	48.310	1.00	0.00	C
ATOM	5154	HA1	GLY	A	354	26.889	33.887	48.133	1.00	0.00	H
ATOM	5155	HA2	GLY	A	354	25.659	32.959	48.676	1.00	0.00	H
ATOM	5156	C	GLY	A	354	26.593	32.205	47.045	1.00	0.00	C
ATOM	5157	O	GLY	A	354	27.048	31.052	47.023	1.00	0.00	O
ATOM	5158	N	ALA	A	355	26.140	32.882	45.952	1.00	0.00	N
ATOM	5159	H	ALA	A	355	25.955	33.864	45.994	1.00	0.00	H
ATOM	5160	CA	ALA	A	355	25.934	32.129	44.719	1.00	0.00	C
ATOM	5161	HA	ALA	A	355	26.349	31.249	44.953	1.00	0.00	H
ATOM	5162	CB	ALA	A	355	24.372	31.974	44.387	1.00	0.00	C
ATOM	5163	HB1	ALA	A	355	24.261	31.455	43.539	1.00	0.00	H
ATOM	5164	HB2	ALA	A	355	23.919	31.490	45.135	1.00	0.00	H
ATOM	5165	HB3	ALA	A	355	23.963	32.880	44.279	1.00	0.00	H
ATOM	5166	C	ALA	A	355	26.731	32.717	43.503	1.00	0.00	C
ATOM	5167	O	ALA	A	355	27.414	33.754	43.610	1.00	0.00	O
ATOM	5168	N	MET	A	356	26.671	31.919	42.399	1.00	0.00	N
ATOM	5169	H	MET	A	356	26.124	31.083	42.430	1.00	0.00	H
ATOM	5170	CA	MET	A	356	27.385	32.256	41.176	1.00	0.00	C
ATOM	5171	HA	MET	A	356	27.548	33.243	41.174	1.00	0.00	H
ATOM	5172	CB	MET	A	356	28.804	31.652	41.118	1.00	0.00	C
ATOM	5173	HB1	MET	A	356	28.756	30.710	41.451	1.00	0.00	H
ATOM	5174	HB2	MET	A	356	29.396	32.189	41.720	1.00	0.00	H
ATOM	5175	CG	MET	A	356	29.446	31.627	39.724	1.00	0.00	C
ATOM	5176	HG1	MET	A	356	30.428	31.691	39.902	1.00	0.00	H
ATOM	5177	HG2	MET	A	356	29.130	32.471	39.290	1.00	0.00	H
ATOM	5178	SD	MET	A	356	29.029	30.158	38.736	1.00	0.00	S
ATOM	5179	CE	MET	A	356	30.225	28.972	39.423	1.00	0.00	C
ATOM	5180	HE1	MET	A	356	30.117	28.088	38.967	1.00	0.00	H
ATOM	5181	HE2	MET	A	356	31.154	29.312	39.276	1.00	0.00	H

ATOM	5182	HE3	MET	A	356	30.061	28.864	40.404	1.00	0.00	H
ATOM	5183	C	MET	A	356	26.447	31.783	40.014	1.00	0.00	C
ATOM	5184	O	MET	A	356	25.819	30.713	40.218	1.00	0.00	O
ATOM	5185	N	VAL	A	357	26.443	32.508	38.855	1.00	0.00	N
ATOM	5186	H	VAL	A	357	27.040	33.309	38.812	1.00	0.00	H
ATOM	5187	CA	VAL	A	357	25.645	32.209	37.686	1.00	0.00	C
ATOM	5188	HA	VAL	A	357	25.305	31.315	37.979	1.00	0.00	H
ATOM	5189	CB	VAL	A	357	24.454	33.161	37.436	1.00	0.00	C
ATOM	5190	HB	VAL	A	357	24.812	34.083	37.288	1.00	0.00	H
ATOM	5191	CG1	VAL	A	357	23.717	32.774	36.167	1.00	0.00	C
ATOM	5192	HG11	VAL	A	357	22.951	33.400	36.022	1.00	0.00	H
ATOM	5193	HG12	VAL	A	357	24.343	32.827	35.389	1.00	0.00	H
ATOM	5194	HG13	VAL	A	357	23.372	31.840	36.254	1.00	0.00	H
ATOM	5195	CG2	VAL	A	357	23.503	33.221	38.660	1.00	0.00	C
ATOM	5196	HG21	VAL	A	357	22.746	33.844	38.464	1.00	0.00	H
ATOM	5197	HG22	VAL	A	357	23.142	32.307	38.847	1.00	0.00	H
ATOM	5198	HG23	VAL	A	357	24.007	33.550	39.458	1.00	0.00	H
ATOM	5199	C	VAL	A	357	26.463	32.074	36.401	1.00	0.00	C
ATOM	5200	O	VAL	A	357	27.048	33.071	35.966	1.00	0.00	O
ATOM	5201	N	TRP	A	358	26.332	30.926	35.734	1.00	0.00	N
ATOM	5202	H	TRP	A	358	26.003	30.127	36.237	1.00	0.00	H
ATOM	5203	CA	TRP	A	358	26.640	30.764	34.313	1.00	0.00	C
ATOM	5204	HA	TRP	A	358	27.123	31.587	34.014	1.00	0.00	H
ATOM	5205	CB	TRP	A	358	27.564	29.560	34.106	1.00	0.00	C
ATOM	5206	HB1	TRP	A	358	27.038	28.718	34.229	1.00	0.00	H
ATOM	5207	HB2	TRP	A	358	28.302	29.593	34.780	1.00	0.00	H
ATOM	5208	CG	TRP	A	358	28.180	29.528	32.744	1.00	0.00	C
ATOM	5209	CD1	TRP	A	358	27.564	29.181	31.587	1.00	0.00	C
ATOM	5210	HD1	TRP	A	358	26.626	28.843	31.510	1.00	0.00	H
ATOM	5211	NE1	TRP	A	358	28.434	29.368	30.531	1.00	0.00	N
ATOM	5212	HE1	TRP	A	358	28.208	29.177	29.576	1.00	0.00	H
ATOM	5213	CE2	TRP	A	358	29.649	29.849	30.955	1.00	0.00	C
ATOM	5214	CZ2	TRP	A	358	30.820	30.197	30.275	1.00	0.00	C
ATOM	5215	HZ2	TRP	A	358	30.868	30.124	29.279	1.00	0.00	H
ATOM	5216	CH2	TRP	A	358	31.927	30.650	31.008	1.00	0.00	C
ATOM	5217	HH2	TRP	A	358	32.765	30.921	30.534	1.00	0.00	H
ATOM	5218	CZ3	TRP	A	358	31.855	30.722	32.410	1.00	0.00	C
ATOM	5219	HZ3	TRP	A	358	32.649	31.032	32.933	1.00	0.00	H
ATOM	5220	CE3	TRP	A	358	30.673	30.361	33.082	1.00	0.00	C
ATOM	5221	HE3	TRP	A	358	30.641	30.409	34.080	1.00	0.00	H
ATOM	5222	CD2	TRP	A	358	29.532	29.932	32.371	1.00	0.00	C
ATOM	5223	C	TRP	A	358	25.321	30.608	33.535	1.00	0.00	C
ATOM	5224	O	TRP	A	358	24.612	29.630	33.745	1.00	0.00	O
ATOM	5225	N	ALA	A	359	24.884	31.544	32.693	1.00	0.00	N
ATOM	5226	H	ALA	A	359	24.031	31.325	32.219	1.00	0.00	H
ATOM	5227	CA	ALA	A	359	25.467	32.846	32.359	1.00	0.00	C
ATOM	5228	HA	ALA	A	359	26.013	33.119	33.151	1.00	0.00	H
ATOM	5229	CB	ALA	A	359	26.359	32.685	31.119	1.00	0.00	C
ATOM	5230	HB1	ALA	A	359	26.763	33.569	30.882	1.00	0.00	H
ATOM	5231	HB2	ALA	A	359	27.087	32.028	31.315	1.00	0.00	H
ATOM	5232	HB3	ALA	A	359	25.808	32.354	30.353	1.00	0.00	H
ATOM	5233	C	ALA	A	359	24.380	33.920	32.137	1.00	0.00	C
ATOM	5234	O	ALA	A	359	23.193	33.627	31.989	1.00	0.00	O
ATOM	5235	N	LEU	A	360	24.810	35.179	32.071	1.00	0.00	N

ATOM	5236	H	LEU	A	360	25.797	35.329	32.121	1.00	0.00	H
ATOM	5237	CA	LEU	A	360	23.981	36.328	31.935	1.00	0.00	C
ATOM	5238	HA	LEU	A	360	23.355	36.121	32.687	1.00	0.00	H
ATOM	5239	CB	LEU	A	360	24.731	37.656	32.079	1.00	0.00	C
ATOM	5240	HB1	LEU	A	360	24.095	38.405	31.894	1.00	0.00	H
ATOM	5241	HB2	LEU	A	360	25.475	37.678	31.412	1.00	0.00	H
ATOM	5242	CG	LEU	A	360	25.319	37.833	33.494	1.00	0.00	C
ATOM	5243	HG	LEU	A	360	25.549	36.919	33.829	1.00	0.00	H
ATOM	5244	CD1	LEU	A	360	26.623	38.547	33.565	1.00	0.00	C
ATOM	5245	HD11	LEU	A	360	26.917	38.610	34.519	1.00	0.00	H
ATOM	5246	HD12	LEU	A	360	27.308	38.044	33.037	1.00	0.00	H
ATOM	5247	HD13	LEU	A	360	26.520	39.467	33.187	1.00	0.00	H
ATOM	5248	CD2	LEU	A	360	24.290	38.542	34.382	1.00	0.00	C
ATOM	5249	HD21	LEU	A	360	24.667	38.659	35.301	1.00	0.00	H
ATOM	5250	HD22	LEU	A	360	24.075	39.438	33.993	1.00	0.00	H
ATOM	5251	HD23	LEU	A	360	23.457	37.992	34.431	1.00	0.00	H
ATOM	5252	C	LEU	A	360	23.290	36.443	30.588	1.00	0.00	C
ATOM	5253	O	LEU	A	360	22.230	37.057	30.458	1.00	0.00	O
ATOM	5254	N	ASP	A	361	23.794	35.734	29.565	1.00	0.00	N
ATOM	5255	H	ASP	A	361	24.644	35.229	29.712	1.00	0.00	H
ATOM	5256	CA	ASP	A	361	23.167	35.662	28.252	1.00	0.00	C
ATOM	5257	HA	ASP	A	361	22.579	36.470	28.225	1.00	0.00	H
ATOM	5258	CB	ASP	A	361	24.346	35.608	27.232	1.00	0.00	C
ATOM	5259	HB1	ASP	A	361	24.812	36.493	27.237	1.00	0.00	H
ATOM	5260	HB2	ASP	A	361	23.971	35.433	26.321	1.00	0.00	H
ATOM	5261	CG	ASP	A	361	25.394	34.528	27.520	1.00	0.00	C
ATOM	5262	OD1	ASP	A	361	25.314	33.438	26.915	1.00	0.00	O
ATOM	5263	OD2	ASP	A	361	26.324	34.802	28.318	1.00	0.00	O
ATOM	5264	C	ASP	A	361	22.215	34.471	27.998	1.00	0.00	C
ATOM	5265	O	ASP	A	361	21.887	34.230	26.846	1.00	0.00	O
ATOM	5266	N	LEU	A	362	21.839	33.755	29.061	1.00	0.00	N
ATOM	5267	H	LEU	A	362	22.065	34.134	29.959	1.00	0.00	H
ATOM	5268	CA	LEU	A	362	21.123	32.460	29.049	1.00	0.00	C
ATOM	5269	HA	LEU	A	362	20.858	32.379	28.088	1.00	0.00	H
ATOM	5270	CB	LEU	A	362	22.069	31.310	29.452	1.00	0.00	C
ATOM	5271	HB1	LEU	A	362	21.545	30.459	29.432	1.00	0.00	H
ATOM	5272	HB2	LEU	A	362	22.386	31.485	30.384	1.00	0.00	H
ATOM	5273	CG	LEU	A	362	23.309	31.122	28.558	1.00	0.00	C
ATOM	5274	HG	LEU	A	362	23.780	32.004	28.532	1.00	0.00	H
ATOM	5275	CD1	LEU	A	362	24.201	30.022	29.138	1.00	0.00	C
ATOM	5276	HD11	LEU	A	362	25.005	29.903	28.555	1.00	0.00	H
ATOM	5277	HD12	LEU	A	362	24.492	30.280	30.059	1.00	0.00	H
ATOM	5278	HD13	LEU	A	362	23.689	29.164	29.179	1.00	0.00	H
ATOM	5279	CD2	LEU	A	362	22.956	30.723	27.122	1.00	0.00	C
ATOM	5280	HD21	LEU	A	362	23.796	30.615	26.590	1.00	0.00	H
ATOM	5281	HD22	LEU	A	362	22.453	29.858	27.130	1.00	0.00	H
ATOM	5282	HD23	LEU	A	362	22.388	31.435	26.708	1.00	0.00	H
ATOM	5283	C	LEU	A	362	19.802	32.419	29.870	1.00	0.00	C
ATOM	5284	O	LEU	A	362	18.984	31.486	29.764	1.00	0.00	O
ATOM	5285	N	ASP	A	363	19.552	33.468	30.664	1.00	0.00	N
ATOM	5286	H	ASP	A	363	20.301	34.109	30.831	1.00	0.00	H
ATOM	5287	CA	ASP	A	363	18.269	33.749	31.305	1.00	0.00	C
ATOM	5288	HA	ASP	A	363	17.809	32.883	31.500	1.00	0.00	H
ATOM	5289	CB	ASP	A	363	18.548	34.494	32.606	1.00	0.00	C

ATOM	5290	HB1	ASP	A	363	18.804	35.435	32.386	1.00	0.00	H
ATOM	5291	HB2	ASP	A	363	19.307	34.043	33.075	1.00	0.00	H
ATOM	5292	CG	ASP	A	363	17.377	34.539	33.552	1.00	0.00	C
ATOM	5293	OD1	ASP	A	363	16.342	33.867	33.377	1.00	0.00	O
ATOM	5294	OD2	ASP	A	363	17.437	35.331	34.514	1.00	0.00	O
ATOM	5295	C	ASP	A	363	17.389	34.570	30.356	1.00	0.00	C
ATOM	5296	O	ASP	A	363	17.921	34.878	29.298	1.00	0.00	O
ATOM	5297	N	ASP	A	364	16.160	34.860	30.738	1.00	0.00	N
ATOM	5298	H	ASP	A	364	15.808	34.438	31.573	1.00	0.00	H
ATOM	5299	CA	ASP	A	364	15.277	35.783	29.986	1.00	0.00	C
ATOM	5300	HA	ASP	A	364	15.421	35.633	29.008	1.00	0.00	H
ATOM	5301	CB	ASP	A	364	13.709	35.515	30.228	1.00	0.00	C
ATOM	5302	HB1	ASP	A	364	13.437	35.816	31.142	1.00	0.00	H
ATOM	5303	HB2	ASP	A	364	13.497	34.544	30.121	1.00	0.00	H
ATOM	5304	CG	ASP	A	364	12.853	36.233	29.283	1.00	0.00	C
ATOM	5305	OD1	ASP	A	364	13.329	37.008	28.506	1.00	0.00	O
ATOM	5306	OD2	ASP	A	364	11.643	36.035	29.263	1.00	0.00	O
ATOM	5307	C	ASP	A	364	15.759	37.185	30.371	1.00	0.00	C
ATOM	5308	O	ASP	A	364	15.134	37.973	31.106	1.00	0.00	O
ATOM	5309	N	PHE	A	365	16.699	37.711	29.621	1.00	0.00	N
ATOM	5310	H	PHE	A	365	17.007	37.191	28.824	1.00	0.00	H
ATOM	5311	CA	PHE	A	365	17.338	39.035	29.880	1.00	0.00	C
ATOM	5312	HA	PHE	A	365	17.361	39.149	30.873	1.00	0.00	H
ATOM	5313	CB	PHE	A	365	18.829	39.089	29.336	1.00	0.00	C
ATOM	5314	HB1	PHE	A	365	19.420	38.472	29.856	1.00	0.00	H
ATOM	5315	HB2	PHE	A	365	19.192	40.019	29.387	1.00	0.00	H
ATOM	5316	CG	PHE	A	365	18.872	38.668	27.926	1.00	0.00	C
ATOM	5317	CD1	PHE	A	365	18.350	39.436	26.872	1.00	0.00	C
ATOM	5318	HD1	PHE	A	365	17.907	40.308	27.082	1.00	0.00	H
ATOM	5319	CE1	PHE	A	365	18.428	39.027	25.559	1.00	0.00	C
ATOM	5320	HE1	PHE	A	365	17.999	39.566	24.835	1.00	0.00	H
ATOM	5321	CZ	PHE	A	365	19.119	37.842	25.250	1.00	0.00	C
ATOM	5322	HZ	PHE	A	365	19.247	37.566	24.297	1.00	0.00	H
ATOM	5323	CE2	PHE	A	365	19.622	37.058	26.280	1.00	0.00	C
ATOM	5324	HE2	PHE	A	365	20.018	36.164	26.072	1.00	0.00	H
ATOM	5325	CD2	PHE	A	365	19.581	37.501	27.591	1.00	0.00	C
ATOM	5326	HD2	PHE	A	365	20.059	36.988	28.304	1.00	0.00	H
ATOM	5327	C	PHE	A	365	16.453	40.140	29.250	1.00	0.00	C
ATOM	5328	O	PHE	A	365	16.572	41.336	29.511	1.00	0.00	O
ATOM	5329	N	ALA	A	366	15.487	39.754	28.372	1.00	0.00	N
ATOM	5330	H	ALA	A	366	15.403	38.767	28.232	1.00	0.00	H
ATOM	5331	CA	ALA	A	366	14.551	40.584	27.602	1.00	0.00	C
ATOM	5332	HA	ALA	A	366	14.930	41.509	27.560	1.00	0.00	H
ATOM	5333	CB	ALA	A	366	14.480	40.005	26.177	1.00	0.00	C
ATOM	5334	HB1	ALA	A	366	13.848	40.551	25.627	1.00	0.00	H
ATOM	5335	HB2	ALA	A	366	15.390	40.028	25.762	1.00	0.00	H
ATOM	5336	HB3	ALA	A	366	14.156	39.060	26.217	1.00	0.00	H
ATOM	5337	C	ALA	A	366	13.150	40.684	28.316	1.00	0.00	C
ATOM	5338	O	ALA	A	366	12.392	41.661	28.007	1.00	0.00	O
ATOM	5339	N	GLY	A	367	12.787	39.671	29.115	1.00	0.00	N
ATOM	5340	H	GLY	A	367	13.480	38.965	29.262	1.00	0.00	H
ATOM	5341	CA	GLY	A	367	11.500	39.457	29.808	1.00	0.00	C
ATOM	5342	HA1	GLY	A	367	11.271	40.307	30.283	1.00	0.00	H
ATOM	5343	HA2	GLY	A	367	11.638	38.727	30.478	1.00	0.00	H

ATOM	5344	C	GLY	A	367	10.347	39.065	28.800	1.00	0.00	C
ATOM	5345	O	GLY	A	367	9.173	39.123	29.085	1.00	0.00	O
ATOM	5346	N	PHE	A	368	10.748	38.539	27.618	1.00	0.00	N
ATOM	5347	H	PHE	A	368	11.720	38.330	27.509	1.00	0.00	H
ATOM	5348	CA	PHE	A	368	9.868	38.257	26.497	1.00	0.00	C
ATOM	5349	HA	PHE	A	368	8.932	38.548	26.696	1.00	0.00	H
ATOM	5350	CB	PHE	A	368	10.702	38.994	25.374	1.00	0.00	C
ATOM	5351	HB1	PHE	A	368	11.604	38.569	25.293	1.00	0.00	H
ATOM	5352	HB2	PHE	A	368	10.809	39.959	25.615	1.00	0.00	H
ATOM	5353	CG	PHE	A	368	10.081	38.952	24.021	1.00	0.00	C
ATOM	5354	CD1	PHE	A	368	10.646	38.060	23.073	1.00	0.00	C
ATOM	5355	HD1	PHE	A	368	11.495	37.583	23.301	1.00	0.00	H
ATOM	5356	CE1	PHE	A	368	10.046	37.828	21.821	1.00	0.00	C
ATOM	5357	HE1	PHE	A	368	10.409	37.088	21.255	1.00	0.00	H
ATOM	5358	CZ	PHE	A	368	8.968	38.574	21.321	1.00	0.00	C
ATOM	5359	HZ	PHE	A	368	8.613	38.482	20.391	1.00	0.00	H
ATOM	5360	CE2	PHE	A	368	8.443	39.463	22.270	1.00	0.00	C
ATOM	5361	HE2	PHE	A	368	7.679	40.038	21.977	1.00	0.00	H
ATOM	5362	CD2	PHE	A	368	8.906	39.629	23.644	1.00	0.00	C
ATOM	5363	HD2	PHE	A	368	8.407	40.203	24.294	1.00	0.00	H
ATOM	5364	C	PHE	A	368	9.619	36.783	26.187	1.00	0.00	C
ATOM	5365	O	PHE	A	368	8.782	36.573	25.245	1.00	0.00	O
ATOM	5366	N	SER	A	369	10.407	35.872	26.781	1.00	0.00	N
ATOM	5367	H	SER	A	369	11.062	36.180	27.471	1.00	0.00	H
ATOM	5368	CA	SER	A	369	10.344	34.443	26.458	1.00	0.00	C
ATOM	5369	HA	SER	A	369	9.745	34.426	25.658	1.00	0.00	H
ATOM	5370	CB	SER	A	369	11.770	33.910	26.221	1.00	0.00	C
ATOM	5371	HB1	SER	A	369	11.761	32.928	26.034	1.00	0.00	H
ATOM	5372	HB2	SER	A	369	12.359	34.094	27.008	1.00	0.00	H
ATOM	5373	OG	SER	A	369	12.384	34.495	25.143	1.00	0.00	O
ATOM	5374	HG	SER	A	369	13.302	34.114	25.035	1.00	0.00	H
ATOM	5375	C	SER	A	369	9.677	33.570	27.470	1.00	0.00	C
ATOM	5376	O	SER	A	369	9.052	32.572	27.044	1.00	0.00	O
ATOM	5377	N	CYS	A	370	9.794	34.014	28.690	1.00	0.00	N
ATOM	5378	H	CYS	A	370	10.365	34.826	28.810	1.00	0.00	H
ATOM	5379	CA	CYS	A	370	9.173	33.451	29.938	1.00	0.00	C
ATOM	5380	HA	CYS	A	370	8.741	32.583	29.692	1.00	0.00	H
ATOM	5381	CB	CYS	A	370	10.304	33.211	30.940	1.00	0.00	C
ATOM	5382	HB1	CYS	A	370	9.882	32.641	31.645	1.00	0.00	H
ATOM	5383	HB2	CYS	A	370	10.490	34.120	31.313	1.00	0.00	H
ATOM	5384	SG	CYS	A	370	11.802	32.444	30.267	1.00	0.00	S
ATOM	5385	C	CYS	A	370	8.006	34.304	30.517	1.00	0.00	C
ATOM	5386	O	CYS	A	370	7.191	33.924	31.412	1.00	0.00	O
ATOM	5387	N	ASN	A	371	7.943	35.528	29.991	1.00	0.00	N
ATOM	5388	H	ASN	A	371	8.692	35.833	29.403	1.00	0.00	H
ATOM	5389	CA	ASN	A	371	6.828	36.446	30.235	1.00	0.00	C
ATOM	5390	HA	ASN	A	371	7.181	37.369	30.083	1.00	0.00	H
ATOM	5391	CB	ASN	A	371	5.715	36.069	29.209	1.00	0.00	C
ATOM	5392	HB1	ASN	A	371	5.072	36.831	29.130	1.00	0.00	H
ATOM	5393	HB2	ASN	A	371	5.231	35.257	29.536	1.00	0.00	H
ATOM	5394	CG	ASN	A	371	6.225	35.764	27.825	1.00	0.00	C
ATOM	5395	OD1	ASN	A	371	6.339	34.642	27.336	1.00	0.00	O
ATOM	5396	ND2	ASN	A	371	6.584	36.819	27.155	1.00	0.00	N
ATOM	5397	HD21	ASN	A	371	6.507	37.725	27.570	1.00	0.00	H

ATOM	5398	HD22	ASN	A	371	6.937	36.724	26.224	1.00	0.00	H
ATOM	5399	C	ASN	A	371	6.372	36.554	31.700	1.00	0.00	C
ATOM	5400	O	ASN	A	371	5.198	36.301	31.934	1.00	0.00	O
ATOM	5401	N	GLN	A	372	7.392	36.779	32.615	1.00	0.00	N
ATOM	5402	H	GLN	A	372	8.292	36.920	32.204	1.00	0.00	H
ATOM	5403	CA	GLN	A	372	7.378	36.845	34.074	1.00	0.00	C
ATOM	5404	HA	GLN	A	372	6.424	37.075	34.266	1.00	0.00	H
ATOM	5405	CB	GLN	A	372	7.769	35.461	34.590	1.00	0.00	C
ATOM	5406	HB1	GLN	A	372	7.158	34.807	34.144	1.00	0.00	H
ATOM	5407	HB2	GLN	A	372	7.593	35.464	35.574	1.00	0.00	H
ATOM	5408	CG	GLN	A	372	9.182	34.922	34.408	1.00	0.00	C
ATOM	5409	HG1	GLN	A	372	9.769	35.343	35.100	1.00	0.00	H
ATOM	5410	HG2	GLN	A	372	9.501	35.182	33.497	1.00	0.00	H
ATOM	5411	CD	GLN	A	372	9.308	33.417	34.538	1.00	0.00	C
ATOM	5412	OE1	GLN	A	372	10.327	32.873	34.125	1.00	0.00	O
ATOM	5413	NE2	GLN	A	372	8.448	32.838	35.348	1.00	0.00	N
ATOM	5414	HE21	GLN	A	372	7.767	33.389	35.831	1.00	0.00	H
ATOM	5415	HE22	GLN	A	372	8.475	31.847	35.480	1.00	0.00	H
ATOM	5416	C	GLN	A	372	8.227	37.992	34.764	1.00	0.00	C
ATOM	5417	O	GLN	A	372	8.492	37.976	35.961	1.00	0.00	O
ATOM	5418	N	GLY	A	373	8.641	38.941	33.970	1.00	0.00	N
ATOM	5419	H	GLY	A	373	8.212	39.015	33.070	1.00	0.00	H
ATOM	5420	CA	GLY	A	373	9.675	39.891	34.302	1.00	0.00	C
ATOM	5421	HA1	GLY	A	373	9.746	39.901	35.299	1.00	0.00	H
ATOM	5422	HA2	GLY	A	373	9.358	40.784	33.982	1.00	0.00	H
ATOM	5423	C	GLY	A	373	11.061	39.548	33.664	1.00	0.00	C
ATOM	5424	O	GLY	A	373	11.314	38.369	33.433	1.00	0.00	O
ATOM	5425	N	ARG	A	374	11.904	40.456	33.289	1.00	0.00	N
ATOM	5426	H	ARG	A	374	11.605	41.410	33.298	1.00	0.00	H
ATOM	5427	CA	ARG	A	374	13.359	40.144	32.824	1.00	0.00	C
ATOM	5428	HA	ARG	A	374	13.257	39.287	32.320	1.00	0.00	H
ATOM	5429	CB	ARG	A	374	13.882	41.287	31.838	1.00	0.00	C
ATOM	5430	HB1	ARG	A	374	13.221	41.439	31.103	1.00	0.00	H
ATOM	5431	HB2	ARG	A	374	14.764	41.027	31.444	1.00	0.00	H
ATOM	5432	CG	ARG	A	374	14.071	42.594	32.563	1.00	0.00	C
ATOM	5433	HG1	ARG	A	374	14.604	42.436	33.395	1.00	0.00	H
ATOM	5434	HG2	ARG	A	374	13.176	42.969	32.806	1.00	0.00	H
ATOM	5435	CD	ARG	A	374	14.828	43.561	31.602	1.00	0.00	C
ATOM	5436	HD1	ARG	A	374	15.594	43.072	31.185	1.00	0.00	H
ATOM	5437	HD2	ARG	A	374	15.174	44.338	32.128	1.00	0.00	H
ATOM	5438	NE	ARG	A	374	13.960	44.107	30.482	1.00	0.00	N
ATOM	5439	HE	ARG	A	374	12.986	44.198	30.690	1.00	0.00	H
ATOM	5440	CZ	ARG	A	374	14.333	44.482	29.251	1.00	0.00	C
ATOM	5441	NH1	ARG	A	374	15.328	43.959	28.587	1.00	0.00	N
ATOM	5442	HH11	ARG	A	374	15.871	43.225	28.995	1.00	0.00	H
ATOM	5443	HH12	ARG	A	374	15.547	44.292	27.670	1.00	0.00	H
ATOM	5444	NH2	ARG	A	374	13.697	45.465	28.645	1.00	0.00	N
ATOM	5445	HH21	ARG	A	374	12.939	45.928	29.104	1.00	0.00	H
ATOM	5446	HH22	ARG	A	374	13.973	45.748	27.726	1.00	0.00	H
ATOM	5447	C	ARG	A	374	14.420	39.775	33.984	1.00	0.00	C
ATOM	5448	O	ARG	A	374	14.233	40.135	35.152	1.00	0.00	O
ATOM	5449	N	TYR	A	375	15.495	39.189	33.457	1.00	0.00	N
ATOM	5450	H	TYR	A	375	15.741	39.352	32.501	1.00	0.00	H
ATOM	5451	CA	TYR	A	375	16.317	38.300	34.294	1.00	0.00	C

ATOM	5452	HA	TYR	A	375	16.613	37.549	33.704	1.00	0.00	H
ATOM	5453	CB	TYR	A	375	17.442	39.211	34.809	1.00	0.00	C
ATOM	5454	HB1	TYR	A	375	18.016	38.697	35.447	1.00	0.00	H
ATOM	5455	HB2	TYR	A	375	17.040	39.995	35.281	1.00	0.00	H
ATOM	5456	CG	TYR	A	375	18.318	39.729	33.710	1.00	0.00	C
ATOM	5457	CD1	TYR	A	375	19.234	38.943	32.963	1.00	0.00	C
ATOM	5458	HD1	TYR	A	375	19.255	37.946	33.043	1.00	0.00	H
ATOM	5459	CE1	TYR	A	375	20.073	39.600	32.142	1.00	0.00	C
ATOM	5460	HE1	TYR	A	375	20.749	39.053	31.649	1.00	0.00	H
ATOM	5461	CZ	TYR	A	375	20.084	41.004	31.913	1.00	0.00	C
ATOM	5462	OH	TYR	A	375	21.074	41.540	31.220	1.00	0.00	O
ATOM	5463	HH	TYR	A	375	20.935	42.528	31.149	1.00	0.00	H
ATOM	5464	CE2	TYR	A	375	19.190	41.755	32.601	1.00	0.00	C
ATOM	5465	HE2	TYR	A	375	19.180	42.747	32.478	1.00	0.00	H
ATOM	5466	CD2	TYR	A	375	18.276	41.155	33.487	1.00	0.00	C
ATOM	5467	HD2	TYR	A	375	17.598	41.716	33.962	1.00	0.00	H
ATOM	5468	C	TYR	A	375	15.613	37.516	35.414	1.00	0.00	C
ATOM	5469	O	TYR	A	375	15.905	37.680	36.577	1.00	0.00	O
ATOM	5470	N	PRO	A	376	14.574	36.683	35.169	1.00	0.00	N
ATOM	5471	CD	PRO	A	376	14.110	36.258	33.822	1.00	0.00	C
ATOM	5472	HD1	PRO	A	376	14.869	35.878	33.292	1.00	0.00	H
ATOM	5473	HD2	PRO	A	376	13.712	37.031	33.327	1.00	0.00	H
ATOM	5474	CG	PRO	A	376	13.059	35.193	34.143	1.00	0.00	C
ATOM	5475	HG1	PRO	A	376	13.475	34.288	34.235	1.00	0.00	H
ATOM	5476	HG2	PRO	A	376	12.347	35.163	33.441	1.00	0.00	H
ATOM	5477	CB	PRO	A	376	12.496	35.671	35.470	1.00	0.00	C
ATOM	5478	HB1	PRO	A	376	12.069	34.912	35.961	1.00	0.00	H
ATOM	5479	HB2	PRO	A	376	11.821	36.393	35.322	1.00	0.00	H
ATOM	5480	CA	PRO	A	376	13.720	36.209	36.241	1.00	0.00	C
ATOM	5481	HA	PRO	A	376	13.585	36.987	36.854	1.00	0.00	H
ATOM	5482	C	PRO	A	376	14.294	35.097	37.157	1.00	0.00	C
ATOM	5483	O	PRO	A	376	14.057	35.084	38.370	1.00	0.00	O
ATOM	5484	N	LEU	A	377	15.168	34.248	36.625	1.00	0.00	N
ATOM	5485	H	LEU	A	377	15.302	34.286	35.635	1.00	0.00	H
ATOM	5486	CA	LEU	A	377	15.954	33.256	37.379	1.00	0.00	C
ATOM	5487	HA	LEU	A	377	15.294	32.655	37.831	1.00	0.00	H
ATOM	5488	CB	LEU	A	377	16.859	32.440	36.441	1.00	0.00	C
ATOM	5489	HB1	LEU	A	377	17.596	32.051	36.994	1.00	0.00	H
ATOM	5490	HB2	LEU	A	377	17.245	33.071	35.768	1.00	0.00	H
ATOM	5491	CG	LEU	A	377	16.177	31.282	35.672	1.00	0.00	C
ATOM	5492	HG	LEU	A	377	15.459	31.616	35.061	1.00	0.00	H
ATOM	5493	CD1	LEU	A	377	17.211	30.566	34.841	1.00	0.00	C
ATOM	5494	HD11	LEU	A	377	16.777	29.816	34.342	1.00	0.00	H
ATOM	5495	HD12	LEU	A	377	17.619	31.207	34.191	1.00	0.00	H
ATOM	5496	HD13	LEU	A	377	17.924	30.199	35.439	1.00	0.00	H
ATOM	5497	CD2	LEU	A	377	15.552	30.332	36.662	1.00	0.00	C
ATOM	5498	HD21	LEU	A	377	15.110	29.582	36.171	1.00	0.00	H
ATOM	5499	HD22	LEU	A	377	16.261	29.964	37.264	1.00	0.00	H
ATOM	5500	HD23	LEU	A	377	14.871	30.820	37.208	1.00	0.00	H
ATOM	5501	C	LEU	A	377	16.880	34.031	38.356	1.00	0.00	C
ATOM	5502	O	LEU	A	377	16.781	33.933	39.566	1.00	0.00	O
ATOM	5503	N	ILE	A	378	17.709	34.863	37.799	1.00	0.00	N
ATOM	5504	H	ILE	A	378	17.661	34.965	36.805	1.00	0.00	H
ATOM	5505	CA	ILE	A	378	18.707	35.662	38.510	1.00	0.00	C

ATOM	5506	HA	ILE	A	378	19.328	34.988	38.911	1.00	0.00	H
ATOM	5507	CB	ILE	A	378	19.605	36.519	37.593	1.00	0.00	C
ATOM	5508	HB	ILE	A	378	19.098	37.237	37.116	1.00	0.00	H
ATOM	5509	CG2	ILE	A	378	20.567	37.399	38.292	1.00	0.00	C
ATOM	5510	HG21	ILE	A	378	21.100	37.911	37.619	1.00	0.00	H
ATOM	5511	HG22	ILE	A	378	20.069	38.038	38.878	1.00	0.00	H
ATOM	5512	HG23	ILE	A	378	21.182	36.843	38.851	1.00	0.00	H
ATOM	5513	CG1	ILE	A	378	20.181	35.589	36.579	1.00	0.00	C
ATOM	5514	HG11	ILE	A	378	19.433	35.281	35.991	1.00	0.00	H
ATOM	5515	HG12	ILE	A	378	20.563	34.806	37.069	1.00	0.00	H
ATOM	5516	CD	ILE	A	378	21.254	36.143	35.699	1.00	0.00	C
ATOM	5517	HD1	ILE	A	378	21.562	35.435	35.063	1.00	0.00	H
ATOM	5518	HD2	ILE	A	378	20.894	36.920	35.183	1.00	0.00	H
ATOM	5519	HD3	ILE	A	378	22.024	36.444	36.262	1.00	0.00	H
ATOM	5520	C	ILE	A	378	18.031	36.447	39.637	1.00	0.00	C
ATOM	5521	O	ILE	A	378	18.374	36.506	40.761	1.00	0.00	O
ATOM	5522	N	GLN	A	379	16.969	37.133	39.288	1.00	0.00	N
ATOM	5523	H	GLN	A	379	16.693	37.127	38.327	1.00	0.00	H
ATOM	5524	CA	GLN	A	379	16.146	37.929	40.267	1.00	0.00	C
ATOM	5525	HA	GLN	A	379	16.765	38.562	40.731	1.00	0.00	H
ATOM	5526	CB	GLN	A	379	15.068	38.708	39.540	1.00	0.00	C
ATOM	5527	HB1	GLN	A	379	14.337	38.882	40.200	1.00	0.00	H
ATOM	5528	HB2	GLN	A	379	14.724	38.119	38.809	1.00	0.00	H
ATOM	5529	CG	GLN	A	379	15.392	40.029	38.906	1.00	0.00	C
ATOM	5530	HG1	GLN	A	379	14.530	40.358	38.521	1.00	0.00	H
ATOM	5531	HG2	GLN	A	379	16.030	39.819	38.165	1.00	0.00	H
ATOM	5532	CD	GLN	A	379	16.012	41.193	39.758	1.00	0.00	C
ATOM	5533	OE1	GLN	A	379	16.341	40.937	40.914	1.00	0.00	O
ATOM	5534	NE2	GLN	A	379	16.209	42.370	39.210	1.00	0.00	N
ATOM	5535	HE21	GLN	A	379	15.964	42.523	38.253	1.00	0.00	H
ATOM	5536	HE22	GLN	A	379	16.604	43.113	39.750	1.00	0.00	H
ATOM	5537	C	GLN	A	379	15.475	36.999	41.279	1.00	0.00	C
ATOM	5538	O	GLN	A	379	15.537	37.381	42.429	1.00	0.00	O
ATOM	5539	N	THR	A	380	14.916	35.824	40.928	1.00	0.00	N
ATOM	5540	H	THR	A	380	14.732	35.608	39.969	1.00	0.00	H
ATOM	5541	CA	THR	A	380	14.562	34.806	42.063	1.00	0.00	C
ATOM	5542	HA	THR	A	380	13.865	35.263	42.615	1.00	0.00	H
ATOM	5543	CB	THR	A	380	13.989	33.505	41.497	1.00	0.00	C
ATOM	5544	HB	THR	A	380	14.583	33.087	40.810	1.00	0.00	H
ATOM	5545	CG2	THR	A	380	13.692	32.522	42.665	1.00	0.00	C
ATOM	5546	HG21	THR	A	380	13.317	31.671	42.297	1.00	0.00	H
ATOM	5547	HG22	THR	A	380	14.539	32.327	43.160	1.00	0.00	H
ATOM	5548	HG23	THR	A	380	13.029	32.936	43.289	1.00	0.00	H
ATOM	5549	OG1	THR	A	380	12.933	33.736	40.664	1.00	0.00	O
ATOM	5550	HG1	THR	A	380	12.587	32.865	40.315	1.00	0.00	H
ATOM	5551	C	THR	A	380	15.724	34.471	43.017	1.00	0.00	C
ATOM	5552	O	THR	A	380	15.597	34.676	44.227	1.00	0.00	O
ATOM	5553	N	LEU	A	381	16.903	34.090	42.530	1.00	0.00	N
ATOM	5554	H	LEU	A	381	16.980	33.936	41.545	1.00	0.00	H
ATOM	5555	CA	LEU	A	381	18.091	33.884	43.355	1.00	0.00	C
ATOM	5556	HA	LEU	A	381	17.888	33.216	44.071	1.00	0.00	H
ATOM	5557	CB	LEU	A	381	19.149	33.355	42.340	1.00	0.00	C
ATOM	5558	HB1	LEU	A	381	18.975	33.786	41.455	1.00	0.00	H
ATOM	5559	HB2	LEU	A	381	19.035	32.365	42.257	1.00	0.00	H

ATOM	5560	CG	LEU	A	381	20.619	33.626	42.711	1.00	0.00	C
ATOM	5561	HG	LEU	A	381	20.751	34.605	42.867	1.00	0.00	H
ATOM	5562	CD1	LEU	A	381	21.100	33.063	44.004	1.00	0.00	C
ATOM	5563	HD11	LEU	A	381	22.062	33.303	44.135	1.00	0.00	H
ATOM	5564	HD12	LEU	A	381	20.557	33.441	44.754	1.00	0.00	H
ATOM	5565	HD13	LEU	A	381	21.003	32.068	43.991	1.00	0.00	H
ATOM	5566	CD2	LEU	A	381	21.412	33.002	41.557	1.00	0.00	C
ATOM	5567	HD21	LEU	A	381	22.391	33.132	41.716	1.00	0.00	H
ATOM	5568	HD22	LEU	A	381	21.210	32.024	41.504	1.00	0.00	H
ATOM	5569	HD23	LEU	A	381	21.152	33.443	40.698	1.00	0.00	H
ATOM	5570	C	LEU	A	381	18.513	35.166	44.168	1.00	0.00	C
ATOM	5571	O	LEU	A	381	18.587	35.029	45.374	1.00	0.00	O
ATOM	5572	N	ARG	A	382	18.479	36.336	43.516	1.00	0.00	N
ATOM	5573	H	ARG	A	382	18.330	36.322	42.527	1.00	0.00	H
ATOM	5574	CA	ARG	A	382	18.648	37.630	44.169	1.00	0.00	C
ATOM	5575	HA	ARG	A	382	19.512	37.617	44.672	1.00	0.00	H
ATOM	5576	CB	ARG	A	382	18.685	38.667	43.073	1.00	0.00	C
ATOM	5577	HB1	ARG	A	382	17.760	38.722	42.698	1.00	0.00	H
ATOM	5578	HB2	ARG	A	382	19.309	38.322	42.372	1.00	0.00	H
ATOM	5579	CG	ARG	A	382	19.119	40.085	43.393	1.00	0.00	C
ATOM	5580	HG1	ARG	A	382	19.222	40.570	42.524	1.00	0.00	H
ATOM	5581	HG2	ARG	A	382	20.006	40.030	43.851	1.00	0.00	H
ATOM	5582	CD	ARG	A	382	18.169	40.902	44.287	1.00	0.00	C
ATOM	5583	HD1	ARG	A	382	18.589	41.789	44.481	1.00	0.00	H
ATOM	5584	HD2	ARG	A	382	18.024	40.407	45.144	1.00	0.00	H
ATOM	5585	NE	ARG	A	382	16.837	41.175	43.722	1.00	0.00	N
ATOM	5586	HE	ARG	A	382	16.723	40.883	42.773	1.00	0.00	H
ATOM	5587	CZ	ARG	A	382	15.764	41.741	44.254	1.00	0.00	C
ATOM	5588	NH1	ARG	A	382	15.823	42.168	45.486	1.00	0.00	N
ATOM	5589	HH11	ARG	A	382	16.668	42.065	46.011	1.00	0.00	H
ATOM	5590	HH12	ARG	A	382	15.023	42.598	45.905	1.00	0.00	H
ATOM	5591	NH2	ARG	A	382	14.630	41.883	43.678	1.00	0.00	N
ATOM	5592	HH21	ARG	A	382	14.499	41.552	42.744	1.00	0.00	H
ATOM	5593	HH22	ARG	A	382	13.875	42.325	44.162	1.00	0.00	H
ATOM	5594	C	ARG	A	382	17.677	37.903	45.276	1.00	0.00	C
ATOM	5595	O	ARG	A	382	18.033	38.277	46.412	1.00	0.00	O
ATOM	5596	N	GLN	A	383	16.359	37.742	45.099	1.00	0.00	N
ATOM	5597	H	GLN	A	383	16.095	37.501	44.165	1.00	0.00	H
ATOM	5598	CA	GLN	A	383	15.299	37.851	45.981	1.00	0.00	C
ATOM	5599	HA	GLN	A	383	15.438	38.749	46.398	1.00	0.00	H
ATOM	5600	CB	GLN	A	383	13.983	37.906	45.199	1.00	0.00	C
ATOM	5601	HB1	GLN	A	383	13.902	37.083	44.636	1.00	0.00	H
ATOM	5602	HB2	GLN	A	383	13.986	38.714	44.610	1.00	0.00	H
ATOM	5603	CG	GLN	A	383	12.784	37.983	46.127	1.00	0.00	C
ATOM	5604	HG1	GLN	A	383	12.814	37.186	46.730	1.00	0.00	H
ATOM	5605	HG2	GLN	A	383	11.959	37.944	45.563	1.00	0.00	H
ATOM	5606	CD	GLN	A	383	12.665	39.196	47.002	1.00	0.00	C
ATOM	5607	OE1	GLN	A	383	13.069	40.320	46.701	1.00	0.00	O
ATOM	5608	NE2	GLN	A	383	12.097	39.024	48.207	1.00	0.00	N
ATOM	5609	HE21	GLN	A	383	11.776	38.118	48.484	1.00	0.00	H
ATOM	5610	HE22	GLN	A	383	11.995	39.802	48.827	1.00	0.00	H
ATOM	5611	C	GLN	A	383	15.393	36.857	47.159	1.00	0.00	C
ATOM	5612	O	GLN	A	383	15.277	37.257	48.315	1.00	0.00	O
ATOM	5613	N	GLU	A	384	15.845	35.631	46.957	1.00	0.00	N

ATOM	5614	H	GLU	A	384	16.221	35.383	46.064	1.00	0.00	H
ATOM	5615	CA	GLU	A	384	15.801	34.632	48.028	1.00	0.00	C
ATOM	5616	HA	GLU	A	384	15.018	34.817	48.622	1.00	0.00	H
ATOM	5617	CB	GLU	A	384	15.576	33.306	47.399	1.00	0.00	C
ATOM	5618	HB1	GLU	A	384	15.600	32.631	48.137	1.00	0.00	H
ATOM	5619	HB2	GLU	A	384	16.344	33.149	46.779	1.00	0.00	H
ATOM	5620	CG	GLU	A	384	14.277	33.106	46.611	1.00	0.00	C
ATOM	5621	HG1	GLU	A	384	14.339	32.267	46.070	1.00	0.00	H
ATOM	5622	HG2	GLU	A	384	14.131	33.886	46.002	1.00	0.00	H
ATOM	5623	CD	GLU	A	384	13.112	33.000	47.651	1.00	0.00	C
ATOM	5624	OE1	GLU	A	384	12.331	33.988	47.752	1.00	0.00	O
ATOM	5625	OE2	GLU	A	384	13.038	32.146	48.543	1.00	0.00	O
ATOM	5626	C	GLU	A	384	17.149	34.683	48.843	1.00	0.00	C
ATOM	5627	O	GLU	A	384	17.339	33.959	49.786	1.00	0.00	O
ATOM	5628	N	LEU	A	385	18.032	35.574	48.427	1.00	0.00	N
ATOM	5629	H	LEU	A	385	17.926	35.934	47.500	1.00	0.00	H
ATOM	5630	CA	LEU	A	385	19.166	36.077	49.232	1.00	0.00	C
ATOM	5631	HA	LEU	A	385	19.288	35.435	49.989	1.00	0.00	H
ATOM	5632	CB	LEU	A	385	20.418	36.151	48.342	1.00	0.00	C
ATOM	5633	HB1	LEU	A	385	21.125	36.654	48.839	1.00	0.00	H
ATOM	5634	HB2	LEU	A	385	20.179	36.648	47.508	1.00	0.00	H
ATOM	5635	CG	LEU	A	385	21.015	34.826	47.924	1.00	0.00	C
ATOM	5636	HG	LEU	A	385	20.292	34.290	47.487	1.00	0.00	H
ATOM	5637	CD1	LEU	A	385	22.144	35.068	46.958	1.00	0.00	C
ATOM	5638	HD11	LEU	A	385	22.539	34.192	46.681	1.00	0.00	H
ATOM	5639	HD12	LEU	A	385	21.796	35.546	46.152	1.00	0.00	H
ATOM	5640	HD13	LEU	A	385	22.847	35.626	47.399	1.00	0.00	H
ATOM	5641	CD2	LEU	A	385	21.558	34.056	49.122	1.00	0.00	C
ATOM	5642	HD21	LEU	A	385	21.945	33.187	48.812	1.00	0.00	H
ATOM	5643	HD22	LEU	A	385	22.270	34.596	49.571	1.00	0.00	H
ATOM	5644	HD23	LEU	A	385	20.816	33.879	49.769	1.00	0.00	H
ATOM	5645	C	LEU	A	385	18.875	37.416	49.854	1.00	0.00	C
ATOM	5646	O	LEU	A	385	19.724	37.952	50.678	1.00	0.00	O
ATOM	5647	N	SER	A	386	17.690	37.936	49.634	1.00	0.00	N
ATOM	5648	H	SER	A	386	17.018	37.402	49.121	1.00	0.00	H
ATOM	5649	CA	SER	A	386	17.314	39.274	50.113	1.00	0.00	C
ATOM	5650	HA	SER	A	386	16.436	39.444	49.665	1.00	0.00	H
ATOM	5651	CB	SER	A	386	16.974	39.313	51.561	1.00	0.00	C
ATOM	5652	HB1	SER	A	386	16.794	40.263	51.815	1.00	0.00	H
ATOM	5653	HB2	SER	A	386	17.761	38.974	52.077	1.00	0.00	H
ATOM	5654	OG	SER	A	386	15.840	38.552	52.001	1.00	0.00	O
ATOM	5655	HG	SER	A	386	15.725	38.666	52.988	1.00	0.00	H
ATOM	5656	C	SER	A	386	18.359	40.364	49.695	1.00	0.00	C
ATOM	5657	O	SER	A	386	18.663	41.205	50.497	1.00	0.00	O
ATOM	5658	N	LEU	A	387	18.974	40.230	48.500	1.00	0.00	N
ATOM	5659	H	LEU	A	387	18.768	39.443	47.919	1.00	0.00	H
ATOM	5660	CA	LEU	A	387	19.926	41.217	48.060	1.00	0.00	C
ATOM	5661	HA	LEU	A	387	20.368	41.379	48.942	1.00	0.00	H
ATOM	5662	CB	LEU	A	387	20.922	40.688	46.927	1.00	0.00	C
ATOM	5663	HB1	LEU	A	387	21.540	41.435	46.682	1.00	0.00	H
ATOM	5664	HB2	LEU	A	387	20.377	40.431	46.129	1.00	0.00	H
ATOM	5665	CG	LEU	A	387	21.813	39.457	47.287	1.00	0.00	C
ATOM	5666	HG	LEU	A	387	21.194	38.735	47.594	1.00	0.00	H
ATOM	5667	CD1	LEU	A	387	22.610	38.989	46.069	1.00	0.00	C

ATOM	5668	HD11	LEU	A	387	23.173	38.202	46.321	1.00	0.00	H
ATOM	5669	HD12	LEU	A	387	21.979	38.728	45.338	1.00	0.00	H
ATOM	5670	HD13	LEU	A	387	23.199	39.732	45.751	1.00	0.00	H
ATOM	5671	CD2	LEU	A	387	22.796	39.731	48.424	1.00	0.00	C
ATOM	5672	HD21	LEU	A	387	23.334	38.908	48.605	1.00	0.00	H
ATOM	5673	HD22	LEU	A	387	23.407	40.478	48.163	1.00	0.00	H
ATOM	5674	HD23	LEU	A	387	22.290	39.986	49.248	1.00	0.00	H
ATOM	5675	C	LEU	A	387	19.288	42.516	47.568	1.00	0.00	C
ATOM	5676	O	LEU	A	387	18.312	42.438	46.859	1.00	0.00	O
ATOM	5677	N	PRO	A	388	19.971	43.649	47.880	1.00	0.00	N
ATOM	5678	CD	PRO	A	388	21.107	43.801	48.807	1.00	0.00	C
ATOM	5679	HD1	PRO	A	388	21.974	43.716	48.315	1.00	0.00	H
ATOM	5680	HD2	PRO	A	388	21.064	43.111	49.529	1.00	0.00	H
ATOM	5681	CG	PRO	A	388	20.899	45.217	49.358	1.00	0.00	C
ATOM	5682	HG1	PRO	A	388	21.782	45.679	49.437	1.00	0.00	H
ATOM	5683	HG2	PRO	A	388	20.468	45.166	50.259	1.00	0.00	H
ATOM	5684	CB	PRO	A	388	20.016	46.020	48.447	1.00	0.00	C
ATOM	5685	HB1	PRO	A	388	20.572	46.650	47.905	1.00	0.00	H
ATOM	5686	HB2	PRO	A	388	19.354	46.539	48.988	1.00	0.00	H
ATOM	5687	CA	PRO	A	388	19.302	44.988	47.538	1.00	0.00	C
ATOM	5688	HA	PRO	A	388	18.313	44.914	47.663	1.00	0.00	H
ATOM	5689	C	PRO	A	388	19.433	45.408	46.095	1.00	0.00	C
ATOM	5690	O	PRO	A	388	20.532	45.405	45.508	1.00	0.00	O
ATOM	5691	N	TYR	A	389	18.231	45.566	45.448	1.00	0.00	N
ATOM	5692	H	TYR	A	389	17.380	45.417	45.952	1.00	0.00	H
ATOM	5693	CA	TYR	A	389	18.167	45.961	43.986	1.00	0.00	C
ATOM	5694	HA	TYR	A	389	18.929	45.465	43.570	1.00	0.00	H
ATOM	5695	CB	TYR	A	389	16.835	45.480	43.410	1.00	0.00	C
ATOM	5696	HB1	TYR	A	389	16.092	45.994	43.839	1.00	0.00	H
ATOM	5697	HB2	TYR	A	389	16.727	44.507	43.612	1.00	0.00	H
ATOM	5698	CG	TYR	A	389	16.686	45.638	41.956	1.00	0.00	C
ATOM	5699	CD1	TYR	A	389	15.389	45.954	41.360	1.00	0.00	C
ATOM	5700	HD1	TYR	A	389	14.568	46.066	41.920	1.00	0.00	H
ATOM	5701	CE1	TYR	A	389	15.351	46.096	39.937	1.00	0.00	C
ATOM	5702	HE1	TYR	A	389	14.511	46.427	39.508	1.00	0.00	H
ATOM	5703	CZ	TYR	A	389	16.428	45.794	39.139	1.00	0.00	C
ATOM	5704	OH	TYR	A	389	16.312	45.911	37.767	1.00	0.00	O
ATOM	5705	HH	TYR	A	389	17.178	45.656	37.336	1.00	0.00	H
ATOM	5706	CE2	TYR	A	389	17.607	45.408	39.701	1.00	0.00	C
ATOM	5707	HE2	TYR	A	389	18.389	45.208	39.111	1.00	0.00	H
ATOM	5708	CD2	TYR	A	389	17.746	45.285	41.105	1.00	0.00	C
ATOM	5709	HD2	TYR	A	389	18.603	44.945	41.492	1.00	0.00	H
ATOM	5710	C	TYR	A	389	18.412	47.502	43.658	1.00	0.00	C
ATOM	5711	O	TYR	A	389	18.679	47.826	42.484	1.00	0.00	O
ATOM	5712	N	LEU	A	390	18.249	48.336	44.662	1.00	0.00	N
ATOM	5713	H	LEU	A	390	17.820	47.952	45.480	1.00	0.00	H
ATOM	5714	CA	LEU	A	390	18.608	49.734	44.740	1.00	0.00	C
ATOM	5715	HA	LEU	A	390	18.257	50.238	43.951	1.00	0.00	H
ATOM	5716	CB	LEU	A	390	17.896	50.328	45.982	1.00	0.00	C
ATOM	5717	HB1	LEU	A	390	18.282	49.876	46.786	1.00	0.00	H
ATOM	5718	HB2	LEU	A	390	16.926	50.097	45.903	1.00	0.00	H
ATOM	5719	CG	LEU	A	390	17.992	51.866	46.220	1.00	0.00	C
ATOM	5720	HG	LEU	A	390	17.452	52.038	47.044	1.00	0.00	H
ATOM	5721	CD1	LEU	A	390	19.400	52.288	46.573	1.00	0.00	C

ATOM	5722	HD11	LEU	A	390	19.425	53.277	46.718	1.00	0.00	H
ATOM	5723	HD12	LEU	A	390	19.688	51.821	47.409	1.00	0.00	H
ATOM	5724	HD13	LEU	A	390	20.018	52.045	45.825	1.00	0.00	H
ATOM	5725	CD2	LEU	A	390	17.226	52.808	45.271	1.00	0.00	C
ATOM	5726	HD21	LEU	A	390	17.382	53.757	45.546	1.00	0.00	H
ATOM	5727	HD22	LEU	A	390	17.551	52.675	44.335	1.00	0.00	H
ATOM	5728	HD23	LEU	A	390	16.248	52.604	45.317	1.00	0.00	H
ATOM	5729	C	LEU	A	390	20.194	49.798	44.734	1.00	0.00	C
ATOM	5730	O	LEU	A	390	20.807	49.192	45.628	1.00	0.00	O
ATOM	5731	N	PRO	A	391	20.844	50.529	43.794	1.00	0.00	N
ATOM	5732	CD	PRO	A	391	20.227	51.183	42.650	1.00	0.00	C
ATOM	5733	HD1	PRO	A	391	19.480	51.759	42.981	1.00	0.00	H
ATOM	5734	HD2	PRO	A	391	19.861	50.477	42.043	1.00	0.00	H
ATOM	5735	CG	PRO	A	391	21.274	52.031	41.917	1.00	0.00	C
ATOM	5736	HG1	PRO	A	391	21.260	52.985	42.218	1.00	0.00	H
ATOM	5737	HG2	PRO	A	391	21.158	51.993	40.924	1.00	0.00	H
ATOM	5738	CB	PRO	A	391	22.543	51.307	42.372	1.00	0.00	C
ATOM	5739	HB1	PRO	A	391	23.307	51.952	42.375	1.00	0.00	H
ATOM	5740	HB2	PRO	A	391	22.741	50.559	41.738	1.00	0.00	H
ATOM	5741	CA	PRO	A	391	22.305	50.744	43.815	1.00	0.00	C
ATOM	5742	HA	PRO	A	391	22.806	49.904	44.021	1.00	0.00	H
ATOM	5743	C	PRO	A	391	22.776	51.657	44.970	1.00	0.00	C
ATOM	5744	O	PRO	A	391	22.632	52.901	44.826	1.00	0.00	O
ATOM	5745	N	SER	A	392	23.265	51.038	46.068	1.00	0.00	N
ATOM	5746	H	SER	A	392	23.345	50.041	46.078	1.00	0.00	H
ATOM	5747	CA	SER	A	392	23.681	51.811	47.247	1.00	0.00	C
ATOM	5748	HA	SER	A	392	23.506	52.757	46.976	1.00	0.00	H
ATOM	5749	CB	SER	A	392	22.841	51.348	48.400	1.00	0.00	C
ATOM	5750	HB1	SER	A	392	22.982	50.371	48.563	1.00	0.00	H
ATOM	5751	HB2	SER	A	392	21.873	51.518	48.215	1.00	0.00	H
ATOM	5752	OG	SER	A	392	23.182	52.037	49.585	1.00	0.00	O
ATOM	5753	HG	SER	A	392	22.609	51.711	50.337	1.00	0.00	H
ATOM	5754	C	SER	A	392	25.171	51.778	47.637	1.00	0.00	C
ATOM	5755	O	SER	A	392	25.763	50.701	47.692	1.00	0.00	O
ATOM	5756	N	GLY	A	393	25.694	52.937	47.979	1.00	0.00	N
ATOM	5757	H	GLY	A	393	25.171	53.770	47.800	1.00	0.00	H
ATOM	5758	CA	GLY	A	393	27.018	53.058	48.614	1.00	0.00	C
ATOM	5759	HA1	GLY	A	393	27.119	52.270	49.221	1.00	0.00	H
ATOM	5760	HA2	GLY	A	393	27.001	53.897	49.157	1.00	0.00	H
ATOM	5761	C	GLY	A	393	28.226	53.108	47.660	1.00	0.00	C
ATOM	5762	O	GLY	A	393	28.186	52.781	46.447	1.00	0.00	O
ATOM	5763	N	THR	A	394	29.397	53.463	48.206	1.00	0.00	N
ATOM	5764	H	THR	A	394	29.397	53.854	49.127	1.00	0.00	H
ATOM	5765	CA	THR	A	394	30.702	53.300	47.498	1.00	0.00	C
ATOM	5766	HA	THR	A	394	30.546	52.747	46.680	1.00	0.00	H
ATOM	5767	CB	THR	A	394	31.248	54.642	47.064	1.00	0.00	C
ATOM	5768	HB	THR	A	394	31.421	55.137	47.916	1.00	0.00	H
ATOM	5769	CG2	THR	A	394	32.467	54.511	46.126	1.00	0.00	C
ATOM	5770	HG21	THR	A	394	32.789	55.422	45.869	1.00	0.00	H
ATOM	5771	HG22	THR	A	394	33.199	54.019	46.598	1.00	0.00	H
ATOM	5772	HG23	THR	A	394	32.202	54.007	45.304	1.00	0.00	H
ATOM	5773	OG1	THR	A	394	30.330	55.413	46.360	1.00	0.00	O
ATOM	5774	HG1	THR	A	394	30.752	56.283	46.104	1.00	0.00	H
ATOM	5775	C	THR	A	394	31.681	52.576	48.380	1.00	0.00	C

ATOM	5776	O	THR	A	394	31.037	50.972	47.639	1.00	0.00	O
ATOM	5777	N	PRO	A	395	32.711	52.257	48.296	1.00	0.00	N
ATOM	5778	CD	PRO	A	395	33.293	53.475	48.809	1.00	0.00	C
ATOM	5779	HD1	PRO	A	395	32.651	53.904	49.445	1.00	0.00	H
ATOM	5780	HD2	PRO	A	395	33.476	54.098	48.048	1.00	0.00	H
ATOM	5781	CG	PRO	A	395	34.595	53.044	49.518	1.00	0.00	C
ATOM	5782	HG1	PRO	A	395	34.427	52.835	50.481	1.00	0.00	H
ATOM	5783	HG2	PRO	A	395	35.294	53.756	49.448	1.00	0.00	H
ATOM	5784	CB	PRO	A	395	35.023	51.785	48.756	1.00	0.00	C
ATOM	5785	HB1	PRO	A	395	35.605	51.195	49.316	1.00	0.00	H
ATOM	5786	HB2	PRO	A	395	35.498	52.012	47.906	1.00	0.00	H
ATOM	5787	CA	PRO	A	395	33.627	51.154	48.496	1.00	0.00	C
ATOM	5788	HA	PRO	A	395	33.644	50.599	47.665	1.00	0.00	H
ATOM	5789	C	PRO	A	395	33.123	50.190	49.589	1.00	0.00	C
ATOM	5790	O	PRO	A	395	32.162	49.485	49.478	1.00	0.00	O
ATOM	5791	N	GLU	A	396	33.814	50.076	50.747	1.00	0.00	N
ATOM	5792	H	GLU	A	396	34.720	50.500	50.749	1.00	0.00	H
ATOM	5793	CA	GLU	A	396	33.431	49.432	51.975	1.00	0.00	C
ATOM	5794	HA	GLU	A	396	33.246	48.529	51.587	1.00	0.00	H
ATOM	5795	CB	GLU	A	396	34.565	49.416	53.044	1.00	0.00	C
ATOM	5796	HB1	GLU	A	396	35.378	49.023	52.614	1.00	0.00	H
ATOM	5797	HB2	GLU	A	396	34.264	48.825	53.792	1.00	0.00	H
ATOM	5798	CG	GLU	A	396	35.004	50.736	53.681	1.00	0.00	C
ATOM	5799	HG1	GLU	A	396	35.284	50.542	54.621	1.00	0.00	H
ATOM	5800	HG2	GLU	A	396	34.213	51.348	53.689	1.00	0.00	H
ATOM	5801	CD	GLU	A	396	36.139	51.487	53.026	1.00	0.00	C
ATOM	5802	OE1	GLU	A	396	35.993	51.773	51.800	1.00	0.00	O
ATOM	5803	OE2	GLU	A	396	37.160	51.831	53.688	1.00	0.00	O
ATOM	5804	C	GLU	A	396	32.147	49.943	52.623	1.00	0.00	C
ATOM	5805	O	GLU	A	396	31.603	49.151	53.386	1.00	0.00	O
ATOM	5806	N	LEU	A	397	31.500	51.126	52.247	1.00	0.00	N
ATOM	5807	H	LEU	A	397	31.871	51.605	51.452	1.00	0.00	H
ATOM	5808	CA	LEU	A	397	30.323	51.743	52.904	1.00	0.00	C
ATOM	5809	HA	LEU	A	397	30.222	51.175	53.721	1.00	0.00	H
ATOM	5810	CB	LEU	A	397	30.660	53.206	53.251	1.00	0.00	C
ATOM	5811	HB1	LEU	A	397	29.863	53.570	53.733	1.00	0.00	H
ATOM	5812	HB2	LEU	A	397	30.768	53.683	52.379	1.00	0.00	H
ATOM	5813	CG	LEU	A	397	31.924	53.538	54.123	1.00	0.00	C
ATOM	5814	HG	LEU	A	397	32.665	53.136	53.585	1.00	0.00	H
ATOM	5815	CD1	LEU	A	397	32.044	55.074	54.343	1.00	0.00	C
ATOM	5816	HD11	LEU	A	397	32.852	55.269	54.899	1.00	0.00	H
ATOM	5817	HD12	LEU	A	397	32.130	55.532	53.458	1.00	0.00	H
ATOM	5818	HD13	LEU	A	397	31.227	55.408	54.813	1.00	0.00	H
ATOM	5819	CD2	LEU	A	397	31.941	52.946	55.593	1.00	0.00	C
ATOM	5820	HD21	LEU	A	397	32.786	53.219	56.052	1.00	0.00	H
ATOM	5821	HD22	LEU	A	397	31.156	53.298	56.103	1.00	0.00	H
ATOM	5822	HD23	LEU	A	397	31.891	51.948	55.551	1.00	0.00	H
ATOM	5823	C	LEU	A	397	28.949	51.680	52.087	1.00	0.00	C
ATOM	5824	O	LEU	A	397	28.807	51.440	50.912	1.00	0.00	O
ATOM	5825	N	GLU	A	398	27.916	51.837	52.895	1.00	0.00	N
ATOM	5826	H	GLU	A	398	28.096	51.940	53.873	1.00	0.00	H
ATOM	5827	CA	GLU	A	398	26.480	51.870	52.429	1.00	0.00	C
ATOM	5828	HA	GLU	A	398	26.456	51.307	51.603	1.00	0.00	H
ATOM	5829	CB	GLU	A	398	25.567	51.272	53.505	1.00	0.00	C

ATOM	5830	HB1	GLU	A	398	24.617	51.372	53.208	1.00	0.00	H
ATOM	5831	HB2	GLU	A	398	25.705	51.778	54.356	1.00	0.00	H
ATOM	5832	CG	GLU	A	398	25.884	49.755	53.741	1.00	0.00	C
ATOM	5833	HG1	GLU	A	398	26.828	49.669	54.059	1.00	0.00	H
ATOM	5834	HG2	GLU	A	398	25.778	49.265	52.876	1.00	0.00	H
ATOM	5835	CD	GLU	A	398	24.931	49.112	54.806	1.00	0.00	C
ATOM	5836	OE1	GLU	A	398	25.291	48.077	55.394	1.00	0.00	O
ATOM	5837	OE2	GLU	A	398	23.756	49.576	55.000	1.00	0.00	O
ATOM	5838	C	GLU	A	398	25.990	53.286	52.038	1.00	0.00	C
ATOM	5839	O	GLU	A	398	24.791	53.523	51.694	1.00	0.00	O
ATOM	5840	N	VAL	A	399	26.909	54.275	52.074	1.00	0.00	N
ATOM	5841	H	VAL	A	399	27.825	54.007	52.373	1.00	0.00	H
ATOM	5842	CA	VAL	A	399	26.744	55.646	51.743	1.00	0.00	C
ATOM	5843	HA	VAL	A	399	25.927	55.798	51.187	1.00	0.00	H
ATOM	5844	CB	VAL	A	399	26.573	56.452	53.142	1.00	0.00	C
ATOM	5845	HB	VAL	A	399	26.377	57.416	52.961	1.00	0.00	H
ATOM	5846	CG1	VAL	A	399	25.436	55.911	53.891	1.00	0.00	C
ATOM	5847	HG11	VAL	A	399	25.335	56.411	54.751	1.00	0.00	H
ATOM	5848	HG12	VAL	A	399	24.602	56.010	53.348	1.00	0.00	H
ATOM	5849	HG13	VAL	A	399	25.595	54.943	54.085	1.00	0.00	H
ATOM	5850	CG2	VAL	A	399	27.824	56.394	54.056	1.00	0.00	C
ATOM	5851	HG21	VAL	A	399	27.648	56.909	54.895	1.00	0.00	H
ATOM	5852	HG22	VAL	A	399	28.025	55.442	54.285	1.00	0.00	H
ATOM	5853	HG23	VAL	A	399	28.605	56.794	53.577	1.00	0.00	H
ATOM	5854	C	VAL	A	399	27.923	55.992	50.812	1.00	0.00	C
ATOM	5855	O	VAL	A	399	28.834	55.169	50.619	1.00	0.00	O
ATOM	5856	N	PRO	A	400	28.024	57.251	50.315	1.00	0.00	N
ATOM	5857	CD	PRO	A	400	27.015	58.296	50.422	1.00	0.00	C
ATOM	5858	HD1	PRO	A	400	27.237	58.900	51.187	1.00	0.00	H
ATOM	5859	HD2	PRO	A	400	26.117	57.882	50.573	1.00	0.00	H
ATOM	5860	CG	PRO	A	400	27.063	59.043	49.096	1.00	0.00	C
ATOM	5861	HG1	PRO	A	400	26.713	59.976	49.180	1.00	0.00	H
ATOM	5862	HG2	PRO	A	400	26.560	58.562	48.378	1.00	0.00	H
ATOM	5863	CB	PRO	A	400	28.545	59.030	48.836	1.00	0.00	C
ATOM	5864	HB1	PRO	A	400	28.971	59.820	49.276	1.00	0.00	H
ATOM	5865	HB2	PRO	A	400	28.714	59.066	47.851	1.00	0.00	H
ATOM	5866	CA	PRO	A	400	29.075	57.693	49.448	1.00	0.00	C
ATOM	5867	HA	PRO	A	400	29.245	56.943	48.808	1.00	0.00	H
ATOM	5868	C	PRO	A	400	30.413	57.867	50.105	1.00	0.00	C
ATOM	5869	O	PRO	A	400	30.529	58.348	51.239	1.00	0.00	O
ATOM	5870	N	LYS	A	401	31.451	57.607	49.289	1.00	0.00	N
ATOM	5871	H	LYS	A	401	31.272	57.157	48.414	1.00	0.00	H
ATOM	5872	CA	LYS	A	401	32.798	57.947	49.620	1.00	0.00	C
ATOM	5873	HA	LYS	A	401	32.698	58.277	50.559	1.00	0.00	H
ATOM	5874	CB	LYS	A	401	33.770	56.784	49.691	1.00	0.00	C
ATOM	5875	HB1	LYS	A	401	34.664	57.156	49.940	1.00	0.00	H
ATOM	5876	HB2	LYS	A	401	33.820	56.380	48.778	1.00	0.00	H
ATOM	5877	CG	LYS	A	401	33.407	55.666	50.701	1.00	0.00	C
ATOM	5878	HG1	LYS	A	401	32.511	55.282	50.479	1.00	0.00	H
ATOM	5879	HG2	LYS	A	401	33.391	56.036	51.630	1.00	0.00	H
ATOM	5880	CD	LYS	A	401	34.455	54.569	50.618	1.00	0.00	C
ATOM	5881	HD1	LYS	A	401	34.507	54.251	49.671	1.00	0.00	H
ATOM	5882	HD2	LYS	A	401	34.170	53.814	51.208	1.00	0.00	H
ATOM	5883	CE	LYS	A	401	35.843	55.040	51.056	1.00	0.00	C

ATOM	5884	HE1	LYS	A	401	35.835	55.240	52.036	1.00	0.00	H
ATOM	5885	HE2	LYS	A	401	36.092	55.866	50.550	1.00	0.00	H
ATOM	5886	NZ	LYS	A	401	36.875	53.997	50.795	1.00	0.00	N
ATOM	5887	HZ1	LYS	A	401	37.770	54.332	51.091	1.00	0.00	H
ATOM	5888	HZ2	LYS	A	401	36.646	53.167	51.303	1.00	0.00	H
ATOM	5889	HZ3	LYS	A	401	36.902	53.793	49.817	1.00	0.00	H
ATOM	5890	C	LYS	A	401	33.306	59.013	48.638	1.00	0.00	C
ATOM	5891	O	LYS	A	401	33.368	58.734	47.446	1.00	0.00	O
ATOM	5892	N	PRO	A	402	33.620	60.227	49.092	1.00	0.00	N
ATOM	5893	CD	PRO	A	402	33.489	60.727	50.474	1.00	0.00	C
ATOM	5894	HD1	PRO	A	402	34.133	60.231	51.056	1.00	0.00	H
ATOM	5895	HD2	PRO	A	402	32.555	60.557	50.789	1.00	0.00	H
ATOM	5896	CG	PRO	A	402	33.796	62.234	50.501	1.00	0.00	C
ATOM	5897	HG1	PRO	A	402	34.741	62.399	50.782	1.00	0.00	H
ATOM	5898	HG2	PRO	A	402	33.175	62.712	51.122	1.00	0.00	H
ATOM	5899	CB	PRO	A	402	33.594	62.732	49.115	1.00	0.00	C
ATOM	5900	HB1	PRO	A	402	34.245	63.464	48.917	1.00	0.00	H
ATOM	5901	HB2	PRO	A	402	32.662	63.078	49.009	1.00	0.00	H
ATOM	5902	CA	PRO	A	402	33.853	61.461	48.228	1.00	0.00	C
ATOM	5903	HA	PRO	A	402	33.190	61.426	47.481	1.00	0.00	H
ATOM	5904	C	PRO	A	402	35.323	61.475	47.579	1.00	0.00	C
ATOM	5905	O	PRO	A	402	35.583	62.200	46.607	1.00	0.00	O
ATOM	5906	N	GLY	A	403	36.176	60.586	47.956	1.00	0.00	N
ATOM	5907	H	GLY	A	403	35.887	59.960	48.680	1.00	0.00	H
ATOM	5908	CA	GLY	A	403	37.528	60.405	47.426	1.00	0.00	C
ATOM	5909	HA1	GLY	A	403	38.040	59.835	48.068	1.00	0.00	H
ATOM	5910	HA2	GLY	A	403	37.960	61.305	47.361	1.00	0.00	H
ATOM	5911	C	GLY	A	403	37.500	59.742	46.052	1.00	0.00	C
ATOM	5912	O	GLY	A	403	36.499	59.158	45.690	1.00	0.00	O
ATOM	5913	N	GLN	A	404	38.566	59.968	45.275	1.00	0.00	N
ATOM	5914	H	GLN	A	404	39.312	60.477	45.705	1.00	0.00	H
ATOM	5915	CA	GLN	A	404	38.795	59.570	43.868	1.00	0.00	C
ATOM	5916	HA	GLN	A	404	38.123	60.039	43.295	1.00	0.00	H
ATOM	5917	CB	GLN	A	404	40.173	60.027	43.409	1.00	0.00	C
ATOM	5918	HB1	GLN	A	404	40.852	59.580	43.991	1.00	0.00	H
ATOM	5919	HB2	GLN	A	404	40.224	61.017	43.537	1.00	0.00	H
ATOM	5920	CG	GLN	A	404	40.456	59.670	41.886	1.00	0.00	C
ATOM	5921	HG1	GLN	A	404	39.835	60.208	41.316	1.00	0.00	H
ATOM	5922	HG2	GLN	A	404	40.272	58.696	41.752	1.00	0.00	H
ATOM	5923	CD	GLN	A	404	41.855	59.917	41.339	1.00	0.00	C
ATOM	5924	OE1	GLN	A	404	42.636	60.632	41.992	1.00	0.00	O
ATOM	5925	NE2	GLN	A	404	42.170	59.492	40.154	1.00	0.00	N
ATOM	5926	HE21	GLN	A	404	41.494	59.003	39.602	1.00	0.00	H
ATOM	5927	HE22	GLN	A	404	43.088	59.653	39.793	1.00	0.00	H
ATOM	5928	C	GLN	A	404	38.588	58.058	43.722	1.00	0.00	C
ATOM	5929	O	GLN	A	404	39.132	57.259	44.525	1.00	0.00	O
ATOM	5930	N	PRO	A	405	37.843	57.598	42.684	1.00	0.00	N
ATOM	5931	CD	PRO	A	405	37.100	58.432	41.689	1.00	0.00	C
ATOM	5932	HD1	PRO	A	405	37.705	59.123	41.293	1.00	0.00	H
ATOM	5933	HD2	PRO	A	405	36.320	58.881	42.125	1.00	0.00	H
ATOM	5934	CG	PRO	A	405	36.627	57.506	40.632	1.00	0.00	C
ATOM	5935	HG1	PRO	A	405	37.269	57.468	39.867	1.00	0.00	H
ATOM	5936	HG2	PRO	A	405	35.723	57.768	40.296	1.00	0.00	H
ATOM	5937	CB	PRO	A	405	36.585	56.211	41.387	1.00	0.00	C

ATOM	5938	HB1	PRO	A	405	36.735	55.453	40.752	1.00	0.00	H
ATOM	5939	HB2	PRO	A	405	35.690	56.110	41.822	1.00	0.00	H
ATOM	5940	CA	PRO	A	405	37.646	56.187	42.438	1.00	0.00	C
ATOM	5941	HA	PRO	A	405	37.385	55.757	43.302	1.00	0.00	H
ATOM	5942	C	PRO	A	405	38.905	55.403	41.935	1.00	0.00	C
ATOM	5943	O	PRO	A	405	39.865	56.012	41.443	1.00	0.00	O
ATOM	5944	N	SER	A	406	38.718	54.081	41.844	1.00	0.00	N
ATOM	5945	H	SER	A	406	37.894	53.714	42.276	1.00	0.00	H
ATOM	5946	CA	SER	A	406	39.589	53.123	41.181	1.00	0.00	C
ATOM	5947	HA	SER	A	406	40.502	53.408	41.472	1.00	0.00	H
ATOM	5948	CB	SER	A	406	39.172	51.722	41.607	1.00	0.00	C
ATOM	5949	HB1	SER	A	406	39.724	51.025	41.149	1.00	0.00	H
ATOM	5950	HB2	SER	A	406	38.204	51.559	41.416	1.00	0.00	H
ATOM	5951	OG	SER	A	406	39.355	51.560	42.969	1.00	0.00	O
ATOM	5952	HG	SER	A	406	39.078	50.637	43.235	1.00	0.00	H
ATOM	5953	C	SER	A	406	39.479	53.129	39.647	1.00	0.00	C
ATOM	5954	O	SER	A	406	40.194	52.431	38.916	1.00	0.00	O
ATOM	5955	N	GLU	A	407	38.620	53.950	39.016	1.00	0.00	N
ATOM	5956	H	GLU	A	407	37.989	54.476	39.587	1.00	0.00	H
ATOM	5957	CA	GLU	A	407	38.527	54.142	37.561	1.00	0.00	C
ATOM	5958	HA	GLU	A	407	39.463	54.218	37.217	1.00	0.00	H
ATOM	5959	CB	GLU	A	407	37.981	52.892	36.838	1.00	0.00	C
ATOM	5960	HB1	GLU	A	407	38.536	52.107	37.113	1.00	0.00	H
ATOM	5961	HB2	GLU	A	407	38.074	53.037	35.853	1.00	0.00	H
ATOM	5962	CG	GLU	A	407	36.517	52.549	37.121	1.00	0.00	C
ATOM	5963	HG1	GLU	A	407	36.225	51.822	36.499	1.00	0.00	H
ATOM	5964	HG2	GLU	A	407	35.955	53.362	36.969	1.00	0.00	H
ATOM	5965	CD	GLU	A	407	36.325	52.077	38.557	1.00	0.00	C
ATOM	5966	OE1	GLU	A	407	35.849	52.915	39.358	1.00	0.00	O
ATOM	5967	OE2	GLU	A	407	36.469	50.862	38.786	1.00	0.00	O
ATOM	5968	C	GLU	A	407	37.892	55.496	37.229	1.00	0.00	C
ATOM	5969	O	GLU	A	407	36.962	55.955	37.965	1.00	0.00	O
ATOM	5970	N	PRO	A	408	38.286	56.216	36.172	1.00	0.00	N
ATOM	5971	CD	PRO	A	408	39.245	55.797	35.097	1.00	0.00	C
ATOM	5972	HD1	PRO	A	408	38.748	55.385	34.333	1.00	0.00	H
ATOM	5973	HD2	PRO	A	408	39.903	55.137	35.460	1.00	0.00	H
ATOM	5974	CG	PRO	A	408	39.951	57.063	34.652	1.00	0.00	C
ATOM	5975	HG1	PRO	A	408	40.332	56.977	33.731	1.00	0.00	H
ATOM	5976	HG2	PRO	A	408	40.673	57.334	35.288	1.00	0.00	H
ATOM	5977	CB	PRO	A	408	38.752	58.009	34.698	1.00	0.00	C
ATOM	5978	HB1	PRO	A	408	38.239	57.942	33.842	1.00	0.00	H
ATOM	5979	HB2	PRO	A	408	39.071	58.949	34.823	1.00	0.00	H
ATOM	5980	CA	PRO	A	408	37.861	57.570	35.903	1.00	0.00	C
ATOM	5981	HA	PRO	A	408	37.949	58.150	36.713	1.00	0.00	H
ATOM	5982	C	PRO	A	408	36.423	57.677	35.712	1.00	0.00	C
ATOM	5983	O	PRO	A	408	35.807	56.894	34.964	1.00	0.00	O
ATOM	5984	N	GLU	A	409	35.823	58.644	36.451	1.00	0.00	N
ATOM	5985	H	GLU	A	409	36.412	59.266	36.967	1.00	0.00	H
ATOM	5986	CA	GLU	A	409	34.333	58.849	36.550	1.00	0.00	C
ATOM	5987	HA	GLU	A	409	34.243	59.457	37.339	1.00	0.00	H
ATOM	5988	CB	GLU	A	409	33.740	59.693	35.386	1.00	0.00	C
ATOM	5989	HB1	GLU	A	409	32.824	59.995	35.649	1.00	0.00	H
ATOM	5990	HB2	GLU	A	409	33.679	59.113	34.574	1.00	0.00	H
ATOM	5991	CG	GLU	A	409	34.556	60.938	35.013	1.00	0.00	C

ATOM	5992	HG1	GLU	A	409	33.955	61.567	34.521	1.00	0.00	H
ATOM	5993	HG2	GLU	A	409	35.302	60.652	34.412	1.00	0.00	H
ATOM	5994	CD	GLU	A	409	35.164	61.692	36.205	1.00	0.00	C
ATOM	5995	OE1	GLU	A	409	34.465	61.995	37.206	1.00	0.00	O
ATOM	5996	OE2	GLU	A	409	36.359	62.116	36.140	1.00	0.00	O
ATOM	5997	C	GLU	A	409	33.537	57.530	36.930	1.00	0.00	C
ATOM	5998	O	GLU	A	409	32.340	57.342	36.552	1.00	0.00	O
ATOM	5999	N	HIS	A	410	34.081	56.603	37.692	1.00	0.00	N
ATOM	6000	H	HIS	A	410	35.013	56.750	38.022	1.00	0.00	H
ATOM	6001	CA	HIS	A	410	33.392	55.363	38.084	1.00	0.00	C
ATOM	6002	HA	HIS	A	410	34.136	54.894	38.561	1.00	0.00	H
ATOM	6003	CB	HIS	A	410	32.194	55.666	38.994	1.00	0.00	C
ATOM	6004	HB1	HIS	A	410	31.851	54.800	39.357	1.00	0.00	H
ATOM	6005	HB2	HIS	A	410	31.483	56.100	38.441	1.00	0.00	H
ATOM	6006	CG	HIS	A	410	32.543	56.603	40.183	1.00	0.00	C
ATOM	6007	ND1	HIS	A	410	32.402	57.985	40.294	1.00	0.00	N
ATOM	6008	CE1	HIS	A	410	32.728	58.380	41.579	1.00	0.00	C
ATOM	6009	HE1	HIS	A	410	32.710	59.313	41.938	1.00	0.00	H
ATOM	6010	NE2	HIS	A	410	33.070	57.282	42.245	1.00	0.00	N
ATOM	6011	HE2	HIS	A	410	33.392	57.266	43.192	1.00	0.00	H
ATOM	6012	CD2	HIS	A	410	32.915	56.145	41.430	1.00	0.00	C
ATOM	6013	HD2	HIS	A	410	33.048	55.192	41.702	1.00	0.00	H
ATOM	6014	C	HIS	A	410	33.006	54.384	36.992	1.00	0.00	C
ATOM	6015	O	HIS	A	410	32.466	53.359	37.325	1.00	0.00	O
ATOM	6016	N	GLY	A	411	33.261	54.577	35.742	1.00	0.00	N
ATOM	6017	H	GLY	A	411	33.617	55.477	35.488	1.00	0.00	H
ATOM	6018	CA	GLY	A	411	33.082	53.613	34.662	1.00	0.00	C
ATOM	6019	HA1	GLY	A	411	32.103	53.439	34.554	1.00	0.00	H
ATOM	6020	HA2	GLY	A	411	33.545	52.764	34.918	1.00	0.00	H
ATOM	6021	C	GLY	A	411	33.643	54.115	33.370	1.00	0.00	C
ATOM	6022	O	GLY	A	411	33.194	55.184	32.852	1.00	0.00	O
ATOM	6023	N	PRO	A	412	34.539	53.319	32.687	1.00	0.00	N
ATOM	6024	CD	PRO	A	412	35.446	52.256	33.149	1.00	0.00	C
ATOM	6025	HD1	PRO	A	412	34.935	51.399	33.208	1.00	0.00	H
ATOM	6026	HD2	PRO	A	412	35.794	52.500	34.054	1.00	0.00	H
ATOM	6027	CG	PRO	A	412	36.588	52.076	32.223	1.00	0.00	C
ATOM	6028	HG1	PRO	A	412	36.844	51.111	32.168	1.00	0.00	H
ATOM	6029	HG2	PRO	A	412	37.374	52.612	32.531	1.00	0.00	H
ATOM	6030	CB	PRO	A	412	35.992	52.612	30.875	1.00	0.00	C
ATOM	6031	HB1	PRO	A	412	35.557	51.881	30.350	1.00	0.00	H
ATOM	6032	HB2	PRO	A	412	36.693	53.053	30.314	1.00	0.00	H
ATOM	6033	CA	PRO	A	412	34.962	53.623	31.304	1.00	0.00	C
ATOM	6034	HA	PRO	A	412	35.326	54.546	31.426	1.00	0.00	H
ATOM	6035	C	PRO	A	412	33.873	53.717	30.188	1.00	0.00	C
ATOM	6036	O	PRO	A	412	32.717	53.473	30.412	1.00	0.00	O
ATOM	6037	N	SER	A	413	34.289	54.191	29.016	1.00	0.00	N
ATOM	6038	H	SER	A	413	35.218	54.560	29.020	1.00	0.00	H
ATOM	6039	CA	SER	A	413	33.590	54.262	27.688	1.00	0.00	C
ATOM	6040	HA	SER	A	413	33.086	53.421	27.491	1.00	0.00	H
ATOM	6041	CB	SER	A	413	32.624	55.515	27.784	1.00	0.00	C
ATOM	6042	HB1	SER	A	413	33.095	56.330	28.120	1.00	0.00	H
ATOM	6043	HB2	SER	A	413	31.824	55.332	28.356	1.00	0.00	H
ATOM	6044	OG	SER	A	413	32.163	55.795	26.483	1.00	0.00	O
ATOM	6045	HG	SER	A	413	31.549	56.584	26.510	1.00	0.00	H

ATOM	6046	C	SER	A	413	34.655	54.410	26.576	1.00	0.00	C
ATOM	6047	O	SER	A	413	35.646	55.177	26.708	1.00	0.00	O
ATOM	6048	N	PRO	A	414	34.534	53.599	25.489	1.00	0.00	N
ATOM	6049	CD	PRO	A	414	34.198	52.148	25.692	1.00	0.00	C
ATOM	6050	HD1	PRO	A	414	33.207	52.017	25.709	1.00	0.00	H
ATOM	6051	HD2	PRO	A	414	34.593	51.813	26.547	1.00	0.00	H
ATOM	6052	CG	PRO	A	414	34.775	51.411	24.559	1.00	0.00	C
ATOM	6053	HG1	PRO	A	414	34.180	50.652	24.296	1.00	0.00	H
ATOM	6054	HG2	PRO	A	414	35.681	51.059	24.795	1.00	0.00	H
ATOM	6055	CB	PRO	A	414	34.853	52.459	23.470	1.00	0.00	C
ATOM	6056	HB1	PRO	A	414	33.968	52.574	23.018	1.00	0.00	H
ATOM	6057	HB2	PRO	A	414	35.546	52.219	22.790	1.00	0.00	H
ATOM	6058	CA	PRO	A	414	35.256	53.719	24.300	1.00	0.00	C
ATOM	6059	HA	PRO	A	414	36.221	53.737	24.562	1.00	0.00	H
ATOM	6060	C	PRO	A	414	35.002	55.073	23.567	1.00	0.00	C
ATOM	6061	O	PRO	A	414	35.522	55.239	22.488	1.00	0.00	O
ATOM	6062	N	GLY	A	415	34.110	55.926	24.040	1.00	0.00	N
ATOM	6063	H	GLY	A	415	33.594	55.704	24.867	1.00	0.00	H
ATOM	6064	CA	GLY	A	415	33.878	57.205	23.345	1.00	0.00	C
ATOM	6065	HA1	GLY	A	415	34.757	57.525	22.992	1.00	0.00	H
ATOM	6066	HA2	GLY	A	415	33.531	57.859	24.017	1.00	0.00	H
ATOM	6067	C	GLY	A	415	32.884	57.089	22.193	1.00	0.00	C
ATOM	6068	O	GLY	A	415	32.751	57.988	21.374	1.00	0.00	O
ATOM	6069	N	GLN	A	416	32.203	55.924	22.169	1.00	0.00	N
ATOM	6070	H	GLN	A	416	32.320	55.268	22.914	1.00	0.00	H
ATOM	6071	CA	GLN	A	416	31.283	55.606	21.049	1.00	0.00	C
ATOM	6072	HA	GLN	A	416	31.507	56.254	20.321	1.00	0.00	H
ATOM	6073	CB	GLN	A	416	31.498	54.124	20.621	1.00	0.00	C
ATOM	6074	HB1	GLN	A	416	30.777	53.876	19.974	1.00	0.00	H
ATOM	6075	HB2	GLN	A	416	31.428	53.548	21.435	1.00	0.00	H
ATOM	6076	CG	GLN	A	416	32.862	53.839	19.950	1.00	0.00	C
ATOM	6077	HG1	GLN	A	416	33.544	53.675	20.663	1.00	0.00	H
ATOM	6078	HG2	GLN	A	416	33.129	54.637	19.410	1.00	0.00	H
ATOM	6079	CD	GLN	A	416	32.843	52.630	19.027	1.00	0.00	C
ATOM	6080	OE1	GLN	A	416	33.030	52.708	17.819	1.00	0.00	O
ATOM	6081	NE2	GLN	A	416	32.623	51.481	19.681	1.00	0.00	N
ATOM	6082	HE21	GLN	A	416	32.493	51.487	20.672	1.00	0.00	H
ATOM	6083	HE22	GLN	A	416	32.589	50.618	19.177	1.00	0.00	H
ATOM	6084	C	GLN	A	416	29.820	55.821	21.422	1.00	0.00	C
ATOM	6085	O	GLN	A	416	29.477	56.109	22.575	1.00	0.00	O
ATOM	6086	N	ASP	A	417	28.948	55.628	20.364	1.00	0.00	N
ATOM	6087	H	ASP	A	417	29.336	55.500	19.451	1.00	0.00	H
ATOM	6088	CA	ASP	A	417	27.524	55.601	20.502	1.00	0.00	C
ATOM	6089	HA	ASP	A	417	27.395	55.898	21.448	1.00	0.00	H
ATOM	6090	CB	ASP	A	417	26.885	56.571	19.480	1.00	0.00	C
ATOM	6091	HB1	ASP	A	417	26.935	56.143	18.578	1.00	0.00	H
ATOM	6092	HB2	ASP	A	417	27.423	57.414	19.479	1.00	0.00	H
ATOM	6093	CG	ASP	A	417	25.400	56.944	19.758	1.00	0.00	C
ATOM	6094	OD1	ASP	A	417	24.836	56.607	20.828	1.00	0.00	O
ATOM	6095	OD2	ASP	A	417	24.774	57.522	18.873	1.00	0.00	O
ATOM	6096	C	ASP	A	417	26.920	54.188	20.336	1.00	0.00	C
ATOM	6097	O	ASP	A	417	27.484	53.369	19.570	1.00	0.00	O
ATOM	6098	N	THR	A	418	25.819	53.957	20.989	1.00	0.00	N
ATOM	6099	H	THR	A	418	25.462	54.710	21.542	1.00	0.00	H

ATOM	6100	CA	THR	A	418	25.046	52.714	21.002	1.00	0.00	C
ATOM	6101	HA	THR	A	418	25.758	52.032	21.173	1.00	0.00	H
ATOM	6102	CB	THR	A	418	23.963	52.762	22.142	1.00	0.00	C
ATOM	6103	HB	THR	A	418	23.070	53.108	21.853	1.00	0.00	H
ATOM	6104	CG2	THR	A	418	23.780	51.362	22.733	1.00	0.00	C
ATOM	6105	HG21	THR	A	418	23.091	51.393	23.458	1.00	0.00	H
ATOM	6106	HG22	THR	A	418	23.480	50.734	22.015	1.00	0.00	H
ATOM	6107	HG23	THR	A	418	24.649	51.044	23.112	1.00	0.00	H
ATOM	6108	OG1	THR	A	418	24.332	53.718	23.102	1.00	0.00	O
ATOM	6109	HG1	THR	A	418	23.642	53.747	23.826	1.00	0.00	H
ATOM	6110	C	THR	A	418	24.361	52.376	19.636	1.00	0.00	C
ATOM	6111	O	THR	A	418	23.358	51.681	19.683	1.00	0.00	O
ATOM	6112	N	PHE	A	419	24.891	52.751	18.489	1.00	0.00	N
ATOM	6113	H	PHE	A	419	25.790	53.188	18.525	1.00	0.00	H
ATOM	6114	CA	PHE	A	419	24.269	52.582	17.136	1.00	0.00	C
ATOM	6115	HA	PHE	A	419	23.340	52.951	17.174	1.00	0.00	H
ATOM	6116	CB	PHE	A	419	25.154	53.391	16.099	1.00	0.00	C
ATOM	6117	HB1	PHE	A	419	26.039	52.948	15.957	1.00	0.00	H
ATOM	6118	HB2	PHE	A	419	25.298	54.332	16.405	1.00	0.00	H
ATOM	6119	CG	PHE	A	419	24.417	53.415	14.803	1.00	0.00	C
ATOM	6120	CD1	PHE	A	419	24.737	52.527	13.794	1.00	0.00	C
ATOM	6121	HD1	PHE	A	419	25.305	51.726	13.982	1.00	0.00	H
ATOM	6122	CE1	PHE	A	419	24.259	52.765	12.524	1.00	0.00	C
ATOM	6123	HE1	PHE	A	419	24.473	52.127	11.784	1.00	0.00	H
ATOM	6124	CZ	PHE	A	419	23.487	53.878	12.269	1.00	0.00	C
ATOM	6125	HZ	PHE	A	419	23.146	54.040	11.343	1.00	0.00	H
ATOM	6126	CE2	PHE	A	419	23.179	54.771	13.273	1.00	0.00	C
ATOM	6127	HE2	PHE	A	419	22.622	55.576	13.071	1.00	0.00	H
ATOM	6128	CD2	PHE	A	419	23.637	54.556	14.552	1.00	0.00	C
ATOM	6129	HD2	PHE	A	419	23.419	55.198	15.287	1.00	0.00	H
ATOM	6130	C	PHE	A	419	24.072	51.083	16.767	1.00	0.00	C
ATOM	6131	O	PHE	A	419	23.000	50.768	16.260	1.00	0.00	O
ATOM	6132	N	CYS	A	420	25.006	50.199	17.091	1.00	0.00	N
ATOM	6133	H	CYS	A	420	25.768	50.448	17.689	1.00	0.00	H
ATOM	6134	CA	CYS	A	420	24.885	48.852	16.547	1.00	0.00	C
ATOM	6135	HA	CYS	A	420	24.449	49.023	15.664	1.00	0.00	H
ATOM	6136	CB	CYS	A	420	26.261	48.236	16.327	1.00	0.00	C
ATOM	6137	HB1	CYS	A	420	26.093	47.335	15.928	1.00	0.00	H
ATOM	6138	HB2	CYS	A	420	26.658	48.128	17.239	1.00	0.00	H
ATOM	6139	SG	CYS	A	420	27.259	49.289	15.260	1.00	0.00	S
ATOM	6140	C	CYS	A	420	23.968	47.894	17.397	1.00	0.00	C
ATOM	6141	O	CYS	A	420	23.887	46.744	16.969	1.00	0.00	O
ATOM	6142	N	GLN	A	421	23.310	48.378	18.489	1.00	0.00	N
ATOM	6143	H	GLN	A	421	23.531	49.314	18.762	1.00	0.00	H
ATOM	6144	CA	GLN	A	421	22.345	47.700	19.282	1.00	0.00	C
ATOM	6145	HA	GLN	A	421	22.837	46.896	19.616	1.00	0.00	H
ATOM	6146	CB	GLN	A	421	21.892	48.628	20.426	1.00	0.00	C
ATOM	6147	HB1	GLN	A	421	21.418	49.416	20.032	1.00	0.00	H
ATOM	6148	HB2	GLN	A	421	22.700	48.940	20.925	1.00	0.00	H
ATOM	6149	CG	GLN	A	421	20.941	47.918	21.413	1.00	0.00	C
ATOM	6150	HG1	GLN	A	421	20.336	47.306	20.904	1.00	0.00	H
ATOM	6151	HG2	GLN	A	421	20.394	48.604	21.894	1.00	0.00	H
ATOM	6152	CD	GLN	A	421	21.678	47.100	22.442	1.00	0.00	C
ATOM	6153	OE1	GLN	A	421	22.501	46.244	22.195	1.00	0.00	O

ATOM	6154	NE2	GLN	A	421	21.188	47.131	23.666	1.00	0.00	N
ATOM	6155	HE21	GLN	A	421	20.373	47.676	23.865	1.00	0.00	H
ATOM	6156	HE22	GLN	A	421	21.631	46.610	24.395	1.00	0.00	H
ATOM	6157	C	GLN	A	421	21.145	47.219	18.464	1.00	0.00	C
ATOM	6158	O	GLN	A	421	20.403	48.061	17.932	1.00	0.00	O
ATOM	6159	N	GLY	A	422	20.885	45.927	18.381	1.00	0.00	N
ATOM	6160	H	GLY	A	422	21.488	45.292	18.864	1.00	0.00	H
ATOM	6161	CA	GLY	A	422	19.772	45.377	17.628	1.00	0.00	C
ATOM	6162	HA1	GLY	A	422	18.964	45.815	18.021	1.00	0.00	H
ATOM	6163	HA2	GLY	A	422	19.770	44.404	17.860	1.00	0.00	H
ATOM	6164	C	GLY	A	422	19.692	45.509	16.078	1.00	0.00	C
ATOM	6165	O	GLY	A	422	18.721	44.968	15.478	1.00	0.00	O
ATOM	6166	N	LYS	A	423	20.758	46.089	15.461	1.00	0.00	N
ATOM	6167	H	LYS	A	423	21.381	46.623	16.033	1.00	0.00	H
ATOM	6168	CA	LYS	A	423	21.075	45.999	14.021	1.00	0.00	C
ATOM	6169	HA	LYS	A	423	20.197	45.999	13.542	1.00	0.00	H
ATOM	6170	CB	LYS	A	423	21.844	47.210	13.548	1.00	0.00	C
ATOM	6171	HB1	LYS	A	423	22.108	47.073	12.593	1.00	0.00	H
ATOM	6172	HB2	LYS	A	423	22.665	47.310	14.109	1.00	0.00	H
ATOM	6173	CG	LYS	A	423	20.972	48.477	13.670	1.00	0.00	C
ATOM	6174	HG1	LYS	A	423	20.713	48.609	14.627	1.00	0.00	H
ATOM	6175	HG2	LYS	A	423	20.148	48.366	13.114	1.00	0.00	H
ATOM	6176	CD	LYS	A	423	21.761	49.713	13.177	1.00	0.00	C
ATOM	6177	HD1	LYS	A	423	21.968	49.620	12.203	1.00	0.00	H
ATOM	6178	HD2	LYS	A	423	22.614	49.802	13.692	1.00	0.00	H
ATOM	6179	CE	LYS	A	423	20.860	50.955	13.416	1.00	0.00	C
ATOM	6180	HE1	LYS	A	423	19.932	50.752	13.103	1.00	0.00	H
ATOM	6181	HE2	LYS	A	423	21.225	51.729	12.898	1.00	0.00	H
ATOM	6182	NZ	LYS	A	423	20.764	51.381	14.843	1.00	0.00	N
ATOM	6183	HZ1	LYS	A	423	20.169	52.182	14.912	1.00	0.00	H
ATOM	6184	HZ2	LYS	A	423	20.383	50.635	15.389	1.00	0.00	H
ATOM	6185	HZ3	LYS	A	423	21.675	51.612	15.184	1.00	0.00	H
ATOM	6186	C	LYS	A	423	21.771	44.698	13.680	1.00	0.00	C
ATOM	6187	O	LYS	A	423	22.464	44.054	14.464	1.00	0.00	O
ATOM	6188	N	ALA	A	424	21.525	44.338	12.389	1.00	0.00	N
ATOM	6189	H	ALA	A	424	20.728	44.761	11.958	1.00	0.00	H
ATOM	6190	CA	ALA	A	424	22.298	43.394	11.547	1.00	0.00	C
ATOM	6191	HA	ALA	A	424	22.476	42.548	12.051	1.00	0.00	H
ATOM	6192	CB	ALA	A	424	21.466	43.017	10.276	1.00	0.00	C
ATOM	6193	HB1	ALA	A	424	21.991	42.380	9.711	1.00	0.00	H
ATOM	6194	HB2	ALA	A	424	20.608	42.586	10.555	1.00	0.00	H
ATOM	6195	HB3	ALA	A	424	21.268	43.844	9.750	1.00	0.00	H
ATOM	6196	C	ALA	A	424	23.639	44.083	11.228	1.00	0.00	C
ATOM	6197	O	ALA	A	424	23.742	45.274	11.051	1.00	0.00	O
ATOM	6198	N	ASP	A	425	24.682	43.260	11.146	1.00	0.00	N
ATOM	6199	H	ASP	A	425	24.531	42.286	11.312	1.00	0.00	H
ATOM	6200	CA	ASP	A	425	26.035	43.706	10.826	1.00	0.00	C
ATOM	6201	HA	ASP	A	425	26.232	44.457	11.456	1.00	0.00	H
ATOM	6202	CB	ASP	A	425	27.031	42.561	11.056	1.00	0.00	C
ATOM	6203	HB1	ASP	A	425	27.936	42.868	10.760	1.00	0.00	H
ATOM	6204	HB2	ASP	A	425	26.746	41.781	10.500	1.00	0.00	H
ATOM	6205	CG	ASP	A	425	27.135	42.098	12.513	1.00	0.00	C
ATOM	6206	OD1	ASP	A	425	26.738	42.809	13.466	1.00	0.00	O
ATOM	6207	OD2	ASP	A	425	27.645	40.980	12.738	1.00	0.00	O

ATOM	6208	C	ASP	A	425	26.112	44.230	9.382	1.00	0.00	C
ATOM	6209	O	ASP	A	425	25.401	43.760	8.490	1.00	0.00	O
ATOM	6210	N	GLY	A	426	26.967	45.224	9.151	1.00	0.00	N
ATOM	6211	H	GLY	A	426	27.551	45.533	9.902	1.00	0.00	H
ATOM	6212	CA	GLY	A	426	27.094	45.884	7.858	1.00	0.00	C
ATOM	6213	HA1	GLY	A	426	26.183	45.912	7.446	1.00	0.00	H
ATOM	6214	HA2	GLY	A	426	27.699	45.322	7.294	1.00	0.00	H
ATOM	6215	C	GLY	A	426	27.654	47.300	7.947	1.00	0.00	C
ATOM	6216	O	GLY	A	426	27.890	47.847	9.027	1.00	0.00	O
ATOM	6217	N	LEU	A	427	27.844	47.906	6.775	1.00	0.00	N
ATOM	6218	H	LEU	A	427	27.765	47.358	5.942	1.00	0.00	H
ATOM	6219	CA	LEU	A	427	28.161	49.325	6.640	1.00	0.00	C
ATOM	6220	HA	LEU	A	427	28.834	49.526	7.352	1.00	0.00	H
ATOM	6221	CB	LEU	A	427	28.765	49.596	5.250	1.00	0.00	C
ATOM	6222	HB1	LEU	A	427	28.899	50.583	5.159	1.00	0.00	H
ATOM	6223	HB2	LEU	A	427	28.110	49.283	4.563	1.00	0.00	H
ATOM	6224	CG	LEU	A	427	30.113	48.906	4.970	1.00	0.00	C
ATOM	6225	HG	LEU	A	427	29.985	47.923	5.100	1.00	0.00	H
ATOM	6226	CD1	LEU	A	427	30.560	49.208	3.541	1.00	0.00	C
ATOM	6227	HD11	LEU	A	427	31.435	48.758	3.363	1.00	0.00	H
ATOM	6228	HD12	LEU	A	427	29.874	48.868	2.898	1.00	0.00	H
ATOM	6229	HD13	LEU	A	427	30.663	50.196	3.426	1.00	0.00	H
ATOM	6230	CD2	LEU	A	427	31.205	49.385	5.925	1.00	0.00	C
ATOM	6231	HD21	LEU	A	427	32.062	48.916	5.713	1.00	0.00	H
ATOM	6232	HD22	LEU	A	427	31.330	50.372	5.821	1.00	0.00	H
ATOM	6233	HD23	LEU	A	427	30.937	49.181	6.867	1.00	0.00	H
ATOM	6234	C	LEU	A	427	26.911	50.185	6.876	1.00	0.00	C
ATOM	6235	O	LEU	A	427	25.813	49.849	6.409	1.00	0.00	O
ATOM	6236	N	TYR	A	428	27.113	51.340	7.510	1.00	0.00	N
ATOM	6237	H	TYR	A	428	28.037	51.524	7.845	1.00	0.00	H
ATOM	6238	CA	TYR	A	428	26.094	52.361	7.757	1.00	0.00	C
ATOM	6239	HA	TYR	A	428	25.407	52.263	7.037	1.00	0.00	H
ATOM	6240	CB	TYR	A	428	25.456	52.121	9.137	1.00	0.00	C
ATOM	6241	HB1	TYR	A	428	24.788	52.848	9.295	1.00	0.00	H
ATOM	6242	HB2	TYR	A	428	26.182	52.180	9.823	1.00	0.00	H
ATOM	6243	CG	TYR	A	428	24.739	50.803	9.362	1.00	0.00	C
ATOM	6244	CD1	TYR	A	428	25.279	49.795	10.183	1.00	0.00	C
ATOM	6245	HD1	TYR	A	428	26.178	49.916	10.605	1.00	0.00	H
ATOM	6246	CE1	TYR	A	428	24.541	48.613	10.405	1.00	0.00	C
ATOM	6247	HE1	TYR	A	428	24.915	47.891	10.987	1.00	0.00	H
ATOM	6248	CZ	TYR	A	428	23.274	48.450	9.805	1.00	0.00	C
ATOM	6249	OH	TYR	A	428	22.628	47.202	9.854	1.00	0.00	O
ATOM	6250	HH	TYR	A	428	21.748	47.264	9.384	1.00	0.00	H
ATOM	6251	CE2	TYR	A	428	22.778	49.440	8.980	1.00	0.00	C
ATOM	6252	HE2	TYR	A	428	21.896	49.310	8.526	1.00	0.00	H
ATOM	6253	CD2	TYR	A	428	23.484	50.603	8.776	1.00	0.00	C
ATOM	6254	HD2	TYR	A	428	23.093	51.319	8.198	1.00	0.00	H
ATOM	6255	C	TYR	A	428	26.741	53.769	7.701	1.00	0.00	C
ATOM	6256	O	TYR	A	428	27.913	53.861	8.031	1.00	0.00	O
ATOM	6257	N	PRO	A	429	25.997	54.823	7.294	1.00	0.00	N
ATOM	6258	CD	PRO	A	429	24.669	54.725	6.663	1.00	0.00	C
ATOM	6259	HD1	PRO	A	429	23.967	54.554	7.354	1.00	0.00	H
ATOM	6260	HD2	PRO	A	429	24.658	53.988	5.987	1.00	0.00	H
ATOM	6261	CG	PRO	A	429	24.501	56.104	6.008	1.00	0.00	C

ATOM	6262	HG1	PRO	A	429	23.549	56.404	6.072	1.00	0.00	H
ATOM	6263	HG2	PRO	A	429	24.775	56.066	5.047	1.00	0.00	H
ATOM	6264	CB	PRO	A	429	25.413	57.066	6.782	1.00	0.00	C
ATOM	6265	HB1	PRO	A	429	24.894	57.534	7.497	1.00	0.00	H
ATOM	6266	HB2	PRO	A	429	25.809	57.742	6.161	1.00	0.00	H
ATOM	6267	CA	PRO	A	429	26.521	56.204	7.407	1.00	0.00	C
ATOM	6268	HA	PRO	A	429	27.418	56.255	6.968	1.00	0.00	H
ATOM	6269	C	PRO	A	429	26.721	56.642	8.845	1.00	0.00	C
ATOM	6270	O	PRO	A	429	26.105	56.104	9.756	1.00	0.00	O
ATOM	6271	N	ASN	A	430	27.605	57.648	9.007	1.00	0.00	N
ATOM	6272	H	ASN	A	430	28.060	58.059	8.217	1.00	0.00	H
ATOM	6273	CA	ASN	A	430	27.890	58.135	10.385	1.00	0.00	C
ATOM	6274	HA	ASN	A	430	27.739	57.386	11.030	1.00	0.00	H
ATOM	6275	CB	ASN	A	430	29.368	58.646	10.459	1.00	0.00	C
ATOM	6276	HB1	ASN	A	430	29.517	59.289	9.708	1.00	0.00	H
ATOM	6277	HB2	ASN	A	430	29.979	57.861	10.353	1.00	0.00	H
ATOM	6278	CG	ASN	A	430	29.824	59.348	11.686	1.00	0.00	C
ATOM	6279	OD1	ASN	A	430	29.139	59.432	12.670	1.00	0.00	O
ATOM	6280	ND2	ASN	A	430	31.023	59.887	11.759	1.00	0.00	N
ATOM	6281	HD21	ASN	A	430	31.646	59.828	10.979	1.00	0.00	H
ATOM	6282	HD22	ASN	A	430	31.311	60.355	12.594	1.00	0.00	H
ATOM	6283	C	ASN	A	430	26.936	59.293	10.670	1.00	0.00	C
ATOM	6284	O	ASN	A	430	26.987	60.271	9.961	1.00	0.00	O
ATOM	6285	N	PRO	A	431	26.138	59.363	11.726	1.00	0.00	N
ATOM	6286	CD	PRO	A	431	25.783	58.353	12.744	1.00	0.00	C
ATOM	6287	HD1	PRO	A	431	26.256	58.534	13.606	1.00	0.00	H
ATOM	6288	HD2	PRO	A	431	26.009	57.432	12.427	1.00	0.00	H
ATOM	6289	CG	PRO	A	431	24.266	58.573	12.856	1.00	0.00	C
ATOM	6290	HG1	PRO	A	431	23.937	58.299	13.759	1.00	0.00	H
ATOM	6291	HG2	PRO	A	431	23.782	58.052	12.153	1.00	0.00	H
ATOM	6292	CB	PRO	A	431	24.058	60.073	12.646	1.00	0.00	C
ATOM	6293	HB1	PRO	A	431	24.093	60.555	13.522	1.00	0.00	H
ATOM	6294	HB2	PRO	A	431	23.176	60.244	12.207	1.00	0.00	H
ATOM	6295	CA	PRO	A	431	25.219	60.518	11.731	1.00	0.00	C
ATOM	6296	HA	PRO	A	431	24.922	60.704	10.794	1.00	0.00	H
ATOM	6297	C	PRO	A	431	25.852	61.873	12.054	1.00	0.00	C
ATOM	6298	O	PRO	A	431	25.264	62.967	11.909	1.00	0.00	O
ATOM	6299	N	ARG	A	432	27.171	61.917	12.393	1.00	0.00	N
ATOM	6300	H	ARG	A	432	27.668	61.049	12.398	1.00	0.00	H
ATOM	6301	CA	ARG	A	432	27.944	63.145	12.759	1.00	0.00	C
ATOM	6302	HA	ARG	A	432	27.314	63.713	13.289	1.00	0.00	H
ATOM	6303	CB	ARG	A	432	29.170	62.640	13.488	1.00	0.00	C
ATOM	6304	HB1	ARG	A	432	29.728	62.147	12.820	1.00	0.00	H
ATOM	6305	HB2	ARG	A	432	28.853	62.004	14.192	1.00	0.00	H
ATOM	6306	CG	ARG	A	432	30.067	63.672	14.168	1.00	0.00	C
ATOM	6307	HG1	ARG	A	432	30.436	64.256	13.445	1.00	0.00	H
ATOM	6308	HG2	ARG	A	432	30.813	63.166	14.601	1.00	0.00	H
ATOM	6309	CD	ARG	A	432	29.380	64.593	15.260	1.00	0.00	C
ATOM	6310	HD1	ARG	A	432	28.670	65.141	14.819	1.00	0.00	H
ATOM	6311	HD2	ARG	A	432	30.072	65.198	15.653	1.00	0.00	H
ATOM	6312	NE	ARG	A	432	28.718	63.928	16.418	1.00	0.00	N
ATOM	6313	HE	ARG	A	432	27.812	63.534	16.265	1.00	0.00	H
ATOM	6314	CZ	ARG	A	432	29.223	63.819	17.608	1.00	0.00	C
ATOM	6315	NH1	ARG	A	432	30.324	64.412	18.001	1.00	0.00	N

ATOM	6316	HH11	ARG	A	432	30.832	64.994	17.366	1.00	0.00	H
ATOM	6317	HH12	ARG	A	432	30.655	64.283	18.936	1.00	0.00	H
ATOM	6318	NH2	ARG	A	432	28.538	63.330	18.625	1.00	0.00	N
ATOM	6319	HH21	ARG	A	432	27.594	63.027	18.493	1.00	0.00	H
ATOM	6320	HH22	ARG	A	432	28.963	63.262	19.528	1.00	0.00	H
ATOM	6321	C	ARG	A	432	28.432	64.030	11.605	1.00	0.00	C
ATOM	6322	O	ARG	A	432	28.497	65.244	11.735	1.00	0.00	O
ATOM	6323	N	GLU	A	433	28.801	63.362	10.489	1.00	0.00	N
ATOM	6324	H	GLU	A	433	28.455	62.437	10.333	1.00	0.00	H
ATOM	6325	CA	GLU	A	433	29.698	63.969	9.506	1.00	0.00	C
ATOM	6326	HA	GLU	A	433	29.465	64.929	9.348	1.00	0.00	H
ATOM	6327	CB	GLU	A	433	31.141	63.868	10.038	1.00	0.00	C
ATOM	6328	HB1	GLU	A	433	31.296	62.937	10.369	1.00	0.00	H
ATOM	6329	HB2	GLU	A	433	31.250	64.513	10.794	1.00	0.00	H
ATOM	6330	CG	GLU	A	433	32.193	64.190	8.960	1.00	0.00	C
ATOM	6331	HG1	GLU	A	433	32.074	63.565	8.188	1.00	0.00	H
ATOM	6332	HG2	GLU	A	433	33.107	64.071	9.348	1.00	0.00	H
ATOM	6333	CD	GLU	A	433	32.079	65.604	8.445	1.00	0.00	C
ATOM	6334	OE1	GLU	A	433	31.749	65.746	7.215	1.00	0.00	O
ATOM	6335	OE2	GLU	A	433	32.333	66.546	9.275	1.00	0.00	O
ATOM	6336	C	GLU	A	433	29.520	63.161	8.241	1.00	0.00	C
ATOM	6337	O	GLU	A	433	29.563	61.936	8.236	1.00	0.00	O
ATOM	6338	N	ARG	A	434	29.282	63.936	7.172	1.00	0.00	N
ATOM	6339	H	ARG	A	434	29.386	64.928	7.238	1.00	0.00	H
ATOM	6340	CA	ARG	A	434	28.868	63.321	5.903	1.00	0.00	C
ATOM	6341	HA	ARG	A	434	28.160	62.676	6.191	1.00	0.00	H
ATOM	6342	CB	ARG	A	434	28.288	64.359	4.921	1.00	0.00	C
ATOM	6343	HB1	ARG	A	434	28.187	63.936	4.021	1.00	0.00	H
ATOM	6344	HB2	ARG	A	434	28.917	65.134	4.856	1.00	0.00	H
ATOM	6345	CG	ARG	A	434	26.913	64.867	5.394	1.00	0.00	C
ATOM	6346	HG1	ARG	A	434	27.032	65.446	6.201	1.00	0.00	H
ATOM	6347	HG2	ARG	A	434	26.332	64.087	5.626	1.00	0.00	H
ATOM	6348	CD	ARG	A	434	26.246	65.683	4.275	1.00	0.00	C
ATOM	6349	HD1	ARG	A	434	26.132	65.121	3.455	1.00	0.00	H
ATOM	6350	HD2	ARG	A	434	26.798	66.487	4.054	1.00	0.00	H
ATOM	6351	NE	ARG	A	434	24.929	66.140	4.698	1.00	0.00	N
ATOM	6352	HE	ARG	A	434	24.638	65.965	5.639	1.00	0.00	H
ATOM	6353	CZ	ARG	A	434	24.083	66.794	3.863	1.00	0.00	C
ATOM	6354	NH1	ARG	A	434	24.417	67.099	2.586	1.00	0.00	N
ATOM	6355	HH11	ARG	A	434	25.315	66.841	2.229	1.00	0.00	H
ATOM	6356	HH12	ARG	A	434	23.766	67.582	2.001	1.00	0.00	H
ATOM	6357	NH2	ARG	A	434	22.864	67.153	4.322	1.00	0.00	N
ATOM	6358	HH21	ARG	A	434	22.600	66.936	5.262	1.00	0.00	H
ATOM	6359	HH22	ARG	A	434	22.226	67.636	3.722	1.00	0.00	H
ATOM	6360	C	ARG	A	434	29.960	62.511	5.239	1.00	0.00	C
ATOM	6361	O	ARG	A	434	29.720	61.449	4.681	1.00	0.00	O
ATOM	6362	N	SER	A	435	31.182	63.049	5.389	1.00	0.00	N
ATOM	6363	H	SER	A	435	31.297	63.910	5.885	1.00	0.00	H
ATOM	6364	CA	SER	A	435	32.348	62.359	4.817	1.00	0.00	C
ATOM	6365	HA	SER	A	435	31.977	61.823	4.059	1.00	0.00	H
ATOM	6366	CB	SER	A	435	33.361	63.422	4.361	1.00	0.00	C
ATOM	6367	HB1	SER	A	435	32.896	64.170	3.887	1.00	0.00	H
ATOM	6368	HB2	SER	A	435	34.046	63.018	3.755	1.00	0.00	H
ATOM	6369	OG	SER	A	435	34.052	63.982	5.487	1.00	0.00	O

ATOM	6370	HG	SER	A	435	34.705	64.670	5.169	1.00	0.00	H
ATOM	6371	C	SER	A	435	33.001	61.328	5.721	1.00	0.00	C
ATOM	6372	O	SER	A	435	34.209	61.132	5.739	1.00	0.00	O
ATOM	6373	N	SER	A	436	32.116	60.684	6.496	1.00	0.00	N
ATOM	6374	H	SER	A	436	31.151	60.946	6.473	1.00	0.00	H
ATOM	6375	CA	SER	A	436	32.558	59.597	7.377	1.00	0.00	C
ATOM	6376	HA	SER	A	436	33.349	59.131	6.981	1.00	0.00	H
ATOM	6377	CB	SER	A	436	32.967	60.230	8.730	1.00	0.00	C
ATOM	6378	HB1	SER	A	436	32.305	60.911	9.043	1.00	0.00	H
ATOM	6379	HB2	SER	A	436	33.877	60.643	8.693	1.00	0.00	H
ATOM	6380	OG	SER	A	436	33.017	59.278	9.684	1.00	0.00	O
ATOM	6381	HG	SER	A	436	33.281	59.692	10.555	1.00	0.00	H
ATOM	6382	C	SER	A	436	31.471	58.536	7.466	1.00	0.00	C
ATOM	6383	O	SER	A	436	30.272	58.755	7.179	1.00	0.00	O
ATOM	6384	N	PHE	A	437	31.931	57.333	7.845	1.00	0.00	N
ATOM	6385	H	PHE	A	437	32.891	57.212	8.098	1.00	0.00	H
ATOM	6386	CA	PHE	A	437	30.999	56.197	7.882	1.00	0.00	C
ATOM	6387	HA	PHE	A	437	30.122	56.610	8.127	1.00	0.00	H
ATOM	6388	CB	PHE	A	437	30.903	55.532	6.502	1.00	0.00	C
ATOM	6389	HB1	PHE	A	437	30.735	56.242	5.818	1.00	0.00	H
ATOM	6390	HB2	PHE	A	437	30.136	54.890	6.511	1.00	0.00	H
ATOM	6391	CG	PHE	A	437	32.147	54.769	6.089	1.00	0.00	C
ATOM	6392	CD1	PHE	A	437	33.266	55.452	5.574	1.00	0.00	C
ATOM	6393	HD1	PHE	A	437	33.239	56.446	5.469	1.00	0.00	H
ATOM	6394	CE1	PHE	A	437	34.421	54.739	5.208	1.00	0.00	C
ATOM	6395	HE1	PHE	A	437	35.216	55.227	4.848	1.00	0.00	H
ATOM	6396	CZ	PHE	A	437	34.459	53.341	5.347	1.00	0.00	C
ATOM	6397	HZ	PHE	A	437	35.277	52.832	5.078	1.00	0.00	H
ATOM	6398	CE2	PHE	A	437	33.343	52.660	5.865	1.00	0.00	C
ATOM	6399	HE2	PHE	A	437	33.370	51.666	5.968	1.00	0.00	H
ATOM	6400	CD2	PHE	A	437	32.191	53.372	6.238	1.00	0.00	C
ATOM	6401	HD2	PHE	A	437	31.401	52.884	6.610	1.00	0.00	H
ATOM	6402	C	PHE	A	437	31.271	55.205	8.989	1.00	0.00	C
ATOM	6403	O	PHE	A	437	32.397	55.026	9.465	1.00	0.00	O
ATOM	6404	N	TYR	A	438	30.164	54.624	9.439	1.00	0.00	N
ATOM	6405	H	TYR	A	438	29.289	54.866	9.020	1.00	0.00	H
ATOM	6406	CA	TYR	A	438	30.179	53.637	10.532	1.00	0.00	C
ATOM	6407	HA	TYR	A	438	30.959	53.826	11.128	1.00	0.00	H
ATOM	6408	CB	TYR	A	438	28.869	53.756	11.318	1.00	0.00	C
ATOM	6409	HB1	TYR	A	438	28.716	52.872	11.760	1.00	0.00	H
ATOM	6410	HB2	TYR	A	438	28.145	53.924	10.649	1.00	0.00	H
ATOM	6411	CG	TYR	A	438	28.721	54.825	12.406	1.00	0.00	C
ATOM	6412	CD1	TYR	A	438	27.451	55.012	12.940	1.00	0.00	C
ATOM	6413	HD1	TYR	A	438	26.663	54.557	12.525	1.00	0.00	H
ATOM	6414	CE1	TYR	A	438	27.268	55.861	14.091	1.00	0.00	C
ATOM	6415	HE1	TYR	A	438	26.371	55.933	14.526	1.00	0.00	H
ATOM	6416	CZ	TYR	A	438	28.391	56.608	14.611	1.00	0.00	C
ATOM	6417	OH	TYR	A	438	28.301	57.404	15.713	1.00	0.00	O
ATOM	6418	HH	TYR	A	438	29.190	57.823	15.895	1.00	0.00	H
ATOM	6419	CE2	TYR	A	438	29.584	56.437	13.944	1.00	0.00	C
ATOM	6420	HE2	TYR	A	438	30.376	56.969	14.244	1.00	0.00	H
ATOM	6421	CD2	TYR	A	438	29.745	55.556	12.866	1.00	0.00	C
ATOM	6422	HD2	TYR	A	438	30.642	55.471	12.432	1.00	0.00	H
ATOM	6423	C	TYR	A	438	30.271	52.232	9.897	1.00	0.00	C

ATOM	6424	O	TYR	A	438	29.930	51.989	8.735	1.00	0.00	O
ATOM	6425	N	SER	A	439	30.654	51.255	10.700	1.00	0.00	N
ATOM	6426	H	SER	A	439	30.915	51.498	11.634	1.00	0.00	H
ATOM	6427	CA	SER	A	439	30.724	49.851	10.323	1.00	0.00	C
ATOM	6428	HA	SER	A	439	30.070	49.620	9.603	1.00	0.00	H
ATOM	6429	CB	SER	A	439	32.143	49.606	9.793	1.00	0.00	C
ATOM	6430	HB1	SER	A	439	32.816	49.762	10.516	1.00	0.00	H
ATOM	6431	HB2	SER	A	439	32.335	50.213	9.022	1.00	0.00	H
ATOM	6432	OG	SER	A	439	32.347	48.289	9.330	1.00	0.00	O
ATOM	6433	HG	SER	A	439	33.286	48.194	8.999	1.00	0.00	H
ATOM	6434	C	SER	A	439	30.366	49.042	11.567	1.00	0.00	C
ATOM	6435	O	SER	A	439	30.984	49.225	12.610	1.00	0.00	O
ATOM	6436	N	CYS	A	440	29.300	48.246	11.517	1.00	0.00	N
ATOM	6437	H	CYS	A	440	28.765	48.226	10.672	1.00	0.00	H
ATOM	6438	CA	CYS	A	440	28.866	47.397	12.630	1.00	0.00	C
ATOM	6439	HA	CYS	A	440	29.339	47.719	13.450	1.00	0.00	H
ATOM	6440	CB	CYS	A	440	27.348	47.519	12.833	1.00	0.00	C
ATOM	6441	HB1	CYS	A	440	27.149	46.790	13.488	1.00	0.00	H
ATOM	6442	HB2	CYS	A	440	26.980	47.271	11.937	1.00	0.00	H
ATOM	6443	SG	CYS	A	440	26.688	49.118	13.401	1.00	0.00	S
ATOM	6444	C	CYS	A	440	29.245	45.946	12.319	1.00	0.00	C
ATOM	6445	O	CYS	A	440	28.850	45.439	11.270	1.00	0.00	O
ATOM	6446	N	ALA	A	441	29.955	45.258	13.217	1.00	0.00	N
ATOM	6447	H	ALA	A	441	30.320	45.740	14.013	1.00	0.00	H
ATOM	6448	CA	ALA	A	441	30.219	43.822	13.079	1.00	0.00	C
ATOM	6449	HA	ALA	A	441	29.438	43.452	12.576	1.00	0.00	H
ATOM	6450	CB	ALA	A	441	31.491	43.592	12.261	1.00	0.00	C
ATOM	6451	HB1	ALA	A	441	31.659	42.610	12.175	1.00	0.00	H
ATOM	6452	HB2	ALA	A	441	31.379	43.992	11.351	1.00	0.00	H
ATOM	6453	HB3	ALA	A	441	32.266	44.024	12.722	1.00	0.00	H
ATOM	6454	C	ALA	A	441	30.272	43.118	14.442	1.00	0.00	C
ATOM	6455	O	ALA	A	441	30.858	43.636	15.395	1.00	0.00	O
ATOM	6456	N	ALA	A	442	29.584	41.977	14.542	1.00	0.00	N
ATOM	6457	H	ALA	A	442	29.342	41.502	13.696	1.00	0.00	H
ATOM	6458	CA	ALA	A	442	29.162	41.377	15.803	1.00	0.00	C
ATOM	6459	HA	ALA	A	442	28.446	40.717	15.577	1.00	0.00	H
ATOM	6460	CB	ALA	A	442	30.324	40.586	16.421	1.00	0.00	C
ATOM	6461	HB1	ALA	A	442	30.027	40.178	17.284	1.00	0.00	H
ATOM	6462	HB2	ALA	A	442	30.608	39.863	15.791	1.00	0.00	H
ATOM	6463	HB3	ALA	A	442	31.094	41.202	16.590	1.00	0.00	H
ATOM	6464	C	ALA	A	442	28.489	42.408	16.734	1.00	0.00	C
ATOM	6465	O	ALA	A	442	28.728	42.440	17.917	1.00	0.00	O
ATOM	6466	N	GLY	A	443	27.735	43.374	16.224	1.00	0.00	N
ATOM	6467	H	GLY	A	443	27.597	43.385	15.234	1.00	0.00	H
ATOM	6468	CA	GLY	A	443	27.089	44.428	17.018	1.00	0.00	C
ATOM	6469	HA1	GLY	A	443	26.564	43.963	17.731	1.00	0.00	H
ATOM	6470	HA2	GLY	A	443	26.463	44.904	16.400	1.00	0.00	H
ATOM	6471	C	GLY	A	443	28.038	45.467	17.680	1.00	0.00	C
ATOM	6472	O	GLY	A	443	27.574	46.364	18.377	1.00	0.00	O
ATOM	6473	N	ARG	A	444	29.365	45.405	17.466	1.00	0.00	N
ATOM	6474	H	ARG	A	444	29.740	44.583	17.037	1.00	0.00	H
ATOM	6475	CA	ARG	A	444	30.297	46.497	17.836	1.00	0.00	C
ATOM	6476	HA	ARG	A	444	29.952	47.028	18.610	1.00	0.00	H
ATOM	6477	CB	ARG	A	444	31.592	45.799	18.230	1.00	0.00	C

```
ATOM   6478  HB1 ARG A 444      31.852  45.238  17.444  1.00  0.00           H
ATOM   6479  HB2 ARG A 444      31.361  45.206  19.001  1.00  0.00           H
ATOM   6480  CG  ARG A 444      32.848  46.643  18.643  1.00  0.00           C
ATOM   6481  HG1 ARG A 444      33.349  46.185  19.377  1.00  0.00           H
ATOM   6482  HG2 ARG A 444      32.570  47.554  18.947  1.00  0.00           H
ATOM   6483  CD  ARG A 444      33.772  46.811  17.505  1.00  0.00           C
ATOM   6484  HD1 ARG A 444      33.265  47.227  16.750  1.00  0.00           H
ATOM   6485  HD2 ARG A 444      34.097  45.906  17.230  1.00  0.00           H
ATOM   6486  NE  ARG A 444      34.952  47.667  17.815  1.00  0.00           N
ATOM   6487  HE  ARG A 444      34.782  48.565  18.221  1.00  0.00           H
ATOM   6488  CZ  ARG A 444      36.180  47.329  17.596  1.00  0.00           C
ATOM   6489  NH1 ARG A 444      36.618  46.112  17.483  1.00  0.00           N
ATOM   6490 HH11 ARG A 444      35.984  45.343  17.566  1.00  0.00           H
ATOM   6491 HH12 ARG A 444      37.589  45.944  17.313  1.00  0.00           H
ATOM   6492  NH2 ARG A 444      37.012  48.295  17.438  1.00  0.00           N
ATOM   6493 HH21 ARG A 444      36.694  49.242  17.488  1.00  0.00           H
ATOM   6494 HH22 ARG A 444      37.977  48.100  17.265  1.00  0.00           H
ATOM   6495  C   ARG A 444      30.427  47.527  16.705  1.00  0.00           C
ATOM   6496  O   ARG A 444      30.662  47.191  15.542  1.00  0.00           O
ATOM   6497  N   LEU A 445      30.327  48.799  17.111  1.00  0.00           N
ATOM   6498  H   LEU A 445      30.095  48.935  18.074  1.00  0.00           H
ATOM   6499  CA  LEU A 445      30.514  50.005  16.325  1.00  0.00           C
ATOM   6500  HA  LEU A 445      30.068  49.789  15.457  1.00  0.00           H
ATOM   6501  CB  LEU A 445      29.773  51.201  16.938  1.00  0.00           C
ATOM   6502  HB1 LEU A 445      30.139  51.351  17.856  1.00  0.00           H
ATOM   6503  HB2 LEU A 445      28.804  50.963  17.000  1.00  0.00           H
ATOM   6504  CG  LEU A 445      29.907  52.524  16.129  1.00  0.00           C
ATOM   6505  HG  LEU A 445      30.857  52.778  15.946  1.00  0.00           H
ATOM   6506  CD1 LEU A 445      29.152  52.344  14.849  1.00  0.00           C
ATOM   6507 HD11 LEU A 445      29.218  53.179  14.303  1.00  0.00           H
ATOM   6508 HD12 LEU A 445      29.542  51.579  14.337  1.00  0.00           H
ATOM   6509 HD13 LEU A 445      28.192  52.154  15.052  1.00  0.00           H
ATOM   6510  CD2 LEU A 445      29.083  53.569  16.930  1.00  0.00           C
ATOM   6511 HD21 LEU A 445      29.124  54.453  16.465  1.00  0.00           H
ATOM   6512 HD22 LEU A 445      28.131  53.268  16.990  1.00  0.00           H
ATOM   6513 HD23 LEU A 445      29.463  53.659  17.850  1.00  0.00           H
ATOM   6514  C   LEU A 445      31.979  50.275  15.971  1.00  0.00           C
ATOM   6515  O   LEU A 445      32.860  50.091  16.784  1.00  0.00           O
ATOM   6516  N   PHE A 446      32.169  50.672  14.709  1.00  0.00           N
ATOM   6517  H   PHE A 446      31.412  50.658  14.056  1.00  0.00           H
ATOM   6518  CA  PHE A 446      33.487  51.124  14.296  1.00  0.00           C
ATOM   6519  HA  PHE A 446      33.865  51.496  15.143  1.00  0.00           H
ATOM   6520  CB  PHE A 446      34.540  50.044  13.990  1.00  0.00           C
ATOM   6521  HB1 PHE A 446      34.722  49.560  14.846  1.00  0.00           H
ATOM   6522  HB2 PHE A 446      35.369  50.518  13.693  1.00  0.00           H
ATOM   6523  CG  PHE A 446      34.279  48.978  12.958  1.00  0.00           C
ATOM   6524  CD1 PHE A 446      33.407  47.921  13.260  1.00  0.00           C
ATOM   6525  HD1 PHE A 446      32.894  47.928  14.118  1.00  0.00           H
ATOM   6526  CE1 PHE A 446      33.253  46.855  12.363  1.00  0.00           C
ATOM   6527  HE1 PHE A 446      32.582  46.138  12.552  1.00  0.00           H
ATOM   6528  CZ  PHE A 446      34.036  46.791  11.203  1.00  0.00           C
ATOM   6529  HZ  PHE A 446      33.964  46.006  10.588  1.00  0.00           H
ATOM   6530  CE2 PHE A 446      34.923  47.840  10.913  1.00  0.00           C
ATOM   6531  HE2 PHE A 446      35.475  47.805  10.080  1.00  0.00           H
```

ATOM	6532	CD2	PHE	A	446	35.043	48.937	11.779	1.00	0.00	C
ATOM	6533	HD2	PHE	A	446	35.668	49.686	11.559	1.00	0.00	H
ATOM	6534	C	PHE	A	446	33.357	52.267	13.304	1.00	0.00	C
ATOM	6535	O	PHE	A	446	32.365	52.348	12.581	1.00	0.00	O
ATOM	6536	N	GLN	A	447	34.274	53.230	13.368	1.00	0.00	N
ATOM	6537	H	GLN	A	447	35.091	53.085	13.926	1.00	0.00	H
ATOM	6538	CA	GLN	A	447	34.125	54.488	12.651	1.00	0.00	C
ATOM	6539	HA	GLN	A	447	33.434	54.288	11.956	1.00	0.00	H
ATOM	6540	CB	GLN	A	447	33.625	55.582	13.589	1.00	0.00	C
ATOM	6541	HB1	GLN	A	447	34.276	55.648	14.345	1.00	0.00	H
ATOM	6542	HB2	GLN	A	447	32.735	55.290	13.940	1.00	0.00	H
ATOM	6543	CG	GLN	A	447	33.463	56.996	12.956	1.00	0.00	C
ATOM	6544	HG1	GLN	A	447	32.686	57.448	13.394	1.00	0.00	H
ATOM	6545	HG2	GLN	A	447	33.276	56.883	11.980	1.00	0.00	H
ATOM	6546	CD	GLN	A	447	34.690	57.922	13.097	1.00	0.00	C
ATOM	6547	OE1	GLN	A	447	35.358	58.193	12.108	1.00	0.00	O
ATOM	6548	NE2	GLN	A	447	34.997	58.478	14.274	1.00	0.00	N
ATOM	6549	HE21	GLN	A	447	34.429	58.296	15.076	1.00	0.00	H
ATOM	6550	HE22	GLN	A	447	35.795	59.076	14.352	1.00	0.00	H
ATOM	6551	C	GLN	A	447	35.429	54.928	11.955	1.00	0.00	C
ATOM	6552	O	GLN	A	447	36.482	54.899	12.587	1.00	0.00	O
ATOM	6553	N	GLN	A	448	35.335	55.460	10.725	1.00	0.00	N
ATOM	6554	H	GLN	A	448	34.476	55.336	10.228	1.00	0.00	H
ATOM	6555	CA	GLN	A	448	36.413	56.215	10.057	1.00	0.00	C
ATOM	6556	HA	GLN	A	448	36.831	56.717	10.814	1.00	0.00	H
ATOM	6557	CB	GLN	A	448	37.456	55.275	9.416	1.00	0.00	C
ATOM	6558	HB1	GLN	A	448	37.852	54.714	10.143	1.00	0.00	H
ATOM	6559	HB2	GLN	A	448	38.170	55.843	9.007	1.00	0.00	H
ATOM	6560	CG	GLN	A	448	36.933	54.325	8.326	1.00	0.00	C
ATOM	6561	HG1	GLN	A	448	37.710	53.955	7.816	1.00	0.00	H
ATOM	6562	HG2	GLN	A	448	36.340	54.837	7.705	1.00	0.00	H
ATOM	6563	CD	GLN	A	448	36.138	53.164	8.910	1.00	0.00	C
ATOM	6564	OE1	GLN	A	448	36.671	52.281	9.562	1.00	0.00	O
ATOM	6565	NE2	GLN	A	448	34.832	53.140	8.757	1.00	0.00	N
ATOM	6566	HE21	GLN	A	448	34.373	53.878	8.262	1.00	0.00	H
ATOM	6567	HE22	GLN	A	448	34.298	52.384	9.135	1.00	0.00	H
ATOM	6568	C	GLN	A	448	35.895	57.245	9.028	1.00	0.00	C
ATOM	6569	O	GLN	A	448	34.819	57.082	8.448	1.00	0.00	O
ATOM	6570	N	SER	A	449	36.717	58.261	8.727	1.00	0.00	N
ATOM	6571	H	SER	A	449	37.466	58.457	9.360	1.00	0.00	H
ATOM	6572	CA	SER	A	449	36.594	59.112	7.524	1.00	0.00	C
ATOM	6573	HA	SER	A	449	35.688	59.532	7.577	1.00	0.00	H
ATOM	6574	CB	SER	A	449	37.700	60.182	7.511	1.00	0.00	C
ATOM	6575	HB1	SER	A	449	38.598	59.746	7.458	1.00	0.00	H
ATOM	6576	HB2	SER	A	449	37.648	60.739	8.340	1.00	0.00	H
ATOM	6577	OG	SER	A	449	37.635	61.085	6.425	1.00	0.00	O
ATOM	6578	HG	SER	A	449	38.385	61.743	6.493	1.00	0.00	H
ATOM	6579	C	SER	A	449	36.715	58.267	6.255	1.00	0.00	C
ATOM	6580	O	SER	A	449	37.508	57.326	6.211	1.00	0.00	O
ATOM	6581	N	CYS	A	450	36.029	58.664	5.182	1.00	0.00	N
ATOM	6582	H	CYS	A	450	35.317	59.359	5.282	1.00	0.00	H
ATOM	6583	CA	CYS	A	450	36.304	58.098	3.862	1.00	0.00	C
ATOM	6584	HA	CYS	A	450	36.539	57.136	4.002	1.00	0.00	H
ATOM	6585	CB	CYS	A	450	35.044	58.165	2.993	1.00	0.00	C

ATOM	6586	HB1	CYS	A	450	34.997	59.114	2.680	1.00	0.00	H
ATOM	6587	HB2	CYS	A	450	34.288	57.984	3.622	1.00	0.00	H
ATOM	6588	SG	CYS	A	450	35.081	56.992	1.614	1.00	0.00	S
ATOM	6589	C	CYS	A	450	37.511	58.808	3.216	1.00	0.00	C
ATOM	6590	O	CYS	A	450	37.594	60.031	3.317	1.00	0.00	O
ATOM	6591	N	PRO	A	451	38.425	58.104	2.518	1.00	0.00	N
ATOM	6592	CD	PRO	A	451	38.590	56.654	2.505	1.00	0.00	C
ATOM	6593	HD1	PRO	A	451	37.732	56.220	2.231	1.00	0.00	H
ATOM	6594	HD2	PRO	A	451	38.847	56.337	3.418	1.00	0.00	H
ATOM	6595	CG	PRO	A	451	39.698	56.381	1.491	1.00	0.00	C
ATOM	6596	HG1	PRO	A	451	39.324	56.266	0.571	1.00	0.00	H
ATOM	6597	HG2	PRO	A	451	40.223	55.567	1.741	1.00	0.00	H
ATOM	6598	CB	PRO	A	451	40.558	57.636	1.580	1.00	0.00	C
ATOM	6599	HB1	PRO	A	451	41.098	57.765	0.749	1.00	0.00	H
ATOM	6600	HB2	PRO	A	451	41.168	57.602	2.372	1.00	0.00	H
ATOM	6601	CA	PRO	A	451	39.500	58.732	1.735	1.00	0.00	C
ATOM	6602	HA	PRO	A	451	39.825	59.535	2.235	1.00	0.00	H
ATOM	6603	C	PRO	A	451	39.034	59.265	0.365	1.00	0.00	C
ATOM	6604	O	PRO	A	451	39.839	59.787	-0.403	1.00	0.00	O
ATOM	6605	N	THR	A	452	37.750	59.106	0.030	1.00	0.00	N
ATOM	6606	H	THR	A	452	37.156	58.656	0.697	1.00	0.00	H
ATOM	6607	CA	THR	A	452	37.136	59.537	-1.236	1.00	0.00	C
ATOM	6608	HA	THR	A	452	37.795	60.177	-1.631	1.00	0.00	H
ATOM	6609	CB	THR	A	452	36.900	58.352	-2.191	1.00	0.00	C
ATOM	6610	HB	THR	A	452	36.410	58.682	-2.998	1.00	0.00	H
ATOM	6611	CG2	THR	A	452	38.199	57.665	-2.605	1.00	0.00	C
ATOM	6612	HG21	THR	A	452	37.993	56.906	-3.222	1.00	0.00	H
ATOM	6613	HG22	THR	A	452	38.790	58.323	-3.072	1.00	0.00	H
ATOM	6614	HG23	THR	A	452	38.665	57.316	-1.792	1.00	0.00	H
ATOM	6615	OG1	THR	A	452	36.059	57.386	-1.589	1.00	0.00	O
ATOM	6616	HG1	THR	A	452	35.918	56.626	-2.223	1.00	0.00	H
ATOM	6617	C	THR	A	452	35.798	60.220	-0.971	1.00	0.00	C
ATOM	6618	O	THR	A	452	35.043	59.746	-0.121	1.00	0.00	O
ATOM	6619	N	GLY	A	453	35.443	61.219	-1.785	1.00	0.00	N
ATOM	6620	H	GLY	A	453	36.107	61.527	-2.467	1.00	0.00	H
ATOM	6621	CA	GLY	A	453	34.139	61.893	-1.741	1.00	0.00	C
ATOM	6622	HA1	GLY	A	453	33.442	61.195	-1.908	1.00	0.00	H
ATOM	6623	HA2	GLY	A	453	34.128	62.560	-2.486	1.00	0.00	H
ATOM	6624	C	GLY	A	453	33.826	62.610	-0.420	1.00	0.00	C
ATOM	6625	O	GLY	A	453	34.649	62.642	0.493	1.00	0.00	O
ATOM	6626	N	LEU	A	454	32.627	63.202	-0.323	1.00	0.00	N
ATOM	6627	H	LEU	A	454	32.003	63.145	-1.102	1.00	0.00	H
ATOM	6628	CA	LEU	A	454	32.187	63.931	0.877	1.00	0.00	C
ATOM	6629	HA	LEU	A	454	32.974	63.765	1.471	1.00	0.00	H
ATOM	6630	CB	LEU	A	454	31.947	65.420	0.565	1.00	0.00	C
ATOM	6631	HB1	LEU	A	454	31.310	65.781	1.246	1.00	0.00	H
ATOM	6632	HB2	LEU	A	454	31.535	65.484	-0.344	1.00	0.00	H
ATOM	6633	CG	LEU	A	454	33.193	66.308	0.576	1.00	0.00	C
ATOM	6634	HG	LEU	A	454	33.873	65.869	-0.011	1.00	0.00	H
ATOM	6635	CD1	LEU	A	454	32.756	67.700	0.090	1.00	0.00	C
ATOM	6636	HD11	LEU	A	454	33.546	68.314	0.083	1.00	0.00	H
ATOM	6637	HD12	LEU	A	454	32.381	67.628	-0.834	1.00	0.00	H
ATOM	6638	HD13	LEU	A	454	32.057	68.064	0.706	1.00	0.00	H
ATOM	6639	CD2	LEU	A	454	33.846	66.470	1.963	1.00	0.00	C

```
ATOM   6640 HD21 LEU A 454      34.649  67.061   1.886  1.00  0.00           H
ATOM   6641 HD22 LEU A 454      33.189  66.881   2.595  1.00  0.00           H
ATOM   6642 HD23 LEU A 454      34.124  65.573   2.308  1.00  0.00           H
ATOM   6643  C   LEU A 454      30.975  63.384   1.626  1.00  0.00           C
ATOM   6644  O   LEU A 454      30.661  63.835   2.746  1.00  0.00           O
ATOM   6645  N   VAL A 455      30.270  62.470   0.957  1.00  0.00           N
ATOM   6646  H   VAL A 455      30.629  62.119   0.092  1.00  0.00           H
ATOM   6647  CA  VAL A 455      29.000  61.969   1.444  1.00  0.00           C
ATOM   6648  HA  VAL A 455      29.077  62.059   2.437  1.00  0.00           H
ATOM   6649  CB  VAL A 455      27.835  62.851   0.948  1.00  0.00           C
ATOM   6650  HB  VAL A 455      28.062  63.771   1.267  1.00  0.00           H
ATOM   6651  CG1 VAL A 455      27.699  62.920  -0.573  1.00  0.00           C
ATOM   6652 HG11 VAL A 455      26.926  63.507  -0.812  1.00  0.00           H
ATOM   6653 HG12 VAL A 455      28.538  63.297  -0.966  1.00  0.00           H
ATOM   6654 HG13 VAL A 455      27.544  62.001  -0.936  1.00  0.00           H
ATOM   6655  CG2 VAL A 455      26.499  62.420   1.542  1.00  0.00           C
ATOM   6656 HG21 VAL A 455      25.773  63.015   1.196  1.00  0.00           H
ATOM   6657 HG22 VAL A 455      26.308  61.474   1.280  1.00  0.00           H
ATOM   6658 HG23 VAL A 455      26.540  62.488   2.539  1.00  0.00           H
ATOM   6659  C   VAL A 455      28.805  60.480   1.263  1.00  0.00           C
ATOM   6660  O   VAL A 455      28.993  59.950   0.166  1.00  0.00           O
ATOM   6661  N   PHE A 456      28.420  59.811   2.356  1.00  0.00           N
ATOM   6662  H   PHE A 456      28.314  60.298   3.223  1.00  0.00           H
ATOM   6663  CA  PHE A 456      28.152  58.370   2.294  1.00  0.00           C
ATOM   6664  HA  PHE A 456      28.552  58.011   1.451  1.00  0.00           H
ATOM   6665  CB  PHE A 456      28.775  57.709   3.543  1.00  0.00           C
ATOM   6666  HB1 PHE A 456      28.205  57.932   4.334  1.00  0.00           H
ATOM   6667  HB2 PHE A 456      29.691  58.088   3.675  1.00  0.00           H
ATOM   6668  CG  PHE A 456      28.911  56.200   3.493  1.00  0.00           C
ATOM   6669  CD1 PHE A 456      30.075  55.607   2.980  1.00  0.00           C
ATOM   6670  HD1 PHE A 456      30.787  56.179   2.573  1.00  0.00           H
ATOM   6671  CE1 PHE A 456      30.245  54.213   3.036  1.00  0.00           C
ATOM   6672  HE1 PHE A 456      31.077  53.795   2.671  1.00  0.00           H
ATOM   6673  CZ  PHE A 456      29.245  53.408   3.606  1.00  0.00           C
ATOM   6674  HZ  PHE A 456      29.369  52.417   3.654  1.00  0.00           H
ATOM   6675  CE2 PHE A 456      28.071  53.994   4.109  1.00  0.00           C
ATOM   6676  HE2 PHE A 456      27.352  53.423   4.506  1.00  0.00           H
ATOM   6677  CD2 PHE A 456      27.915  55.390   4.050  1.00  0.00           C
ATOM   6678  HD2 PHE A 456      27.082  55.809   4.411  1.00  0.00           H
ATOM   6679  C   PHE A 456      26.657  58.132   2.255  1.00  0.00           C
ATOM   6680  O   PHE A 456      25.915  58.530   3.143  1.00  0.00           O
ATOM   6681  N   SER A 457      26.263  57.437   1.174  1.00  0.00           N
ATOM   6682  H   SER A 457      26.929  57.166   0.479  1.00  0.00           H
ATOM   6683  CA  SER A 457      24.848  57.078   1.020  1.00  0.00           C
ATOM   6684  HA  SER A 457      24.317  57.708   1.587  1.00  0.00           H
ATOM   6685  CB  SER A 457      24.475  57.189  -0.467  1.00  0.00           C
ATOM   6686  HB1 SER A 457      25.022  56.557  -1.016  1.00  0.00           H
ATOM   6687  HB2 SER A 457      24.615  58.123  -0.797  1.00  0.00           H
ATOM   6688  OG  SER A 457      23.093  56.857  -0.663  1.00  0.00           O
ATOM   6689  HG  SER A 457      22.866  56.934  -1.634  1.00  0.00           H
ATOM   6690  C   SER A 457      24.601  55.666   1.505  1.00  0.00           C
ATOM   6691  O   SER A 457      25.390  54.740   1.298  1.00  0.00           O
ATOM   6692  N   ASN A 458      23.359  55.491   1.988  1.00  0.00           N
ATOM   6693  H   ASN A 458      22.715  56.255   2.009  1.00  0.00           H
```

ATOM	6694	CA	ASN	A	458	22.954	54.167	2.486	1.00	0.00	C
ATOM	6695	HA	ASN	A	458	23.805	53.792	2.853	1.00	0.00	H
ATOM	6696	CB	ASN	A	458	21.852	54.321	3.548	1.00	0.00	C
ATOM	6697	HB1	ASN	A	458	20.974	54.468	3.093	1.00	0.00	H
ATOM	6698	HB2	ASN	A	458	22.062	55.108	4.129	1.00	0.00	H
ATOM	6699	CG	ASN	A	458	21.740	53.093	4.424	1.00	0.00	C
ATOM	6700	OD1	ASN	A	458	22.492	52.130	4.354	1.00	0.00	O
ATOM	6701	ND2	ASN	A	458	20.740	53.206	5.307	1.00	0.00	N
ATOM	6702	HD21	ASN	A	458	20.179	54.034	5.324	1.00	0.00	H
ATOM	6703	HD22	ASN	A	458	20.554	52.463	5.950	1.00	0.00	H
ATOM	6704	C	ASN	A	458	22.482	53.204	1.418	1.00	0.00	C
ATOM	6705	O	ASN	A	458	22.435	51.997	1.610	1.00	0.00	O
ATOM	6706	N	SER	A	459	22.170	53.808	0.259	1.00	0.00	N
ATOM	6707	H	SER	A	459	22.233	54.803	0.177	1.00	0.00	H
ATOM	6708	CA	SER	A	459	21.736	52.998	-0.887	1.00	0.00	C
ATOM	6709	HA	SER	A	459	21.041	52.386	-0.509	1.00	0.00	H
ATOM	6710	CB	SER	A	459	21.116	53.876	-1.991	1.00	0.00	C
ATOM	6711	HB1	SER	A	459	21.787	54.532	-2.338	1.00	0.00	H
ATOM	6712	HB2	SER	A	459	20.318	54.368	-1.643	1.00	0.00	H
ATOM	6713	OG	SER	A	459	20.682	53.073	-3.097	1.00	0.00	O
ATOM	6714	HG	SER	A	459	20.284	53.662	-3.800	1.00	0.00	H
ATOM	6715	C	SER	A	459	22.849	52.126	-1.433	1.00	0.00	C
ATOM	6716	O	SER	A	459	22.724	50.916	-1.553	1.00	0.00	O
ATOM	6717	N	CYS	A	460	23.958	52.801	-1.758	1.00	0.00	N
ATOM	6718	H	CYS	A	460	23.985	53.790	-1.614	1.00	0.00	H
ATOM	6719	CA	CYS	A	460	25.130	52.132	-2.317	1.00	0.00	C
ATOM	6720	HA	CYS	A	460	24.767	51.331	-2.792	1.00	0.00	H
ATOM	6721	CB	CYS	A	460	25.826	53.085	-3.289	1.00	0.00	C
ATOM	6722	HB1	CYS	A	460	25.134	53.277	-3.985	1.00	0.00	H
ATOM	6723	HB2	CYS	A	460	26.559	52.536	-3.691	1.00	0.00	H
ATOM	6724	SG	CYS	A	460	26.457	54.601	-2.527	1.00	0.00	S
ATOM	6725	C	CYS	A	460	26.106	51.612	-1.250	1.00	0.00	C
ATOM	6726	O	CYS	A	460	27.068	50.934	-1.603	1.00	0.00	O
ATOM	6727	N	LYS	A	461	25.911	51.976	0.031	1.00	0.00	N
ATOM	6728	H	LYS	A	461	25.040	52.410	0.262	1.00	0.00	H
ATOM	6729	CA	LYS	A	461	26.889	51.782	1.122	1.00	0.00	C
ATOM	6730	HA	LYS	A	461	26.541	52.303	1.901	1.00	0.00	H
ATOM	6731	CB	LYS	A	461	26.966	50.296	1.522	1.00	0.00	C
ATOM	6732	HB1	LYS	A	461	27.547	50.224	2.333	1.00	0.00	H
ATOM	6733	HB2	LYS	A	461	27.385	49.793	0.766	1.00	0.00	H
ATOM	6734	CG	LYS	A	461	25.624	49.626	1.844	1.00	0.00	C
ATOM	6735	HG1	LYS	A	461	25.764	48.636	1.867	1.00	0.00	H
ATOM	6736	HG2	LYS	A	461	24.976	49.854	1.118	1.00	0.00	H
ATOM	6737	CD	LYS	A	461	25.019	50.060	3.185	1.00	0.00	C
ATOM	6738	HD1	LYS	A	461	24.774	51.029	3.158	1.00	0.00	H
ATOM	6739	HD2	LYS	A	461	25.670	49.901	3.928	1.00	0.00	H
ATOM	6740	CE	LYS	A	461	23.767	49.203	3.385	1.00	0.00	C
ATOM	6741	HE1	LYS	A	461	23.961	48.270	3.081	1.00	0.00	H
ATOM	6742	HE2	LYS	A	461	23.022	49.584	2.837	1.00	0.00	H
ATOM	6743	NZ	LYS	A	461	23.292	49.124	4.785	1.00	0.00	N
ATOM	6744	HZ1	LYS	A	461	22.475	48.549	4.828	1.00	0.00	H
ATOM	6745	HZ2	LYS	A	461	24.009	48.728	5.358	1.00	0.00	H
ATOM	6746	HZ3	LYS	A	461	23.071	50.042	5.114	1.00	0.00	H
ATOM	6747	C	LYS	A	461	28.264	52.364	0.751	1.00	0.00	C

ATOM	6748	O	LYS	A	461	29.291	51.707	0.919	1.00	0.00	O
ATOM	6749	N	CYS	A	462	28.267	53.546	0.132	1.00	0.00	N
ATOM	6750	H	CYS	A	462	27.440	54.106	0.173	1.00	0.00	H
ATOM	6751	CA	CYS	A	462	29.419	54.062	-0.602	1.00	0.00	C
ATOM	6752	HA	CYS	A	462	30.213	53.653	-0.152	1.00	0.00	H
ATOM	6753	CB	CYS	A	462	29.312	53.617	-2.070	1.00	0.00	C
ATOM	6754	HB1	CYS	A	462	28.906	52.705	-2.005	1.00	0.00	H
ATOM	6755	HB2	CYS	A	462	30.267	53.537	-2.356	1.00	0.00	H
ATOM	6756	SG	CYS	A	462	28.371	54.679	-3.203	1.00	0.00	S
ATOM	6757	C	CYS	A	462	29.596	55.581	-0.481	1.00	0.00	C
ATOM	6758	O	CYS	A	462	28.634	56.325	-0.276	1.00	0.00	O
ATOM	6759	N	CYS	A	463	30.837	56.034	-0.668	1.00	0.00	N
ATOM	6760	H	CYS	A	463	31.558	55.355	-0.809	1.00	0.00	H
ATOM	6761	CA	CYS	A	463	31.228	57.442	-0.683	1.00	0.00	C
ATOM	6762	HA	CYS	A	463	30.613	57.897	-0.039	1.00	0.00	H
ATOM	6763	CB	CYS	A	463	32.683	57.591	-0.220	1.00	0.00	C
ATOM	6764	HB1	CYS	A	463	32.790	58.572	-0.058	1.00	0.00	H
ATOM	6765	HB2	CYS	A	463	33.223	57.324	-1.018	1.00	0.00	H
ATOM	6766	SG	CYS	A	463	33.122	56.619	1.242	1.00	0.00	S
ATOM	6767	C	CYS	A	463	31.041	58.052	-2.080	1.00	0.00	C
ATOM	6768	O	CYS	A	463	31.390	57.432	-3.089	1.00	0.00	O
ATOM	6769	N	THR	A	464	30.524	59.280	-2.114	1.00	0.00	N
ATOM	6770	H	THR	A	464	30.405	59.739	-1.234	1.00	0.00	H
ATOM	6771	CA	THR	A	464	30.110	60.032	-3.311	1.00	0.00	C
ATOM	6772	HA	THR	A	464	30.707	59.719	-4.050	1.00	0.00	H
ATOM	6773	CB	THR	A	464	28.631	59.730	-3.636	1.00	0.00	C
ATOM	6774	HB	THR	A	464	28.343	60.364	-4.353	1.00	0.00	H
ATOM	6775	CG2	THR	A	464	28.386	58.286	-4.061	1.00	0.00	C
ATOM	6776	HG21	THR	A	464	27.414	58.156	-4.256	1.00	0.00	H
ATOM	6777	HG22	THR	A	464	28.921	58.085	-4.882	1.00	0.00	H
ATOM	6778	HG23	THR	A	464	28.663	57.670	-3.324	1.00	0.00	H
ATOM	6779	OG1	THR	A	464	27.805	59.975	-2.521	1.00	0.00	O
ATOM	6780	HG1	THR	A	464	26.855	59.772	-2.759	1.00	0.00	H
ATOM	6781	C	THR	A	464	30.301	61.545	-3.101	1.00	0.00	C
ATOM	6782	O	THR	A	464	30.819	61.974	-2.068	1.00	0.00	O
ATOM	6783	N	TRP	A	465	29.892	62.378	-4.059	1.00	0.00	N
ATOM	6784	H	TRP	A	465	29.749	61.988	-4.969	1.00	0.00	H
ATOM	6785	CA	TRP	A	465	29.633	63.814	-3.901	1.00	0.00	C
ATOM	6786	HA	TRP	A	465	29.140	63.916	-3.037	1.00	0.00	H
ATOM	6787	CB	TRP	A	465	30.941	64.622	-3.763	1.00	0.00	C
ATOM	6788	HB1	TRP	A	465	31.382	64.353	-2.907	1.00	0.00	H
ATOM	6789	HB2	TRP	A	465	30.702	65.592	-3.724	1.00	0.00	H
ATOM	6790	CG	TRP	A	465	31.976	64.482	-4.835	1.00	0.00	C
ATOM	6791	CD1	TRP	A	465	32.872	63.476	-4.941	1.00	0.00	C
ATOM	6792	HD1	TRP	A	465	32.942	62.702	-4.311	1.00	0.00	H
ATOM	6793	NE1	TRP	A	465	33.677	63.673	-6.047	1.00	0.00	N
ATOM	6794	HE1	TRP	A	465	34.423	63.065	-6.319	1.00	0.00	H
ATOM	6795	CE2	TRP	A	465	33.318	64.814	-6.725	1.00	0.00	C
ATOM	6796	CZ2	TRP	A	465	33.774	65.395	-7.916	1.00	0.00	C
ATOM	6797	HZ2	TRP	A	465	34.566	65.017	-8.395	1.00	0.00	H
ATOM	6798	CH2	TRP	A	465	33.102	66.515	-8.426	1.00	0.00	C
ATOM	6799	HH2	TRP	A	465	33.386	66.915	-9.298	1.00	0.00	H
ATOM	6800	CZ3	TRP	A	465	32.028	67.076	-7.710	1.00	0.00	C
ATOM	6801	HZ3	TRP	A	465	31.556	67.880	-8.071	1.00	0.00	H

```
ATOM   6802  CE3 TRP A 465      31.614  66.511  -6.488  1.00  0.00           C
ATOM   6803  HE3 TRP A 465      30.876  66.943  -5.970  1.00  0.00           H
ATOM   6804  CD2 TRP A 465      32.225  65.345  -5.984  1.00  0.00           C
ATOM   6805  C   TRP A 465      28.731  64.305  -5.047  1.00  0.00           C
ATOM   6806  O   TRP A 465      28.358  63.529  -5.928  1.00  0.00           O
ATOM   6807  N   ASN A 466      28.283  65.567  -4.890  1.00  0.00           N
ATOM   6808  H   ASN A 466      28.582  66.110  -4.105  1.00  0.00           H
ATOM   6809  CA  ASN A 466      27.352  66.147  -5.876  1.00  0.00           C
ATOM   6810  HA  ASN A 466      27.640  65.803  -6.770  1.00  0.00           H
ATOM   6811  CB  ASN A 466      25.901  65.700  -5.600  1.00  0.00           C
ATOM   6812  HB1 ASN A 466      25.580  66.144  -4.763  1.00  0.00           H
ATOM   6813  HB2 ASN A 466      25.887  64.708  -5.477  1.00  0.00           H
ATOM   6814  CG  ASN A 466      24.935  66.048  -6.716  1.00  0.00           C
ATOM   6815  OD1 ASN A 466      23.717  66.026  -6.607  1.00  0.00           O
ATOM   6816  ND2 ASN A 466      25.579  66.410  -7.835  1.00  0.00           N
ATOM   6817 HD21 ASN A 466      26.579  66.424  -7.854  1.00  0.00           H
ATOM   6818 HD22 ASN A 466      25.060  66.666  -8.650  1.00  0.00           H
ATOM   6819  C   ASN A 466      27.466  67.659  -5.877  1.00  0.00           C
ATOM   6820  OC1 ASN A 466      28.249  68.074  -6.341  1.00  0.00           O
ATOM   6821  OC2 ASN A 466      26.651  68.399  -5.340  1.00  0.00           O
TER
ENDMDL
```
