# Supplementary material for: Elucidation of the Catalytic Apparatus and Mechanism of Human Chitotriosidase‑1
Source: ACS Catal. 2025 Sep 19;15(19):16748–61. doi: 10.1021/acscatal.5c00507 (PMC12502790; doi:10.1021/acscatal.5c00507)
Supplement: Supplementary file 2 [file cs5c00507_si_002.pdf]

```
TITLE     Atomic coordinates of hCHIT1 catalytic domain with (GlcNAc)3 - model
CRYST1   68.708   50.012   46.311  90.00  90.00  90.00 P 1           1
MODEL        1
ATOM      1  N   ALA A  22      38.487  29.297  40.775  1.00  0.00           N
ATOM      2  H1  ALA A  22      39.368  28.783  40.767  1.00  0.00           H
ATOM      3  H2  ALA A  22      38.680  30.271  41.001  1.00  0.00           H
ATOM      4  H3  ALA A  22      37.977  28.937  41.578  1.00  0.00           H
ATOM      5  CA  ALA A  22      37.717  29.118  39.517  1.00  0.00           C
ATOM      6  HA  ALA A  22      37.676  28.048  39.299  1.00  0.00           H
ATOM      7  CB  ALA A  22      36.273  29.585  39.728  1.00  0.00           C
ATOM      8  HB1 ALA A  22      35.703  29.443  38.808  1.00  0.00           H
ATOM      9  HB2 ALA A  22      35.803  29.001  40.517  1.00  0.00           H
ATOM     10  HB3 ALA A  22      36.246  30.638  40.005  1.00  0.00           H
ATOM     11  C   ALA A  22      38.337  29.807  38.274  1.00  0.00           C
ATOM     12  O   ALA A  22      38.749  30.966  38.344  1.00  0.00           O
ATOM     13  N   LYS A  23      38.346  29.113  37.131  1.00  0.00           N
ATOM     14  H   LYS A  23      38.069  28.140  37.169  1.00  0.00           H
ATOM     15  CA  LYS A  23      38.533  29.697  35.792  1.00  0.00           C
ATOM     16  HA  LYS A  23      39.155  30.590  35.861  1.00  0.00           H
ATOM     17  CB  LYS A  23      39.214  28.670  34.860  1.00  0.00           C
ATOM     18  HB1 LYS A  23      38.740  27.695  34.987  1.00  0.00           H
ATOM     19  HB2 LYS A  23      39.048  28.985  33.830  1.00  0.00           H
ATOM     20  CG  LYS A  23      40.732  28.534  35.041  1.00  0.00           C
ATOM     21  HG1 LYS A  23      41.190  29.498  34.818  1.00  0.00           H
ATOM     22  HG2 LYS A  23      40.952  28.251  36.070  1.00  0.00           H
ATOM     23  CD  LYS A  23      41.302  27.471  34.082  1.00  0.00           C
ATOM     24  HD1 LYS A  23      40.875  27.621  33.090  1.00  0.00           H
ATOM     25  HD2 LYS A  23      41.003  26.481  34.428  1.00  0.00           H
ATOM     26  CE  LYS A  23      42.830  27.508  33.925  1.00  0.00           C
ATOM     27  HE1 LYS A  23      43.115  26.767  33.175  1.00  0.00           H
ATOM     28  HE2 LYS A  23      43.130  28.488  33.547  1.00  0.00           H
ATOM     29  NZ  LYS A  23      43.551  27.220  35.185  1.00  0.00           N
ATOM     30  HZ1 LYS A  23      43.452  27.990  35.839  1.00  0.00           H
ATOM     31  HZ2 LYS A  23      43.209  26.365  35.611  1.00  0.00           H
ATOM     32  HZ3 LYS A  23      44.545  27.092  35.003  1.00  0.00           H
ATOM     33  C   LYS A  23      37.188  30.102  35.153  1.00  0.00           C
ATOM     34  O   LYS A  23      36.170  29.441  35.372  1.00  0.00           O
ATOM     35  N   LEU A  24      37.222  31.090  34.254  1.00  0.00           N
ATOM     36  H   LEU A  24      38.084  31.616  34.155  1.00  0.00           H
ATOM     37  CA  LEU A  24      36.235  31.245  33.179  1.00  0.00           C
ATOM     38  HA  LEU A  24      35.591  30.367  33.159  1.00  0.00           H
ATOM     39  CB  LEU A  24      35.333  32.465  33.450  1.00  0.00           C
ATOM     40  HB1 LEU A  24      34.796  32.286  34.383  1.00  0.00           H
ATOM     41  HB2 LEU A  24      35.956  33.348  33.590  1.00  0.00           H
ATOM     42  CG  LEU A  24      34.302  32.786  32.346  1.00  0.00           C
ATOM     43  HG  LEU A  24      34.838  33.160  31.475  1.00  0.00           H
ATOM     44  CD1 LEU A  24      33.463  31.573  31.915  1.00  0.00           C
ATOM     45 HD11 LEU A  24      32.716  31.887  31.186  1.00  0.00           H
ATOM     46 HD12 LEU A  24      34.091  30.818  31.445  1.00  0.00           H
ATOM     47 HD13 LEU A  24      32.961  31.142  32.781  1.00  0.00           H
ATOM     48  CD2 LEU A  24      33.354  33.890  32.837  1.00  0.00           C
ATOM     49 HD21 LEU A  24      32.678  34.186  32.034  1.00  0.00           H
ATOM     50 HD22 LEU A  24      32.769  33.533  33.685  1.00  0.00           H
ATOM     51 HD23 LEU A  24      33.928  34.761  33.147  1.00  0.00           H
```

ATOM	52	C	LEU	A	24	37.004	31.316	31.853	1.00	0.00	C
ATOM	53	O	LEU	A	24	37.762	32.263	31.640	1.00	0.00	O
ATOM	54	N	VAL	A	25	36.857	30.281	31.024	1.00	0.00	N
ATOM	55	H	VAL	A	25	36.219	29.540	31.317	1.00	0.00	H
ATOM	56	CA	VAL	A	25	37.619	30.064	29.781	1.00	0.00	C
ATOM	57	HA	VAL	A	25	38.472	30.741	29.774	1.00	0.00	H
ATOM	58	CB	VAL	A	25	38.168	28.618	29.692	1.00	0.00	C
ATOM	59	HB	VAL	A	25	37.333	27.925	29.600	1.00	0.00	H
ATOM	60	CG1	VAL	A	25	39.071	28.453	28.463	1.00	0.00	C
ATOM	61	HG11	VAL	A	25	39.440	27.429	28.412	1.00	0.00	H
ATOM	62	HG12	VAL	A	25	38.513	28.657	27.548	1.00	0.00	H
ATOM	63	HG13	VAL	A	25	39.916	29.140	28.529	1.00	0.00	H
ATOM	64	CG2	VAL	A	25	38.956	28.215	30.951	1.00	0.00	C
ATOM	65	HG21	VAL	A	25	39.393	27.224	30.816	1.00	0.00	H
ATOM	66	HG22	VAL	A	25	39.753	28.931	31.147	1.00	0.00	H
ATOM	67	HG23	VAL	A	25	38.287	28.172	31.810	1.00	0.00	H
ATOM	68	C	VAL	A	25	36.730	30.361	28.567	1.00	0.00	C
ATOM	69	O	VAL	A	25	35.775	29.634	28.298	1.00	0.00	O
ATOM	70	N	CYS	A	26	37.031	31.424	27.824	1.00	0.00	N
ATOM	71	H	CYS	A	26	37.841	31.984	28.089	1.00	0.00	H
ATOM	72	CA	CYS	A	26	36.169	31.925	26.746	1.00	0.00	C
ATOM	73	HA	CYS	A	26	35.238	31.359	26.703	1.00	0.00	H
ATOM	74	CB	CYS	A	26	35.813	33.376	27.071	1.00	0.00	C
ATOM	75	HB1	CYS	A	26	36.735	33.952	27.166	1.00	0.00	H
ATOM	76	HB2	CYS	A	26	35.228	33.797	26.255	1.00	0.00	H
ATOM	77	SG	CYS	A	26	34.861	33.539	28.601	1.00	0.00	S
ATOM	78	C	CYS	A	26	36.835	31.821	25.374	1.00	0.00	C
ATOM	79	O	CYS	A	26	37.918	32.367	25.172	1.00	0.00	O
ATOM	80	N	TYR	A	27	36.182	31.159	24.416	1.00	0.00	N
ATOM	81	H	TYR	A	27	35.298	30.716	24.632	1.00	0.00	H
ATOM	82	CA	TYR	A	27	36.648	31.110	23.026	1.00	0.00	C
ATOM	83	HA	TYR	A	27	37.738	31.135	23.021	1.00	0.00	H
ATOM	84	CB	TYR	A	27	36.233	29.787	22.366	1.00	0.00	C
ATOM	85	HB1	TYR	A	27	35.163	29.631	22.518	1.00	0.00	H
ATOM	86	HB2	TYR	A	27	36.390	29.879	21.290	1.00	0.00	H
ATOM	87	CG	TYR	A	27	36.997	28.559	22.841	1.00	0.00	C
ATOM	88	CD1	TYR	A	27	38.194	28.174	22.200	1.00	0.00	C
ATOM	89	HD1	TYR	A	27	38.594	28.781	21.400	1.00	0.00	H
ATOM	90	CE1	TYR	A	27	38.862	26.991	22.582	1.00	0.00	C
ATOM	91	HE1	TYR	A	27	39.769	26.685	22.079	1.00	0.00	H
ATOM	92	CZ	TYR	A	27	38.339	26.187	23.619	1.00	0.00	C
ATOM	93	OH	TYR	A	27	38.957	25.037	24.005	1.00	0.00	O
ATOM	94	HH	TYR	A	27	39.791	24.858	23.528	1.00	0.00	H
ATOM	95	CE2	TYR	A	27	37.144	26.577	24.259	1.00	0.00	C
ATOM	96	HE2	TYR	A	27	36.740	25.947	25.032	1.00	0.00	H
ATOM	97	CD2	TYR	A	27	36.478	27.756	23.876	1.00	0.00	C
ATOM	98	HD2	TYR	A	27	35.549	28.026	24.357	1.00	0.00	H
ATOM	99	C	TYR	A	27	36.151	32.321	22.209	1.00	0.00	C
ATOM	100	O	TYR	A	27	34.953	32.625	22.185	1.00	0.00	O
ATOM	101	N	PHE	A	28	37.088	32.979	21.523	1.00	0.00	N
ATOM	102	H	PHE	A	28	38.041	32.642	21.622	1.00	0.00	H
ATOM	103	CA	PHE	A	28	36.879	33.970	20.464	1.00	0.00	C
ATOM	104	HA	PHE	A	28	35.866	34.364	20.518	1.00	0.00	H
ATOM	105	CB	PHE	A	28	37.887	35.122	20.659	1.00	0.00	C

ATOM	106	HB1	PHE	A	28	37.532	35.778	21.452	1.00	0.00	H
ATOM	107	HB2	PHE	A	28	38.840	34.699	20.981	1.00	0.00	H
ATOM	108	CG	PHE	A	28	38.132	35.944	19.405	1.00	0.00	C
ATOM	109	CD1	PHE	A	28	37.121	36.789	18.913	1.00	0.00	C
ATOM	110	HD1	PHE	A	28	36.200	36.904	19.464	1.00	0.00	H
ATOM	111	CE1	PHE	A	28	37.278	37.426	17.671	1.00	0.00	C
ATOM	112	HE1	PHE	A	28	36.474	38.023	17.266	1.00	0.00	H
ATOM	113	CZ	PHE	A	28	38.457	37.247	16.927	1.00	0.00	C
ATOM	114	HZ	PHE	A	28	38.569	37.715	15.959	1.00	0.00	H
ATOM	115	CE2	PHE	A	28	39.477	36.422	17.426	1.00	0.00	C
ATOM	116	HE2	PHE	A	28	40.370	36.259	16.841	1.00	0.00	H
ATOM	117	CD2	PHE	A	28	39.314	35.767	18.659	1.00	0.00	C
ATOM	118	HD2	PHE	A	28	40.085	35.097	19.010	1.00	0.00	H
ATOM	119	C	PHE	A	28	37.088	33.302	19.094	1.00	0.00	C
ATOM	120	O	PHE	A	28	37.997	32.480	18.967	1.00	0.00	O
ATOM	121	N	THR	A	29	36.307	33.672	18.067	1.00	0.00	N
ATOM	122	H	THR	A	29	35.599	34.383	18.226	1.00	0.00	H
ATOM	123	CA	THR	A	29	36.432	33.123	16.703	1.00	0.00	C
ATOM	124	HA	THR	A	29	37.217	32.368	16.694	1.00	0.00	H
ATOM	125	CB	THR	A	29	35.147	32.416	16.255	1.00	0.00	C
ATOM	126	HB	THR	A	29	35.332	31.991	15.271	1.00	0.00	H
ATOM	127	CG2	THR	A	29	34.724	31.288	17.199	1.00	0.00	C
ATOM	128	HG21	THR	A	29	33.832	30.804	16.803	1.00	0.00	H
ATOM	129	HG22	THR	A	29	35.521	30.549	17.270	1.00	0.00	H
ATOM	130	HG23	THR	A	29	34.502	31.676	18.193	1.00	0.00	H
ATOM	131	OG1	THR	A	29	34.080	33.321	16.149	1.00	0.00	O
ATOM	132	HG1	THR	A	29	33.545	33.271	16.963	1.00	0.00	H
ATOM	133	C	THR	A	29	36.847	34.197	15.699	1.00	0.00	C
ATOM	134	O	THR	A	29	36.228	35.257	15.600	1.00	0.00	O
ATOM	135	N	ASN	A	30	37.887	33.922	14.906	1.00	0.00	N
ATOM	136	H	ASN	A	30	38.375	33.040	15.035	1.00	0.00	H
ATOM	137	CA	ASN	A	30	38.421	34.870	13.914	1.00	0.00	C
ATOM	138	HA	ASN	A	30	38.343	35.861	14.362	1.00	0.00	H
ATOM	139	CB	ASN	A	30	39.931	34.636	13.699	1.00	0.00	C
ATOM	140	HB1	ASN	A	30	40.341	35.512	13.195	1.00	0.00	H
ATOM	141	HB2	ASN	A	30	40.420	34.568	14.671	1.00	0.00	H
ATOM	142	CG	ASN	A	30	40.315	33.405	12.881	1.00	0.00	C
ATOM	143	OD1	ASN	A	30	39.523	32.512	12.606	1.00	0.00	O
ATOM	144	ND2	ASN	A	30	41.559	33.321	12.451	1.00	0.00	N
ATOM	145	HD21	ASN	A	30	42.218	34.085	12.598	1.00	0.00	H
ATOM	146	HD22	ASN	A	30	41.840	32.537	11.886	1.00	0.00	H
ATOM	147	C	ASN	A	30	37.607	34.959	12.599	1.00	0.00	C
ATOM	148	O	ASN	A	30	38.012	35.660	11.670	1.00	0.00	O
ATOM	149	N	TRP	A	31	36.450	34.285	12.511	1.00	0.00	N
ATOM	150	H	TRP	A	31	36.200	33.690	13.288	1.00	0.00	H
ATOM	151	CA	TRP	A	31	35.533	34.331	11.360	1.00	0.00	C
ATOM	152	HA	TRP	A	31	36.010	34.877	10.545	1.00	0.00	H
ATOM	153	CB	TRP	A	31	35.297	32.896	10.867	1.00	0.00	C
ATOM	154	HB1	TRP	A	31	36.257	32.482	10.555	1.00	0.00	H
ATOM	155	HB2	TRP	A	31	34.661	32.925	9.981	1.00	0.00	H
ATOM	156	CG	TRP	A	31	34.683	31.953	11.857	1.00	0.00	C
ATOM	157	CD1	TRP	A	31	33.372	31.886	12.178	1.00	0.00	C
ATOM	158	HD1	TRP	A	31	32.593	32.507	11.756	1.00	0.00	H
ATOM	159	NE1	TRP	A	31	33.183	30.936	13.161	1.00	0.00	N

ATOM	160	HE1	TRP	A	31	32.319	30.847	13.692	1.00	0.00	H
ATOM	161	CE2	TRP	A	31	34.359	30.306	13.494	1.00	0.00	C
ATOM	162	CZ2	TRP	A	31	34.680	29.309	14.426	1.00	0.00	C
ATOM	163	HZ2	TRP	A	31	33.903	28.854	15.023	1.00	0.00	H
ATOM	164	CH2	TRP	A	31	36.027	28.963	14.616	1.00	0.00	C
ATOM	165	HH2	TRP	A	31	36.293	28.238	15.369	1.00	0.00	H
ATOM	166	CZ3	TRP	A	31	37.027	29.598	13.858	1.00	0.00	C
ATOM	167	HZ3	TRP	A	31	38.068	29.365	14.028	1.00	0.00	H
ATOM	168	CE3	TRP	A	31	36.687	30.570	12.899	1.00	0.00	C
ATOM	169	HE3	TRP	A	31	37.465	31.056	12.331	1.00	0.00	H
ATOM	170	CD2	TRP	A	31	35.346	30.959	12.698	1.00	0.00	C
ATOM	171	C	TRP	A	31	34.193	35.048	11.631	1.00	0.00	C
ATOM	172	O	TRP	A	31	33.459	35.329	10.680	1.00	0.00	O
ATOM	173	N	ALA	A	32	33.851	35.376	12.885	1.00	0.00	N
ATOM	174	H	ALA	A	32	34.469	35.113	13.644	1.00	0.00	H
ATOM	175	CA	ALA	A	32	32.604	36.080	13.225	1.00	0.00	C
ATOM	176	HA	ALA	A	32	31.768	35.553	12.760	1.00	0.00	H
ATOM	177	CB	ALA	A	32	32.416	36.005	14.741	1.00	0.00	C
ATOM	178	HB1	ALA	A	32	31.506	36.535	15.023	1.00	0.00	H
ATOM	179	HB2	ALA	A	32	32.316	34.962	15.044	1.00	0.00	H
ATOM	180	HB3	ALA	A	32	33.272	36.446	15.256	1.00	0.00	H
ATOM	181	C	ALA	A	32	32.548	37.534	12.705	1.00	0.00	C
ATOM	182	O	ALA	A	32	31.461	38.062	12.468	1.00	0.00	O
ATOM	183	N	GLN	A	33	33.708	38.146	12.447	1.00	0.00	N
ATOM	184	H	GLN	A	33	34.546	37.706	12.816	1.00	0.00	H
ATOM	185	CA	GLN	A	33	33.872	39.446	11.782	1.00	0.00	C
ATOM	186	HA	GLN	A	33	33.395	40.209	12.396	1.00	0.00	H
ATOM	187	CB	GLN	A	33	35.374	39.770	11.691	1.00	0.00	C
ATOM	188	HB1	GLN	A	33	35.476	40.751	11.226	1.00	0.00	H
ATOM	189	HB2	GLN	A	33	35.771	39.846	12.703	1.00	0.00	H
ATOM	190	CG	GLN	A	33	36.225	38.746	10.901	1.00	0.00	C
ATOM	191	HG1	GLN	A	33	36.183	37.775	11.393	1.00	0.00	H
ATOM	192	HG2	GLN	A	33	35.824	38.630	9.895	1.00	0.00	H
ATOM	193	CD	GLN	A	33	37.690	39.174	10.774	1.00	0.00	C
ATOM	194	OE1	GLN	A	33	38.006	40.358	10.730	1.00	0.00	O
ATOM	195	NE2	GLN	A	33	38.641	38.266	10.725	1.00	0.00	N
ATOM	196	HE21	GLN	A	33	38.415	37.285	10.861	1.00	0.00	H
ATOM	197	HE22	GLN	A	33	39.606	38.552	10.614	1.00	0.00	H
ATOM	198	C	GLN	A	33	33.225	39.553	10.389	1.00	0.00	C
ATOM	199	O	GLN	A	33	33.003	40.666	9.903	1.00	0.00	O
ATOM	200	N	TYR	A	34	32.927	38.419	9.747	1.00	0.00	N
ATOM	201	H	TYR	A	34	33.164	37.544	10.198	1.00	0.00	H
ATOM	202	CA	TYR	A	34	32.301	38.334	8.424	1.00	0.00	C
ATOM	203	HA	TYR	A	34	32.546	39.235	7.860	1.00	0.00	H
ATOM	204	CB	TYR	A	34	32.900	37.125	7.682	1.00	0.00	C
ATOM	205	HB1	TYR	A	34	32.588	36.222	8.204	1.00	0.00	H
ATOM	206	HB2	TYR	A	34	32.471	37.077	6.682	1.00	0.00	H
ATOM	207	CG	TYR	A	34	34.415	37.110	7.531	1.00	0.00	C
ATOM	208	CD1	TYR	A	34	35.092	38.228	7.002	1.00	0.00	C
ATOM	209	HD1	TYR	A	34	34.540	39.112	6.715	1.00	0.00	H
ATOM	210	CE1	TYR	A	34	36.487	38.191	6.814	1.00	0.00	C
ATOM	211	HE1	TYR	A	34	36.998	39.042	6.389	1.00	0.00	H
ATOM	212	CZ	TYR	A	34	37.216	37.026	7.142	1.00	0.00	C
ATOM	213	OH	TYR	A	34	38.555	36.979	6.907	1.00	0.00	O

ATOM	214	HH	TYR	A	34	38.942	36.096	7.041	1.00	0.00	H
ATOM	215	CE2	TYR	A	34	36.543	35.909	7.683	1.00	0.00	C
ATOM	216	HE2	TYR	A	34	37.095	35.013	7.929	1.00	0.00	H
ATOM	217	CD2	TYR	A	34	35.148	35.956	7.874	1.00	0.00	C
ATOM	218	HD2	TYR	A	34	34.638	35.086	8.261	1.00	0.00	H
ATOM	219	C	TYR	A	34	30.758	38.245	8.466	1.00	0.00	C
ATOM	220	O	TYR	A	34	30.122	38.209	7.406	1.00	0.00	O
ATOM	221	N	ARG	A	35	30.135	38.207	9.653	1.00	0.00	N
ATOM	222	H	ARG	A	35	30.706	38.252	10.490	1.00	0.00	H
ATOM	223	CA	ARG	A	35	28.669	38.271	9.814	1.00	0.00	C
ATOM	224	HA	ARG	A	35	28.208	37.666	9.036	1.00	0.00	H
ATOM	225	CB	ARG	A	35	28.246	37.689	11.170	1.00	0.00	C
ATOM	226	HB1	ARG	A	35	28.794	38.182	11.973	1.00	0.00	H
ATOM	227	HB2	ARG	A	35	27.178	37.853	11.323	1.00	0.00	H
ATOM	228	CG	ARG	A	35	28.520	36.181	11.191	1.00	0.00	C
ATOM	229	HG1	ARG	A	35	27.933	35.693	10.412	1.00	0.00	H
ATOM	230	HG2	ARG	A	35	29.579	36.001	10.999	1.00	0.00	H
ATOM	231	CD	ARG	A	35	28.170	35.566	12.541	1.00	0.00	C
ATOM	232	HD1	ARG	A	35	28.712	36.114	13.312	1.00	0.00	H
ATOM	233	HD2	ARG	A	35	27.097	35.645	12.724	1.00	0.00	H
ATOM	234	NE	ARG	A	35	28.582	34.158	12.546	1.00	0.00	N
ATOM	235	HE	ARG	A	35	28.696	33.713	11.642	1.00	0.00	H
ATOM	236	CZ	ARG	A	35	29.047	33.474	13.577	1.00	0.00	C
ATOM	237	NH1	ARG	A	35	29.037	33.945	14.804	1.00	0.00	N
ATOM	238	HH11	ARG	A	35	28.628	34.850	15.007	1.00	0.00	H
ATOM	239	HH12	ARG	A	35	29.519	33.461	15.562	1.00	0.00	H
ATOM	240	NH2	ARG	A	35	29.563	32.295	13.341	1.00	0.00	N
ATOM	241	HH21	ARG	A	35	29.657	31.974	12.384	1.00	0.00	H
ATOM	242	HH22	ARG	A	35	30.006	31.786	14.100	1.00	0.00	H
ATOM	243	C	ARG	A	35	28.162	39.707	9.646	1.00	0.00	C
ATOM	244	O	ARG	A	35	28.942	40.642	9.771	1.00	0.00	O
ATOM	245	N	GLN	A	36	26.881	39.903	9.342	1.00	0.00	N
ATOM	246	H	GLN	A	36	26.267	39.101	9.285	1.00	0.00	H
ATOM	247	CA	GLN	A	36	26.322	41.243	9.107	1.00	0.00	C
ATOM	248	HA	GLN	A	36	27.125	41.931	8.834	1.00	0.00	H
ATOM	249	CB	GLN	A	36	25.329	41.211	7.929	1.00	0.00	C
ATOM	250	HB1	GLN	A	36	24.431	40.679	8.241	1.00	0.00	H
ATOM	251	HB2	GLN	A	36	25.040	42.236	7.690	1.00	0.00	H
ATOM	252	CG	GLN	A	36	25.863	40.546	6.646	1.00	0.00	C
ATOM	253	HG1	GLN	A	36	25.922	39.471	6.812	1.00	0.00	H
ATOM	254	HG2	GLN	A	36	25.147	40.712	5.840	1.00	0.00	H
ATOM	255	CD	GLN	A	36	27.234	41.057	6.196	1.00	0.00	C
ATOM	256	OE1	GLN	A	36	27.526	42.247	6.193	1.00	0.00	O
ATOM	257	NE2	GLN	A	36	28.148	40.184	5.837	1.00	0.00	N
ATOM	258	HE21	GLN	A	36	27.927	39.203	5.771	1.00	0.00	H
ATOM	259	HE22	GLN	A	36	29.053	40.545	5.572	1.00	0.00	H
ATOM	260	C	GLN	A	36	25.662	41.831	10.365	1.00	0.00	C
ATOM	261	O	GLN	A	36	25.337	41.110	11.311	1.00	0.00	O
ATOM	262	N	GLY	A	37	25.444	43.151	10.357	1.00	0.00	N
ATOM	263	H	GLY	A	37	25.790	43.687	9.567	1.00	0.00	H
ATOM	264	CA	GLY	A	37	24.563	43.856	11.293	1.00	0.00	C
ATOM	265	HA1	GLY	A	37	24.454	44.897	10.988	1.00	0.00	H
ATOM	266	HA2	GLY	A	37	23.581	43.384	11.255	1.00	0.00	H
ATOM	267	C	GLY	A	37	25.033	43.832	12.746	1.00	0.00	C

ATOM	268	O	GLY	A	37	26.236	43.812	13.037	1.00	0.00	O
ATOM	269	N	GLU	A	38	24.070	43.835	13.665	1.00	0.00	N
ATOM	270	H	GLU	A	38	23.113	43.968	13.342	1.00	0.00	H
ATOM	271	CA	GLU	A	38	24.286	43.792	15.113	1.00	0.00	C
ATOM	272	HA	GLU	A	38	24.888	44.654	15.404	1.00	0.00	H
ATOM	273	CB	GLU	A	38	22.939	43.870	15.856	1.00	0.00	C
ATOM	274	HB1	GLU	A	38	22.330	43.006	15.589	1.00	0.00	H
ATOM	275	HB2	GLU	A	38	23.167	43.806	16.920	1.00	0.00	H
ATOM	276	CG	GLU	A	38	22.117	45.155	15.649	1.00	0.00	C
ATOM	277	HG1	GLU	A	38	21.563	45.353	16.566	1.00	0.00	H
ATOM	278	HG2	GLU	A	38	22.790	45.999	15.487	1.00	0.00	H
ATOM	279	CD	GLU	A	38	21.114	45.068	14.495	1.00	0.00	C
ATOM	280	OE1	GLU	A	38	21.552	44.928	13.332	1.00	0.00	O
ATOM	281	OE2	GLU	A	38	19.890	45.223	14.724	1.00	0.00	O
ATOM	282	C	GLU	A	38	25.031	42.520	15.559	1.00	0.00	C
ATOM	283	O	GLU	A	38	25.748	42.547	16.558	1.00	0.00	O
ATOM	284	N	ALA	A	39	24.914	41.424	14.800	1.00	0.00	N
ATOM	285	H	ALA	A	39	24.325	41.473	13.981	1.00	0.00	H
ATOM	286	CA	ALA	A	39	25.547	40.139	15.091	1.00	0.00	C
ATOM	287	HA	ALA	A	39	25.492	39.952	16.166	1.00	0.00	H
ATOM	288	CB	ALA	A	39	24.736	39.070	14.363	1.00	0.00	C
ATOM	289	HB1	ALA	A	39	25.155	38.089	14.576	1.00	0.00	H
ATOM	290	HB2	ALA	A	39	23.698	39.095	14.699	1.00	0.00	H
ATOM	291	HB3	ALA	A	39	24.784	39.245	13.289	1.00	0.00	H
ATOM	292	C	ALA	A	39	27.032	40.054	14.690	1.00	0.00	C
ATOM	293	O	ALA	A	39	27.717	39.118	15.112	1.00	0.00	O
ATOM	294	N	ARG	A	40	27.549	41.005	13.895	1.00	0.00	N
ATOM	295	H	ARG	A	40	26.927	41.721	13.543	1.00	0.00	H
ATOM	296	CA	ARG	A	40	28.981	41.089	13.577	1.00	0.00	C
ATOM	297	HA	ARG	A	40	29.288	40.148	13.120	1.00	0.00	H
ATOM	298	CB	ARG	A	40	29.255	42.226	12.579	1.00	0.00	C
ATOM	299	HB1	ARG	A	40	28.546	42.159	11.755	1.00	0.00	H
ATOM	300	HB2	ARG	A	40	29.103	43.185	13.072	1.00	0.00	H
ATOM	301	CG	ARG	A	40	30.694	42.164	12.032	1.00	0.00	C
ATOM	302	HG1	ARG	A	40	31.398	42.084	12.860	1.00	0.00	H
ATOM	303	HG2	ARG	A	40	30.808	41.272	11.417	1.00	0.00	H
ATOM	304	CD	ARG	A	40	31.088	43.404	11.217	1.00	0.00	C
ATOM	305	HD1	ARG	A	40	31.112	44.264	11.888	1.00	0.00	H
ATOM	306	HD2	ARG	A	40	32.096	43.265	10.824	1.00	0.00	H
ATOM	307	NE	ARG	A	40	30.152	43.697	10.120	1.00	0.00	N
ATOM	308	HE	ARG	A	40	29.390	44.327	10.348	1.00	0.00	H
ATOM	309	CZ	ARG	A	40	30.173	43.199	8.891	1.00	0.00	C
ATOM	310	NH1	ARG	A	40	31.057	42.307	8.505	1.00	0.00	N
ATOM	311	HH11	ARG	A	40	31.673	41.874	9.180	1.00	0.00	H
ATOM	312	HH12	ARG	A	40	30.995	41.930	7.570	1.00	0.00	H
ATOM	313	NH2	ARG	A	40	29.267	43.580	8.020	1.00	0.00	N
ATOM	314	HH21	ARG	A	40	28.523	44.217	8.284	1.00	0.00	H
ATOM	315	HH22	ARG	A	40	29.169	43.113	7.129	1.00	0.00	H
ATOM	316	C	ARG	A	40	29.788	41.312	14.859	1.00	0.00	C
ATOM	317	O	ARG	A	40	29.621	42.346	15.514	1.00	0.00	O
ATOM	318	N	PHE	A	41	30.684	40.381	15.184	1.00	0.00	N
ATOM	319	H	PHE	A	41	30.761	39.571	14.586	1.00	0.00	H
ATOM	320	CA	PHE	A	41	31.616	40.490	16.307	1.00	0.00	C
ATOM	321	HA	PHE	A	41	31.188	41.137	17.076	1.00	0.00	H

ATOM	322	CB	PHE	A	41	31.813	39.094	16.911	1.00	0.00	C
ATOM	323	HB1	PHE	A	41	30.841	38.604	16.998	1.00	0.00	H
ATOM	324	HB2	PHE	A	41	32.426	38.495	16.237	1.00	0.00	H
ATOM	325	CG	PHE	A	41	32.455	39.110	18.281	1.00	0.00	C
ATOM	326	CD1	PHE	A	41	33.855	39.155	18.417	1.00	0.00	C
ATOM	327	HD1	PHE	A	41	34.483	39.177	17.538	1.00	0.00	H
ATOM	328	CE1	PHE	A	41	34.436	39.191	19.697	1.00	0.00	C
ATOM	329	HE1	PHE	A	41	35.509	39.239	19.801	1.00	0.00	H
ATOM	330	CZ	PHE	A	41	33.619	39.165	20.841	1.00	0.00	C
ATOM	331	HZ	PHE	A	41	34.056	39.189	21.826	1.00	0.00	H
ATOM	332	CE2	PHE	A	41	32.222	39.113	20.704	1.00	0.00	C
ATOM	333	HE2	PHE	A	41	31.586	39.086	21.578	1.00	0.00	H
ATOM	334	CD2	PHE	A	41	31.643	39.094	19.427	1.00	0.00	C
ATOM	335	HD2	PHE	A	41	30.569	39.064	19.331	1.00	0.00	H
ATOM	336	C	PHE	A	41	32.950	41.091	15.835	1.00	0.00	C
ATOM	337	O	PHE	A	41	33.416	40.752	14.748	1.00	0.00	O
ATOM	338	N	LEU	A	42	33.569	41.964	16.636	1.00	0.00	N
ATOM	339	H	LEU	A	42	33.152	42.180	17.535	1.00	0.00	H
ATOM	340	CA	LEU	A	42	34.820	42.668	16.309	1.00	0.00	C
ATOM	341	HA	LEU	A	42	35.409	42.050	15.631	1.00	0.00	H
ATOM	342	CB	LEU	A	42	34.466	44.013	15.632	1.00	0.00	C
ATOM	343	HB1	LEU	A	42	33.749	44.530	16.270	1.00	0.00	H
ATOM	344	HB2	LEU	A	42	35.353	44.634	15.589	1.00	0.00	H
ATOM	345	CG	LEU	A	42	33.890	43.941	14.202	1.00	0.00	C
ATOM	346	HG	LEU	A	42	32.979	43.344	14.204	1.00	0.00	H
ATOM	347	CD1	LEU	A	42	33.522	45.356	13.736	1.00	0.00	C
ATOM	348	HD11	LEU	A	42	33.086	45.309	12.738	1.00	0.00	H
ATOM	349	HD12	LEU	A	42	32.788	45.794	14.411	1.00	0.00	H
ATOM	350	HD13	LEU	A	42	34.409	45.990	13.709	1.00	0.00	H
ATOM	351	CD2	LEU	A	42	34.886	43.340	13.197	1.00	0.00	C
ATOM	352	HD21	LEU	A	42	34.449	43.341	12.198	1.00	0.00	H
ATOM	353	HD22	LEU	A	42	35.806	43.926	13.184	1.00	0.00	H
ATOM	354	HD23	LEU	A	42	35.121	42.311	13.462	1.00	0.00	H
ATOM	355	C	LEU	A	42	35.672	42.897	17.581	1.00	0.00	C
ATOM	356	O	LEU	A	42	35.143	42.739	18.684	1.00	0.00	O
ATOM	357	N	PRO	A	43	36.963	43.290	17.463	1.00	0.00	N
ATOM	358	CD	PRO	A	43	37.736	43.361	16.228	1.00	0.00	C
ATOM	359	HD1	PRO	A	43	37.450	44.246	15.667	1.00	0.00	H
ATOM	360	HD2	PRO	A	43	37.600	42.469	15.620	1.00	0.00	H
ATOM	361	CG	PRO	A	43	39.200	43.488	16.644	1.00	0.00	C
ATOM	362	HG1	PRO	A	43	39.778	44.067	15.924	1.00	0.00	H
ATOM	363	HG2	PRO	A	43	39.633	42.497	16.782	1.00	0.00	H
ATOM	364	CB	PRO	A	43	39.108	44.192	17.991	1.00	0.00	C
ATOM	365	HB1	PRO	A	43	38.980	45.264	17.831	1.00	0.00	H
ATOM	366	HB2	PRO	A	43	39.981	43.998	18.612	1.00	0.00	H
ATOM	367	CA	PRO	A	43	37.842	43.585	18.599	1.00	0.00	C
ATOM	368	HA	PRO	A	43	38.100	42.646	19.088	1.00	0.00	H
ATOM	369	C	PRO	A	43	37.239	44.527	19.641	1.00	0.00	C
ATOM	370	O	PRO	A	43	37.417	44.301	20.835	1.00	0.00	O
ATOM	371	N	LYS	A	44	36.482	45.543	19.205	1.00	0.00	N
ATOM	372	H	LYS	A	44	36.429	45.724	18.210	1.00	0.00	H
ATOM	373	CA	LYS	A	44	35.808	46.482	20.106	1.00	0.00	C
ATOM	374	HA	LYS	A	44	36.578	46.983	20.695	1.00	0.00	H
ATOM	375	CB	LYS	A	44	35.079	47.553	19.269	1.00	0.00	C

ATOM	376	HB1	LYS	A	44	34.749	48.349	19.940	1.00	0.00	H
ATOM	377	HB2	LYS	A	44	35.776	47.986	18.550	1.00	0.00	H
ATOM	378	CG	LYS	A	44	33.854	46.994	18.527	1.00	0.00	C
ATOM	379	HG1	LYS	A	44	34.171	46.205	17.845	1.00	0.00	H
ATOM	380	HG2	LYS	A	44	33.165	46.577	19.258	1.00	0.00	H
ATOM	381	CD	LYS	A	44	33.105	48.071	17.736	1.00	0.00	C
ATOM	382	HD1	LYS	A	44	32.779	48.863	18.414	1.00	0.00	H
ATOM	383	HD2	LYS	A	44	33.781	48.505	16.998	1.00	0.00	H
ATOM	384	CE	LYS	A	44	31.885	47.486	17.010	1.00	0.00	C
ATOM	385	HE1	LYS	A	44	31.403	48.284	16.443	1.00	0.00	H
ATOM	386	HE2	LYS	A	44	32.214	46.721	16.301	1.00	0.00	H
ATOM	387	NZ	LYS	A	44	30.900	46.889	17.943	1.00	0.00	N
ATOM	388	HZ1	LYS	A	44	30.053	46.636	17.446	1.00	0.00	H
ATOM	389	HZ2	LYS	A	44	31.263	46.037	18.366	1.00	0.00	H
ATOM	390	HZ3	LYS	A	44	30.643	47.535	18.684	1.00	0.00	H
ATOM	391	C	LYS	A	44	34.858	45.819	21.125	1.00	0.00	C
ATOM	392	O	LYS	A	44	34.672	46.371	22.206	1.00	0.00	O
ATOM	393	N	ASP	A	45	34.263	44.662	20.809	1.00	0.00	N
ATOM	394	H	ASP	A	45	34.518	44.220	19.931	1.00	0.00	H
ATOM	395	CA	ASP	A	45	33.254	43.988	21.641	1.00	0.00	C
ATOM	396	HA	ASP	A	45	32.686	44.747	22.184	1.00	0.00	H
ATOM	397	CB	ASP	A	45	32.261	43.227	20.742	1.00	0.00	C
ATOM	398	HB1	ASP	A	45	32.800	42.477	20.160	1.00	0.00	H
ATOM	399	HB2	ASP	A	45	31.544	42.702	21.374	1.00	0.00	H
ATOM	400	CG	ASP	A	45	31.486	44.152	19.798	1.00	0.00	C
ATOM	401	OD1	ASP	A	45	30.485	44.780	20.219	1.00	0.00	O
ATOM	402	OD2	ASP	A	45	31.886	44.277	18.618	1.00	0.00	O
ATOM	403	C	ASP	A	45	33.872	43.051	22.700	1.00	0.00	C
ATOM	404	O	ASP	A	45	33.145	42.479	23.515	1.00	0.00	O
ATOM	405	N	LEU	A	46	35.202	42.894	22.721	1.00	0.00	N
ATOM	406	H	LEU	A	46	35.752	43.399	22.034	1.00	0.00	H
ATOM	407	CA	LEU	A	46	35.918	42.156	23.765	1.00	0.00	C
ATOM	408	HA	LEU	A	46	35.328	41.283	24.042	1.00	0.00	H
ATOM	409	CB	LEU	A	46	37.286	41.685	23.232	1.00	0.00	C
ATOM	410	HB1	LEU	A	46	37.816	42.552	22.833	1.00	0.00	H
ATOM	411	HB2	LEU	A	46	37.873	41.295	24.064	1.00	0.00	H
ATOM	412	CG	LEU	A	46	37.189	40.595	22.142	1.00	0.00	C
ATOM	413	HG	LEU	A	46	36.428	40.882	21.415	1.00	0.00	H
ATOM	414	CD1	LEU	A	46	38.518	40.444	21.390	1.00	0.00	C
ATOM	415	HD11	LEU	A	46	38.399	39.733	20.572	1.00	0.00	H
ATOM	416	HD12	LEU	A	46	38.825	41.406	20.978	1.00	0.00	H
ATOM	417	HD13	LEU	A	46	39.293	40.079	22.064	1.00	0.00	H
ATOM	418	CD2	LEU	A	46	36.814	39.232	22.748	1.00	0.00	C
ATOM	419	HD21	LEU	A	46	36.731	38.486	21.958	1.00	0.00	H
ATOM	420	HD22	LEU	A	46	37.579	38.913	23.458	1.00	0.00	H
ATOM	421	HD23	LEU	A	46	35.859	39.296	23.267	1.00	0.00	H
ATOM	422	C	LEU	A	46	36.069	43.014	25.030	1.00	0.00	C
ATOM	423	O	LEU	A	46	36.418	44.193	24.967	1.00	0.00	O
ATOM	424	N	ASP	A	47	35.813	42.423	26.193	1.00	0.00	N
ATOM	425	H	ASP	A	47	35.514	41.454	26.172	1.00	0.00	H
ATOM	426	CA	ASP	A	47	35.988	43.032	27.517	1.00	0.00	C
ATOM	427	HA	ASP	A	47	36.116	44.109	27.418	1.00	0.00	H
ATOM	428	CB	ASP	A	47	34.703	42.786	28.327	1.00	0.00	C
ATOM	429	HB1	ASP	A	47	33.868	43.234	27.785	1.00	0.00	H

ATOM	430	HB2	ASP	A	47	34.523	41.713	28.372	1.00	0.00	H
ATOM	431	CG	ASP	A	47	34.697	43.351	29.756	1.00	0.00	C
ATOM	432	OD1	ASP	A	47	35.737	43.814	30.272	1.00	0.00	O
ATOM	433	OD2	ASP	A	47	33.604	43.363	30.366	1.00	0.00	O
ATOM	434	C	ASP	A	47	37.217	42.407	28.207	1.00	0.00	C
ATOM	435	O	ASP	A	47	37.253	41.180	28.338	1.00	0.00	O
ATOM	436	N	PRO	A	48	38.210	43.197	28.666	1.00	0.00	N
ATOM	437	CD	PRO	A	48	38.308	44.646	28.531	1.00	0.00	C
ATOM	438	HD1	PRO	A	48	37.407	45.148	28.881	1.00	0.00	H
ATOM	439	HD2	PRO	A	48	38.497	44.900	27.487	1.00	0.00	H
ATOM	440	CG	PRO	A	48	39.498	45.068	29.389	1.00	0.00	C
ATOM	441	HG1	PRO	A	48	39.166	45.250	30.410	1.00	0.00	H
ATOM	442	HG2	PRO	A	48	39.999	45.948	28.984	1.00	0.00	H
ATOM	443	CB	PRO	A	48	40.397	43.836	29.361	1.00	0.00	C
ATOM	444	HB1	PRO	A	48	41.041	43.795	30.237	1.00	0.00	H
ATOM	445	HB2	PRO	A	48	41.002	43.851	28.454	1.00	0.00	H
ATOM	446	CA	PRO	A	48	39.412	42.662	29.297	1.00	0.00	C
ATOM	447	HA	PRO	A	48	39.836	41.893	28.651	1.00	0.00	H
ATOM	448	C	PRO	A	48	39.168	42.017	30.674	1.00	0.00	C
ATOM	449	O	PRO	A	48	40.068	41.345	31.166	1.00	0.00	O
ATOM	450	N	SER	A	49	37.989	42.165	31.295	1.00	0.00	N
ATOM	451	H	SER	A	49	37.290	42.793	30.895	1.00	0.00	H
ATOM	452	CA	SER	A	49	37.671	41.542	32.598	1.00	0.00	C
ATOM	453	HA	SER	A	49	38.390	40.744	32.790	1.00	0.00	H
ATOM	454	CB	SER	A	49	37.837	42.567	33.728	1.00	0.00	C
ATOM	455	HB1	SER	A	49	37.714	42.067	34.687	1.00	0.00	H
ATOM	456	HB2	SER	A	49	38.844	42.986	33.685	1.00	0.00	H
ATOM	457	OG	SER	A	49	36.889	43.612	33.629	1.00	0.00	O
ATOM	458	HG	SER	A	49	35.996	43.222	33.638	1.00	0.00	H
ATOM	459	C	SER	A	49	36.292	40.851	32.648	1.00	0.00	C
ATOM	460	O	SER	A	49	35.603	40.862	33.675	1.00	0.00	O
ATOM	461	N	LEU	A	50	35.858	40.250	31.533	1.00	0.00	N
ATOM	462	H	LEU	A	50	36.439	40.300	30.705	1.00	0.00	H
ATOM	463	CA	LEU	A	50	34.710	39.335	31.531	1.00	0.00	C
ATOM	464	HA	LEU	A	50	34.001	39.657	32.290	1.00	0.00	H
ATOM	465	CB	LEU	A	50	34.009	39.411	30.164	1.00	0.00	C
ATOM	466	HB1	LEU	A	50	33.708	40.445	30.001	1.00	0.00	H
ATOM	467	HB2	LEU	A	50	34.720	39.130	29.385	1.00	0.00	H
ATOM	468	CG	LEU	A	50	32.752	38.527	30.034	1.00	0.00	C
ATOM	469	HG	LEU	A	50	33.027	37.488	30.215	1.00	0.00	H
ATOM	470	CD1	LEU	A	50	31.647	38.925	31.028	1.00	0.00	C
ATOM	471	HD11	LEU	A	50	30.749	38.339	30.836	1.00	0.00	H
ATOM	472	HD12	LEU	A	50	31.969	38.728	32.050	1.00	0.00	H
ATOM	473	HD13	LEU	A	50	31.409	39.984	30.921	1.00	0.00	H
ATOM	474	CD2	LEU	A	50	32.219	38.638	28.600	1.00	0.00	C
ATOM	475	HD21	LEU	A	50	31.301	38.060	28.494	1.00	0.00	H
ATOM	476	HD22	LEU	A	50	32.010	39.680	28.360	1.00	0.00	H
ATOM	477	HD23	LEU	A	50	32.956	38.243	27.901	1.00	0.00	H
ATOM	478	C	LEU	A	50	35.149	37.912	31.905	1.00	0.00	C
ATOM	479	O	LEU	A	50	34.559	37.297	32.791	1.00	0.00	O
ATOM	480	N	CYS	A	51	36.198	37.399	31.264	1.00	0.00	N
ATOM	481	H	CYS	A	51	36.645	37.968	30.551	1.00	0.00	H
ATOM	482	CA	CYS	A	51	36.724	36.041	31.445	1.00	0.00	C
ATOM	483	HA	CYS	A	51	36.038	35.463	32.065	1.00	0.00	H

ATOM	484	CB	CYS	A	51	36.779	35.375	30.063	1.00	0.00	C
ATOM	485	HB1	CYS	A	51	37.540	35.870	29.457	1.00	0.00	H
ATOM	486	HB2	CYS	A	51	37.067	34.330	30.175	1.00	0.00	H
ATOM	487	SG	CYS	A	51	35.197	35.453	29.168	1.00	0.00	S
ATOM	488	C	CYS	A	51	38.094	36.045	32.149	1.00	0.00	C
ATOM	489	O	CYS	A	51	38.732	37.094	32.288	1.00	0.00	O
ATOM	490	N	THR	A	52	38.579	34.873	32.587	1.00	0.00	N
ATOM	491	H	THR	A	52	38.022	34.037	32.447	1.00	0.00	H
ATOM	492	CA	THR	A	52	39.918	34.731	33.199	1.00	0.00	C
ATOM	493	HA	THR	A	52	40.266	35.723	33.487	1.00	0.00	H
ATOM	494	CB	THR	A	52	39.917	33.909	34.499	1.00	0.00	C
ATOM	495	HB	THR	A	52	40.780	34.232	35.082	1.00	0.00	H
ATOM	496	CG2	THR	A	52	38.678	34.137	35.356	1.00	0.00	C
ATOM	497	HG21	THR	A	52	38.775	33.608	36.301	1.00	0.00	H
ATOM	498	HG22	THR	A	52	38.568	35.201	35.553	1.00	0.00	H
ATOM	499	HG23	THR	A	52	37.788	33.780	34.846	1.00	0.00	H
ATOM	500	OG1	THR	A	52	40.051	32.521	34.288	1.00	0.00	O
ATOM	501	HG1	THR	A	52	40.621	32.220	35.023	1.00	0.00	H
ATOM	502	C	THR	A	52	40.945	34.219	32.204	1.00	0.00	C
ATOM	503	O	THR	A	52	42.117	34.578	32.308	1.00	0.00	O
ATOM	504	N	HID	A	53	40.528	33.393	31.248	1.00	0.00	N
ATOM	505	H	HID	A	53	39.549	33.119	31.235	1.00	0.00	H
ATOM	506	CA	HID	A	53	41.343	32.941	30.121	1.00	0.00	C
ATOM	507	HA	HID	A	53	42.270	33.513	30.076	1.00	0.00	H
ATOM	508	CB	HID	A	53	41.691	31.449	30.287	1.00	0.00	C
ATOM	509	HB1	HID	A	53	40.768	30.886	30.420	1.00	0.00	H
ATOM	510	HB2	HID	A	53	42.156	31.102	29.363	1.00	0.00	H
ATOM	511	CG	HID	A	53	42.630	31.125	31.433	1.00	0.00	C
ATOM	512	ND1	HID	A	53	42.530	31.573	32.730	1.00	0.00	N
ATOM	513	HD1	HID	A	53	41.818	32.199	33.081	1.00	0.00	H
ATOM	514	CE1	HID	A	53	43.572	31.099	33.428	1.00	0.00	C
ATOM	515	HE1	HID	A	53	43.771	31.319	34.470	1.00	0.00	H
ATOM	516	NE2	HID	A	53	44.337	30.315	32.650	1.00	0.00	N
ATOM	517	CD2	HID	A	53	43.742	30.327	31.382	1.00	0.00	C
ATOM	518	HD2	HID	A	53	44.104	29.802	30.510	1.00	0.00	H
ATOM	519	C	HID	A	53	40.566	33.186	28.813	1.00	0.00	C
ATOM	520	O	HID	A	53	39.366	32.911	28.739	1.00	0.00	O
ATOM	521	N	LEU	A	54	41.249	33.696	27.787	1.00	0.00	N
ATOM	522	H	LEU	A	54	42.240	33.880	27.911	1.00	0.00	H
ATOM	523	CA	LEU	A	54	40.693	33.993	26.465	1.00	0.00	C
ATOM	524	HA	LEU	A	54	39.630	33.750	26.443	1.00	0.00	H
ATOM	525	CB	LEU	A	54	40.874	35.505	26.219	1.00	0.00	C
ATOM	526	HB1	LEU	A	54	40.313	36.041	26.986	1.00	0.00	H
ATOM	527	HB2	LEU	A	54	41.930	35.750	26.349	1.00	0.00	H
ATOM	528	CG	LEU	A	54	40.428	36.032	24.841	1.00	0.00	C
ATOM	529	HG	LEU	A	54	41.011	35.535	24.067	1.00	0.00	H
ATOM	530	CD1	LEU	A	54	38.940	35.776	24.568	1.00	0.00	C
ATOM	531	HD11	LEU	A	54	38.646	36.263	23.637	1.00	0.00	H
ATOM	532	HD12	LEU	A	54	38.758	34.709	24.464	1.00	0.00	H
ATOM	533	HD13	LEU	A	54	38.337	36.171	25.386	1.00	0.00	H
ATOM	534	CD2	LEU	A	54	40.705	37.542	24.769	1.00	0.00	C
ATOM	535	HD21	LEU	A	54	40.403	37.931	23.796	1.00	0.00	H
ATOM	536	HD22	LEU	A	54	40.147	38.065	25.546	1.00	0.00	H
ATOM	537	HD23	LEU	A	54	41.771	37.731	24.902	1.00	0.00	H

ATOM	538	C	LEU	A	54	41.418	33.127	25.429	1.00	0.00	C
ATOM	539	O	LEU	A	54	42.647	33.146	25.374	1.00	0.00	O
ATOM	540	N	ILE	A	55	40.686	32.351	24.628	1.00	0.00	N
ATOM	541	H	ILE	A	55	39.672	32.388	24.707	1.00	0.00	H
ATOM	542	CA	ILE	A	55	41.272	31.417	23.652	1.00	0.00	C
ATOM	543	HA	ILE	A	55	42.351	31.489	23.725	1.00	0.00	H
ATOM	544	CB	ILE	A	55	40.916	29.942	23.967	1.00	0.00	C
ATOM	545	HB	ILE	A	55	39.859	29.780	23.756	1.00	0.00	H
ATOM	546	CG2	ILE	A	55	41.747	29.032	23.039	1.00	0.00	C
ATOM	547	HG21	ILE	A	55	41.471	27.988	23.182	1.00	0.00	H
ATOM	548	HG22	ILE	A	55	41.556	29.274	21.995	1.00	0.00	H
ATOM	549	HG23	ILE	A	55	42.811	29.148	23.246	1.00	0.00	H
ATOM	550	CG1	ILE	A	55	41.162	29.618	25.464	1.00	0.00	C
ATOM	551	HG11	ILE	A	55	42.094	30.079	25.783	1.00	0.00	H
ATOM	552	HG12	ILE	A	55	40.361	30.071	26.049	1.00	0.00	H
ATOM	553	CD	ILE	A	55	41.222	28.127	25.831	1.00	0.00	C
ATOM	554	HD1	ILE	A	55	41.352	28.024	26.907	1.00	0.00	H
ATOM	555	HD2	ILE	A	55	40.301	27.629	25.528	1.00	0.00	H
ATOM	556	HD3	ILE	A	55	42.076	27.647	25.353	1.00	0.00	H
ATOM	557	C	ILE	A	55	40.902	31.819	22.221	1.00	0.00	C
ATOM	558	O	ILE	A	55	39.732	32.003	21.900	1.00	0.00	O
ATOM	559	N	TYR	A	56	41.905	31.948	21.355	1.00	0.00	N
ATOM	560	H	TYR	A	56	42.845	31.777	21.698	1.00	0.00	H
ATOM	561	CA	TYR	A	56	41.763	32.348	19.953	1.00	0.00	C
ATOM	562	HA	TYR	A	56	40.904	33.013	19.855	1.00	0.00	H
ATOM	563	CB	TYR	A	56	43.035	33.133	19.581	1.00	0.00	C
ATOM	564	HB1	TYR	A	56	43.246	33.831	20.390	1.00	0.00	H
ATOM	565	HB2	TYR	A	56	43.882	32.450	19.547	1.00	0.00	H
ATOM	566	CG	TYR	A	56	42.996	33.926	18.286	1.00	0.00	C
ATOM	567	CD1	TYR	A	56	43.049	33.284	17.030	1.00	0.00	C
ATOM	568	HD1	TYR	A	56	43.092	32.206	16.970	1.00	0.00	H
ATOM	569	CE1	TYR	A	56	43.089	34.046	15.844	1.00	0.00	C
ATOM	570	HE1	TYR	A	56	43.147	33.555	14.884	1.00	0.00	H
ATOM	571	CZ	TYR	A	56	43.078	35.459	15.914	1.00	0.00	C
ATOM	572	OH	TYR	A	56	43.126	36.222	14.792	1.00	0.00	O
ATOM	573	HH	TYR	A	56	43.285	35.719	13.972	1.00	0.00	H
ATOM	574	CE2	TYR	A	56	43.031	36.098	17.170	1.00	0.00	C
ATOM	575	HE2	TYR	A	56	43.044	37.176	17.223	1.00	0.00	H
ATOM	576	CD2	TYR	A	56	42.993	35.333	18.346	1.00	0.00	C
ATOM	577	HD2	TYR	A	56	42.988	35.837	19.299	1.00	0.00	H
ATOM	578	C	TYR	A	56	41.546	31.125	19.039	1.00	0.00	C
ATOM	579	O	TYR	A	56	42.469	30.333	18.837	1.00	0.00	O
ATOM	580	N	ALA	A	57	40.348	30.971	18.466	1.00	0.00	N
ATOM	581	H	ALA	A	57	39.612	31.631	18.701	1.00	0.00	H
ATOM	582	CA	ALA	A	57	40.020	29.955	17.463	1.00	0.00	C
ATOM	583	HA	ALA	A	57	40.708	29.121	17.558	1.00	0.00	H
ATOM	584	CB	ALA	A	57	38.619	29.406	17.764	1.00	0.00	C
ATOM	585	HB1	ALA	A	57	38.368	28.626	17.047	1.00	0.00	H
ATOM	586	HB2	ALA	A	57	38.590	28.984	18.769	1.00	0.00	H
ATOM	587	HB3	ALA	A	57	37.877	30.201	17.687	1.00	0.00	H
ATOM	588	C	ALA	A	57	40.105	30.552	16.038	1.00	0.00	C
ATOM	589	O	ALA	A	57	39.402	31.516	15.740	1.00	0.00	O
ATOM	590	N	PHE	A	58	40.925	30.040	15.113	1.00	0.00	N
ATOM	591	H	PHE	A	58	40.899	30.509	14.219	1.00	0.00	H

ATOM	592	CA	PHE	A	58	41.949	28.994	15.252	1.00	0.00	C
ATOM	593	HA	PHE	A	58	42.210	28.838	16.296	1.00	0.00	H
ATOM	594	CB	PHE	A	58	41.446	27.667	14.647	1.00	0.00	C
ATOM	595	HB1	PHE	A	58	41.150	27.848	13.612	1.00	0.00	H
ATOM	596	HB2	PHE	A	58	42.278	26.961	14.612	1.00	0.00	H
ATOM	597	CG	PHE	A	58	40.315	26.968	15.377	1.00	0.00	C
ATOM	598	CD1	PHE	A	58	40.538	26.398	16.644	1.00	0.00	C
ATOM	599	HD1	PHE	A	58	41.511	26.471	17.101	1.00	0.00	H
ATOM	600	CE1	PHE	A	58	39.502	25.719	17.309	1.00	0.00	C
ATOM	601	HE1	PHE	A	58	39.672	25.280	18.282	1.00	0.00	H
ATOM	602	CZ	PHE	A	58	38.242	25.593	16.702	1.00	0.00	C
ATOM	603	HZ	PHE	A	58	37.450	25.059	17.210	1.00	0.00	H
ATOM	604	CE2	PHE	A	58	38.021	26.137	15.425	1.00	0.00	C
ATOM	605	HE2	PHE	A	58	37.060	26.012	14.947	1.00	0.00	H
ATOM	606	CD2	PHE	A	58	39.058	26.819	14.763	1.00	0.00	C
ATOM	607	HD2	PHE	A	58	38.895	27.214	13.772	1.00	0.00	H
ATOM	608	C	PHE	A	58	43.250	29.393	14.537	1.00	0.00	C
ATOM	609	O	PHE	A	58	43.256	30.240	13.640	1.00	0.00	O
ATOM	610	N	ALA	A	59	44.343	28.727	14.911	1.00	0.00	N
ATOM	611	H	ALA	A	59	44.249	28.106	15.709	1.00	0.00	H
ATOM	612	CA	ALA	A	59	45.572	28.601	14.134	1.00	0.00	C
ATOM	613	HA	ALA	A	59	45.672	29.455	13.469	1.00	0.00	H
ATOM	614	CB	ALA	A	59	46.761	28.599	15.095	1.00	0.00	C
ATOM	615	HB1	ALA	A	59	47.688	28.498	14.530	1.00	0.00	H
ATOM	616	HB2	ALA	A	59	46.783	29.543	15.630	1.00	0.00	H
ATOM	617	HB3	ALA	A	59	46.672	27.775	15.805	1.00	0.00	H
ATOM	618	C	ALA	A	59	45.563	27.333	13.269	1.00	0.00	C
ATOM	619	O	ALA	A	59	45.017	26.302	13.671	1.00	0.00	O
ATOM	620	N	GLY	A	60	46.219	27.420	12.108	1.00	0.00	N
ATOM	621	H	GLY	A	60	46.725	28.278	11.920	1.00	0.00	H
ATOM	622	CA	GLY	A	60	46.395	26.329	11.149	1.00	0.00	C
ATOM	623	HA1	GLY	A	60	45.675	25.533	11.345	1.00	0.00	H
ATOM	624	HA2	GLY	A	60	46.230	26.719	10.145	1.00	0.00	H
ATOM	625	C	GLY	A	60	47.803	25.732	11.187	1.00	0.00	C
ATOM	626	O	GLY	A	60	48.604	26.025	12.077	1.00	0.00	O
ATOM	627	N	MET	A	61	48.114	24.889	10.200	1.00	0.00	N
ATOM	628	H	MET	A	61	47.448	24.786	9.441	1.00	0.00	H
ATOM	629	CA	MET	A	61	49.387	24.163	10.108	1.00	0.00	C
ATOM	630	HA	MET	A	61	50.144	24.733	10.643	1.00	0.00	H
ATOM	631	CB	MET	A	61	49.227	22.795	10.788	1.00	0.00	C
ATOM	632	HB1	MET	A	61	48.769	22.937	11.767	1.00	0.00	H
ATOM	633	HB2	MET	A	61	48.562	22.177	10.185	1.00	0.00	H
ATOM	634	CG	MET	A	61	50.549	22.043	10.975	1.00	0.00	C
ATOM	635	HG1	MET	A	61	51.125	22.092	10.051	1.00	0.00	H
ATOM	636	HG2	MET	A	61	51.133	22.535	11.756	1.00	0.00	H
ATOM	637	SD	MET	A	61	50.341	20.291	11.394	1.00	0.00	S
ATOM	638	CE	MET	A	61	49.485	20.425	12.982	1.00	0.00	C
ATOM	639	HE1	MET	A	61	49.356	19.429	13.403	1.00	0.00	H
ATOM	640	HE2	MET	A	61	50.072	21.033	13.670	1.00	0.00	H
ATOM	641	HE3	MET	A	61	48.501	20.873	12.845	1.00	0.00	H
ATOM	642	C	MET	A	61	49.834	24.000	8.651	1.00	0.00	C
ATOM	643	O	MET	A	61	49.085	23.458	7.830	1.00	0.00	O
ATOM	644	N	THR	A	62	51.070	24.399	8.333	1.00	0.00	N
ATOM	645	H	THR	A	62	51.644	24.781	9.083	1.00	0.00	H

ATOM	646	CA	THR	A	62	51.656	24.355	6.981	1.00	0.00	C
ATOM	647	HA	THR	A	62	51.033	23.760	6.320	1.00	0.00	H
ATOM	648	CB	THR	A	62	51.702	25.766	6.384	1.00	0.00	C
ATOM	649	HB	THR	A	62	52.339	26.412	6.989	1.00	0.00	H
ATOM	650	CG2	THR	A	62	52.189	25.776	4.935	1.00	0.00	C
ATOM	651	HG21	THR	A	62	52.135	26.791	4.541	1.00	0.00	H
ATOM	652	HG22	THR	A	62	53.226	25.447	4.891	1.00	0.00	H
ATOM	653	HG23	THR	A	62	51.573	25.120	4.322	1.00	0.00	H
ATOM	654	OG1	THR	A	62	50.388	26.277	6.400	1.00	0.00	O
ATOM	655	HG1	THR	A	62	50.264	26.654	7.288	1.00	0.00	H
ATOM	656	C	THR	A	62	53.033	23.706	7.055	1.00	0.00	C
ATOM	657	O	THR	A	62	53.894	24.179	7.792	1.00	0.00	O
ATOM	658	N	ASN	A	63	53.228	22.597	6.334	1.00	0.00	N
ATOM	659	H	ASN	A	63	52.461	22.263	5.768	1.00	0.00	H
ATOM	660	CA	ASN	A	63	54.445	21.761	6.376	1.00	0.00	C
ATOM	661	HA	ASN	A	63	54.218	20.839	5.841	1.00	0.00	H
ATOM	662	CB	ASN	A	63	55.620	22.446	5.651	1.00	0.00	C
ATOM	663	HB1	ASN	A	63	55.893	23.355	6.185	1.00	0.00	H
ATOM	664	HB2	ASN	A	63	56.486	21.784	5.661	1.00	0.00	H
ATOM	665	CG	ASN	A	63	55.306	22.775	4.202	1.00	0.00	C
ATOM	666	OD1	ASN	A	63	54.923	21.909	3.419	1.00	0.00	O
ATOM	667	ND2	ASN	A	63	55.447	24.028	3.817	1.00	0.00	N
ATOM	668	HD21	ASN	A	63	55.832	24.710	4.460	1.00	0.00	H
ATOM	669	HD22	ASN	A	63	55.131	24.286	2.892	1.00	0.00	H
ATOM	670	C	ASN	A	63	54.817	21.352	7.818	1.00	0.00	C
ATOM	671	O	ASN	A	63	55.942	21.567	8.271	1.00	0.00	O
ATOM	672	N	HIS	A	64	53.824	20.892	8.590	1.00	0.00	N
ATOM	673	H	HIS	A	64	52.937	20.711	8.136	1.00	0.00	H
ATOM	674	CA	HIS	A	64	53.891	20.570	10.028	1.00	0.00	C
ATOM	675	HA	HIS	A	64	52.929	20.141	10.305	1.00	0.00	H
ATOM	676	CB	HIS	A	64	54.953	19.494	10.313	1.00	0.00	C
ATOM	677	HB1	HIS	A	64	55.941	19.928	10.204	1.00	0.00	H
ATOM	678	HB2	HIS	A	64	54.857	19.179	11.352	1.00	0.00	H
ATOM	679	CG	HIS	A	64	54.891	18.262	9.457	1.00	0.00	C
ATOM	680	ND1	HIS	A	64	53.773	17.454	9.248	1.00	0.00	N
ATOM	681	CE1	HIS	A	64	54.225	16.389	8.567	1.00	0.00	C
ATOM	682	HE1	HIS	A	64	53.632	15.531	8.293	1.00	0.00	H
ATOM	683	NE2	HIS	A	64	55.529	16.520	8.284	1.00	0.00	N
ATOM	684	HE2	HIS	A	64	56.099	15.827	7.800	1.00	0.00	H
ATOM	685	CD2	HIS	A	64	55.970	17.685	8.861	1.00	0.00	C
ATOM	686	HD2	HIS	A	64	56.982	18.053	8.885	1.00	0.00	H
ATOM	687	C	HIS	A	64	54.111	21.785	10.965	1.00	0.00	C
ATOM	688	O	HIS	A	64	53.916	21.651	12.169	1.00	0.00	O
ATOM	689	N	GLN	A	65	54.510	22.957	10.461	1.00	0.00	N
ATOM	690	H	GLN	A	65	54.602	23.044	9.457	1.00	0.00	H
ATOM	691	CA	GLN	A	65	54.744	24.158	11.271	1.00	0.00	C
ATOM	692	HA	GLN	A	65	55.168	23.856	12.228	1.00	0.00	H
ATOM	693	CB	GLN	A	65	55.742	25.102	10.570	1.00	0.00	C
ATOM	694	HB1	GLN	A	65	55.890	25.985	11.194	1.00	0.00	H
ATOM	695	HB2	GLN	A	65	55.320	25.433	9.622	1.00	0.00	H
ATOM	696	CG	GLN	A	65	57.113	24.453	10.311	1.00	0.00	C
ATOM	697	HG1	GLN	A	65	56.994	23.588	9.664	1.00	0.00	H
ATOM	698	HG2	GLN	A	65	57.529	24.120	11.263	1.00	0.00	H
ATOM	699	CD	GLN	A	65	58.104	25.400	9.635	1.00	0.00	C

```
ATOM    700  OE1 GLN A  65      57.935  25.807   8.489  1.00  0.00           O
ATOM    701  NE2 GLN A  65      59.174  25.772  10.302  1.00  0.00           N
ATOM    702 HE21 GLN A  65      59.401  25.355  11.195  1.00  0.00           H
ATOM    703 HE22 GLN A  65      59.822  26.424   9.876  1.00  0.00           H
ATOM    704  C   GLN A  65      53.418  24.878  11.558  1.00  0.00           C
ATOM    705  O   GLN A  65      52.488  24.806  10.755  1.00  0.00           O
ATOM    706  N   LEU A  66      53.324  25.583  12.690  1.00  0.00           N
ATOM    707  H   LEU A  66      54.128  25.615  13.306  1.00  0.00           H
ATOM    708  CA  LEU A  66      52.147  26.386  13.058  1.00  0.00           C
ATOM    709  HA  LEU A  66      51.254  25.770  12.934  1.00  0.00           H
ATOM    710  CB  LEU A  66      52.303  26.781  14.542  1.00  0.00           C
ATOM    711  HB1 LEU A  66      52.533  25.888  15.126  1.00  0.00           H
ATOM    712  HB2 LEU A  66      53.161  27.452  14.619  1.00  0.00           H
ATOM    713  CG  LEU A  66      51.079  27.473  15.178  1.00  0.00           C
ATOM    714  HG  LEU A  66      50.760  28.305  14.549  1.00  0.00           H
ATOM    715  CD1 LEU A  66      49.904  26.510  15.382  1.00  0.00           C
ATOM    716 HD11 LEU A  66      49.067  27.040  15.832  1.00  0.00           H
ATOM    717 HD12 LEU A  66      49.584  26.090  14.432  1.00  0.00           H
ATOM    718 HD13 LEU A  66      50.200  25.705  16.054  1.00  0.00           H
ATOM    719  CD2 LEU A  66      51.465  28.028  16.551  1.00  0.00           C
ATOM    720 HD21 LEU A  66      50.637  28.602  16.960  1.00  0.00           H
ATOM    721 HD22 LEU A  66      51.714  27.217  17.235  1.00  0.00           H
ATOM    722 HD23 LEU A  66      52.323  28.683  16.444  1.00  0.00           H
ATOM    723  C   LEU A  66      52.015  27.613  12.133  1.00  0.00           C
ATOM    724  O   LEU A  66      53.025  28.244  11.812  1.00  0.00           O
ATOM    725  N   SER A  67      50.803  27.992  11.714  1.00  0.00           N
ATOM    726  H   SER A  67      49.992  27.437  11.976  1.00  0.00           H
ATOM    727  CA  SER A  67      50.597  29.062  10.716  1.00  0.00           C
ATOM    728  HA  SER A  67      51.361  29.824  10.872  1.00  0.00           H
ATOM    729  CB  SER A  67      50.810  28.497   9.296  1.00  0.00           C
ATOM    730  HB1 SER A  67      50.685  29.300   8.571  1.00  0.00           H
ATOM    731  HB2 SER A  67      51.839  28.141   9.220  1.00  0.00           H
ATOM    732  OG  SER A  67      49.942  27.418   8.967  1.00  0.00           O
ATOM    733  HG  SER A  67      49.095  27.765   8.615  1.00  0.00           H
ATOM    734  C   SER A  67      49.253  29.814  10.830  1.00  0.00           C
ATOM    735  O   SER A  67      48.338  29.384  11.540  1.00  0.00           O
ATOM    736  N   THR A  68      49.136  30.966  10.143  1.00  0.00           N
ATOM    737  H   THR A  68      49.937  31.285   9.608  1.00  0.00           H
ATOM    738  CA  THR A  68      47.920  31.811  10.116  1.00  0.00           C
ATOM    739  HA  THR A  68      47.480  31.803  11.108  1.00  0.00           H
ATOM    740  CB  THR A  68      48.233  33.281   9.812  1.00  0.00           C
ATOM    741  HB  THR A  68      47.299  33.847   9.814  1.00  0.00           H
ATOM    742  CG2 THR A  68      49.155  33.911  10.855  1.00  0.00           C
ATOM    743 HG21 THR A  68      49.303  34.964  10.617  1.00  0.00           H
ATOM    744 HG22 THR A  68      48.695  33.836  11.841  1.00  0.00           H
ATOM    745 HG23 THR A  68      50.123  33.411  10.868  1.00  0.00           H
ATOM    746  OG1 THR A  68      48.834  33.410   8.544  1.00  0.00           O
ATOM    747  HG1 THR A  68      49.028  32.518   8.198  1.00  0.00           H
ATOM    748  C   THR A  68      46.825  31.273   9.193  1.00  0.00           C
ATOM    749  O   THR A  68      47.101  30.452   8.313  1.00  0.00           O
ATOM    750  N   THR A  69      45.590  31.752   9.381  1.00  0.00           N
ATOM    751  H   THR A  69      45.463  32.440  10.119  1.00  0.00           H
ATOM    752  CA  THR A  69      44.404  31.387   8.587  1.00  0.00           C
ATOM    753  HA  THR A  69      44.709  30.726   7.777  1.00  0.00           H
```

ATOM	754	CB	THR	A	69	43.365	30.621	9.422	1.00	0.00	C
ATOM	755	HB	THR	A	69	42.472	30.471	8.811	1.00	0.00	H
ATOM	756	CG2	THR	A	69	43.884	29.252	9.864	1.00	0.00	C
ATOM	757	HG21	THR	A	69	43.113	28.734	10.436	1.00	0.00	H
ATOM	758	HG22	THR	A	69	44.133	28.651	8.989	1.00	0.00	H
ATOM	759	HG23	THR	A	69	44.771	29.361	10.488	1.00	0.00	H
ATOM	760	OG1	THR	A	69	43.011	31.334	10.584	1.00	0.00	O
ATOM	761	HG1	THR	A	69	43.791	31.329	11.168	1.00	0.00	H
ATOM	762	C	THR	A	69	43.775	32.608	7.926	1.00	0.00	C
ATOM	763	O	THR	A	69	43.526	32.551	6.720	1.00	0.00	O
ATOM	764	N	GLU	A	70	43.589	33.716	8.647	1.00	0.00	N
ATOM	765	H	GLU	A	70	43.885	33.718	9.621	1.00	0.00	H
ATOM	766	CA	GLU	A	70	43.016	34.960	8.121	1.00	0.00	C
ATOM	767	HA	GLU	A	70	42.589	34.764	7.140	1.00	0.00	H
ATOM	768	CB	GLU	A	70	41.862	35.469	9.004	1.00	0.00	C
ATOM	769	HB1	GLU	A	70	42.247	35.725	9.992	1.00	0.00	H
ATOM	770	HB2	GLU	A	70	41.460	36.378	8.554	1.00	0.00	H
ATOM	771	CG	GLU	A	70	40.711	34.462	9.165	1.00	0.00	C
ATOM	772	HG1	GLU	A	70	41.043	33.652	9.813	1.00	0.00	H
ATOM	773	HG2	GLU	A	70	39.873	34.960	9.652	1.00	0.00	H
ATOM	774	CD	GLU	A	70	40.232	33.859	7.844	1.00	0.00	C
ATOM	775	OE1	GLU	A	70	39.856	34.600	6.904	1.00	0.00	O
ATOM	776	OE2	GLU	A	70	40.224	32.615	7.720	1.00	0.00	O
ATOM	777	C	GLU	A	70	44.079	36.052	7.936	1.00	0.00	C
ATOM	778	O	GLU	A	70	45.031	36.176	8.709	1.00	0.00	O
ATOM	779	N	TRP	A	71	43.904	36.861	6.890	1.00	0.00	N
ATOM	780	H	TRP	A	71	43.095	36.695	6.311	1.00	0.00	H
ATOM	781	CA	TRP	A	71	44.758	37.984	6.479	1.00	0.00	C
ATOM	782	HA	TRP	A	71	45.697	37.592	6.084	1.00	0.00	H
ATOM	783	CB	TRP	A	71	44.017	38.725	5.346	1.00	0.00	C
ATOM	784	HB1	TRP	A	71	44.611	39.591	5.049	1.00	0.00	H
ATOM	785	HB2	TRP	A	71	43.959	38.065	4.479	1.00	0.00	H
ATOM	786	CG	TRP	A	71	42.622	39.186	5.679	1.00	0.00	C
ATOM	787	CD1	TRP	A	71	41.532	38.389	5.743	1.00	0.00	C
ATOM	788	HD1	TRP	A	71	41.517	37.330	5.529	1.00	0.00	H
ATOM	789	NE1	TRP	A	71	40.445	39.103	6.190	1.00	0.00	N
ATOM	790	HE1	TRP	A	71	39.547	38.664	6.375	1.00	0.00	H
ATOM	791	CE2	TRP	A	71	40.756	40.427	6.390	1.00	0.00	C
ATOM	792	CZ2	TRP	A	71	40.005	41.531	6.817	1.00	0.00	C
ATOM	793	HZ2	TRP	A	71	38.973	41.409	7.112	1.00	0.00	H
ATOM	794	CH2	TRP	A	71	40.617	42.793	6.864	1.00	0.00	C
ATOM	795	HH2	TRP	A	71	40.059	43.655	7.204	1.00	0.00	H
ATOM	796	CZ3	TRP	A	71	41.963	42.930	6.482	1.00	0.00	C
ATOM	797	HZ3	TRP	A	71	42.434	43.901	6.521	1.00	0.00	H
ATOM	798	CE3	TRP	A	71	42.714	41.808	6.079	1.00	0.00	C
ATOM	799	HE3	TRP	A	71	43.753	41.927	5.813	1.00	0.00	H
ATOM	800	CD2	TRP	A	71	42.135	40.523	6.027	1.00	0.00	C
ATOM	801	C	TRP	A	71	45.114	38.950	7.625	1.00	0.00	C
ATOM	802	O	TRP	A	71	46.245	39.444	7.698	1.00	0.00	O
ATOM	803	N	ASN	A	72	44.164	39.195	8.532	1.00	0.00	N
ATOM	804	H	ASN	A	72	43.268	38.740	8.416	1.00	0.00	H
ATOM	805	CA	ASN	A	72	44.315	40.091	9.675	1.00	0.00	C
ATOM	806	HA	ASN	A	72	45.136	40.770	9.450	1.00	0.00	H
ATOM	807	CB	ASN	A	72	43.074	40.998	9.802	1.00	0.00	C

```
ATOM    808  HB1 ASN A  72      43.242  41.712  10.608  1.00  0.00           H
ATOM    809  HB2 ASN A  72      42.980  41.578   8.886  1.00  0.00           H
ATOM    810  CG  ASN A  72      41.738  40.305  10.051  1.00  0.00           C
ATOM    811  OD1 ASN A  72      41.610  39.089  10.110  1.00  0.00           O
ATOM    812  ND2 ASN A  72      40.686  41.088  10.185  1.00  0.00           N
ATOM    813 HD21 ASN A  72      40.763  42.092  10.080  1.00  0.00           H
ATOM    814 HD22 ASN A  72      39.776  40.670  10.317  1.00  0.00           H
ATOM    815  C   ASN A  72      44.720  39.399  10.995  1.00  0.00           C
ATOM    816  O   ASN A  72      44.793  40.088  12.008  1.00  0.00           O
ATOM    817  N   ASP A  73      45.068  38.102  11.020  1.00  0.00           N
ATOM    818  H   ASP A  73      44.972  37.552  10.171  1.00  0.00           H
ATOM    819  CA  ASP A  73      45.507  37.406  12.251  1.00  0.00           C
ATOM    820  HA  ASP A  73      44.675  37.383  12.956  1.00  0.00           H
ATOM    821  CB  ASP A  73      45.931  35.955  11.943  1.00  0.00           C
ATOM    822  HB1 ASP A  73      46.535  35.941  11.034  1.00  0.00           H
ATOM    823  HB2 ASP A  73      46.569  35.605  12.756  1.00  0.00           H
ATOM    824  CG  ASP A  73      44.784  34.943  11.822  1.00  0.00           C
ATOM    825  OD1 ASP A  73      43.725  35.116  12.462  1.00  0.00           O
ATOM    826  OD2 ASP A  73      44.989  33.903  11.153  1.00  0.00           O
ATOM    827  C   ASP A  73      46.668  38.133  12.962  1.00  0.00           C
ATOM    828  O   ASP A  73      46.590  38.405  14.157  1.00  0.00           O
ATOM    829  N   GLU A  74      47.708  38.537  12.222  1.00  0.00           N
ATOM    830  H   GLU A  74      47.704  38.302  11.241  1.00  0.00           H
ATOM    831  CA  GLU A  74      48.870  39.261  12.773  1.00  0.00           C
ATOM    832  HA  GLU A  74      49.204  38.724  13.662  1.00  0.00           H
ATOM    833  CB  GLU A  74      50.035  39.262  11.765  1.00  0.00           C
ATOM    834  HB1 GLU A  74      49.725  39.748  10.839  1.00  0.00           H
ATOM    835  HB2 GLU A  74      50.862  39.830  12.190  1.00  0.00           H
ATOM    836  CG  GLU A  74      50.529  37.842  11.459  1.00  0.00           C
ATOM    837  HG1 GLU A  74      50.605  37.279  12.389  1.00  0.00           H
ATOM    838  HG2 GLU A  74      49.805  37.341  10.815  1.00  0.00           H
ATOM    839  CD  GLU A  74      51.901  37.842  10.785  1.00  0.00           C
ATOM    840  OE1 GLU A  74      51.998  37.421   9.611  1.00  0.00           O
ATOM    841  OE2 GLU A  74      52.910  38.159  11.458  1.00  0.00           O
ATOM    842  C   GLU A  74      48.555  40.700  13.237  1.00  0.00           C
ATOM    843  O   GLU A  74      49.417  41.352  13.834  1.00  0.00           O
ATOM    844  N   THR A  75      47.331  41.186  12.985  1.00  0.00           N
ATOM    845  H   THR A  75      46.686  40.562  12.514  1.00  0.00           H
ATOM    846  CA  THR A  75      46.778  42.457  13.482  1.00  0.00           C
ATOM    847  HA  THR A  75      47.588  43.110  13.813  1.00  0.00           H
ATOM    848  CB  THR A  75      46.019  43.161  12.349  1.00  0.00           C
ATOM    849  HB  THR A  75      45.224  42.519  11.969  1.00  0.00           H
ATOM    850  CG2 THR A  75      45.400  44.489  12.779  1.00  0.00           C
ATOM    851 HG21 THR A  75      44.945  44.979  11.919  1.00  0.00           H
ATOM    852 HG22 THR A  75      44.625  44.320  13.527  1.00  0.00           H
ATOM    853 HG23 THR A  75      46.171  45.131  13.197  1.00  0.00           H
ATOM    854  OG1 THR A  75      46.927  43.449  11.308  1.00  0.00           O
ATOM    855  HG1 THR A  75      47.160  42.617  10.861  1.00  0.00           H
ATOM    856  C   THR A  75      45.865  42.210  14.687  1.00  0.00           C
ATOM    857  O   THR A  75      45.932  42.954  15.664  1.00  0.00           O
ATOM    858  N   LEU A  76      45.052  41.149  14.655  1.00  0.00           N
ATOM    859  H   LEU A  76      45.050  40.578  13.818  1.00  0.00           H
ATOM    860  CA  LEU A  76      44.157  40.736  15.743  1.00  0.00           C
ATOM    861  HA  LEU A  76      43.560  41.591  16.055  1.00  0.00           H
```

ATOM	862	CB	LEU	A	76	43.222	39.625	15.224	1.00	0.00	C
ATOM	863	HB1	LEU	A	76	43.837	38.836	14.788	1.00	0.00	H
ATOM	864	HB2	LEU	A	76	42.687	39.195	16.071	1.00	0.00	H
ATOM	865	CG	LEU	A	76	42.180	40.083	14.180	1.00	0.00	C
ATOM	866	HG	LEU	A	76	42.680	40.610	13.368	1.00	0.00	H
ATOM	867	CD1	LEU	A	76	41.458	38.867	13.583	1.00	0.00	C
ATOM	868	HD11	LEU	A	76	40.706	39.195	12.865	1.00	0.00	H
ATOM	869	HD12	LEU	A	76	42.176	38.231	13.064	1.00	0.00	H
ATOM	870	HD13	LEU	A	76	40.970	38.292	14.372	1.00	0.00	H
ATOM	871	CD2	LEU	A	76	41.142	41.033	14.793	1.00	0.00	C
ATOM	872	HD21	LEU	A	76	40.401	41.293	14.040	1.00	0.00	H
ATOM	873	HD22	LEU	A	76	40.643	40.551	15.635	1.00	0.00	H
ATOM	874	HD23	LEU	A	76	41.620	41.952	15.128	1.00	0.00	H
ATOM	875	C	LEU	A	76	44.934	40.283	16.986	1.00	0.00	C
ATOM	876	O	LEU	A	76	44.573	40.661	18.099	1.00	0.00	O
ATOM	877	N	TYR	A	77	46.064	39.590	16.818	1.00	0.00	N
ATOM	878	H	TYR	A	77	46.281	39.244	15.887	1.00	0.00	H
ATOM	879	CA	TYR	A	77	46.986	39.255	17.917	1.00	0.00	C
ATOM	880	HA	TYR	A	77	46.444	38.680	18.667	1.00	0.00	H
ATOM	881	CB	TYR	A	77	48.142	38.396	17.367	1.00	0.00	C
ATOM	882	HB1	TYR	A	77	48.656	38.968	16.592	1.00	0.00	H
ATOM	883	HB2	TYR	A	77	48.853	38.239	18.177	1.00	0.00	H
ATOM	884	CG	TYR	A	77	47.790	37.021	16.806	1.00	0.00	C
ATOM	885	CD1	TYR	A	77	46.696	36.287	17.307	1.00	0.00	C
ATOM	886	HD1	TYR	A	77	46.065	36.693	18.082	1.00	0.00	H
ATOM	887	CE1	TYR	A	77	46.407	35.007	16.805	1.00	0.00	C
ATOM	888	HE1	TYR	A	77	45.573	34.447	17.197	1.00	0.00	H
ATOM	889	CZ	TYR	A	77	47.199	34.443	15.787	1.00	0.00	C
ATOM	890	OH	TYR	A	77	46.885	33.202	15.327	1.00	0.00	O
ATOM	891	HH	TYR	A	77	47.491	32.840	14.669	1.00	0.00	H
ATOM	892	CE2	TYR	A	77	48.311	35.160	15.291	1.00	0.00	C
ATOM	893	HE2	TYR	A	77	48.934	34.730	14.521	1.00	0.00	H
ATOM	894	CD2	TYR	A	77	48.602	36.441	15.806	1.00	0.00	C
ATOM	895	HD2	TYR	A	77	49.450	36.988	15.422	1.00	0.00	H
ATOM	896	C	TYR	A	77	47.533	40.494	18.660	1.00	0.00	C
ATOM	897	O	TYR	A	77	47.962	40.396	19.809	1.00	0.00	O
ATOM	898	N	GLN	A	78	47.486	41.678	18.045	1.00	0.00	N
ATOM	899	H	GLN	A	78	47.097	41.720	17.111	1.00	0.00	H
ATOM	900	CA	GLN	A	78	47.889	42.932	18.685	1.00	0.00	C
ATOM	901	HA	GLN	A	78	48.678	42.732	19.413	1.00	0.00	H
ATOM	902	CB	GLN	A	78	48.471	43.878	17.625	1.00	0.00	C
ATOM	903	HB1	GLN	A	78	47.702	44.126	16.901	1.00	0.00	H
ATOM	904	HB2	GLN	A	78	48.780	44.804	18.111	1.00	0.00	H
ATOM	905	CG	GLN	A	78	49.685	43.260	16.899	1.00	0.00	C
ATOM	906	HG1	GLN	A	78	50.485	43.112	17.625	1.00	0.00	H
ATOM	907	HG2	GLN	A	78	49.429	42.287	16.481	1.00	0.00	H
ATOM	908	CD	GLN	A	78	50.207	44.113	15.745	1.00	0.00	C
ATOM	909	OE1	GLN	A	78	49.505	44.932	15.160	1.00	0.00	O
ATOM	910	NE2	GLN	A	78	51.464	43.985	15.385	1.00	0.00	N
ATOM	911	HE21	GLN	A	78	52.049	43.320	15.879	1.00	0.00	H
ATOM	912	HE22	GLN	A	78	51.795	44.464	14.558	1.00	0.00	H
ATOM	913	C	GLN	A	78	46.725	43.572	19.466	1.00	0.00	C
ATOM	914	O	GLN	A	78	46.977	44.259	20.453	1.00	0.00	O
ATOM	915	N	GLU	A	79	45.464	43.293	19.107	1.00	0.00	N

ATOM	916	H	GLU	A	79	45.310	42.678	18.317	1.00	0.00	H
ATOM	917	CA	GLU	A	79	44.276	43.710	19.872	1.00	0.00	C
ATOM	918	HA	GLU	A	79	44.337	44.780	20.071	1.00	0.00	H
ATOM	919	CB	GLU	A	79	42.983	43.412	19.083	1.00	0.00	C
ATOM	920	HB1	GLU	A	79	42.858	42.331	19.020	1.00	0.00	H
ATOM	921	HB2	GLU	A	79	42.133	43.790	19.652	1.00	0.00	H
ATOM	922	CG	GLU	A	79	42.911	43.967	17.651	1.00	0.00	C
ATOM	923	HG1	GLU	A	79	43.833	43.751	17.113	1.00	0.00	H
ATOM	924	HG2	GLU	A	79	42.107	43.445	17.133	1.00	0.00	H
ATOM	925	CD	GLU	A	79	42.627	45.464	17.568	1.00	0.00	C
ATOM	926	OE1	GLU	A	79	42.816	46.201	18.558	1.00	0.00	O
ATOM	927	OE2	GLU	A	79	42.215	45.931	16.480	1.00	0.00	O
ATOM	928	C	GLU	A	79	44.201	42.969	21.219	1.00	0.00	C
ATOM	929	O	GLU	A	79	43.894	43.569	22.255	1.00	0.00	O
ATOM	930	N	PHE	A	80	44.528	41.668	21.203	1.00	0.00	N
ATOM	931	H	PHE	A	80	44.723	41.250	20.301	1.00	0.00	H
ATOM	932	CA	PHE	A	80	44.670	40.809	22.383	1.00	0.00	C
ATOM	933	HA	PHE	A	80	43.735	40.803	22.943	1.00	0.00	H
ATOM	934	CB	PHE	A	80	44.985	39.374	21.913	1.00	0.00	C
ATOM	935	HB1	PHE	A	80	45.542	39.408	20.976	1.00	0.00	H
ATOM	936	HB2	PHE	A	80	45.649	38.920	22.651	1.00	0.00	H
ATOM	937	CG	PHE	A	80	43.800	38.437	21.732	1.00	0.00	C
ATOM	938	CD1	PHE	A	80	43.919	37.096	22.148	1.00	0.00	C
ATOM	939	HD1	PHE	A	80	44.852	36.739	22.559	1.00	0.00	H
ATOM	940	CE1	PHE	A	80	42.833	36.212	22.029	1.00	0.00	C
ATOM	941	HE1	PHE	A	80	42.929	35.189	22.366	1.00	0.00	H
ATOM	942	CZ	PHE	A	80	41.627	36.657	21.462	1.00	0.00	C
ATOM	943	HZ	PHE	A	80	40.794	35.977	21.365	1.00	0.00	H
ATOM	944	CE2	PHE	A	80	41.513	37.983	21.008	1.00	0.00	C
ATOM	945	HE2	PHE	A	80	40.591	38.318	20.555	1.00	0.00	H
ATOM	946	CD2	PHE	A	80	42.594	38.873	21.145	1.00	0.00	C
ATOM	947	HD2	PHE	A	80	42.493	39.889	20.793	1.00	0.00	H
ATOM	948	C	PHE	A	80	45.762	41.322	23.335	1.00	0.00	C
ATOM	949	O	PHE	A	80	45.578	41.305	24.552	1.00	0.00	O
ATOM	950	N	ASN	A	81	46.873	41.840	22.801	1.00	0.00	N
ATOM	951	H	ASN	A	81	46.992	41.801	21.799	1.00	0.00	H
ATOM	952	CA	ASN	A	81	47.925	42.464	23.611	1.00	0.00	C
ATOM	953	HA	ASN	A	81	48.156	41.799	24.446	1.00	0.00	H
ATOM	954	CB	ASN	A	81	49.206	42.635	22.781	1.00	0.00	C
ATOM	955	HB1	ASN	A	81	48.983	43.140	21.843	1.00	0.00	H
ATOM	956	HB2	ASN	A	81	49.905	43.260	23.338	1.00	0.00	H
ATOM	957	CG	ASN	A	81	49.902	41.315	22.484	1.00	0.00	C
ATOM	958	OD1	ASN	A	81	49.357	40.233	22.673	1.00	0.00	O
ATOM	959	ND2	ASN	A	81	51.139	41.372	22.033	1.00	0.00	N
ATOM	960	HD21	ASN	A	81	51.620	42.260	21.944	1.00	0.00	H
ATOM	961	HD22	ASN	A	81	51.590	40.501	21.798	1.00	0.00	H
ATOM	962	C	ASN	A	81	47.480	43.800	24.226	1.00	0.00	C
ATOM	963	O	ASN	A	81	48.024	44.206	25.255	1.00	0.00	O
ATOM	964	N	GLY	A	82	46.459	44.458	23.663	1.00	0.00	N
ATOM	965	H	GLY	A	82	46.069	44.097	22.799	1.00	0.00	H
ATOM	966	CA	GLY	A	82	45.809	45.609	24.287	1.00	0.00	C
ATOM	967	HA1	GLY	A	82	46.564	46.316	24.633	1.00	0.00	H
ATOM	968	HA2	GLY	A	82	45.154	46.093	23.566	1.00	0.00	H
ATOM	969	C	GLY	A	82	44.984	45.164	25.492	1.00	0.00	C

ATOM	970	O	GLY	A	82	45.137	45.731	26.572	1.00	0.00	O
ATOM	971	N	LEU	A	83	44.191	44.094	25.356	1.00	0.00	N
ATOM	972	H	LEU	A	83	44.123	43.666	24.439	1.00	0.00	H
ATOM	973	CA	LEU	A	83	43.420	43.514	26.467	1.00	0.00	C
ATOM	974	HA	LEU	A	83	42.761	44.285	26.867	1.00	0.00	H
ATOM	975	CB	LEU	A	83	42.562	42.333	25.973	1.00	0.00	C
ATOM	976	HB1	LEU	A	83	43.225	41.554	25.596	1.00	0.00	H
ATOM	977	HB2	LEU	A	83	42.026	41.918	26.828	1.00	0.00	H
ATOM	978	CG	LEU	A	83	41.529	42.676	24.879	1.00	0.00	C
ATOM	979	HG	LEU	A	83	42.036	43.148	24.040	1.00	0.00	H
ATOM	980	CD1	LEU	A	83	40.885	41.382	24.368	1.00	0.00	C
ATOM	981	HD11	LEU	A	83	40.220	41.621	23.540	1.00	0.00	H
ATOM	982	HD12	LEU	A	83	41.651	40.695	24.008	1.00	0.00	H
ATOM	983	HD13	LEU	A	83	40.317	40.900	25.165	1.00	0.00	H
ATOM	984	CD2	LEU	A	83	40.432	43.626	25.382	1.00	0.00	C
ATOM	985	HD21	LEU	A	83	39.714	43.815	24.583	1.00	0.00	H
ATOM	986	HD22	LEU	A	83	39.908	43.178	26.227	1.00	0.00	H
ATOM	987	HD23	LEU	A	83	40.864	44.579	25.686	1.00	0.00	H
ATOM	988	C	LEU	A	83	44.332	43.096	27.631	1.00	0.00	C
ATOM	989	O	LEU	A	83	44.082	43.500	28.764	1.00	0.00	O
ATOM	990	N	LYS	A	84	45.435	42.383	27.368	1.00	0.00	N
ATOM	991	H	LYS	A	84	45.583	42.052	26.420	1.00	0.00	H
ATOM	992	CA	LYS	A	84	46.417	42.025	28.408	1.00	0.00	C
ATOM	993	HA	LYS	A	84	45.875	41.587	29.249	1.00	0.00	H
ATOM	994	CB	LYS	A	84	47.408	40.968	27.885	1.00	0.00	C
ATOM	995	HB1	LYS	A	84	47.850	41.298	26.943	1.00	0.00	H
ATOM	996	HB2	LYS	A	84	48.211	40.882	28.618	1.00	0.00	H
ATOM	997	CG	LYS	A	84	46.775	39.577	27.711	1.00	0.00	C
ATOM	998	HG1	LYS	A	84	45.943	39.467	28.407	1.00	0.00	H
ATOM	999	HG2	LYS	A	84	46.385	39.477	26.697	1.00	0.00	H
ATOM	1000	CD	LYS	A	84	47.778	38.447	28.002	1.00	0.00	C
ATOM	1001	HD1	LYS	A	84	48.149	38.555	29.023	1.00	0.00	H
ATOM	1002	HD2	LYS	A	84	47.254	37.493	27.935	1.00	0.00	H
ATOM	1003	CE	LYS	A	84	48.962	38.434	27.034	1.00	0.00	C
ATOM	1004	HE1	LYS	A	84	48.575	38.268	26.026	1.00	0.00	H
ATOM	1005	HE2	LYS	A	84	49.469	39.401	27.052	1.00	0.00	H
ATOM	1006	NZ	LYS	A	84	49.923	37.360	27.371	1.00	0.00	N
ATOM	1007	HZ1	LYS	A	84	50.534	37.191	26.578	1.00	0.00	H
ATOM	1008	HZ2	LYS	A	84	50.488	37.594	28.181	1.00	0.00	H
ATOM	1009	HZ3	LYS	A	84	49.431	36.491	27.577	1.00	0.00	H
ATOM	1010	C	LYS	A	84	47.183	43.222	29.013	1.00	0.00	C
ATOM	1011	O	LYS	A	84	47.805	43.063	30.064	1.00	0.00	O
ATOM	1012	N	LYS	A	85	47.152	44.410	28.398	1.00	0.00	N
ATOM	1013	H	LYS	A	85	46.659	44.475	27.517	1.00	0.00	H
ATOM	1014	CA	LYS	A	85	47.721	45.645	28.963	1.00	0.00	C
ATOM	1015	HA	LYS	A	85	48.540	45.384	29.638	1.00	0.00	H
ATOM	1016	CB	LYS	A	85	48.329	46.466	27.813	1.00	0.00	C
ATOM	1017	HB1	LYS	A	85	49.088	45.841	27.346	1.00	0.00	H
ATOM	1018	HB2	LYS	A	85	47.563	46.688	27.070	1.00	0.00	H
ATOM	1019	CG	LYS	A	85	48.995	47.784	28.247	1.00	0.00	C
ATOM	1020	HG1	LYS	A	85	48.217	48.510	28.489	1.00	0.00	H
ATOM	1021	HG2	LYS	A	85	49.614	47.613	29.127	1.00	0.00	H
ATOM	1022	CD	LYS	A	85	49.886	48.331	27.118	1.00	0.00	C
ATOM	1023	HD1	LYS	A	85	50.815	47.759	27.078	1.00	0.00	H

ATOM	1024	HD2	LYS	A	85	49.369	48.187	26.168	1.00	0.00	H
ATOM	1025	CE	LYS	A	85	50.196	49.829	27.243	1.00	0.00	C
ATOM	1026	HE1	LYS	A	85	50.705	50.157	26.333	1.00	0.00	H
ATOM	1027	HE2	LYS	A	85	49.250	50.373	27.305	1.00	0.00	H
ATOM	1028	NZ	LYS	A	85	51.029	50.180	28.418	1.00	0.00	N
ATOM	1029	HZ1	LYS	A	85	50.567	49.919	29.285	1.00	0.00	H
ATOM	1030	HZ2	LYS	A	85	51.952	49.758	28.382	1.00	0.00	H
ATOM	1031	HZ3	LYS	A	85	51.140	51.191	28.451	1.00	0.00	H
ATOM	1032	C	LYS	A	85	46.697	46.418	29.815	1.00	0.00	C
ATOM	1033	O	LYS	A	85	47.051	46.873	30.903	1.00	0.00	O
ATOM	1034	N	MET	A	86	45.426	46.489	29.393	1.00	0.00	N
ATOM	1035	H	MET	A	86	45.213	46.145	28.462	1.00	0.00	H
ATOM	1036	CA	MET	A	86	44.319	47.019	30.213	1.00	0.00	C
ATOM	1037	HA	MET	A	86	44.642	47.959	30.667	1.00	0.00	H
ATOM	1038	CB	MET	A	86	43.085	47.316	29.339	1.00	0.00	C
ATOM	1039	HB1	MET	A	86	42.914	46.506	28.628	1.00	0.00	H
ATOM	1040	HB2	MET	A	86	42.212	47.374	29.993	1.00	0.00	H
ATOM	1041	CG	MET	A	86	43.183	48.665	28.608	1.00	0.00	C
ATOM	1042	HG1	MET	A	86	42.173	48.973	28.333	1.00	0.00	H
ATOM	1043	HG2	MET	A	86	43.546	49.416	29.314	1.00	0.00	H
ATOM	1044	SD	MET	A	86	44.208	48.739	27.115	1.00	0.00	S
ATOM	1045	CE	MET	A	86	43.018	48.080	25.918	1.00	0.00	C
ATOM	1046	HE1	MET	A	86	42.139	48.724	25.875	1.00	0.00	H
ATOM	1047	HE2	MET	A	86	43.473	48.031	24.929	1.00	0.00	H
ATOM	1048	HE3	MET	A	86	42.713	47.076	26.214	1.00	0.00	H
ATOM	1049	C	MET	A	86	43.945	46.101	31.393	1.00	0.00	C
ATOM	1050	O	MET	A	86	43.449	46.595	32.405	1.00	0.00	O
ATOM	1051	N	ASN	A	87	44.223	44.796	31.313	1.00	0.00	N
ATOM	1052	H	ASN	A	87	44.485	44.419	30.409	1.00	0.00	H
ATOM	1053	CA	ASN	A	87	44.144	43.861	32.441	1.00	0.00	C
ATOM	1054	HA	ASN	A	87	44.221	44.421	33.373	1.00	0.00	H
ATOM	1055	CB	ASN	A	87	42.770	43.171	32.454	1.00	0.00	C
ATOM	1056	HB1	ASN	A	87	41.981	43.917	32.534	1.00	0.00	H
ATOM	1057	HB2	ASN	A	87	42.633	42.628	31.518	1.00	0.00	H
ATOM	1058	CG	ASN	A	87	42.627	42.189	33.615	1.00	0.00	C
ATOM	1059	OD1	ASN	A	87	43.362	42.224	34.597	1.00	0.00	O
ATOM	1060	ND2	ASN	A	87	41.703	41.258	33.508	1.00	0.00	N
ATOM	1061	HD21	ASN	A	87	41.127	41.217	32.672	1.00	0.00	H
ATOM	1062	HD22	ASN	A	87	41.602	40.551	34.216	1.00	0.00	H
ATOM	1063	C	ASN	A	87	45.288	42.819	32.399	1.00	0.00	C
ATOM	1064	O	ASN	A	87	45.152	41.793	31.723	1.00	0.00	O
ATOM	1065	N	PRO	A	88	46.391	43.040	33.144	1.00	0.00	N
ATOM	1066	CD	PRO	A	88	46.738	44.296	33.794	1.00	0.00	C
ATOM	1067	HD1	PRO	A	88	46.236	44.348	34.761	1.00	0.00	H
ATOM	1068	HD2	PRO	A	88	46.473	45.160	33.183	1.00	0.00	H
ATOM	1069	CG	PRO	A	88	48.251	44.249	33.996	1.00	0.00	C
ATOM	1070	HG1	PRO	A	88	48.571	44.853	34.846	1.00	0.00	H
ATOM	1071	HG2	PRO	A	88	48.757	44.569	33.083	1.00	0.00	H
ATOM	1072	CB	PRO	A	88	48.495	42.762	34.229	1.00	0.00	C
ATOM	1073	HB1	PRO	A	88	48.251	42.513	35.264	1.00	0.00	H
ATOM	1074	HB2	PRO	A	88	49.521	42.478	34.000	1.00	0.00	H
ATOM	1075	CA	PRO	A	88	47.501	42.095	33.273	1.00	0.00	C
ATOM	1076	HA	PRO	A	88	47.977	41.991	32.299	1.00	0.00	H
ATOM	1077	C	PRO	A	88	47.126	40.692	33.768	1.00	0.00	C

ATOM	1078	O	PRO	A	88	47.864	39.749	33.489	1.00	0.00	O
ATOM	1079	N	LYS	A	89	45.995	40.510	34.468	1.00	0.00	N
ATOM	1080	H	LYS	A	89	45.398	41.298	34.690	1.00	0.00	H
ATOM	1081	CA	LYS	A	89	45.587	39.174	34.924	1.00	0.00	C
ATOM	1082	HA	LYS	A	89	46.445	38.695	35.398	1.00	0.00	H
ATOM	1083	CB	LYS	A	89	44.448	39.261	35.948	1.00	0.00	C
ATOM	1084	HB1	LYS	A	89	43.588	39.740	35.479	1.00	0.00	H
ATOM	1085	HB2	LYS	A	89	44.159	38.243	36.218	1.00	0.00	H
ATOM	1086	CG	LYS	A	89	44.818	40.002	37.237	1.00	0.00	C
ATOM	1087	HG1	LYS	A	89	45.772	39.629	37.612	1.00	0.00	H
ATOM	1088	HG2	LYS	A	89	44.918	41.068	37.029	1.00	0.00	H
ATOM	1089	CD	LYS	A	89	43.750	39.788	38.319	1.00	0.00	C
ATOM	1090	HD1	LYS	A	89	43.776	38.746	38.640	1.00	0.00	H
ATOM	1091	HD2	LYS	A	89	44.007	40.410	39.175	1.00	0.00	H
ATOM	1092	CE	LYS	A	89	42.337	40.129	37.820	1.00	0.00	C
ATOM	1093	HE1	LYS	A	89	42.389	41.030	37.203	1.00	0.00	H
ATOM	1094	HE2	LYS	A	89	41.967	39.314	37.192	1.00	0.00	H
ATOM	1095	NZ	LYS	A	89	41.389	40.379	38.927	1.00	0.00	N
ATOM	1096	HZ1	LYS	A	89	40.475	40.625	38.554	1.00	0.00	H
ATOM	1097	HZ2	LYS	A	89	41.275	39.564	39.524	1.00	0.00	H
ATOM	1098	HZ3	LYS	A	89	41.711	41.159	39.489	1.00	0.00	H
ATOM	1099	C	LYS	A	89	45.121	38.252	33.789	1.00	0.00	C
ATOM	1100	O	LYS	A	89	45.158	37.028	33.973	1.00	0.00	O
ATOM	1101	N	LEU	A	90	44.625	38.800	32.674	1.00	0.00	N
ATOM	1102	H	LEU	A	90	44.693	39.804	32.566	1.00	0.00	H
ATOM	1103	CA	LEU	A	90	44.040	38.030	31.575	1.00	0.00	C
ATOM	1104	HA	LEU	A	90	43.288	37.356	31.988	1.00	0.00	H
ATOM	1105	CB	LEU	A	90	43.351	39.008	30.602	1.00	0.00	C
ATOM	1106	HB1	LEU	A	90	42.624	39.606	31.156	1.00	0.00	H
ATOM	1107	HB2	LEU	A	90	44.107	39.689	30.207	1.00	0.00	H
ATOM	1108	CG	LEU	A	90	42.628	38.337	29.414	1.00	0.00	C
ATOM	1109	HG	LEU	A	90	43.335	37.710	28.870	1.00	0.00	H
ATOM	1110	CD1	LEU	A	90	41.453	37.460	29.876	1.00	0.00	C
ATOM	1111	HD11	LEU	A	90	40.922	37.067	29.011	1.00	0.00	H
ATOM	1112	HD12	LEU	A	90	41.816	36.620	30.464	1.00	0.00	H
ATOM	1113	HD13	LEU	A	90	40.759	38.051	30.477	1.00	0.00	H
ATOM	1114	CD2	LEU	A	90	42.125	39.417	28.448	1.00	0.00	C
ATOM	1115	HD21	LEU	A	90	41.638	38.951	27.591	1.00	0.00	H
ATOM	1116	HD22	LEU	A	90	41.413	40.070	28.953	1.00	0.00	H
ATOM	1117	HD23	LEU	A	90	42.967	40.011	28.091	1.00	0.00	H
ATOM	1118	C	LEU	A	90	45.125	37.195	30.885	1.00	0.00	C
ATOM	1119	O	LEU	A	90	46.176	37.719	30.535	1.00	0.00	O
ATOM	1120	N	LYS	A	91	44.874	35.902	30.674	1.00	0.00	N
ATOM	1121	H	LYS	A	91	43.992	35.520	30.988	1.00	0.00	H
ATOM	1122	CA	LYS	A	91	45.799	35.005	29.964	1.00	0.00	C
ATOM	1123	HA	LYS	A	91	46.739	35.522	29.767	1.00	0.00	H
ATOM	1124	CB	LYS	A	91	46.095	33.773	30.839	1.00	0.00	C
ATOM	1125	HB1	LYS	A	91	45.158	33.254	31.053	1.00	0.00	H
ATOM	1126	HB2	LYS	A	91	46.734	33.088	30.279	1.00	0.00	H
ATOM	1127	CG	LYS	A	91	46.788	34.103	32.174	1.00	0.00	C
ATOM	1128	HG1	LYS	A	91	46.148	34.752	32.772	1.00	0.00	H
ATOM	1129	HG2	LYS	A	91	46.915	33.170	32.720	1.00	0.00	H
ATOM	1130	CD	LYS	A	91	48.163	34.767	31.997	1.00	0.00	C
ATOM	1131	HD1	LYS	A	91	48.780	34.137	31.359	1.00	0.00	H

ATOM	1132	HD2	LYS	A	91	48.048	35.746	31.528	1.00	0.00	H
ATOM	1133	CE	LYS	A	91	48.867	34.942	33.345	1.00	0.00	C
ATOM	1134	HE1	LYS	A	91	48.253	35.587	33.978	1.00	0.00	H
ATOM	1135	HE2	LYS	A	91	48.963	33.967	33.831	1.00	0.00	H
ATOM	1136	NZ	LYS	A	91	50.206	35.550	33.178	1.00	0.00	N
ATOM	1137	HZ1	LYS	A	91	50.613	35.778	34.079	1.00	0.00	H
ATOM	1138	HZ2	LYS	A	91	50.864	34.911	32.741	1.00	0.00	H
ATOM	1139	HZ3	LYS	A	91	50.156	36.412	32.642	1.00	0.00	H
ATOM	1140	C	LYS	A	91	45.228	34.604	28.597	1.00	0.00	C
ATOM	1141	O	LYS	A	91	44.043	34.257	28.508	1.00	0.00	O
ATOM	1142	N	THR	A	92	46.050	34.655	27.542	1.00	0.00	N
ATOM	1143	H	THR	A	92	47.025	34.904	27.695	1.00	0.00	H
ATOM	1144	CA	THR	A	92	45.642	34.386	26.153	1.00	0.00	C
ATOM	1145	HA	THR	A	92	44.560	34.297	26.108	1.00	0.00	H
ATOM	1146	CB	THR	A	92	46.036	35.545	25.222	1.00	0.00	C
ATOM	1147	HB	THR	A	92	45.942	35.222	24.183	1.00	0.00	H
ATOM	1148	CG2	THR	A	92	45.120	36.750	25.450	1.00	0.00	C
ATOM	1149	HG21	THR	A	92	45.437	37.579	24.818	1.00	0.00	H
ATOM	1150	HG22	THR	A	92	44.095	36.484	25.194	1.00	0.00	H
ATOM	1151	HG23	THR	A	92	45.158	37.066	26.493	1.00	0.00	H
ATOM	1152	OG1	THR	A	92	47.355	35.973	25.464	1.00	0.00	O
ATOM	1153	HG1	THR	A	92	47.593	36.608	24.766	1.00	0.00	H
ATOM	1154	C	THR	A	92	46.225	33.061	25.680	1.00	0.00	C
ATOM	1155	O	THR	A	92	47.431	32.853	25.759	1.00	0.00	O
ATOM	1156	N	LEU	A	93	45.384	32.149	25.186	1.00	0.00	N
ATOM	1157	H	LEU	A	93	44.398	32.387	25.131	1.00	0.00	H
ATOM	1158	CA	LEU	A	93	45.818	30.879	24.587	1.00	0.00	C
ATOM	1159	HA	LEU	A	93	46.907	30.836	24.594	1.00	0.00	H
ATOM	1160	CB	LEU	A	93	45.303	29.655	25.372	1.00	0.00	C
ATOM	1161	HB1	LEU	A	93	44.221	29.699	25.459	1.00	0.00	H
ATOM	1162	HB2	LEU	A	93	45.536	28.773	24.772	1.00	0.00	H
ATOM	1163	CG	LEU	A	93	45.937	29.455	26.767	1.00	0.00	C
ATOM	1164	HG	LEU	A	93	46.982	29.758	26.730	1.00	0.00	H
ATOM	1165	CD1	LEU	A	93	45.233	30.263	27.870	1.00	0.00	C
ATOM	1166	HD11	LEU	A	93	45.709	30.058	28.829	1.00	0.00	H
ATOM	1167	HD12	LEU	A	93	45.315	31.331	27.680	1.00	0.00	H
ATOM	1168	HD13	LEU	A	93	44.180	29.988	27.924	1.00	0.00	H
ATOM	1169	CD2	LEU	A	93	45.890	27.965	27.142	1.00	0.00	C
ATOM	1170	HD21	LEU	A	93	46.314	27.815	28.135	1.00	0.00	H
ATOM	1171	HD22	LEU	A	93	44.860	27.607	27.132	1.00	0.00	H
ATOM	1172	HD23	LEU	A	93	46.471	27.381	26.428	1.00	0.00	H
ATOM	1173	C	LEU	A	93	45.395	30.808	23.116	1.00	0.00	C
ATOM	1174	O	LEU	A	93	44.413	31.427	22.708	1.00	0.00	O
ATOM	1175	N	LEU	A	94	46.143	30.058	22.310	1.00	0.00	N
ATOM	1176	H	LEU	A	94	46.942	29.577	22.706	1.00	0.00	H
ATOM	1177	CA	LEU	A	94	45.873	29.871	20.885	1.00	0.00	C
ATOM	1178	HA	LEU	A	94	45.116	30.584	20.556	1.00	0.00	H
ATOM	1179	CB	LEU	A	94	47.185	30.140	20.128	1.00	0.00	C
ATOM	1180	HB1	LEU	A	94	47.561	31.121	20.426	1.00	0.00	H
ATOM	1181	HB2	LEU	A	94	47.920	29.396	20.440	1.00	0.00	H
ATOM	1182	CG	LEU	A	94	47.076	30.113	18.593	1.00	0.00	C
ATOM	1183	HG	LEU	A	94	46.738	29.127	18.270	1.00	0.00	H
ATOM	1184	CD1	LEU	A	94	46.092	31.168	18.068	1.00	0.00	C
ATOM	1185	HD11	LEU	A	94	46.139	31.220	16.983	1.00	0.00	H

ATOM	1186	HD12	LEU	A	94	45.076	30.892	18.340	1.00	0.00	H
ATOM	1187	HD13	LEU	A	94	46.332	32.149	18.480	1.00	0.00	H
ATOM	1188	CD2	LEU	A	94	48.477	30.357	18.018	1.00	0.00	C
ATOM	1189	HD21	LEU	A	94	48.450	30.362	16.930	1.00	0.00	H
ATOM	1190	HD22	LEU	A	94	48.863	31.313	18.368	1.00	0.00	H
ATOM	1191	HD23	LEU	A	94	49.144	29.563	18.349	1.00	0.00	H
ATOM	1192	C	LEU	A	94	45.343	28.454	20.651	1.00	0.00	C
ATOM	1193	O	LEU	A	94	46.015	27.491	21.013	1.00	0.00	O
ATOM	1194	N	ALA	A	95	44.161	28.312	20.049	1.00	0.00	N
ATOM	1195	H	ALA	A	95	43.661	29.130	19.720	1.00	0.00	H
ATOM	1196	CA	ALA	A	95	43.614	27.003	19.697	1.00	0.00	C
ATOM	1197	HA	ALA	A	95	44.002	26.273	20.398	1.00	0.00	H
ATOM	1198	CB	ALA	A	95	42.090	27.015	19.851	1.00	0.00	C
ATOM	1199	HB1	ALA	A	95	41.690	26.033	19.594	1.00	0.00	H
ATOM	1200	HB2	ALA	A	95	41.829	27.223	20.886	1.00	0.00	H
ATOM	1201	HB3	ALA	A	95	41.646	27.771	19.205	1.00	0.00	H
ATOM	1202	C	ALA	A	95	44.053	26.589	18.286	1.00	0.00	C
ATOM	1203	O	ALA	A	95	43.982	27.390	17.355	1.00	0.00	O
ATOM	1204	N	ILE	A	96	44.470	25.335	18.102	1.00	0.00	N
ATOM	1205	H	ILE	A	96	44.493	24.725	18.916	1.00	0.00	H
ATOM	1206	CA	ILE	A	96	44.783	24.764	16.777	1.00	0.00	C
ATOM	1207	HA	ILE	A	96	44.803	25.574	16.047	1.00	0.00	H
ATOM	1208	CB	ILE	A	96	46.197	24.128	16.760	1.00	0.00	C
ATOM	1209	HB	ILE	A	96	46.903	24.923	17.009	1.00	0.00	H
ATOM	1210	CG2	ILE	A	96	46.384	23.008	17.803	1.00	0.00	C
ATOM	1211	HG21	ILE	A	96	47.422	22.681	17.824	1.00	0.00	H
ATOM	1212	HG22	ILE	A	96	46.142	23.380	18.796	1.00	0.00	H
ATOM	1213	HG23	ILE	A	96	45.748	22.155	17.570	1.00	0.00	H
ATOM	1214	CG1	ILE	A	96	46.539	23.634	15.334	1.00	0.00	C
ATOM	1215	HG11	ILE	A	96	45.891	22.803	15.056	1.00	0.00	H
ATOM	1216	HG12	ILE	A	96	46.367	24.446	14.632	1.00	0.00	H
ATOM	1217	CD	ILE	A	96	47.989	23.192	15.154	1.00	0.00	C
ATOM	1218	HD1	ILE	A	96	48.185	23.021	14.096	1.00	0.00	H
ATOM	1219	HD2	ILE	A	96	48.643	23.980	15.516	1.00	0.00	H
ATOM	1220	HD3	ILE	A	96	48.183	22.271	15.701	1.00	0.00	H
ATOM	1221	C	ILE	A	96	43.689	23.797	16.311	1.00	0.00	C
ATOM	1222	O	ILE	A	96	43.276	22.919	17.074	1.00	0.00	O
ATOM	1223	N	GLY	A	97	43.276	23.928	15.041	1.00	0.00	N
ATOM	1224	H	GLY	A	97	43.664	24.689	14.490	1.00	0.00	H
ATOM	1225	CA	GLY	A	97	42.418	22.966	14.341	1.00	0.00	C
ATOM	1226	HA1	GLY	A	97	42.826	22.805	13.345	1.00	0.00	H
ATOM	1227	HA2	GLY	A	97	42.419	22.015	14.872	1.00	0.00	H
ATOM	1228	C	GLY	A	97	40.972	23.429	14.152	1.00	0.00	C
ATOM	1229	O	GLY	A	97	40.695	24.280	13.301	1.00	0.00	O
ATOM	1230	N	GLY	A	98	40.048	22.785	14.870	1.00	0.00	N
ATOM	1231	H	GLY	A	98	40.386	22.144	15.570	1.00	0.00	H
ATOM	1232	CA	GLY	A	98	38.591	22.878	14.713	1.00	0.00	C
ATOM	1233	HA1	GLY	A	98	38.131	22.859	15.702	1.00	0.00	H
ATOM	1234	HA2	GLY	A	98	38.318	23.809	14.219	1.00	0.00	H
ATOM	1235	C	GLY	A	98	37.982	21.727	13.907	1.00	0.00	C
ATOM	1236	O	GLY	A	98	38.684	20.929	13.285	1.00	0.00	O
ATOM	1237	N	TRP	A	99	36.651	21.647	13.910	1.00	0.00	N
ATOM	1238	H	TRP	A	99	36.151	22.345	14.439	1.00	0.00	H
ATOM	1239	CA	TRP	A	99	35.859	20.646	13.181	1.00	0.00	C

ATOM	1240	HA	TRP	A	99	36.097	19.654	13.563	1.00	0.00	H
ATOM	1241	CB	TRP	A	99	34.373	20.925	13.453	1.00	0.00	C
ATOM	1242	HB1	TRP	A	99	34.149	20.634	14.478	1.00	0.00	H
ATOM	1243	HB2	TRP	A	99	34.199	21.998	13.367	1.00	0.00	H
ATOM	1244	CG	TRP	A	99	33.399	20.247	12.536	1.00	0.00	C
ATOM	1245	CD1	TRP	A	99	32.562	20.892	11.694	1.00	0.00	C
ATOM	1246	HD1	TRP	A	99	32.494	21.968	11.585	1.00	0.00	H
ATOM	1247	NE1	TRP	A	99	31.835	19.980	10.961	1.00	0.00	N
ATOM	1248	HE1	TRP	A	99	31.172	20.258	10.241	1.00	0.00	H
ATOM	1249	CE2	TRP	A	99	32.161	18.684	11.295	1.00	0.00	C
ATOM	1250	CZ2	TRP	A	99	31.711	17.435	10.839	1.00	0.00	C
ATOM	1251	HZ2	TRP	A	99	30.959	17.375	10.066	1.00	0.00	H
ATOM	1252	CH2	TRP	A	99	32.269	16.267	11.385	1.00	0.00	C
ATOM	1253	HH2	TRP	A	99	31.947	15.296	11.037	1.00	0.00	H
ATOM	1254	CZ3	TRP	A	99	33.272	16.366	12.364	1.00	0.00	C
ATOM	1255	HZ3	TRP	A	99	33.717	15.469	12.767	1.00	0.00	H
ATOM	1256	CE3	TRP	A	99	33.723	17.625	12.803	1.00	0.00	C
ATOM	1257	HE3	TRP	A	99	34.518	17.678	13.528	1.00	0.00	H
ATOM	1258	CD2	TRP	A	99	33.168	18.820	12.298	1.00	0.00	C
ATOM	1259	C	TRP	A	99	36.153	20.626	11.670	1.00	0.00	C
ATOM	1260	O	TRP	A	99	36.439	19.561	11.115	1.00	0.00	O
ATOM	1261	N	ASN	A	100	36.133	21.794	11.015	1.00	0.00	N
ATOM	1262	H	ASN	A	100	35.914	22.630	11.540	1.00	0.00	H
ATOM	1263	CA	ASN	A	100	36.318	21.903	9.561	1.00	0.00	C
ATOM	1264	HA	ASN	A	100	35.601	21.244	9.067	1.00	0.00	H
ATOM	1265	CB	ASN	A	100	36.053	23.348	9.102	1.00	0.00	C
ATOM	1266	HB1	ASN	A	100	36.660	24.033	9.693	1.00	0.00	H
ATOM	1267	HB2	ASN	A	100	36.361	23.450	8.061	1.00	0.00	H
ATOM	1268	CG	ASN	A	100	34.599	23.795	9.187	1.00	0.00	C
ATOM	1269	OD1	ASN	A	100	33.679	23.032	9.441	1.00	0.00	O
ATOM	1270	ND2	ASN	A	100	34.345	25.062	8.937	1.00	0.00	N
ATOM	1271	HD21	ASN	A	100	35.084	25.661	8.587	1.00	0.00	H
ATOM	1272	HD22	ASN	A	100	33.403	25.410	9.028	1.00	0.00	H
ATOM	1273	C	ASN	A	100	37.725	21.481	9.101	1.00	0.00	C
ATOM	1274	O	ASN	A	100	37.883	21.004	7.976	1.00	0.00	O
ATOM	1275	N	PHE	A	101	38.741	21.645	9.959	1.00	0.00	N
ATOM	1276	H	PHE	A	101	38.520	22.027	10.870	1.00	0.00	H
ATOM	1277	CA	PHE	A	101	40.162	21.468	9.635	1.00	0.00	C
ATOM	1278	HA	PHE	A	101	40.424	22.163	8.836	1.00	0.00	H
ATOM	1279	CB	PHE	A	101	40.965	21.841	10.888	1.00	0.00	C
ATOM	1280	HB1	PHE	A	101	40.662	22.839	11.197	1.00	0.00	H
ATOM	1281	HB2	PHE	A	101	40.705	21.153	11.692	1.00	0.00	H
ATOM	1282	CG	PHE	A	101	42.474	21.856	10.737	1.00	0.00	C
ATOM	1283	CD1	PHE	A	101	43.109	22.940	10.103	1.00	0.00	C
ATOM	1284	HD1	PHE	A	101	42.519	23.740	9.684	1.00	0.00	H
ATOM	1285	CE1	PHE	A	101	44.512	23.000	10.039	1.00	0.00	C
ATOM	1286	HE1	PHE	A	101	44.991	23.844	9.566	1.00	0.00	H
ATOM	1287	CZ	PHE	A	101	45.284	21.976	10.612	1.00	0.00	C
ATOM	1288	HZ	PHE	A	101	46.360	22.028	10.581	1.00	0.00	H
ATOM	1289	CE2	PHE	A	101	44.655	20.887	11.236	1.00	0.00	C
ATOM	1290	HE2	PHE	A	101	45.248	20.100	11.681	1.00	0.00	H
ATOM	1291	CD2	PHE	A	101	43.251	20.825	11.297	1.00	0.00	C
ATOM	1292	HD2	PHE	A	101	42.772	19.994	11.792	1.00	0.00	H
ATOM	1293	C	PHE	A	101	40.531	20.059	9.145	1.00	0.00	C

```
ATOM   1294  O   PHE A 101      41.474  19.928   8.366  1.00  0.00           O
ATOM   1295  N   GLY A 102      39.801  19.021   9.567  1.00  0.00           N
ATOM   1296  H   GLY A 102      39.041  19.200  10.208  1.00  0.00           H
ATOM   1297  CA  GLY A 102      40.080  17.628   9.202  1.00  0.00           C
ATOM   1298  HA1 GLY A 102      39.170  17.045   9.320  1.00  0.00           H
ATOM   1299  HA2 GLY A 102      40.401  17.572   8.162  1.00  0.00           H
ATOM   1300  C   GLY A 102      41.171  16.994  10.069  1.00  0.00           C
ATOM   1301  O   GLY A 102      41.799  17.663  10.894  1.00  0.00           O
ATOM   1302  N   THR A 103      41.426  15.693   9.879  1.00  0.00           N
ATOM   1303  H   THR A 103      40.890  15.208   9.165  1.00  0.00           H
ATOM   1304  CA  THR A 103      42.420  14.930  10.662  1.00  0.00           C
ATOM   1305  HA  THR A 103      42.564  15.432  11.620  1.00  0.00           H
ATOM   1306  CB  THR A 103      41.926  13.518  10.983  1.00  0.00           C
ATOM   1307  HB  THR A 103      42.720  13.009  11.532  1.00  0.00           H
ATOM   1308  CG2 THR A 103      40.668  13.521  11.846  1.00  0.00           C
ATOM   1309 HG21 THR A 103      40.400  12.499  12.106  1.00  0.00           H
ATOM   1310 HG22 THR A 103      40.851  14.083  12.758  1.00  0.00           H
ATOM   1311 HG23 THR A 103      39.834  13.979  11.318  1.00  0.00           H
ATOM   1312  OG1 THR A 103      41.672  12.801   9.802  1.00  0.00           O
ATOM   1313  HG1 THR A 103      40.734  12.890   9.576  1.00  0.00           H
ATOM   1314  C   THR A 103      43.803  14.856  10.020  1.00  0.00           C
ATOM   1315  O   THR A 103      44.781  14.931  10.759  1.00  0.00           O
ATOM   1316  N   GLN A 104      43.909  14.794   8.691  1.00  0.00           N
ATOM   1317  H   GLN A 104      43.042  14.781   8.169  1.00  0.00           H
ATOM   1318  CA  GLN A 104      45.133  14.404   7.969  1.00  0.00           C
ATOM   1319  HA  GLN A 104      45.322  13.356   8.210  1.00  0.00           H
ATOM   1320  CB  GLN A 104      44.869  14.512   6.455  1.00  0.00           C
ATOM   1321  HB1 GLN A 104      43.892  14.082   6.229  1.00  0.00           H
ATOM   1322  HB2 GLN A 104      44.850  15.565   6.171  1.00  0.00           H
ATOM   1323  CG  GLN A 104      45.917  13.792   5.590  1.00  0.00           C
ATOM   1324  HG1 GLN A 104      45.693  13.987   4.543  1.00  0.00           H
ATOM   1325  HG2 GLN A 104      46.914  14.181   5.800  1.00  0.00           H
ATOM   1326  CD  GLN A 104      45.886  12.282   5.806  1.00  0.00           C
ATOM   1327  OE1 GLN A 104      44.965  11.597   5.369  1.00  0.00           O
ATOM   1328  NE2 GLN A 104      46.832  11.724   6.527  1.00  0.00           N
ATOM   1329 HE21 GLN A 104      47.640  12.255   6.840  1.00  0.00           H
ATOM   1330 HE22 GLN A 104      46.747  10.731   6.730  1.00  0.00           H
ATOM   1331  C   GLN A 104      46.420  15.164   8.349  1.00  0.00           C
ATOM   1332  O   GLN A 104      47.437  14.521   8.596  1.00  0.00           O
ATOM   1333  N   LYS A 105      46.415  16.503   8.420  1.00  0.00           N
ATOM   1334  H   LYS A 105      45.564  17.001   8.184  1.00  0.00           H
ATOM   1335  CA  LYS A 105      47.609  17.291   8.783  1.00  0.00           C
ATOM   1336  HA  LYS A 105      48.401  17.082   8.062  1.00  0.00           H
ATOM   1337  CB  LYS A 105      47.295  18.797   8.757  1.00  0.00           C
ATOM   1338  HB1 LYS A 105      46.396  18.999   9.339  1.00  0.00           H
ATOM   1339  HB2 LYS A 105      48.124  19.322   9.236  1.00  0.00           H
ATOM   1340  CG  LYS A 105      47.130  19.373   7.345  1.00  0.00           C
ATOM   1341  HG1 LYS A 105      48.037  19.179   6.771  1.00  0.00           H
ATOM   1342  HG2 LYS A 105      46.282  18.907   6.843  1.00  0.00           H
ATOM   1343  CD  LYS A 105      46.905  20.887   7.446  1.00  0.00           C
ATOM   1344  HD1 LYS A 105      45.966  21.085   7.964  1.00  0.00           H
ATOM   1345  HD2 LYS A 105      47.718  21.322   8.025  1.00  0.00           H
ATOM   1346  CE  LYS A 105      46.877  21.523   6.055  1.00  0.00           C
ATOM   1347  HE1 LYS A 105      47.793  21.243   5.527  1.00  0.00           H
```

ATOM	1348	HE2	LYS	A	105	46.029	21.124	5.492	1.00	0.00	H
ATOM	1349	NZ	LYS	A	105	46.786	23.001	6.123	1.00	0.00	N
ATOM	1350	HZ1	LYS	A	105	46.932	23.409	5.206	1.00	0.00	H
ATOM	1351	HZ2	LYS	A	105	45.878	23.299	6.467	1.00	0.00	H
ATOM	1352	HZ3	LYS	A	105	47.495	23.397	6.733	1.00	0.00	H
ATOM	1353	C	LYS	A	105	48.178	16.918	10.162	1.00	0.00	C
ATOM	1354	O	LYS	A	105	49.393	16.787	10.310	1.00	0.00	O
ATOM	1355	N	PHE	A	106	47.316	16.731	11.169	1.00	0.00	N
ATOM	1356	H	PHE	A	106	46.338	16.899	10.989	1.00	0.00	H
ATOM	1357	CA	PHE	A	106	47.717	16.235	12.485	1.00	0.00	C
ATOM	1358	HA	PHE	A	106	48.503	16.879	12.880	1.00	0.00	H
ATOM	1359	CB	PHE	A	106	46.531	16.261	13.459	1.00	0.00	C
ATOM	1360	HB1	PHE	A	106	45.704	15.690	13.041	1.00	0.00	H
ATOM	1361	HB2	PHE	A	106	46.863	15.738	14.349	1.00	0.00	H
ATOM	1362	CG	PHE	A	106	46.023	17.606	13.948	1.00	0.00	C
ATOM	1363	CD1	PHE	A	106	46.853	18.429	14.734	1.00	0.00	C
ATOM	1364	HD1	PHE	A	106	47.873	18.136	14.932	1.00	0.00	H
ATOM	1365	CE1	PHE	A	106	46.345	19.611	15.303	1.00	0.00	C
ATOM	1366	HE1	PHE	A	106	46.979	20.229	15.921	1.00	0.00	H
ATOM	1367	CZ	PHE	A	106	45.003	19.972	15.095	1.00	0.00	C
ATOM	1368	HZ	PHE	A	106	44.602	20.869	15.548	1.00	0.00	H
ATOM	1369	CE2	PHE	A	106	44.172	19.153	14.313	1.00	0.00	C
ATOM	1370	HE2	PHE	A	106	43.139	19.426	14.159	1.00	0.00	H
ATOM	1371	CD2	PHE	A	106	44.677	17.969	13.748	1.00	0.00	C
ATOM	1372	HD2	PHE	A	106	44.017	17.327	13.185	1.00	0.00	H
ATOM	1373	C	PHE	A	106	48.297	14.810	12.399	1.00	0.00	C
ATOM	1374	O	PHE	A	106	49.389	14.591	12.927	1.00	0.00	O
ATOM	1375	N	THR	A	107	47.607	13.878	11.715	1.00	0.00	N
ATOM	1376	H	THR	A	107	46.714	14.148	11.316	1.00	0.00	H
ATOM	1377	CA	THR	A	107	48.038	12.478	11.504	1.00	0.00	C
ATOM	1378	HA	THR	A	107	48.077	11.964	12.465	1.00	0.00	H
ATOM	1379	CB	THR	A	107	47.026	11.744	10.612	1.00	0.00	C
ATOM	1380	HB	THR	A	107	46.932	12.267	9.659	1.00	0.00	H
ATOM	1381	CG2	THR	A	107	47.400	10.289	10.331	1.00	0.00	C
ATOM	1382	HG21	THR	A	107	46.606	9.816	9.756	1.00	0.00	H
ATOM	1383	HG22	THR	A	107	48.319	10.241	9.746	1.00	0.00	H
ATOM	1384	HG23	THR	A	107	47.537	9.746	11.265	1.00	0.00	H
ATOM	1385	OG1	THR	A	107	45.771	11.723	11.254	1.00	0.00	O
ATOM	1386	HG1	THR	A	107	45.863	11.121	12.028	1.00	0.00	H
ATOM	1387	C	THR	A	107	49.442	12.410	10.904	1.00	0.00	C
ATOM	1388	O	THR	A	107	50.285	11.684	11.429	1.00	0.00	O
ATOM	1389	N	ASP	A	108	49.704	13.203	9.862	1.00	0.00	N
ATOM	1390	H	ASP	A	108	48.928	13.725	9.463	1.00	0.00	H
ATOM	1391	CA	ASP	A	108	51.022	13.353	9.228	1.00	0.00	C
ATOM	1392	HA	ASP	A	108	51.380	12.380	8.889	1.00	0.00	H
ATOM	1393	CB	ASP	A	108	50.912	14.308	8.021	1.00	0.00	C
ATOM	1394	HB1	ASP	A	108	50.466	15.246	8.353	1.00	0.00	H
ATOM	1395	HB2	ASP	A	108	51.924	14.530	7.688	1.00	0.00	H
ATOM	1396	CG	ASP	A	108	50.160	13.827	6.778	1.00	0.00	C
ATOM	1397	OD1	ASP	A	108	49.628	12.689	6.745	1.00	0.00	O
ATOM	1398	OD2	ASP	A	108	50.177	14.614	5.794	1.00	0.00	O
ATOM	1399	C	ASP	A	108	52.069	13.936	10.201	1.00	0.00	C
ATOM	1400	O	ASP	A	108	53.204	13.456	10.273	1.00	0.00	O
ATOM	1401	N	MET	A	109	51.691	14.978	10.953	1.00	0.00	N

```
ATOM   1402  H   MET A 109      50.759  15.351  10.804  1.00  0.00           H
ATOM   1403  CA  MET A 109      52.580  15.689  11.880  1.00  0.00           C
ATOM   1404  HA  MET A 109      53.484  15.955  11.339  1.00  0.00           H
ATOM   1405  CB  MET A 109      51.908  16.999  12.323  1.00  0.00           C
ATOM   1406  HB1 MET A 109      51.707  17.586  11.427  1.00  0.00           H
ATOM   1407  HB2 MET A 109      50.958  16.790  12.813  1.00  0.00           H
ATOM   1408  CG  MET A 109      52.789  17.847  13.251  1.00  0.00           C
ATOM   1409  HG1 MET A 109      53.828  17.748  12.933  1.00  0.00           H
ATOM   1410  HG2 MET A 109      52.512  18.894  13.122  1.00  0.00           H
ATOM   1411  SD  MET A 109      52.695  17.455  15.020  1.00  0.00           S
ATOM   1412  CE  MET A 109      51.113  18.234  15.414  1.00  0.00           C
ATOM   1413  HE1 MET A 109      50.959  18.207  16.491  1.00  0.00           H
ATOM   1414  HE2 MET A 109      51.129  19.276  15.093  1.00  0.00           H
ATOM   1415  HE3 MET A 109      50.305  17.700  14.917  1.00  0.00           H
ATOM   1416  C   MET A 109      53.023  14.818  13.056  1.00  0.00           C
ATOM   1417  O   MET A 109      54.189  14.866  13.440  1.00  0.00           O
ATOM   1418  N   VAL A 110      52.146  13.968  13.587  1.00  0.00           N
ATOM   1419  H   VAL A 110      51.185  14.005  13.244  1.00  0.00           H
ATOM   1420  CA  VAL A 110      52.513  13.079  14.703  1.00  0.00           C
ATOM   1421  HA  VAL A 110      53.175  13.646  15.353  1.00  0.00           H
ATOM   1422  CB  VAL A 110      51.311  12.681  15.576  1.00  0.00           C
ATOM   1423  HB  VAL A 110      51.716  12.072  16.379  1.00  0.00           H
ATOM   1424  CG1 VAL A 110      50.680  13.905  16.247  1.00  0.00           C
ATOM   1425 HG11 VAL A 110      49.950  13.579  16.987  1.00  0.00           H
ATOM   1426 HG12 VAL A 110      51.459  14.474  16.756  1.00  0.00           H
ATOM   1427 HG13 VAL A 110      50.190  14.544  15.512  1.00  0.00           H
ATOM   1428  CG2 VAL A 110      50.267  11.852  14.814  1.00  0.00           C
ATOM   1429 HG21 VAL A 110      49.556  11.419  15.512  1.00  0.00           H
ATOM   1430 HG22 VAL A 110      49.728  12.470  14.106  1.00  0.00           H
ATOM   1431 HG23 VAL A 110      50.750  11.047  14.270  1.00  0.00           H
ATOM   1432  C   VAL A 110      53.298  11.825  14.284  1.00  0.00           C
ATOM   1433  O   VAL A 110      53.845  11.145  15.154  1.00  0.00           O
ATOM   1434  N   ALA A 111      53.369  11.502  12.984  1.00  0.00           N
ATOM   1435  H   ALA A 111      52.902  12.106  12.315  1.00  0.00           H
ATOM   1436  CA  ALA A 111      53.898  10.228  12.483  1.00  0.00           C
ATOM   1437  HA  ALA A 111      53.332   9.418  12.946  1.00  0.00           H
ATOM   1438  CB  ALA A 111      53.643  10.178  10.971  1.00  0.00           C
ATOM   1439  HB1 ALA A 111      53.960   9.214  10.574  1.00  0.00           H
ATOM   1440  HB2 ALA A 111      52.578  10.305  10.765  1.00  0.00           H
ATOM   1441  HB3 ALA A 111      54.200  10.967  10.464  1.00  0.00           H
ATOM   1442  C   ALA A 111      55.385   9.970  12.806  1.00  0.00           C
ATOM   1443  O   ALA A 111      55.790   8.809  12.926  1.00  0.00           O
ATOM   1444  N   THR A 112      56.195  11.026  12.972  1.00  0.00           N
ATOM   1445  H   THR A 112      55.776  11.947  12.934  1.00  0.00           H
ATOM   1446  CA  THR A 112      57.610  10.943  13.380  1.00  0.00           C
ATOM   1447  HA  THR A 112      57.776   9.991  13.880  1.00  0.00           H
ATOM   1448  CB  THR A 112      58.591  11.021  12.200  1.00  0.00           C
ATOM   1449  HB  THR A 112      59.609  10.982  12.593  1.00  0.00           H
ATOM   1450  CG2 THR A 112      58.427   9.877  11.206  1.00  0.00           C
ATOM   1451 HG21 THR A 112      59.204   9.945  10.444  1.00  0.00           H
ATOM   1452 HG22 THR A 112      58.529   8.922  11.722  1.00  0.00           H
ATOM   1453 HG23 THR A 112      57.453   9.918  10.719  1.00  0.00           H
ATOM   1454  OG1 THR A 112      58.428  12.229  11.503  1.00  0.00           O
ATOM   1455  HG1 THR A 112      57.619  12.153  10.981  1.00  0.00           H
```

```
ATOM   1456  C   THR A 112      57.941  12.024  14.393  1.00  0.00           C
ATOM   1457  O   THR A 112      57.322  13.086  14.402  1.00  0.00           O
ATOM   1458  N   ALA A 113      58.943  11.770  15.233  1.00  0.00           N
ATOM   1459  H   ALA A 113      59.434  10.887  15.156  1.00  0.00           H
ATOM   1460  CA  ALA A 113      59.392  12.739  16.232  1.00  0.00           C
ATOM   1461  HA  ALA A 113      58.538  13.043  16.840  1.00  0.00           H
ATOM   1462  CB  ALA A 113      60.408  12.054  17.140  1.00  0.00           C
ATOM   1463  HB1 ALA A 113      60.711  12.737  17.935  1.00  0.00           H
ATOM   1464  HB2 ALA A 113      59.941  11.176  17.577  1.00  0.00           H
ATOM   1465  HB3 ALA A 113      61.290  11.753  16.575  1.00  0.00           H
ATOM   1466  C   ALA A 113      59.958  14.016  15.600  1.00  0.00           C
ATOM   1467  O   ALA A 113      59.771  15.100  16.149  1.00  0.00           O
ATOM   1468  N   ASN A 114      60.599  13.925  14.432  1.00  0.00           N
ATOM   1469  H   ASN A 114      60.805  13.003  14.066  1.00  0.00           H
ATOM   1470  CA  ASN A 114      61.026  15.098  13.671  1.00  0.00           C
ATOM   1471  HA  ASN A 114      61.628  15.735  14.320  1.00  0.00           H
ATOM   1472  CB  ASN A 114      61.914  14.660  12.503  1.00  0.00           C
ATOM   1473  HB1 ASN A 114      62.846  14.247  12.888  1.00  0.00           H
ATOM   1474  HB2 ASN A 114      61.399  13.884  11.946  1.00  0.00           H
ATOM   1475  CG  ASN A 114      62.237  15.825  11.575  1.00  0.00           C
ATOM   1476  OD1 ASN A 114      62.964  16.747  11.925  1.00  0.00           O
ATOM   1477  ND2 ASN A 114      61.690  15.841  10.377  1.00  0.00           N
ATOM   1478 HD21 ASN A 114      61.123  15.065  10.061  1.00  0.00           H
ATOM   1479 HD22 ASN A 114      61.875  16.642   9.786  1.00  0.00           H
ATOM   1480  C   ASN A 114      59.818  15.923  13.196  1.00  0.00           C
ATOM   1481  O   ASN A 114      59.817  17.136  13.369  1.00  0.00           O
ATOM   1482  N   ASN A 115      58.749  15.296  12.690  1.00  0.00           N
ATOM   1483  H   ASN A 115      58.762  14.289  12.588  1.00  0.00           H
ATOM   1484  CA  ASN A 115      57.531  16.026  12.312  1.00  0.00           C
ATOM   1485  HA  ASN A 115      57.807  16.816  11.611  1.00  0.00           H
ATOM   1486  CB  ASN A 115      56.551  15.088  11.595  1.00  0.00           C
ATOM   1487  HB1 ASN A 115      56.327  14.237  12.235  1.00  0.00           H
ATOM   1488  HB2 ASN A 115      55.630  15.639  11.420  1.00  0.00           H
ATOM   1489  CG  ASN A 115      57.012  14.563  10.242  1.00  0.00           C
ATOM   1490  OD1 ASN A 115      58.074  14.892   9.726  1.00  0.00           O
ATOM   1491  ND2 ASN A 115      56.210  13.716   9.623  1.00  0.00           N
ATOM   1492 HD21 ASN A 115      55.276  13.540   9.978  1.00  0.00           H
ATOM   1493 HD22 ASN A 115      56.446  13.477   8.665  1.00  0.00           H
ATOM   1494  C   ASN A 115      56.874  16.712  13.534  1.00  0.00           C
ATOM   1495  O   ASN A 115      56.463  17.871  13.436  1.00  0.00           O
ATOM   1496  N   ARG A 116      56.886  16.068  14.713  1.00  0.00           N
ATOM   1497  H   ARG A 116      57.160  15.091  14.719  1.00  0.00           H
ATOM   1498  CA  ARG A 116      56.467  16.696  15.979  1.00  0.00           C
ATOM   1499  HA  ARG A 116      55.483  17.139  15.821  1.00  0.00           H
ATOM   1500  CB  ARG A 116      56.334  15.653  17.101  1.00  0.00           C
ATOM   1501  HB1 ARG A 116      57.279  15.126  17.232  1.00  0.00           H
ATOM   1502  HB2 ARG A 116      56.100  16.177  18.030  1.00  0.00           H
ATOM   1503  CG  ARG A 116      55.211  14.645  16.815  1.00  0.00           C
ATOM   1504  HG1 ARG A 116      54.317  15.195  16.518  1.00  0.00           H
ATOM   1505  HG2 ARG A 116      55.501  13.993  15.993  1.00  0.00           H
ATOM   1506  CD  ARG A 116      54.844  13.785  18.030  1.00  0.00           C
ATOM   1507  HD1 ARG A 116      54.445  14.438  18.808  1.00  0.00           H
ATOM   1508  HD2 ARG A 116      54.052  13.090  17.747  1.00  0.00           H
ATOM   1509  NE  ARG A 116      55.992  13.048  18.586  1.00  0.00           N
```

ATOM	1510	HE	ARG	A	116	56.431	13.464	19.408	1.00	0.00	H
ATOM	1511	CZ	ARG	A	116	56.398	11.824	18.277	1.00	0.00	C
ATOM	1512	NH1	ARG	A	116	55.881	11.135	17.282	1.00	0.00	N
ATOM	1513	HH11	ARG	A	116	55.165	11.528	16.689	1.00	0.00	H
ATOM	1514	HH12	ARG	A	116	56.201	10.190	17.097	1.00	0.00	H
ATOM	1515	NH2	ARG	A	116	57.341	11.264	19.001	1.00	0.00	N
ATOM	1516	HH21	ARG	A	116	57.765	11.775	19.775	1.00	0.00	H
ATOM	1517	HH22	ARG	A	116	57.571	10.286	18.878	1.00	0.00	H
ATOM	1518	C	ARG	A	116	57.402	17.837	16.410	1.00	0.00	C
ATOM	1519	O	ARG	A	116	56.922	18.889	16.826	1.00	0.00	O
ATOM	1520	N	GLN	A	117	58.721	17.686	16.277	1.00	0.00	N
ATOM	1521	H	GLN	A	117	59.069	16.775	15.997	1.00	0.00	H
ATOM	1522	CA	GLN	A	117	59.702	18.746	16.539	1.00	0.00	C
ATOM	1523	HA	GLN	A	117	59.595	19.087	17.570	1.00	0.00	H
ATOM	1524	CB	GLN	A	117	61.120	18.178	16.369	1.00	0.00	C
ATOM	1525	HB1	GLN	A	117	61.258	17.374	17.091	1.00	0.00	H
ATOM	1526	HB2	GLN	A	117	61.218	17.765	15.368	1.00	0.00	H
ATOM	1527	CG	GLN	A	117	62.235	19.216	16.586	1.00	0.00	C
ATOM	1528	HG1	GLN	A	117	62.063	20.079	15.944	1.00	0.00	H
ATOM	1529	HG2	GLN	A	117	62.223	19.540	17.626	1.00	0.00	H
ATOM	1530	CD	GLN	A	117	63.619	18.666	16.244	1.00	0.00	C
ATOM	1531	OE1	GLN	A	117	64.525	18.625	17.064	1.00	0.00	O
ATOM	1532	NE2	GLN	A	117	63.845	18.264	15.013	1.00	0.00	N
ATOM	1533	HE21	GLN	A	117	63.136	18.384	14.302	1.00	0.00	H
ATOM	1534	HE22	GLN	A	117	64.737	17.838	14.794	1.00	0.00	H
ATOM	1535	C	GLN	A	117	59.455	19.963	15.638	1.00	0.00	C
ATOM	1536	O	GLN	A	117	59.431	21.075	16.154	1.00	0.00	O
ATOM	1537	N	THR	A	118	59.201	19.764	14.336	1.00	0.00	N
ATOM	1538	H	THR	A	118	59.301	18.823	13.968	1.00	0.00	H
ATOM	1539	CA	THR	A	118	58.831	20.817	13.374	1.00	0.00	C
ATOM	1540	HA	THR	A	118	59.663	21.514	13.270	1.00	0.00	H
ATOM	1541	CB	THR	A	118	58.546	20.180	12.007	1.00	0.00	C
ATOM	1542	HB	THR	A	118	57.700	19.498	12.084	1.00	0.00	H
ATOM	1543	CG2	THR	A	118	58.239	21.218	10.938	1.00	0.00	C
ATOM	1544	HG21	THR	A	118	58.174	20.731	9.967	1.00	0.00	H
ATOM	1545	HG22	THR	A	118	57.285	21.693	11.156	1.00	0.00	H
ATOM	1546	HG23	THR	A	118	59.024	21.971	10.916	1.00	0.00	H
ATOM	1547	OG1	THR	A	118	59.677	19.463	11.573	1.00	0.00	O
ATOM	1548	HG1	THR	A	118	59.451	19.025	10.735	1.00	0.00	H
ATOM	1549	C	THR	A	118	57.631	21.624	13.866	1.00	0.00	C
ATOM	1550	O	THR	A	118	57.644	22.856	13.808	1.00	0.00	O
ATOM	1551	N	PHE	A	119	56.609	20.950	14.403	1.00	0.00	N
ATOM	1552	H	PHE	A	119	56.629	19.939	14.381	1.00	0.00	H
ATOM	1553	CA	PHE	A	119	55.474	21.614	15.038	1.00	0.00	C
ATOM	1554	HA	PHE	A	119	55.100	22.378	14.355	1.00	0.00	H
ATOM	1555	CB	PHE	A	119	54.345	20.603	15.277	1.00	0.00	C
ATOM	1556	HB1	PHE	A	119	54.058	20.169	14.321	1.00	0.00	H
ATOM	1557	HB2	PHE	A	119	54.696	19.790	15.910	1.00	0.00	H
ATOM	1558	CG	PHE	A	119	53.121	21.237	15.904	1.00	0.00	C
ATOM	1559	CD1	PHE	A	119	52.425	22.238	15.204	1.00	0.00	C
ATOM	1560	HD1	PHE	A	119	52.740	22.531	14.214	1.00	0.00	H
ATOM	1561	CE1	PHE	A	119	51.333	22.885	15.797	1.00	0.00	C
ATOM	1562	HE1	PHE	A	119	50.826	23.665	15.252	1.00	0.00	H
ATOM	1563	CZ	PHE	A	119	50.917	22.526	17.091	1.00	0.00	C

ATOM	1564	HZ	PHE	A	119	50.078	23.030	17.550	1.00	0.00	H
ATOM	1565	CE2	PHE	A	119	51.601	21.516	17.791	1.00	0.00	C
ATOM	1566	HE2	PHE	A	119	51.286	21.243	18.788	1.00	0.00	H
ATOM	1567	CD2	PHE	A	119	52.707	20.876	17.202	1.00	0.00	C
ATOM	1568	HD2	PHE	A	119	53.243	20.116	17.751	1.00	0.00	H
ATOM	1569	C	PHE	A	119	55.875	22.334	16.332	1.00	0.00	C
ATOM	1570	O	PHE	A	119	55.706	23.545	16.427	1.00	0.00	O
ATOM	1571	N	VAL	A	120	56.431	21.612	17.306	1.00	0.00	N
ATOM	1572	H	VAL	A	120	56.593	20.626	17.107	1.00	0.00	H
ATOM	1573	CA	VAL	A	120	56.751	22.120	18.655	1.00	0.00	C
ATOM	1574	HA	VAL	A	120	55.816	22.433	19.123	1.00	0.00	H
ATOM	1575	CB	VAL	A	120	57.368	20.992	19.520	1.00	0.00	C
ATOM	1576	HB	VAL	A	120	58.185	20.533	18.958	1.00	0.00	H
ATOM	1577	CG1	VAL	A	120	57.930	21.490	20.860	1.00	0.00	C
ATOM	1578	HG11	VAL	A	120	58.280	20.637	21.441	1.00	0.00	H
ATOM	1579	HG12	VAL	A	120	58.775	22.158	20.697	1.00	0.00	H
ATOM	1580	HG13	VAL	A	120	57.155	22.014	21.421	1.00	0.00	H
ATOM	1581	CG2	VAL	A	120	56.338	19.890	19.840	1.00	0.00	C
ATOM	1582	HG21	VAL	A	120	56.844	19.022	20.264	1.00	0.00	H
ATOM	1583	HG22	VAL	A	120	55.614	20.255	20.563	1.00	0.00	H
ATOM	1584	HG23	VAL	A	120	55.798	19.579	18.949	1.00	0.00	H
ATOM	1585	C	VAL	A	120	57.687	23.339	18.596	1.00	0.00	C
ATOM	1586	O	VAL	A	120	57.400	24.373	19.200	1.00	0.00	O
ATOM	1587	N	ASN	A	121	58.784	23.247	17.838	1.00	0.00	N
ATOM	1588	H	ASN	A	121	58.958	22.382	17.335	1.00	0.00	H
ATOM	1589	CA	ASN	A	121	59.810	24.288	17.767	1.00	0.00	C
ATOM	1590	HA	ASN	A	121	60.081	24.579	18.783	1.00	0.00	H
ATOM	1591	CB	ASN	A	121	61.069	23.728	17.078	1.00	0.00	C
ATOM	1592	HB1	ASN	A	121	60.805	23.301	16.110	1.00	0.00	H
ATOM	1593	HB2	ASN	A	121	61.754	24.554	16.891	1.00	0.00	H
ATOM	1594	CG	ASN	A	121	61.823	22.681	17.902	1.00	0.00	C
ATOM	1595	OD1	ASN	A	121	61.394	22.217	18.956	1.00	0.00	O
ATOM	1596	ND2	ASN	A	121	63.001	22.289	17.452	1.00	0.00	N
ATOM	1597	HD21	ASN	A	121	63.360	22.649	16.578	1.00	0.00	H
ATOM	1598	HD22	ASN	A	121	63.548	21.662	18.021	1.00	0.00	H
ATOM	1599	C	ASN	A	121	59.306	25.551	17.046	1.00	0.00	C
ATOM	1600	O	ASN	A	121	59.649	26.664	17.449	1.00	0.00	O
ATOM	1601	N	SER	A	122	58.456	25.413	16.022	1.00	0.00	N
ATOM	1602	H	SER	A	122	58.171	24.480	15.752	1.00	0.00	H
ATOM	1603	CA	SER	A	122	57.815	26.572	15.383	1.00	0.00	C
ATOM	1604	HA	SER	A	122	58.560	27.360	15.256	1.00	0.00	H
ATOM	1605	CB	SER	A	122	57.303	26.227	13.974	1.00	0.00	C
ATOM	1606	HB1	SER	A	122	56.848	27.120	13.543	1.00	0.00	H
ATOM	1607	HB2	SER	A	122	58.150	25.942	13.346	1.00	0.00	H
ATOM	1608	OG	SER	A	122	56.348	25.180	13.965	1.00	0.00	O
ATOM	1609	HG	SER	A	122	56.831	24.325	13.968	1.00	0.00	H
ATOM	1610	C	SER	A	122	56.702	27.176	16.258	1.00	0.00	C
ATOM	1611	O	SER	A	122	56.615	28.401	16.359	1.00	0.00	O
ATOM	1612	N	ALA	A	123	55.896	26.357	16.946	1.00	0.00	N
ATOM	1613	H	ALA	A	123	55.982	25.355	16.795	1.00	0.00	H
ATOM	1614	CA	ALA	A	123	54.816	26.812	17.824	1.00	0.00	C
ATOM	1615	HA	ALA	A	123	54.158	27.463	17.250	1.00	0.00	H
ATOM	1616	CB	ALA	A	123	54.013	25.587	18.284	1.00	0.00	C
ATOM	1617	HB1	ALA	A	123	53.194	25.906	18.929	1.00	0.00	H

```
ATOM   1618  HB2 ALA A 123      53.601  25.063  17.421  1.00  0.00           H
ATOM   1619  HB3 ALA A 123      54.653  24.902  18.844  1.00  0.00           H
ATOM   1620  C   ALA A 123      55.322  27.641  19.016  1.00  0.00           C
ATOM   1621  O   ALA A 123      54.777  28.710  19.287  1.00  0.00           O
ATOM   1622  N   ILE A 124      56.392  27.200  19.688  1.00  0.00           N
ATOM   1623  H   ILE A 124      56.748  26.281  19.445  1.00  0.00           H
ATOM   1624  CA  ILE A 124      56.985  27.920  20.833  1.00  0.00           C
ATOM   1625  HA  ILE A 124      56.213  28.080  21.589  1.00  0.00           H
ATOM   1626  CB  ILE A 124      58.111  27.063  21.464  1.00  0.00           C
ATOM   1627  HB  ILE A 124      58.765  26.708  20.666  1.00  0.00           H
ATOM   1628  CG2 ILE A 124      58.965  27.879  22.451  1.00  0.00           C
ATOM   1629 HG21 ILE A 124      59.749  27.252  22.873  1.00  0.00           H
ATOM   1630 HG22 ILE A 124      59.460  28.706  21.942  1.00  0.00           H
ATOM   1631 HG23 ILE A 124      58.342  28.271  23.256  1.00  0.00           H
ATOM   1632  CG1 ILE A 124      57.502  25.837  22.187  1.00  0.00           C
ATOM   1633 HG11 ILE A 124      56.956  26.170  23.072  1.00  0.00           H
ATOM   1634 HG12 ILE A 124      56.786  25.344  21.531  1.00  0.00           H
ATOM   1635  CD  ILE A 124      58.536  24.784  22.607  1.00  0.00           C
ATOM   1636  HD1 ILE A 124      58.016  23.892  22.954  1.00  0.00           H
ATOM   1637  HD2 ILE A 124      59.163  24.516  21.755  1.00  0.00           H
ATOM   1638  HD3 ILE A 124      59.160  25.158  23.418  1.00  0.00           H
ATOM   1639  C   ILE A 124      57.448  29.324  20.416  1.00  0.00           C
ATOM   1640  O   ILE A 124      57.083  30.309  21.067  1.00  0.00           O
ATOM   1641  N   ARG A 125      58.170  29.433  19.289  1.00  0.00           N
ATOM   1642  H   ARG A 125      58.416  28.586  18.797  1.00  0.00           H
ATOM   1643  CA  ARG A 125      58.565  30.724  18.709  1.00  0.00           C
ATOM   1644  HA  ARG A 125      59.216  31.234  19.420  1.00  0.00           H
ATOM   1645  CB  ARG A 125      59.334  30.526  17.390  1.00  0.00           C
ATOM   1646  HB1 ARG A 125      58.859  29.752  16.787  1.00  0.00           H
ATOM   1647  HB2 ARG A 125      59.299  31.462  16.829  1.00  0.00           H
ATOM   1648  CG  ARG A 125      60.808  30.176  17.622  1.00  0.00           C
ATOM   1649  HG1 ARG A 125      61.246  30.927  18.280  1.00  0.00           H
ATOM   1650  HG2 ARG A 125      60.892  29.203  18.103  1.00  0.00           H
ATOM   1651  CD  ARG A 125      61.601  30.181  16.308  1.00  0.00           C
ATOM   1652  HD1 ARG A 125      61.350  31.078  15.739  1.00  0.00           H
ATOM   1653  HD2 ARG A 125      62.663  30.239  16.545  1.00  0.00           H
ATOM   1654  NE  ARG A 125      61.353  28.994  15.473  1.00  0.00           N
ATOM   1655  HE  ARG A 125      60.702  29.096  14.702  1.00  0.00           H
ATOM   1656  CZ  ARG A 125      62.062  27.872  15.506  1.00  0.00           C
ATOM   1657  NH1 ARG A 125      61.883  26.971  14.569  1.00  0.00           N
ATOM   1658 HH11 ARG A 125      61.239  27.171  13.809  1.00  0.00           H
ATOM   1659 HH12 ARG A 125      62.518  26.188  14.515  1.00  0.00           H
ATOM   1660  NH2 ARG A 125      62.969  27.622  16.427  1.00  0.00           N
ATOM   1661 HH21 ARG A 125      63.165  28.292  17.164  1.00  0.00           H
ATOM   1662 HH22 ARG A 125      63.533  26.790  16.352  1.00  0.00           H
ATOM   1663  C   ARG A 125      57.356  31.633  18.465  1.00  0.00           C
ATOM   1664  O   ARG A 125      57.409  32.805  18.845  1.00  0.00           O
ATOM   1665  N   PHE A 126      56.288  31.085  17.874  1.00  0.00           N
ATOM   1666  H   PHE A 126      56.348  30.103  17.634  1.00  0.00           H
ATOM   1667  CA  PHE A 126      55.053  31.780  17.499  1.00  0.00           C
ATOM   1668  HA  PHE A 126      55.302  32.604  16.828  1.00  0.00           H
ATOM   1669  CB  PHE A 126      54.170  30.770  16.742  1.00  0.00           C
ATOM   1670  HB1 PHE A 126      54.810  30.131  16.138  1.00  0.00           H
ATOM   1671  HB2 PHE A 126      53.683  30.121  17.467  1.00  0.00           H
```

ATOM	1672	CG	PHE	A	126	53.111	31.314	15.800	1.00	0.00	C
ATOM	1673	CD1	PHE	A	126	51.805	31.571	16.258	1.00	0.00	C
ATOM	1674	HD1	PHE	A	126	51.564	31.473	17.307	1.00	0.00	H
ATOM	1675	CE1	PHE	A	126	50.797	31.933	15.346	1.00	0.00	C
ATOM	1676	HE1	PHE	A	126	49.794	32.121	15.702	1.00	0.00	H
ATOM	1677	CZ	PHE	A	126	51.086	32.038	13.974	1.00	0.00	C
ATOM	1678	HZ	PHE	A	126	50.305	32.311	13.280	1.00	0.00	H
ATOM	1679	CE2	PHE	A	126	52.389	31.787	13.513	1.00	0.00	C
ATOM	1680	HE2	PHE	A	126	52.618	31.868	12.460	1.00	0.00	H
ATOM	1681	CD2	PHE	A	126	53.398	31.429	14.425	1.00	0.00	C
ATOM	1682	HD2	PHE	A	126	54.395	31.229	14.063	1.00	0.00	H
ATOM	1683	C	PHE	A	126	54.336	32.358	18.729	1.00	0.00	C
ATOM	1684	O	PHE	A	126	54.043	33.553	18.759	1.00	0.00	O
ATOM	1685	N	LEU	A	127	54.105	31.549	19.775	1.00	0.00	N
ATOM	1686	H	LEU	A	127	54.391	30.575	19.710	1.00	0.00	H
ATOM	1687	CA	LEU	A	127	53.421	32.014	20.989	1.00	0.00	C
ATOM	1688	HA	LEU	A	127	52.468	32.460	20.698	1.00	0.00	H
ATOM	1689	CB	LEU	A	127	53.154	30.851	21.965	1.00	0.00	C
ATOM	1690	HB1	LEU	A	127	54.109	30.411	22.260	1.00	0.00	H
ATOM	1691	HB2	LEU	A	127	52.706	31.279	22.864	1.00	0.00	H
ATOM	1692	CG	LEU	A	127	52.236	29.715	21.464	1.00	0.00	C
ATOM	1693	HG	LEU	A	127	52.797	29.094	20.771	1.00	0.00	H
ATOM	1694	CD1	LEU	A	127	51.838	28.848	22.666	1.00	0.00	C
ATOM	1695	HD11	LEU	A	127	51.250	27.998	22.326	1.00	0.00	H
ATOM	1696	HD12	LEU	A	127	52.727	28.486	23.180	1.00	0.00	H
ATOM	1697	HD13	LEU	A	127	51.246	29.435	23.368	1.00	0.00	H
ATOM	1698	CD2	LEU	A	127	50.960	30.176	20.744	1.00	0.00	C
ATOM	1699	HD21	LEU	A	127	50.389	29.301	20.434	1.00	0.00	H
ATOM	1700	HD22	LEU	A	127	50.342	30.777	21.408	1.00	0.00	H
ATOM	1701	HD23	LEU	A	127	51.210	30.751	19.853	1.00	0.00	H
ATOM	1702	C	LEU	A	127	54.212	33.127	21.685	1.00	0.00	C
ATOM	1703	O	LEU	A	127	53.640	34.175	21.994	1.00	0.00	O
ATOM	1704	N	ARG	A	128	55.524	32.945	21.885	1.00	0.00	N
ATOM	1705	H	ARG	A	128	55.948	32.067	21.596	1.00	0.00	H
ATOM	1706	CA	ARG	A	128	56.368	33.975	22.505	1.00	0.00	C
ATOM	1707	HA	ARG	A	128	55.914	34.250	23.456	1.00	0.00	H
ATOM	1708	CB	ARG	A	128	57.779	33.428	22.776	1.00	0.00	C
ATOM	1709	HB1	ARG	A	128	58.188	33.004	21.857	1.00	0.00	H
ATOM	1710	HB2	ARG	A	128	58.423	34.258	23.071	1.00	0.00	H
ATOM	1711	CG	ARG	A	128	57.838	32.364	23.887	1.00	0.00	C
ATOM	1712	HG1	ARG	A	128	57.316	31.464	23.562	1.00	0.00	H
ATOM	1713	HG2	ARG	A	128	58.885	32.108	24.042	1.00	0.00	H
ATOM	1714	CD	ARG	A	128	57.232	32.840	25.217	1.00	0.00	C
ATOM	1715	HD1	ARG	A	128	57.546	33.865	25.411	1.00	0.00	H
ATOM	1716	HD2	ARG	A	128	56.145	32.813	25.137	1.00	0.00	H
ATOM	1717	NE	ARG	A	128	57.666	32.001	26.340	1.00	0.00	N
ATOM	1718	HE	ARG	A	128	58.507	31.437	26.195	1.00	0.00	H
ATOM	1719	CZ	ARG	A	128	57.137	31.945	27.554	1.00	0.00	C
ATOM	1720	NH1	ARG	A	128	56.094	32.650	27.936	1.00	0.00	N
ATOM	1721	HH11	ARG	A	128	55.623	33.275	27.292	1.00	0.00	H
ATOM	1722	HH12	ARG	A	128	55.752	32.530	28.881	1.00	0.00	H
ATOM	1723	NH2	ARG	A	128	57.674	31.146	28.435	1.00	0.00	N
ATOM	1724	HH21	ARG	A	128	58.424	30.516	28.137	1.00	0.00	H
ATOM	1725	HH22	ARG	A	128	57.263	31.063	29.357	1.00	0.00	H

ATOM	1726	C	ARG	A	128	56.415	35.263	21.671	1.00	0.00	C
ATOM	1727	O	ARG	A	128	56.268	36.347	22.244	1.00	0.00	O
ATOM	1728	N	LYS	A	129	56.526	35.160	20.341	1.00	0.00	N
ATOM	1729	H	LYS	A	129	56.625	34.235	19.940	1.00	0.00	H
ATOM	1730	CA	LYS	A	129	56.465	36.293	19.405	1.00	0.00	C
ATOM	1731	HA	LYS	A	129	57.296	36.969	19.613	1.00	0.00	H
ATOM	1732	CB	LYS	A	129	56.633	35.758	17.968	1.00	0.00	C
ATOM	1733	HB1	LYS	A	129	57.638	35.346	17.863	1.00	0.00	H
ATOM	1734	HB2	LYS	A	129	55.918	34.955	17.796	1.00	0.00	H
ATOM	1735	CG	LYS	A	129	56.423	36.827	16.884	1.00	0.00	C
ATOM	1736	HG1	LYS	A	129	55.427	37.261	16.980	1.00	0.00	H
ATOM	1737	HG2	LYS	A	129	57.166	37.614	17.003	1.00	0.00	H
ATOM	1738	CD	LYS	A	129	56.544	36.217	15.485	1.00	0.00	C
ATOM	1739	HD1	LYS	A	129	57.568	35.875	15.323	1.00	0.00	H
ATOM	1740	HD2	LYS	A	129	55.865	35.365	15.407	1.00	0.00	H
ATOM	1741	CE	LYS	A	129	56.162	37.261	14.432	1.00	0.00	C
ATOM	1742	HE1	LYS	A	129	55.145	37.607	14.638	1.00	0.00	H
ATOM	1743	HE2	LYS	A	129	56.840	38.117	14.514	1.00	0.00	H
ATOM	1744	NZ	LYS	A	129	56.229	36.688	13.070	1.00	0.00	N
ATOM	1745	HZ1	LYS	A	129	57.192	36.464	12.835	1.00	0.00	H
ATOM	1746	HZ2	LYS	A	129	55.692	35.825	13.017	1.00	0.00	H
ATOM	1747	HZ3	LYS	A	129	55.874	37.335	12.375	1.00	0.00	H
ATOM	1748	C	LYS	A	129	55.172	37.109	19.578	1.00	0.00	C
ATOM	1749	O	LYS	A	129	55.233	38.294	19.897	1.00	0.00	O
ATOM	1750	N	TYR	A	130	53.997	36.497	19.407	1.00	0.00	N
ATOM	1751	H	TYR	A	130	53.987	35.517	19.139	1.00	0.00	H
ATOM	1752	CA	TYR	A	130	52.729	37.240	19.417	1.00	0.00	C
ATOM	1753	HA	TYR	A	130	52.896	38.190	18.909	1.00	0.00	H
ATOM	1754	CB	TYR	A	130	51.684	36.479	18.593	1.00	0.00	C
ATOM	1755	HB1	TYR	A	130	51.575	35.475	19.004	1.00	0.00	H
ATOM	1756	HB2	TYR	A	130	50.724	36.983	18.689	1.00	0.00	H
ATOM	1757	CG	TYR	A	130	52.031	36.396	17.116	1.00	0.00	C
ATOM	1758	CD1	TYR	A	130	52.052	37.560	16.318	1.00	0.00	C
ATOM	1759	HD1	TYR	A	130	51.791	38.516	16.750	1.00	0.00	H
ATOM	1760	CE1	TYR	A	130	52.410	37.484	14.956	1.00	0.00	C
ATOM	1761	HE1	TYR	A	130	52.416	38.376	14.345	1.00	0.00	H
ATOM	1762	CZ	TYR	A	130	52.768	36.238	14.394	1.00	0.00	C
ATOM	1763	OH	TYR	A	130	53.140	36.141	13.090	1.00	0.00	O
ATOM	1764	HH	TYR	A	130	53.050	36.987	12.604	1.00	0.00	H
ATOM	1765	CE2	TYR	A	130	52.752	35.079	15.192	1.00	0.00	C
ATOM	1766	HE2	TYR	A	130	53.025	34.129	14.763	1.00	0.00	H
ATOM	1767	CD2	TYR	A	130	52.367	35.158	16.540	1.00	0.00	C
ATOM	1768	HD2	TYR	A	130	52.321	34.258	17.135	1.00	0.00	H
ATOM	1769	C	TYR	A	130	52.221	37.611	20.826	1.00	0.00	C
ATOM	1770	O	TYR	A	130	51.438	38.550	20.942	1.00	0.00	O
ATOM	1771	N	SER	A	131	52.701	36.926	21.873	1.00	0.00	N
ATOM	1772	H	SER	A	131	53.280	36.125	21.644	1.00	0.00	H
ATOM	1773	CA	SER	A	131	52.508	37.195	23.318	1.00	0.00	C
ATOM	1774	HA	SER	A	131	53.422	36.890	23.823	1.00	0.00	H
ATOM	1775	CB	SER	A	131	52.328	38.682	23.659	1.00	0.00	C
ATOM	1776	HB1	SER	A	131	53.009	39.271	23.048	1.00	0.00	H
ATOM	1777	HB2	SER	A	131	51.306	38.983	23.447	1.00	0.00	H
ATOM	1778	OG	SER	A	131	52.610	38.920	25.025	1.00	0.00	O
ATOM	1779	HG	SER	A	131	52.707	39.884	25.157	1.00	0.00	H

```
ATOM   1780  C    SER A 131      51.397  36.326  23.931  1.00  0.00           C
ATOM   1781  O    SER A 131      50.563  36.786  24.715  1.00  0.00           O
ATOM   1782  N    PHE A 132      51.345  35.052  23.548  1.00  0.00           N
ATOM   1783  H    PHE A 132      52.053  34.712  22.909  1.00  0.00           H
ATOM   1784  CA   PHE A 132      50.432  34.068  24.121  1.00  0.00           C
ATOM   1785  HA   PHE A 132      49.522  34.569  24.457  1.00  0.00           H
ATOM   1786  CB   PHE A 132      50.027  33.066  23.034  1.00  0.00           C
ATOM   1787  HB1  PHE A 132      50.905  32.789  22.454  1.00  0.00           H
ATOM   1788  HB2  PHE A 132      49.644  32.160  23.507  1.00  0.00           H
ATOM   1789  CG   PHE A 132      48.959  33.608  22.104  1.00  0.00           C
ATOM   1790  CD1  PHE A 132      47.612  33.280  22.333  1.00  0.00           C
ATOM   1791  HD1  PHE A 132      47.361  32.631  23.150  1.00  0.00           H
ATOM   1792  CE1  PHE A 132      46.597  33.803  21.516  1.00  0.00           C
ATOM   1793  HE1  PHE A 132      45.563  33.551  21.702  1.00  0.00           H
ATOM   1794  CZ   PHE A 132      46.928  34.675  20.468  1.00  0.00           C
ATOM   1795  HZ   PHE A 132      46.147  35.103  19.857  1.00  0.00           H
ATOM   1796  CE2  PHE A 132      48.273  35.017  20.242  1.00  0.00           C
ATOM   1797  HE2  PHE A 132      48.522  35.729  19.467  1.00  0.00           H
ATOM   1798  CD2  PHE A 132      49.289  34.481  21.053  1.00  0.00           C
ATOM   1799  HD2  PHE A 132      50.317  34.763  20.883  1.00  0.00           H
ATOM   1800  C    PHE A 132      51.044  33.387  25.353  1.00  0.00           C
ATOM   1801  O    PHE A 132      52.264  33.298  25.510  1.00  0.00           O
ATOM   1802  N    ASP A 133      50.162  32.907  26.224  1.00  0.00           N
ATOM   1803  H    ASP A 133      49.177  33.066  26.022  1.00  0.00           H
ATOM   1804  CA   ASP A 133      50.452  32.201  27.471  1.00  0.00           C
ATOM   1805  HA   ASP A 133      51.479  32.405  27.781  1.00  0.00           H
ATOM   1806  CB   ASP A 133      49.501  32.732  28.555  1.00  0.00           C
ATOM   1807  HB1  ASP A 133      48.467  32.541  28.262  1.00  0.00           H
ATOM   1808  HB2  ASP A 133      49.687  32.186  29.478  1.00  0.00           H
ATOM   1809  CG   ASP A 133      49.694  34.222  28.834  1.00  0.00           C
ATOM   1810  OD1  ASP A 133      50.723  34.581  29.457  1.00  0.00           O
ATOM   1811  OD2  ASP A 133      48.810  35.025  28.458  1.00  0.00           O
ATOM   1812  C    ASP A 133      50.297  30.673  27.346  1.00  0.00           C
ATOM   1813  O    ASP A 133      50.639  29.955  28.283  1.00  0.00           O
ATOM   1814  N    GLY A 134      49.790  30.152  26.218  1.00  0.00           N
ATOM   1815  H    GLY A 134      49.499  30.785  25.487  1.00  0.00           H
ATOM   1816  CA   GLY A 134      49.625  28.708  26.005  1.00  0.00           C
ATOM   1817  HA1  GLY A 134      50.608  28.237  26.029  1.00  0.00           H
ATOM   1818  HA2  GLY A 134      49.069  28.287  26.843  1.00  0.00           H
ATOM   1819  C    GLY A 134      48.929  28.288  24.709  1.00  0.00           C
ATOM   1820  O    GLY A 134      48.531  29.125  23.896  1.00  0.00           O
ATOM   1821  N    LEU A 135      48.796  26.971  24.531  1.00  0.00           N
ATOM   1822  H    LEU A 135      49.121  26.370  25.284  1.00  0.00           H
ATOM   1823  CA   LEU A 135      48.211  26.301  23.362  1.00  0.00           C
ATOM   1824  HA   LEU A 135      47.868  27.046  22.645  1.00  0.00           H
ATOM   1825  CB   LEU A 135      49.314  25.425  22.724  1.00  0.00           C
ATOM   1826  HB1  LEU A 135      50.233  26.002  22.637  1.00  0.00           H
ATOM   1827  HB2  LEU A 135      49.518  24.599  23.409  1.00  0.00           H
ATOM   1828  CG   LEU A 135      48.969  24.846  21.335  1.00  0.00           C
ATOM   1829  HG   LEU A 135      47.961  24.430  21.351  1.00  0.00           H
ATOM   1830  CD1  LEU A 135      49.055  25.916  20.235  1.00  0.00           C
ATOM   1831 HD11  LEU A 135      48.764  25.483  19.277  1.00  0.00           H
ATOM   1832 HD12  LEU A 135      48.388  26.745  20.457  1.00  0.00           H
ATOM   1833 HD13  LEU A 135      50.074  26.298  20.159  1.00  0.00           H
```

```
ATOM   1834  CD2 LEU A 135      49.945  23.711  20.993  1.00  0.00           C
ATOM   1835 HD21 LEU A 135      49.680  23.274  20.030  1.00  0.00           H
ATOM   1836 HD22 LEU A 135      50.966  24.091  20.944  1.00  0.00           H
ATOM   1837 HD23 LEU A 135      49.889  22.933  21.754  1.00  0.00           H
ATOM   1838  C   LEU A 135      47.014  25.431  23.784  1.00  0.00           C
ATOM   1839  O   LEU A 135      47.108  24.712  24.777  1.00  0.00           O
ATOM   1840  N   ASP A 136      45.929  25.447  23.005  1.00  0.00           N
ATOM   1841  H   ASP A 136      45.925  26.074  22.206  1.00  0.00           H
ATOM   1842  CA  ASP A 136      44.780  24.541  23.147  1.00  0.00           C
ATOM   1843  HA  ASP A 136      44.930  23.914  24.024  1.00  0.00           H
ATOM   1844  CB  ASP A 136      43.493  25.352  23.383  1.00  0.00           C
ATOM   1845  HB1 ASP A 136      43.657  26.041  24.213  1.00  0.00           H
ATOM   1846  HB2 ASP A 136      43.268  25.949  22.500  1.00  0.00           H
ATOM   1847  CG  ASP A 136      42.296  24.456  23.724  1.00  0.00           C
ATOM   1848  OD1 ASP A 136      42.326  23.829  24.810  1.00  0.00           O
ATOM   1849  OD2 ASP A 136      41.334  24.407  22.927  1.00  0.00           O
ATOM   1850  C   ASP A 136      44.649  23.626  21.915  1.00  0.00           C
ATOM   1851  O   ASP A 136      44.622  24.085  20.771  1.00  0.00           O
ATOM   1852  N   LEU A 137      44.587  22.311  22.132  1.00  0.00           N
ATOM   1853  H   LEU A 137      44.691  21.985  23.090  1.00  0.00           H
ATOM   1854  CA  LEU A 137      44.529  21.324  21.048  1.00  0.00           C
ATOM   1855  HA  LEU A 137      45.018  21.732  20.162  1.00  0.00           H
ATOM   1856  CB  LEU A 137      45.293  20.055  21.482  1.00  0.00           C
ATOM   1857  HB1 LEU A 137      44.788  19.627  22.348  1.00  0.00           H
ATOM   1858  HB2 LEU A 137      45.239  19.328  20.670  1.00  0.00           H
ATOM   1859  CG  LEU A 137      46.778  20.269  21.853  1.00  0.00           C
ATOM   1860  HG  LEU A 137      46.850  20.959  22.695  1.00  0.00           H
ATOM   1861  CD1 LEU A 137      47.375  18.925  22.291  1.00  0.00           C
ATOM   1862 HD11 LEU A 137      48.414  19.058  22.592  1.00  0.00           H
ATOM   1863 HD12 LEU A 137      46.815  18.528  23.138  1.00  0.00           H
ATOM   1864 HD13 LEU A 137      47.331  18.216  21.465  1.00  0.00           H
ATOM   1865  CD2 LEU A 137      47.593  20.840  20.682  1.00  0.00           C
ATOM   1866 HD21 LEU A 137      48.648  20.872  20.949  1.00  0.00           H
ATOM   1867 HD22 LEU A 137      47.468  20.218  19.796  1.00  0.00           H
ATOM   1868 HD23 LEU A 137      47.268  21.857  20.464  1.00  0.00           H
ATOM   1869  C   LEU A 137      43.078  20.999  20.662  1.00  0.00           C
ATOM   1870  O   LEU A 137      42.373  20.364  21.441  1.00  0.00           O
ATOM   1871  N   ASP A 138      42.634  21.334  19.375  1.00  0.00           N
ATOM   1872  H   ASP A 138      43.188  21.943  18.764  1.00  0.00           H
ATOM   1873  CA  ASP A 138      41.271  21.028  18.928  1.00  0.00           C
ATOM   1874  HA  ASP A 138      40.798  20.407  19.704  1.00  0.00           H
ATOM   1875  CB  ASP A 138      40.352  22.227  18.724  1.00  0.00           C
ATOM   1876  HB1 ASP A 138      40.462  22.916  19.574  1.00  0.00           H
ATOM   1877  HB2 ASP A 138      40.639  22.791  17.826  1.00  0.00           H
ATOM   1878  CG  ASP A 138      38.867  21.822  18.618  1.00  0.00           C
ATOM   1879  OD1 ASP A 138      38.591  20.570  18.664  1.00  0.00           O
ATOM   1880  OD2 ASP A 138      38.031  22.759  18.517  1.00  0.00           O
ATOM   1881  C   ASP A 138      41.404  20.124  17.700  1.00  0.00           C
ATOM   1882  O   ASP A 138      41.182  20.506  16.553  1.00  0.00           O
ATOM   1883  N   TRP A 139      41.807  18.834  17.936  1.00  0.00           N
ATOM   1884  H   TRP A 139      42.046  18.517  18.889  1.00  0.00           H
ATOM   1885  CA  TRP A 139      41.768  17.790  16.903  1.00  0.00           C
ATOM   1886  HA  TRP A 139      41.908  18.228  15.914  1.00  0.00           H
ATOM   1887  CB  TRP A 139      42.948  16.833  17.151  1.00  0.00           C
```

ATOM	1888	HB1	TRP	A	139	43.849	17.446	17.220	1.00	0.00	H
ATOM	1889	HB2	TRP	A	139	42.810	16.360	18.124	1.00	0.00	H
ATOM	1890	CG	TRP	A	139	43.254	15.745	16.154	1.00	0.00	C
ATOM	1891	CD1	TRP	A	139	42.475	15.285	15.145	1.00	0.00	C
ATOM	1892	HD1	TRP	A	139	41.498	15.648	14.878	1.00	0.00	H
ATOM	1893	NE1	TRP	A	139	43.105	14.248	14.492	1.00	0.00	N
ATOM	1894	HE1	TRP	A	139	42.675	13.706	13.755	1.00	0.00	H
ATOM	1895	CE2	TRP	A	139	44.364	14.031	14.990	1.00	0.00	C
ATOM	1896	CZ2	TRP	A	139	45.395	13.162	14.619	1.00	0.00	C
ATOM	1897	HZ2	TRP	A	139	45.260	12.485	13.791	1.00	0.00	H
ATOM	1898	CH2	TRP	A	139	46.616	13.219	15.306	1.00	0.00	C
ATOM	1899	HH2	TRP	A	139	47.434	12.582	14.994	1.00	0.00	H
ATOM	1900	CZ3	TRP	A	139	46.774	14.125	16.372	1.00	0.00	C
ATOM	1901	HZ3	TRP	A	139	47.720	14.188	16.894	1.00	0.00	H
ATOM	1902	CE3	TRP	A	139	45.725	14.988	16.741	1.00	0.00	C
ATOM	1903	HE3	TRP	A	139	45.878	15.704	17.535	1.00	0.00	H
ATOM	1904	CD2	TRP	A	139	44.491	14.969	16.056	1.00	0.00	C
ATOM	1905	C	TRP	A	139	40.404	17.077	16.949	1.00	0.00	C
ATOM	1906	O	TRP	A	139	40.000	16.602	18.003	1.00	0.00	O
ATOM	1907	N	GLH	A	140	39.659	16.985	15.768	1.00	0.00	N
ATOM	1908	H	GLH	A	140	39.968	17.566	14.977	1.00	0.00	H
ATOM	1909	CA	GLH	A	140	38.393	16.253	15.660	1.00	0.00	C
ATOM	1910	HA	GLH	A	140	38.189	15.795	16.636	1.00	0.00	H
ATOM	1911	CB	GLH	A	140	37.207	17.146	15.328	1.00	0.00	C
ATOM	1912	HB1	GLH	A	140	37.417	17.719	14.413	1.00	0.00	H
ATOM	1913	HB2	GLH	A	140	36.334	16.512	15.118	1.00	0.00	H
ATOM	1914	CG	GLH	A	140	36.883	18.071	16.489	1.00	0.00	C
ATOM	1915	HG1	GLH	A	140	36.957	17.497	17.433	1.00	0.00	H
ATOM	1916	HG2	GLH	A	140	37.574	18.919	16.587	1.00	0.00	H
ATOM	1917	CD	GLH	A	140	35.464	18.585	16.480	1.00	0.00	C
ATOM	1918	OE1	GLH	A	140	34.511	17.980	15.983	1.00	0.00	O
ATOM	1919	OE2	GLH	A	140	35.364	19.738	17.122	1.00	0.00	O
ATOM	1920	HE2	GLH	A	140	34.384	20.041	17.201	1.00	0.00	H
ATOM	1921	C	GLH	A	140	38.580	15.135	14.630	1.00	0.00	C
ATOM	1922	O	GLH	A	140	38.235	15.310	13.461	1.00	0.00	O
ATOM	1923	N	TYR	A	141	39.138	13.933	14.960	1.00	0.00	N
ATOM	1924	H	TYR	A	141	39.256	13.207	14.227	1.00	0.00	H
ATOM	1925	CA	TYR	A	141	39.647	13.513	16.281	1.00	0.00	C
ATOM	1926	HA	TYR	A	141	39.984	14.369	16.858	1.00	0.00	H
ATOM	1927	CB	TYR	A	141	38.542	12.769	17.058	1.00	0.00	C
ATOM	1928	HB1	TYR	A	141	38.284	11.876	16.496	1.00	0.00	H
ATOM	1929	HB2	TYR	A	141	38.943	12.428	18.012	1.00	0.00	H
ATOM	1930	CG	TYR	A	141	37.270	13.547	17.335	1.00	0.00	C
ATOM	1931	CD1	TYR	A	141	36.224	13.543	16.391	1.00	0.00	C
ATOM	1932	HD1	TYR	A	141	36.321	12.970	15.480	1.00	0.00	H
ATOM	1933	CE1	TYR	A	141	35.057	14.293	16.624	1.00	0.00	C
ATOM	1934	HE1	TYR	A	141	34.250	14.291	15.905	1.00	0.00	H
ATOM	1935	CZ	TYR	A	141	34.920	15.035	17.816	1.00	0.00	C
ATOM	1936	OH	TYR	A	141	33.769	15.723	18.048	1.00	0.00	O
ATOM	1937	HH	TYR	A	141	33.733	16.146	18.908	1.00	0.00	H
ATOM	1938	CE2	TYR	A	141	35.970	15.044	18.763	1.00	0.00	C
ATOM	1939	HE2	TYR	A	141	35.869	15.618	19.669	1.00	0.00	H
ATOM	1940	CD2	TYR	A	141	37.141	14.300	18.520	1.00	0.00	C
ATOM	1941	HD2	TYR	A	141	37.947	14.312	19.241	1.00	0.00	H

ATOM	1942	C	TYR	A	141	40.840	12.553	16.095	1.00	0.00	C
ATOM	1943	O	TYR	A	141	40.942	11.955	15.021	1.00	0.00	O
ATOM	1944	N	PRO	A	142	41.705	12.339	17.105	1.00	0.00	N
ATOM	1945	CD	PRO	A	142	41.815	13.075	18.359	1.00	0.00	C
ATOM	1946	HD1	PRO	A	142	40.842	13.319	18.786	1.00	0.00	H
ATOM	1947	HD2	PRO	A	142	42.387	13.987	18.191	1.00	0.00	H
ATOM	1948	CG	PRO	A	142	42.585	12.159	19.309	1.00	0.00	C
ATOM	1949	HG1	PRO	A	142	41.885	11.512	19.835	1.00	0.00	H
ATOM	1950	HG2	PRO	A	142	43.181	12.724	20.025	1.00	0.00	H
ATOM	1951	CB	PRO	A	142	43.455	11.326	18.365	1.00	0.00	C
ATOM	1952	HB1	PRO	A	142	43.717	10.362	18.802	1.00	0.00	H
ATOM	1953	HB2	PRO	A	142	44.356	11.887	18.112	1.00	0.00	H
ATOM	1954	CA	PRO	A	142	42.585	11.178	17.112	1.00	0.00	C
ATOM	1955	HA	PRO	A	142	43.220	11.173	16.224	1.00	0.00	H
ATOM	1956	C	PRO	A	142	41.715	9.914	17.136	1.00	0.00	C
ATOM	1957	O	PRO	A	142	40.800	9.808	17.950	1.00	0.00	O
ATOM	1958	N	GLY	A	143	41.959	8.981	16.215	1.00	0.00	N
ATOM	1959	H	GLY	A	143	42.670	9.170	15.512	1.00	0.00	H
ATOM	1960	CA	GLY	A	143	41.276	7.687	16.133	1.00	0.00	C
ATOM	1961	HA1	GLY	A	143	41.861	7.033	15.487	1.00	0.00	H
ATOM	1962	HA2	GLY	A	143	41.218	7.255	17.132	1.00	0.00	H
ATOM	1963	C	GLY	A	143	39.851	7.711	15.570	1.00	0.00	C
ATOM	1964	O	GLY	A	143	39.200	6.672	15.592	1.00	0.00	O
ATOM	1965	N	SER	A	144	39.337	8.836	15.065	1.00	0.00	N
ATOM	1966	H	SER	A	144	39.940	9.645	14.994	1.00	0.00	H
ATOM	1967	CA	SER	A	144	37.996	8.911	14.445	1.00	0.00	C
ATOM	1968	HA	SER	A	144	37.741	7.934	14.037	1.00	0.00	H
ATOM	1969	CB	SER	A	144	36.913	9.270	15.484	1.00	0.00	C
ATOM	1970	HB1	SER	A	144	37.098	10.267	15.877	1.00	0.00	H
ATOM	1971	HB2	SER	A	144	35.941	9.288	14.993	1.00	0.00	H
ATOM	1972	OG	SER	A	144	36.840	8.353	16.563	1.00	0.00	O
ATOM	1973	HG	SER	A	144	37.435	8.678	17.273	1.00	0.00	H
ATOM	1974	C	SER	A	144	37.954	9.912	13.272	1.00	0.00	C
ATOM	1975	O	SER	A	144	38.924	10.634	13.043	1.00	0.00	O
ATOM	1976	N	GLN	A	145	36.843	9.982	12.519	1.00	0.00	N
ATOM	1977	H	GLN	A	145	36.074	9.352	12.734	1.00	0.00	H
ATOM	1978	CA	GLN	A	145	36.625	10.973	11.442	1.00	0.00	C
ATOM	1979	HA	GLN	A	145	35.701	10.701	10.929	1.00	0.00	H
ATOM	1980	CB	GLN	A	145	36.431	12.384	12.054	1.00	0.00	C
ATOM	1981	HB1	GLN	A	145	37.136	12.542	12.870	1.00	0.00	H
ATOM	1982	HB2	GLN	A	145	36.629	13.156	11.311	1.00	0.00	H
ATOM	1983	CG	GLN	A	145	34.998	12.597	12.573	1.00	0.00	C
ATOM	1984	HG1	GLN	A	145	34.732	11.787	13.254	1.00	0.00	H
ATOM	1985	HG2	GLN	A	145	34.960	13.530	13.133	1.00	0.00	H
ATOM	1986	CD	GLN	A	145	33.980	12.677	11.430	1.00	0.00	C
ATOM	1987	OE1	GLN	A	145	34.176	13.356	10.432	1.00	0.00	O
ATOM	1988	NE2	GLN	A	145	32.882	11.958	11.493	1.00	0.00	N
ATOM	1989	HE21	GLN	A	145	32.666	11.382	12.300	1.00	0.00	H
ATOM	1990	HE22	GLN	A	145	32.262	12.003	10.701	1.00	0.00	H
ATOM	1991	C	GLN	A	145	37.687	10.918	10.316	1.00	0.00	C
ATOM	1992	O	GLN	A	145	37.960	11.922	9.651	1.00	0.00	O
ATOM	1993	N	GLY	A	146	38.277	9.736	10.090	1.00	0.00	N
ATOM	1994	H	GLY	A	146	38.028	8.960	10.692	1.00	0.00	H
ATOM	1995	CA	GLY	A	146	39.301	9.459	9.073	1.00	0.00	C

ATOM	1996	HA1	GLY	A	146	39.014	8.564	8.522	1.00	0.00	H
ATOM	1997	HA2	GLY	A	146	39.388	10.296	8.381	1.00	0.00	H
ATOM	1998	C	GLY	A	146	40.685	9.188	9.664	1.00	0.00	C
ATOM	1999	O	GLY	A	146	41.514	8.550	9.019	1.00	0.00	O
ATOM	2000	N	SER	A	147	40.935	9.610	10.904	1.00	0.00	N
ATOM	2001	H	SER	A	147	40.183	10.058	11.418	1.00	0.00	H
ATOM	2002	CA	SER	A	147	42.196	9.339	11.604	1.00	0.00	C
ATOM	2003	HA	SER	A	147	43.010	9.672	10.966	1.00	0.00	H
ATOM	2004	CB	SER	A	147	42.241	10.173	12.886	1.00	0.00	C
ATOM	2005	HB1	SER	A	147	42.359	11.224	12.636	1.00	0.00	H
ATOM	2006	HB2	SER	A	147	41.291	10.044	13.400	1.00	0.00	H
ATOM	2007	OG	SER	A	147	43.251	9.792	13.792	1.00	0.00	O
ATOM	2008	HG	SER	A	147	44.165	9.943	13.447	1.00	0.00	H
ATOM	2009	C	SER	A	147	42.363	7.836	11.921	1.00	0.00	C
ATOM	2010	O	SER	A	147	41.424	7.232	12.459	1.00	0.00	O
ATOM	2011	N	PRO	A	148	43.528	7.230	11.604	1.00	0.00	N
ATOM	2012	CD	PRO	A	148	44.639	7.816	10.862	1.00	0.00	C
ATOM	2013	HD1	PRO	A	148	44.963	8.757	11.303	1.00	0.00	H
ATOM	2014	HD2	PRO	A	148	44.339	7.970	9.824	1.00	0.00	H
ATOM	2015	CG	PRO	A	148	45.779	6.803	10.921	1.00	0.00	C
ATOM	2016	HG1	PRO	A	148	46.383	6.994	11.809	1.00	0.00	H
ATOM	2017	HG2	PRO	A	148	46.395	6.835	10.022	1.00	0.00	H
ATOM	2018	CB	PRO	A	148	45.042	5.472	11.062	1.00	0.00	C
ATOM	2019	HB1	PRO	A	148	45.664	4.706	11.526	1.00	0.00	H
ATOM	2020	HB2	PRO	A	148	44.705	5.139	10.079	1.00	0.00	H
ATOM	2021	CA	PRO	A	148	43.826	5.837	11.920	1.00	0.00	C
ATOM	2022	HA	PRO	A	148	42.983	5.206	11.637	1.00	0.00	H
ATOM	2023	C	PRO	A	148	44.103	5.664	13.417	1.00	0.00	C
ATOM	2024	O	PRO	A	148	44.572	6.579	14.090	1.00	0.00	O
ATOM	2025	N	ALA	A	149	43.838	4.466	13.944	1.00	0.00	N
ATOM	2026	H	ALA	A	149	43.500	3.734	13.334	1.00	0.00	H
ATOM	2027	CA	ALA	A	149	43.797	4.209	15.386	1.00	0.00	C
ATOM	2028	HA	ALA	A	149	43.117	4.938	15.830	1.00	0.00	H
ATOM	2029	CB	ALA	A	149	43.192	2.818	15.583	1.00	0.00	C
ATOM	2030	HB1	ALA	A	149	43.144	2.567	16.642	1.00	0.00	H
ATOM	2031	HB2	ALA	A	149	42.186	2.788	15.163	1.00	0.00	H
ATOM	2032	HB3	ALA	A	149	43.801	2.071	15.087	1.00	0.00	H
ATOM	2033	C	ALA	A	149	45.146	4.378	16.117	1.00	0.00	C
ATOM	2034	O	ALA	A	149	45.148	4.589	17.335	1.00	0.00	O
ATOM	2035	N	VAL	A	150	46.267	4.365	15.386	1.00	0.00	N
ATOM	2036	H	VAL	A	150	46.151	4.091	14.419	1.00	0.00	H
ATOM	2037	CA	VAL	A	150	47.612	4.764	15.869	1.00	0.00	C
ATOM	2038	HA	VAL	A	150	47.892	4.057	16.648	1.00	0.00	H
ATOM	2039	CB	VAL	A	150	48.700	4.707	14.767	1.00	0.00	C
ATOM	2040	HB	VAL	A	150	49.643	5.052	15.198	1.00	0.00	H
ATOM	2041	CG1	VAL	A	150	48.925	3.274	14.281	1.00	0.00	C
ATOM	2042	HG11	VAL	A	150	49.787	3.240	13.614	1.00	0.00	H
ATOM	2043	HG12	VAL	A	150	49.114	2.617	15.130	1.00	0.00	H
ATOM	2044	HG13	VAL	A	150	48.044	2.936	13.739	1.00	0.00	H
ATOM	2045	CG2	VAL	A	150	48.386	5.595	13.549	1.00	0.00	C
ATOM	2046	HG21	VAL	A	150	49.169	5.483	12.799	1.00	0.00	H
ATOM	2047	HG22	VAL	A	150	47.435	5.303	13.107	1.00	0.00	H
ATOM	2048	HG23	VAL	A	150	48.343	6.643	13.842	1.00	0.00	H
ATOM	2049	C	VAL	A	150	47.655	6.173	16.480	1.00	0.00	C

```
ATOM   2050  O   VAL A 150      48.501   6.451  17.332  1.00  0.00           O
ATOM   2051  N   ASP A 151      46.739   7.060  16.074  1.00  0.00           N
ATOM   2052  H   ASP A 151      46.066   6.791  15.362  1.00  0.00           H
ATOM   2053  CA  ASP A 151      46.696   8.440  16.552  1.00  0.00           C
ATOM   2054  HA  ASP A 151      47.703   8.836  16.503  1.00  0.00           H
ATOM   2055  CB  ASP A 151      45.850   9.310  15.606  1.00  0.00           C
ATOM   2056  HB1 ASP A 151      44.889   8.819  15.468  1.00  0.00           H
ATOM   2057  HB2 ASP A 151      45.658  10.268  16.091  1.00  0.00           H
ATOM   2058  CG  ASP A 151      46.502   9.610  14.237  1.00  0.00           C
ATOM   2059  OD1 ASP A 151      47.735   9.455  14.083  1.00  0.00           O
ATOM   2060  OD2 ASP A 151      45.767  10.069  13.326  1.00  0.00           O
ATOM   2061  C   ASP A 151      46.250   8.550  18.026  1.00  0.00           C
ATOM   2062  O   ASP A 151      46.450   9.600  18.631  1.00  0.00           O
ATOM   2063  N   LYS A 152      45.730   7.484  18.655  1.00  0.00           N
ATOM   2064  H   LYS A 152      45.595   6.634  18.120  1.00  0.00           H
ATOM   2065  CA  LYS A 152      45.461   7.469  20.104  1.00  0.00           C
ATOM   2066  HA  LYS A 152      44.904   8.376  20.348  1.00  0.00           H
ATOM   2067  CB  LYS A 152      44.567   6.266  20.461  1.00  0.00           C
ATOM   2068  HB1 LYS A 152      43.638   6.366  19.902  1.00  0.00           H
ATOM   2069  HB2 LYS A 152      45.048   5.338  20.158  1.00  0.00           H
ATOM   2070  CG  LYS A 152      44.239   6.187  21.963  1.00  0.00           C
ATOM   2071  HG1 LYS A 152      45.151   5.970  22.520  1.00  0.00           H
ATOM   2072  HG2 LYS A 152      43.844   7.144  22.299  1.00  0.00           H
ATOM   2073  CD  LYS A 152      43.208   5.091  22.262  1.00  0.00           C
ATOM   2074  HD1 LYS A 152      42.314   5.257  21.662  1.00  0.00           H
ATOM   2075  HD2 LYS A 152      43.633   4.121  21.997  1.00  0.00           H
ATOM   2076  CE  LYS A 152      42.826   5.098  23.747  1.00  0.00           C
ATOM   2077  HE1 LYS A 152      43.724   4.914  24.344  1.00  0.00           H
ATOM   2078  HE2 LYS A 152      42.427   6.080  24.016  1.00  0.00           H
ATOM   2079  NZ  LYS A 152      41.821   4.057  24.058  1.00  0.00           N
ATOM   2080  HZ1 LYS A 152      40.980   4.168  23.502  1.00  0.00           H
ATOM   2081  HZ2 LYS A 152      42.208   3.135  23.880  1.00  0.00           H
ATOM   2082  HZ3 LYS A 152      41.556   4.097  25.039  1.00  0.00           H
ATOM   2083  C   LYS A 152      46.763   7.539  20.927  1.00  0.00           C
ATOM   2084  O   LYS A 152      46.942   8.494  21.686  1.00  0.00           O
ATOM   2085  N   GLU A 153      47.694   6.594  20.733  1.00  0.00           N
ATOM   2086  H   GLU A 153      47.477   5.837  20.091  1.00  0.00           H
ATOM   2087  CA  GLU A 153      49.034   6.618  21.350  1.00  0.00           C
ATOM   2088  HA  GLU A 153      48.935   6.644  22.436  1.00  0.00           H
ATOM   2089  CB  GLU A 153      49.840   5.364  20.949  1.00  0.00           C
ATOM   2090  HB1 GLU A 153      49.688   5.165  19.887  1.00  0.00           H
ATOM   2091  HB2 GLU A 153      50.902   5.579  21.074  1.00  0.00           H
ATOM   2092  CG  GLU A 153      49.502   4.099  21.758  1.00  0.00           C
ATOM   2093  HG1 GLU A 153      48.678   4.299  22.445  1.00  0.00           H
ATOM   2094  HG2 GLU A 153      49.184   3.313  21.072  1.00  0.00           H
ATOM   2095  CD  GLU A 153      50.710   3.603  22.559  1.00  0.00           C
ATOM   2096  OE1 GLU A 153      51.231   2.497  22.287  1.00  0.00           O
ATOM   2097  OE2 GLU A 153      51.155   4.328  23.483  1.00  0.00           O
ATOM   2098  C   GLU A 153      49.835   7.860  20.930  1.00  0.00           C
ATOM   2099  O   GLU A 153      50.592   8.420  21.728  1.00  0.00           O
ATOM   2100  N   ARG A 154      49.700   8.306  19.676  1.00  0.00           N
ATOM   2101  H   ARG A 154      49.113   7.793  19.028  1.00  0.00           H
ATOM   2102  CA  ARG A 154      50.449   9.466  19.185  1.00  0.00           C
ATOM   2103  HA  ARG A 154      51.479   9.376  19.526  1.00  0.00           H
```

ATOM	2104	CB	ARG	A	154	50.493	9.460	17.655	1.00	0.00	C
ATOM	2105	HB1	ARG	A	154	49.494	9.343	17.258	1.00	0.00	H
ATOM	2106	HB2	ARG	A	154	50.880	10.416	17.330	1.00	0.00	H
ATOM	2107	CG	ARG	A	154	51.412	8.359	17.107	1.00	0.00	C
ATOM	2108	HG1	ARG	A	154	52.423	8.535	17.480	1.00	0.00	H
ATOM	2109	HG2	ARG	A	154	51.078	7.383	17.461	1.00	0.00	H
ATOM	2110	CD	ARG	A	154	51.443	8.348	15.574	1.00	0.00	C
ATOM	2111	HD1	ARG	A	154	50.461	8.053	15.197	1.00	0.00	H
ATOM	2112	HD2	ARG	A	154	51.679	9.348	15.215	1.00	0.00	H
ATOM	2113	NE	ARG	A	154	52.484	7.426	15.104	1.00	0.00	N
ATOM	2114	HE	ARG	A	154	53.247	7.246	15.749	1.00	0.00	H
ATOM	2115	CZ	ARG	A	154	52.542	6.755	13.965	1.00	0.00	C
ATOM	2116	NH1	ARG	A	154	51.666	6.910	13.000	1.00	0.00	N
ATOM	2117	HH11	ARG	A	154	50.953	7.631	13.060	1.00	0.00	H
ATOM	2118	HH12	ARG	A	154	51.728	6.337	12.170	1.00	0.00	H
ATOM	2119	NH2	ARG	A	154	53.519	5.898	13.784	1.00	0.00	N
ATOM	2120	HH21	ARG	A	154	54.236	5.786	14.494	1.00	0.00	H
ATOM	2121	HH22	ARG	A	154	53.543	5.350	12.932	1.00	0.00	H
ATOM	2122	C	ARG	A	154	49.936	10.799	19.753	1.00	0.00	C
ATOM	2123	O	ARG	A	154	50.748	11.701	19.957	1.00	0.00	O
ATOM	2124	N	PHE	A	155	48.652	10.916	20.120	1.00	0.00	N
ATOM	2125	H	PHE	A	155	48.015	10.161	19.905	1.00	0.00	H
ATOM	2126	CA	PHE	A	155	48.103	12.129	20.744	1.00	0.00	C
ATOM	2127	HA	PHE	A	155	48.399	12.994	20.146	1.00	0.00	H
ATOM	2128	CB	PHE	A	155	46.563	12.063	20.767	1.00	0.00	C
ATOM	2129	HB1	PHE	A	155	46.211	11.883	19.752	1.00	0.00	H
ATOM	2130	HB2	PHE	A	155	46.238	11.219	21.373	1.00	0.00	H
ATOM	2131	CG	PHE	A	155	45.890	13.329	21.272	1.00	0.00	C
ATOM	2132	CD1	PHE	A	155	45.465	14.308	20.353	1.00	0.00	C
ATOM	2133	HD1	PHE	A	155	45.590	14.133	19.295	1.00	0.00	H
ATOM	2134	CE1	PHE	A	155	44.914	15.521	20.804	1.00	0.00	C
ATOM	2135	HE1	PHE	A	155	44.621	16.276	20.089	1.00	0.00	H
ATOM	2136	CZ	PHE	A	155	44.777	15.758	22.184	1.00	0.00	C
ATOM	2137	HZ	PHE	A	155	44.377	16.696	22.542	1.00	0.00	H
ATOM	2138	CE2	PHE	A	155	45.173	14.772	23.105	1.00	0.00	C
ATOM	2139	HE2	PHE	A	155	45.065	14.949	24.167	1.00	0.00	H
ATOM	2140	CD2	PHE	A	155	45.724	13.559	22.651	1.00	0.00	C
ATOM	2141	HD2	PHE	A	155	46.036	12.814	23.369	1.00	0.00	H
ATOM	2142	C	PHE	A	155	48.686	12.301	22.150	1.00	0.00	C
ATOM	2143	O	PHE	A	155	49.100	13.402	22.530	1.00	0.00	O
ATOM	2144	N	THR	A	156	48.792	11.189	22.885	1.00	0.00	N
ATOM	2145	H	THR	A	156	48.388	10.335	22.513	1.00	0.00	H
ATOM	2146	CA	THR	A	156	49.423	11.094	24.204	1.00	0.00	C
ATOM	2147	HA	THR	A	156	48.890	11.734	24.905	1.00	0.00	H
ATOM	2148	CB	THR	A	156	49.349	9.650	24.694	1.00	0.00	C
ATOM	2149	HB	THR	A	156	50.027	9.023	24.119	1.00	0.00	H
ATOM	2150	CG2	THR	A	156	49.695	9.564	26.170	1.00	0.00	C
ATOM	2151	HG21	THR	A	156	49.588	8.535	26.490	1.00	0.00	H
ATOM	2152	HG22	THR	A	156	50.729	9.867	26.325	1.00	0.00	H
ATOM	2153	HG23	THR	A	156	49.032	10.204	26.752	1.00	0.00	H
ATOM	2154	OG1	THR	A	156	48.048	9.162	24.515	1.00	0.00	O
ATOM	2155	HG1	THR	A	156	48.098	8.182	24.630	1.00	0.00	H
ATOM	2156	C	THR	A	156	50.874	11.550	24.154	1.00	0.00	C
ATOM	2157	O	THR	A	156	51.251	12.448	24.909	1.00	0.00	O

```
ATOM   2158  N    THR A 157      51.659  10.989  23.224  1.00  0.00           N
ATOM   2159  H    THR A 157      51.273  10.217  22.689  1.00  0.00           H
ATOM   2160  CA   THR A 157      53.045  11.398  22.960  1.00  0.00           C
ATOM   2161  HA   THR A 157      53.628  11.302  23.878  1.00  0.00           H
ATOM   2162  CB   THR A 157      53.670  10.478  21.909  1.00  0.00           C
ATOM   2163  HB   THR A 157      52.946  10.281  21.117  1.00  0.00           H
ATOM   2164  CG2  THR A 157      54.943  11.027  21.278  1.00  0.00           C
ATOM   2165 HG21 THR A 157      55.397  10.258  20.654  1.00  0.00           H
ATOM   2166 HG22 THR A 157      54.700  11.887  20.658  1.00  0.00           H
ATOM   2167 HG23 THR A 157      55.648  11.335  22.050  1.00  0.00           H
ATOM   2168  OG1 THR A 157      54.038   9.270  22.539  1.00  0.00           O
ATOM   2169  HG1 THR A 157      53.352   9.043  23.199  1.00  0.00           H
ATOM   2170  C    THR A 157      53.121  12.874  22.578  1.00  0.00           C
ATOM   2171  O    THR A 157      53.936  13.576  23.156  1.00  0.00           O
ATOM   2172  N    LEU A 158      52.260  13.391  21.694  1.00  0.00           N
ATOM   2173  H    LEU A 158      51.628  12.769  21.202  1.00  0.00           H
ATOM   2174  CA   LEU A 158      52.256  14.818  21.339  1.00  0.00           C
ATOM   2175  HA   LEU A 158      53.261  15.076  21.004  1.00  0.00           H
ATOM   2176  CB   LEU A 158      51.283  15.050  20.163  1.00  0.00           C
ATOM   2177  HB1  LEU A 158      51.631  14.478  19.301  1.00  0.00           H
ATOM   2178  HB2  LEU A 158      50.306  14.659  20.438  1.00  0.00           H
ATOM   2179  CG   LEU A 158      51.119  16.531  19.747  1.00  0.00           C
ATOM   2180  HG   LEU A 158      50.778  17.102  20.610  1.00  0.00           H
ATOM   2181  CD1  LEU A 158      52.437  17.141  19.239  1.00  0.00           C
ATOM   2182 HD11 LEU A 158      52.268  18.172  18.929  1.00  0.00           H
ATOM   2183 HD12 LEU A 158      53.187  17.143  20.029  1.00  0.00           H
ATOM   2184 HD13 LEU A 158      52.812  16.571  18.388  1.00  0.00           H
ATOM   2185  CD2  LEU A 158      50.050  16.680  18.655  1.00  0.00           C
ATOM   2186 HD21 LEU A 158      49.830  17.737  18.503  1.00  0.00           H
ATOM   2187 HD22 LEU A 158      50.401  16.252  17.720  1.00  0.00           H
ATOM   2188 HD23 LEU A 158      49.131  16.175  18.949  1.00  0.00           H
ATOM   2189  C    LEU A 158      51.981  15.724  22.555  1.00  0.00           C
ATOM   2190  O    LEU A 158      52.681  16.719  22.715  1.00  0.00           O
ATOM   2191  N    VAL A 159      51.048  15.362  23.443  1.00  0.00           N
ATOM   2192  H    VAL A 159      50.491  14.540  23.230  1.00  0.00           H
ATOM   2193  CA   VAL A 159      50.818  16.086  24.714  1.00  0.00           C
ATOM   2194  HA   VAL A 159      50.604  17.126  24.465  1.00  0.00           H
ATOM   2195  CB   VAL A 159      49.599  15.517  25.489  1.00  0.00           C
ATOM   2196  HB   VAL A 159      49.653  14.429  25.473  1.00  0.00           H
ATOM   2197  CG1  VAL A 159      49.543  15.961  26.960  1.00  0.00           C
ATOM   2198 HG11 VAL A 159      48.642  15.575  27.433  1.00  0.00           H
ATOM   2199 HG12 VAL A 159      50.396  15.559  27.500  1.00  0.00           H
ATOM   2200 HG13 VAL A 159      49.549  17.048  27.029  1.00  0.00           H
ATOM   2201  CG2  VAL A 159      48.279  15.933  24.823  1.00  0.00           C
ATOM   2202 HG21 VAL A 159      47.433  15.536  25.385  1.00  0.00           H
ATOM   2203 HG22 VAL A 159      48.205  17.020  24.781  1.00  0.00           H
ATOM   2204 HG23 VAL A 159      48.233  15.533  23.816  1.00  0.00           H
ATOM   2205  C    VAL A 159      52.076  16.068  25.601  1.00  0.00           C
ATOM   2206  O    VAL A 159      52.442  17.093  26.173  1.00  0.00           O
ATOM   2207  N    GLN A 160      52.745  14.916  25.706  1.00  0.00           N
ATOM   2208  H    GLN A 160      52.405  14.113  25.189  1.00  0.00           H
ATOM   2209  CA   GLN A 160      53.959  14.735  26.507  1.00  0.00           C
ATOM   2210  HA   GLN A 160      53.791  15.120  27.514  1.00  0.00           H
ATOM   2211  CB   GLN A 160      54.230  13.224  26.576  1.00  0.00           C
```

ATOM	2212	HB1	GLN	A	160	53.303	12.707	26.823	1.00	0.00	H
ATOM	2213	HB2	GLN	A	160	54.551	12.883	25.592	1.00	0.00	H
ATOM	2214	CG	GLN	A	160	55.280	12.805	27.613	1.00	0.00	C
ATOM	2215	HG1	GLN	A	160	56.223	13.312	27.426	1.00	0.00	H
ATOM	2216	HG2	GLN	A	160	54.933	13.085	28.609	1.00	0.00	H
ATOM	2217	CD	GLN	A	160	55.521	11.298	27.553	1.00	0.00	C
ATOM	2218	OE1	GLN	A	160	55.714	10.714	26.491	1.00	0.00	O
ATOM	2219	NE2	GLN	A	160	55.467	10.591	28.660	1.00	0.00	N
ATOM	2220	HE21	GLN	A	160	55.239	11.030	29.545	1.00	0.00	H
ATOM	2221	HE22	GLN	A	160	55.647	9.601	28.606	1.00	0.00	H
ATOM	2222	C	GLN	A	160	55.159	15.492	25.910	1.00	0.00	C
ATOM	2223	O	GLN	A	160	56.021	15.975	26.640	1.00	0.00	O
ATOM	2224	N	ASP	A	161	55.237	15.601	24.585	1.00	0.00	N
ATOM	2225	H	ASP	A	161	54.556	15.087	24.031	1.00	0.00	H
ATOM	2226	CA	ASP	A	161	56.285	16.311	23.850	1.00	0.00	C
ATOM	2227	HA	ASP	A	161	57.257	16.091	24.282	1.00	0.00	H
ATOM	2228	CB	ASP	A	161	56.295	15.841	22.387	1.00	0.00	C
ATOM	2229	HB1	ASP	A	161	55.285	15.902	21.980	1.00	0.00	H
ATOM	2230	HB2	ASP	A	161	56.917	16.530	21.814	1.00	0.00	H
ATOM	2231	CG	ASP	A	161	56.846	14.425	22.162	1.00	0.00	C
ATOM	2232	OD1	ASP	A	161	57.140	13.691	23.141	1.00	0.00	O
ATOM	2233	OD2	ASP	A	161	57.021	14.101	20.962	1.00	0.00	O
ATOM	2234	C	ASP	A	161	56.113	17.828	23.938	1.00	0.00	C
ATOM	2235	O	ASP	A	161	57.104	18.533	24.138	1.00	0.00	O
ATOM	2236	N	LEU	A	162	54.876	18.326	23.842	1.00	0.00	N
ATOM	2237	H	LEU	A	162	54.115	17.681	23.646	1.00	0.00	H
ATOM	2238	CA	LEU	A	162	54.550	19.733	24.079	1.00	0.00	C
ATOM	2239	HA	LEU	A	162	55.197	20.359	23.464	1.00	0.00	H
ATOM	2240	CB	LEU	A	162	53.078	19.984	23.693	1.00	0.00	C
ATOM	2241	HB1	LEU	A	162	52.469	19.200	24.145	1.00	0.00	H
ATOM	2242	HB2	LEU	A	162	52.756	20.941	24.108	1.00	0.00	H
ATOM	2243	CG	LEU	A	162	52.830	20.001	22.169	1.00	0.00	C
ATOM	2244	HG	LEU	A	162	53.394	19.193	21.702	1.00	0.00	H
ATOM	2245	CD1	LEU	A	162	51.342	19.783	21.864	1.00	0.00	C
ATOM	2246	HD11	LEU	A	162	51.189	19.727	20.786	1.00	0.00	H
ATOM	2247	HD12	LEU	A	162	50.999	18.852	22.316	1.00	0.00	H
ATOM	2248	HD13	LEU	A	162	50.756	20.609	22.263	1.00	0.00	H
ATOM	2249	CD2	LEU	A	162	53.262	21.336	21.542	1.00	0.00	C
ATOM	2250	HD21	LEU	A	162	53.203	21.269	20.456	1.00	0.00	H
ATOM	2251	HD22	LEU	A	162	52.607	22.137	21.882	1.00	0.00	H
ATOM	2252	HD23	LEU	A	162	54.285	21.580	21.825	1.00	0.00	H
ATOM	2253	C	LEU	A	162	54.848	20.110	25.535	1.00	0.00	C
ATOM	2254	O	LEU	A	162	55.607	21.046	25.768	1.00	0.00	O
ATOM	2255	N	ALA	A	163	54.336	19.359	26.516	1.00	0.00	N
ATOM	2256	H	ALA	A	163	53.742	18.578	26.260	1.00	0.00	H
ATOM	2257	CA	ALA	A	163	54.526	19.650	27.941	1.00	0.00	C
ATOM	2258	HA	ALA	A	163	54.115	20.641	28.144	1.00	0.00	H
ATOM	2259	CB	ALA	A	163	53.731	18.622	28.756	1.00	0.00	C
ATOM	2260	HB1	ALA	A	163	53.833	18.834	29.821	1.00	0.00	H
ATOM	2261	HB2	ALA	A	163	52.675	18.675	28.488	1.00	0.00	H
ATOM	2262	HB3	ALA	A	163	54.104	17.615	28.559	1.00	0.00	H
ATOM	2263	C	ALA	A	163	56.009	19.684	28.358	1.00	0.00	C
ATOM	2264	O	ALA	A	163	56.437	20.647	29.006	1.00	0.00	O
ATOM	2265	N	ASN	A	164	56.799	18.680	27.952	1.00	0.00	N

ATOM	2266	H	ASN	A	164	56.380	17.904	27.449	1.00	0.00	H
ATOM	2267	CA	ASN	A	164	58.231	18.610	28.269	1.00	0.00	C
ATOM	2268	HA	ASN	A	164	58.352	18.790	29.337	1.00	0.00	H
ATOM	2269	CB	ASN	A	164	58.771	17.197	27.963	1.00	0.00	C
ATOM	2270	HB1	ASN	A	164	58.496	16.916	26.946	1.00	0.00	H
ATOM	2271	HB2	ASN	A	164	59.860	17.218	28.010	1.00	0.00	H
ATOM	2272	CG	ASN	A	164	58.299	16.112	28.940	1.00	0.00	C
ATOM	2273	OD1	ASN	A	164	57.846	16.375	30.048	1.00	0.00	O
ATOM	2274	ND2	ASN	A	164	58.435	14.847	28.587	1.00	0.00	N
ATOM	2275	HD21	ASN	A	164	58.795	14.593	27.679	1.00	0.00	H
ATOM	2276	HD22	ASN	A	164	58.120	14.131	29.230	1.00	0.00	H
ATOM	2277	C	ASN	A	164	59.051	19.703	27.548	1.00	0.00	C
ATOM	2278	O	ASN	A	164	60.020	20.216	28.123	1.00	0.00	O
ATOM	2279	N	ALA	A	165	58.656	20.115	26.333	1.00	0.00	N
ATOM	2280	H	ALA	A	165	57.856	19.663	25.903	1.00	0.00	H
ATOM	2281	CA	ALA	A	165	59.301	21.209	25.595	1.00	0.00	C
ATOM	2282	HA	ALA	A	165	60.385	21.082	25.647	1.00	0.00	H
ATOM	2283	CB	ALA	A	165	58.878	21.140	24.126	1.00	0.00	C
ATOM	2284	HB1	ALA	A	165	59.360	21.941	23.564	1.00	0.00	H
ATOM	2285	HB2	ALA	A	165	59.187	20.193	23.693	1.00	0.00	H
ATOM	2286	HB3	ALA	A	165	57.795	21.243	24.035	1.00	0.00	H
ATOM	2287	C	ALA	A	165	58.965	22.592	26.168	1.00	0.00	C
ATOM	2288	O	ALA	A	165	59.853	23.443	26.253	1.00	0.00	O
ATOM	2289	N	PHE	A	166	57.709	22.801	26.585	1.00	0.00	N
ATOM	2290	H	PHE	A	166	57.023	22.069	26.423	1.00	0.00	H
ATOM	2291	CA	PHE	A	166	57.229	24.047	27.183	1.00	0.00	C
ATOM	2292	HA	PHE	A	166	57.480	24.882	26.526	1.00	0.00	H
ATOM	2293	CB	PHE	A	166	55.697	24.008	27.362	1.00	0.00	C
ATOM	2294	HB1	PHE	A	166	55.431	23.103	27.910	1.00	0.00	H
ATOM	2295	HB2	PHE	A	166	55.421	24.854	27.993	1.00	0.00	H
ATOM	2296	CG	PHE	A	166	54.811	24.083	26.120	1.00	0.00	C
ATOM	2297	CD1	PHE	A	166	55.326	24.004	24.807	1.00	0.00	C
ATOM	2298	HD1	PHE	A	166	56.384	23.896	24.634	1.00	0.00	H
ATOM	2299	CE1	PHE	A	166	54.462	24.032	23.699	1.00	0.00	C
ATOM	2300	HE1	PHE	A	166	54.867	23.940	22.700	1.00	0.00	H
ATOM	2301	CZ	PHE	A	166	53.077	24.172	23.887	1.00	0.00	C
ATOM	2302	HZ	PHE	A	166	52.410	24.197	23.037	1.00	0.00	H
ATOM	2303	CE2	PHE	A	166	52.557	24.269	25.187	1.00	0.00	C
ATOM	2304	HE2	PHE	A	166	51.492	24.373	25.337	1.00	0.00	H
ATOM	2305	CD2	PHE	A	166	53.419	24.215	26.296	1.00	0.00	C
ATOM	2306	HD2	PHE	A	166	53.005	24.271	27.292	1.00	0.00	H
ATOM	2307	C	PHE	A	166	57.928	24.299	28.521	1.00	0.00	C
ATOM	2308	O	PHE	A	166	58.332	25.433	28.780	1.00	0.00	O
ATOM	2309	N	GLN	A	167	58.150	23.265	29.348	1.00	0.00	N
ATOM	2310	H	GLN	A	167	57.750	22.355	29.134	1.00	0.00	H
ATOM	2311	CA	GLN	A	167	59.014	23.429	30.518	1.00	0.00	C
ATOM	2312	HA	GLN	A	167	58.655	24.295	31.077	1.00	0.00	H
ATOM	2313	CB	GLN	A	167	58.967	22.219	31.470	1.00	0.00	C
ATOM	2314	HB1	GLN	A	167	57.943	22.077	31.821	1.00	0.00	H
ATOM	2315	HB2	GLN	A	167	59.298	21.318	30.951	1.00	0.00	H
ATOM	2316	CG	GLN	A	167	59.894	22.500	32.675	1.00	0.00	C
ATOM	2317	HG1	GLN	A	167	60.929	22.492	32.338	1.00	0.00	H
ATOM	2318	HG2	GLN	A	167	59.671	23.492	33.070	1.00	0.00	H
ATOM	2319	CD	GLN	A	167	59.812	21.514	33.831	1.00	0.00	C

ATOM	2320	OE1	GLN	A	167	59.230	20.436	33.752	1.00	0.00	O
ATOM	2321	NE2	GLN	A	167	60.423	21.834	34.948	1.00	0.00	N
ATOM	2322	HE21	GLN	A	167	60.939	22.707	35.022	1.00	0.00	H
ATOM	2323	HE22	GLN	A	167	60.333	21.221	35.752	1.00	0.00	H
ATOM	2324	C	GLN	A	167	60.451	23.770	30.108	1.00	0.00	C
ATOM	2325	O	GLN	A	167	61.014	24.684	30.699	1.00	0.00	O
ATOM	2326	N	GLN	A	168	61.057	23.095	29.123	1.00	0.00	N
ATOM	2327	H	GLN	A	168	60.571	22.347	28.644	1.00	0.00	H
ATOM	2328	CA	GLN	A	168	62.461	23.359	28.790	1.00	0.00	C
ATOM	2329	HA	GLN	A	168	63.028	23.263	29.716	1.00	0.00	H
ATOM	2330	CB	GLN	A	168	62.987	22.318	27.784	1.00	0.00	C
ATOM	2331	HB1	GLN	A	168	62.692	21.320	28.112	1.00	0.00	H
ATOM	2332	HB2	GLN	A	168	62.538	22.503	26.807	1.00	0.00	H
ATOM	2333	CG	GLN	A	168	64.523	22.347	27.645	1.00	0.00	C
ATOM	2334	HG1	GLN	A	168	64.818	21.639	26.870	1.00	0.00	H
ATOM	2335	HG2	GLN	A	168	64.847	23.338	27.330	1.00	0.00	H
ATOM	2336	CD	GLN	A	168	65.236	21.986	28.949	1.00	0.00	C
ATOM	2337	OE1	GLN	A	168	65.427	22.804	29.838	1.00	0.00	O
ATOM	2338	NE2	GLN	A	168	65.614	20.747	29.169	1.00	0.00	N
ATOM	2339	HE21	GLN	A	168	65.587	20.052	28.437	1.00	0.00	H
ATOM	2340	HE22	GLN	A	168	66.028	20.543	30.066	1.00	0.00	H
ATOM	2341	C	GLN	A	168	62.688	24.804	28.314	1.00	0.00	C
ATOM	2342	O	GLN	A	168	63.667	25.423	28.728	1.00	0.00	O
ATOM	2343	N	GLU	A	169	61.763	25.388	27.543	1.00	0.00	N
ATOM	2344	H	GLU	A	169	60.984	24.836	27.198	1.00	0.00	H
ATOM	2345	CA	GLU	A	169	61.822	26.815	27.200	1.00	0.00	C
ATOM	2346	HA	GLU	A	169	62.796	27.006	26.753	1.00	0.00	H
ATOM	2347	CB	GLU	A	169	60.775	27.148	26.125	1.00	0.00	C
ATOM	2348	HB1	GLU	A	169	60.950	26.481	25.280	1.00	0.00	H
ATOM	2349	HB2	GLU	A	169	59.769	26.943	26.493	1.00	0.00	H
ATOM	2350	CG	GLU	A	169	60.891	28.595	25.603	1.00	0.00	C
ATOM	2351	HG1	GLU	A	169	61.941	28.890	25.576	1.00	0.00	H
ATOM	2352	HG2	GLU	A	169	60.528	28.619	24.578	1.00	0.00	H
ATOM	2353	CD	GLU	A	169	60.096	29.641	26.386	1.00	0.00	C
ATOM	2354	OE1	GLU	A	169	59.308	29.284	27.289	1.00	0.00	O
ATOM	2355	OE2	GLU	A	169	60.210	30.844	26.055	1.00	0.00	O
ATOM	2356	C	GLU	A	169	61.710	27.710	28.448	1.00	0.00	C
ATOM	2357	O	GLU	A	169	62.439	28.695	28.563	1.00	0.00	O
ATOM	2358	N	ALA	A	170	60.885	27.352	29.438	1.00	0.00	N
ATOM	2359	H	ALA	A	170	60.328	26.515	29.325	1.00	0.00	H
ATOM	2360	CA	ALA	A	170	60.770	28.099	30.695	1.00	0.00	C
ATOM	2361	HA	ALA	A	170	60.651	29.159	30.456	1.00	0.00	H
ATOM	2362	CB	ALA	A	170	59.509	27.633	31.428	1.00	0.00	C
ATOM	2363	HB1	ALA	A	170	59.331	28.275	32.292	1.00	0.00	H
ATOM	2364	HB2	ALA	A	170	58.658	27.703	30.755	1.00	0.00	H
ATOM	2365	HB3	ALA	A	170	59.621	26.603	31.766	1.00	0.00	H
ATOM	2366	C	ALA	A	170	62.019	27.973	31.588	1.00	0.00	C
ATOM	2367	O	ALA	A	170	62.348	28.917	32.307	1.00	0.00	O
ATOM	2368	N	GLN	A	171	62.732	26.842	31.545	1.00	0.00	N
ATOM	2369	H	GLN	A	171	62.374	26.083	30.972	1.00	0.00	H
ATOM	2370	CA	GLN	A	171	63.997	26.663	32.271	1.00	0.00	C
ATOM	2371	HA	GLN	A	171	63.903	27.087	33.274	1.00	0.00	H
ATOM	2372	CB	GLN	A	171	64.359	25.168	32.385	1.00	0.00	C
ATOM	2373	HB1	GLN	A	171	64.484	24.771	31.378	1.00	0.00	H

ATOM	2374	HB2	GLN	A	171	65.325	25.085	32.883	1.00	0.00	H
ATOM	2375	CG	GLN	A	171	63.366	24.253	33.126	1.00	0.00	C
ATOM	2376	HG1	GLN	A	171	62.446	24.204	32.560	1.00	0.00	H
ATOM	2377	HG2	GLN	A	171	63.792	23.250	33.150	1.00	0.00	H
ATOM	2378	CD	GLN	A	171	63.008	24.639	34.561	1.00	0.00	C
ATOM	2379	OE1	GLN	A	171	61.888	24.411	35.013	1.00	0.00	O
ATOM	2380	NE2	GLN	A	171	63.914	25.197	35.335	1.00	0.00	N
ATOM	2381	HE21	GLN	A	171	64.860	25.361	35.025	1.00	0.00	H
ATOM	2382	HE22	GLN	A	171	63.680	25.277	36.316	1.00	0.00	H
ATOM	2383	C	GLN	A	171	65.164	27.395	31.586	1.00	0.00	C
ATOM	2384	O	GLN	A	171	66.048	27.907	32.282	1.00	0.00	O
ATOM	2385	N	THR	A	172	65.184	27.428	30.243	1.00	0.00	N
ATOM	2386	H	THR	A	172	64.437	26.955	29.744	1.00	0.00	H
ATOM	2387	CA	THR	A	172	66.274	28.010	29.437	1.00	0.00	C
ATOM	2388	HA	THR	A	172	67.203	27.906	29.992	1.00	0.00	H
ATOM	2389	CB	THR	A	172	66.460	27.272	28.107	1.00	0.00	C
ATOM	2390	HB	THR	A	172	67.167	27.831	27.494	1.00	0.00	H
ATOM	2391	CG2	THR	A	172	67.018	25.863	28.310	1.00	0.00	C
ATOM	2392	HG21	THR	A	172	67.155	25.383	27.342	1.00	0.00	H
ATOM	2393	HG22	THR	A	172	67.986	25.923	28.808	1.00	0.00	H
ATOM	2394	HG23	THR	A	172	66.339	25.263	28.916	1.00	0.00	H
ATOM	2395	OG1	THR	A	172	65.242	27.185	27.412	1.00	0.00	O
ATOM	2396	HG1	THR	A	172	64.719	26.472	27.826	1.00	0.00	H
ATOM	2397	C	THR	A	172	66.115	29.504	29.183	1.00	0.00	C
ATOM	2398	O	THR	A	172	67.113	30.206	29.049	1.00	0.00	O
ATOM	2399	N	SER	A	173	64.890	30.020	29.156	1.00	0.00	N
ATOM	2400	H	SER	A	173	64.099	29.385	29.118	1.00	0.00	H
ATOM	2401	CA	SER	A	173	64.644	31.467	29.140	1.00	0.00	C
ATOM	2402	HA	SER	A	173	65.453	31.960	28.602	1.00	0.00	H
ATOM	2403	CB	SER	A	173	63.341	31.785	28.390	1.00	0.00	C
ATOM	2404	HB1	SER	A	173	63.239	32.867	28.316	1.00	0.00	H
ATOM	2405	HB2	SER	A	173	63.396	31.376	27.380	1.00	0.00	H
ATOM	2406	OG	SER	A	173	62.202	31.254	29.046	1.00	0.00	O
ATOM	2407	HG	SER	A	173	62.127	30.312	28.773	1.00	0.00	H
ATOM	2408	C	SER	A	173	64.615	32.075	30.553	1.00	0.00	C
ATOM	2409	O	SER	A	173	65.225	33.122	30.782	1.00	0.00	O
ATOM	2410	N	GLY	A	174	63.945	31.423	31.513	1.00	0.00	N
ATOM	2411	H	GLY	A	174	63.522	30.535	31.275	1.00	0.00	H
ATOM	2412	CA	GLY	A	174	63.601	31.988	32.828	1.00	0.00	C
ATOM	2413	HA1	GLY	A	174	63.596	31.185	33.564	1.00	0.00	H
ATOM	2414	HA2	GLY	A	174	64.333	32.744	33.115	1.00	0.00	H
ATOM	2415	C	GLY	A	174	62.214	32.645	32.869	1.00	0.00	C
ATOM	2416	O	GLY	A	174	61.828	33.193	33.900	1.00	0.00	O
ATOM	2417	N	LYS	A	175	61.449	32.607	31.773	1.00	0.00	N
ATOM	2418	H	LYS	A	175	61.818	32.136	30.955	1.00	0.00	H
ATOM	2419	CA	LYS	A	175	60.096	33.182	31.672	1.00	0.00	C
ATOM	2420	HA	LYS	A	175	60.099	34.182	32.109	1.00	0.00	H
ATOM	2421	CB	LYS	A	175	59.714	33.280	30.187	1.00	0.00	C
ATOM	2422	HB1	LYS	A	175	59.793	32.284	29.748	1.00	0.00	H
ATOM	2423	HB2	LYS	A	175	58.677	33.609	30.095	1.00	0.00	H
ATOM	2424	CG	LYS	A	175	60.593	34.253	29.396	1.00	0.00	C
ATOM	2425	HG1	LYS	A	175	60.399	35.272	29.734	1.00	0.00	H
ATOM	2426	HG2	LYS	A	175	61.647	34.025	29.556	1.00	0.00	H
ATOM	2427	CD	LYS	A	175	60.277	34.131	27.903	1.00	0.00	C

ATOM	2428	HD1	LYS	A	175	60.420	33.097	27.582	1.00	0.00	H
ATOM	2429	HD2	LYS	A	175	59.242	34.427	27.723	1.00	0.00	H
ATOM	2430	CE	LYS	A	175	61.223	35.041	27.124	1.00	0.00	C
ATOM	2431	HE1	LYS	A	175	61.107	36.064	27.489	1.00	0.00	H
ATOM	2432	HE2	LYS	A	175	62.254	34.727	27.307	1.00	0.00	H
ATOM	2433	NZ	LYS	A	175	60.939	35.001	25.677	1.00	0.00	N
ATOM	2434	HZ1	LYS	A	175	61.031	34.054	25.320	1.00	0.00	H
ATOM	2435	HZ2	LYS	A	175	60.005	35.347	25.481	1.00	0.00	H
ATOM	2436	HZ3	LYS	A	175	61.596	35.575	25.160	1.00	0.00	H
ATOM	2437	C	LYS	A	175	59.030	32.354	32.413	1.00	0.00	C
ATOM	2438	O	LYS	A	175	59.249	31.183	32.732	1.00	0.00	O
ATOM	2439	N	GLU	A	176	57.844	32.936	32.608	1.00	0.00	N
ATOM	2440	H	GLU	A	176	57.731	33.906	32.336	1.00	0.00	H
ATOM	2441	CA	GLU	A	176	56.638	32.210	33.015	1.00	0.00	C
ATOM	2442	HA	GLU	A	176	56.837	31.703	33.959	1.00	0.00	H
ATOM	2443	CB	GLU	A	176	55.512	33.227	33.244	1.00	0.00	C
ATOM	2444	HB1	GLU	A	176	55.873	33.988	33.937	1.00	0.00	H
ATOM	2445	HB2	GLU	A	176	55.274	33.715	32.298	1.00	0.00	H
ATOM	2446	CG	GLU	A	176	54.238	32.614	33.831	1.00	0.00	C
ATOM	2447	HG1	GLU	A	176	53.827	31.878	33.140	1.00	0.00	H
ATOM	2448	HG2	GLU	A	176	54.473	32.117	34.773	1.00	0.00	H
ATOM	2449	CD	GLU	A	176	53.211	33.714	34.071	1.00	0.00	C
ATOM	2450	OE1	GLU	A	176	52.641	34.227	33.075	1.00	0.00	O
ATOM	2451	OE2	GLU	A	176	53.045	34.137	35.244	1.00	0.00	O
ATOM	2452	C	GLU	A	176	56.259	31.161	31.951	1.00	0.00	C
ATOM	2453	O	GLU	A	176	56.185	31.478	30.760	1.00	0.00	O
ATOM	2454	N	ARG	A	177	56.052	29.909	32.372	1.00	0.00	N
ATOM	2455	H	ARG	A	177	56.109	29.728	33.368	1.00	0.00	H
ATOM	2456	CA	ARG	A	177	55.877	28.750	31.487	1.00	0.00	C
ATOM	2457	HA	ARG	A	177	56.726	28.730	30.806	1.00	0.00	H
ATOM	2458	CB	ARG	A	177	55.902	27.461	32.335	1.00	0.00	C
ATOM	2459	HB1	ARG	A	177	56.799	27.461	32.957	1.00	0.00	H
ATOM	2460	HB2	ARG	A	177	55.032	27.448	32.991	1.00	0.00	H
ATOM	2461	CG	ARG	A	177	55.903	26.187	31.476	1.00	0.00	C
ATOM	2462	HG1	ARG	A	177	55.035	26.188	30.829	1.00	0.00	H
ATOM	2463	HG2	ARG	A	177	56.789	26.193	30.844	1.00	0.00	H
ATOM	2464	CD	ARG	A	177	55.895	24.880	32.279	1.00	0.00	C
ATOM	2465	HD1	ARG	A	177	55.941	24.047	31.577	1.00	0.00	H
ATOM	2466	HD2	ARG	A	177	56.796	24.843	32.895	1.00	0.00	H
ATOM	2467	NE	ARG	A	177	54.720	24.711	33.153	1.00	0.00	N
ATOM	2468	HE	ARG	A	177	54.912	24.602	34.140	1.00	0.00	H
ATOM	2469	CZ	ARG	A	177	53.456	24.481	32.806	1.00	0.00	C
ATOM	2470	NH1	ARG	A	177	52.987	24.526	31.580	1.00	0.00	N
ATOM	2471	HH11	ARG	A	177	53.542	24.846	30.798	1.00	0.00	H
ATOM	2472	HH12	ARG	A	177	52.023	24.246	31.418	1.00	0.00	H
ATOM	2473	NH2	ARG	A	177	52.600	24.179	33.745	1.00	0.00	N
ATOM	2474	HH21	ARG	A	177	52.915	24.065	34.699	1.00	0.00	H
ATOM	2475	HH22	ARG	A	177	51.625	24.012	33.513	1.00	0.00	H
ATOM	2476	C	ARG	A	177	54.598	28.840	30.640	1.00	0.00	C
ATOM	2477	O	ARG	A	177	53.530	29.168	31.167	1.00	0.00	O
ATOM	2478	N	LEU	A	178	54.699	28.485	29.353	1.00	0.00	N
ATOM	2479	H	LEU	A	178	55.624	28.254	29.005	1.00	0.00	H
ATOM	2480	CA	LEU	A	178	53.570	28.287	28.435	1.00	0.00	C
ATOM	2481	HA	LEU	A	178	52.946	29.179	28.450	1.00	0.00	H

ATOM	2482	CB	LEU	A	178	54.099	28.053	27.003	1.00	0.00	C
ATOM	2483	HB1	LEU	A	178	54.790	27.208	27.031	1.00	0.00	H
ATOM	2484	HB2	LEU	A	178	53.263	27.766	26.363	1.00	0.00	H
ATOM	2485	CG	LEU	A	178	54.817	29.257	26.357	1.00	0.00	C
ATOM	2486	HG	LEU	A	178	55.590	29.618	27.034	1.00	0.00	H
ATOM	2487	CD1	LEU	A	178	55.503	28.814	25.056	1.00	0.00	C
ATOM	2488	HD11	LEU	A	178	56.015	29.661	24.599	1.00	0.00	H
ATOM	2489	HD12	LEU	A	178	56.241	28.041	25.274	1.00	0.00	H
ATOM	2490	HD13	LEU	A	178	54.769	28.418	24.356	1.00	0.00	H
ATOM	2491	CD2	LEU	A	178	53.846	30.411	26.065	1.00	0.00	C
ATOM	2492	HD21	LEU	A	178	54.371	31.215	25.550	1.00	0.00	H
ATOM	2493	HD22	LEU	A	178	53.024	30.064	25.437	1.00	0.00	H
ATOM	2494	HD23	LEU	A	178	53.442	30.808	26.996	1.00	0.00	H
ATOM	2495	C	LEU	A	178	52.701	27.096	28.872	1.00	0.00	C
ATOM	2496	O	LEU	A	178	53.222	26.027	29.195	1.00	0.00	O
ATOM	2497	N	LEU	A	179	51.380	27.276	28.868	1.00	0.00	N
ATOM	2498	H	LEU	A	179	51.030	28.187	28.592	1.00	0.00	H
ATOM	2499	CA	LEU	A	179	50.391	26.266	29.260	1.00	0.00	C
ATOM	2500	HA	LEU	A	179	50.835	25.602	30.001	1.00	0.00	H
ATOM	2501	CB	LEU	A	179	49.167	26.961	29.888	1.00	0.00	C
ATOM	2502	HB1	LEU	A	179	48.651	27.523	29.108	1.00	0.00	H
ATOM	2503	HB2	LEU	A	179	48.483	26.188	30.243	1.00	0.00	H
ATOM	2504	CG	LEU	A	179	49.465	27.925	31.055	1.00	0.00	C
ATOM	2505	HG	LEU	A	179	50.019	28.786	30.679	1.00	0.00	H
ATOM	2506	CD1	LEU	A	179	48.131	28.428	31.622	1.00	0.00	C
ATOM	2507	HD11	LEU	A	179	48.318	29.099	32.458	1.00	0.00	H
ATOM	2508	HD12	LEU	A	179	47.578	28.967	30.851	1.00	0.00	H
ATOM	2509	HD13	LEU	A	179	47.532	27.587	31.972	1.00	0.00	H
ATOM	2510	CD2	LEU	A	179	50.293	27.274	32.170	1.00	0.00	C
ATOM	2511	HD21	LEU	A	179	50.431	27.986	32.982	1.00	0.00	H
ATOM	2512	HD22	LEU	A	179	49.778	26.395	32.549	1.00	0.00	H
ATOM	2513	HD23	LEU	A	179	51.278	26.991	31.799	1.00	0.00	H
ATOM	2514	C	LEU	A	179	49.955	25.393	28.076	1.00	0.00	C
ATOM	2515	O	LEU	A	179	50.038	25.814	26.921	1.00	0.00	O
ATOM	2516	N	LEU	A	180	49.433	24.200	28.371	1.00	0.00	N
ATOM	2517	H	LEU	A	180	49.419	23.905	29.340	1.00	0.00	H
ATOM	2518	CA	LEU	A	180	48.880	23.279	27.374	1.00	0.00	C
ATOM	2519	HA	LEU	A	180	48.770	23.800	26.423	1.00	0.00	H
ATOM	2520	CB	LEU	A	180	49.879	22.118	27.201	1.00	0.00	C
ATOM	2521	HB1	LEU	A	180	50.859	22.526	26.952	1.00	0.00	H
ATOM	2522	HB2	LEU	A	180	49.970	21.599	28.158	1.00	0.00	H
ATOM	2523	CG	LEU	A	180	49.480	21.096	26.120	1.00	0.00	C
ATOM	2524	HG	LEU	A	180	48.473	20.733	26.317	1.00	0.00	H
ATOM	2525	CD1	LEU	A	180	49.497	21.697	24.708	1.00	0.00	C
ATOM	2526	HD11	LEU	A	180	49.233	20.927	23.985	1.00	0.00	H
ATOM	2527	HD12	LEU	A	180	48.761	22.495	24.627	1.00	0.00	H
ATOM	2528	HD13	LEU	A	180	50.487	22.089	24.474	1.00	0.00	H
ATOM	2529	CD2	LEU	A	180	50.442	19.903	26.199	1.00	0.00	C
ATOM	2530	HD21	LEU	A	180	50.192	19.176	25.427	1.00	0.00	H
ATOM	2531	HD22	LEU	A	180	51.468	20.242	26.065	1.00	0.00	H
ATOM	2532	HD23	LEU	A	180	50.359	19.423	27.174	1.00	0.00	H
ATOM	2533	C	LEU	A	180	47.495	22.773	27.798	1.00	0.00	C
ATOM	2534	O	LEU	A	180	47.361	22.144	28.846	1.00	0.00	O
ATOM	2535	N	SER	A	181	46.476	22.989	26.974	1.00	0.00	N

```
ATOM   2536  H   SER A 181      46.628  23.537  26.127  1.00  0.00           H
ATOM   2537  CA  SER A 181      45.123  22.456  27.176  1.00  0.00           C
ATOM   2538  HA  SER A 181      45.150  21.718  27.976  1.00  0.00           H
ATOM   2539  CB  SER A 181      44.172  23.582  27.630  1.00  0.00           C
ATOM   2540  HB1 SER A 181      43.195  23.161  27.872  1.00  0.00           H
ATOM   2541  HB2 SER A 181      44.576  24.028  28.540  1.00  0.00           H
ATOM   2542  OG  SER A 181      44.000  24.604  26.665  1.00  0.00           O
ATOM   2543  HG  SER A 181      43.393  24.283  25.958  1.00  0.00           H
ATOM   2544  C   SER A 181      44.596  21.707  25.938  1.00  0.00           C
ATOM   2545  O   SER A 181      45.268  21.614  24.907  1.00  0.00           O
ATOM   2546  N   ALA A 182      43.384  21.148  26.023  1.00  0.00           N
ATOM   2547  H   ALA A 182      42.855  21.244  26.878  1.00  0.00           H
ATOM   2548  CA  ALA A 182      42.728  20.494  24.893  1.00  0.00           C
ATOM   2549  HA  ALA A 182      43.035  21.000  23.977  1.00  0.00           H
ATOM   2550  CB  ALA A 182      43.191  19.030  24.827  1.00  0.00           C
ATOM   2551  HB1 ALA A 182      42.733  18.538  23.968  1.00  0.00           H
ATOM   2552  HB2 ALA A 182      44.277  18.989  24.727  1.00  0.00           H
ATOM   2553  HB3 ALA A 182      42.897  18.507  25.738  1.00  0.00           H
ATOM   2554  C   ALA A 182      41.200  20.593  24.972  1.00  0.00           C
ATOM   2555  O   ALA A 182      40.604  20.368  26.027  1.00  0.00           O
ATOM   2556  N   ALA A 183      40.565  20.843  23.825  1.00  0.00           N
ATOM   2557  H   ALA A 183      41.127  21.071  23.014  1.00  0.00           H
ATOM   2558  CA  ALA A 183      39.128  20.694  23.629  1.00  0.00           C
ATOM   2559  HA  ALA A 183      38.601  21.074  24.503  1.00  0.00           H
ATOM   2560  CB  ALA A 183      38.725  21.551  22.423  1.00  0.00           C
ATOM   2561  HB1 ALA A 183      37.658  21.439  22.231  1.00  0.00           H
ATOM   2562  HB2 ALA A 183      38.944  22.600  22.626  1.00  0.00           H
ATOM   2563  HB3 ALA A 183      39.279  21.245  21.536  1.00  0.00           H
ATOM   2564  C   ALA A 183      38.785  19.205  23.441  1.00  0.00           C
ATOM   2565  O   ALA A 183      39.015  18.650  22.363  1.00  0.00           O
ATOM   2566  N   VAL A 184      38.251  18.548  24.473  1.00  0.00           N
ATOM   2567  H   VAL A 184      38.071  19.090  25.320  1.00  0.00           H
ATOM   2568  CA  VAL A 184      38.041  17.081  24.505  1.00  0.00           C
ATOM   2569  HA  VAL A 184      38.571  16.664  23.650  1.00  0.00           H
ATOM   2570  CB  VAL A 184      38.668  16.427  25.756  1.00  0.00           C
ATOM   2571  HB  VAL A 184      38.530  15.357  25.639  1.00  0.00           H
ATOM   2572  CG1 VAL A 184      40.183  16.686  25.816  1.00  0.00           C
ATOM   2573 HG11 VAL A 184      40.629  16.100  26.620  1.00  0.00           H
ATOM   2574 HG12 VAL A 184      40.648  16.397  24.874  1.00  0.00           H
ATOM   2575 HG13 VAL A 184      40.382  17.740  26.002  1.00  0.00           H
ATOM   2576  CG2 VAL A 184      38.001  16.853  27.072  1.00  0.00           C
ATOM   2577 HG21 VAL A 184      38.458  16.317  27.902  1.00  0.00           H
ATOM   2578 HG22 VAL A 184      38.121  17.923  27.230  1.00  0.00           H
ATOM   2579 HG23 VAL A 184      36.942  16.602  27.060  1.00  0.00           H
ATOM   2580  C   VAL A 184      36.556  16.674  24.383  1.00  0.00           C
ATOM   2581  O   VAL A 184      35.694  17.429  24.842  1.00  0.00           O
ATOM   2582  N   PRO A 185      36.236  15.519  23.754  1.00  0.00           N
ATOM   2583  CD  PRO A 185      37.170  14.609  23.101  1.00  0.00           C
ATOM   2584  HD1 PRO A 185      37.937  14.257  23.791  1.00  0.00           H
ATOM   2585  HD2 PRO A 185      37.632  15.116  22.254  1.00  0.00           H
ATOM   2586  CG  PRO A 185      36.344  13.426  22.597  1.00  0.00           C
ATOM   2587  HG1 PRO A 185      36.296  12.658  23.369  1.00  0.00           H
ATOM   2588  HG2 PRO A 185      36.751  13.015  21.674  1.00  0.00           H
ATOM   2589  CB  PRO A 185      34.961  14.034  22.382  1.00  0.00           C
```

```
ATOM   2590  HB1 PRO A 185      34.174  13.281  22.443  1.00  0.00           H
ATOM   2591  HB2 PRO A 185      34.923  14.537  21.418  1.00  0.00           H
ATOM   2592  CA  PRO A 185      34.865  15.072  23.503  1.00  0.00           C
ATOM   2593  HA  PRO A 185      34.257  15.909  23.162  1.00  0.00           H
ATOM   2594  C   PRO A 185      34.199  14.463  24.739  1.00  0.00           C
ATOM   2595  O   PRO A 185      34.863  13.988  25.655  1.00  0.00           O
ATOM   2596  N   ALA A 186      32.865  14.460  24.734  1.00  0.00           N
ATOM   2597  H   ALA A 186      32.394  14.904  23.949  1.00  0.00           H
ATOM   2598  CA  ALA A 186      32.027  14.043  25.856  1.00  0.00           C
ATOM   2599  HA  ALA A 186      32.615  14.069  26.775  1.00  0.00           H
ATOM   2600  CB  ALA A 186      30.921  15.100  25.957  1.00  0.00           C
ATOM   2601  HB1 ALA A 186      30.277  14.875  26.798  1.00  0.00           H
ATOM   2602  HB2 ALA A 186      31.363  16.085  26.105  1.00  0.00           H
ATOM   2603  HB3 ALA A 186      30.318  15.106  25.047  1.00  0.00           H
ATOM   2604  C   ALA A 186      31.445  12.615  25.743  1.00  0.00           C
ATOM   2605  O   ALA A 186      30.946  12.088  26.742  1.00  0.00           O
ATOM   2606  N   GLY A 187      31.489  11.989  24.560  1.00  0.00           N
ATOM   2607  H   GLY A 187      31.882  12.490  23.772  1.00  0.00           H
ATOM   2608  CA  GLY A 187      30.899  10.673  24.272  1.00  0.00           C
ATOM   2609  HA1 GLY A 187      30.131  10.436  25.008  1.00  0.00           H
ATOM   2610  HA2 GLY A 187      30.430  10.703  23.290  1.00  0.00           H
ATOM   2611  C   GLY A 187      31.916   9.531  24.228  1.00  0.00           C
ATOM   2612  O   GLY A 187      33.034   9.678  23.722  1.00  0.00           O
ATOM   2613  N   GLN A 188      31.512   8.367  24.741  1.00  0.00           N
ATOM   2614  H   GLN A 188      30.568   8.316  25.110  1.00  0.00           H
ATOM   2615  CA  GLN A 188      32.357   7.176  24.855  1.00  0.00           C
ATOM   2616  HA  GLN A 188      33.212   7.437  25.482  1.00  0.00           H
ATOM   2617  CB  GLN A 188      31.605   6.054  25.591  1.00  0.00           C
ATOM   2618  HB1 GLN A 188      32.312   5.250  25.802  1.00  0.00           H
ATOM   2619  HB2 GLN A 188      31.263   6.441  26.551  1.00  0.00           H
ATOM   2620  CG  GLN A 188      30.405   5.449  24.837  1.00  0.00           C
ATOM   2621  HG1 GLN A 188      29.742   6.244  24.498  1.00  0.00           H
ATOM   2622  HG2 GLN A 188      30.754   4.895  23.966  1.00  0.00           H
ATOM   2623  CD  GLN A 188      29.633   4.500  25.750  1.00  0.00           C
ATOM   2624  OE1 GLN A 188      30.063   3.393  26.056  1.00  0.00           O
ATOM   2625  NE2 GLN A 188      28.503   4.902  26.284  1.00  0.00           N
ATOM   2626 HE21 GLN A 188      28.081   5.775  25.992  1.00  0.00           H
ATOM   2627 HE22 GLN A 188      28.116   4.340  27.032  1.00  0.00           H
ATOM   2628  C   GLN A 188      32.948   6.681  23.526  1.00  0.00           C
ATOM   2629  O   GLN A 188      34.031   6.099  23.567  1.00  0.00           O
ATOM   2630  N   THR A 189      32.318   6.917  22.360  1.00  0.00           N
ATOM   2631  H   THR A 189      31.415   7.389  22.366  1.00  0.00           H
ATOM   2632  CA  THR A 189      32.896   6.535  21.057  1.00  0.00           C
ATOM   2633  HA  THR A 189      33.151   5.480  21.082  1.00  0.00           H
ATOM   2634  CB  THR A 189      31.911   6.732  19.901  1.00  0.00           C
ATOM   2635  HB  THR A 189      31.672   7.789  19.799  1.00  0.00           H
ATOM   2636  CG2 THR A 189      32.477   6.217  18.577  1.00  0.00           C
ATOM   2637 HG21 THR A 189      31.722   6.294  17.795  1.00  0.00           H
ATOM   2638 HG22 THR A 189      33.337   6.817  18.282  1.00  0.00           H
ATOM   2639 HG23 THR A 189      32.781   5.174  18.676  1.00  0.00           H
ATOM   2640  OG1 THR A 189      30.721   6.021  20.147  1.00  0.00           O
ATOM   2641  HG1 THR A 189      30.942   5.158  20.549  1.00  0.00           H
ATOM   2642  C   THR A 189      34.200   7.285  20.820  1.00  0.00           C
ATOM   2643  O   THR A 189      35.256   6.655  20.766  1.00  0.00           O
```

ATOM	2644	N	TYR	A	190	34.157	8.620	20.731	1.00	0.00	N
ATOM	2645	H	TYR	A	190	33.255	9.086	20.777	1.00	0.00	H
ATOM	2646	CA	TYR	A	190	35.363	9.419	20.478	1.00	0.00	C
ATOM	2647	HA	TYR	A	190	35.869	8.997	19.610	1.00	0.00	H
ATOM	2648	CB	TYR	A	190	35.008	10.885	20.164	1.00	0.00	C
ATOM	2649	HB1	TYR	A	190	34.509	11.299	21.041	1.00	0.00	H
ATOM	2650	HB2	TYR	A	190	35.942	11.433	20.032	1.00	0.00	H
ATOM	2651	CG	TYR	A	190	34.151	11.181	18.939	1.00	0.00	C
ATOM	2652	CD1	TYR	A	190	34.177	10.364	17.789	1.00	0.00	C
ATOM	2653	HD1	TYR	A	190	34.811	9.492	17.754	1.00	0.00	H
ATOM	2654	CE1	TYR	A	190	33.368	10.668	16.675	1.00	0.00	C
ATOM	2655	HE1	TYR	A	190	33.400	10.033	15.801	1.00	0.00	H
ATOM	2656	CZ	TYR	A	190	32.509	11.790	16.706	1.00	0.00	C
ATOM	2657	OH	TYR	A	190	31.706	12.088	15.644	1.00	0.00	O
ATOM	2658	HH	TYR	A	190	31.796	11.442	14.921	1.00	0.00	H
ATOM	2659	CE2	TYR	A	190	32.483	12.609	17.855	1.00	0.00	C
ATOM	2660	HE2	TYR	A	190	31.841	13.478	17.880	1.00	0.00	H
ATOM	2661	CD2	TYR	A	190	33.309	12.312	18.955	1.00	0.00	C
ATOM	2662	HD2	TYR	A	190	33.285	12.960	19.822	1.00	0.00	H
ATOM	2663	C	TYR	A	190	36.369	9.359	21.643	1.00	0.00	C
ATOM	2664	O	TYR	A	190	37.576	9.376	21.385	1.00	0.00	O
ATOM	2665	N	VAL	A	191	35.899	9.262	22.895	1.00	0.00	N
ATOM	2666	H	VAL	A	191	34.886	9.272	23.020	1.00	0.00	H
ATOM	2667	CA	VAL	A	191	36.770	9.173	24.086	1.00	0.00	C
ATOM	2668	HA	VAL	A	191	37.489	9.993	24.024	1.00	0.00	H
ATOM	2669	CB	VAL	A	191	35.981	9.349	25.409	1.00	0.00	C
ATOM	2670	HB	VAL	A	191	35.136	8.660	25.401	1.00	0.00	H
ATOM	2671	CG1	VAL	A	191	36.842	9.061	26.657	1.00	0.00	C
ATOM	2672	HG11	VAL	A	191	36.295	9.326	27.562	1.00	0.00	H
ATOM	2673	HG12	VAL	A	191	37.091	8.001	26.712	1.00	0.00	H
ATOM	2674	HG13	VAL	A	191	37.761	9.647	26.620	1.00	0.00	H
ATOM	2675	CG2	VAL	A	191	35.448	10.787	25.532	1.00	0.00	C
ATOM	2676	HG21	VAL	A	191	34.799	10.870	26.405	1.00	0.00	H
ATOM	2677	HG22	VAL	A	191	36.280	11.479	25.638	1.00	0.00	H
ATOM	2678	HG23	VAL	A	191	34.881	11.073	24.650	1.00	0.00	H
ATOM	2679	C	VAL	A	191	37.565	7.860	24.099	1.00	0.00	C
ATOM	2680	O	VAL	A	191	38.795	7.901	24.160	1.00	0.00	O
ATOM	2681	N	ASP	A	192	36.905	6.696	24.006	1.00	0.00	N
ATOM	2682	H	ASP	A	192	35.890	6.687	23.910	1.00	0.00	H
ATOM	2683	CA	ASP	A	192	37.594	5.397	24.058	1.00	0.00	C
ATOM	2684	HA	ASP	A	192	38.319	5.426	24.867	1.00	0.00	H
ATOM	2685	CB	ASP	A	192	36.615	4.260	24.407	1.00	0.00	C
ATOM	2686	HB1	ASP	A	192	35.788	4.267	23.702	1.00	0.00	H
ATOM	2687	HB2	ASP	A	192	37.134	3.309	24.286	1.00	0.00	H
ATOM	2688	CG	ASP	A	192	36.099	4.318	25.853	1.00	0.00	C
ATOM	2689	OD1	ASP	A	192	36.920	4.520	26.782	1.00	0.00	O
ATOM	2690	OD2	ASP	A	192	34.887	4.104	26.092	1.00	0.00	O
ATOM	2691	C	ASP	A	192	38.393	5.094	22.775	1.00	0.00	C
ATOM	2692	O	ASP	A	192	39.276	4.236	22.798	1.00	0.00	O
ATOM	2693	N	ALA	A	193	38.135	5.799	21.665	1.00	0.00	N
ATOM	2694	H	ALA	A	193	37.344	6.433	21.680	1.00	0.00	H
ATOM	2695	CA	ALA	A	193	38.911	5.682	20.423	1.00	0.00	C
ATOM	2696	HA	ALA	A	193	39.291	4.663	20.330	1.00	0.00	H
ATOM	2697	CB	ALA	A	193	37.972	5.937	19.237	1.00	0.00	C

ATOM	2698	HB1	ALA	A	193	38.526	5.854	18.301	1.00	0.00	H
ATOM	2699	HB2	ALA	A	193	37.175	5.195	19.227	1.00	0.00	H
ATOM	2700	HB3	ALA	A	193	37.535	6.933	19.302	1.00	0.00	H
ATOM	2701	C	ALA	A	193	40.135	6.615	20.343	1.00	0.00	C
ATOM	2702	O	ALA	A	193	41.057	6.310	19.586	1.00	0.00	O
ATOM	2703	N	GLY	A	194	40.164	7.738	21.077	1.00	0.00	N
ATOM	2704	H	GLY	A	194	39.372	7.932	21.676	1.00	0.00	H
ATOM	2705	CA	GLY	A	194	41.125	8.824	20.812	1.00	0.00	C
ATOM	2706	HA1	GLY	A	194	41.765	8.576	19.965	1.00	0.00	H
ATOM	2707	HA2	GLY	A	194	40.544	9.706	20.549	1.00	0.00	H
ATOM	2708	C	GLY	A	194	42.033	9.268	21.954	1.00	0.00	C
ATOM	2709	O	GLY	A	194	43.179	9.630	21.698	1.00	0.00	O
ATOM	2710	N	TYR	A	195	41.540	9.284	23.194	1.00	0.00	N
ATOM	2711	H	TYR	A	195	40.607	8.927	23.354	1.00	0.00	H
ATOM	2712	CA	TYR	A	195	42.173	10.041	24.285	1.00	0.00	C
ATOM	2713	HA	TYR	A	195	43.067	10.549	23.921	1.00	0.00	H
ATOM	2714	CB	TYR	A	195	41.199	11.134	24.758	1.00	0.00	C
ATOM	2715	HB1	TYR	A	195	40.251	10.674	25.046	1.00	0.00	H
ATOM	2716	HB2	TYR	A	195	41.617	11.598	25.652	1.00	0.00	H
ATOM	2717	CG	TYR	A	195	40.928	12.223	23.728	1.00	0.00	C
ATOM	2718	CD1	TYR	A	195	39.984	12.017	22.700	1.00	0.00	C
ATOM	2719	HD1	TYR	A	195	39.437	11.086	22.650	1.00	0.00	H
ATOM	2720	CE1	TYR	A	195	39.757	13.014	21.732	1.00	0.00	C
ATOM	2721	HE1	TYR	A	195	39.046	12.843	20.938	1.00	0.00	H
ATOM	2722	CZ	TYR	A	195	40.445	14.246	21.809	1.00	0.00	C
ATOM	2723	OH	TYR	A	195	40.190	15.228	20.899	1.00	0.00	O
ATOM	2724	HH	TYR	A	195	40.719	16.021	21.054	1.00	0.00	H
ATOM	2725	CE2	TYR	A	195	41.383	14.457	22.843	1.00	0.00	C
ATOM	2726	HE2	TYR	A	195	41.913	15.396	22.912	1.00	0.00	H
ATOM	2727	CD2	TYR	A	195	41.627	13.445	23.791	1.00	0.00	C
ATOM	2728	HD2	TYR	A	195	42.352	13.610	24.576	1.00	0.00	H
ATOM	2729	C	TYR	A	195	42.599	9.133	25.449	1.00	0.00	C
ATOM	2730	O	TYR	A	195	41.759	8.521	26.107	1.00	0.00	O
ATOM	2731	N	GLU	A	196	43.900	9.043	25.730	1.00	0.00	N
ATOM	2732	H	GLU	A	196	44.559	9.554	25.148	1.00	0.00	H
ATOM	2733	CA	GLU	A	196	44.425	8.304	26.883	1.00	0.00	C
ATOM	2734	HA	GLU	A	196	43.783	7.445	27.063	1.00	0.00	H
ATOM	2735	CB	GLU	A	196	45.821	7.741	26.606	1.00	0.00	C
ATOM	2736	HB1	GLU	A	196	46.518	8.558	26.435	1.00	0.00	H
ATOM	2737	HB2	GLU	A	196	46.146	7.192	27.488	1.00	0.00	H
ATOM	2738	CG	GLU	A	196	45.816	6.786	25.404	1.00	0.00	C
ATOM	2739	HG1	GLU	A	196	44.988	6.088	25.495	1.00	0.00	H
ATOM	2740	HG2	GLU	A	196	45.688	7.354	24.481	1.00	0.00	H
ATOM	2741	CD	GLU	A	196	47.116	6.003	25.337	1.00	0.00	C
ATOM	2742	OE1	GLU	A	196	48.128	6.593	24.907	1.00	0.00	O
ATOM	2743	OE2	GLU	A	196	47.151	4.833	25.787	1.00	0.00	O
ATOM	2744	C	GLU	A	196	44.378	9.202	28.126	1.00	0.00	C
ATOM	2745	O	GLU	A	196	45.350	9.876	28.470	1.00	0.00	O
ATOM	2746	N	VAL	A	197	43.198	9.244	28.746	1.00	0.00	N
ATOM	2747	H	VAL	A	197	42.517	8.540	28.459	1.00	0.00	H
ATOM	2748	CA	VAL	A	197	42.741	10.279	29.684	1.00	0.00	C
ATOM	2749	HA	VAL	A	197	42.724	11.225	29.141	1.00	0.00	H
ATOM	2750	CB	VAL	A	197	41.298	9.982	30.164	1.00	0.00	C
ATOM	2751	HB	VAL	A	197	41.296	9.014	30.661	1.00	0.00	H

```
ATOM   2752  CG1 VAL A 197      40.784  11.050  31.144  1.00  0.00           C
ATOM   2753 HG11 VAL A 197      39.750  10.845  31.422  1.00  0.00           H
ATOM   2754 HG12 VAL A 197      41.383  11.059  32.054  1.00  0.00           H
ATOM   2755 HG13 VAL A 197      40.840  12.029  30.673  1.00  0.00           H
ATOM   2756  CG2 VAL A 197      40.310   9.891  28.986  1.00  0.00           C
ATOM   2757 HG21 VAL A 197      39.292   9.773  29.358  1.00  0.00           H
ATOM   2758 HG22 VAL A 197      40.366  10.793  28.375  1.00  0.00           H
ATOM   2759 HG23 VAL A 197      40.541   9.024  28.370  1.00  0.00           H
ATOM   2760  C   VAL A 197      43.683  10.430  30.884  1.00  0.00           C
ATOM   2761  O   VAL A 197      44.067  11.551  31.211  1.00  0.00           O
ATOM   2762  N   ASP A 198      44.086   9.323  31.513  1.00  0.00           N
ATOM   2763  H   ASP A 198      43.745   8.433  31.178  1.00  0.00           H
ATOM   2764  CA  ASP A 198      44.968   9.316  32.688  1.00  0.00           C
ATOM   2765  HA  ASP A 198      44.660  10.110  33.372  1.00  0.00           H
ATOM   2766  CB  ASP A 198      44.791   7.977  33.410  1.00  0.00           C
ATOM   2767  HB1 ASP A 198      43.742   7.846  33.665  1.00  0.00           H
ATOM   2768  HB2 ASP A 198      45.079   7.169  32.736  1.00  0.00           H
ATOM   2769  CG  ASP A 198      45.604   7.875  34.699  1.00  0.00           C
ATOM   2770  OD1 ASP A 198      45.162   8.409  35.741  1.00  0.00           O
ATOM   2771  OD2 ASP A 198      46.629   7.154  34.685  1.00  0.00           O
ATOM   2772  C   ASP A 198      46.444   9.548  32.322  1.00  0.00           C
ATOM   2773  O   ASP A 198      47.206  10.071  33.133  1.00  0.00           O
ATOM   2774  N   LYS A 199      46.863   9.212  31.094  1.00  0.00           N
ATOM   2775  H   LYS A 199      46.181   8.847  30.437  1.00  0.00           H
ATOM   2776  CA  LYS A 199      48.203   9.563  30.610  1.00  0.00           C
ATOM   2777  HA  LYS A 199      48.926   9.348  31.394  1.00  0.00           H
ATOM   2778  CB  LYS A 199      48.580   8.738  29.369  1.00  0.00           C
ATOM   2779  HB1 LYS A 199      47.824   8.885  28.598  1.00  0.00           H
ATOM   2780  HB2 LYS A 199      49.527   9.130  28.995  1.00  0.00           H
ATOM   2781  CG  LYS A 199      48.766   7.230  29.621  1.00  0.00           C
ATOM   2782  HG1 LYS A 199      49.518   7.077  30.395  1.00  0.00           H
ATOM   2783  HG2 LYS A 199      47.819   6.789  29.936  1.00  0.00           H
ATOM   2784  CD  LYS A 199      49.238   6.561  28.324  1.00  0.00           C
ATOM   2785  HD1 LYS A 199      48.510   6.787  27.548  1.00  0.00           H
ATOM   2786  HD2 LYS A 199      50.201   6.989  28.037  1.00  0.00           H
ATOM   2787  CE  LYS A 199      49.401   5.042  28.409  1.00  0.00           C
ATOM   2788  HE1 LYS A 199      50.243   4.814  29.069  1.00  0.00           H
ATOM   2789  HE2 LYS A 199      48.496   4.603  28.839  1.00  0.00           H
ATOM   2790  NZ  LYS A 199      49.643   4.485  27.056  1.00  0.00           N
ATOM   2791  HZ1 LYS A 199      48.807   4.571  26.477  1.00  0.00           H
ATOM   2792  HZ2 LYS A 199      50.376   5.010  26.584  1.00  0.00           H
ATOM   2793  HZ3 LYS A 199      49.933   3.517  27.078  1.00  0.00           H
ATOM   2794  C   LYS A 199      48.328  11.071  30.332  1.00  0.00           C
ATOM   2795  O   LYS A 199      49.291  11.693  30.791  1.00  0.00           O
ATOM   2796  N   ILE A 200      47.360  11.676  29.628  1.00  0.00           N
ATOM   2797  H   ILE A 200      46.601  11.095  29.268  1.00  0.00           H
ATOM   2798  CA  ILE A 200      47.384  13.122  29.310  1.00  0.00           C
ATOM   2799  HA  ILE A 200      48.405  13.384  29.019  1.00  0.00           H
ATOM   2800  CB  ILE A 200      46.475  13.471  28.104  1.00  0.00           C
ATOM   2801  HB  ILE A 200      46.618  14.531  27.892  1.00  0.00           H
ATOM   2802  CG2 ILE A 200      46.955  12.703  26.857  1.00  0.00           C
ATOM   2803 HG21 ILE A 200      46.479  13.099  25.961  1.00  0.00           H
ATOM   2804 HG22 ILE A 200      48.032  12.824  26.747  1.00  0.00           H
ATOM   2805 HG23 ILE A 200      46.722  11.642  26.944  1.00  0.00           H
```

```
ATOM   2806  CG1 ILE A 200      44.968  13.282  28.395  1.00  0.00           C
ATOM   2807 HG11 ILE A 200      44.794  12.280  28.771  1.00  0.00           H
ATOM   2808 HG12 ILE A 200      44.674  13.985  29.174  1.00  0.00           H
ATOM   2809  CD  ILE A 200      44.043  13.505  27.191  1.00  0.00           C
ATOM   2810  HD1 ILE A 200      43.006  13.464  27.525  1.00  0.00           H
ATOM   2811  HD2 ILE A 200      44.236  14.481  26.746  1.00  0.00           H
ATOM   2812  HD3 ILE A 200      44.196  12.724  26.447  1.00  0.00           H
ATOM   2813  C   ILE A 200      47.091  14.004  30.534  1.00  0.00           C
ATOM   2814  O   ILE A 200      47.651  15.093  30.634  1.00  0.00           O
ATOM   2815  N   ALA A 201      46.301  13.510  31.496  1.00  0.00           N
ATOM   2816  H   ALA A 201      45.848  12.622  31.319  1.00  0.00           H
ATOM   2817  CA  ALA A 201      45.932  14.192  32.745  1.00  0.00           C
ATOM   2818  HA  ALA A 201      45.246  15.012  32.524  1.00  0.00           H
ATOM   2819  CB  ALA A 201      45.224  13.163  33.636  1.00  0.00           C
ATOM   2820  HB1 ALA A 201      45.185  13.528  34.658  1.00  0.00           H
ATOM   2821  HB2 ALA A 201      44.208  12.985  33.290  1.00  0.00           H
ATOM   2822  HB3 ALA A 201      45.774  12.223  33.652  1.00  0.00           H
ATOM   2823  C   ALA A 201      47.117  14.776  33.526  1.00  0.00           C
ATOM   2824  O   ALA A 201      46.995  15.828  34.158  1.00  0.00           O
ATOM   2825  N   GLN A 202      48.258  14.088  33.505  1.00  0.00           N
ATOM   2826  H   GLN A 202      48.285  13.237  32.964  1.00  0.00           H
ATOM   2827  CA  GLN A 202      49.447  14.496  34.250  1.00  0.00           C
ATOM   2828  HA  GLN A 202      49.152  14.794  35.258  1.00  0.00           H
ATOM   2829  CB  GLN A 202      50.431  13.315  34.358  1.00  0.00           C
ATOM   2830  HB1 GLN A 202      50.974  13.221  33.416  1.00  0.00           H
ATOM   2831  HB2 GLN A 202      51.162  13.550  35.133  1.00  0.00           H
ATOM   2832  CG  GLN A 202      49.802  11.943  34.666  1.00  0.00           C
ATOM   2833  HG1 GLN A 202      49.283  11.592  33.775  1.00  0.00           H
ATOM   2834  HG2 GLN A 202      50.606  11.237  34.859  1.00  0.00           H
ATOM   2835  CD  GLN A 202      48.836  11.897  35.855  1.00  0.00           C
ATOM   2836  OE1 GLN A 202      48.944  12.641  36.828  1.00  0.00           O
ATOM   2837  NE2 GLN A 202      47.847  11.035  35.799  1.00  0.00           N
ATOM   2838 HE21 GLN A 202      47.719  10.452  34.979  1.00  0.00           H
ATOM   2839 HE22 GLN A 202      47.304  10.842  36.631  1.00  0.00           H
ATOM   2840  C   GLN A 202      50.136  15.701  33.592  1.00  0.00           C
ATOM   2841  O   GLN A 202      50.669  16.553  34.300  1.00  0.00           O
ATOM   2842  N   ASN A 203      50.098  15.770  32.255  1.00  0.00           N
ATOM   2843  H   ASN A 203      49.519  15.096  31.770  1.00  0.00           H
ATOM   2844  CA  ASN A 203      50.911  16.657  31.415  1.00  0.00           C
ATOM   2845  HA  ASN A 203      51.768  17.019  31.987  1.00  0.00           H
ATOM   2846  CB  ASN A 203      51.420  15.819  30.228  1.00  0.00           C
ATOM   2847  HB1 ASN A 203      50.556  15.389  29.723  1.00  0.00           H
ATOM   2848  HB2 ASN A 203      51.936  16.465  29.520  1.00  0.00           H
ATOM   2849  CG  ASN A 203      52.373  14.687  30.611  1.00  0.00           C
ATOM   2850  OD1 ASN A 203      53.171  14.779  31.535  1.00  0.00           O
ATOM   2851  ND2 ASN A 203      52.334  13.578  29.896  1.00  0.00           N
ATOM   2852 HD21 ASN A 203      51.666  13.466  29.140  1.00  0.00           H
ATOM   2853 HD22 ASN A 203      52.945  12.818  30.155  1.00  0.00           H
ATOM   2854  C   ASN A 203      50.144  17.889  30.883  1.00  0.00           C
ATOM   2855  O   ASN A 203      50.759  18.913  30.566  1.00  0.00           O
ATOM   2856  N   LEU A 204      48.811  17.812  30.765  1.00  0.00           N
ATOM   2857  H   LEU A 204      48.362  16.929  30.987  1.00  0.00           H
ATOM   2858  CA  LEU A 204      47.955  18.969  30.472  1.00  0.00           C
ATOM   2859  HA  LEU A 204      48.406  19.558  29.672  1.00  0.00           H
```

ATOM	2860	CB	LEU	A	204	46.552	18.499	30.029	1.00	0.00	C
ATOM	2861	HB1	LEU	A	204	46.143	17.852	30.808	1.00	0.00	H
ATOM	2862	HB2	LEU	A	204	45.900	19.372	29.952	1.00	0.00	H
ATOM	2863	CG	LEU	A	204	46.511	17.746	28.683	1.00	0.00	C
ATOM	2864	HG	LEU	A	204	47.169	16.881	28.739	1.00	0.00	H
ATOM	2865	CD1	LEU	A	204	45.091	17.242	28.398	1.00	0.00	C
ATOM	2866	HD11	LEU	A	204	45.085	16.662	27.475	1.00	0.00	H
ATOM	2867	HD12	LEU	A	204	44.752	16.604	29.215	1.00	0.00	H
ATOM	2868	HD13	LEU	A	204	44.410	18.087	28.287	1.00	0.00	H
ATOM	2869	CD2	LEU	A	204	46.963	18.627	27.512	1.00	0.00	C
ATOM	2870	HD21	LEU	A	204	46.839	18.091	26.571	1.00	0.00	H
ATOM	2871	HD22	LEU	A	204	46.373	19.544	27.478	1.00	0.00	H
ATOM	2872	HD23	LEU	A	204	48.015	18.876	27.626	1.00	0.00	H
ATOM	2873	C	LEU	A	204	47.863	19.890	31.695	1.00	0.00	C
ATOM	2874	O	LEU	A	204	47.898	19.431	32.835	1.00	0.00	O
ATOM	2875	N	ASP	A	205	47.712	21.191	31.465	1.00	0.00	N
ATOM	2876	H	ASP	A	205	47.684	21.505	30.498	1.00	0.00	H
ATOM	2877	CA	ASP	A	205	47.476	22.190	32.510	1.00	0.00	C
ATOM	2878	HA	ASP	A	205	48.012	21.899	33.414	1.00	0.00	H
ATOM	2879	CB	ASP	A	205	48.023	23.543	32.035	1.00	0.00	C
ATOM	2880	HB1	ASP	A	205	47.807	23.702	30.978	1.00	0.00	H
ATOM	2881	HB2	ASP	A	205	47.539	24.342	32.599	1.00	0.00	H
ATOM	2882	CG	ASP	A	205	49.522	23.620	32.300	1.00	0.00	C
ATOM	2883	OD1	ASP	A	205	49.868	23.829	33.487	1.00	0.00	O
ATOM	2884	OD2	ASP	A	205	50.340	23.474	31.361	1.00	0.00	O
ATOM	2885	C	ASP	A	205	45.992	22.290	32.886	1.00	0.00	C
ATOM	2886	O	ASP	A	205	45.664	22.401	34.070	1.00	0.00	O
ATOM	2887	N	PHE	A	206	45.101	22.179	31.897	1.00	0.00	N
ATOM	2888	H	PHE	A	206	45.453	22.080	30.953	1.00	0.00	H
ATOM	2889	CA	PHE	A	206	43.653	22.090	32.075	1.00	0.00	C
ATOM	2890	HA	PHE	A	206	43.481	21.403	32.902	1.00	0.00	H
ATOM	2891	CB	PHE	A	206	43.052	23.448	32.490	1.00	0.00	C
ATOM	2892	HB1	PHE	A	206	41.993	23.309	32.703	1.00	0.00	H
ATOM	2893	HB2	PHE	A	206	43.508	23.759	33.431	1.00	0.00	H
ATOM	2894	CG	PHE	A	206	43.195	24.587	31.498	1.00	0.00	C
ATOM	2895	CD1	PHE	A	206	44.355	25.386	31.501	1.00	0.00	C
ATOM	2896	HD1	PHE	A	206	45.168	25.161	32.178	1.00	0.00	H
ATOM	2897	CE1	PHE	A	206	44.457	26.489	30.635	1.00	0.00	C
ATOM	2898	HE1	PHE	A	206	45.350	27.097	30.639	1.00	0.00	H
ATOM	2899	CZ	PHE	A	206	43.396	26.801	29.767	1.00	0.00	C
ATOM	2900	HZ	PHE	A	206	43.473	27.646	29.098	1.00	0.00	H
ATOM	2901	CE2	PHE	A	206	42.238	26.006	29.760	1.00	0.00	C
ATOM	2902	HE2	PHE	A	206	41.422	26.245	29.094	1.00	0.00	H
ATOM	2903	CD2	PHE	A	206	42.140	24.896	30.617	1.00	0.00	C
ATOM	2904	HD2	PHE	A	206	41.246	24.288	30.598	1.00	0.00	H
ATOM	2905	C	PHE	A	206	42.977	21.468	30.844	1.00	0.00	C
ATOM	2906	O	PHE	A	206	43.559	21.398	29.764	1.00	0.00	O
ATOM	2907	N	VAL	A	207	41.756	20.971	31.031	1.00	0.00	N
ATOM	2908	H	VAL	A	207	41.353	21.084	31.958	1.00	0.00	H
ATOM	2909	CA	VAL	A	207	41.026	20.124	30.078	1.00	0.00	C
ATOM	2910	HA	VAL	A	207	41.572	20.075	29.134	1.00	0.00	H
ATOM	2911	CB	VAL	A	207	40.880	18.686	30.637	1.00	0.00	C
ATOM	2912	HB	VAL	A	207	40.336	18.730	31.584	1.00	0.00	H
ATOM	2913	CG1	VAL	A	207	40.097	17.781	29.677	1.00	0.00	C

ATOM	2914	HG11	VAL	A	207	40.045	16.769	30.081	1.00	0.00	H
ATOM	2915	HG12	VAL	A	207	39.079	18.150	29.558	1.00	0.00	H
ATOM	2916	HG13	VAL	A	207	40.590	17.755	28.704	1.00	0.00	H
ATOM	2917	CG2	VAL	A	207	42.255	18.058	30.918	1.00	0.00	C
ATOM	2918	HG21	VAL	A	207	42.139	17.016	31.212	1.00	0.00	H
ATOM	2919	HG22	VAL	A	207	42.871	18.106	30.022	1.00	0.00	H
ATOM	2920	HG23	VAL	A	207	42.758	18.587	31.729	1.00	0.00	H
ATOM	2921	C	VAL	A	207	39.653	20.743	29.822	1.00	0.00	C
ATOM	2922	O	VAL	A	207	38.856	20.873	30.750	1.00	0.00	O
ATOM	2923	N	ASN	A	208	39.387	21.128	28.572	1.00	0.00	N
ATOM	2924	H	ASN	A	208	40.072	20.931	27.848	1.00	0.00	H
ATOM	2925	CA	ASN	A	208	38.164	21.816	28.156	1.00	0.00	C
ATOM	2926	HA	ASN	A	208	37.711	22.309	29.018	1.00	0.00	H
ATOM	2927	CB	ASN	A	208	38.531	22.907	27.131	1.00	0.00	C
ATOM	2928	HB1	ASN	A	208	39.033	22.459	26.275	1.00	0.00	H
ATOM	2929	HB2	ASN	A	208	37.626	23.387	26.768	1.00	0.00	H
ATOM	2930	CG	ASN	A	208	39.429	23.986	27.731	1.00	0.00	C
ATOM	2931	OD1	ASN	A	208	39.128	24.541	28.782	1.00	0.00	O
ATOM	2932	ND2	ASN	A	208	40.552	24.299	27.107	1.00	0.00	N
ATOM	2933	HD21	ASN	A	208	40.803	23.892	26.209	1.00	0.00	H
ATOM	2934	HD22	ASN	A	208	41.150	24.999	27.509	1.00	0.00	H
ATOM	2935	C	ASN	A	208	37.150	20.796	27.605	1.00	0.00	C
ATOM	2936	O	ASN	A	208	37.276	20.321	26.470	1.00	0.00	O
ATOM	2937	N	LEU	A	209	36.152	20.424	28.414	1.00	0.00	N
ATOM	2938	H	LEU	A	209	36.080	20.853	29.335	1.00	0.00	H
ATOM	2939	CA	LEU	A	209	35.172	19.388	28.069	1.00	0.00	C
ATOM	2940	HA	LEU	A	209	35.678	18.609	27.498	1.00	0.00	H
ATOM	2941	CB	LEU	A	209	34.648	18.761	29.376	1.00	0.00	C
ATOM	2942	HB1	LEU	A	209	35.500	18.387	29.945	1.00	0.00	H
ATOM	2943	HB2	LEU	A	209	34.173	19.546	29.969	1.00	0.00	H
ATOM	2944	CG	LEU	A	209	33.637	17.610	29.193	1.00	0.00	C
ATOM	2945	HG	LEU	A	209	32.778	17.976	28.632	1.00	0.00	H
ATOM	2946	CD1	LEU	A	209	34.234	16.420	28.427	1.00	0.00	C
ATOM	2947	HD11	LEU	A	209	33.525	15.593	28.417	1.00	0.00	H
ATOM	2948	HD12	LEU	A	209	34.443	16.701	27.395	1.00	0.00	H
ATOM	2949	HD13	LEU	A	209	35.157	16.092	28.907	1.00	0.00	H
ATOM	2950	CD2	LEU	A	209	33.140	17.148	30.571	1.00	0.00	C
ATOM	2951	HD21	LEU	A	209	32.378	16.379	30.451	1.00	0.00	H
ATOM	2952	HD22	LEU	A	209	33.966	16.739	31.154	1.00	0.00	H
ATOM	2953	HD23	LEU	A	209	32.701	17.988	31.109	1.00	0.00	H
ATOM	2954	C	LEU	A	209	34.049	19.967	27.199	1.00	0.00	C
ATOM	2955	O	LEU	A	209	33.259	20.785	27.668	1.00	0.00	O
ATOM	2956	N	MET	A	210	33.946	19.509	25.947	1.00	0.00	N
ATOM	2957	H	MET	A	210	34.623	18.821	25.639	1.00	0.00	H
ATOM	2958	CA	MET	A	210	32.905	19.899	24.980	1.00	0.00	C
ATOM	2959	HA	MET	A	210	32.746	20.978	25.038	1.00	0.00	H
ATOM	2960	CB	MET	A	210	33.361	19.562	23.547	1.00	0.00	C
ATOM	2961	HB1	MET	A	210	33.519	18.486	23.471	1.00	0.00	H
ATOM	2962	HB2	MET	A	210	32.563	19.838	22.854	1.00	0.00	H
ATOM	2963	CG	MET	A	210	34.640	20.292	23.117	1.00	0.00	C
ATOM	2964	HG1	MET	A	210	34.465	21.365	23.216	1.00	0.00	H
ATOM	2965	HG2	MET	A	210	35.460	20.025	23.784	1.00	0.00	H
ATOM	2966	SD	MET	A	210	35.144	19.967	21.404	1.00	0.00	S
ATOM	2967	CE	MET	A	210	35.696	18.249	21.513	1.00	0.00	C

ATOM	2968	HE1	MET	A	210	36.582	18.198	22.139	1.00	0.00	H
ATOM	2969	HE2	MET	A	210	34.919	17.627	21.954	1.00	0.00	H
ATOM	2970	HE3	MET	A	210	35.930	17.882	20.515	1.00	0.00	H
ATOM	2971	C	MET	A	210	31.562	19.228	25.313	1.00	0.00	C
ATOM	2972	O	MET	A	210	31.085	18.357	24.582	1.00	0.00	O
ATOM	2973	N	ALA	A	211	30.974	19.574	26.461	1.00	0.00	N
ATOM	2974	H	ALA	A	211	31.444	20.277	27.021	1.00	0.00	H
ATOM	2975	CA	ALA	A	211	29.739	18.988	26.986	1.00	0.00	C
ATOM	2976	HA	ALA	A	211	29.761	17.913	26.787	1.00	0.00	H
ATOM	2977	CB	ALA	A	211	29.726	19.185	28.507	1.00	0.00	C
ATOM	2978	HB1	ALA	A	211	28.924	18.589	28.939	1.00	0.00	H
ATOM	2979	HB2	ALA	A	211	30.666	18.852	28.944	1.00	0.00	H
ATOM	2980	HB3	ALA	A	211	29.570	20.238	28.749	1.00	0.00	H
ATOM	2981	C	ALA	A	211	28.489	19.536	26.265	1.00	0.00	C
ATOM	2982	O	ALA	A	211	27.574	20.074	26.870	1.00	0.00	O
ATOM	2983	N	TYR	A	212	28.458	19.394	24.879	1.00	0.00	N
ATOM	2984	H	TYR	A	212	29.263	18.945	24.434	1.00	0.00	H
ATOM	2985	CA	TYR	A	212	27.504	20.039	24.015	1.00	0.00	C
ATOM	2986	HA	TYR	A	212	26.479	19.871	24.388	1.00	0.00	H
ATOM	2987	CB	TYR	A	212	27.685	21.585	23.863	1.00	0.00	C
ATOM	2988	HB1	TYR	A	212	27.063	21.929	23.020	1.00	0.00	H
ATOM	2989	HB2	TYR	A	212	27.260	22.046	24.768	1.00	0.00	H
ATOM	2990	CG	TYR	A	212	29.103	22.079	23.706	1.00	0.00	C
ATOM	2991	CD1	TYR	A	212	29.828	21.917	22.514	1.00	0.00	C
ATOM	2992	HD1	TYR	A	212	29.379	21.383	21.674	1.00	0.00	H
ATOM	2993	CE1	TYR	A	212	31.103	22.461	22.353	1.00	0.00	C
ATOM	2994	HE1	TYR	A	212	31.627	22.360	21.402	1.00	0.00	H
ATOM	2995	CZ	TYR	A	212	31.694	23.203	23.394	1.00	0.00	C
ATOM	2996	OH	TYR	A	212	32.910	23.799	23.293	1.00	0.00	O
ATOM	2997	HH	TYR	A	212	33.270	23.756	22.352	1.00	0.00	H
ATOM	2998	CE2	TYR	A	212	30.994	23.341	24.605	1.00	0.00	C
ATOM	2999	HE2	TYR	A	212	31.443	23.942	25.397	1.00	0.00	H
ATOM	3000	CD2	TYR	A	212	29.725	22.781	24.750	1.00	0.00	C
ATOM	3001	HD2	TYR	A	212	29.190	22.917	25.694	1.00	0.00	H
ATOM	3002	C	TYR	A	212	27.576	19.276	22.681	1.00	0.00	C
ATOM	3003	O	TYR	A	212	28.484	18.460	22.451	1.00	0.00	O
ATOM	3004	N	ASP	A	213	26.574	19.513	21.808	1.00	0.00	N
ATOM	3005	H	ASP	A	213	25.873	20.240	21.967	1.00	0.00	H
ATOM	3006	CA	ASP	A	213	26.435	18.756	20.563	1.00	0.00	C
ATOM	3007	HA	ASP	A	213	25.519	19.156	20.094	1.00	0.00	H
ATOM	3008	CB	ASP	A	213	27.602	18.959	19.595	1.00	0.00	C
ATOM	3009	HB1	ASP	A	213	28.514	18.508	20.001	1.00	0.00	H
ATOM	3010	HB2	ASP	A	213	27.389	18.417	18.662	1.00	0.00	H
ATOM	3011	CG	ASP	A	213	27.872	20.422	19.259	1.00	0.00	C
ATOM	3012	OD1	ASP	A	213	27.014	21.290	19.566	1.00	0.00	O
ATOM	3013	OD2	ASP	A	213	28.982	20.646	18.652	1.00	0.00	O
ATOM	3014	C	ASP	A	213	26.138	17.275	20.822	1.00	0.00	C
ATOM	3015	O	ASP	A	213	26.454	16.428	19.989	1.00	0.00	O
ATOM	3016	N	PHE	A	214	25.475	16.915	21.978	1.00	0.00	N
ATOM	3017	H	PHE	A	214	25.183	17.624	22.670	1.00	0.00	H
ATOM	3018	CA	PHE	A	214	25.177	15.515	22.324	1.00	0.00	C
ATOM	3019	HA	PHE	A	214	26.089	14.920	22.252	1.00	0.00	H
ATOM	3020	CB	PHE	A	214	24.672	15.455	23.769	1.00	0.00	C
ATOM	3021	HB1	PHE	A	214	23.908	16.219	23.924	1.00	0.00	H

ATOM	3022	HB2	PHE	A	214	24.175	14.498	23.916	1.00	0.00	H
ATOM	3023	CG	PHE	A	214	25.740	15.568	24.840	1.00	0.00	C
ATOM	3024	CD1	PHE	A	214	26.630	14.503	25.070	1.00	0.00	C
ATOM	3025	HD1	PHE	A	214	26.603	13.628	24.436	1.00	0.00	H
ATOM	3026	CE1	PHE	A	214	27.528	14.557	26.151	1.00	0.00	C
ATOM	3027	HE1	PHE	A	214	28.188	13.723	26.342	1.00	0.00	H
ATOM	3028	CZ	PHE	A	214	27.545	15.682	26.997	1.00	0.00	C
ATOM	3029	HZ	PHE	A	214	28.217	15.718	27.841	1.00	0.00	H
ATOM	3030	CE2	PHE	A	214	26.672	16.754	26.756	1.00	0.00	C
ATOM	3031	HE2	PHE	A	214	26.675	17.612	27.412	1.00	0.00	H
ATOM	3032	CD2	PHE	A	214	25.782	16.698	25.671	1.00	0.00	C
ATOM	3033	HD2	PHE	A	214	25.111	17.513	25.481	1.00	0.00	H
ATOM	3034	C	PHE	A	214	24.138	14.868	21.392	1.00	0.00	C
ATOM	3035	O	PHE	A	214	24.329	13.741	20.935	1.00	0.00	O
ATOM	3036	N	HIS	A	215	23.075	15.599	21.075	1.00	0.00	N
ATOM	3037	H	HIS	A	215	22.990	16.511	21.495	1.00	0.00	H
ATOM	3038	CA	HIS	A	215	22.143	15.287	19.993	1.00	0.00	C
ATOM	3039	HA	HIS	A	215	22.346	14.291	19.595	1.00	0.00	H
ATOM	3040	CB	HIS	A	215	20.717	15.310	20.557	1.00	0.00	C
ATOM	3041	HB1	HIS	A	215	20.547	16.242	21.089	1.00	0.00	H
ATOM	3042	HB2	HIS	A	215	19.998	15.269	19.739	1.00	0.00	H
ATOM	3043	CG	HIS	A	215	20.463	14.137	21.469	1.00	0.00	C
ATOM	3044	ND1	HIS	A	215	20.709	12.819	21.160	1.00	0.00	N
ATOM	3045	HD1	HIS	A	215	21.008	12.473	20.250	1.00	0.00	H
ATOM	3046	CE1	HIS	A	215	20.396	12.075	22.229	1.00	0.00	C
ATOM	3047	HE1	HIS	A	215	20.463	10.999	22.275	1.00	0.00	H
ATOM	3048	NE2	HIS	A	215	19.909	12.849	23.212	1.00	0.00	N
ATOM	3049	CD2	HIS	A	215	19.987	14.167	22.752	1.00	0.00	C
ATOM	3050	HD2	HIS	A	215	19.725	15.051	23.316	1.00	0.00	H
ATOM	3051	C	HIS	A	215	22.338	16.275	18.834	1.00	0.00	C
ATOM	3052	O	HIS	A	215	22.822	17.391	19.054	1.00	0.00	O
ATOM	3053	N	GLY	A	216	21.967	15.887	17.607	1.00	0.00	N
ATOM	3054	H	GLY	A	216	21.492	14.994	17.499	1.00	0.00	H
ATOM	3055	CA	GLY	A	216	22.257	16.676	16.401	1.00	0.00	C
ATOM	3056	HA1	GLY	A	216	22.100	17.734	16.615	1.00	0.00	H
ATOM	3057	HA2	GLY	A	216	23.311	16.562	16.161	1.00	0.00	H
ATOM	3058	C	GLY	A	216	21.449	16.306	15.160	1.00	0.00	C
ATOM	3059	O	GLY	A	216	20.718	15.321	15.143	1.00	0.00	O
ATOM	3060	N	SER	A	217	21.595	17.105	14.102	1.00	0.00	N
ATOM	3061	H	SER	A	217	22.239	17.886	14.196	1.00	0.00	H
ATOM	3062	CA	SER	A	217	20.831	17.004	12.842	1.00	0.00	C
ATOM	3063	HA	SER	A	217	19.772	17.134	13.067	1.00	0.00	H
ATOM	3064	CB	SER	A	217	21.254	18.126	11.879	1.00	0.00	C
ATOM	3065	HB1	SER	A	217	20.528	18.174	11.068	1.00	0.00	H
ATOM	3066	HB2	SER	A	217	21.241	19.079	12.410	1.00	0.00	H
ATOM	3067	OG	SER	A	217	22.548	17.935	11.324	1.00	0.00	O
ATOM	3068	HG	SER	A	217	22.429	17.513	10.443	1.00	0.00	H
ATOM	3069	C	SER	A	217	20.952	15.650	12.133	1.00	0.00	C
ATOM	3070	O	SER	A	217	20.090	15.277	11.340	1.00	0.00	O
ATOM	3071	N	TRP	A	218	21.996	14.890	12.460	1.00	0.00	N
ATOM	3072	H	TRP	A	218	22.651	15.275	13.124	1.00	0.00	H
ATOM	3073	CA	TRP	A	218	22.246	13.524	12.022	1.00	0.00	C
ATOM	3074	HA	TRP	A	218	22.090	13.473	10.942	1.00	0.00	H
ATOM	3075	CB	TRP	A	218	23.728	13.245	12.316	1.00	0.00	C

ATOM	3076	HB1	TRP	A	218	23.957	12.211	12.062	1.00	0.00	H
ATOM	3077	HB2	TRP	A	218	24.323	13.878	11.659	1.00	0.00	H
ATOM	3078	CG	TRP	A	218	24.188	13.489	13.723	1.00	0.00	C
ATOM	3079	CD1	TRP	A	218	24.062	12.613	14.740	1.00	0.00	C
ATOM	3080	HD1	TRP	A	218	23.610	11.634	14.659	1.00	0.00	H
ATOM	3081	NE1	TRP	A	218	24.595	13.148	15.894	1.00	0.00	N
ATOM	3082	HE1	TRP	A	218	24.605	12.639	16.776	1.00	0.00	H
ATOM	3083	CE2	TRP	A	218	25.125	14.402	15.672	1.00	0.00	C
ATOM	3084	CZ2	TRP	A	218	25.786	15.328	16.494	1.00	0.00	C
ATOM	3085	HZ2	TRP	A	218	25.956	15.107	17.538	1.00	0.00	H
ATOM	3086	CH2	TRP	A	218	26.228	16.543	15.942	1.00	0.00	C
ATOM	3087	HH2	TRP	A	218	26.748	17.260	16.563	1.00	0.00	H
ATOM	3088	CZ3	TRP	A	218	25.995	16.820	14.584	1.00	0.00	C
ATOM	3089	HZ3	TRP	A	218	26.343	17.753	14.158	1.00	0.00	H
ATOM	3090	CE3	TRP	A	218	25.312	15.893	13.772	1.00	0.00	C
ATOM	3091	HE3	TRP	A	218	25.138	16.124	12.730	1.00	0.00	H
ATOM	3092	CD2	TRP	A	218	24.870	14.654	14.289	1.00	0.00	C
ATOM	3093	C	TRP	A	218	21.313	12.467	12.656	1.00	0.00	C
ATOM	3094	O	TRP	A	218	21.456	11.284	12.335	1.00	0.00	O
ATOM	3095	N	GLU	A	219	20.393	12.854	13.551	1.00	0.00	N
ATOM	3096	H	GLU	A	219	20.373	13.835	13.810	1.00	0.00	H
ATOM	3097	CA	GLU	A	219	19.459	11.964	14.256	1.00	0.00	C
ATOM	3098	HA	GLU	A	219	19.717	10.929	14.043	1.00	0.00	H
ATOM	3099	CB	GLU	A	219	19.550	12.182	15.772	1.00	0.00	C
ATOM	3100	HB1	GLU	A	219	19.192	13.186	16.005	1.00	0.00	H
ATOM	3101	HB2	GLU	A	219	18.890	11.466	16.265	1.00	0.00	H
ATOM	3102	CG	GLU	A	219	20.964	12.012	16.338	1.00	0.00	C
ATOM	3103	HG1	GLU	A	219	21.340	11.018	16.092	1.00	0.00	H
ATOM	3104	HG2	GLU	A	219	21.624	12.763	15.908	1.00	0.00	H
ATOM	3105	CD	GLU	A	219	20.951	12.204	17.846	1.00	0.00	C
ATOM	3106	OE1	GLU	A	219	20.445	13.240	18.316	1.00	0.00	O
ATOM	3107	OE2	GLU	A	219	21.388	11.298	18.596	1.00	0.00	O
ATOM	3108	C	GLU	A	219	17.999	12.197	13.837	1.00	0.00	C
ATOM	3109	O	GLU	A	219	17.530	13.331	13.743	1.00	0.00	O
ATOM	3110	N	LYS	A	220	17.222	11.127	13.651	1.00	0.00	N
ATOM	3111	H	LYS	A	220	17.642	10.211	13.763	1.00	0.00	H
ATOM	3112	CA	LYS	A	220	15.845	11.199	13.122	1.00	0.00	C
ATOM	3113	HA	LYS	A	220	15.839	11.973	12.354	1.00	0.00	H
ATOM	3114	CB	LYS	A	220	15.503	9.869	12.422	1.00	0.00	C
ATOM	3115	HB1	LYS	A	220	14.519	9.957	11.959	1.00	0.00	H
ATOM	3116	HB2	LYS	A	220	16.228	9.703	11.623	1.00	0.00	H
ATOM	3117	CG	LYS	A	220	15.505	8.643	13.350	1.00	0.00	C
ATOM	3118	HG1	LYS	A	220	16.518	8.480	13.720	1.00	0.00	H
ATOM	3119	HG2	LYS	A	220	14.840	8.806	14.197	1.00	0.00	H
ATOM	3120	CD	LYS	A	220	15.040	7.401	12.580	1.00	0.00	C
ATOM	3121	HD1	LYS	A	220	13.964	7.455	12.404	1.00	0.00	H
ATOM	3122	HD2	LYS	A	220	15.557	7.369	11.620	1.00	0.00	H
ATOM	3123	CE	LYS	A	220	15.379	6.140	13.377	1.00	0.00	C
ATOM	3124	HE1	LYS	A	220	16.423	6.207	13.686	1.00	0.00	H
ATOM	3125	HE2	LYS	A	220	14.767	6.095	14.280	1.00	0.00	H
ATOM	3126	NZ	LYS	A	220	15.200	4.908	12.580	1.00	0.00	N
ATOM	3127	HZ1	LYS	A	220	15.625	4.130	13.073	1.00	0.00	H
ATOM	3128	HZ2	LYS	A	220	14.216	4.706	12.426	1.00	0.00	H
ATOM	3129	HZ3	LYS	A	220	15.670	5.005	11.684	1.00	0.00	H

ATOM	3130	C	LYS	A	220	14.754	11.636	14.128	1.00	0.00	C
ATOM	3131	O	LYS	A	220	13.562	11.478	13.846	1.00	0.00	O
ATOM	3132	N	VAL	A	221	15.134	12.156	15.296	1.00	0.00	N
ATOM	3133	H	VAL	A	221	16.114	12.396	15.383	1.00	0.00	H
ATOM	3134	CA	VAL	A	221	14.232	12.486	16.420	1.00	0.00	C
ATOM	3135	HA	VAL	A	221	13.284	12.796	15.986	1.00	0.00	H
ATOM	3136	CB	VAL	A	221	13.964	11.294	17.376	1.00	0.00	C
ATOM	3137	HB	VAL	A	221	13.438	11.678	18.250	1.00	0.00	H
ATOM	3138	CG1	VAL	A	221	13.041	10.247	16.743	1.00	0.00	C
ATOM	3139	HG11	VAL	A	221	12.784	9.495	17.488	1.00	0.00	H
ATOM	3140	HG12	VAL	A	221	12.124	10.725	16.396	1.00	0.00	H
ATOM	3141	HG13	VAL	A	221	13.535	9.754	15.907	1.00	0.00	H
ATOM	3142	CG2	VAL	A	221	15.254	10.612	17.867	1.00	0.00	C
ATOM	3143	HG21	VAL	A	221	15.005	9.820	18.574	1.00	0.00	H
ATOM	3144	HG22	VAL	A	221	15.802	10.176	17.030	1.00	0.00	H
ATOM	3145	HG23	VAL	A	221	15.892	11.335	18.375	1.00	0.00	H
ATOM	3146	C	VAL	A	221	14.752	13.654	17.259	1.00	0.00	C
ATOM	3147	O	VAL	A	221	15.966	13.870	17.330	1.00	0.00	O
ATOM	3148	N	THR	A	222	13.829	14.379	17.905	1.00	0.00	N
ATOM	3149	H	THR	A	222	12.849	14.142	17.777	1.00	0.00	H
ATOM	3150	CA	THR	A	222	14.137	15.515	18.783	1.00	0.00	C
ATOM	3151	HA	THR	A	222	14.860	16.140	18.261	1.00	0.00	H
ATOM	3152	CB	THR	A	222	12.904	16.391	19.042	1.00	0.00	C
ATOM	3153	HB	THR	A	222	13.179	17.179	19.742	1.00	0.00	H
ATOM	3154	CG2	THR	A	222	12.374	17.035	17.760	1.00	0.00	C
ATOM	3155	HG21	THR	A	222	11.509	17.652	17.995	1.00	0.00	H
ATOM	3156	HG22	THR	A	222	13.150	17.662	17.318	1.00	0.00	H
ATOM	3157	HG23	THR	A	222	12.076	16.270	17.042	1.00	0.00	H
ATOM	3158	OG1	THR	A	222	11.875	15.615	19.597	1.00	0.00	O
ATOM	3159	HG1	THR	A	222	11.179	16.208	19.934	1.00	0.00	H
ATOM	3160	C	THR	A	222	14.795	15.077	20.094	1.00	0.00	C
ATOM	3161	O	THR	A	222	14.447	14.055	20.689	1.00	0.00	O
ATOM	3162	N	GLY	A	223	15.765	15.879	20.528	1.00	0.00	N
ATOM	3163	H	GLY	A	223	15.937	16.705	19.970	1.00	0.00	H
ATOM	3164	CA	GLY	A	223	16.657	15.653	21.670	1.00	0.00	C
ATOM	3165	HA1	GLY	A	223	16.062	15.514	22.572	1.00	0.00	H
ATOM	3166	HA2	GLY	A	223	17.227	14.736	21.521	1.00	0.00	H
ATOM	3167	C	GLY	A	223	17.631	16.817	21.871	1.00	0.00	C
ATOM	3168	O	GLY	A	223	17.986	17.516	20.917	1.00	0.00	O
ATOM	3169	N	HIS	A	224	18.039	17.068	23.112	1.00	0.00	N
ATOM	3170	H	HIS	A	224	17.719	16.441	23.850	1.00	0.00	H
ATOM	3171	CA	HIS	A	224	18.705	18.312	23.520	1.00	0.00	C
ATOM	3172	HA	HIS	A	224	18.216	19.125	22.981	1.00	0.00	H
ATOM	3173	CB	HIS	A	224	18.407	18.527	25.008	1.00	0.00	C
ATOM	3174	HB1	HIS	A	224	17.363	18.282	25.205	1.00	0.00	H
ATOM	3175	HB2	HIS	A	224	19.025	17.858	25.605	1.00	0.00	H
ATOM	3176	CG	HIS	A	224	18.638	19.946	25.434	1.00	0.00	C
ATOM	3177	ND1	HIS	A	224	19.875	20.497	25.765	1.00	0.00	N
ATOM	3178	CE1	HIS	A	224	19.642	21.796	26.005	1.00	0.00	C
ATOM	3179	HE1	HIS	A	224	20.389	22.523	26.302	1.00	0.00	H
ATOM	3180	NE2	HIS	A	224	18.338	22.075	25.841	1.00	0.00	N
ATOM	3181	HE2	HIS	A	224	17.921	22.994	25.990	1.00	0.00	H
ATOM	3182	CD2	HIS	A	224	17.690	20.924	25.464	1.00	0.00	C
ATOM	3183	HD2	HIS	A	224	16.638	20.815	25.243	1.00	0.00	H

```
ATOM   3184  C   HIS A 224      20.224  18.413  23.206  1.00  0.00           C
ATOM   3185  O   HIS A 224      20.970  17.436  23.251  1.00  0.00           O
ATOM   3186  N   ASN A 225      20.700  19.632  22.927  1.00  0.00           N
ATOM   3187  H   ASN A 225      20.035  20.389  22.868  1.00  0.00           H
ATOM   3188  CA  ASN A 225      22.101  19.948  22.619  1.00  0.00           C
ATOM   3189  HA  ASN A 225      22.431  19.317  21.791  1.00  0.00           H
ATOM   3190  CB  ASN A 225      22.148  21.417  22.160  1.00  0.00           C
ATOM   3191  HB1 ASN A 225      21.541  21.540  21.262  1.00  0.00           H
ATOM   3192  HB2 ASN A 225      21.719  22.048  22.940  1.00  0.00           H
ATOM   3193  CG  ASN A 225      23.563  21.897  21.851  1.00  0.00           C
ATOM   3194  OD1 ASN A 225      24.343  21.210  21.209  1.00  0.00           O
ATOM   3195  ND2 ASN A 225      23.945  23.067  22.321  1.00  0.00           N
ATOM   3196 HD21 ASN A 225      23.314  23.613  22.903  1.00  0.00           H
ATOM   3197 HD22 ASN A 225      24.853  23.422  22.073  1.00  0.00           H
ATOM   3198  C   ASN A 225      23.068  19.720  23.803  1.00  0.00           C
ATOM   3199  O   ASN A 225      24.165  19.197  23.620  1.00  0.00           O
ATOM   3200  N   SER A 226      22.680  20.099  25.021  1.00  0.00           N
ATOM   3201  H   SER A 226      21.742  20.476  25.123  1.00  0.00           H
ATOM   3202  CA  SER A 226      23.521  20.049  26.227  1.00  0.00           C
ATOM   3203  HA  SER A 226      24.234  19.238  26.132  1.00  0.00           H
ATOM   3204  CB  SER A 226      24.348  21.343  26.303  1.00  0.00           C
ATOM   3205  HB1 SER A 226      24.851  21.487  25.347  1.00  0.00           H
ATOM   3206  HB2 SER A 226      23.675  22.186  26.466  1.00  0.00           H
ATOM   3207  OG  SER A 226      25.328  21.312  27.330  1.00  0.00           O
ATOM   3208  HG  SER A 226      26.091  20.759  27.050  1.00  0.00           H
ATOM   3209  C   SER A 226      22.707  19.753  27.509  1.00  0.00           C
ATOM   3210  O   SER A 226      22.658  20.589  28.417  1.00  0.00           O
ATOM   3211  N   PRO A 227      22.050  18.578  27.599  1.00  0.00           N
ATOM   3212  CD  PRO A 227      21.988  17.542  26.572  1.00  0.00           C
ATOM   3213  HD1 PRO A 227      22.978  17.264  26.215  1.00  0.00           H
ATOM   3214  HD2 PRO A 227      21.373  17.891  25.746  1.00  0.00           H
ATOM   3215  CG  PRO A 227      21.317  16.335  27.213  1.00  0.00           C
ATOM   3216  HG1 PRO A 227      22.075  15.697  27.658  1.00  0.00           H
ATOM   3217  HG2 PRO A 227      20.715  15.774  26.495  1.00  0.00           H
ATOM   3218  CB  PRO A 227      20.463  16.961  28.310  1.00  0.00           C
ATOM   3219  HB1 PRO A 227      20.303  16.257  29.125  1.00  0.00           H
ATOM   3220  HB2 PRO A 227      19.513  17.290  27.888  1.00  0.00           H
ATOM   3221  CA  PRO A 227      21.268  18.178  28.767  1.00  0.00           C
ATOM   3222  HA  PRO A 227      20.586  18.979  29.054  1.00  0.00           H
ATOM   3223  C   PRO A 227      22.166  17.827  29.959  1.00  0.00           C
ATOM   3224  O   PRO A 227      23.273  17.314  29.776  1.00  0.00           O
ATOM   3225  N   LEU A 228      21.681  18.066  31.187  1.00  0.00           N
ATOM   3226  H   LEU A 228      20.765  18.492  31.293  1.00  0.00           H
ATOM   3227  CA  LEU A 228      22.443  17.749  32.400  1.00  0.00           C
ATOM   3228  HA  LEU A 228      23.463  18.068  32.225  1.00  0.00           H
ATOM   3229  CB  LEU A 228      21.906  18.560  33.596  1.00  0.00           C
ATOM   3230  HB1 LEU A 228      21.892  19.613  33.315  1.00  0.00           H
ATOM   3231  HB2 LEU A 228      20.881  18.251  33.803  1.00  0.00           H
ATOM   3232  CG  LEU A 228      22.734  18.412  34.894  1.00  0.00           C
ATOM   3233  HG  LEU A 228      22.664  17.382  35.242  1.00  0.00           H
ATOM   3234  CD1 LEU A 228      24.222  18.754  34.717  1.00  0.00           C
ATOM   3235 HD11 LEU A 228      24.715  18.780  35.689  1.00  0.00           H
ATOM   3236 HD12 LEU A 228      24.710  17.985  34.122  1.00  0.00           H
ATOM   3237 HD13 LEU A 228      24.333  19.725  34.233  1.00  0.00           H
```

```
ATOM   3238  CD2 LEU A 228      22.144  19.329  35.974  1.00  0.00           C
ATOM   3239 HD21 LEU A 228      22.680  19.190  36.914  1.00  0.00           H
ATOM   3240 HD22 LEU A 228      22.228  20.371  35.665  1.00  0.00           H
ATOM   3241 HD23 LEU A 228      21.096  19.085  36.132  1.00  0.00           H
ATOM   3242  C   LEU A 228      22.492  16.238  32.666  1.00  0.00           C
ATOM   3243  O   LEU A 228      23.578  15.679  32.768  1.00  0.00           O
ATOM   3244  N   TYR A 229      21.330  15.586  32.730  1.00  0.00           N
ATOM   3245  H   TYR A 229      20.499  16.126  32.555  1.00  0.00           H
ATOM   3246  CA  TYR A 229      21.159  14.143  32.978  1.00  0.00           C
ATOM   3247  HA  TYR A 229      22.130  13.672  33.144  1.00  0.00           H
ATOM   3248  CB  TYR A 229      20.304  13.976  34.249  1.00  0.00           C
ATOM   3249  HB1 TYR A 229      19.316  14.401  34.067  1.00  0.00           H
ATOM   3250  HB2 TYR A 229      20.168  12.914  34.455  1.00  0.00           H
ATOM   3251  CG  TYR A 229      20.908  14.610  35.486  1.00  0.00           C
ATOM   3252  CD1 TYR A 229      22.106  14.093  36.011  1.00  0.00           C
ATOM   3253  HD1 TYR A 229      22.585  13.248  35.533  1.00  0.00           H
ATOM   3254  CE1 TYR A 229      22.705  14.695  37.130  1.00  0.00           C
ATOM   3255  HE1 TYR A 229      23.645  14.317  37.504  1.00  0.00           H
ATOM   3256  CZ  TYR A 229      22.101  15.816  37.739  1.00  0.00           C
ATOM   3257  OH  TYR A 229      22.702  16.393  38.816  1.00  0.00           O
ATOM   3258  HH  TYR A 229      22.294  17.228  39.088  1.00  0.00           H
ATOM   3259  CE2 TYR A 229      20.886  16.325  37.227  1.00  0.00           C
ATOM   3260  HE2 TYR A 229      20.420  17.181  37.692  1.00  0.00           H
ATOM   3261  CD2 TYR A 229      20.295  15.723  36.098  1.00  0.00           C
ATOM   3262  HD2 TYR A 229      19.377  16.119  35.693  1.00  0.00           H
ATOM   3263  C   TYR A 229      20.494  13.426  31.786  1.00  0.00           C
ATOM   3264  O   TYR A 229      20.155  14.067  30.788  1.00  0.00           O
ATOM   3265  N   LYS A 230      20.232  12.115  31.887  1.00  0.00           N
ATOM   3266  H   LYS A 230      20.539  11.605  32.709  1.00  0.00           H
ATOM   3267  CA  LYS A 230      19.344  11.441  30.926  1.00  0.00           C
ATOM   3268  HA  LYS A 230      19.680  11.703  29.923  1.00  0.00           H
ATOM   3269  CB  LYS A 230      19.438   9.904  31.045  1.00  0.00           C
ATOM   3270  HB1 LYS A 230      19.062   9.498  30.105  1.00  0.00           H
ATOM   3271  HB2 LYS A 230      20.483   9.607  31.130  1.00  0.00           H
ATOM   3272  CG  LYS A 230      18.634   9.273  32.202  1.00  0.00           C
ATOM   3273  HG1 LYS A 230      19.170   9.405  33.142  1.00  0.00           H
ATOM   3274  HG2 LYS A 230      17.665   9.760  32.297  1.00  0.00           H
ATOM   3275  CD  LYS A 230      18.372   7.780  31.952  1.00  0.00           C
ATOM   3276  HD1 LYS A 230      18.057   7.632  30.918  1.00  0.00           H
ATOM   3277  HD2 LYS A 230      19.286   7.218  32.131  1.00  0.00           H
ATOM   3278  CE  LYS A 230      17.256   7.286  32.876  1.00  0.00           C
ATOM   3279  HE1 LYS A 230      17.597   7.348  33.913  1.00  0.00           H
ATOM   3280  HE2 LYS A 230      16.396   7.948  32.759  1.00  0.00           H
ATOM   3281  NZ  LYS A 230      16.842   5.899  32.564  1.00  0.00           N
ATOM   3282  HZ1 LYS A 230      17.579   5.237  32.787  1.00  0.00           H
ATOM   3283  HZ2 LYS A 230      16.027   5.650  33.115  1.00  0.00           H
ATOM   3284  HZ3 LYS A 230      16.611   5.794  31.579  1.00  0.00           H
ATOM   3285  C   LYS A 230      17.890  11.937  31.038  1.00  0.00           C
ATOM   3286  O   LYS A 230      17.486  12.461  32.084  1.00  0.00           O
ATOM   3287  N   ARG A 231      17.070  11.695  30.016  1.00  0.00           N
ATOM   3288  H   ARG A 231      17.475  11.284  29.175  1.00  0.00           H
ATOM   3289  CA  ARG A 231      15.606  11.713  30.121  1.00  0.00           C
ATOM   3290  HA  ARG A 231      15.305  12.504  30.808  1.00  0.00           H
ATOM   3291  CB  ARG A 231      15.042  12.038  28.731  1.00  0.00           C
```

ATOM	3292	HB1	ARG	A	231	15.557	12.915	28.333	1.00	0.00	H
ATOM	3293	HB2	ARG	A	231	15.215	11.203	28.055	1.00	0.00	H
ATOM	3294	CG	ARG	A	231	13.549	12.341	28.796	1.00	0.00	C
ATOM	3295	HG1	ARG	A	231	13.011	11.454	29.129	1.00	0.00	H
ATOM	3296	HG2	ARG	A	231	13.387	13.152	29.508	1.00	0.00	H
ATOM	3297	CD	ARG	A	231	13.000	12.761	27.435	1.00	0.00	C
ATOM	3298	HD1	ARG	A	231	13.549	13.635	27.083	1.00	0.00	H
ATOM	3299	HD2	ARG	A	231	13.147	11.946	26.726	1.00	0.00	H
ATOM	3300	NE	ARG	A	231	11.577	13.100	27.577	1.00	0.00	N
ATOM	3301	HE	ARG	A	231	11.308	13.485	28.476	1.00	0.00	H
ATOM	3302	CZ	ARG	A	231	10.573	12.709	26.807	1.00	0.00	C
ATOM	3303	NH1	ARG	A	231	9.351	13.077	27.110	1.00	0.00	N
ATOM	3304	HH11	ARG	A	231	9.173	13.556	27.980	1.00	0.00	H
ATOM	3305	HH12	ARG	A	231	8.585	12.633	26.626	1.00	0.00	H
ATOM	3306	NH2	ARG	A	231	10.729	11.937	25.756	1.00	0.00	N
ATOM	3307	HH21	ARG	A	231	11.651	11.644	25.451	1.00	0.00	H
ATOM	3308	HH22	ARG	A	231	9.909	11.569	25.300	1.00	0.00	H
ATOM	3309	C	ARG	A	231	15.109	10.361	30.672	1.00	0.00	C
ATOM	3310	O	ARG	A	231	15.748	9.336	30.432	1.00	0.00	O
ATOM	3311	N	GLN	A	232	14.000	10.303	31.419	1.00	0.00	N
ATOM	3312	H	GLN	A	232	13.491	11.159	31.638	1.00	0.00	H
ATOM	3313	CA	GLN	A	232	13.533	9.025	31.989	1.00	0.00	C
ATOM	3314	HA	GLN	A	232	14.359	8.577	32.538	1.00	0.00	H
ATOM	3315	CB	GLN	A	232	12.371	9.234	32.975	1.00	0.00	C
ATOM	3316	HB1	GLN	A	232	11.631	9.897	32.526	1.00	0.00	H
ATOM	3317	HB2	GLN	A	232	11.896	8.270	33.161	1.00	0.00	H
ATOM	3318	CG	GLN	A	232	12.799	9.813	34.335	1.00	0.00	C
ATOM	3319	HG1	GLN	A	232	13.149	10.832	34.197	1.00	0.00	H
ATOM	3320	HG2	GLN	A	232	11.928	9.844	34.989	1.00	0.00	H
ATOM	3321	CD	GLN	A	232	13.899	9.006	35.026	1.00	0.00	C
ATOM	3322	OE1	GLN	A	232	13.704	7.907	35.531	1.00	0.00	O
ATOM	3323	NE2	GLN	A	232	15.129	9.471	35.025	1.00	0.00	N
ATOM	3324	HE21	GLN	A	232	15.815	8.981	35.582	1.00	0.00	H
ATOM	3325	HE22	GLN	A	232	15.333	10.411	34.700	1.00	0.00	H
ATOM	3326	C	GLN	A	232	13.194	7.973	30.921	1.00	0.00	C
ATOM	3327	O	GLN	A	232	13.533	6.810	31.119	1.00	0.00	O
ATOM	3328	N	GLU	A	233	12.657	8.363	29.760	1.00	0.00	N
ATOM	3329	H	GLU	A	233	12.228	9.285	29.718	1.00	0.00	H
ATOM	3330	CA	GLU	A	233	12.430	7.481	28.603	1.00	0.00	C
ATOM	3331	HA	GLU	A	233	11.842	6.630	28.946	1.00	0.00	H
ATOM	3332	CB	GLU	A	233	11.598	8.238	27.541	1.00	0.00	C
ATOM	3333	HB1	GLU	A	233	12.065	9.198	27.311	1.00	0.00	H
ATOM	3334	HB2	GLU	A	233	11.604	7.649	26.625	1.00	0.00	H
ATOM	3335	CG	GLU	A	233	10.115	8.443	27.892	1.00	0.00	C
ATOM	3336	HG1	GLU	A	233	9.553	8.409	26.957	1.00	0.00	H
ATOM	3337	HG2	GLU	A	233	9.757	7.618	28.504	1.00	0.00	H
ATOM	3338	CD	GLU	A	233	9.791	9.770	28.576	1.00	0.00	C
ATOM	3339	OE1	GLU	A	233	10.468	10.153	29.564	1.00	0.00	O
ATOM	3340	OE2	GLU	A	233	8.820	10.422	28.126	1.00	0.00	O
ATOM	3341	C	GLU	A	233	13.708	6.879	27.953	1.00	0.00	C
ATOM	3342	O	GLU	A	233	13.590	6.161	26.952	1.00	0.00	O
ATOM	3343	N	GLU	A	234	14.921	7.161	28.447	1.00	0.00	N
ATOM	3344	H	GLU	A	234	14.978	7.723	29.288	1.00	0.00	H
ATOM	3345	CA	GLU	A	234	16.194	6.714	27.851	1.00	0.00	C

ATOM	3346	HA	GLU	A	234	16.003	6.364	26.839	1.00	0.00	H
ATOM	3347	CB	GLU	A	234	17.182	7.889	27.733	1.00	0.00	C
ATOM	3348	HB1	GLU	A	234	17.334	8.331	28.715	1.00	0.00	H
ATOM	3349	HB2	GLU	A	234	18.144	7.520	27.374	1.00	0.00	H
ATOM	3350	CG	GLU	A	234	16.681	8.948	26.743	1.00	0.00	C
ATOM	3351	HG1	GLU	A	234	16.607	8.491	25.755	1.00	0.00	H
ATOM	3352	HG2	GLU	A	234	15.687	9.284	27.035	1.00	0.00	H
ATOM	3353	CD	GLU	A	234	17.598	10.165	26.659	1.00	0.00	C
ATOM	3354	OE1	GLU	A	234	18.196	10.566	27.682	1.00	0.00	O
ATOM	3355	OE2	GLU	A	234	17.688	10.754	25.557	1.00	0.00	O
ATOM	3356	C	GLU	A	234	16.861	5.551	28.608	1.00	0.00	C
ATOM	3357	O	GLU	A	234	16.624	5.321	29.797	1.00	0.00	O
ATOM	3358	N	SER	A	235	17.750	4.839	27.919	1.00	0.00	N
ATOM	3359	H	SER	A	235	17.894	5.082	26.939	1.00	0.00	H
ATOM	3360	CA	SER	A	235	18.562	3.720	28.419	1.00	0.00	C
ATOM	3361	HA	SER	A	235	18.865	3.934	29.444	1.00	0.00	H
ATOM	3362	CB	SER	A	235	17.714	2.429	28.453	1.00	0.00	C
ATOM	3363	HB1	SER	A	235	18.273	1.636	28.944	1.00	0.00	H
ATOM	3364	HB2	SER	A	235	16.828	2.621	29.062	1.00	0.00	H
ATOM	3365	OG	SER	A	235	17.287	1.970	27.175	1.00	0.00	O
ATOM	3366	HG	SER	A	235	18.011	1.490	26.730	1.00	0.00	H
ATOM	3367	C	SER	A	235	19.861	3.573	27.590	1.00	0.00	C
ATOM	3368	O	SER	A	235	20.195	4.458	26.797	1.00	0.00	O
ATOM	3369	N	GLY	A	236	20.616	2.478	27.758	1.00	0.00	N
ATOM	3370	H	GLY	A	236	20.345	1.797	28.460	1.00	0.00	H
ATOM	3371	CA	GLY	A	236	21.761	2.140	26.898	1.00	0.00	C
ATOM	3372	HA1	GLY	A	236	22.174	1.179	27.196	1.00	0.00	H
ATOM	3373	HA2	GLY	A	236	21.414	2.061	25.868	1.00	0.00	H
ATOM	3374	C	GLY	A	236	22.885	3.175	26.930	1.00	0.00	C
ATOM	3375	O	GLY	A	236	23.134	3.803	27.960	1.00	0.00	O
ATOM	3376	N	ALA	A	237	23.579	3.350	25.803	1.00	0.00	N
ATOM	3377	H	ALA	A	237	23.330	2.795	24.991	1.00	0.00	H
ATOM	3378	CA	ALA	A	237	24.584	4.402	25.629	1.00	0.00	C
ATOM	3379	HA	ALA	A	237	25.223	4.434	26.514	1.00	0.00	H
ATOM	3380	CB	ALA	A	237	25.449	4.037	24.416	1.00	0.00	C
ATOM	3381	HB1	ALA	A	237	26.216	4.795	24.265	1.00	0.00	H
ATOM	3382	HB2	ALA	A	237	25.941	3.079	24.589	1.00	0.00	H
ATOM	3383	HB3	ALA	A	237	24.832	3.973	23.518	1.00	0.00	H
ATOM	3384	C	ALA	A	237	23.950	5.800	25.476	1.00	0.00	C
ATOM	3385	O	ALA	A	237	24.583	6.800	25.832	1.00	0.00	O
ATOM	3386	N	ALA	A	238	22.702	5.879	24.993	1.00	0.00	N
ATOM	3387	H	ALA	A	238	22.242	5.018	24.711	1.00	0.00	H
ATOM	3388	CA	ALA	A	238	21.949	7.127	24.855	1.00	0.00	C
ATOM	3389	HA	ALA	A	238	22.513	7.797	24.210	1.00	0.00	H
ATOM	3390	CB	ALA	A	238	20.610	6.826	24.173	1.00	0.00	C
ATOM	3391	HB1	ALA	A	238	20.039	7.750	24.067	1.00	0.00	H
ATOM	3392	HB2	ALA	A	238	20.781	6.404	23.182	1.00	0.00	H
ATOM	3393	HB3	ALA	A	238	20.033	6.122	24.771	1.00	0.00	H
ATOM	3394	C	ALA	A	238	21.759	7.847	26.199	1.00	0.00	C
ATOM	3395	O	ALA	A	238	21.914	9.065	26.263	1.00	0.00	O
ATOM	3396	N	ALA	A	239	21.548	7.100	27.288	1.00	0.00	N
ATOM	3397	H	ALA	A	239	21.367	6.110	27.146	1.00	0.00	H
ATOM	3398	CA	ALA	A	239	21.506	7.612	28.664	1.00	0.00	C
ATOM	3399	HA	ALA	A	239	20.756	8.405	28.706	1.00	0.00	H

```
ATOM   3400  CB  ALA A 239      21.042   6.467  29.569  1.00  0.00           C
ATOM   3401  HB1 ALA A 239      21.009   6.814  30.601  1.00  0.00           H
ATOM   3402  HB2 ALA A 239      20.046   6.143  29.272  1.00  0.00           H
ATOM   3403  HB3 ALA A 239      21.739   5.629  29.503  1.00  0.00           H
ATOM   3404  C   ALA A 239      22.825   8.244  29.174  1.00  0.00           C
ATOM   3405  O   ALA A 239      22.864   8.779  30.285  1.00  0.00           O
ATOM   3406  N   SER A 240      23.901   8.224  28.383  1.00  0.00           N
ATOM   3407  H   SER A 240      23.839   7.704  27.517  1.00  0.00           H
ATOM   3408  CA  SER A 240      25.171   8.908  28.679  1.00  0.00           C
ATOM   3409  HA  SER A 240      25.123   9.328  29.681  1.00  0.00           H
ATOM   3410  CB  SER A 240      26.321   7.893  28.679  1.00  0.00           C
ATOM   3411  HB1 SER A 240      26.338   7.345  27.737  1.00  0.00           H
ATOM   3412  HB2 SER A 240      27.269   8.417  28.798  1.00  0.00           H
ATOM   3413  OG  SER A 240      26.166   6.990  29.756  1.00  0.00           O
ATOM   3414  HG  SER A 240      27.028   6.551  29.917  1.00  0.00           H
ATOM   3415  C   SER A 240      25.476  10.096  27.741  1.00  0.00           C
ATOM   3416  O   SER A 240      26.572  10.664  27.794  1.00  0.00           O
ATOM   3417  N   LEU A 241      24.524  10.503  26.891  1.00  0.00           N
ATOM   3418  H   LEU A 241      23.637  10.009  26.891  1.00  0.00           H
ATOM   3419  CA  LEU A 241      24.631  11.668  26.000  1.00  0.00           C
ATOM   3420  HA  LEU A 241      25.670  11.783  25.694  1.00  0.00           H
ATOM   3421  CB  LEU A 241      23.772  11.425  24.741  1.00  0.00           C
ATOM   3422  HB1 LEU A 241      22.732  11.306  25.053  1.00  0.00           H
ATOM   3423  HB2 LEU A 241      23.815  12.306  24.104  1.00  0.00           H
ATOM   3424  CG  LEU A 241      24.194  10.205  23.899  1.00  0.00           C
ATOM   3425  HG  LEU A 241      24.203   9.316  24.528  1.00  0.00           H
ATOM   3426  CD1 LEU A 241      23.163   9.986  22.785  1.00  0.00           C
ATOM   3427 HD11 LEU A 241      23.392   9.066  22.249  1.00  0.00           H
ATOM   3428 HD12 LEU A 241      22.170   9.884  23.222  1.00  0.00           H
ATOM   3429 HD13 LEU A 241      23.166  10.829  22.093  1.00  0.00           H
ATOM   3430  CD2 LEU A 241      25.592  10.374  23.281  1.00  0.00           C
ATOM   3431 HD21 LEU A 241      25.822   9.516  22.649  1.00  0.00           H
ATOM   3432 HD22 LEU A 241      25.631  11.283  22.680  1.00  0.00           H
ATOM   3433 HD23 LEU A 241      26.347  10.427  24.065  1.00  0.00           H
ATOM   3434  C   LEU A 241      24.268  12.971  26.740  1.00  0.00           C
ATOM   3435  O   LEU A 241      23.399  13.730  26.323  1.00  0.00           O
ATOM   3436  N   ASN A 242      24.931  13.221  27.869  1.00  0.00           N
ATOM   3437  H   ASN A 242      25.666  12.579  28.137  1.00  0.00           H
ATOM   3438  CA  ASN A 242      24.610  14.313  28.790  1.00  0.00           C
ATOM   3439  HA  ASN A 242      24.337  15.184  28.194  1.00  0.00           H
ATOM   3440  CB  ASN A 242      23.382  13.929  29.635  1.00  0.00           C
ATOM   3441  HB1 ASN A 242      23.199  14.684  30.395  1.00  0.00           H
ATOM   3442  HB2 ASN A 242      22.506  13.907  28.987  1.00  0.00           H
ATOM   3443  CG  ASN A 242      23.513  12.570  30.308  1.00  0.00           C
ATOM   3444  OD1 ASN A 242      24.251  12.383  31.269  1.00  0.00           O
ATOM   3445  ND2 ASN A 242      22.800  11.579  29.813  1.00  0.00           N
ATOM   3446 HD21 ASN A 242      22.171  11.727  29.026  1.00  0.00           H
ATOM   3447 HD22 ASN A 242      22.840  10.658  30.225  1.00  0.00           H
ATOM   3448  C   ASN A 242      25.813  14.737  29.653  1.00  0.00           C
ATOM   3449  O   ASN A 242      26.796  14.003  29.790  1.00  0.00           O
ATOM   3450  N   VAL A 243      25.738  15.951  30.210  1.00  0.00           N
ATOM   3451  H   VAL A 243      24.860  16.456  30.091  1.00  0.00           H
ATOM   3452  CA  VAL A 243      26.835  16.617  30.940  1.00  0.00           C
ATOM   3453  HA  VAL A 243      27.668  16.749  30.248  1.00  0.00           H
```

ATOM	3454	CB	VAL	A	243	26.398	18.013	31.448	1.00	0.00	C
ATOM	3455	HB	VAL	A	243	25.466	17.884	31.984	1.00	0.00	H
ATOM	3456	CG1	VAL	A	243	27.405	18.645	32.424	1.00	0.00	C
ATOM	3457	HG11	VAL	A	243	27.138	19.684	32.621	1.00	0.00	H
ATOM	3458	HG12	VAL	A	243	27.400	18.110	33.375	1.00	0.00	H
ATOM	3459	HG13	VAL	A	243	28.405	18.596	31.997	1.00	0.00	H
ATOM	3460	CG2	VAL	A	243	26.134	19.003	30.298	1.00	0.00	C
ATOM	3461	HG21	VAL	A	243	25.624	19.886	30.685	1.00	0.00	H
ATOM	3462	HG22	VAL	A	243	27.071	19.318	29.846	1.00	0.00	H
ATOM	3463	HG23	VAL	A	243	25.509	18.554	29.528	1.00	0.00	H
ATOM	3464	C	VAL	A	243	27.317	15.767	32.123	1.00	0.00	C
ATOM	3465	O	VAL	A	243	28.523	15.608	32.305	1.00	0.00	O
ATOM	3466	N	ASP	A	244	26.397	15.195	32.903	1.00	0.00	N
ATOM	3467	H	ASP	A	244	25.410	15.359	32.715	1.00	0.00	H
ATOM	3468	CA	ASP	A	244	26.718	14.389	34.080	1.00	0.00	C
ATOM	3469	HA	ASP	A	244	27.312	14.996	34.761	1.00	0.00	H
ATOM	3470	CB	ASP	A	244	25.415	13.999	34.784	1.00	0.00	C
ATOM	3471	HB1	ASP	A	244	24.939	14.899	35.176	1.00	0.00	H
ATOM	3472	HB2	ASP	A	244	24.739	13.534	34.064	1.00	0.00	H
ATOM	3473	CG	ASP	A	244	25.670	13.024	35.929	1.00	0.00	C
ATOM	3474	OD1	ASP	A	244	26.337	13.413	36.912	1.00	0.00	O
ATOM	3475	OD2	ASP	A	244	25.214	11.862	35.835	1.00	0.00	O
ATOM	3476	C	ASP	A	244	27.546	13.149	33.726	1.00	0.00	C
ATOM	3477	O	ASP	A	244	28.607	12.936	34.313	1.00	0.00	O
ATOM	3478	N	ALA	A	245	27.110	12.375	32.726	1.00	0.00	N
ATOM	3479	H	ALA	A	245	26.233	12.609	32.276	1.00	0.00	H
ATOM	3480	CA	ALA	A	245	27.864	11.221	32.239	1.00	0.00	C
ATOM	3481	HA	ALA	A	245	28.025	10.526	33.066	1.00	0.00	H
ATOM	3482	CB	ALA	A	245	27.044	10.519	31.159	1.00	0.00	C
ATOM	3483	HB1	ALA	A	245	27.623	9.692	30.748	1.00	0.00	H
ATOM	3484	HB2	ALA	A	245	26.132	10.121	31.598	1.00	0.00	H
ATOM	3485	HB3	ALA	A	245	26.794	11.221	30.364	1.00	0.00	H
ATOM	3486	C	ALA	A	245	29.241	11.629	31.706	1.00	0.00	C
ATOM	3487	O	ALA	A	245	30.232	10.958	31.988	1.00	0.00	O
ATOM	3488	N	ALA	A	246	29.325	12.756	30.993	1.00	0.00	N
ATOM	3489	H	ALA	A	246	28.462	13.241	30.767	1.00	0.00	H
ATOM	3490	CA	ALA	A	246	30.586	13.279	30.474	1.00	0.00	C
ATOM	3491	HA	ALA	A	246	31.077	12.488	29.905	1.00	0.00	H
ATOM	3492	CB	ALA	A	246	30.263	14.425	29.516	1.00	0.00	C
ATOM	3493	HB1	ALA	A	246	31.179	14.765	29.034	1.00	0.00	H
ATOM	3494	HB2	ALA	A	246	29.567	14.062	28.760	1.00	0.00	H
ATOM	3495	HB3	ALA	A	246	29.807	15.259	30.050	1.00	0.00	H
ATOM	3496	C	ALA	A	246	31.556	13.700	31.591	1.00	0.00	C
ATOM	3497	O	ALA	A	246	32.736	13.364	31.522	1.00	0.00	O
ATOM	3498	N	VAL	A	247	31.064	14.383	32.629	1.00	0.00	N
ATOM	3499	H	VAL	A	247	30.076	14.628	32.601	1.00	0.00	H
ATOM	3500	CA	VAL	A	247	31.871	14.808	33.786	1.00	0.00	C
ATOM	3501	HA	VAL	A	247	32.780	15.275	33.401	1.00	0.00	H
ATOM	3502	CB	VAL	A	247	31.130	15.864	34.641	1.00	0.00	C
ATOM	3503	HB	VAL	A	247	30.106	15.526	34.805	1.00	0.00	H
ATOM	3504	CG1	VAL	A	247	31.789	16.098	36.014	1.00	0.00	C
ATOM	3505	HG11	VAL	A	247	31.290	16.911	36.538	1.00	0.00	H
ATOM	3506	HG12	VAL	A	247	31.703	15.205	36.634	1.00	0.00	H
ATOM	3507	HG13	VAL	A	247	32.843	16.350	35.887	1.00	0.00	H

```
ATOM   3508  CG2 VAL A 247      31.094  17.208  33.887  1.00  0.00           C
ATOM   3509 HG21 VAL A 247      30.515  17.939  34.450  1.00  0.00           H
ATOM   3510 HG22 VAL A 247      32.107  17.589  33.752  1.00  0.00           H
ATOM   3511 HG23 VAL A 247      30.633  17.086  32.907  1.00  0.00           H
ATOM   3512  C   VAL A 247      32.298  13.600  34.629  1.00  0.00           C
ATOM   3513  O   VAL A 247      33.479  13.489  34.952  1.00  0.00           O
ATOM   3514  N   GLN A 248      31.382  12.676  34.954  1.00  0.00           N
ATOM   3515  H   GLN A 248      30.415  12.806  34.665  1.00  0.00           H
ATOM   3516  CA  GLN A 248      31.733  11.456  35.688  1.00  0.00           C
ATOM   3517  HA  GLN A 248      32.255  11.737  36.605  1.00  0.00           H
ATOM   3518  CB  GLN A 248      30.476  10.652  36.080  1.00  0.00           C
ATOM   3519  HB1 GLN A 248      29.822  10.536  35.214  1.00  0.00           H
ATOM   3520  HB2 GLN A 248      30.798   9.657  36.394  1.00  0.00           H
ATOM   3521  CG  GLN A 248      29.691  11.281  37.250  1.00  0.00           C
ATOM   3522  HG1 GLN A 248      30.376  11.503  38.068  1.00  0.00           H
ATOM   3523  HG2 GLN A 248      29.235  12.216  36.923  1.00  0.00           H
ATOM   3524  CD  GLN A 248      28.607  10.337  37.782  1.00  0.00           C
ATOM   3525  OE1 GLN A 248      28.865   9.401  38.534  1.00  0.00           O
ATOM   3526  NE2 GLN A 248      27.357  10.521  37.426  1.00  0.00           N
ATOM   3527 HE21 GLN A 248      27.064  11.333  36.893  1.00  0.00           H
ATOM   3528 HE22 GLN A 248      26.697   9.781  37.617  1.00  0.00           H
ATOM   3529  C   GLN A 248      32.730  10.600  34.892  1.00  0.00           C
ATOM   3530  O   GLN A 248      33.699  10.131  35.483  1.00  0.00           O
ATOM   3531  N   GLN A 249      32.562  10.458  33.570  1.00  0.00           N
ATOM   3532  H   GLN A 249      31.732  10.851  33.136  1.00  0.00           H
ATOM   3533  CA  GLN A 249      33.507   9.752  32.698  1.00  0.00           C
ATOM   3534  HA  GLN A 249      33.597   8.721  33.047  1.00  0.00           H
ATOM   3535  CB  GLN A 249      32.973   9.740  31.249  1.00  0.00           C
ATOM   3536  HB1 GLN A 249      32.055   9.151  31.220  1.00  0.00           H
ATOM   3537  HB2 GLN A 249      32.744  10.762  30.948  1.00  0.00           H
ATOM   3538  CG  GLN A 249      33.977   9.145  30.243  1.00  0.00           C
ATOM   3539  HG1 GLN A 249      34.866   9.774  30.207  1.00  0.00           H
ATOM   3540  HG2 GLN A 249      34.263   8.155  30.593  1.00  0.00           H
ATOM   3541  CD  GLN A 249      33.462   9.018  28.806  1.00  0.00           C
ATOM   3542  OE1 GLN A 249      33.898   8.156  28.054  1.00  0.00           O
ATOM   3543  NE2 GLN A 249      32.557   9.860  28.351  1.00  0.00           N
ATOM   3544 HE21 GLN A 249      32.169  10.576  28.940  1.00  0.00           H
ATOM   3545 HE22 GLN A 249      32.251   9.749  27.402  1.00  0.00           H
ATOM   3546  C   GLN A 249      34.911  10.358  32.770  1.00  0.00           C
ATOM   3547  O   GLN A 249      35.859   9.632  33.055  1.00  0.00           O
ATOM   3548  N   TRP A 250      35.078  11.660  32.509  1.00  0.00           N
ATOM   3549  H   TRP A 250      34.274  12.235  32.276  1.00  0.00           H
ATOM   3550  CA  TRP A 250      36.414  12.263  32.460  1.00  0.00           C
ATOM   3551  HA  TRP A 250      37.046  11.618  31.846  1.00  0.00           H
ATOM   3552  CB  TRP A 250      36.351  13.629  31.760  1.00  0.00           C
ATOM   3553  HB1 TRP A 250      35.429  14.149  32.027  1.00  0.00           H
ATOM   3554  HB2 TRP A 250      37.186  14.241  32.103  1.00  0.00           H
ATOM   3555  CG  TRP A 250      36.461  13.515  30.268  1.00  0.00           C
ATOM   3556  CD1 TRP A 250      35.453  13.214  29.420  1.00  0.00           C
ATOM   3557  HD1 TRP A 250      34.422  13.070  29.713  1.00  0.00           H
ATOM   3558  NE1 TRP A 250      35.947  13.096  28.135  1.00  0.00           N
ATOM   3559  HE1 TRP A 250      35.365  12.977  27.311  1.00  0.00           H
ATOM   3560  CE2 TRP A 250      37.314  13.257  28.097  1.00  0.00           C
ATOM   3561  CZ2 TRP A 250      38.266  13.170  27.070  1.00  0.00           C
```

ATOM	3562	HZ2	TRP	A	250	37.967	12.904	26.068	1.00	0.00	H
ATOM	3563	CH2	TRP	A	250	39.610	13.461	27.355	1.00	0.00	C
ATOM	3564	HH2	TRP	A	250	40.355	13.413	26.574	1.00	0.00	H
ATOM	3565	CZ3	TRP	A	250	39.977	13.860	28.651	1.00	0.00	C
ATOM	3566	HZ3	TRP	A	250	41.006	14.112	28.870	1.00	0.00	H
ATOM	3567	CE3	TRP	A	250	39.011	13.933	29.671	1.00	0.00	C
ATOM	3568	HE3	TRP	A	250	39.306	14.254	30.654	1.00	0.00	H
ATOM	3569	CD2	TRP	A	250	37.661	13.605	29.434	1.00	0.00	C
ATOM	3570	C	TRP	A	250	37.102	12.308	33.833	1.00	0.00	C
ATOM	3571	O	TRP	A	250	38.326	12.159	33.892	1.00	0.00	O
ATOM	3572	N	LEU	A	251	36.345	12.443	34.931	1.00	0.00	N
ATOM	3573	H	LEU	A	251	35.344	12.552	34.808	1.00	0.00	H
ATOM	3574	CA	LEU	A	251	36.875	12.310	36.294	1.00	0.00	C
ATOM	3575	HA	LEU	A	251	37.769	12.926	36.396	1.00	0.00	H
ATOM	3576	CB	LEU	A	251	35.823	12.767	37.325	1.00	0.00	C
ATOM	3577	HB1	LEU	A	251	34.889	12.238	37.128	1.00	0.00	H
ATOM	3578	HB2	LEU	A	251	36.166	12.461	38.315	1.00	0.00	H
ATOM	3579	CG	LEU	A	251	35.541	14.282	37.378	1.00	0.00	C
ATOM	3580	HG	LEU	A	251	35.221	14.624	36.397	1.00	0.00	H
ATOM	3581	CD1	LEU	A	251	34.399	14.536	38.374	1.00	0.00	C
ATOM	3582	HD11	LEU	A	251	34.157	15.598	38.403	1.00	0.00	H
ATOM	3583	HD12	LEU	A	251	33.509	13.987	38.064	1.00	0.00	H
ATOM	3584	HD13	LEU	A	251	34.690	14.210	39.373	1.00	0.00	H
ATOM	3585	CD2	LEU	A	251	36.776	15.098	37.789	1.00	0.00	C
ATOM	3586	HD21	LEU	A	251	36.500	16.144	37.919	1.00	0.00	H
ATOM	3587	HD22	LEU	A	251	37.185	14.719	38.726	1.00	0.00	H
ATOM	3588	HD23	LEU	A	251	37.538	15.044	37.013	1.00	0.00	H
ATOM	3589	C	LEU	A	251	37.310	10.868	36.587	1.00	0.00	C
ATOM	3590	O	LEU	A	251	38.440	10.656	37.028	1.00	0.00	O
ATOM	3591	N	GLN	A	252	36.451	9.876	36.339	1.00	0.00	N
ATOM	3592	H	GLN	A	252	35.542	10.100	35.939	1.00	0.00	H
ATOM	3593	CA	GLN	A	252	36.732	8.474	36.664	1.00	0.00	C
ATOM	3594	HA	GLN	A	252	37.087	8.417	37.695	1.00	0.00	H
ATOM	3595	CB	GLN	A	252	35.442	7.648	36.532	1.00	0.00	C
ATOM	3596	HB1	GLN	A	252	34.967	7.865	35.575	1.00	0.00	H
ATOM	3597	HB2	GLN	A	252	35.691	6.588	36.535	1.00	0.00	H
ATOM	3598	CG	GLN	A	252	34.454	7.926	37.680	1.00	0.00	C
ATOM	3599	HG1	GLN	A	252	34.285	8.997	37.786	1.00	0.00	H
ATOM	3600	HG2	GLN	A	252	33.500	7.458	37.436	1.00	0.00	H
ATOM	3601	CD	GLN	A	252	34.948	7.364	39.011	1.00	0.00	C
ATOM	3602	OE1	GLN	A	252	35.488	8.064	39.863	1.00	0.00	O
ATOM	3603	NE2	GLN	A	252	34.814	6.076	39.221	1.00	0.00	N
ATOM	3604	HE21	GLN	A	252	34.415	5.485	38.513	1.00	0.00	H
ATOM	3605	HE22	GLN	A	252	35.143	5.681	40.095	1.00	0.00	H
ATOM	3606	C	GLN	A	252	37.852	7.891	35.796	1.00	0.00	C
ATOM	3607	O	GLN	A	252	38.697	7.153	36.304	1.00	0.00	O
ATOM	3608	N	LYS	A	253	37.940	8.279	34.523	1.00	0.00	N
ATOM	3609	H	LYS	A	253	37.192	8.849	34.132	1.00	0.00	H
ATOM	3610	CA	LYS	A	253	39.053	7.921	33.637	1.00	0.00	C
ATOM	3611	HA	LYS	A	253	39.306	6.876	33.824	1.00	0.00	H
ATOM	3612	CB	LYS	A	253	38.593	8.019	32.168	1.00	0.00	C
ATOM	3613	HB1	LYS	A	253	38.203	9.017	31.967	1.00	0.00	H
ATOM	3614	HB2	LYS	A	253	39.448	7.844	31.515	1.00	0.00	H
ATOM	3615	CG	LYS	A	253	37.525	6.958	31.842	1.00	0.00	C

ATOM	3616	HG1	LYS	A	253	37.933	5.968	32.056	1.00	0.00	H
ATOM	3617	HG2	LYS	A	253	36.647	7.111	32.471	1.00	0.00	H
ATOM	3618	CD	LYS	A	253	37.092	7.008	30.371	1.00	0.00	C
ATOM	3619	HD1	LYS	A	253	36.677	7.992	30.151	1.00	0.00	H
ATOM	3620	HD2	LYS	A	253	37.960	6.840	29.733	1.00	0.00	H
ATOM	3621	CE	LYS	A	253	36.039	5.918	30.121	1.00	0.00	C
ATOM	3622	HE1	LYS	A	253	36.486	4.942	30.338	1.00	0.00	H
ATOM	3623	HE2	LYS	A	253	35.209	6.064	30.818	1.00	0.00	H
ATOM	3624	NZ	LYS	A	253	35.522	5.931	28.732	1.00	0.00	N
ATOM	3625	HZ1	LYS	A	253	34.790	5.240	28.621	1.00	0.00	H
ATOM	3626	HZ2	LYS	A	253	35.113	6.828	28.482	1.00	0.00	H
ATOM	3627	HZ3	LYS	A	253	36.245	5.682	28.061	1.00	0.00	H
ATOM	3628	C	LYS	A	253	40.354	8.704	33.939	1.00	0.00	C
ATOM	3629	O	LYS	A	253	41.323	8.569	33.194	1.00	0.00	O
ATOM	3630	N	GLY	A	254	40.424	9.469	35.039	1.00	0.00	N
ATOM	3631	H	GLY	A	254	39.588	9.538	35.607	1.00	0.00	H
ATOM	3632	CA	GLY	A	254	41.687	9.934	35.639	1.00	0.00	C
ATOM	3633	HA1	GLY	A	254	41.631	9.763	36.715	1.00	0.00	H
ATOM	3634	HA2	GLY	A	254	42.528	9.364	35.241	1.00	0.00	H
ATOM	3635	C	GLY	A	254	42.019	11.416	35.466	1.00	0.00	C
ATOM	3636	O	GLY	A	254	43.147	11.803	35.757	1.00	0.00	O
ATOM	3637	N	THR	A	255	41.083	12.265	35.019	1.00	0.00	N
ATOM	3638	H	THR	A	255	40.157	11.910	34.818	1.00	0.00	H
ATOM	3639	CA	THR	A	255	41.323	13.722	34.946	1.00	0.00	C
ATOM	3640	HA	THR	A	255	42.338	13.873	34.586	1.00	0.00	H
ATOM	3641	CB	THR	A	255	40.392	14.440	33.971	1.00	0.00	C
ATOM	3642	HB	THR	A	255	39.376	14.481	34.370	1.00	0.00	H
ATOM	3643	CG2	THR	A	255	40.876	15.858	33.667	1.00	0.00	C
ATOM	3644	HG21	THR	A	255	40.252	16.297	32.890	1.00	0.00	H
ATOM	3645	HG22	THR	A	255	40.811	16.477	34.560	1.00	0.00	H
ATOM	3646	HG23	THR	A	255	41.911	15.836	33.323	1.00	0.00	H
ATOM	3647	OG1	THR	A	255	40.399	13.726	32.768	1.00	0.00	O
ATOM	3648	HG1	THR	A	255	39.803	12.972	32.896	1.00	0.00	H
ATOM	3649	C	THR	A	255	41.156	14.334	36.337	1.00	0.00	C
ATOM	3650	O	THR	A	255	40.102	14.112	36.939	1.00	0.00	O
ATOM	3651	N	PRO	A	256	42.123	15.121	36.845	1.00	0.00	N
ATOM	3652	CD	PRO	A	256	43.476	15.269	36.339	1.00	0.00	C
ATOM	3653	HD1	PRO	A	256	43.492	15.985	35.517	1.00	0.00	H
ATOM	3654	HD2	PRO	A	256	43.870	14.307	36.029	1.00	0.00	H
ATOM	3655	CG	PRO	A	256	44.303	15.782	37.509	1.00	0.00	C
ATOM	3656	HG1	PRO	A	256	45.141	16.393	37.179	1.00	0.00	H
ATOM	3657	HG2	PRO	A	256	44.653	14.934	38.097	1.00	0.00	H
ATOM	3658	CB	PRO	A	256	43.298	16.585	38.324	1.00	0.00	C
ATOM	3659	HB1	PRO	A	256	43.251	17.604	37.942	1.00	0.00	H
ATOM	3660	HB2	PRO	A	256	43.571	16.588	39.376	1.00	0.00	H
ATOM	3661	CA	PRO	A	256	41.966	15.864	38.084	1.00	0.00	C
ATOM	3662	HA	PRO	A	256	41.786	15.150	38.885	1.00	0.00	H
ATOM	3663	C	PRO	A	256	40.804	16.853	37.990	1.00	0.00	C
ATOM	3664	O	PRO	A	256	40.675	17.570	36.997	1.00	0.00	O
ATOM	3665	N	ALA	A	257	39.990	16.936	39.046	1.00	0.00	N
ATOM	3666	H	ALA	A	257	40.154	16.326	39.836	1.00	0.00	H
ATOM	3667	CA	ALA	A	257	38.917	17.927	39.146	1.00	0.00	C
ATOM	3668	HA	ALA	A	257	38.250	17.812	38.290	1.00	0.00	H
ATOM	3669	CB	ALA	A	257	38.126	17.649	40.429	1.00	0.00	C

ATOM	3670	HB1	ALA	A	257	37.278	18.331	40.492	1.00	0.00	H
ATOM	3671	HB2	ALA	A	257	37.751	16.624	40.424	1.00	0.00	H
ATOM	3672	HB3	ALA	A	257	38.767	17.797	41.298	1.00	0.00	H
ATOM	3673	C	ALA	A	257	39.461	19.368	39.108	1.00	0.00	C
ATOM	3674	O	ALA	A	257	38.815	20.259	38.564	1.00	0.00	O
ATOM	3675	N	SER	A	258	40.681	19.588	39.602	1.00	0.00	N
ATOM	3676	H	SER	A	258	41.159	18.808	40.046	1.00	0.00	H
ATOM	3677	CA	SER	A	258	41.377	20.880	39.523	1.00	0.00	C
ATOM	3678	HA	SER	A	258	40.714	21.655	39.906	1.00	0.00	H
ATOM	3679	CB	SER	A	258	42.621	20.826	40.426	1.00	0.00	C
ATOM	3680	HB1	SER	A	258	42.943	21.842	40.650	1.00	0.00	H
ATOM	3681	HB2	SER	A	258	42.366	20.342	41.370	1.00	0.00	H
ATOM	3682	OG	SER	A	258	43.692	20.133	39.800	1.00	0.00	O
ATOM	3683	HG	SER	A	258	44.049	19.467	40.431	1.00	0.00	H
ATOM	3684	C	SER	A	258	41.768	21.286	38.084	1.00	0.00	C
ATOM	3685	O	SER	A	258	41.991	22.472	37.810	1.00	0.00	O
ATOM	3686	N	LYS	A	259	41.855	20.316	37.161	1.00	0.00	N
ATOM	3687	H	LYS	A	259	41.666	19.369	37.470	1.00	0.00	H
ATOM	3688	CA	LYS	A	259	42.186	20.510	35.743	1.00	0.00	C
ATOM	3689	HA	LYS	A	259	42.620	21.502	35.599	1.00	0.00	H
ATOM	3690	CB	LYS	A	259	43.231	19.458	35.311	1.00	0.00	C
ATOM	3691	HB1	LYS	A	259	42.848	18.455	35.506	1.00	0.00	H
ATOM	3692	HB2	LYS	A	259	43.385	19.551	34.235	1.00	0.00	H
ATOM	3693	CG	LYS	A	259	44.595	19.625	36.005	1.00	0.00	C
ATOM	3694	HG1	LYS	A	259	44.880	20.675	35.975	1.00	0.00	H
ATOM	3695	HG2	LYS	A	259	44.518	19.317	37.047	1.00	0.00	H
ATOM	3696	CD	LYS	A	259	45.680	18.796	35.300	1.00	0.00	C
ATOM	3697	HD1	LYS	A	259	45.366	17.753	35.248	1.00	0.00	H
ATOM	3698	HD2	LYS	A	259	45.797	19.171	34.283	1.00	0.00	H
ATOM	3699	CE	LYS	A	259	47.026	18.884	36.036	1.00	0.00	C
ATOM	3700	HE1	LYS	A	259	47.302	19.933	36.153	1.00	0.00	H
ATOM	3701	HE2	LYS	A	259	46.912	18.447	37.032	1.00	0.00	H
ATOM	3702	NZ	LYS	A	259	48.103	18.179	35.301	1.00	0.00	N
ATOM	3703	HZ1	LYS	A	259	48.992	18.227	35.787	1.00	0.00	H
ATOM	3704	HZ2	LYS	A	259	47.853	17.207	35.149	1.00	0.00	H
ATOM	3705	HZ3	LYS	A	259	48.233	18.598	34.384	1.00	0.00	H
ATOM	3706	C	LYS	A	259	40.960	20.433	34.811	1.00	0.00	C
ATOM	3707	O	LYS	A	259	41.023	20.962	33.699	1.00	0.00	O
ATOM	3708	N	LEU	A	260	39.865	19.772	35.206	1.00	0.00	N
ATOM	3709	H	LEU	A	260	39.873	19.341	36.122	1.00	0.00	H
ATOM	3710	CA	LEU	A	260	38.677	19.588	34.362	1.00	0.00	C
ATOM	3711	HA	LEU	A	260	39.016	19.439	33.337	1.00	0.00	H
ATOM	3712	CB	LEU	A	260	37.931	18.313	34.807	1.00	0.00	C
ATOM	3713	HB1	LEU	A	260	38.636	17.482	34.828	1.00	0.00	H
ATOM	3714	HB2	LEU	A	260	37.574	18.462	35.827	1.00	0.00	H
ATOM	3715	CG	LEU	A	260	36.736	17.917	33.910	1.00	0.00	C
ATOM	3716	HG	LEU	A	260	36.006	18.727	33.907	1.00	0.00	H
ATOM	3717	CD1	LEU	A	260	37.159	17.633	32.459	1.00	0.00	C
ATOM	3718	HD11	LEU	A	260	36.307	17.254	31.895	1.00	0.00	H
ATOM	3719	HD12	LEU	A	260	37.506	18.546	31.976	1.00	0.00	H
ATOM	3720	HD13	LEU	A	260	37.952	16.885	32.436	1.00	0.00	H
ATOM	3721	CD2	LEU	A	260	36.054	16.666	34.482	1.00	0.00	C
ATOM	3722	HD21	LEU	A	260	35.192	16.398	33.870	1.00	0.00	H
ATOM	3723	HD22	LEU	A	260	36.753	15.829	34.501	1.00	0.00	H

```
ATOM   3724 HD23 LEU A 260      35.707  16.869  35.494  1.00  0.00           H
ATOM   3725  C   LEU A 260      37.785  20.839  34.383  1.00  0.00           C
ATOM   3726  O   LEU A 260      37.375  21.304  35.446  1.00  0.00           O
ATOM   3727  N   ILE A 261      37.485  21.380  33.201  1.00  0.00           N
ATOM   3728  H   ILE A 261      37.847  20.936  32.363  1.00  0.00           H
ATOM   3729  CA  ILE A 261      36.707  22.610  32.985  1.00  0.00           C
ATOM   3730  HA  ILE A 261      36.381  23.015  33.944  1.00  0.00           H
ATOM   3731  CB  ILE A 261      37.576  23.671  32.258  1.00  0.00           C
ATOM   3732  HB  ILE A 261      37.688  23.357  31.218  1.00  0.00           H
ATOM   3733  CG2 ILE A 261      36.856  25.025  32.248  1.00  0.00           C
ATOM   3734 HG21 ILE A 261      37.419  25.738  31.647  1.00  0.00           H
ATOM   3735 HG22 ILE A 261      35.866  24.907  31.814  1.00  0.00           H
ATOM   3736 HG23 ILE A 261      36.755  25.415  33.258  1.00  0.00           H
ATOM   3737  CG1 ILE A 261      39.009  23.853  32.813  1.00  0.00           C
ATOM   3738 HG11 ILE A 261      39.561  22.927  32.666  1.00  0.00           H
ATOM   3739 HG12 ILE A 261      39.515  24.617  32.222  1.00  0.00           H
ATOM   3740  CD  ILE A 261      39.114  24.252  34.292  1.00  0.00           C
ATOM   3741  HD1 ILE A 261      40.162  24.238  34.591  1.00  0.00           H
ATOM   3742  HD2 ILE A 261      38.718  25.255  34.442  1.00  0.00           H
ATOM   3743  HD3 ILE A 261      38.568  23.551  34.918  1.00  0.00           H
ATOM   3744  C   ILE A 261      35.463  22.263  32.149  1.00  0.00           C
ATOM   3745  O   ILE A 261      35.587  21.663  31.078  1.00  0.00           O
ATOM   3746  N   LEU A 262      34.269  22.626  32.632  1.00  0.00           N
ATOM   3747  H   LEU A 262      34.239  23.174  33.482  1.00  0.00           H
ATOM   3748  CA  LEU A 262      32.989  22.259  32.009  1.00  0.00           C
ATOM   3749  HA  LEU A 262      33.109  21.290  31.522  1.00  0.00           H
ATOM   3750  CB  LEU A 262      31.927  22.121  33.121  1.00  0.00           C
ATOM   3751  HB1 LEU A 262      32.210  21.290  33.769  1.00  0.00           H
ATOM   3752  HB2 LEU A 262      31.933  23.033  33.720  1.00  0.00           H
ATOM   3753  CG  LEU A 262      30.483  21.893  32.619  1.00  0.00           C
ATOM   3754  HG  LEU A 262      30.170  22.756  32.031  1.00  0.00           H
ATOM   3755  CD1 LEU A 262      30.357  20.643  31.738  1.00  0.00           C
ATOM   3756 HD11 LEU A 262      29.335  20.572  31.368  1.00  0.00           H
ATOM   3757 HD12 LEU A 262      31.016  20.705  30.876  1.00  0.00           H
ATOM   3758 HD13 LEU A 262      30.604  19.750  32.313  1.00  0.00           H
ATOM   3759  CD2 LEU A 262      29.533  21.776  33.819  1.00  0.00           C
ATOM   3760 HD21 LEU A 262      28.506  21.669  33.468  1.00  0.00           H
ATOM   3761 HD22 LEU A 262      29.796  20.909  34.427  1.00  0.00           H
ATOM   3762 HD23 LEU A 262      29.597  22.675  34.431  1.00  0.00           H
ATOM   3763  C   LEU A 262      32.564  23.275  30.938  1.00  0.00           C
ATOM   3764  O   LEU A 262      32.408  24.454  31.252  1.00  0.00           O
ATOM   3765  N   GLY A 263      32.331  22.811  29.704  1.00  0.00           N
ATOM   3766  H   GLY A 263      32.516  21.836  29.512  1.00  0.00           H
ATOM   3767  CA  GLY A 263      31.902  23.628  28.563  1.00  0.00           C
ATOM   3768  HA1 GLY A 263      32.453  24.565  28.569  1.00  0.00           H
ATOM   3769  HA2 GLY A 263      32.128  23.102  27.637  1.00  0.00           H
ATOM   3770  C   GLY A 263      30.407  23.949  28.536  1.00  0.00           C
ATOM   3771  O   GLY A 263      29.560  23.075  28.741  1.00  0.00           O
ATOM   3772  N   MET A 264      30.097  25.204  28.209  1.00  0.00           N
ATOM   3773  H   MET A 264      30.867  25.861  28.123  1.00  0.00           H
ATOM   3774  CA  MET A 264      28.750  25.778  28.123  1.00  0.00           C
ATOM   3775  HA  MET A 264      28.011  25.032  28.403  1.00  0.00           H
ATOM   3776  CB  MET A 264      28.637  26.949  29.120  1.00  0.00           C
ATOM   3777  HB1 MET A 264      29.234  27.785  28.751  1.00  0.00           H
```

ATOM	3778	HB2	MET	A	264	27.597	27.269	29.164	1.00	0.00	H
ATOM	3779	CG	MET	A	264	29.105	26.641	30.551	1.00	0.00	C
ATOM	3780	HG1	MET	A	264	30.136	26.287	30.523	1.00	0.00	H
ATOM	3781	HG2	MET	A	264	29.110	27.579	31.102	1.00	0.00	H
ATOM	3782	SD	MET	A	264	28.114	25.450	31.485	1.00	0.00	S
ATOM	3783	CE	MET	A	264	29.095	25.467	33.006	1.00	0.00	C
ATOM	3784	HE1	MET	A	264	28.659	24.783	33.733	1.00	0.00	H
ATOM	3785	HE2	MET	A	264	30.116	25.154	32.783	1.00	0.00	H
ATOM	3786	HE3	MET	A	264	29.108	26.474	33.422	1.00	0.00	H
ATOM	3787	C	MET	A	264	28.485	26.293	26.688	1.00	0.00	C
ATOM	3788	O	MET	A	264	29.319	27.041	26.172	1.00	0.00	O
ATOM	3789	N	PRO	A	265	27.368	25.932	26.027	1.00	0.00	N
ATOM	3790	CD	PRO	A	265	26.469	24.846	26.397	1.00	0.00	C
ATOM	3791	HD1	PRO	A	265	25.903	25.094	27.296	1.00	0.00	H
ATOM	3792	HD2	PRO	A	265	27.045	23.933	26.550	1.00	0.00	H
ATOM	3793	CG	PRO	A	265	25.518	24.653	25.215	1.00	0.00	C
ATOM	3794	HG1	PRO	A	265	24.589	25.187	25.405	1.00	0.00	H
ATOM	3795	HG2	PRO	A	265	25.316	23.604	25.021	1.00	0.00	H
ATOM	3796	CB	PRO	A	265	26.253	25.296	24.041	1.00	0.00	C
ATOM	3797	HB1	PRO	A	265	25.561	25.649	23.276	1.00	0.00	H
ATOM	3798	HB2	PRO	A	265	26.958	24.578	23.619	1.00	0.00	H
ATOM	3799	CA	PRO	A	265	27.029	26.439	24.699	1.00	0.00	C
ATOM	3800	HA	PRO	A	265	27.928	26.642	24.116	1.00	0.00	H
ATOM	3801	C	PRO	A	265	26.172	27.711	24.790	1.00	0.00	C
ATOM	3802	O	PRO	A	265	25.090	27.689	25.375	1.00	0.00	O
ATOM	3803	N	THR	A	266	26.606	28.810	24.161	1.00	0.00	N
ATOM	3804	H	THR	A	266	27.514	28.796	23.704	1.00	0.00	H
ATOM	3805	CA	THR	A	266	25.804	30.046	24.022	1.00	0.00	C
ATOM	3806	HA	THR	A	266	25.117	30.106	24.864	1.00	0.00	H
ATOM	3807	CB	THR	A	266	26.688	31.300	24.089	1.00	0.00	C
ATOM	3808	HB	THR	A	266	26.107	32.178	23.798	1.00	0.00	H
ATOM	3809	CG2	THR	A	266	27.219	31.508	25.506	1.00	0.00	C
ATOM	3810	HG21	THR	A	266	27.932	32.330	25.521	1.00	0.00	H
ATOM	3811	HG22	THR	A	266	26.399	31.741	26.180	1.00	0.00	H
ATOM	3812	HG23	THR	A	266	27.711	30.602	25.851	1.00	0.00	H
ATOM	3813	OG1	THR	A	266	27.807	31.166	23.252	1.00	0.00	O
ATOM	3814	HG1	THR	A	266	28.180	32.048	23.118	1.00	0.00	H
ATOM	3815	C	THR	A	266	24.900	30.000	22.786	1.00	0.00	C
ATOM	3816	O	THR	A	266	24.733	30.979	22.068	1.00	0.00	O
ATOM	3817	N	TYR	A	267	24.304	28.835	22.526	1.00	0.00	N
ATOM	3818	H	TYR	A	267	24.470	28.073	23.169	1.00	0.00	H
ATOM	3819	CA	TYR	A	267	23.522	28.544	21.317	1.00	0.00	C
ATOM	3820	HA	TYR	A	267	22.906	29.416	21.088	1.00	0.00	H
ATOM	3821	CB	TYR	A	267	24.476	28.316	20.126	1.00	0.00	C
ATOM	3822	HB1	TYR	A	267	23.890	28.173	19.219	1.00	0.00	H
ATOM	3823	HB2	TYR	A	267	25.072	29.216	19.972	1.00	0.00	H
ATOM	3824	CG	TYR	A	267	25.410	27.131	20.285	1.00	0.00	C
ATOM	3825	CD1	TYR	A	267	26.659	27.304	20.911	1.00	0.00	C
ATOM	3826	HD1	TYR	A	267	26.957	28.284	21.260	1.00	0.00	H
ATOM	3827	CE1	TYR	A	267	27.506	26.201	21.112	1.00	0.00	C
ATOM	3828	HE1	TYR	A	267	28.446	26.322	21.630	1.00	0.00	H
ATOM	3829	CZ	TYR	A	267	27.114	24.922	20.672	1.00	0.00	C
ATOM	3830	OH	TYR	A	267	27.932	23.863	20.893	1.00	0.00	O
ATOM	3831	HH	TYR	A	267	27.575	23.044	20.544	1.00	0.00	H

ATOM	3832	CE2	TYR	A	267	25.869	24.744	20.028	1.00	0.00	C
ATOM	3833	HE2	TYR	A	267	25.567	23.761	19.697	1.00	0.00	H
ATOM	3834	CD2	TYR	A	267	25.020	25.851	19.839	1.00	0.00	C
ATOM	3835	HD2	TYR	A	267	24.059	25.717	19.363	1.00	0.00	H
ATOM	3836	C	TYR	A	267	22.567	27.349	21.495	1.00	0.00	C
ATOM	3837	O	TYR	A	267	22.716	26.529	22.412	1.00	0.00	O
ATOM	3838	N	GLY	A	268	21.601	27.241	20.577	1.00	0.00	N
ATOM	3839	H	GLY	A	268	21.584	27.944	19.841	1.00	0.00	H
ATOM	3840	CA	GLY	A	268	20.681	26.112	20.426	1.00	0.00	C
ATOM	3841	HA1	GLY	A	268	20.813	25.396	21.236	1.00	0.00	H
ATOM	3842	HA2	GLY	A	268	19.660	26.489	20.435	1.00	0.00	H
ATOM	3843	C	GLY	A	268	20.860	25.386	19.095	1.00	0.00	C
ATOM	3844	O	GLY	A	268	21.549	25.870	18.196	1.00	0.00	O
ATOM	3845	N	ARG	A	269	20.216	24.225	18.953	1.00	0.00	N
ATOM	3846	H	ARG	A	269	19.668	23.885	19.737	1.00	0.00	H
ATOM	3847	CA	ARG	A	269	20.222	23.432	17.718	1.00	0.00	C
ATOM	3848	HA	ARG	A	269	20.678	24.023	16.929	1.00	0.00	H
ATOM	3849	CB	ARG	A	269	21.063	22.166	17.892	1.00	0.00	C
ATOM	3850	HB1	ARG	A	269	20.785	21.681	18.826	1.00	0.00	H
ATOM	3851	HB2	ARG	A	269	20.851	21.493	17.060	1.00	0.00	H
ATOM	3852	CG	ARG	A	269	22.567	22.478	17.899	1.00	0.00	C
ATOM	3853	HG1	ARG	A	269	22.798	23.180	17.097	1.00	0.00	H
ATOM	3854	HG2	ARG	A	269	22.843	22.935	18.850	1.00	0.00	H
ATOM	3855	CD	ARG	A	269	23.388	21.205	17.679	1.00	0.00	C
ATOM	3856	HD1	ARG	A	269	23.239	20.535	18.528	1.00	0.00	H
ATOM	3857	HD2	ARG	A	269	23.041	20.697	16.779	1.00	0.00	H
ATOM	3858	NE	ARG	A	269	24.817	21.531	17.573	1.00	0.00	N
ATOM	3859	HE	ARG	A	269	25.284	21.705	18.452	1.00	0.00	H
ATOM	3860	CZ	ARG	A	269	25.566	21.602	16.478	1.00	0.00	C
ATOM	3861	NH1	ARG	A	269	25.117	21.321	15.277	1.00	0.00	N
ATOM	3862	HH11	ARG	A	269	24.206	20.895	15.168	1.00	0.00	H
ATOM	3863	HH12	ARG	A	269	25.742	21.338	14.488	1.00	0.00	H
ATOM	3864	NH2	ARG	A	269	26.822	21.955	16.577	1.00	0.00	N
ATOM	3865	HH21	ARG	A	269	27.204	22.166	17.484	1.00	0.00	H
ATOM	3866	HH22	ARG	A	269	27.446	21.840	15.797	1.00	0.00	H
ATOM	3867	C	ARG	A	269	18.812	23.095	17.237	1.00	0.00	C
ATOM	3868	O	ARG	A	269	17.945	22.710	18.019	1.00	0.00	O
ATOM	3869	N	SER	A	270	18.611	23.268	15.936	1.00	0.00	N
ATOM	3870	H	SER	A	270	19.421	23.493	15.371	1.00	0.00	H
ATOM	3871	CA	SER	A	270	17.327	23.468	15.252	1.00	0.00	C
ATOM	3872	HA	SER	A	270	16.522	23.563	15.982	1.00	0.00	H
ATOM	3873	CB	SER	A	270	17.427	24.782	14.455	1.00	0.00	C
ATOM	3874	HB1	SER	A	270	16.603	24.843	13.746	1.00	0.00	H
ATOM	3875	HB2	SER	A	270	17.361	25.623	15.145	1.00	0.00	H
ATOM	3876	OG	SER	A	270	18.671	24.847	13.766	1.00	0.00	O
ATOM	3877	HG	SER	A	270	18.671	25.585	13.119	1.00	0.00	H
ATOM	3878	C	SER	A	270	16.986	22.317	14.289	1.00	0.00	C
ATOM	3879	O	SER	A	270	17.841	21.842	13.538	1.00	0.00	O
ATOM	3880	N	PHE	A	271	15.732	21.852	14.300	1.00	0.00	N
ATOM	3881	H	PHE	A	271	15.070	22.280	14.940	1.00	0.00	H
ATOM	3882	CA	PHE	A	271	15.275	20.638	13.610	1.00	0.00	C
ATOM	3883	HA	PHE	A	271	15.993	20.338	12.847	1.00	0.00	H
ATOM	3884	CB	PHE	A	271	15.136	19.493	14.631	1.00	0.00	C
ATOM	3885	HB1	PHE	A	271	14.495	19.824	15.450	1.00	0.00	H

ATOM	3886	HB2	PHE	A	271	14.632	18.663	14.135	1.00	0.00	H
ATOM	3887	CG	PHE	A	271	16.413	18.951	15.235	1.00	0.00	C
ATOM	3888	CD1	PHE	A	271	16.832	17.640	14.933	1.00	0.00	C
ATOM	3889	HD1	PHE	A	271	16.254	17.022	14.260	1.00	0.00	H
ATOM	3890	CE1	PHE	A	271	17.994	17.119	15.527	1.00	0.00	C
ATOM	3891	HE1	PHE	A	271	18.290	16.099	15.319	1.00	0.00	H
ATOM	3892	CZ	PHE	A	271	18.760	17.918	16.393	1.00	0.00	C
ATOM	3893	HZ	PHE	A	271	19.646	17.518	16.862	1.00	0.00	H
ATOM	3894	CE2	PHE	A	271	18.352	19.230	16.680	1.00	0.00	C
ATOM	3895	HE2	PHE	A	271	18.928	19.846	17.354	1.00	0.00	H
ATOM	3896	CD2	PHE	A	271	17.160	19.729	16.139	1.00	0.00	C
ATOM	3897	HD2	PHE	A	271	16.817	20.709	16.434	1.00	0.00	H
ATOM	3898	C	PHE	A	271	13.913	20.868	12.936	1.00	0.00	C
ATOM	3899	O	PHE	A	271	13.036	21.514	13.521	1.00	0.00	O
ATOM	3900	N	THR	A	272	13.703	20.274	11.752	1.00	0.00	N
ATOM	3901	H	THR	A	272	14.439	19.685	11.375	1.00	0.00	H
ATOM	3902	CA	THR	A	272	12.401	20.266	11.064	1.00	0.00	C
ATOM	3903	HA	THR	A	272	11.827	21.137	11.381	1.00	0.00	H
ATOM	3904	CB	THR	A	272	12.561	20.359	9.543	1.00	0.00	C
ATOM	3905	HB	THR	A	272	13.143	19.514	9.168	1.00	0.00	H
ATOM	3906	CG2	THR	A	272	11.188	20.375	8.872	1.00	0.00	C
ATOM	3907	HG21	THR	A	272	11.290	20.585	7.812	1.00	0.00	H
ATOM	3908	HG22	THR	A	272	10.704	19.405	8.976	1.00	0.00	H
ATOM	3909	HG23	THR	A	272	10.562	21.137	9.333	1.00	0.00	H
ATOM	3910	OG1	THR	A	272	13.200	21.573	9.205	1.00	0.00	O
ATOM	3911	HG1	THR	A	272	14.163	21.425	9.264	1.00	0.00	H
ATOM	3912	C	THR	A	272	11.614	19.011	11.443	1.00	0.00	C
ATOM	3913	O	THR	A	272	12.089	17.893	11.244	1.00	0.00	O
ATOM	3914	N	LEU	A	273	10.391	19.209	11.939	1.00	0.00	N
ATOM	3915	H	LEU	A	273	10.084	20.168	12.042	1.00	0.00	H
ATOM	3916	CA	LEU	A	273	9.438	18.166	12.326	1.00	0.00	C
ATOM	3917	HA	LEU	A	273	9.970	17.385	12.870	1.00	0.00	H
ATOM	3918	CB	LEU	A	273	8.349	18.781	13.231	1.00	0.00	C
ATOM	3919	HB1	LEU	A	273	7.917	19.638	12.712	1.00	0.00	H
ATOM	3920	HB2	LEU	A	273	7.547	18.055	13.364	1.00	0.00	H
ATOM	3921	CG	LEU	A	273	8.824	19.224	14.627	1.00	0.00	C
ATOM	3922	HG	LEU	A	273	9.678	19.892	14.520	1.00	0.00	H
ATOM	3923	CD1	LEU	A	273	7.710	20.018	15.322	1.00	0.00	C
ATOM	3924	HD11	LEU	A	273	8.047	20.332	16.309	1.00	0.00	H
ATOM	3925	HD12	LEU	A	273	7.468	20.907	14.740	1.00	0.00	H
ATOM	3926	HD13	LEU	A	273	6.815	19.403	15.423	1.00	0.00	H
ATOM	3927	CD2	LEU	A	273	9.225	18.019	15.496	1.00	0.00	C
ATOM	3928	HD21	LEU	A	273	9.494	18.354	16.499	1.00	0.00	H
ATOM	3929	HD22	LEU	A	273	8.401	17.315	15.561	1.00	0.00	H
ATOM	3930	HD23	LEU	A	273	10.073	17.491	15.065	1.00	0.00	H
ATOM	3931	C	LEU	A	273	8.770	17.525	11.106	1.00	0.00	C
ATOM	3932	O	LEU	A	273	8.454	18.204	10.126	1.00	0.00	O
ATOM	3933	N	ALA	A	274	8.454	16.233	11.208	1.00	0.00	N
ATOM	3934	H	ALA	A	274	8.713	15.733	12.050	1.00	0.00	H
ATOM	3935	CA	ALA	A	274	7.618	15.539	10.225	1.00	0.00	C
ATOM	3936	HA	ALA	A	274	8.037	15.696	9.231	1.00	0.00	H
ATOM	3937	CB	ALA	A	274	7.656	14.043	10.554	1.00	0.00	C
ATOM	3938	HB1	ALA	A	274	7.096	13.487	9.800	1.00	0.00	H
ATOM	3939	HB2	ALA	A	274	8.683	13.679	10.572	1.00	0.00	H

ATOM	3940	HB3	ALA	A	274	7.200	13.871	11.528	1.00	0.00	H
ATOM	3941	C	ALA	A	274	6.171	16.070	10.195	1.00	0.00	C
ATOM	3942	O	ALA	A	274	5.535	16.087	9.138	1.00	0.00	O
ATOM	3943	N	SER	A	275	5.655	16.540	11.330	1.00	0.00	N
ATOM	3944	H	SER	A	275	6.203	16.453	12.182	1.00	0.00	H
ATOM	3945	CA	SER	A	275	4.310	17.103	11.466	1.00	0.00	C
ATOM	3946	HA	SER	A	275	4.050	17.638	10.552	1.00	0.00	H
ATOM	3947	CB	SER	A	275	3.310	15.952	11.684	1.00	0.00	C
ATOM	3948	HB1	SER	A	275	3.756	15.010	11.358	1.00	0.00	H
ATOM	3949	HB2	SER	A	275	3.081	15.862	12.748	1.00	0.00	H
ATOM	3950	OG	SER	A	275	2.110	16.137	10.950	1.00	0.00	O
ATOM	3951	HG	SER	A	275	2.325	16.042	9.993	1.00	0.00	H
ATOM	3952	C	SER	A	275	4.264	18.100	12.638	1.00	0.00	C
ATOM	3953	O	SER	A	275	4.833	17.838	13.697	1.00	0.00	O
ATOM	3954	N	SER	A	276	3.572	19.235	12.498	1.00	0.00	N
ATOM	3955	H	SER	A	276	3.026	19.396	11.652	1.00	0.00	H
ATOM	3956	CA	SER	A	276	3.480	20.266	13.551	1.00	0.00	C
ATOM	3957	HA	SER	A	276	4.466	20.388	14.004	1.00	0.00	H
ATOM	3958	CB	SER	A	276	3.090	21.617	12.924	1.00	0.00	C
ATOM	3959	HB1	SER	A	276	3.159	22.400	13.680	1.00	0.00	H
ATOM	3960	HB2	SER	A	276	3.797	21.852	12.126	1.00	0.00	H
ATOM	3961	OG	SER	A	276	1.775	21.600	12.389	1.00	0.00	O
ATOM	3962	HG	SER	A	276	1.150	21.750	13.131	1.00	0.00	H
ATOM	3963	C	SER	A	276	2.525	19.876	14.702	1.00	0.00	C
ATOM	3964	O	SER	A	276	2.249	20.674	15.597	1.00	0.00	O
ATOM	3965	N	SER	A	277	1.983	18.661	14.666	1.00	0.00	N
ATOM	3966	H	SER	A	277	2.197	18.081	13.866	1.00	0.00	H
ATOM	3967	CA	SER	A	277	1.154	18.010	15.687	1.00	0.00	C
ATOM	3968	HA	SER	A	277	0.708	18.763	16.338	1.00	0.00	H
ATOM	3969	CB	SER	A	277	0.021	17.260	14.966	1.00	0.00	C
ATOM	3970	HB1	SER	A	277	-0.463	16.560	15.649	1.00	0.00	H
ATOM	3971	HB2	SER	A	277	-0.722	17.992	14.646	1.00	0.00	H
ATOM	3972	OG	SER	A	277	0.491	16.569	13.813	1.00	0.00	O
ATOM	3973	HG	SER	A	277	-0.111	16.787	13.067	1.00	0.00	H
ATOM	3974	C	SER	A	277	1.952	17.063	16.610	1.00	0.00	C
ATOM	3975	O	SER	A	277	1.371	16.434	17.500	1.00	0.00	O
ATOM	3976	N	ASP	A	278	3.279	16.972	16.452	1.00	0.00	N
ATOM	3977	H	ASP	A	278	3.717	17.471	15.686	1.00	0.00	H
ATOM	3978	CA	ASP	A	278	4.153	16.300	17.417	1.00	0.00	C
ATOM	3979	HA	ASP	A	278	3.612	16.223	18.356	1.00	0.00	H
ATOM	3980	CB	ASP	A	278	4.475	14.872	16.945	1.00	0.00	C
ATOM	3981	HB1	ASP	A	278	3.555	14.395	16.603	1.00	0.00	H
ATOM	3982	HB2	ASP	A	278	5.167	14.910	16.104	1.00	0.00	H
ATOM	3983	CG	ASP	A	278	5.062	14.004	18.062	1.00	0.00	C
ATOM	3984	OD1	ASP	A	278	4.717	14.237	19.241	1.00	0.00	O
ATOM	3985	OD2	ASP	A	278	5.806	13.048	17.737	1.00	0.00	O
ATOM	3986	C	ASP	A	278	5.407	17.130	17.714	1.00	0.00	C
ATOM	3987	O	ASP	A	278	6.339	17.184	16.913	1.00	0.00	O
ATOM	3988	N	THR	A	279	5.388	17.795	18.877	1.00	0.00	N
ATOM	3989	H	THR	A	279	4.538	17.702	19.423	1.00	0.00	H
ATOM	3990	CA	THR	A	279	6.366	18.798	19.345	1.00	0.00	C
ATOM	3991	HA	THR	A	279	7.131	18.920	18.580	1.00	0.00	H
ATOM	3992	CB	THR	A	279	5.674	20.158	19.551	1.00	0.00	C
ATOM	3993	HB	THR	A	279	6.375	20.854	20.012	1.00	0.00	H

ATOM	3994	CG2	THR	A	279	5.218	20.764	18.225	1.00	0.00	C
ATOM	3995	HG21	THR	A	279	4.830	21.766	18.403	1.00	0.00	H
ATOM	3996	HG22	THR	A	279	6.065	20.845	17.547	1.00	0.00	H
ATOM	3997	HG23	THR	A	279	4.443	20.149	17.765	1.00	0.00	H
ATOM	3998	OG1	THR	A	279	4.535	20.025	20.376	1.00	0.00	O
ATOM	3999	HG1	THR	A	279	4.022	20.846	20.286	1.00	0.00	H
ATOM	4000	C	THR	A	279	7.112	18.382	20.618	1.00	0.00	C
ATOM	4001	O	THR	A	279	7.872	19.177	21.171	1.00	0.00	O
ATOM	4002	N	ARG	A	280	6.936	17.143	21.089	1.00	0.00	N
ATOM	4003	H	ARG	A	280	6.316	16.528	20.581	1.00	0.00	H
ATOM	4004	CA	ARG	A	280	7.640	16.609	22.265	1.00	0.00	C
ATOM	4005	HA	ARG	A	280	7.523	17.330	23.075	1.00	0.00	H
ATOM	4006	CB	ARG	A	280	6.965	15.299	22.717	1.00	0.00	C
ATOM	4007	HB1	ARG	A	280	7.380	15.009	23.682	1.00	0.00	H
ATOM	4008	HB2	ARG	A	280	5.898	15.480	22.858	1.00	0.00	H
ATOM	4009	CG	ARG	A	280	7.152	14.132	21.734	1.00	0.00	C
ATOM	4010	HG1	ARG	A	280	6.660	14.365	20.795	1.00	0.00	H
ATOM	4011	HG2	ARG	A	280	8.209	13.971	21.535	1.00	0.00	H
ATOM	4012	CD	ARG	A	280	6.554	12.849	22.313	1.00	0.00	C
ATOM	4013	HD1	ARG	A	280	6.937	12.692	23.322	1.00	0.00	H
ATOM	4014	HD2	ARG	A	280	5.472	12.969	22.377	1.00	0.00	H
ATOM	4015	NE	ARG	A	280	6.850	11.679	21.473	1.00	0.00	N
ATOM	4016	HE	ARG	A	280	6.275	11.560	20.645	1.00	0.00	H
ATOM	4017	CZ	ARG	A	280	7.701	10.697	21.740	1.00	0.00	C
ATOM	4018	NH1	ARG	A	280	8.613	10.789	22.680	1.00	0.00	N
ATOM	4019	HH11	ARG	A	280	8.764	11.676	23.136	1.00	0.00	H
ATOM	4020	HH12	ARG	A	280	9.278	10.038	22.837	1.00	0.00	H
ATOM	4021	NH2	ARG	A	280	7.626	9.585	21.051	1.00	0.00	N
ATOM	4022	HH21	ARG	A	280	6.918	9.503	20.334	1.00	0.00	H
ATOM	4023	HH22	ARG	A	280	8.121	8.759	21.363	1.00	0.00	H
ATOM	4024	C	ARG	A	280	9.152	16.415	22.036	1.00	0.00	C
ATOM	4025	O	ARG	A	280	9.651	16.494	20.913	1.00	0.00	O
ATOM	4026	N	VAL	A	281	9.876	16.069	23.099	1.00	0.00	N
ATOM	4027	H	VAL	A	281	9.403	16.079	24.002	1.00	0.00	H
ATOM	4028	CA	VAL	A	281	11.182	15.393	23.026	1.00	0.00	C
ATOM	4029	HA	VAL	A	281	11.795	15.897	22.278	1.00	0.00	H
ATOM	4030	CB	VAL	A	281	11.922	15.460	24.383	1.00	0.00	C
ATOM	4031	HB	VAL	A	281	11.300	14.985	25.142	1.00	0.00	H
ATOM	4032	CG1	VAL	A	281	13.269	14.728	24.327	1.00	0.00	C
ATOM	4033	HG11	VAL	A	281	13.823	14.901	25.249	1.00	0.00	H
ATOM	4034	HG12	VAL	A	281	13.105	13.657	24.221	1.00	0.00	H
ATOM	4035	HG13	VAL	A	281	13.859	15.093	23.486	1.00	0.00	H
ATOM	4036	CG2	VAL	A	281	12.175	16.913	24.826	1.00	0.00	C
ATOM	4037	HG21	VAL	A	281	12.613	16.914	25.824	1.00	0.00	H
ATOM	4038	HG22	VAL	A	281	12.862	17.403	24.136	1.00	0.00	H
ATOM	4039	HG23	VAL	A	281	11.244	17.476	24.868	1.00	0.00	H
ATOM	4040	C	VAL	A	281	10.949	13.933	22.599	1.00	0.00	C
ATOM	4041	O	VAL	A	281	10.057	13.271	23.133	1.00	0.00	O
ATOM	4042	N	GLY	A	282	11.713	13.448	21.612	1.00	0.00	N
ATOM	4043	H	GLY	A	282	12.436	14.050	21.226	1.00	0.00	H
ATOM	4044	CA	GLY	A	282	11.540	12.134	20.970	1.00	0.00	C
ATOM	4045	HA1	GLY	A	282	12.510	11.803	20.599	1.00	0.00	H
ATOM	4046	HA2	GLY	A	282	11.170	11.411	21.697	1.00	0.00	H
ATOM	4047	C	GLY	A	282	10.577	12.134	19.772	1.00	0.00	C

ATOM	4048	O	GLY	A	282	10.267	11.068	19.237	1.00	0.00	O
ATOM	4049	N	ALA	A	283	10.102	13.307	19.341	1.00	0.00	N
ATOM	4050	H	ALA	A	283	10.492	14.144	19.757	1.00	0.00	H
ATOM	4051	CA	ALA	A	283	9.229	13.482	18.178	1.00	0.00	C
ATOM	4052	HA	ALA	A	283	8.406	12.773	18.267	1.00	0.00	H
ATOM	4053	CB	ALA	A	283	8.637	14.898	18.205	1.00	0.00	C
ATOM	4054	HB1	ALA	A	283	7.952	15.024	17.366	1.00	0.00	H
ATOM	4055	HB2	ALA	A	283	8.085	15.060	19.127	1.00	0.00	H
ATOM	4056	HB3	ALA	A	283	9.431	15.642	18.125	1.00	0.00	H
ATOM	4057	C	ALA	A	283	9.995	13.249	16.855	1.00	0.00	C
ATOM	4058	O	ALA	A	283	11.160	13.648	16.771	1.00	0.00	O
ATOM	4059	N	PRO	A	284	9.370	12.656	15.815	1.00	0.00	N
ATOM	4060	CD	PRO	A	284	8.094	11.957	15.857	1.00	0.00	C
ATOM	4061	HD1	PRO	A	284	7.290	12.681	15.720	1.00	0.00	H
ATOM	4062	HD2	PRO	A	284	7.956	11.400	16.783	1.00	0.00	H
ATOM	4063	CG	PRO	A	284	8.115	10.984	14.682	1.00	0.00	C
ATOM	4064	HG1	PRO	A	284	7.111	10.744	14.332	1.00	0.00	H
ATOM	4065	HG2	PRO	A	284	8.655	10.077	14.960	1.00	0.00	H
ATOM	4066	CB	PRO	A	284	8.911	11.766	13.645	1.00	0.00	C
ATOM	4067	HB1	PRO	A	284	8.252	12.492	13.171	1.00	0.00	H
ATOM	4068	HB2	PRO	A	284	9.355	11.116	12.894	1.00	0.00	H
ATOM	4069	CA	PRO	A	284	9.969	12.483	14.493	1.00	0.00	C
ATOM	4070	HA	PRO	A	284	10.836	11.832	14.602	1.00	0.00	H
ATOM	4071	C	PRO	A	284	10.394	13.799	13.831	1.00	0.00	C
ATOM	4072	O	PRO	A	284	9.594	14.729	13.686	1.00	0.00	O
ATOM	4073	N	ALA	A	285	11.648	13.831	13.368	1.00	0.00	N
ATOM	4074	H	ALA	A	285	12.244	13.033	13.557	1.00	0.00	H
ATOM	4075	CA	ALA	A	285	12.221	14.907	12.559	1.00	0.00	C
ATOM	4076	HA	ALA	A	285	11.515	15.734	12.496	1.00	0.00	H
ATOM	4077	CB	ALA	A	285	13.487	15.419	13.262	1.00	0.00	C
ATOM	4078	HB1	ALA	A	285	13.905	16.259	12.705	1.00	0.00	H
ATOM	4079	HB2	ALA	A	285	13.234	15.763	14.267	1.00	0.00	H
ATOM	4080	HB3	ALA	A	285	14.233	14.626	13.329	1.00	0.00	H
ATOM	4081	C	ALA	A	285	12.523	14.420	11.129	1.00	0.00	C
ATOM	4082	O	ALA	A	285	12.938	13.269	10.931	1.00	0.00	O
ATOM	4083	N	THR	A	286	12.343	15.292	10.129	1.00	0.00	N
ATOM	4084	H	THR	A	286	12.003	16.222	10.362	1.00	0.00	H
ATOM	4085	CA	THR	A	286	12.702	15.039	8.717	1.00	0.00	C
ATOM	4086	HA	THR	A	286	12.788	13.965	8.561	1.00	0.00	H
ATOM	4087	CB	THR	A	286	11.619	15.542	7.756	1.00	0.00	C
ATOM	4088	HB	THR	A	286	11.987	15.499	6.728	1.00	0.00	H
ATOM	4089	CG2	THR	A	286	10.384	14.652	7.850	1.00	0.00	C
ATOM	4090	HG21	THR	A	286	9.612	15.025	7.179	1.00	0.00	H
ATOM	4091	HG22	THR	A	286	10.636	13.634	7.552	1.00	0.00	H
ATOM	4092	HG23	THR	A	286	10.007	14.641	8.869	1.00	0.00	H
ATOM	4093	OG1	THR	A	286	11.253	16.866	8.063	1.00	0.00	O
ATOM	4094	HG1	THR	A	286	11.903	17.457	7.633	1.00	0.00	H
ATOM	4095	C	THR	A	286	14.061	15.611	8.334	1.00	0.00	C
ATOM	4096	O	THR	A	286	14.545	15.290	7.248	1.00	0.00	O
ATOM	4097	N	GLY	A	287	14.707	16.382	9.212	1.00	0.00	N
ATOM	4098	H	GLY	A	287	14.236	16.645	10.066	1.00	0.00	H
ATOM	4099	CA	GLY	A	287	16.091	16.830	9.044	1.00	0.00	C
ATOM	4100	HA1	GLY	A	287	16.765	16.040	9.377	1.00	0.00	H
ATOM	4101	HA2	GLY	A	287	16.291	16.998	7.989	1.00	0.00	H

ATOM	4102	C	GLY	A	287	16.424	18.088	9.837	1.00	0.00	C
ATOM	4103	O	GLY	A	287	15.789	18.390	10.849	1.00	0.00	O
ATOM	4104	N	SER	A	288	17.430	18.832	9.383	1.00	0.00	N
ATOM	4105	H	SER	A	288	17.845	18.607	8.483	1.00	0.00	H
ATOM	4106	CA	SER	A	288	17.855	20.078	10.028	1.00	0.00	C
ATOM	4107	HA	SER	A	288	18.005	19.875	11.088	1.00	0.00	H
ATOM	4108	CB	SER	A	288	19.207	20.547	9.458	1.00	0.00	C
ATOM	4109	HB1	SER	A	288	19.470	21.515	9.887	1.00	0.00	H
ATOM	4110	HB2	SER	A	288	19.972	19.830	9.751	1.00	0.00	H
ATOM	4111	OG	SER	A	288	19.211	20.636	8.041	1.00	0.00	O
ATOM	4112	HG	SER	A	288	19.117	21.578	7.787	1.00	0.00	H
ATOM	4113	C	SER	A	288	16.787	21.182	9.938	1.00	0.00	C
ATOM	4114	O	SER	A	288	15.895	21.156	9.080	1.00	0.00	O
ATOM	4115	N	GLY	A	289	16.864	22.162	10.841	1.00	0.00	N
ATOM	4116	H	GLY	A	289	17.563	22.110	11.575	1.00	0.00	H
ATOM	4117	CA	GLY	A	289	15.981	23.327	10.851	1.00	0.00	C
ATOM	4118	HA1	GLY	A	289	14.957	23.012	10.651	1.00	0.00	H
ATOM	4119	HA2	GLY	A	289	16.013	23.810	11.826	1.00	0.00	H
ATOM	4120	C	GLY	A	289	16.363	24.373	9.803	1.00	0.00	C
ATOM	4121	O	GLY	A	289	17.439	24.315	9.194	1.00	0.00	O
ATOM	4122	N	THR	A	290	15.471	25.344	9.607	1.00	0.00	N
ATOM	4123	H	THR	A	290	14.594	25.287	10.116	1.00	0.00	H
ATOM	4124	CA	THR	A	290	15.679	26.514	8.746	1.00	0.00	C
ATOM	4125	HA	THR	A	290	15.871	26.154	7.740	1.00	0.00	H
ATOM	4126	CB	THR	A	290	14.395	27.348	8.707	1.00	0.00	C
ATOM	4127	HB	THR	A	290	14.146	27.688	9.714	1.00	0.00	H
ATOM	4128	CG2	THR	A	290	14.488	28.554	7.777	1.00	0.00	C
ATOM	4129	HG21	THR	A	290	13.504	29.012	7.686	1.00	0.00	H
ATOM	4130	HG22	THR	A	290	15.181	29.287	8.184	1.00	0.00	H
ATOM	4131	HG23	THR	A	290	14.826	28.250	6.788	1.00	0.00	H
ATOM	4132	OG1	THR	A	290	13.358	26.528	8.226	1.00	0.00	O
ATOM	4133	HG1	THR	A	290	13.587	26.261	7.318	1.00	0.00	H
ATOM	4134	C	THR	A	290	16.876	27.336	9.255	1.00	0.00	C
ATOM	4135	O	THR	A	290	16.996	27.517	10.470	1.00	0.00	O
ATOM	4136	N	PRO	A	291	17.775	27.829	8.377	1.00	0.00	N
ATOM	4137	CD	PRO	A	291	17.859	27.539	6.953	1.00	0.00	C
ATOM	4138	HD1	PRO	A	291	17.179	28.193	6.407	1.00	0.00	H
ATOM	4139	HD2	PRO	A	291	17.643	26.494	6.732	1.00	0.00	H
ATOM	4140	CG	PRO	A	291	19.299	27.846	6.559	1.00	0.00	C
ATOM	4141	HG1	PRO	A	291	19.367	28.150	5.515	1.00	0.00	H
ATOM	4142	HG2	PRO	A	291	19.926	26.975	6.754	1.00	0.00	H
ATOM	4143	CB	PRO	A	291	19.693	28.975	7.506	1.00	0.00	C
ATOM	4144	HB1	PRO	A	291	19.370	29.928	7.085	1.00	0.00	H
ATOM	4145	HB2	PRO	A	291	20.767	28.987	7.691	1.00	0.00	H
ATOM	4146	CA	PRO	A	291	18.908	28.658	8.783	1.00	0.00	C
ATOM	4147	HA	PRO	A	291	19.544	28.068	9.445	1.00	0.00	H
ATOM	4148	C	PRO	A	291	18.481	29.942	9.501	1.00	0.00	C
ATOM	4149	O	PRO	A	291	17.429	30.519	9.210	1.00	0.00	O
ATOM	4150	N	GLY	A	292	19.340	30.408	10.408	1.00	0.00	N
ATOM	4151	H	GLY	A	292	20.191	29.882	10.583	1.00	0.00	H
ATOM	4152	CA	GLY	A	292	19.185	31.656	11.151	1.00	0.00	C
ATOM	4153	HA1	GLY	A	292	18.151	31.742	11.484	1.00	0.00	H
ATOM	4154	HA2	GLY	A	292	19.843	31.648	12.022	1.00	0.00	H
ATOM	4155	C	GLY	A	292	19.550	32.888	10.314	1.00	0.00	C

ATOM	4156	O	GLY	A	292	20.260	32.764	9.304	1.00	0.00	O
ATOM	4157	N	PRO	A	293	19.060	34.080	10.703	1.00	0.00	N
ATOM	4158	CD	PRO	A	293	18.215	34.314	11.867	1.00	0.00	C
ATOM	4159	HD1	PRO	A	293	18.614	33.835	12.762	1.00	0.00	H
ATOM	4160	HD2	PRO	A	293	17.207	33.949	11.665	1.00	0.00	H
ATOM	4161	CG	PRO	A	293	18.183	35.826	12.052	1.00	0.00	C
ATOM	4162	HG1	PRO	A	293	19.037	36.136	12.651	1.00	0.00	H
ATOM	4163	HG2	PRO	A	293	17.250	36.158	12.506	1.00	0.00	H
ATOM	4164	CB	PRO	A	293	18.334	36.341	10.622	1.00	0.00	C
ATOM	4165	HB1	PRO	A	293	18.762	37.342	10.607	1.00	0.00	H
ATOM	4166	HB2	PRO	A	293	17.358	36.340	10.135	1.00	0.00	H
ATOM	4167	CA	PRO	A	293	19.242	35.311	9.938	1.00	0.00	C
ATOM	4168	HA	PRO	A	293	18.890	35.159	8.917	1.00	0.00	H
ATOM	4169	C	PRO	A	293	20.702	35.785	9.880	1.00	0.00	C
ATOM	4170	O	PRO	A	293	21.079	36.441	8.906	1.00	0.00	O
ATOM	4171	N	PHE	A	294	21.527	35.455	10.882	1.00	0.00	N
ATOM	4172	H	PHE	A	294	21.168	34.889	11.652	1.00	0.00	H
ATOM	4173	CA	PHE	A	294	22.904	35.934	11.012	1.00	0.00	C
ATOM	4174	HA	PHE	A	294	23.137	36.638	10.218	1.00	0.00	H
ATOM	4175	CB	PHE	A	294	23.030	36.675	12.344	1.00	0.00	C
ATOM	4176	HB1	PHE	A	294	22.758	35.984	13.141	1.00	0.00	H
ATOM	4177	HB2	PHE	A	294	24.075	36.948	12.493	1.00	0.00	H
ATOM	4178	CG	PHE	A	294	22.192	37.937	12.452	1.00	0.00	C
ATOM	4179	CD1	PHE	A	294	22.557	39.090	11.730	1.00	0.00	C
ATOM	4180	HD1	PHE	A	294	23.417	39.074	11.076	1.00	0.00	H
ATOM	4181	CE1	PHE	A	294	21.831	40.283	11.888	1.00	0.00	C
ATOM	4182	HE1	PHE	A	294	22.132	41.170	11.348	1.00	0.00	H
ATOM	4183	CZ	PHE	A	294	20.724	40.325	12.752	1.00	0.00	C
ATOM	4184	HZ	PHE	A	294	20.171	41.246	12.881	1.00	0.00	H
ATOM	4185	CE2	PHE	A	294	20.338	39.169	13.451	1.00	0.00	C
ATOM	4186	HE2	PHE	A	294	19.487	39.200	14.116	1.00	0.00	H
ATOM	4187	CD2	PHE	A	294	21.075	37.980	13.308	1.00	0.00	C
ATOM	4188	HD2	PHE	A	294	20.787	37.103	13.870	1.00	0.00	H
ATOM	4189	C	PHE	A	294	23.950	34.814	10.944	1.00	0.00	C
ATOM	4190	O	PHE	A	294	24.995	35.030	10.320	1.00	0.00	O
ATOM	4191	N	THR	A	295	23.688	33.649	11.556	1.00	0.00	N
ATOM	4192	H	THR	A	295	22.815	33.561	12.080	1.00	0.00	H
ATOM	4193	CA	THR	A	295	24.620	32.502	11.570	1.00	0.00	C
ATOM	4194	HA	THR	A	295	25.635	32.870	11.706	1.00	0.00	H
ATOM	4195	CB	THR	A	295	24.308	31.553	12.732	1.00	0.00	C
ATOM	4196	HB	THR	A	295	24.938	30.670	12.623	1.00	0.00	H
ATOM	4197	CG2	THR	A	295	24.623	32.181	14.089	1.00	0.00	C
ATOM	4198	HG21	THR	A	295	24.376	31.471	14.877	1.00	0.00	H
ATOM	4199	HG22	THR	A	295	25.685	32.418	14.148	1.00	0.00	H
ATOM	4200	HG23	THR	A	295	24.046	33.094	14.235	1.00	0.00	H
ATOM	4201	OG1	THR	A	295	22.954	31.158	12.683	1.00	0.00	O
ATOM	4202	HG1	THR	A	295	22.461	31.607	13.417	1.00	0.00	H
ATOM	4203	C	THR	A	295	24.623	31.726	10.257	1.00	0.00	C
ATOM	4204	O	THR	A	295	25.676	31.261	9.823	1.00	0.00	O
ATOM	4205	N	LYS	A	296	23.463	31.610	9.604	1.00	0.00	N
ATOM	4206	H	LYS	A	296	22.644	31.961	10.082	1.00	0.00	H
ATOM	4207	CA	LYS	A	296	23.277	31.076	8.245	1.00	0.00	C
ATOM	4208	HA	LYS	A	296	22.200	31.024	8.082	1.00	0.00	H
ATOM	4209	CB	LYS	A	296	23.855	32.066	7.206	1.00	0.00	C

ATOM	4210	HB1	LYS	A	296	24.914	32.242	7.405	1.00	0.00	H
ATOM	4211	HB2	LYS	A	296	23.770	31.619	6.215	1.00	0.00	H
ATOM	4212	CG	LYS	A	296	23.105	33.406	7.171	1.00	0.00	C
ATOM	4213	HG1	LYS	A	296	22.036	33.208	7.090	1.00	0.00	H
ATOM	4214	HG2	LYS	A	296	23.290	33.966	8.088	1.00	0.00	H
ATOM	4215	CD	LYS	A	296	23.552	34.236	5.959	1.00	0.00	C
ATOM	4216	HD1	LYS	A	296	24.584	34.565	6.100	1.00	0.00	H
ATOM	4217	HD2	LYS	A	296	23.498	33.619	5.060	1.00	0.00	H
ATOM	4218	CE	LYS	A	296	22.636	35.449	5.777	1.00	0.00	C
ATOM	4219	HE1	LYS	A	296	21.598	35.107	5.739	1.00	0.00	H
ATOM	4220	HE2	LYS	A	296	22.744	36.111	6.638	1.00	0.00	H
ATOM	4221	NZ	LYS	A	296	22.954	36.190	4.535	1.00	0.00	N
ATOM	4222	HZ1	LYS	A	296	23.909	36.535	4.576	1.00	0.00	H
ATOM	4223	HZ2	LYS	A	296	22.872	35.601	3.710	1.00	0.00	H
ATOM	4224	HZ3	LYS	A	296	22.351	37.000	4.450	1.00	0.00	H
ATOM	4225	C	LYS	A	296	23.774	29.624	8.018	1.00	0.00	C
ATOM	4226	O	LYS	A	296	23.960	29.227	6.861	1.00	0.00	O
ATOM	4227	N	GLU	A	297	23.971	28.824	9.069	1.00	0.00	N
ATOM	4228	H	GLU	A	297	23.733	29.201	9.981	1.00	0.00	H
ATOM	4229	CA	GLU	A	297	24.296	27.393	9.008	1.00	0.00	C
ATOM	4230	HA	GLU	A	297	24.426	27.100	7.967	1.00	0.00	H
ATOM	4231	CB	GLU	A	297	25.643	27.129	9.709	1.00	0.00	C
ATOM	4232	HB1	GLU	A	297	26.412	27.597	9.099	1.00	0.00	H
ATOM	4233	HB2	GLU	A	297	25.658	27.597	10.693	1.00	0.00	H
ATOM	4234	CG	GLU	A	297	25.973	25.636	9.860	1.00	0.00	C
ATOM	4235	HG1	GLU	A	297	25.452	25.237	10.731	1.00	0.00	H
ATOM	4236	HG2	GLU	A	297	25.607	25.107	8.978	1.00	0.00	H
ATOM	4237	CD	GLU	A	297	27.472	25.346	9.997	1.00	0.00	C
ATOM	4238	OE1	GLU	A	297	27.926	24.342	9.396	1.00	0.00	O
ATOM	4239	OE2	GLU	A	297	28.223	26.079	10.678	1.00	0.00	O
ATOM	4240	C	GLU	A	297	23.142	26.550	9.581	1.00	0.00	C
ATOM	4241	O	GLU	A	297	22.737	26.704	10.735	1.00	0.00	O
ATOM	4242	N	GLY	A	298	22.598	25.644	8.764	1.00	0.00	N
ATOM	4243	H	GLY	A	298	22.930	25.596	7.807	1.00	0.00	H
ATOM	4244	CA	GLY	A	298	21.450	24.815	9.130	1.00	0.00	C
ATOM	4245	HA1	GLY	A	298	20.635	25.463	9.457	1.00	0.00	H
ATOM	4246	HA2	GLY	A	298	21.121	24.239	8.267	1.00	0.00	H
ATOM	4247	C	GLY	A	298	21.779	23.842	10.260	1.00	0.00	C
ATOM	4248	O	GLY	A	298	22.746	23.075	10.178	1.00	0.00	O
ATOM	4249	N	GLY	A	299	20.946	23.848	11.304	1.00	0.00	N
ATOM	4250	H	GLY	A	299	20.210	24.547	11.313	1.00	0.00	H
ATOM	4251	CA	GLY	A	299	21.047	22.957	12.467	1.00	0.00	C
ATOM	4252	HA1	GLY	A	299	20.048	22.596	12.704	1.00	0.00	H
ATOM	4253	HA2	GLY	A	299	21.690	22.105	12.247	1.00	0.00	H
ATOM	4254	C	GLY	A	299	21.569	23.621	13.743	1.00	0.00	C
ATOM	4255	O	GLY	A	299	21.491	22.995	14.797	1.00	0.00	O
ATOM	4256	N	MET	A	300	22.042	24.872	13.688	1.00	0.00	N
ATOM	4257	H	MET	A	300	22.057	25.338	12.787	1.00	0.00	H
ATOM	4258	CA	MET	A	300	22.357	25.702	14.864	1.00	0.00	C
ATOM	4259	HA	MET	A	300	21.908	25.253	15.747	1.00	0.00	H
ATOM	4260	CB	MET	A	300	23.883	25.744	15.098	1.00	0.00	C
ATOM	4261	HB1	MET	A	300	24.209	24.728	15.322	1.00	0.00	H
ATOM	4262	HB2	MET	A	300	24.395	26.059	14.189	1.00	0.00	H
ATOM	4263	CG	MET	A	300	24.312	26.650	16.270	1.00	0.00	C

```
ATOM   4264  HG1 MET A 300      23.576  26.572  17.067  1.00  0.00           H
ATOM   4265  HG2 MET A 300      25.237  26.269  16.687  1.00  0.00           H
ATOM   4266  SD  MET A 300      24.568  28.412  15.907  1.00  0.00           S
ATOM   4267  CE  MET A 300      26.259  28.382  15.267  1.00  0.00           C
ATOM   4268  HE1 MET A 300      26.526  29.370  14.891  1.00  0.00           H
ATOM   4269  HE2 MET A 300      26.945  28.129  16.075  1.00  0.00           H
ATOM   4270  HE3 MET A 300      26.357  27.653  14.461  1.00  0.00           H
ATOM   4271  C   MET A 300      21.741  27.097  14.717  1.00  0.00           C
ATOM   4272  O   MET A 300      21.633  27.610  13.601  1.00  0.00           O
ATOM   4273  N   LEU A 301      21.356  27.697  15.847  1.00  0.00           N
ATOM   4274  H   LEU A 301      21.495  27.195  16.718  1.00  0.00           H
ATOM   4275  CA  LEU A 301      20.937  29.093  15.988  1.00  0.00           C
ATOM   4276  HA  LEU A 301      21.256  29.678  15.122  1.00  0.00           H
ATOM   4277  CB  LEU A 301      19.404  29.154  16.130  1.00  0.00           C
ATOM   4278  HB1 LEU A 301      19.124  28.579  17.015  1.00  0.00           H
ATOM   4279  HB2 LEU A 301      19.121  30.191  16.306  1.00  0.00           H
ATOM   4280  CG  LEU A 301      18.600  28.629  14.923  1.00  0.00           C
ATOM   4281  HG  LEU A 301      18.950  27.634  14.652  1.00  0.00           H
ATOM   4282  CD1 LEU A 301      17.120  28.507  15.305  1.00  0.00           C
ATOM   4283 HD11 LEU A 301      16.548  28.124  14.460  1.00  0.00           H
ATOM   4284 HD12 LEU A 301      17.011  27.819  16.144  1.00  0.00           H
ATOM   4285 HD13 LEU A 301      16.732  29.483  15.592  1.00  0.00           H
ATOM   4286  CD2 LEU A 301      18.737  29.551  13.706  1.00  0.00           C
ATOM   4287 HD21 LEU A 301      18.134  29.168  12.882  1.00  0.00           H
ATOM   4288 HD22 LEU A 301      18.399  30.557  13.957  1.00  0.00           H
ATOM   4289 HD23 LEU A 301      19.777  29.596  13.384  1.00  0.00           H
ATOM   4290  C   LEU A 301      21.609  29.679  17.241  1.00  0.00           C
ATOM   4291  O   LEU A 301      21.628  29.025  18.287  1.00  0.00           O
ATOM   4292  N   ALA A 302      22.155  30.893  17.153  1.00  0.00           N
ATOM   4293  H   ALA A 302      22.057  31.397  16.271  1.00  0.00           H
ATOM   4294  CA  ALA A 302      22.786  31.577  18.291  1.00  0.00           C
ATOM   4295  HA  ALA A 302      23.495  30.896  18.765  1.00  0.00           H
ATOM   4296  CB  ALA A 302      23.556  32.791  17.761  1.00  0.00           C
ATOM   4297  HB1 ALA A 302      24.061  33.294  18.585  1.00  0.00           H
ATOM   4298  HB2 ALA A 302      24.304  32.473  17.037  1.00  0.00           H
ATOM   4299  HB3 ALA A 302      22.871  33.491  17.284  1.00  0.00           H
ATOM   4300  C   ALA A 302      21.754  31.984  19.360  1.00  0.00           C
ATOM   4301  O   ALA A 302      20.564  32.115  19.057  1.00  0.00           O
ATOM   4302  N   TYR A 303      22.186  32.268  20.597  1.00  0.00           N
ATOM   4303  H   TYR A 303      23.179  32.204  20.814  1.00  0.00           H
ATOM   4304  CA  TYR A 303      21.276  32.733  21.657  1.00  0.00           C
ATOM   4305  HA  TYR A 303      20.577  31.927  21.883  1.00  0.00           H
ATOM   4306  CB  TYR A 303      22.071  33.052  22.933  1.00  0.00           C
ATOM   4307  HB1 TYR A 303      22.652  32.173  23.212  1.00  0.00           H
ATOM   4308  HB2 TYR A 303      22.775  33.859  22.727  1.00  0.00           H
ATOM   4309  CG  TYR A 303      21.203  33.433  24.119  1.00  0.00           C
ATOM   4310  CD1 TYR A 303      20.482  32.437  24.805  1.00  0.00           C
ATOM   4311  HD1 TYR A 303      20.571  31.405  24.495  1.00  0.00           H
ATOM   4312  CE1 TYR A 303      19.688  32.771  25.919  1.00  0.00           C
ATOM   4313  HE1 TYR A 303      19.159  31.999  26.459  1.00  0.00           H
ATOM   4314  CZ  TYR A 303      19.578  34.117  26.329  1.00  0.00           C
ATOM   4315  OH  TYR A 303      18.768  34.440  27.374  1.00  0.00           O
ATOM   4316  HH  TYR A 303      18.724  35.399  27.523  1.00  0.00           H
ATOM   4317  CE2 TYR A 303      20.298  35.118  25.641  1.00  0.00           C
```

ATOM	4318	HE2	TYR	A	303	20.224	36.149	25.955	1.00	0.00	H
ATOM	4319	CD2	TYR	A	303	21.113	34.773	24.545	1.00	0.00	C
ATOM	4320	HD2	TYR	A	303	21.673	35.542	24.029	1.00	0.00	H
ATOM	4321	C	TYR	A	303	20.449	33.944	21.188	1.00	0.00	C
ATOM	4322	O	TYR	A	303	19.216	33.910	21.216	1.00	0.00	O
ATOM	4323	N	TYR	A	304	21.109	34.951	20.604	1.00	0.00	N
ATOM	4324	H	TYR	A	304	22.121	34.892	20.580	1.00	0.00	H
ATOM	4325	CA	TYR	A	304	20.467	36.137	20.022	1.00	0.00	C
ATOM	4326	HA	TYR	A	304	19.820	36.561	20.788	1.00	0.00	H
ATOM	4327	CB	TYR	A	304	21.541	37.193	19.714	1.00	0.00	C
ATOM	4328	HB1	TYR	A	304	21.051	38.123	19.428	1.00	0.00	H
ATOM	4329	HB2	TYR	A	304	22.101	37.399	20.627	1.00	0.00	H
ATOM	4330	CG	TYR	A	304	22.509	36.817	18.611	1.00	0.00	C
ATOM	4331	CD1	TYR	A	304	22.101	36.872	17.264	1.00	0.00	C
ATOM	4332	HD1	TYR	A	304	21.106	37.200	17.007	1.00	0.00	H
ATOM	4333	CE1	TYR	A	304	22.984	36.492	16.242	1.00	0.00	C
ATOM	4334	HE1	TYR	A	304	22.664	36.521	15.217	1.00	0.00	H
ATOM	4335	CZ	TYR	A	304	24.294	36.076	16.552	1.00	0.00	C
ATOM	4336	OH	TYR	A	304	25.137	35.707	15.549	1.00	0.00	O
ATOM	4337	HH	TYR	A	304	26.048	35.614	15.854	1.00	0.00	H
ATOM	4338	CE2	TYR	A	304	24.713	36.060	17.899	1.00	0.00	C
ATOM	4339	HE2	TYR	A	304	25.710	35.754	18.156	1.00	0.00	H
ATOM	4340	CD2	TYR	A	304	23.819	36.415	18.924	1.00	0.00	C
ATOM	4341	HD2	TYR	A	304	24.146	36.374	19.955	1.00	0.00	H
ATOM	4342	C	TYR	A	304	19.560	35.863	18.797	1.00	0.00	C
ATOM	4343	O	TYR	A	304	18.872	36.782	18.344	1.00	0.00	O
ATOM	4344	N	GLU	A	305	19.531	34.642	18.251	1.00	0.00	N
ATOM	4345	H	GLU	A	305	20.154	33.931	18.624	1.00	0.00	H
ATOM	4346	CA	GLU	A	305	18.594	34.229	17.195	1.00	0.00	C
ATOM	4347	HA	GLU	A	305	18.240	35.114	16.666	1.00	0.00	H
ATOM	4348	CB	GLU	A	305	19.308	33.341	16.151	1.00	0.00	C
ATOM	4349	HB1	GLU	A	305	19.775	32.495	16.651	1.00	0.00	H
ATOM	4350	HB2	GLU	A	305	18.572	32.950	15.450	1.00	0.00	H
ATOM	4351	CG	GLU	A	305	20.364	34.119	15.348	1.00	0.00	C
ATOM	4352	HG1	GLU	A	305	19.883	34.966	14.857	1.00	0.00	H
ATOM	4353	HG2	GLU	A	305	21.116	34.503	16.033	1.00	0.00	H
ATOM	4354	CD	GLU	A	305	21.077	33.264	14.296	1.00	0.00	C
ATOM	4355	OE1	GLU	A	305	21.548	32.159	14.643	1.00	0.00	O
ATOM	4356	OE2	GLU	A	305	21.215	33.715	13.134	1.00	0.00	O
ATOM	4357	C	GLU	A	305	17.333	33.533	17.757	1.00	0.00	C
ATOM	4358	O	GLU	A	305	16.343	33.446	17.027	1.00	0.00	O
ATOM	4359	N	VAL	A	306	17.314	33.081	19.024	1.00	0.00	N
ATOM	4360	H	VAL	A	306	18.177	33.148	19.560	1.00	0.00	H
ATOM	4361	CA	VAL	A	306	16.168	32.344	19.624	1.00	0.00	C
ATOM	4362	HA	VAL	A	306	15.325	32.432	18.940	1.00	0.00	H
ATOM	4363	CB	VAL	A	306	16.464	30.830	19.771	1.00	0.00	C
ATOM	4364	HB	VAL	A	306	15.584	30.348	20.199	1.00	0.00	H
ATOM	4365	CG1	VAL	A	306	16.692	30.183	18.399	1.00	0.00	C
ATOM	4366	HG11	VAL	A	306	16.823	29.108	18.513	1.00	0.00	H
ATOM	4367	HG12	VAL	A	306	15.829	30.365	17.758	1.00	0.00	H
ATOM	4368	HG13	VAL	A	306	17.582	30.600	17.932	1.00	0.00	H
ATOM	4369	CG2	VAL	A	306	17.651	30.531	20.706	1.00	0.00	C
ATOM	4370	HG21	VAL	A	306	17.782	29.453	20.801	1.00	0.00	H
ATOM	4371	HG22	VAL	A	306	18.567	30.965	20.310	1.00	0.00	H

ATOM	4372	HG23	VAL	A	306	17.462	30.943	21.696	1.00	0.00	H
ATOM	4373	C	VAL	A	306	15.652	32.882	20.974	1.00	0.00	C
ATOM	4374	O	VAL	A	306	14.619	32.417	21.458	1.00	0.00	O
ATOM	4375	N	CYS	A	307	16.325	33.850	21.603	1.00	0.00	N
ATOM	4376	H	CYS	A	307	17.184	34.198	21.193	1.00	0.00	H
ATOM	4377	CA	CYS	A	307	15.966	34.354	22.939	1.00	0.00	C
ATOM	4378	HA	CYS	A	307	15.823	33.510	23.609	1.00	0.00	H
ATOM	4379	CB	CYS	A	307	17.138	35.181	23.465	1.00	0.00	C
ATOM	4380	HB1	CYS	A	307	16.897	35.539	24.465	1.00	0.00	H
ATOM	4381	HB2	CYS	A	307	18.016	34.539	23.546	1.00	0.00	H
ATOM	4382	SG	CYS	A	307	17.562	36.602	22.433	1.00	0.00	S
ATOM	4383	C	CYS	A	307	14.658	35.155	22.993	1.00	0.00	C
ATOM	4384	O	CYS	A	307	13.970	35.141	24.008	1.00	0.00	O
ATOM	4385	N	SER	A	308	14.277	35.812	21.902	1.00	0.00	N
ATOM	4386	H	SER	A	308	14.926	35.809	21.117	1.00	0.00	H
ATOM	4387	CA	SER	A	308	13.016	36.544	21.722	1.00	0.00	C
ATOM	4388	HA	SER	A	308	12.339	36.345	22.555	1.00	0.00	H
ATOM	4389	CB	SER	A	308	13.311	38.054	21.687	1.00	0.00	C
ATOM	4390	HB1	SER	A	308	14.215	38.244	21.107	1.00	0.00	H
ATOM	4391	HB2	SER	A	308	12.482	38.581	21.213	1.00	0.00	H
ATOM	4392	OG	SER	A	308	13.448	38.587	22.992	1.00	0.00	O
ATOM	4393	HG	SER	A	308	14.090	38.075	23.519	1.00	0.00	H
ATOM	4394	C	SER	A	308	12.293	36.063	20.447	1.00	0.00	C
ATOM	4395	O	SER	A	308	11.853	36.863	19.616	1.00	0.00	O
ATOM	4396	N	TRP	A	309	12.228	34.741	20.240	1.00	0.00	N
ATOM	4397	H	TRP	A	309	12.618	34.127	20.943	1.00	0.00	H
ATOM	4398	CA	TRP	A	309	11.692	34.122	19.023	1.00	0.00	C
ATOM	4399	HA	TRP	A	309	12.181	34.602	18.177	1.00	0.00	H
ATOM	4400	CB	TRP	A	309	12.088	32.636	19.010	1.00	0.00	C
ATOM	4401	HB1	TRP	A	309	13.115	32.557	19.357	1.00	0.00	H
ATOM	4402	HB2	TRP	A	309	11.468	32.096	19.727	1.00	0.00	H
ATOM	4403	CG	TRP	A	309	12.048	31.917	17.693	1.00	0.00	C
ATOM	4404	CD1	TRP	A	309	12.273	32.465	16.477	1.00	0.00	C
ATOM	4405	HD1	TRP	A	309	12.495	33.509	16.288	1.00	0.00	H
ATOM	4406	NE1	TRP	A	309	12.221	31.487	15.508	1.00	0.00	N
ATOM	4407	HE1	TRP	A	309	12.389	31.675	14.526	1.00	0.00	H
ATOM	4408	CE2	TRP	A	309	11.972	30.244	16.043	1.00	0.00	C
ATOM	4409	CZ2	TRP	A	309	11.855	28.962	15.484	1.00	0.00	C
ATOM	4410	HZ2	TRP	A	309	11.945	28.820	14.417	1.00	0.00	H
ATOM	4411	CH2	TRP	A	309	11.650	27.863	16.336	1.00	0.00	C
ATOM	4412	HH2	TRP	A	309	11.576	26.863	15.932	1.00	0.00	H
ATOM	4413	CZ3	TRP	A	309	11.573	28.063	17.725	1.00	0.00	C
ATOM	4414	HZ3	TRP	A	309	11.451	27.215	18.381	1.00	0.00	H
ATOM	4415	CE3	TRP	A	309	11.689	29.356	18.269	1.00	0.00	C
ATOM	4416	HE3	TRP	A	309	11.656	29.493	19.336	1.00	0.00	H
ATOM	4417	CD2	TRP	A	309	11.877	30.485	17.445	1.00	0.00	C
ATOM	4418	C	TRP	A	309	10.172	34.351	18.894	1.00	0.00	C
ATOM	4419	O	TRP	A	309	9.371	33.861	19.696	1.00	0.00	O
ATOM	4420	N	LYS	A	310	9.765	35.136	17.893	1.00	0.00	N
ATOM	4421	H	LYS	A	310	10.451	35.459	17.223	1.00	0.00	H
ATOM	4422	CA	LYS	A	310	8.408	35.692	17.790	1.00	0.00	C
ATOM	4423	HA	LYS	A	310	8.116	36.081	18.767	1.00	0.00	H
ATOM	4424	CB	LYS	A	310	8.437	36.875	16.802	1.00	0.00	C
ATOM	4425	HB1	LYS	A	310	8.968	36.578	15.896	1.00	0.00	H

```
ATOM   4426  HB2 LYS A 310       7.421  37.154  16.521  1.00  0.00           H
ATOM   4427  CG  LYS A 310       9.121  38.089  17.458  1.00  0.00           C
ATOM   4428  HG1 LYS A 310       8.389  38.601  18.080  1.00  0.00           H
ATOM   4429  HG2 LYS A 310       9.917  37.737  18.108  1.00  0.00           H
ATOM   4430  CD  LYS A 310       9.726  39.082  16.452  1.00  0.00           C
ATOM   4431  HD1 LYS A 310      10.436  38.556  15.811  1.00  0.00           H
ATOM   4432  HD2 LYS A 310       8.930  39.481  15.820  1.00  0.00           H
ATOM   4433  CE  LYS A 310      10.446  40.255  17.146  1.00  0.00           C
ATOM   4434  HE1 LYS A 310      10.893  40.892  16.376  1.00  0.00           H
ATOM   4435  HE2 LYS A 310       9.703  40.854  17.679  1.00  0.00           H
ATOM   4436  NZ  LYS A 310      11.495  39.806  18.101  1.00  0.00           N
ATOM   4437  HZ1 LYS A 310      12.171  39.196  17.653  1.00  0.00           H
ATOM   4438  HZ2 LYS A 310      11.969  40.599  18.523  1.00  0.00           H
ATOM   4439  HZ3 LYS A 310      11.066  39.295  18.867  1.00  0.00           H
ATOM   4440  C   LYS A 310       7.381  34.612  17.418  1.00  0.00           C
ATOM   4441  O   LYS A 310       7.602  33.829  16.492  1.00  0.00           O
ATOM   4442  N   GLY A 311       6.291  34.508  18.186  1.00  0.00           N
ATOM   4443  H   GLY A 311       6.181  35.173  18.944  1.00  0.00           H
ATOM   4444  CA  GLY A 311       5.224  33.517  17.977  1.00  0.00           C
ATOM   4445  HA1 GLY A 311       4.357  33.804  18.573  1.00  0.00           H
ATOM   4446  HA2 GLY A 311       4.952  33.507  16.921  1.00  0.00           H
ATOM   4447  C   GLY A 311       5.596  32.080  18.369  1.00  0.00           C
ATOM   4448  O   GLY A 311       4.865  31.154  18.018  1.00  0.00           O
ATOM   4449  N   ALA A 312       6.725  31.862  19.048  1.00  0.00           N
ATOM   4450  H   ALA A 312       7.266  32.667  19.328  1.00  0.00           H
ATOM   4451  CA  ALA A 312       7.180  30.548  19.512  1.00  0.00           C
ATOM   4452  HA  ALA A 312       6.878  29.789  18.788  1.00  0.00           H
ATOM   4453  CB  ALA A 312       8.711  30.581  19.548  1.00  0.00           C
ATOM   4454  HB1 ALA A 312       9.093  29.578  19.725  1.00  0.00           H
ATOM   4455  HB2 ALA A 312       9.103  30.937  18.595  1.00  0.00           H
ATOM   4456  HB3 ALA A 312       9.051  31.243  20.345  1.00  0.00           H
ATOM   4457  C   ALA A 312       6.570  30.143  20.875  1.00  0.00           C
ATOM   4458  O   ALA A 312       5.913  30.956  21.535  1.00  0.00           O
ATOM   4459  N   THR A 313       6.791  28.888  21.288  1.00  0.00           N
ATOM   4460  H   THR A 313       7.318  28.277  20.672  1.00  0.00           H
ATOM   4461  CA  THR A 313       6.256  28.289  22.524  1.00  0.00           C
ATOM   4462  HA  THR A 313       5.850  29.066  23.170  1.00  0.00           H
ATOM   4463  CB  THR A 313       5.110  27.333  22.176  1.00  0.00           C
ATOM   4464  HB  THR A 313       5.472  26.549  21.509  1.00  0.00           H
ATOM   4465  CG2 THR A 313       4.489  26.695  23.416  1.00  0.00           C
ATOM   4466 HG21 THR A 313       3.626  26.097  23.124  1.00  0.00           H
ATOM   4467 HG22 THR A 313       5.209  26.042  23.909  1.00  0.00           H
ATOM   4468 HG23 THR A 313       4.165  27.470  24.111  1.00  0.00           H
ATOM   4469  OG1 THR A 313       4.088  28.067  21.539  1.00  0.00           O
ATOM   4470  HG1 THR A 313       4.243  28.073  20.588  1.00  0.00           H
ATOM   4471  C   THR A 313       7.372  27.574  23.284  1.00  0.00           C
ATOM   4472  O   THR A 313       7.987  26.651  22.753  1.00  0.00           O
ATOM   4473  N   LYS A 314       7.650  28.022  24.514  1.00  0.00           N
ATOM   4474  H   LYS A 314       7.043  28.744  24.888  1.00  0.00           H
ATOM   4475  CA  LYS A 314       8.782  27.607  25.362  1.00  0.00           C
ATOM   4476  HA  LYS A 314       9.455  26.990  24.767  1.00  0.00           H
ATOM   4477  CB  LYS A 314       9.549  28.886  25.784  1.00  0.00           C
ATOM   4478  HB1 LYS A 314       9.820  29.429  24.879  1.00  0.00           H
ATOM   4479  HB2 LYS A 314       8.874  29.521  26.361  1.00  0.00           H
```

ATOM	4480	CG	LYS	A	314	10.833	28.661	26.610	1.00	0.00	C
ATOM	4481	HG1	LYS	A	314	10.561	28.251	27.584	1.00	0.00	H
ATOM	4482	HG2	LYS	A	314	11.474	27.944	26.094	1.00	0.00	H
ATOM	4483	CD	LYS	A	314	11.622	29.966	26.836	1.00	0.00	C
ATOM	4484	HD1	LYS	A	314	12.002	30.328	25.879	1.00	0.00	H
ATOM	4485	HD2	LYS	A	314	10.956	30.723	27.255	1.00	0.00	H
ATOM	4486	CE	LYS	A	314	12.790	29.727	27.810	1.00	0.00	C
ATOM	4487	HE1	LYS	A	314	12.377	29.409	28.771	1.00	0.00	H
ATOM	4488	HE2	LYS	A	314	13.410	28.912	27.434	1.00	0.00	H
ATOM	4489	NZ	LYS	A	314	13.633	30.933	28.019	1.00	0.00	N
ATOM	4490	HZ1	LYS	A	314	14.045	31.277	27.155	1.00	0.00	H
ATOM	4491	HZ2	LYS	A	314	13.102	31.695	28.433	1.00	0.00	H
ATOM	4492	HZ3	LYS	A	314	14.399	30.725	28.657	1.00	0.00	H
ATOM	4493	C	LYS	A	314	8.341	26.762	26.579	1.00	0.00	C
ATOM	4494	O	LYS	A	314	7.221	26.886	27.082	1.00	0.00	O
ATOM	4495	N	GLN	A	315	9.252	25.922	27.078	1.00	0.00	N
ATOM	4496	H	GLN	A	315	10.147	25.857	26.605	1.00	0.00	H
ATOM	4497	CA	GLN	A	315	9.135	25.107	28.293	1.00	0.00	C
ATOM	4498	HA	GLN	A	315	8.814	25.748	29.115	1.00	0.00	H
ATOM	4499	CB	GLN	A	315	8.080	23.999	28.119	1.00	0.00	C
ATOM	4500	HB1	GLN	A	315	8.029	23.419	29.041	1.00	0.00	H
ATOM	4501	HB2	GLN	A	315	7.095	24.447	27.984	1.00	0.00	H
ATOM	4502	CG	GLN	A	315	8.373	23.031	26.962	1.00	0.00	C
ATOM	4503	HG1	GLN	A	315	9.438	23.009	26.739	1.00	0.00	H
ATOM	4504	HG2	GLN	A	315	8.068	22.035	27.277	1.00	0.00	H
ATOM	4505	CD	GLN	A	315	7.593	23.370	25.700	1.00	0.00	C
ATOM	4506	OE1	GLN	A	315	6.415	23.057	25.603	1.00	0.00	O
ATOM	4507	NE2	GLN	A	315	8.194	24.015	24.721	1.00	0.00	N
ATOM	4508	HE21	GLN	A	315	9.182	24.223	24.766	1.00	0.00	H
ATOM	4509	HE22	GLN	A	315	7.643	24.230	23.902	1.00	0.00	H
ATOM	4510	C	GLN	A	315	10.518	24.537	28.665	1.00	0.00	C
ATOM	4511	O	GLN	A	315	11.506	24.896	28.023	1.00	0.00	O
ATOM	4512	N	ARG	A	316	10.604	23.639	29.661	1.00	0.00	N
ATOM	4513	H	ARG	A	316	9.760	23.371	30.153	1.00	0.00	H
ATOM	4514	CA	ARG	A	316	11.868	23.028	30.108	1.00	0.00	C
ATOM	4515	HA	ARG	A	316	12.615	23.179	29.337	1.00	0.00	H
ATOM	4516	CB	ARG	A	316	12.373	23.686	31.411	1.00	0.00	C
ATOM	4517	HB1	ARG	A	316	11.735	23.370	32.235	1.00	0.00	H
ATOM	4518	HB2	ARG	A	316	13.389	23.340	31.609	1.00	0.00	H
ATOM	4519	CG	ARG	A	316	12.363	25.223	31.373	1.00	0.00	C
ATOM	4520	HG1	ARG	A	316	12.890	25.577	30.488	1.00	0.00	H
ATOM	4521	HG2	ARG	A	316	11.327	25.548	31.307	1.00	0.00	H
ATOM	4522	CD	ARG	A	316	12.956	25.874	32.631	1.00	0.00	C
ATOM	4523	HD1	ARG	A	316	12.476	26.842	32.784	1.00	0.00	H
ATOM	4524	HD2	ARG	A	316	12.731	25.259	33.505	1.00	0.00	H
ATOM	4525	NE	ARG	A	316	14.410	26.074	32.518	1.00	0.00	N
ATOM	4526	HE	ARG	A	316	15.011	25.449	33.042	1.00	0.00	H
ATOM	4527	CZ	ARG	A	316	15.018	27.088	31.913	1.00	0.00	C
ATOM	4528	NH1	ARG	A	316	16.324	27.142	31.950	1.00	0.00	N
ATOM	4529	HH11	ARG	A	316	16.828	26.482	32.530	1.00	0.00	H
ATOM	4530	HH12	ARG	A	316	16.796	27.968	31.585	1.00	0.00	H
ATOM	4531	NH2	ARG	A	316	14.379	28.045	31.275	1.00	0.00	N
ATOM	4532	HH21	ARG	A	316	13.367	28.035	31.211	1.00	0.00	H
ATOM	4533	HH22	ARG	A	316	14.903	28.833	30.899	1.00	0.00	H

```
ATOM   4534  C   ARG A 316      11.738  21.515  30.296  1.00  0.00           C
ATOM   4535  O   ARG A 316      10.730  21.028  30.808  1.00  0.00           O
ATOM   4536  N   ILE A 317      12.769  20.755  29.921  1.00  0.00           N
ATOM   4537  H   ILE A 317      13.579  21.229  29.539  1.00  0.00           H
ATOM   4538  CA  ILE A 317      12.847  19.300  30.147  1.00  0.00           C
ATOM   4539  HA  ILE A 317      11.892  18.851  29.870  1.00  0.00           H
ATOM   4540  CB  ILE A 317      13.944  18.624  29.282  1.00  0.00           C
ATOM   4541  HB  ILE A 317      14.921  18.920  29.665  1.00  0.00           H
ATOM   4542  CG2 ILE A 317      13.793  17.096  29.428  1.00  0.00           C
ATOM   4543 HG21 ILE A 317      14.589  16.581  28.895  1.00  0.00           H
ATOM   4544 HG22 ILE A 317      13.854  16.798  30.474  1.00  0.00           H
ATOM   4545 HG23 ILE A 317      12.834  16.773  29.021  1.00  0.00           H
ATOM   4546  CG1 ILE A 317      13.888  19.049  27.796  1.00  0.00           C
ATOM   4547 HG11 ILE A 317      12.911  18.797  27.384  1.00  0.00           H
ATOM   4548 HG12 ILE A 317      14.025  20.127  27.736  1.00  0.00           H
ATOM   4549  CD  ILE A 317      14.975  18.428  26.910  1.00  0.00           C
ATOM   4550  HD1 ILE A 317      14.943  18.894  25.925  1.00  0.00           H
ATOM   4551  HD2 ILE A 317      15.959  18.595  27.350  1.00  0.00           H
ATOM   4552  HD3 ILE A 317      14.809  17.359  26.787  1.00  0.00           H
ATOM   4553  C   ILE A 317      13.087  19.050  31.642  1.00  0.00           C
ATOM   4554  O   ILE A 317      14.197  19.284  32.122  1.00  0.00           O
ATOM   4555  N   GLN A 318      12.069  18.575  32.364  1.00  0.00           N
ATOM   4556  H   GLN A 318      11.179  18.433  31.894  1.00  0.00           H
ATOM   4557  CA  GLN A 318      12.078  18.446  33.830  1.00  0.00           C
ATOM   4558  HA  GLN A 318      12.303  19.428  34.253  1.00  0.00           H
ATOM   4559  CB  GLN A 318      10.672  18.037  34.313  1.00  0.00           C
ATOM   4560  HB1 GLN A 318      10.630  18.144  35.398  1.00  0.00           H
ATOM   4561  HB2 GLN A 318       9.952  18.737  33.888  1.00  0.00           H
ATOM   4562  CG  GLN A 318      10.248  16.598  33.950  1.00  0.00           C
ATOM   4563  HG1 GLN A 318      10.406  16.417  32.887  1.00  0.00           H
ATOM   4564  HG2 GLN A 318      10.863  15.893  34.508  1.00  0.00           H
ATOM   4565  CD  GLN A 318       8.773  16.330  34.258  1.00  0.00           C
ATOM   4566  OE1 GLN A 318       7.908  17.170  34.047  1.00  0.00           O
ATOM   4567  NE2 GLN A 318       8.411  15.163  34.737  1.00  0.00           N
ATOM   4568 HE21 GLN A 318       9.087  14.425  34.894  1.00  0.00           H
ATOM   4569 HE22 GLN A 318       7.424  15.018  34.915  1.00  0.00           H
ATOM   4570  C   GLN A 318      13.151  17.483  34.359  1.00  0.00           C
ATOM   4571  O   GLN A 318      13.620  17.637  35.487  1.00  0.00           O
ATOM   4572  N   ASP A 319      13.551  16.505  33.543  1.00  0.00           N
ATOM   4573  H   ASP A 319      13.057  16.398  32.670  1.00  0.00           H
ATOM   4574  CA  ASP A 319      14.606  15.538  33.846  1.00  0.00           C
ATOM   4575  HA  ASP A 319      14.534  15.230  34.888  1.00  0.00           H
ATOM   4576  CB  ASP A 319      14.426  14.312  32.934  1.00  0.00           C
ATOM   4577  HB1 ASP A 319      14.345  14.644  31.897  1.00  0.00           H
ATOM   4578  HB2 ASP A 319      15.318  13.693  33.010  1.00  0.00           H
ATOM   4579  CG  ASP A 319      13.223  13.435  33.278  1.00  0.00           C
ATOM   4580  OD1 ASP A 319      12.764  12.714  32.360  1.00  0.00           O
ATOM   4581  OD2 ASP A 319      12.792  13.455  34.457  1.00  0.00           O
ATOM   4582  C   ASP A 319      16.008  16.115  33.617  1.00  0.00           C
ATOM   4583  O   ASP A 319      16.920  15.878  34.410  1.00  0.00           O
ATOM   4584  N   GLN A 320      16.180  16.860  32.525  1.00  0.00           N
ATOM   4585  H   GLN A 320      15.358  17.097  31.991  1.00  0.00           H
ATOM   4586  CA  GLN A 320      17.490  17.242  31.992  1.00  0.00           C
ATOM   4587  HA  GLN A 320      18.258  16.616  32.448  1.00  0.00           H
```

ATOM	4588	CB	GLN	A	320	17.511	16.981	30.479	1.00	0.00	C
ATOM	4589	HB1	GLN	A	320	16.788	17.621	29.976	1.00	0.00	H
ATOM	4590	HB2	GLN	A	320	18.500	17.238	30.112	1.00	0.00	H
ATOM	4591	CG	GLN	A	320	17.212	15.509	30.144	1.00	0.00	C
ATOM	4592	HG1	GLN	A	320	17.806	14.869	30.790	1.00	0.00	H
ATOM	4593	HG2	GLN	A	320	16.165	15.288	30.344	1.00	0.00	H
ATOM	4594	CD	GLN	A	320	17.490	15.162	28.684	1.00	0.00	C
ATOM	4595	OE1	GLN	A	320	16.805	15.629	27.788	1.00	0.00	O
ATOM	4596	NE2	GLN	A	320	18.486	14.352	28.403	1.00	0.00	N
ATOM	4597	HE21	GLN	A	320	19.139	14.060	29.116	1.00	0.00	H
ATOM	4598	HE22	GLN	A	320	18.617	14.046	27.436	1.00	0.00	H
ATOM	4599	C	GLN	A	320	17.871	18.691	32.325	1.00	0.00	C
ATOM	4600	O	GLN	A	320	19.007	19.095	32.089	1.00	0.00	O
ATOM	4601	N	LYS	A	321	16.940	19.465	32.895	1.00	0.00	N
ATOM	4602	H	LYS	A	321	16.000	19.081	32.962	1.00	0.00	H
ATOM	4603	CA	LYS	A	321	17.172	20.735	33.603	1.00	0.00	C
ATOM	4604	HA	LYS	A	321	16.226	21.005	34.074	1.00	0.00	H
ATOM	4605	CB	LYS	A	321	18.216	20.574	34.735	1.00	0.00	C
ATOM	4606	HB1	LYS	A	321	19.217	20.607	34.305	1.00	0.00	H
ATOM	4607	HB2	LYS	A	321	18.116	21.435	35.396	1.00	0.00	H
ATOM	4608	CG	LYS	A	321	18.137	19.303	35.601	1.00	0.00	C
ATOM	4609	HG1	LYS	A	321	18.498	18.452	35.022	1.00	0.00	H
ATOM	4610	HG2	LYS	A	321	18.812	19.435	36.447	1.00	0.00	H
ATOM	4611	CD	LYS	A	321	16.743	18.973	36.143	1.00	0.00	C
ATOM	4612	HD1	LYS	A	321	16.354	19.824	36.699	1.00	0.00	H
ATOM	4613	HD2	LYS	A	321	16.066	18.745	35.320	1.00	0.00	H
ATOM	4614	CE	LYS	A	321	16.852	17.753	37.061	1.00	0.00	C
ATOM	4615	HE1	LYS	A	321	17.298	16.928	36.501	1.00	0.00	H
ATOM	4616	HE2	LYS	A	321	17.515	17.991	37.899	1.00	0.00	H
ATOM	4617	NZ	LYS	A	321	15.532	17.337	37.577	1.00	0.00	N
ATOM	4618	HZ1	LYS	A	321	15.610	16.506	38.160	1.00	0.00	H
ATOM	4619	HZ2	LYS	A	321	15.116	18.059	38.144	1.00	0.00	H
ATOM	4620	HZ3	LYS	A	321	14.891	17.135	36.814	1.00	0.00	H
ATOM	4621	C	LYS	A	321	17.499	21.900	32.646	1.00	0.00	C
ATOM	4622	O	LYS	A	321	18.242	22.821	32.998	1.00	0.00	O
ATOM	4623	N	VAL	A	322	16.974	21.835	31.422	1.00	0.00	N
ATOM	4624	H	VAL	A	322	16.383	21.033	31.254	1.00	0.00	H
ATOM	4625	CA	VAL	A	322	17.307	22.701	30.269	1.00	0.00	C
ATOM	4626	HA	VAL	A	322	17.741	23.623	30.653	1.00	0.00	H
ATOM	4627	CB	VAL	A	322	18.337	22.022	29.333	1.00	0.00	C
ATOM	4628	HB	VAL	A	322	18.421	22.639	28.443	1.00	0.00	H
ATOM	4629	CG1	VAL	A	322	19.731	21.963	29.968	1.00	0.00	C
ATOM	4630	HG11	VAL	A	322	20.443	21.585	29.237	1.00	0.00	H
ATOM	4631	HG12	VAL	A	322	20.043	22.962	30.267	1.00	0.00	H
ATOM	4632	HG13	VAL	A	322	19.730	21.305	30.834	1.00	0.00	H
ATOM	4633	CG2	VAL	A	322	17.904	20.604	28.907	1.00	0.00	C
ATOM	4634	HG21	VAL	A	322	18.670	20.155	28.279	1.00	0.00	H
ATOM	4635	HG22	VAL	A	322	17.762	19.962	29.774	1.00	0.00	H
ATOM	4636	HG23	VAL	A	322	16.973	20.648	28.342	1.00	0.00	H
ATOM	4637	C	VAL	A	322	16.056	23.055	29.439	1.00	0.00	C
ATOM	4638	O	VAL	A	322	15.119	22.251	29.432	1.00	0.00	O
ATOM	4639	N	PRO	A	323	16.007	24.220	28.758	1.00	0.00	N
ATOM	4640	CD	PRO	A	323	16.992	25.297	28.824	1.00	0.00	C
ATOM	4641	HD1	PRO	A	323	17.968	24.968	28.472	1.00	0.00	H

ATOM	4642	HD2	PRO	A	323	17.074	25.650	29.851	1.00	0.00	H
ATOM	4643	CG	PRO	A	323	16.456	26.429	27.943	1.00	0.00	C
ATOM	4644	HG1	PRO	A	323	16.818	26.321	26.922	1.00	0.00	H
ATOM	4645	HG2	PRO	A	323	16.721	27.409	28.340	1.00	0.00	H
ATOM	4646	CB	PRO	A	323	14.948	26.193	27.970	1.00	0.00	C
ATOM	4647	HB1	PRO	A	323	14.443	26.619	27.107	1.00	0.00	H
ATOM	4648	HB2	PRO	A	323	14.532	26.613	28.886	1.00	0.00	H
ATOM	4649	CA	PRO	A	323	14.833	24.669	28.012	1.00	0.00	C
ATOM	4650	HA	PRO	A	323	13.937	24.431	28.571	1.00	0.00	H
ATOM	4651	C	PRO	A	323	14.711	24.051	26.606	1.00	0.00	C
ATOM	4652	O	PRO	A	323	15.695	23.629	26.000	1.00	0.00	O
ATOM	4653	N	TYR	A	324	13.480	24.045	26.075	1.00	0.00	N
ATOM	4654	H	TYR	A	324	12.716	24.395	26.650	1.00	0.00	H
ATOM	4655	CA	TYR	A	324	13.157	23.713	24.678	1.00	0.00	C
ATOM	4656	HA	TYR	A	324	14.027	23.989	24.081	1.00	0.00	H
ATOM	4657	CB	TYR	A	324	12.965	22.196	24.483	1.00	0.00	C
ATOM	4658	HB1	TYR	A	324	12.982	21.995	23.412	1.00	0.00	H
ATOM	4659	HB2	TYR	A	324	13.837	21.687	24.896	1.00	0.00	H
ATOM	4660	CG	TYR	A	324	11.719	21.540	25.069	1.00	0.00	C
ATOM	4661	CD1	TYR	A	324	11.654	21.257	26.446	1.00	0.00	C
ATOM	4662	HD1	TYR	A	324	12.444	21.605	27.093	1.00	0.00	H
ATOM	4663	CE1	TYR	A	324	10.583	20.506	26.971	1.00	0.00	C
ATOM	4664	HE1	TYR	A	324	10.540	20.273	28.023	1.00	0.00	H
ATOM	4665	CZ	TYR	A	324	9.547	20.056	26.123	1.00	0.00	C
ATOM	4666	OH	TYR	A	324	8.505	19.354	26.654	1.00	0.00	O
ATOM	4667	HH	TYR	A	324	7.805	19.160	26.018	1.00	0.00	H
ATOM	4668	CE2	TYR	A	324	9.585	20.375	24.747	1.00	0.00	C
ATOM	4669	HE2	TYR	A	324	8.792	20.047	24.089	1.00	0.00	H
ATOM	4670	CD2	TYR	A	324	10.673	21.105	24.227	1.00	0.00	C
ATOM	4671	HD2	TYR	A	324	10.712	21.313	23.168	1.00	0.00	H
ATOM	4672	C	TYR	A	324	11.978	24.549	24.133	1.00	0.00	C
ATOM	4673	O	TYR	A	324	11.047	24.909	24.866	1.00	0.00	O
ATOM	4674	N	ILE	A	325	12.038	24.914	22.848	1.00	0.00	N
ATOM	4675	H	ILE	A	325	12.755	24.496	22.262	1.00	0.00	H
ATOM	4676	CA	ILE	A	325	11.171	25.915	22.202	1.00	0.00	C
ATOM	4677	HA	ILE	A	325	10.213	25.905	22.715	1.00	0.00	H
ATOM	4678	CB	ILE	A	325	11.792	27.329	22.366	1.00	0.00	C
ATOM	4679	HB	ILE	A	325	12.012	27.466	23.425	1.00	0.00	H
ATOM	4680	CG2	ILE	A	325	13.113	27.485	21.587	1.00	0.00	C
ATOM	4681	HG21	ILE	A	325	13.645	28.377	21.917	1.00	0.00	H
ATOM	4682	HG22	ILE	A	325	13.760	26.628	21.764	1.00	0.00	H
ATOM	4683	HG23	ILE	A	325	12.919	27.566	20.518	1.00	0.00	H
ATOM	4684	CG1	ILE	A	325	10.795	28.439	21.982	1.00	0.00	C
ATOM	4685	HG11	ILE	A	325	10.528	28.338	20.933	1.00	0.00	H
ATOM	4686	HG12	ILE	A	325	9.894	28.317	22.575	1.00	0.00	H
ATOM	4687	CD	ILE	A	325	11.315	29.858	22.239	1.00	0.00	C
ATOM	4688	HD1	ILE	A	325	10.520	30.575	22.043	1.00	0.00	H
ATOM	4689	HD2	ILE	A	325	11.623	29.952	23.280	1.00	0.00	H
ATOM	4690	HD3	ILE	A	325	12.156	30.091	21.587	1.00	0.00	H
ATOM	4691	C	ILE	A	325	10.885	25.558	20.735	1.00	0.00	C
ATOM	4692	O	ILE	A	325	11.788	25.167	19.990	1.00	0.00	O
ATOM	4693	N	PHE	A	326	9.630	25.708	20.309	1.00	0.00	N
ATOM	4694	H	PHE	A	326	8.929	26.027	20.968	1.00	0.00	H
ATOM	4695	CA	PHE	A	326	9.171	25.350	18.963	1.00	0.00	C

ATOM	4696	HA	PHE	A	326	10.034	25.302	18.298	1.00	0.00	H
ATOM	4697	CB	PHE	A	326	8.548	23.940	18.989	1.00	0.00	C
ATOM	4698	HB1	PHE	A	326	8.084	23.731	18.024	1.00	0.00	H
ATOM	4699	HB2	PHE	A	326	9.362	23.224	19.103	1.00	0.00	H
ATOM	4700	CG	PHE	A	326	7.527	23.670	20.082	1.00	0.00	C
ATOM	4701	CD1	PHE	A	326	6.223	24.190	19.987	1.00	0.00	C
ATOM	4702	HD1	PHE	A	326	5.946	24.807	19.146	1.00	0.00	H
ATOM	4703	CE1	PHE	A	326	5.273	23.907	20.985	1.00	0.00	C
ATOM	4704	HE1	PHE	A	326	4.273	24.312	20.911	1.00	0.00	H
ATOM	4705	CZ	PHE	A	326	5.623	23.102	22.082	1.00	0.00	C
ATOM	4706	HZ	PHE	A	326	4.896	22.887	22.854	1.00	0.00	H
ATOM	4707	CE2	PHE	A	326	6.920	22.571	22.175	1.00	0.00	C
ATOM	4708	HE2	PHE	A	326	7.182	21.935	23.008	1.00	0.00	H
ATOM	4709	CD2	PHE	A	326	7.873	22.861	21.183	1.00	0.00	C
ATOM	4710	HD2	PHE	A	326	8.868	22.447	21.264	1.00	0.00	H
ATOM	4711	C	PHE	A	326	8.238	26.408	18.353	1.00	0.00	C
ATOM	4712	O	PHE	A	326	7.664	27.242	19.061	1.00	0.00	O
ATOM	4713	N	ARG	A	327	8.104	26.371	17.021	1.00	0.00	N
ATOM	4714	H	ARG	A	327	8.615	25.642	16.527	1.00	0.00	H
ATOM	4715	CA	ARG	A	327	7.309	27.298	16.210	1.00	0.00	C
ATOM	4716	HA	ARG	A	327	6.378	27.499	16.743	1.00	0.00	H
ATOM	4717	CB	ARG	A	327	8.070	28.629	16.048	1.00	0.00	C
ATOM	4718	HB1	ARG	A	327	8.399	28.979	17.027	1.00	0.00	H
ATOM	4719	HB2	ARG	A	327	8.955	28.474	15.433	1.00	0.00	H
ATOM	4720	CG	ARG	A	327	7.173	29.707	15.424	1.00	0.00	C
ATOM	4721	HG1	ARG	A	327	6.823	29.380	14.446	1.00	0.00	H
ATOM	4722	HG2	ARG	A	327	6.307	29.832	16.073	1.00	0.00	H
ATOM	4723	CD	ARG	A	327	7.859	31.072	15.283	1.00	0.00	C
ATOM	4724	HD1	ARG	A	327	7.095	31.807	15.026	1.00	0.00	H
ATOM	4725	HD2	ARG	A	327	8.304	31.350	16.240	1.00	0.00	H
ATOM	4726	NE	ARG	A	327	8.891	31.083	14.233	1.00	0.00	N
ATOM	4727	HE	ARG	A	327	9.044	30.212	13.737	1.00	0.00	H
ATOM	4728	CZ	ARG	A	327	9.641	32.120	13.877	1.00	0.00	C
ATOM	4729	NH1	ARG	A	327	10.548	31.968	12.936	1.00	0.00	N
ATOM	4730	HH11	ARG	A	327	10.671	31.073	12.471	1.00	0.00	H
ATOM	4731	HH12	ARG	A	327	11.146	32.732	12.662	1.00	0.00	H
ATOM	4732	NH2	ARG	A	327	9.522	33.299	14.454	1.00	0.00	N
ATOM	4733	HH21	ARG	A	327	8.797	33.456	15.145	1.00	0.00	H
ATOM	4734	HH22	ARG	A	327	10.149	34.065	14.242	1.00	0.00	H
ATOM	4735	C	ARG	A	327	6.969	26.657	14.855	1.00	0.00	C
ATOM	4736	O	ARG	A	327	7.859	26.390	14.042	1.00	0.00	O
ATOM	4737	N	ASP	A	328	5.680	26.412	14.617	1.00	0.00	N
ATOM	4738	H	ASP	A	328	5.029	26.665	15.353	1.00	0.00	H
ATOM	4739	CA	ASP	A	328	5.141	25.606	13.511	1.00	0.00	C
ATOM	4740	HA	ASP	A	328	4.117	25.335	13.774	1.00	0.00	H
ATOM	4741	CB	ASP	A	328	5.059	26.439	12.213	1.00	0.00	C
ATOM	4742	HB1	ASP	A	328	6.039	26.859	11.978	1.00	0.00	H
ATOM	4743	HB2	ASP	A	328	4.761	25.788	11.391	1.00	0.00	H
ATOM	4744	CG	ASP	A	328	4.018	27.558	12.306	1.00	0.00	C
ATOM	4745	OD1	ASP	A	328	2.922	27.321	12.866	1.00	0.00	O
ATOM	4746	OD2	ASP	A	328	4.266	28.673	11.797	1.00	0.00	O
ATOM	4747	C	ASP	A	328	5.876	24.255	13.391	1.00	0.00	C
ATOM	4748	O	ASP	A	328	5.871	23.483	14.351	1.00	0.00	O
ATOM	4749	N	ASN	A	329	6.511	23.961	12.253	1.00	0.00	N

ATOM	4750	H	ASN	A	329	6.464	24.622	11.489	1.00	0.00	H
ATOM	4751	CA	ASN	A	329	7.295	22.739	12.043	1.00	0.00	C
ATOM	4752	HA	ASN	A	329	6.817	21.939	12.609	1.00	0.00	H
ATOM	4753	CB	ASN	A	329	7.229	22.337	10.557	1.00	0.00	C
ATOM	4754	HB1	ASN	A	329	7.559	21.300	10.475	1.00	0.00	H
ATOM	4755	HB2	ASN	A	329	6.197	22.373	10.208	1.00	0.00	H
ATOM	4756	CG	ASN	A	329	8.101	23.188	9.633	1.00	0.00	C
ATOM	4757	OD1	ASN	A	329	8.435	24.337	9.908	1.00	0.00	O
ATOM	4758	ND2	ASN	A	329	8.530	22.637	8.517	1.00	0.00	N
ATOM	4759	HD21	ASN	A	329	8.282	21.678	8.288	1.00	0.00	H
ATOM	4760	HD22	ASN	A	329	9.125	23.173	7.898	1.00	0.00	H
ATOM	4761	C	ASN	A	329	8.757	22.824	12.542	1.00	0.00	C
ATOM	4762	O	ASN	A	329	9.520	21.885	12.321	1.00	0.00	O
ATOM	4763	N	GLN	A	330	9.184	23.926	13.167	1.00	0.00	N
ATOM	4764	H	GLN	A	330	8.509	24.651	13.386	1.00	0.00	H
ATOM	4765	CA	GLN	A	330	10.555	24.107	13.659	1.00	0.00	C
ATOM	4766	HA	GLN	A	330	11.214	23.370	13.196	1.00	0.00	H
ATOM	4767	CB	GLN	A	330	11.076	25.509	13.293	1.00	0.00	C
ATOM	4768	HB1	GLN	A	330	10.449	26.264	13.765	1.00	0.00	H
ATOM	4769	HB2	GLN	A	330	12.082	25.620	13.700	1.00	0.00	H
ATOM	4770	CG	GLN	A	330	11.130	25.776	11.777	1.00	0.00	C
ATOM	4771	HG1	GLN	A	330	10.123	25.762	11.363	1.00	0.00	H
ATOM	4772	HG2	GLN	A	330	11.544	26.768	11.608	1.00	0.00	H
ATOM	4773	CD	GLN	A	330	12.013	24.769	11.047	1.00	0.00	C
ATOM	4774	OE1	GLN	A	330	13.210	24.694	11.289	1.00	0.00	O
ATOM	4775	NE2	GLN	A	330	11.469	23.933	10.191	1.00	0.00	N
ATOM	4776	HE21	GLN	A	330	10.497	24.027	9.931	1.00	0.00	H
ATOM	4777	HE22	GLN	A	330	12.078	23.265	9.738	1.00	0.00	H
ATOM	4778	C	GLN	A	330	10.626	23.876	15.174	1.00	0.00	C
ATOM	4779	O	GLN	A	330	9.728	24.270	15.915	1.00	0.00	O
ATOM	4780	N	TRP	A	331	11.710	23.245	15.631	1.00	0.00	N
ATOM	4781	H	TRP	A	331	12.412	22.956	14.960	1.00	0.00	H
ATOM	4782	CA	TRP	A	331	11.912	22.794	17.012	1.00	0.00	C
ATOM	4783	HA	TRP	A	331	11.240	23.327	17.685	1.00	0.00	H
ATOM	4784	CB	TRP	A	331	11.591	21.290	17.042	1.00	0.00	C
ATOM	4785	HB1	TRP	A	331	10.665	21.130	16.487	1.00	0.00	H
ATOM	4786	HB2	TRP	A	331	12.383	20.762	16.513	1.00	0.00	H
ATOM	4787	CG	TRP	A	331	11.422	20.641	18.380	1.00	0.00	C
ATOM	4788	CD1	TRP	A	331	10.227	20.356	18.940	1.00	0.00	C
ATOM	4789	HD1	TRP	A	331	9.263	20.575	18.496	1.00	0.00	H
ATOM	4790	NE1	TRP	A	331	10.415	19.723	20.147	1.00	0.00	N
ATOM	4791	HE1	TRP	A	331	9.635	19.399	20.721	1.00	0.00	H
ATOM	4792	CE2	TRP	A	331	11.749	19.575	20.447	1.00	0.00	C
ATOM	4793	CZ2	TRP	A	331	12.423	19.062	21.563	1.00	0.00	C
ATOM	4794	HZ2	TRP	A	331	11.865	18.649	22.390	1.00	0.00	H
ATOM	4795	CH2	TRP	A	331	13.826	19.107	21.594	1.00	0.00	C
ATOM	4796	HH2	TRP	A	331	14.364	18.714	22.446	1.00	0.00	H
ATOM	4797	CZ3	TRP	A	331	14.529	19.657	20.507	1.00	0.00	C
ATOM	4798	HZ3	TRP	A	331	15.609	19.683	20.527	1.00	0.00	H
ATOM	4799	CE3	TRP	A	331	13.842	20.164	19.386	1.00	0.00	C
ATOM	4800	HE3	TRP	A	331	14.406	20.575	18.561	1.00	0.00	H
ATOM	4801	CD2	TRP	A	331	12.430	20.152	19.329	1.00	0.00	C
ATOM	4802	C	TRP	A	331	13.366	23.043	17.435	1.00	0.00	C
ATOM	4803	O	TRP	A	331	14.273	22.708	16.671	1.00	0.00	O

ATOM	4804	N	VAL	A	332	13.605	23.601	18.627	1.00	0.00	N
ATOM	4805	H	VAL	A	332	12.813	23.944	19.184	1.00	0.00	H
ATOM	4806	CA	VAL	A	332	14.957	23.953	19.097	1.00	0.00	C
ATOM	4807	HA	VAL	A	332	15.678	23.452	18.457	1.00	0.00	H
ATOM	4808	CB	VAL	A	332	15.223	25.477	18.993	1.00	0.00	C
ATOM	4809	HB	VAL	A	332	14.600	25.987	19.722	1.00	0.00	H
ATOM	4810	CG1	VAL	A	332	16.693	25.816	19.301	1.00	0.00	C
ATOM	4811	HG11	VAL	A	332	16.843	26.894	19.263	1.00	0.00	H
ATOM	4812	HG12	VAL	A	332	16.959	25.477	20.299	1.00	0.00	H
ATOM	4813	HG13	VAL	A	332	17.351	25.342	18.573	1.00	0.00	H
ATOM	4814	CG2	VAL	A	332	14.887	26.049	17.602	1.00	0.00	C
ATOM	4815	HG21	VAL	A	332	15.111	27.116	17.578	1.00	0.00	H
ATOM	4816	HG22	VAL	A	332	15.473	25.543	16.835	1.00	0.00	H
ATOM	4817	HG23	VAL	A	332	13.827	25.926	17.385	1.00	0.00	H
ATOM	4818	C	VAL	A	332	15.190	23.468	20.534	1.00	0.00	C
ATOM	4819	O	VAL	A	332	14.365	23.690	21.422	1.00	0.00	O
ATOM	4820	N	GLY	A	333	16.346	22.831	20.760	1.00	0.00	N
ATOM	4821	H	GLY	A	333	16.958	22.700	19.960	1.00	0.00	H
ATOM	4822	CA	GLY	A	333	16.911	22.555	22.089	1.00	0.00	C
ATOM	4823	HA1	GLY	A	333	16.163	22.694	22.871	1.00	0.00	H
ATOM	4824	HA2	GLY	A	333	17.289	21.534	22.125	1.00	0.00	H
ATOM	4825	C	GLY	A	333	18.095	23.486	22.348	1.00	0.00	C
ATOM	4826	O	GLY	A	333	18.978	23.588	21.497	1.00	0.00	O
ATOM	4827	N	PHE	A	334	18.108	24.175	23.492	1.00	0.00	N
ATOM	4828	H	PHE	A	334	17.371	24.017	24.167	1.00	0.00	H
ATOM	4829	CA	PHE	A	334	19.000	25.313	23.757	1.00	0.00	C
ATOM	4830	HA	PHE	A	334	19.974	25.107	23.312	1.00	0.00	H
ATOM	4831	CB	PHE	A	334	18.414	26.564	23.074	1.00	0.00	C
ATOM	4832	HB1	PHE	A	334	19.191	27.325	23.016	1.00	0.00	H
ATOM	4833	HB2	PHE	A	334	18.142	26.325	22.049	1.00	0.00	H
ATOM	4834	CG	PHE	A	334	17.196	27.155	23.761	1.00	0.00	C
ATOM	4835	CD1	PHE	A	334	15.949	26.507	23.693	1.00	0.00	C
ATOM	4836	HD1	PHE	A	334	15.840	25.587	23.140	1.00	0.00	H
ATOM	4837	CE1	PHE	A	334	14.835	27.058	24.348	1.00	0.00	C
ATOM	4838	HE1	PHE	A	334	13.887	26.552	24.311	1.00	0.00	H
ATOM	4839	CZ	PHE	A	334	14.951	28.270	25.044	1.00	0.00	C
ATOM	4840	HZ	PHE	A	334	14.086	28.707	25.513	1.00	0.00	H
ATOM	4841	CE2	PHE	A	334	16.199	28.903	25.139	1.00	0.00	C
ATOM	4842	HE2	PHE	A	334	16.303	29.810	25.713	1.00	0.00	H
ATOM	4843	CD2	PHE	A	334	17.316	28.353	24.489	1.00	0.00	C
ATOM	4844	HD2	PHE	A	334	18.271	28.850	24.553	1.00	0.00	H
ATOM	4845	C	PHE	A	334	19.241	25.548	25.261	1.00	0.00	C
ATOM	4846	O	PHE	A	334	18.713	24.820	26.098	1.00	0.00	O
ATOM	4847	N	ASP	A	335	20.018	26.576	25.605	1.00	0.00	N
ATOM	4848	H	ASP	A	335	20.404	27.150	24.862	1.00	0.00	H
ATOM	4849	CA	ASP	A	335	20.320	26.991	26.981	1.00	0.00	C
ATOM	4850	HA	ASP	A	335	19.766	26.373	27.688	1.00	0.00	H
ATOM	4851	CB	ASP	A	335	21.822	26.804	27.241	1.00	0.00	C
ATOM	4852	HB1	ASP	A	335	22.394	27.014	26.335	1.00	0.00	H
ATOM	4853	HB2	ASP	A	335	22.135	27.523	27.999	1.00	0.00	H
ATOM	4854	CG	ASP	A	335	22.148	25.408	27.765	1.00	0.00	C
ATOM	4855	OD1	ASP	A	335	22.548	24.524	26.973	1.00	0.00	O
ATOM	4856	OD2	ASP	A	335	22.036	25.213	28.998	1.00	0.00	O
ATOM	4857	C	ASP	A	335	19.921	28.454	27.250	1.00	0.00	C

ATOM	4858	O	ASP	A	335	19.926	29.289	26.344	1.00	0.00	O
ATOM	4859	N	ASP	A	336	19.627	28.779	28.512	1.00	0.00	N
ATOM	4860	H	ASP	A	336	19.751	28.075	29.236	1.00	0.00	H
ATOM	4861	CA	ASP	A	336	19.345	30.143	28.970	1.00	0.00	C
ATOM	4862	HA	ASP	A	336	19.843	30.821	28.280	1.00	0.00	H
ATOM	4863	CB	ASP	A	336	17.830	30.442	28.852	1.00	0.00	C
ATOM	4864	HB1	ASP	A	336	17.715	31.516	28.723	1.00	0.00	H
ATOM	4865	HB2	ASP	A	336	17.454	29.992	27.932	1.00	0.00	H
ATOM	4866	CG	ASP	A	336	16.918	30.008	30.009	1.00	0.00	C
ATOM	4867	OD1	ASP	A	336	17.394	29.509	31.052	1.00	0.00	O
ATOM	4868	OD2	ASP	A	336	15.684	30.213	29.882	1.00	0.00	O
ATOM	4869	C	ASP	A	336	19.974	30.429	30.351	1.00	0.00	C
ATOM	4870	O	ASP	A	336	20.673	29.577	30.904	1.00	0.00	O
ATOM	4871	N	VAL	A	337	19.735	31.617	30.921	1.00	0.00	N
ATOM	4872	H	VAL	A	337	19.120	32.277	30.444	1.00	0.00	H
ATOM	4873	CA	VAL	A	337	20.370	32.059	32.180	1.00	0.00	C
ATOM	4874	HA	VAL	A	337	21.445	32.108	31.998	1.00	0.00	H
ATOM	4875	CB	VAL	A	337	19.890	33.487	32.549	1.00	0.00	C
ATOM	4876	HB	VAL	A	337	18.809	33.531	32.418	1.00	0.00	H
ATOM	4877	CG1	VAL	A	337	20.178	33.928	33.992	1.00	0.00	C
ATOM	4878	HG11	VAL	A	337	19.845	34.956	34.142	1.00	0.00	H
ATOM	4879	HG12	VAL	A	337	19.636	33.295	34.692	1.00	0.00	H
ATOM	4880	HG13	VAL	A	337	21.245	33.872	34.194	1.00	0.00	H
ATOM	4881	CG2	VAL	A	337	20.556	34.511	31.610	1.00	0.00	C
ATOM	4882	HG21	VAL	A	337	20.167	35.508	31.810	1.00	0.00	H
ATOM	4883	HG22	VAL	A	337	21.635	34.513	31.768	1.00	0.00	H
ATOM	4884	HG23	VAL	A	337	20.358	34.268	30.567	1.00	0.00	H
ATOM	4885	C	VAL	A	337	20.157	31.068	33.340	1.00	0.00	C
ATOM	4886	O	VAL	A	337	21.061	30.913	34.159	1.00	0.00	O
ATOM	4887	N	GLU	A	338	19.023	30.358	33.406	1.00	0.00	N
ATOM	4888	H	GLU	A	338	18.362	30.411	32.635	1.00	0.00	H
ATOM	4889	CA	GLU	A	338	18.728	29.439	34.514	1.00	0.00	C
ATOM	4890	HA	GLU	A	338	19.124	29.872	35.431	1.00	0.00	H
ATOM	4891	CB	GLU	A	338	17.206	29.330	34.692	1.00	0.00	C
ATOM	4892	HB1	GLU	A	338	16.769	30.327	34.638	1.00	0.00	H
ATOM	4893	HB2	GLU	A	338	16.798	28.733	33.883	1.00	0.00	H
ATOM	4894	CG	GLU	A	338	16.817	28.708	36.041	1.00	0.00	C
ATOM	4895	HG1	GLU	A	338	17.443	27.839	36.239	1.00	0.00	H
ATOM	4896	HG2	GLU	A	338	16.992	29.436	36.835	1.00	0.00	H
ATOM	4897	CD	GLU	A	338	15.364	28.245	36.095	1.00	0.00	C
ATOM	4898	OE1	GLU	A	338	15.095	27.296	36.874	1.00	0.00	O
ATOM	4899	OE2	GLU	A	338	14.498	28.781	35.366	1.00	0.00	O
ATOM	4900	C	GLU	A	338	19.382	28.055	34.325	1.00	0.00	C
ATOM	4901	O	GLU	A	338	19.772	27.409	35.304	1.00	0.00	O
ATOM	4902	N	SER	A	339	19.575	27.586	33.087	1.00	0.00	N
ATOM	4903	H	SER	A	339	19.367	28.178	32.286	1.00	0.00	H
ATOM	4904	CA	SER	A	339	20.344	26.354	32.862	1.00	0.00	C
ATOM	4905	HA	SER	A	339	20.068	25.628	33.625	1.00	0.00	H
ATOM	4906	CB	SER	A	339	20.006	25.697	31.519	1.00	0.00	C
ATOM	4907	HB1	SER	A	339	20.587	24.779	31.432	1.00	0.00	H
ATOM	4908	HB2	SER	A	339	18.947	25.436	31.513	1.00	0.00	H
ATOM	4909	OG	SER	A	339	20.279	26.518	30.405	1.00	0.00	O
ATOM	4910	HG	SER	A	339	20.975	26.060	29.872	1.00	0.00	H
ATOM	4911	C	SER	A	339	21.849	26.597	33.038	1.00	0.00	C

ATOM	4912	O	SER	A	339	22.516	25.793	33.688	1.00	0.00	O
ATOM	4913	N	PHE	A	340	22.374	27.745	32.591	1.00	0.00	N
ATOM	4914	H	PHE	A	340	21.782	28.366	32.045	1.00	0.00	H
ATOM	4915	CA	PHE	A	340	23.746	28.179	32.883	1.00	0.00	C
ATOM	4916	HA	PHE	A	340	24.440	27.433	32.492	1.00	0.00	H
ATOM	4917	CB	PHE	A	340	24.021	29.521	32.176	1.00	0.00	C
ATOM	4918	HB1	PHE	A	340	23.095	30.091	32.079	1.00	0.00	H
ATOM	4919	HB2	PHE	A	340	24.685	30.110	32.810	1.00	0.00	H
ATOM	4920	CG	PHE	A	340	24.674	29.402	30.807	1.00	0.00	C
ATOM	4921	CD1	PHE	A	340	24.222	28.461	29.860	1.00	0.00	C
ATOM	4922	HD1	PHE	A	340	23.377	27.824	30.080	1.00	0.00	H
ATOM	4923	CE1	PHE	A	340	24.864	28.348	28.614	1.00	0.00	C
ATOM	4924	HE1	PHE	A	340	24.515	27.626	27.891	1.00	0.00	H
ATOM	4925	CZ	PHE	A	340	25.954	29.176	28.301	1.00	0.00	C
ATOM	4926	HZ	PHE	A	340	26.437	29.092	27.338	1.00	0.00	H
ATOM	4927	CE2	PHE	A	340	26.411	30.114	29.240	1.00	0.00	C
ATOM	4928	HE2	PHE	A	340	27.240	30.761	28.995	1.00	0.00	H
ATOM	4929	CD2	PHE	A	340	25.760	30.236	30.481	1.00	0.00	C
ATOM	4930	HD2	PHE	A	340	26.092	30.983	31.185	1.00	0.00	H
ATOM	4931	C	PHE	A	340	24.003	28.267	34.397	1.00	0.00	C
ATOM	4932	O	PHE	A	340	25.035	27.788	34.880	1.00	0.00	O
ATOM	4933	N	LYS	A	341	23.063	28.823	35.172	1.00	0.00	N
ATOM	4934	H	LYS	A	341	22.260	29.250	34.721	1.00	0.00	H
ATOM	4935	CA	LYS	A	341	23.109	28.807	36.642	1.00	0.00	C
ATOM	4936	HA	LYS	A	341	24.017	29.305	36.988	1.00	0.00	H
ATOM	4937	CB	LYS	A	341	21.893	29.563	37.204	1.00	0.00	C
ATOM	4938	HB1	LYS	A	341	21.002	29.292	36.639	1.00	0.00	H
ATOM	4939	HB2	LYS	A	341	21.734	29.277	38.244	1.00	0.00	H
ATOM	4940	CG	LYS	A	341	22.109	31.080	37.150	1.00	0.00	C
ATOM	4941	HG1	LYS	A	341	22.811	31.363	37.927	1.00	0.00	H
ATOM	4942	HG2	LYS	A	341	22.524	31.362	36.185	1.00	0.00	H
ATOM	4943	CD	LYS	A	341	20.803	31.835	37.379	1.00	0.00	C
ATOM	4944	HD1	LYS	A	341	20.080	31.525	36.626	1.00	0.00	H
ATOM	4945	HD2	LYS	A	341	20.414	31.588	38.365	1.00	0.00	H
ATOM	4946	CE	LYS	A	341	21.035	33.343	37.263	1.00	0.00	C
ATOM	4947	HE1	LYS	A	341	21.829	33.644	37.950	1.00	0.00	H
ATOM	4948	HE2	LYS	A	341	21.365	33.569	36.248	1.00	0.00	H
ATOM	4949	NZ	LYS	A	341	19.798	34.095	37.570	1.00	0.00	N
ATOM	4950	HZ1	LYS	A	341	19.010	33.695	37.070	1.00	0.00	H
ATOM	4951	HZ2	LYS	A	341	19.595	34.052	38.561	1.00	0.00	H
ATOM	4952	HZ3	LYS	A	341	19.900	35.071	37.308	1.00	0.00	H
ATOM	4953	C	LYS	A	341	23.176	27.370	37.173	1.00	0.00	C
ATOM	4954	O	LYS	A	341	24.169	27.003	37.800	1.00	0.00	O
ATOM	4955	N	THR	A	342	22.186	26.532	36.837	1.00	0.00	N
ATOM	4956	H	THR	A	342	21.443	26.918	36.264	1.00	0.00	H
ATOM	4957	CA	THR	A	342	22.075	25.115	37.252	1.00	0.00	C
ATOM	4958	HA	THR	A	342	21.912	25.075	38.331	1.00	0.00	H
ATOM	4959	CB	THR	A	342	20.855	24.489	36.564	1.00	0.00	C
ATOM	4960	HB	THR	A	342	20.991	24.502	35.481	1.00	0.00	H
ATOM	4961	CG2	THR	A	342	20.602	23.050	37.010	1.00	0.00	C
ATOM	4962	HG21	THR	A	342	19.684	22.685	36.554	1.00	0.00	H
ATOM	4963	HG22	THR	A	342	21.422	22.409	36.689	1.00	0.00	H
ATOM	4964	HG23	THR	A	342	20.509	23.001	38.094	1.00	0.00	H
ATOM	4965	OG1	THR	A	342	19.708	25.244	36.908	1.00	0.00	O

ATOM	4966	HG1	THR	A	342	19.652	26.002	36.287	1.00	0.00	H
ATOM	4967	C	THR	A	342	23.354	24.314	36.968	1.00	0.00	C
ATOM	4968	O	THR	A	342	23.791	23.529	37.812	1.00	0.00	O
ATOM	4969	N	LYS	A	343	23.997	24.552	35.822	1.00	0.00	N
ATOM	4970	H	LYS	A	343	23.534	25.138	35.133	1.00	0.00	H
ATOM	4971	CA	LYS	A	343	25.260	23.914	35.423	1.00	0.00	C
ATOM	4972	HA	LYS	A	343	25.195	22.842	35.618	1.00	0.00	H
ATOM	4973	CB	LYS	A	343	25.450	24.122	33.910	1.00	0.00	C
ATOM	4974	HB1	LYS	A	343	25.315	25.178	33.668	1.00	0.00	H
ATOM	4975	HB2	LYS	A	343	26.466	23.836	33.634	1.00	0.00	H
ATOM	4976	CG	LYS	A	343	24.476	23.266	33.082	1.00	0.00	C
ATOM	4977	HG1	LYS	A	343	24.769	22.219	33.169	1.00	0.00	H
ATOM	4978	HG2	LYS	A	343	23.461	23.371	33.462	1.00	0.00	H
ATOM	4979	CD	LYS	A	343	24.498	23.684	31.608	1.00	0.00	C
ATOM	4980	HD1	LYS	A	343	24.102	24.697	31.508	1.00	0.00	H
ATOM	4981	HD2	LYS	A	343	25.528	23.669	31.252	1.00	0.00	H
ATOM	4982	CE	LYS	A	343	23.649	22.716	30.778	1.00	0.00	C
ATOM	4983	HE1	LYS	A	343	24.012	21.699	30.947	1.00	0.00	H
ATOM	4984	HE2	LYS	A	343	22.609	22.771	31.110	1.00	0.00	H
ATOM	4985	NZ	LYS	A	343	23.730	23.018	29.332	1.00	0.00	N
ATOM	4986	HZ1	LYS	A	343	23.302	22.269	28.796	1.00	0.00	H
ATOM	4987	HZ2	LYS	A	343	23.223	23.872	29.102	1.00	0.00	H
ATOM	4988	HZ3	LYS	A	343	24.694	23.105	29.033	1.00	0.00	H
ATOM	4989	C	LYS	A	343	26.485	24.421	36.208	1.00	0.00	C
ATOM	4990	O	LYS	A	343	27.367	23.624	36.530	1.00	0.00	O
ATOM	4991	N	VAL	A	344	26.546	25.708	36.564	1.00	0.00	N
ATOM	4992	H	VAL	A	344	25.790	26.320	36.268	1.00	0.00	H
ATOM	4993	CA	VAL	A	344	27.603	26.238	37.450	1.00	0.00	C
ATOM	4994	HA	VAL	A	344	28.542	25.785	37.136	1.00	0.00	H
ATOM	4995	CB	VAL	A	344	27.776	27.767	37.303	1.00	0.00	C
ATOM	4996	HB	VAL	A	344	26.798	28.242	37.377	1.00	0.00	H
ATOM	4997	CG1	VAL	A	344	28.708	28.368	38.368	1.00	0.00	C
ATOM	4998	HG11	VAL	A	344	28.924	29.410	38.136	1.00	0.00	H
ATOM	4999	HG12	VAL	A	344	28.231	28.335	39.345	1.00	0.00	H
ATOM	5000	HG13	VAL	A	344	29.641	27.806	38.403	1.00	0.00	H
ATOM	5001	CG2	VAL	A	344	28.390	28.073	35.922	1.00	0.00	C
ATOM	5002	HG21	VAL	A	344	28.545	29.146	35.810	1.00	0.00	H
ATOM	5003	HG22	VAL	A	344	29.354	27.574	35.819	1.00	0.00	H
ATOM	5004	HG23	VAL	A	344	27.729	27.730	35.127	1.00	0.00	H
ATOM	5005	C	VAL	A	344	27.378	25.807	38.908	1.00	0.00	C
ATOM	5006	O	VAL	A	344	28.340	25.477	39.597	1.00	0.00	O
ATOM	5007	N	SER	A	345	26.129	25.698	39.364	1.00	0.00	N
ATOM	5008	H	SER	A	345	25.365	26.104	38.828	1.00	0.00	H
ATOM	5009	CA	SER	A	345	25.790	25.083	40.655	1.00	0.00	C
ATOM	5010	HA	SER	A	345	26.273	25.640	41.458	1.00	0.00	H
ATOM	5011	CB	SER	A	345	24.269	25.162	40.888	1.00	0.00	C
ATOM	5012	HB1	SER	A	345	23.752	24.568	40.135	1.00	0.00	H
ATOM	5013	HB2	SER	A	345	24.037	24.755	41.873	1.00	0.00	H
ATOM	5014	OG	SER	A	345	23.814	26.508	40.816	1.00	0.00	O
ATOM	5015	HG	SER	A	345	22.838	26.540	40.896	1.00	0.00	H
ATOM	5016	C	SER	A	345	26.308	23.631	40.721	1.00	0.00	C
ATOM	5017	O	SER	A	345	26.959	23.251	41.696	1.00	0.00	O
ATOM	5018	N	TYR	A	346	26.132	22.848	39.646	1.00	0.00	N
ATOM	5019	H	TYR	A	346	25.590	23.214	38.873	1.00	0.00	H

ATOM	5020	CA	TYR	A	346	26.686	21.492	39.517	1.00	0.00	C
ATOM	5021	HA	TYR	A	346	26.350	20.898	40.367	1.00	0.00	H
ATOM	5022	CB	TYR	A	346	26.123	20.840	38.241	1.00	0.00	C
ATOM	5023	HB1	TYR	A	346	25.037	20.808	38.314	1.00	0.00	H
ATOM	5024	HB2	TYR	A	346	26.364	21.463	37.383	1.00	0.00	H
ATOM	5025	CG	TYR	A	346	26.624	19.434	37.955	1.00	0.00	C
ATOM	5026	CD1	TYR	A	346	25.891	18.309	38.385	1.00	0.00	C
ATOM	5027	HD1	TYR	A	346	24.974	18.442	38.943	1.00	0.00	H
ATOM	5028	CE1	TYR	A	346	26.338	17.008	38.078	1.00	0.00	C
ATOM	5029	HE1	TYR	A	346	25.764	16.149	38.397	1.00	0.00	H
ATOM	5030	CZ	TYR	A	346	27.543	16.827	37.361	1.00	0.00	C
ATOM	5031	OH	TYR	A	346	28.016	15.582	37.079	1.00	0.00	O
ATOM	5032	HH	TYR	A	346	27.367	14.871	37.213	1.00	0.00	H
ATOM	5033	CE2	TYR	A	346	28.277	17.956	36.937	1.00	0.00	C
ATOM	5034	HE2	TYR	A	346	29.184	17.816	36.374	1.00	0.00	H
ATOM	5035	CD2	TYR	A	346	27.812	19.253	37.221	1.00	0.00	C
ATOM	5036	HD2	TYR	A	346	28.367	20.109	36.866	1.00	0.00	H
ATOM	5037	C	TYR	A	346	28.227	21.465	39.552	1.00	0.00	C
ATOM	5038	O	TYR	A	346	28.801	20.678	40.307	1.00	0.00	O
ATOM	5039	N	LEU	A	347	28.925	22.329	38.797	1.00	0.00	N
ATOM	5040	H	LEU	A	347	28.419	22.956	38.180	1.00	0.00	H
ATOM	5041	CA	LEU	A	347	30.401	22.319	38.764	1.00	0.00	C
ATOM	5042	HA	LEU	A	347	30.697	21.281	38.599	1.00	0.00	H
ATOM	5043	CB	LEU	A	347	30.920	23.114	37.540	1.00	0.00	C
ATOM	5044	HB1	LEU	A	347	31.824	22.628	37.182	1.00	0.00	H
ATOM	5045	HB2	LEU	A	347	30.184	22.993	36.744	1.00	0.00	H
ATOM	5046	CG	LEU	A	347	31.247	24.618	37.687	1.00	0.00	C
ATOM	5047	HG	LEU	A	347	30.486	25.100	38.295	1.00	0.00	H
ATOM	5048	CD1	LEU	A	347	32.632	24.881	38.308	1.00	0.00	C
ATOM	5049	HD11	LEU	A	347	32.819	25.954	38.352	1.00	0.00	H
ATOM	5050	HD12	LEU	A	347	32.691	24.494	39.322	1.00	0.00	H
ATOM	5051	HD13	LEU	A	347	33.411	24.415	37.705	1.00	0.00	H
ATOM	5052	CD2	LEU	A	347	31.240	25.262	36.292	1.00	0.00	C
ATOM	5053	HD21	LEU	A	347	31.446	26.329	36.377	1.00	0.00	H
ATOM	5054	HD22	LEU	A	347	31.997	24.800	35.658	1.00	0.00	H
ATOM	5055	HD23	LEU	A	347	30.264	25.133	35.823	1.00	0.00	H
ATOM	5056	C	LEU	A	347	31.020	22.695	40.123	1.00	0.00	C
ATOM	5057	O	LEU	A	347	32.015	22.092	40.529	1.00	0.00	O
ATOM	5058	N	LYS	A	348	30.383	23.611	40.864	1.00	0.00	N
ATOM	5059	H	LYS	A	348	29.582	24.078	40.453	1.00	0.00	H
ATOM	5060	CA	LYS	A	348	30.735	23.966	42.251	1.00	0.00	C
ATOM	5061	HA	LYS	A	348	31.815	24.111	42.327	1.00	0.00	H
ATOM	5062	CB	LYS	A	348	30.035	25.282	42.634	1.00	0.00	C
ATOM	5063	HB1	LYS	A	348	28.966	25.172	42.444	1.00	0.00	H
ATOM	5064	HB2	LYS	A	348	30.172	25.451	43.704	1.00	0.00	H
ATOM	5065	CG	LYS	A	348	30.584	26.515	41.895	1.00	0.00	C
ATOM	5066	HG1	LYS	A	348	31.657	26.589	42.077	1.00	0.00	H
ATOM	5067	HG2	LYS	A	348	30.432	26.404	40.822	1.00	0.00	H
ATOM	5068	CD	LYS	A	348	29.920	27.820	42.365	1.00	0.00	C
ATOM	5069	HD1	LYS	A	348	30.171	27.998	43.413	1.00	0.00	H
ATOM	5070	HD2	LYS	A	348	30.321	28.642	41.769	1.00	0.00	H
ATOM	5071	CE	LYS	A	348	28.394	27.770	42.207	1.00	0.00	C
ATOM	5072	HE1	LYS	A	348	28.154	27.475	41.182	1.00	0.00	H
ATOM	5073	HE2	LYS	A	348	27.982	27.013	42.879	1.00	0.00	H

ATOM	5074	NZ	LYS	A	348	27.751	29.067	42.501	1.00	0.00	N
ATOM	5075	HZ1	LYS	A	348	27.808	29.291	43.492	1.00	0.00	H
ATOM	5076	HZ2	LYS	A	348	28.180	29.819	41.971	1.00	0.00	H
ATOM	5077	HZ3	LYS	A	348	26.771	29.009	42.240	1.00	0.00	H
ATOM	5078	C	LYS	A	348	30.375	22.876	43.281	1.00	0.00	C
ATOM	5079	O	LYS	A	348	30.862	22.927	44.410	1.00	0.00	O
ATOM	5080	N	GLN	A	349	29.533	21.898	42.933	1.00	0.00	N
ATOM	5081	H	GLN	A	349	29.110	21.927	42.016	1.00	0.00	H
ATOM	5082	CA	GLN	A	349	29.294	20.722	43.773	1.00	0.00	C
ATOM	5083	HA	GLN	A	349	29.336	21.017	44.825	1.00	0.00	H
ATOM	5084	CB	GLN	A	349	27.894	20.139	43.489	1.00	0.00	C
ATOM	5085	HB1	GLN	A	349	27.173	20.955	43.496	1.00	0.00	H
ATOM	5086	HB2	GLN	A	349	27.861	19.684	42.499	1.00	0.00	H
ATOM	5087	CG	GLN	A	349	27.435	19.113	44.540	1.00	0.00	C
ATOM	5088	HG1	GLN	A	349	27.711	19.471	45.529	1.00	0.00	H
ATOM	5089	HG2	GLN	A	349	26.347	19.045	44.513	1.00	0.00	H
ATOM	5090	CD	GLN	A	349	27.998	17.709	44.313	1.00	0.00	C
ATOM	5091	OE1	GLN	A	349	27.571	16.983	43.420	1.00	0.00	O
ATOM	5092	NE2	GLN	A	349	28.956	17.267	45.097	1.00	0.00	N
ATOM	5093	HE21	GLN	A	349	29.304	17.859	45.846	1.00	0.00	H
ATOM	5094	HE22	GLN	A	349	29.439	16.409	44.885	1.00	0.00	H
ATOM	5095	C	GLN	A	349	30.414	19.704	43.539	1.00	0.00	C
ATOM	5096	O	GLN	A	349	31.080	19.298	44.486	1.00	0.00	O
ATOM	5097	N	LYS	A	350	30.661	19.325	42.280	1.00	0.00	N
ATOM	5098	H	LYS	A	350	30.082	19.706	41.538	1.00	0.00	H
ATOM	5099	CA	LYS	A	350	31.641	18.290	41.921	1.00	0.00	C
ATOM	5100	HA	LYS	A	350	31.488	17.432	42.577	1.00	0.00	H
ATOM	5101	CB	LYS	A	350	31.401	17.857	40.460	1.00	0.00	C
ATOM	5102	HB1	LYS	A	350	31.546	18.723	39.812	1.00	0.00	H
ATOM	5103	HB2	LYS	A	350	32.150	17.111	40.188	1.00	0.00	H
ATOM	5104	CG	LYS	A	350	30.013	17.267	40.161	1.00	0.00	C
ATOM	5105	HG1	LYS	A	350	29.247	18.023	40.335	1.00	0.00	H
ATOM	5106	HG2	LYS	A	350	29.986	17.000	39.104	1.00	0.00	H
ATOM	5107	CD	LYS	A	350	29.680	16.018	40.988	1.00	0.00	C
ATOM	5108	HD1	LYS	A	350	30.448	15.260	40.829	1.00	0.00	H
ATOM	5109	HD2	LYS	A	350	29.644	16.274	42.046	1.00	0.00	H
ATOM	5110	CE	LYS	A	350	28.321	15.472	40.541	1.00	0.00	C
ATOM	5111	HE1	LYS	A	350	27.585	16.281	40.549	1.00	0.00	H
ATOM	5112	HE2	LYS	A	350	28.411	15.113	39.513	1.00	0.00	H
ATOM	5113	NZ	LYS	A	350	27.845	14.370	41.405	1.00	0.00	N
ATOM	5114	HZ1	LYS	A	350	27.089	13.867	40.944	1.00	0.00	H
ATOM	5115	HZ2	LYS	A	350	28.577	13.701	41.615	1.00	0.00	H
ATOM	5116	HZ3	LYS	A	350	27.487	14.707	42.291	1.00	0.00	H
ATOM	5117	C	LYS	A	350	33.115	18.695	42.133	1.00	0.00	C
ATOM	5118	O	LYS	A	350	33.982	17.821	42.089	1.00	0.00	O
ATOM	5119	N	GLY	A	351	33.412	19.983	42.348	1.00	0.00	N
ATOM	5120	H	GLY	A	351	32.646	20.641	42.360	1.00	0.00	H
ATOM	5121	CA	GLY	A	351	34.764	20.480	42.652	1.00	0.00	C
ATOM	5122	HA1	GLY	A	351	34.677	21.377	43.264	1.00	0.00	H
ATOM	5123	HA2	GLY	A	351	35.314	19.720	43.210	1.00	0.00	H
ATOM	5124	C	GLY	A	351	35.599	20.841	41.420	1.00	0.00	C
ATOM	5125	O	GLY	A	351	36.831	20.823	41.492	1.00	0.00	O
ATOM	5126	N	LEU	A	352	34.950	21.139	40.287	1.00	0.00	N
ATOM	5127	H	LEU	A	352	33.943	21.235	40.330	1.00	0.00	H

ATOM	5128	CA	LEU	A	352	35.621	21.373	39.002	1.00	0.00	C
ATOM	5129	HA	LEU	A	352	36.386	20.605	38.876	1.00	0.00	H
ATOM	5130	CB	LEU	A	352	34.620	21.248	37.837	1.00	0.00	C
ATOM	5131	HB1	LEU	A	352	33.874	22.034	37.942	1.00	0.00	H
ATOM	5132	HB2	LEU	A	352	35.160	21.430	36.907	1.00	0.00	H
ATOM	5133	CG	LEU	A	352	33.894	19.892	37.713	1.00	0.00	C
ATOM	5134	HG	LEU	A	352	33.174	19.806	38.527	1.00	0.00	H
ATOM	5135	CD1	LEU	A	352	33.127	19.842	36.382	1.00	0.00	C
ATOM	5136	HD11	LEU	A	352	32.566	18.912	36.314	1.00	0.00	H
ATOM	5137	HD12	LEU	A	352	32.424	20.669	36.318	1.00	0.00	H
ATOM	5138	HD13	LEU	A	352	33.823	19.898	35.543	1.00	0.00	H
ATOM	5139	CD2	LEU	A	352	34.849	18.692	37.782	1.00	0.00	C
ATOM	5140	HD21	LEU	A	352	34.304	17.775	37.559	1.00	0.00	H
ATOM	5141	HD22	LEU	A	352	35.653	18.817	37.059	1.00	0.00	H
ATOM	5142	HD23	LEU	A	352	35.271	18.601	38.783	1.00	0.00	H
ATOM	5143	C	LEU	A	352	36.360	22.718	38.953	1.00	0.00	C
ATOM	5144	O	LEU	A	352	35.994	23.681	39.637	1.00	0.00	O
ATOM	5145	N	GLY	A	353	37.382	22.798	38.095	1.00	0.00	N
ATOM	5146	H	GLY	A	353	37.604	21.965	37.556	1.00	0.00	H
ATOM	5147	CA	GLY	A	353	38.322	23.923	38.003	1.00	0.00	C
ATOM	5148	HA1	GLY	A	353	38.652	24.174	39.008	1.00	0.00	H
ATOM	5149	HA2	GLY	A	353	39.180	23.616	37.405	1.00	0.00	H
ATOM	5150	C	GLY	A	353	37.753	25.204	37.388	1.00	0.00	C
ATOM	5151	O	GLY	A	353	38.458	26.213	37.350	1.00	0.00	O
ATOM	5152	N	GLY	A	354	36.496	25.190	36.933	1.00	0.00	N
ATOM	5153	H	GLY	A	354	35.997	24.313	36.978	1.00	0.00	H
ATOM	5154	CA	GLY	A	354	35.777	26.350	36.398	1.00	0.00	C
ATOM	5155	HA1	GLY	A	354	35.136	26.760	37.179	1.00	0.00	H
ATOM	5156	HA2	GLY	A	354	36.485	27.121	36.105	1.00	0.00	H
ATOM	5157	C	GLY	A	354	34.889	26.022	35.200	1.00	0.00	C
ATOM	5158	O	GLY	A	354	34.668	24.853	34.877	1.00	0.00	O
ATOM	5159	N	ALA	A	355	34.397	27.073	34.541	1.00	0.00	N
ATOM	5160	H	ALA	A	355	34.688	27.998	34.838	1.00	0.00	H
ATOM	5161	CA	ALA	A	355	33.590	26.975	33.323	1.00	0.00	C
ATOM	5162	HA	ALA	A	355	33.247	25.945	33.200	1.00	0.00	H
ATOM	5163	CB	ALA	A	355	32.346	27.863	33.460	1.00	0.00	C
ATOM	5164	HB1	ALA	A	355	31.712	27.742	32.579	1.00	0.00	H
ATOM	5165	HB2	ALA	A	355	31.774	27.576	34.341	1.00	0.00	H
ATOM	5166	HB3	ALA	A	355	32.639	28.909	33.540	1.00	0.00	H
ATOM	5167	C	ALA	A	355	34.394	27.350	32.067	1.00	0.00	C
ATOM	5168	O	ALA	A	355	35.252	28.238	32.083	1.00	0.00	O
ATOM	5169	N	MET	A	356	34.064	26.691	30.963	1.00	0.00	N
ATOM	5170	H	MET	A	356	33.320	26.000	31.040	1.00	0.00	H
ATOM	5171	CA	MET	A	356	34.473	27.012	29.600	1.00	0.00	C
ATOM	5172	HA	MET	A	356	35.139	27.864	29.630	1.00	0.00	H
ATOM	5173	CB	MET	A	356	35.240	25.808	29.017	1.00	0.00	C
ATOM	5174	HB1	MET	A	356	36.168	25.699	29.578	1.00	0.00	H
ATOM	5175	HB2	MET	A	356	34.663	24.894	29.157	1.00	0.00	H
ATOM	5176	CG	MET	A	356	35.619	25.944	27.540	1.00	0.00	C
ATOM	5177	HG1	MET	A	356	35.913	26.974	27.334	1.00	0.00	H
ATOM	5178	HG2	MET	A	356	36.499	25.328	27.355	1.00	0.00	H
ATOM	5179	SD	MET	A	356	34.322	25.442	26.376	1.00	0.00	S
ATOM	5180	CE	MET	A	356	34.605	23.653	26.349	1.00	0.00	C
ATOM	5181	HE1	MET	A	356	34.598	23.250	27.361	1.00	0.00	H

```
ATOM   5182  HE2 MET A 356      33.816  23.175  25.769  1.00  0.00           H
ATOM   5183  HE3 MET A 356      35.558  23.442  25.866  1.00  0.00           H
ATOM   5184  C   MET A 356      33.219  27.405  28.808  1.00  0.00           C
ATOM   5185  O   MET A 356      32.129  26.947  29.139  1.00  0.00           O
ATOM   5186  N   VAL A 357      33.340  28.258  27.793  1.00  0.00           N
ATOM   5187  H   VAL A 357      34.255  28.644  27.593  1.00  0.00           H
ATOM   5188  CA  VAL A 357      32.199  28.701  26.982  1.00  0.00           C
ATOM   5189  HA  VAL A 357      31.461  27.902  27.023  1.00  0.00           H
ATOM   5190  CB  VAL A 357      31.524  29.954  27.592  1.00  0.00           C
ATOM   5191  HB  VAL A 357      31.225  29.695  28.608  1.00  0.00           H
ATOM   5192  CG1 VAL A 357      32.452  31.175  27.702  1.00  0.00           C
ATOM   5193 HG11 VAL A 357      31.934  31.983  28.220  1.00  0.00           H
ATOM   5194 HG12 VAL A 357      33.340  30.919  28.278  1.00  0.00           H
ATOM   5195 HG13 VAL A 357      32.747  31.527  26.713  1.00  0.00           H
ATOM   5196  CG2 VAL A 357      30.251  30.347  26.834  1.00  0.00           C
ATOM   5197 HG21 VAL A 357      29.729  31.133  27.376  1.00  0.00           H
ATOM   5198 HG22 VAL A 357      30.491  30.703  25.831  1.00  0.00           H
ATOM   5199 HG23 VAL A 357      29.584  29.491  26.765  1.00  0.00           H
ATOM   5200  C   VAL A 357      32.565  28.875  25.506  1.00  0.00           C
ATOM   5201  O   VAL A 357      33.550  29.539  25.171  1.00  0.00           O
ATOM   5202  N   TRP A 358      31.739  28.273  24.644  1.00  0.00           N
ATOM   5203  H   TRP A 358      30.958  27.754  25.038  1.00  0.00           H
ATOM   5204  CA  TRP A 358      31.757  28.404  23.187  1.00  0.00           C
ATOM   5205  HA  TRP A 358      32.602  29.015  22.880  1.00  0.00           H
ATOM   5206  CB  TRP A 358      31.954  27.019  22.552  1.00  0.00           C
ATOM   5207  HB1 TRP A 358      32.839  26.561  22.995  1.00  0.00           H
ATOM   5208  HB2 TRP A 358      31.103  26.381  22.796  1.00  0.00           H
ATOM   5209  CG  TRP A 358      32.142  27.035  21.063  1.00  0.00           C
ATOM   5210  CD1 TRP A 358      31.155  27.036  20.139  1.00  0.00           C
ATOM   5211  HD1 TRP A 358      30.096  27.019  20.359  1.00  0.00           H
ATOM   5212  NE1 TRP A 358      31.702  27.070  18.873  1.00  0.00           N
ATOM   5213  HE1 TRP A 358      31.151  27.051  18.021  1.00  0.00           H
ATOM   5214  CE2 TRP A 358      33.076  27.126  18.911  1.00  0.00           C
ATOM   5215  CZ2 TRP A 358      34.060  27.209  17.916  1.00  0.00           C
ATOM   5216  HZ2 TRP A 358      33.777  27.243  16.873  1.00  0.00           H
ATOM   5217  CH2 TRP A 358      35.412  27.238  18.294  1.00  0.00           C
ATOM   5218  HH2 TRP A 358      36.185  27.304  17.543  1.00  0.00           H
ATOM   5219  CZ3 TRP A 358      35.757  27.175  19.654  1.00  0.00           C
ATOM   5220  HZ3 TRP A 358      36.799  27.183  19.942  1.00  0.00           H
ATOM   5221  CE3 TRP A 358      34.756  27.102  20.642  1.00  0.00           C
ATOM   5222  HE3 TRP A 358      35.028  27.053  21.684  1.00  0.00           H
ATOM   5223  CD2 TRP A 358      33.389  27.082  20.301  1.00  0.00           C
ATOM   5224  C   TRP A 358      30.432  29.052  22.733  1.00  0.00           C
ATOM   5225  O   TRP A 358      29.374  28.436  22.846  1.00  0.00           O
ATOM   5226  N   ALA A 359      30.413  30.295  22.249  1.00  0.00           N
ATOM   5227  H   ALA A 359      29.479  30.658  22.095  1.00  0.00           H
ATOM   5228  CA  ALA A 359      31.520  31.239  22.058  1.00  0.00           C
ATOM   5229  HA  ALA A 359      32.311  31.046  22.784  1.00  0.00           H
ATOM   5230  CB  ALA A 359      32.087  31.040  20.642  1.00  0.00           C
ATOM   5231  HB1 ALA A 359      32.939  31.701  20.478  1.00  0.00           H
ATOM   5232  HB2 ALA A 359      32.417  30.009  20.509  1.00  0.00           H
ATOM   5233  HB3 ALA A 359      31.322  31.269  19.897  1.00  0.00           H
ATOM   5234  C   ALA A 359      31.067  32.692  22.297  1.00  0.00           C
ATOM   5235  O   ALA A 359      29.869  32.978  22.392  1.00  0.00           O
```

ATOM	5236	N	LEU	A	360	32.037	33.612	22.385	1.00	0.00	N
ATOM	5237	H	LEU	A	360	32.997	33.306	22.262	1.00	0.00	H
ATOM	5238	CA	LEU	A	360	31.806	35.043	22.647	1.00	0.00	C
ATOM	5239	HA	LEU	A	360	31.251	35.151	23.579	1.00	0.00	H
ATOM	5240	CB	LEU	A	360	33.175	35.744	22.770	1.00	0.00	C
ATOM	5241	HB1	LEU	A	360	33.783	35.459	21.911	1.00	0.00	H
ATOM	5242	HB2	LEU	A	360	33.028	36.825	22.720	1.00	0.00	H
ATOM	5243	CG	LEU	A	360	33.939	35.413	24.068	1.00	0.00	C
ATOM	5244	HG	LEU	A	360	33.866	34.344	24.273	1.00	0.00	H
ATOM	5245	CD1	LEU	A	360	35.422	35.766	23.910	1.00	0.00	C
ATOM	5246	HD11	LEU	A	360	35.960	35.525	24.827	1.00	0.00	H
ATOM	5247	HD12	LEU	A	360	35.853	35.188	23.095	1.00	0.00	H
ATOM	5248	HD13	LEU	A	360	35.533	36.828	23.694	1.00	0.00	H
ATOM	5249	CD2	LEU	A	360	33.370	36.189	25.266	1.00	0.00	C
ATOM	5250	HD21	LEU	A	360	33.895	35.905	26.178	1.00	0.00	H
ATOM	5251	HD22	LEU	A	360	33.486	37.261	25.106	1.00	0.00	H
ATOM	5252	HD23	LEU	A	360	32.313	35.969	25.389	1.00	0.00	H
ATOM	5253	C	LEU	A	360	30.945	35.723	21.571	1.00	0.00	C
ATOM	5254	O	LEU	A	360	30.237	36.675	21.876	1.00	0.00	O
ATOM	5255	N	ASP	A	361	30.979	35.221	20.336	1.00	0.00	N
ATOM	5256	H	ASP	A	361	31.608	34.448	20.162	1.00	0.00	H
ATOM	5257	CA	ASP	A	361	30.189	35.712	19.201	1.00	0.00	C
ATOM	5258	HA	ASP	A	361	30.051	36.787	19.320	1.00	0.00	H
ATOM	5259	CB	ASP	A	361	30.982	35.480	17.904	1.00	0.00	C
ATOM	5260	HB1	ASP	A	361	30.393	35.818	17.051	1.00	0.00	H
ATOM	5261	HB2	ASP	A	361	31.879	36.100	17.929	1.00	0.00	H
ATOM	5262	CG	ASP	A	361	31.389	34.018	17.698	1.00	0.00	C
ATOM	5263	OD1	ASP	A	361	30.720	33.300	16.924	1.00	0.00	O
ATOM	5264	OD2	ASP	A	361	32.421	33.621	18.291	1.00	0.00	O
ATOM	5265	C	ASP	A	361	28.773	35.114	19.089	1.00	0.00	C
ATOM	5266	O	ASP	A	361	28.077	35.450	18.131	1.00	0.00	O
ATOM	5267	N	LEU	A	362	28.352	34.237	20.013	1.00	0.00	N
ATOM	5268	H	LEU	A	362	28.986	34.009	20.768	1.00	0.00	H
ATOM	5269	CA	LEU	A	362	27.040	33.562	19.984	1.00	0.00	C
ATOM	5270	HA	LEU	A	362	26.498	33.858	19.088	1.00	0.00	H
ATOM	5271	CB	LEU	A	362	27.261	32.036	19.920	1.00	0.00	C
ATOM	5272	HB1	LEU	A	362	27.782	31.733	20.823	1.00	0.00	H
ATOM	5273	HB2	LEU	A	362	26.285	31.547	19.920	1.00	0.00	H
ATOM	5274	CG	LEU	A	362	28.069	31.523	18.708	1.00	0.00	C
ATOM	5275	HG	LEU	A	362	29.045	32.008	18.700	1.00	0.00	H
ATOM	5276	CD1	LEU	A	362	28.304	30.011	18.851	1.00	0.00	C
ATOM	5277	HD11	LEU	A	362	28.919	29.654	18.025	1.00	0.00	H
ATOM	5278	HD12	LEU	A	362	28.821	29.800	19.787	1.00	0.00	H
ATOM	5279	HD13	LEU	A	362	27.352	29.480	18.840	1.00	0.00	H
ATOM	5280	CD2	LEU	A	362	27.373	31.799	17.367	1.00	0.00	C
ATOM	5281	HD21	LEU	A	362	27.979	31.405	16.550	1.00	0.00	H
ATOM	5282	HD22	LEU	A	362	26.395	31.317	17.348	1.00	0.00	H
ATOM	5283	HD23	LEU	A	362	27.252	32.871	17.216	1.00	0.00	H
ATOM	5284	C	LEU	A	362	26.108	33.956	21.152	1.00	0.00	C
ATOM	5285	O	LEU	A	362	24.890	33.876	20.998	1.00	0.00	O
ATOM	5286	N	ASP	A	363	26.656	34.442	22.275	1.00	0.00	N
ATOM	5287	H	ASP	A	363	27.655	34.559	22.283	1.00	0.00	H
ATOM	5288	CA	ASP	A	363	25.888	35.167	23.307	1.00	0.00	C
ATOM	5289	HA	ASP	A	363	25.011	34.572	23.572	1.00	0.00	H

ATOM	5290	CB	ASP	A	363	26.768	35.341	24.569	1.00	0.00	C
ATOM	5291	HB1	ASP	A	363	27.275	34.397	24.766	1.00	0.00	H
ATOM	5292	HB2	ASP	A	363	27.539	36.084	24.357	1.00	0.00	H
ATOM	5293	CG	ASP	A	363	26.029	35.742	25.861	1.00	0.00	C
ATOM	5294	OD1	ASP	A	363	24.780	35.777	25.884	1.00	0.00	O
ATOM	5295	OD2	ASP	A	363	26.713	36.007	26.880	1.00	0.00	O
ATOM	5296	C	ASP	A	363	25.404	36.524	22.745	1.00	0.00	C
ATOM	5297	O	ASP	A	363	25.924	36.998	21.734	1.00	0.00	O
ATOM	5298	N	ASP	A	364	24.444	37.190	23.396	1.00	0.00	N
ATOM	5299	H	ASP	A	364	24.108	36.790	24.269	1.00	0.00	H
ATOM	5300	CA	ASP	A	364	23.999	38.538	22.991	1.00	0.00	C
ATOM	5301	HA	ASP	A	364	24.013	38.572	21.900	1.00	0.00	H
ATOM	5302	CB	ASP	A	364	22.538	38.798	23.406	1.00	0.00	C
ATOM	5303	HB1	ASP	A	364	21.939	37.943	23.094	1.00	0.00	H
ATOM	5304	HB2	ASP	A	364	22.476	38.873	24.491	1.00	0.00	H
ATOM	5305	CG	ASP	A	364	21.926	40.063	22.776	1.00	0.00	C
ATOM	5306	OD1	ASP	A	364	22.557	40.696	21.901	1.00	0.00	O
ATOM	5307	OD2	ASP	A	364	20.789	40.429	23.165	1.00	0.00	O
ATOM	5308	C	ASP	A	364	24.997	39.609	23.475	1.00	0.00	C
ATOM	5309	O	ASP	A	364	24.703	40.445	24.333	1.00	0.00	O
ATOM	5310	N	PHE	A	365	26.217	39.555	22.931	1.00	0.00	N
ATOM	5311	H	PHE	A	365	26.381	38.797	22.275	1.00	0.00	H
ATOM	5312	CA	PHE	A	365	27.360	40.404	23.275	1.00	0.00	C
ATOM	5313	HA	PHE	A	365	27.608	40.238	24.323	1.00	0.00	H
ATOM	5314	CB	PHE	A	365	28.573	39.970	22.433	1.00	0.00	C
ATOM	5315	HB1	PHE	A	365	29.453	40.498	22.802	1.00	0.00	H
ATOM	5316	HB2	PHE	A	365	28.750	38.904	22.589	1.00	0.00	H
ATOM	5317	CG	PHE	A	365	28.450	40.236	20.942	1.00	0.00	C
ATOM	5318	CD1	PHE	A	365	28.831	41.487	20.415	1.00	0.00	C
ATOM	5319	HD1	PHE	A	365	29.220	42.254	21.069	1.00	0.00	H
ATOM	5320	CE1	PHE	A	365	28.681	41.752	19.043	1.00	0.00	C
ATOM	5321	HE1	PHE	A	365	28.957	42.717	18.645	1.00	0.00	H
ATOM	5322	CZ	PHE	A	365	28.147	40.768	18.195	1.00	0.00	C
ATOM	5323	HZ	PHE	A	365	28.004	40.983	17.148	1.00	0.00	H
ATOM	5324	CE2	PHE	A	365	27.786	39.511	18.710	1.00	0.00	C
ATOM	5325	HE2	PHE	A	365	27.379	38.751	18.058	1.00	0.00	H
ATOM	5326	CD2	PHE	A	365	27.943	39.244	20.081	1.00	0.00	C
ATOM	5327	HD2	PHE	A	365	27.672	38.274	20.471	1.00	0.00	H
ATOM	5328	C	PHE	A	365	27.081	41.905	23.104	1.00	0.00	C
ATOM	5329	O	PHE	A	365	27.729	42.716	23.758	1.00	0.00	O
ATOM	5330	N	ALA	A	366	26.117	42.278	22.253	1.00	0.00	N
ATOM	5331	H	ALA	A	366	25.644	41.540	21.750	1.00	0.00	H
ATOM	5332	CA	ALA	A	366	25.709	43.661	21.993	1.00	0.00	C
ATOM	5333	HA	ALA	A	366	26.476	44.337	22.367	1.00	0.00	H
ATOM	5334	CB	ALA	A	366	25.627	43.832	20.473	1.00	0.00	C
ATOM	5335	HB1	ALA	A	366	25.429	44.875	20.234	1.00	0.00	H
ATOM	5336	HB2	ALA	A	366	26.577	43.545	20.020	1.00	0.00	H
ATOM	5337	HB3	ALA	A	366	24.834	43.209	20.061	1.00	0.00	H
ATOM	5338	C	ALA	A	366	24.391	44.070	22.685	1.00	0.00	C
ATOM	5339	O	ALA	A	366	23.988	45.237	22.603	1.00	0.00	O
ATOM	5340	N	GLY	A	367	23.704	43.128	23.343	1.00	0.00	N
ATOM	5341	H	GLY	A	367	24.076	42.184	23.355	1.00	0.00	H
ATOM	5342	CA	GLY	A	367	22.415	43.338	24.011	1.00	0.00	C
ATOM	5343	HA1	GLY	A	367	22.151	42.420	24.526	1.00	0.00	H

ATOM	5344	HA2	GLY	A	367	22.503	44.147	24.735	1.00	0.00	H
ATOM	5345	C	GLY	A	367	21.269	43.676	23.056	1.00	0.00	C
ATOM	5346	O	GLY	A	367	20.296	44.308	23.468	1.00	0.00	O
ATOM	5347	N	PHE	A	368	21.393	43.329	21.772	1.00	0.00	N
ATOM	5348	H	PHE	A	368	22.131	42.674	21.532	1.00	0.00	H
ATOM	5349	CA	PHE	A	368	20.514	43.824	20.714	1.00	0.00	C
ATOM	5350	HA	PHE	A	368	20.236	44.851	20.960	1.00	0.00	H
ATOM	5351	CB	PHE	A	368	21.281	43.872	19.382	1.00	0.00	C
ATOM	5352	HB1	PHE	A	368	20.804	44.630	18.760	1.00	0.00	H
ATOM	5353	HB2	PHE	A	368	22.300	44.220	19.559	1.00	0.00	H
ATOM	5354	CG	PHE	A	368	21.328	42.580	18.579	1.00	0.00	C
ATOM	5355	CD1	PHE	A	368	22.342	41.628	18.798	1.00	0.00	C
ATOM	5356	HD1	PHE	A	368	23.072	41.780	19.578	1.00	0.00	H
ATOM	5357	CE1	PHE	A	368	22.422	40.481	17.989	1.00	0.00	C
ATOM	5358	HE1	PHE	A	368	23.213	39.762	18.153	1.00	0.00	H
ATOM	5359	CZ	PHE	A	368	21.485	40.275	16.962	1.00	0.00	C
ATOM	5360	HZ	PHE	A	368	21.559	39.408	16.323	1.00	0.00	H
ATOM	5361	CE2	PHE	A	368	20.454	41.206	16.757	1.00	0.00	C
ATOM	5362	HE2	PHE	A	368	19.731	41.050	15.968	1.00	0.00	H
ATOM	5363	CD2	PHE	A	368	20.380	42.357	17.560	1.00	0.00	C
ATOM	5364	HD2	PHE	A	368	19.607	43.085	17.372	1.00	0.00	H
ATOM	5365	C	PHE	A	368	19.214	43.023	20.587	1.00	0.00	C
ATOM	5366	O	PHE	A	368	18.188	43.586	20.207	1.00	0.00	O
ATOM	5367	N	SER	A	369	19.221	41.730	20.909	1.00	0.00	N
ATOM	5368	H	SER	A	369	20.071	41.337	21.314	1.00	0.00	H
ATOM	5369	CA	SER	A	369	18.097	40.835	20.597	1.00	0.00	C
ATOM	5370	HA	SER	A	369	17.427	41.300	19.875	1.00	0.00	H
ATOM	5371	CB	SER	A	369	18.644	39.566	19.946	1.00	0.00	C
ATOM	5372	HB1	SER	A	369	19.278	39.842	19.102	1.00	0.00	H
ATOM	5373	HB2	SER	A	369	19.243	39.016	20.673	1.00	0.00	H
ATOM	5374	OG	SER	A	369	17.587	38.747	19.480	1.00	0.00	O
ATOM	5375	HG	SER	A	369	18.003	37.948	19.095	1.00	0.00	H
ATOM	5376	C	SER	A	369	17.253	40.459	21.818	1.00	0.00	C
ATOM	5377	O	SER	A	369	16.020	40.440	21.732	1.00	0.00	O
ATOM	5378	N	CYS	A	370	17.904	40.154	22.944	1.00	0.00	N
ATOM	5379	H	CYS	A	370	18.923	40.210	22.945	1.00	0.00	H
ATOM	5380	CA	CYS	A	370	17.273	39.494	24.093	1.00	0.00	C
ATOM	5381	HA	CYS	A	370	16.395	38.949	23.746	1.00	0.00	H
ATOM	5382	CB	CYS	A	370	18.263	38.478	24.680	1.00	0.00	C
ATOM	5383	HB1	CYS	A	370	19.030	39.006	25.243	1.00	0.00	H
ATOM	5384	HB2	CYS	A	370	17.727	37.832	25.378	1.00	0.00	H
ATOM	5385	SG	CYS	A	370	19.095	37.439	23.452	1.00	0.00	S
ATOM	5386	C	CYS	A	370	16.795	40.459	25.181	1.00	0.00	C
ATOM	5387	O	CYS	A	370	16.063	40.043	26.078	1.00	0.00	O
ATOM	5388	N	ASN	A	371	17.214	41.727	25.122	1.00	0.00	N
ATOM	5389	H	ASN	A	371	17.784	41.991	24.331	1.00	0.00	H
ATOM	5390	CA	ASN	A	371	16.825	42.798	26.047	1.00	0.00	C
ATOM	5391	HA	ASN	A	371	17.356	43.687	25.708	1.00	0.00	H
ATOM	5392	CB	ASN	A	371	15.311	43.081	25.895	1.00	0.00	C
ATOM	5393	HB1	ASN	A	371	14.968	42.732	24.920	1.00	0.00	H
ATOM	5394	HB2	ASN	A	371	14.747	42.540	26.655	1.00	0.00	H
ATOM	5395	CG	ASN	A	371	14.994	44.570	25.975	1.00	0.00	C
ATOM	5396	OD1	ASN	A	371	14.612	45.195	24.988	1.00	0.00	O
ATOM	5397	ND2	ASN	A	371	15.178	45.186	27.126	1.00	0.00	N

```
ATOM   5398 HD21 ASN A 371      15.062  46.188  27.162  1.00  0.00           H
ATOM   5399 HD22 ASN A 371      15.493  44.665  27.932  1.00  0.00           H
ATOM   5400  C   ASN A 371      17.306  42.543  27.502  1.00  0.00           C
ATOM   5401  O   ASN A 371      16.670  42.970  28.469  1.00  0.00           O
ATOM   5402  N   GLN A 372      18.411  41.805  27.667  1.00  0.00           N
ATOM   5403  H   GLN A 372      18.902  41.516  26.825  1.00  0.00           H
ATOM   5404  CA  GLN A 372      19.032  41.421  28.945  1.00  0.00           C
ATOM   5405  HA  GLN A 372      18.418  41.772  29.776  1.00  0.00           H
ATOM   5406  CB  GLN A 372      19.136  39.877  29.016  1.00  0.00           C
ATOM   5407  HB1 GLN A 372      19.651  39.519  28.123  1.00  0.00           H
ATOM   5408  HB2 GLN A 372      19.730  39.599  29.887  1.00  0.00           H
ATOM   5409  CG  GLN A 372      17.799  39.130  29.146  1.00  0.00           C
ATOM   5410  HG1 GLN A 372      17.310  39.428  30.075  1.00  0.00           H
ATOM   5411  HG2 GLN A 372      17.146  39.398  28.318  1.00  0.00           H
ATOM   5412  CD  GLN A 372      17.989  37.608  29.141  1.00  0.00           C
ATOM   5413  OE1 GLN A 372      17.928  36.951  28.108  1.00  0.00           O
ATOM   5414  NE2 GLN A 372      18.240  36.971  30.267  1.00  0.00           N
ATOM   5415 HE21 GLN A 372      18.339  37.461  31.151  1.00  0.00           H
ATOM   5416 HE22 GLN A 372      18.295  35.963  30.237  1.00  0.00           H
ATOM   5417  C   GLN A 372      20.426  42.072  29.128  1.00  0.00           C
ATOM   5418  O   GLN A 372      21.225  41.596  29.937  1.00  0.00           O
ATOM   5419  N   GLY A 373      20.762  43.126  28.371  1.00  0.00           N
ATOM   5420  H   GLY A 373      20.067  43.517  27.741  1.00  0.00           H
ATOM   5421  CA  GLY A 373      22.091  43.758  28.363  1.00  0.00           C
ATOM   5422  HA1 GLY A 373      22.009  44.760  27.941  1.00  0.00           H
ATOM   5423  HA2 GLY A 373      22.471  43.831  29.383  1.00  0.00           H
ATOM   5424  C   GLY A 373      23.117  42.979  27.531  1.00  0.00           C
ATOM   5425  O   GLY A 373      22.788  41.956  26.927  1.00  0.00           O
ATOM   5426  N   ARG A 374      24.368  43.457  27.507  1.00  0.00           N
ATOM   5427  H   ARG A 374      24.572  44.289  28.042  1.00  0.00           H
ATOM   5428  CA  ARG A 374      25.490  42.769  26.842  1.00  0.00           C
ATOM   5429  HA  ARG A 374      25.185  42.510  25.828  1.00  0.00           H
ATOM   5430  CB  ARG A 374      26.731  43.678  26.755  1.00  0.00           C
ATOM   5431  HB1 ARG A 374      26.973  44.074  27.743  1.00  0.00           H
ATOM   5432  HB2 ARG A 374      27.571  43.062  26.428  1.00  0.00           H
ATOM   5433  CG  ARG A 374      26.570  44.840  25.761  1.00  0.00           C
ATOM   5434  HG1 ARG A 374      26.111  44.463  24.849  1.00  0.00           H
ATOM   5435  HG2 ARG A 374      25.916  45.602  26.185  1.00  0.00           H
ATOM   5436  CD  ARG A 374      27.935  45.457  25.410  1.00  0.00           C
ATOM   5437  HD1 ARG A 374      28.317  45.989  26.284  1.00  0.00           H
ATOM   5438  HD2 ARG A 374      28.638  44.661  25.175  1.00  0.00           H
ATOM   5439  NE  ARG A 374      27.830  46.398  24.279  1.00  0.00           N
ATOM   5440  HE  ARG A 374      27.362  47.270  24.495  1.00  0.00           H
ATOM   5441  CZ  ARG A 374      28.308  46.241  23.045  1.00  0.00           C
ATOM   5442  NH1 ARG A 374      28.072  47.158  22.136  1.00  0.00           N
ATOM   5443 HH11 ARG A 374      27.554  47.995  22.374  1.00  0.00           H
ATOM   5444 HH12 ARG A 374      28.363  47.000  21.178  1.00  0.00           H
ATOM   5445  NH2 ARG A 374      29.007  45.191  22.670  1.00  0.00           N
ATOM   5446 HH21 ARG A 374      29.232  44.457  23.338  1.00  0.00           H
ATOM   5447 HH22 ARG A 374      29.436  45.139  21.752  1.00  0.00           H
ATOM   5448  C   ARG A 374      25.863  41.469  27.570  1.00  0.00           C
ATOM   5449  O   ARG A 374      25.855  41.427  28.801  1.00  0.00           O
ATOM   5450  N   TYR A 375      26.222  40.422  26.826  1.00  0.00           N
ATOM   5451  H   TYR A 375      26.081  40.505  25.826  1.00  0.00           H
```

ATOM	5452	CA	TYR	A	375	26.717	39.128	27.336	1.00	0.00	C
ATOM	5453	HA	TYR	A	375	26.697	38.426	26.503	1.00	0.00	H
ATOM	5454	CB	TYR	A	375	28.186	39.269	27.791	1.00	0.00	C
ATOM	5455	HB1	TYR	A	375	28.258	40.074	28.521	1.00	0.00	H
ATOM	5456	HB2	TYR	A	375	28.482	38.353	28.301	1.00	0.00	H
ATOM	5457	CG	TYR	A	375	29.218	39.482	26.706	1.00	0.00	C
ATOM	5458	CD1	TYR	A	375	29.537	38.427	25.828	1.00	0.00	C
ATOM	5459	HD1	TYR	A	375	28.982	37.500	25.873	1.00	0.00	H
ATOM	5460	CE1	TYR	A	375	30.591	38.570	24.906	1.00	0.00	C
ATOM	5461	HE1	TYR	A	375	30.829	37.767	24.223	1.00	0.00	H
ATOM	5462	CZ	TYR	A	375	31.327	39.774	24.858	1.00	0.00	C
ATOM	5463	OH	TYR	A	375	32.361	39.909	23.984	1.00	0.00	O
ATOM	5464	HH	TYR	A	375	32.631	40.835	23.876	1.00	0.00	H
ATOM	5465	CE2	TYR	A	375	31.001	40.831	25.734	1.00	0.00	C
ATOM	5466	HE2	TYR	A	375	31.572	41.748	25.718	1.00	0.00	H
ATOM	5467	CD2	TYR	A	375	29.946	40.685	26.653	1.00	0.00	C
ATOM	5468	HD2	TYR	A	375	29.720	41.489	27.338	1.00	0.00	H
ATOM	5469	C	TYR	A	375	25.841	38.496	28.457	1.00	0.00	C
ATOM	5470	O	TYR	A	375	26.364	38.199	29.540	1.00	0.00	O
ATOM	5471	N	PRO	A	376	24.516	38.322	28.270	1.00	0.00	N
ATOM	5472	CD	PRO	A	376	23.763	38.556	27.047	1.00	0.00	C
ATOM	5473	HD1	PRO	A	376	24.274	38.164	26.170	1.00	0.00	H
ATOM	5474	HD2	PRO	A	376	23.594	39.624	26.923	1.00	0.00	H
ATOM	5475	CG	PRO	A	376	22.423	37.854	27.254	1.00	0.00	C
ATOM	5476	HG1	PRO	A	376	22.513	36.798	26.998	1.00	0.00	H
ATOM	5477	HG2	PRO	A	376	21.623	38.325	26.682	1.00	0.00	H
ATOM	5478	CB	PRO	A	376	22.208	38.007	28.758	1.00	0.00	C
ATOM	5479	HB1	PRO	A	376	21.529	37.250	29.153	1.00	0.00	H
ATOM	5480	HB2	PRO	A	376	21.827	39.007	28.962	1.00	0.00	H
ATOM	5481	CA	PRO	A	376	23.617	37.901	29.345	1.00	0.00	C
ATOM	5482	HA	PRO	A	376	23.703	38.600	30.177	1.00	0.00	H
ATOM	5483	C	PRO	A	376	23.913	36.497	29.887	1.00	0.00	C
ATOM	5484	O	PRO	A	376	23.683	36.258	31.077	1.00	0.00	O
ATOM	5485	N	LEU	A	377	24.456	35.589	29.064	1.00	0.00	N
ATOM	5486	H	LEU	A	377	24.610	35.824	28.082	1.00	0.00	H
ATOM	5487	CA	LEU	A	377	24.893	34.267	29.514	1.00	0.00	C
ATOM	5488	HA	LEU	A	377	24.224	33.908	30.295	1.00	0.00	H
ATOM	5489	CB	LEU	A	377	24.842	33.285	28.330	1.00	0.00	C
ATOM	5490	HB1	LEU	A	377	25.510	33.638	27.546	1.00	0.00	H
ATOM	5491	HB2	LEU	A	377	25.227	32.332	28.676	1.00	0.00	H
ATOM	5492	CG	LEU	A	377	23.449	33.034	27.716	1.00	0.00	C
ATOM	5493	HG	LEU	A	377	23.057	33.965	27.305	1.00	0.00	H
ATOM	5494	CD1	LEU	A	377	23.587	32.017	26.573	1.00	0.00	C
ATOM	5495	HD11	LEU	A	377	22.605	31.742	26.196	1.00	0.00	H
ATOM	5496	HD12	LEU	A	377	24.163	32.457	25.759	1.00	0.00	H
ATOM	5497	HD13	LEU	A	377	24.081	31.113	26.929	1.00	0.00	H
ATOM	5498	CD2	LEU	A	377	22.447	32.497	28.749	1.00	0.00	C
ATOM	5499	HD21	LEU	A	377	21.492	32.313	28.262	1.00	0.00	H
ATOM	5500	HD22	LEU	A	377	22.815	31.568	29.186	1.00	0.00	H
ATOM	5501	HD23	LEU	A	377	22.287	33.231	29.536	1.00	0.00	H
ATOM	5502	C	LEU	A	377	26.295	34.310	30.138	1.00	0.00	C
ATOM	5503	O	LEU	A	377	26.515	33.724	31.198	1.00	0.00	O
ATOM	5504	N	ILE	A	378	27.256	35.011	29.532	1.00	0.00	N
ATOM	5505	H	ILE	A	378	27.034	35.447	28.631	1.00	0.00	H

ATOM	5506	CA	ILE	A	378	28.653	34.993	30.013	1.00	0.00	C
ATOM	5507	HA	ILE	A	378	28.871	33.984	30.364	1.00	0.00	H
ATOM	5508	CB	ILE	A	378	29.637	35.260	28.845	1.00	0.00	C
ATOM	5509	HB	ILE	A	378	29.419	36.233	28.405	1.00	0.00	H
ATOM	5510	CG2	ILE	A	378	31.095	35.246	29.346	1.00	0.00	C
ATOM	5511	HG21	ILE	A	378	31.780	35.499	28.538	1.00	0.00	H
ATOM	5512	HG22	ILE	A	378	31.234	35.984	30.133	1.00	0.00	H
ATOM	5513	HG23	ILE	A	378	31.350	34.261	29.739	1.00	0.00	H
ATOM	5514	CG1	ILE	A	378	29.456	34.162	27.764	1.00	0.00	C
ATOM	5515	HG11	ILE	A	378	29.690	33.196	28.209	1.00	0.00	H
ATOM	5516	HG12	ILE	A	378	28.418	34.126	27.438	1.00	0.00	H
ATOM	5517	CD	ILE	A	378	30.307	34.337	26.501	1.00	0.00	C
ATOM	5518	HD1	ILE	A	378	30.024	33.582	25.767	1.00	0.00	H
ATOM	5519	HD2	ILE	A	378	30.131	35.324	26.074	1.00	0.00	H
ATOM	5520	HD3	ILE	A	378	31.364	34.209	26.731	1.00	0.00	H
ATOM	5521	C	ILE	A	378	28.853	35.872	31.265	1.00	0.00	C
ATOM	5522	O	ILE	A	378	29.609	35.481	32.155	1.00	0.00	O
ATOM	5523	N	GLN	A	379	28.108	36.972	31.437	1.00	0.00	N
ATOM	5524	H	GLN	A	379	27.484	37.270	30.692	1.00	0.00	H
ATOM	5525	CA	GLN	A	379	28.060	37.678	32.729	1.00	0.00	C
ATOM	5526	HA	GLN	A	379	29.080	37.913	33.037	1.00	0.00	H
ATOM	5527	CB	GLN	A	379	27.262	38.995	32.646	1.00	0.00	C
ATOM	5528	HB1	GLN	A	379	26.313	38.833	32.133	1.00	0.00	H
ATOM	5529	HB2	GLN	A	379	27.033	39.307	33.666	1.00	0.00	H
ATOM	5530	CG	GLN	A	379	28.033	40.144	31.974	1.00	0.00	C
ATOM	5531	HG1	GLN	A	379	29.061	40.152	32.334	1.00	0.00	H
ATOM	5532	HG2	GLN	A	379	28.051	39.988	30.896	1.00	0.00	H
ATOM	5533	CD	GLN	A	379	27.419	41.510	32.298	1.00	0.00	C
ATOM	5534	OE1	GLN	A	379	27.448	41.975	33.431	1.00	0.00	O
ATOM	5535	NE2	GLN	A	379	26.838	42.208	31.350	1.00	0.00	N
ATOM	5536	HE21	GLN	A	379	26.716	41.825	30.421	1.00	0.00	H
ATOM	5537	HE22	GLN	A	379	26.408	43.080	31.605	1.00	0.00	H
ATOM	5538	C	GLN	A	379	27.475	36.790	33.841	1.00	0.00	C
ATOM	5539	O	GLN	A	379	27.936	36.876	34.982	1.00	0.00	O
ATOM	5540	N	THR	A	380	26.516	35.909	33.519	1.00	0.00	N
ATOM	5541	H	THR	A	380	26.189	35.882	32.562	1.00	0.00	H
ATOM	5542	CA	THR	A	380	25.953	34.940	34.472	1.00	0.00	C
ATOM	5543	HA	THR	A	380	25.661	35.478	35.373	1.00	0.00	H
ATOM	5544	CB	THR	A	380	24.697	34.281	33.886	1.00	0.00	C
ATOM	5545	HB	THR	A	380	24.880	33.928	32.874	1.00	0.00	H
ATOM	5546	CG2	THR	A	380	24.175	33.105	34.706	1.00	0.00	C
ATOM	5547	HG21	THR	A	380	23.219	32.774	34.303	1.00	0.00	H
ATOM	5548	HG22	THR	A	380	24.869	32.273	34.629	1.00	0.00	H
ATOM	5549	HG23	THR	A	380	24.063	33.382	35.753	1.00	0.00	H
ATOM	5550	OG1	THR	A	380	23.692	35.265	33.856	1.00	0.00	O
ATOM	5551	HG1	THR	A	380	23.619	35.586	32.943	1.00	0.00	H
ATOM	5552	C	THR	A	380	27.006	33.934	34.924	1.00	0.00	C
ATOM	5553	O	THR	A	380	27.129	33.712	36.128	1.00	0.00	O
ATOM	5554	N	LEU	A	381	27.826	33.395	34.009	1.00	0.00	N
ATOM	5555	H	LEU	A	381	27.675	33.631	33.034	1.00	0.00	H
ATOM	5556	CA	LEU	A	381	28.983	32.561	34.374	1.00	0.00	C
ATOM	5557	HA	LEU	A	381	28.632	31.692	34.932	1.00	0.00	H
ATOM	5558	CB	LEU	A	381	29.754	32.088	33.121	1.00	0.00	C
ATOM	5559	HB1	LEU	A	381	30.121	32.962	32.583	1.00	0.00	H

ATOM	5560	HB2	LEU	A	381	30.627	31.527	33.460	1.00	0.00	H
ATOM	5561	CG	LEU	A	381	28.972	31.201	32.132	1.00	0.00	C
ATOM	5562	HG	LEU	A	381	28.123	31.766	31.756	1.00	0.00	H
ATOM	5563	CD1	LEU	A	381	29.868	30.842	30.938	1.00	0.00	C
ATOM	5564	HD11	LEU	A	381	29.304	30.249	30.218	1.00	0.00	H
ATOM	5565	HD12	LEU	A	381	30.213	31.752	30.446	1.00	0.00	H
ATOM	5566	HD13	LEU	A	381	30.729	30.264	31.273	1.00	0.00	H
ATOM	5567	CD2	LEU	A	381	28.458	29.911	32.786	1.00	0.00	C
ATOM	5568	HD21	LEU	A	381	27.957	29.292	32.042	1.00	0.00	H
ATOM	5569	HD22	LEU	A	381	29.289	29.350	33.217	1.00	0.00	H
ATOM	5570	HD23	LEU	A	381	27.737	30.148	33.563	1.00	0.00	H
ATOM	5571	C	LEU	A	381	29.926	33.309	35.327	1.00	0.00	C
ATOM	5572	O	LEU	A	381	30.263	32.777	36.385	1.00	0.00	O
ATOM	5573	N	ARG	A	382	30.306	34.551	34.994	1.00	0.00	N
ATOM	5574	H	ARG	A	382	30.004	34.924	34.099	1.00	0.00	H
ATOM	5575	CA	ARG	A	382	31.183	35.368	35.843	1.00	0.00	C
ATOM	5576	HA	ARG	A	382	32.110	34.810	35.986	1.00	0.00	H
ATOM	5577	CB	ARG	A	382	31.536	36.682	35.119	1.00	0.00	C
ATOM	5578	HB1	ARG	A	382	31.909	36.450	34.120	1.00	0.00	H
ATOM	5579	HB2	ARG	A	382	30.638	37.285	35.013	1.00	0.00	H
ATOM	5580	CG	ARG	A	382	32.621	37.476	35.871	1.00	0.00	C
ATOM	5581	HG1	ARG	A	382	32.315	37.643	36.900	1.00	0.00	H
ATOM	5582	HG2	ARG	A	382	33.533	36.879	35.886	1.00	0.00	H
ATOM	5583	CD	ARG	A	382	32.953	38.834	35.239	1.00	0.00	C
ATOM	5584	HD1	ARG	A	382	33.798	39.270	35.775	1.00	0.00	H
ATOM	5585	HD2	ARG	A	382	33.253	38.678	34.207	1.00	0.00	H
ATOM	5586	NE	ARG	A	382	31.819	39.773	35.264	1.00	0.00	N
ATOM	5587	HE	ARG	A	382	30.957	39.468	35.709	1.00	0.00	H
ATOM	5588	CZ	ARG	A	382	31.779	40.954	34.662	1.00	0.00	C
ATOM	5589	NH1	ARG	A	382	32.820	41.475	34.049	1.00	0.00	N
ATOM	5590	HH11	ARG	A	382	33.734	41.035	34.075	1.00	0.00	H
ATOM	5591	HH12	ARG	A	382	32.748	42.392	33.624	1.00	0.00	H
ATOM	5592	NH2	ARG	A	382	30.663	41.641	34.664	1.00	0.00	N
ATOM	5593	HH21	ARG	A	382	29.872	41.317	35.210	1.00	0.00	H
ATOM	5594	HH22	ARG	A	382	30.617	42.527	34.175	1.00	0.00	H
ATOM	5595	C	ARG	A	382	30.576	35.610	37.239	1.00	0.00	C
ATOM	5596	O	ARG	A	382	31.298	35.514	38.232	1.00	0.00	O
ATOM	5597	N	GLN	A	383	29.268	35.876	37.349	1.00	0.00	N
ATOM	5598	H	GLN	A	383	28.717	35.959	36.500	1.00	0.00	H
ATOM	5599	CA	GLN	A	383	28.613	36.116	38.640	1.00	0.00	C
ATOM	5600	HA	GLN	A	383	29.235	36.807	39.213	1.00	0.00	H
ATOM	5601	CB	GLN	A	383	27.238	36.777	38.440	1.00	0.00	C
ATOM	5602	HB1	GLN	A	383	27.379	37.715	37.906	1.00	0.00	H
ATOM	5603	HB2	GLN	A	383	26.608	36.129	37.829	1.00	0.00	H
ATOM	5604	CG	GLN	A	383	26.505	37.068	39.769	1.00	0.00	C
ATOM	5605	HG1	GLN	A	383	25.568	37.580	39.546	1.00	0.00	H
ATOM	5606	HG2	GLN	A	383	26.252	36.129	40.263	1.00	0.00	H
ATOM	5607	CD	GLN	A	383	27.309	37.946	40.729	1.00	0.00	C
ATOM	5608	OE1	GLN	A	383	27.628	39.085	40.436	1.00	0.00	O
ATOM	5609	NE2	GLN	A	383	27.708	37.478	41.896	1.00	0.00	N
ATOM	5610	HE21	GLN	A	383	27.575	36.505	42.149	1.00	0.00	H
ATOM	5611	HE22	GLN	A	383	28.368	38.047	42.405	1.00	0.00	H
ATOM	5612	C	GLN	A	383	28.488	34.841	39.477	1.00	0.00	C
ATOM	5613	O	GLN	A	383	28.866	34.859	40.645	1.00	0.00	O

ATOM	5614	N	GLU	A	384	27.988	33.732	38.928	1.00	0.00	N
ATOM	5615	H	GLU	A	384	27.682	33.745	37.956	1.00	0.00	H
ATOM	5616	CA	GLU	A	384	27.776	32.500	39.705	1.00	0.00	C
ATOM	5617	HA	GLU	A	384	27.226	32.753	40.613	1.00	0.00	H
ATOM	5618	CB	GLU	A	384	26.918	31.522	38.891	1.00	0.00	C
ATOM	5619	HB1	GLU	A	384	27.400	31.367	37.925	1.00	0.00	H
ATOM	5620	HB2	GLU	A	384	26.891	30.563	39.403	1.00	0.00	H
ATOM	5621	CG	GLU	A	384	25.474	32.002	38.648	1.00	0.00	C
ATOM	5622	HG1	GLU	A	384	25.480	33.019	38.254	1.00	0.00	H
ATOM	5623	HG2	GLU	A	384	25.046	31.362	37.880	1.00	0.00	H
ATOM	5624	CD	GLU	A	384	24.557	31.932	39.876	1.00	0.00	C
ATOM	5625	OE1	GLU	A	384	24.421	30.840	40.476	1.00	0.00	O
ATOM	5626	OE2	GLU	A	384	23.890	32.943	40.201	1.00	0.00	O
ATOM	5627	C	GLU	A	384	29.106	31.851	40.149	1.00	0.00	C
ATOM	5628	O	GLU	A	384	29.158	31.194	41.195	1.00	0.00	O
ATOM	5629	N	LEU	A	385	30.202	32.093	39.414	1.00	0.00	N
ATOM	5630	H	LEU	A	385	30.083	32.569	38.525	1.00	0.00	H
ATOM	5631	CA	LEU	A	385	31.576	31.775	39.833	1.00	0.00	C
ATOM	5632	HA	LEU	A	385	31.558	30.863	40.427	1.00	0.00	H
ATOM	5633	CB	LEU	A	385	32.446	31.540	38.582	1.00	0.00	C
ATOM	5634	HB1	LEU	A	385	32.412	32.439	37.965	1.00	0.00	H
ATOM	5635	HB2	LEU	A	385	33.478	31.395	38.902	1.00	0.00	H
ATOM	5636	CG	LEU	A	385	32.051	30.321	37.724	1.00	0.00	C
ATOM	5637	HG	LEU	A	385	31.002	30.396	37.439	1.00	0.00	H
ATOM	5638	CD1	LEU	A	385	32.894	30.310	36.443	1.00	0.00	C
ATOM	5639	HD11	LEU	A	385	32.620	29.450	35.836	1.00	0.00	H
ATOM	5640	HD12	LEU	A	385	32.710	31.218	35.868	1.00	0.00	H
ATOM	5641	HD13	LEU	A	385	33.953	30.249	36.692	1.00	0.00	H
ATOM	5642	CD2	LEU	A	385	32.266	28.999	38.477	1.00	0.00	C
ATOM	5643	HD21	LEU	A	385	32.024	28.164	37.820	1.00	0.00	H
ATOM	5644	HD22	LEU	A	385	33.303	28.910	38.799	1.00	0.00	H
ATOM	5645	HD23	LEU	A	385	31.616	28.947	39.349	1.00	0.00	H
ATOM	5646	C	LEU	A	385	32.214	32.855	40.732	1.00	0.00	C
ATOM	5647	O	LEU	A	385	33.312	32.644	41.244	1.00	0.00	O
ATOM	5648	N	SER	A	386	31.565	34.006	40.920	1.00	0.00	N
ATOM	5649	H	SER	A	386	30.643	34.087	40.505	1.00	0.00	H
ATOM	5650	CA	SER	A	386	31.998	35.150	41.754	1.00	0.00	C
ATOM	5651	HA	SER	A	386	31.205	35.898	41.716	1.00	0.00	H
ATOM	5652	CB	SER	A	386	32.126	34.749	43.235	1.00	0.00	C
ATOM	5653	HB1	SER	A	386	32.980	34.088	43.374	1.00	0.00	H
ATOM	5654	HB2	SER	A	386	32.289	35.648	43.830	1.00	0.00	H
ATOM	5655	OG	SER	A	386	30.945	34.104	43.694	1.00	0.00	O
ATOM	5656	HG	SER	A	386	31.028	33.145	43.512	1.00	0.00	H
ATOM	5657	C	SER	A	386	33.261	35.867	41.218	1.00	0.00	C
ATOM	5658	O	SER	A	386	33.914	36.647	41.915	1.00	0.00	O
ATOM	5659	N	LEU	A	387	33.615	35.647	39.947	1.00	0.00	N
ATOM	5660	H	LEU	A	387	32.972	35.095	39.388	1.00	0.00	H
ATOM	5661	CA	LEU	A	387	34.820	36.151	39.268	1.00	0.00	C
ATOM	5662	HA	LEU	A	387	35.649	36.150	39.974	1.00	0.00	H
ATOM	5663	CB	LEU	A	387	35.156	35.181	38.112	1.00	0.00	C
ATOM	5664	HB1	LEU	A	387	34.299	35.134	37.439	1.00	0.00	H
ATOM	5665	HB2	LEU	A	387	35.999	35.576	37.543	1.00	0.00	H
ATOM	5666	CG	LEU	A	387	35.523	33.751	38.561	1.00	0.00	C
ATOM	5667	HG	LEU	A	387	34.727	33.359	39.188	1.00	0.00	H

ATOM	5668	CD1	LEU	A	387	35.638	32.835	37.335	1.00	0.00	C
ATOM	5669	HD11	LEU	A	387	35.873	31.818	37.649	1.00	0.00	H
ATOM	5670	HD12	LEU	A	387	34.693	32.826	36.794	1.00	0.00	H
ATOM	5671	HD13	LEU	A	387	36.420	33.195	36.672	1.00	0.00	H
ATOM	5672	CD2	LEU	A	387	36.829	33.720	39.367	1.00	0.00	C
ATOM	5673	HD21	LEU	A	387	37.094	32.695	39.613	1.00	0.00	H
ATOM	5674	HD22	LEU	A	387	37.635	34.164	38.786	1.00	0.00	H
ATOM	5675	HD23	LEU	A	387	36.712	34.267	40.302	1.00	0.00	H
ATOM	5676	C	LEU	A	387	34.687	37.616	38.800	1.00	0.00	C
ATOM	5677	O	LEU	A	387	35.344	38.033	37.847	1.00	0.00	O
ATOM	5678	N	VAL	A	388	33.820	38.392	39.448	1.00	0.00	N
ATOM	5679	H	VAL	A	388	33.438	38.015	40.307	1.00	0.00	H
ATOM	5680	CA	VAL	A	388	33.474	39.774	39.077	1.00	0.00	C
ATOM	5681	HA	VAL	A	388	33.490	39.840	37.992	1.00	0.00	H
ATOM	5682	CB	VAL	A	388	32.046	40.140	39.544	1.00	0.00	C
ATOM	5683	HB	VAL	A	388	32.020	40.099	40.631	1.00	0.00	H
ATOM	5684	CG1	VAL	A	388	31.658	41.551	39.082	1.00	0.00	C
ATOM	5685	HG11	VAL	A	388	30.655	41.793	39.434	1.00	0.00	H
ATOM	5686	HG12	VAL	A	388	32.345	42.290	39.487	1.00	0.00	H
ATOM	5687	HG13	VAL	A	388	31.688	41.604	37.993	1.00	0.00	H
ATOM	5688	CG2	VAL	A	388	30.991	39.150	39.021	1.00	0.00	C
ATOM	5689	HG21	VAL	A	388	30.006	39.444	39.378	1.00	0.00	H
ATOM	5690	HG22	VAL	A	388	30.980	39.153	37.933	1.00	0.00	H
ATOM	5691	HG23	VAL	A	388	31.198	38.143	39.383	1.00	0.00	H
ATOM	5692	C	VAL	A	388	34.493	40.747	39.705	1.00	0.00	C
ATOM	5693	O	VAL	A	388	34.753	40.616	40.904	1.00	0.00	O
ATOM	5694	N	PRO	A	389	35.061	41.717	38.956	1.00	0.00	N
ATOM	5695	CD	PRO	A	389	34.941	41.887	37.515	1.00	0.00	C
ATOM	5696	HD1	PRO	A	389	34.026	42.436	37.287	1.00	0.00	H
ATOM	5697	HD2	PRO	A	389	34.958	40.933	36.989	1.00	0.00	H
ATOM	5698	CG	PRO	A	389	36.157	42.708	37.100	1.00	0.00	C
ATOM	5699	HG1	PRO	A	389	35.951	43.310	36.215	1.00	0.00	H
ATOM	5700	HG2	PRO	A	389	36.998	42.038	36.928	1.00	0.00	H
ATOM	5701	CB	PRO	A	389	36.436	43.579	38.322	1.00	0.00	C
ATOM	5702	HB1	PRO	A	389	35.842	44.491	38.249	1.00	0.00	H
ATOM	5703	HB2	PRO	A	389	37.495	43.829	38.401	1.00	0.00	H
ATOM	5704	CA	PRO	A	389	35.969	42.723	39.508	1.00	0.00	C
ATOM	5705	HA	PRO	A	389	36.832	42.218	39.943	1.00	0.00	H
ATOM	5706	C	PRO	A	389	35.283	43.596	40.565	1.00	0.00	C
ATOM	5707	O	PRO	A	389	34.185	44.100	40.337	1.00	0.00	O
ATOM	5708	N	ARG	A	390	35.942	43.814	41.705	1.00	0.00	N
ATOM	5709	H	ARG	A	390	36.848	43.382	41.825	1.00	0.00	H
ATOM	5710	CA	ARG	A	390	35.554	44.854	42.679	1.00	0.00	C
ATOM	5711	HA	ARG	A	390	34.469	44.962	42.674	1.00	0.00	H
ATOM	5712	CB	ARG	A	390	35.966	44.453	44.112	1.00	0.00	C
ATOM	5713	HB1	ARG	A	390	37.053	44.473	44.202	1.00	0.00	H
ATOM	5714	HB2	ARG	A	390	35.553	45.189	44.805	1.00	0.00	H
ATOM	5715	CG	ARG	A	390	35.455	43.056	44.506	1.00	0.00	C
ATOM	5716	HG1	ARG	A	390	34.396	42.979	44.256	1.00	0.00	H
ATOM	5717	HG2	ARG	A	390	35.995	42.307	43.924	1.00	0.00	H
ATOM	5718	CD	ARG	A	390	35.635	42.729	45.998	1.00	0.00	C
ATOM	5719	HD1	ARG	A	390	36.685	42.858	46.268	1.00	0.00	H
ATOM	5720	HD2	ARG	A	390	35.023	43.407	46.594	1.00	0.00	H
ATOM	5721	NE	ARG	A	390	35.228	41.338	46.249	1.00	0.00	N

```
ATOM   5722  HE  ARG A 390      34.748  40.891  45.475  1.00  0.00           H
ATOM   5723  CZ  ARG A 390      35.442  40.580  47.315  1.00  0.00           C
ATOM   5724  NH1 ARG A 390      35.965  41.032  48.432  1.00  0.00           N
ATOM   5725 HH11 ARG A 390      36.086  42.026  48.576  1.00  0.00           H
ATOM   5726 HH12 ARG A 390      36.119  40.400  49.203  1.00  0.00           H
ATOM   5727  NH2 ARG A 390      35.117  39.310  47.265  1.00  0.00           N
ATOM   5728 HH21 ARG A 390      34.757  38.916  46.409  1.00  0.00           H
ATOM   5729 HH22 ARG A 390      35.279  38.684  48.046  1.00  0.00           H
ATOM   5730  C   ARG A 390      36.129  46.230  42.293  1.00  0.00           C
ATOM   5731  OC1 ARG A 390      35.639  47.255  42.817  1.00  0.00           O
ATOM   5732  OC2 ARG A 390      37.097  46.280  41.502  1.00  0.00           O
ATOM   5733  C   UNL B 391      30.678  17.654  16.357  1.00  0.00           C
ATOM   5734  C1  UNL B 391      30.291  19.027  15.764  1.00  0.00           C
ATOM   5735  C2  UNL B 391      31.425  20.049  15.977  1.00  0.00           C
ATOM   5736  C3  UNL B 391      31.828  20.085  17.459  1.00  0.00           C
ATOM   5737  C4  UNL B 391      31.977  18.698  18.120  1.00  0.00           C
ATOM   5738  C5  UNL B 391      31.918  18.774  19.650  1.00  0.00           C
ATOM   5739  C6  UNL B 391      28.899  19.419  13.715  1.00  0.00           C
ATOM   5740  C7  UNL B 391      28.696  18.992  12.276  1.00  0.00           C
ATOM   5741  O   UNL B 391      30.932  17.772  17.744  1.00  0.00           O
ATOM   5742  O1  UNL B 391      31.076  21.346  15.498  1.00  0.00           O
ATOM   5743  O2  UNL B 391      33.104  20.793  17.524  1.00  0.00           O
ATOM   5744  O3  UNL B 391      32.907  17.922  20.278  1.00  0.00           O
ATOM   5745  O4  UNL B 391      28.129  20.236  14.252  1.00  0.00           O
ATOM   5746  N   UNL B 391      29.952  18.844  14.359  1.00  0.00           N
ATOM   5747  O5  UNL B 391      31.713  17.002  15.635  1.00  0.00           O
ATOM   5748  C8  UNL B 391      33.359  21.722  18.681  1.00  0.00           C
ATOM   5749  C9  UNL B 391      34.524  22.600  18.192  1.00  0.00           C
ATOM   5750  C10 UNL B 391      34.051  23.666  17.150  1.00  0.00           C
ATOM   5751  C11 UNL B 391      32.524  23.775  17.080  1.00  0.00           C
ATOM   5752  C12 UNL B 391      31.883  23.644  18.464  1.00  0.00           C
ATOM   5753  C13 UNL B 391      30.367  23.733  18.451  1.00  0.00           C
ATOM   5754  C14 UNL B 391      35.075  23.548  20.462  1.00  0.00           C
ATOM   5755  C15 UNL B 391      36.228  23.897  21.372  1.00  0.00           C
ATOM   5756  N1  UNL B 391      35.416  23.088  19.238  1.00  0.00           N
ATOM   5757  O6  UNL B 391      34.588  23.320  15.849  1.00  0.00           O
ATOM   5758  O7  UNL B 391      32.055  24.998  16.475  1.00  0.00           O
ATOM   5759  O8  UNL B 391      32.186  22.340  19.029  1.00  0.00           O
ATOM   5760  O9  UNL B 391      29.745  22.817  17.535  1.00  0.00           O
ATOM   5761  O10 UNL B 391      33.884  23.668  20.833  1.00  0.00           O
ATOM   5762  C16 UNL B 391      31.874  24.934  15.085  1.00  0.00           C
ATOM   5763  C17 UNL B 391      30.687  25.802  14.628  1.00  0.00           C
ATOM   5764  C18 UNL B 391      30.579  25.801  13.078  1.00  0.00           C
ATOM   5765  C19 UNL B 391      31.950  26.093  12.427  1.00  0.00           C
ATOM   5766  C20 UNL B 391      33.027  25.191  13.039  1.00  0.00           C
ATOM   5767  C21 UNL B 391      34.450  25.455  12.589  1.00  0.00           C
ATOM   5768  C22 UNL B 391      28.862  26.005  16.292  1.00  0.00           C
ATOM   5769  C23 UNL B 391      27.697  25.278  16.922  1.00  0.00           C
ATOM   5770  N2  UNL B 391      29.452  25.347  15.243  1.00  0.00           N
ATOM   5771  O11 UNL B 391      29.602  26.754  12.698  1.00  0.00           O
ATOM   5772  O12 UNL B 391      31.912  25.984  11.002  1.00  0.00           O
ATOM   5773  O13 UNL B 391      33.056  25.415  14.458  1.00  0.00           O
ATOM   5774  O14 UNL B 391      35.355  24.514  13.187  1.00  0.00           O
ATOM   5775  O15 UNL B 391      29.249  27.108  16.694  1.00  0.00           O
```

```
ATOM   5776  H   UNL B 391      29.812  16.978  16.284  1.00  0.00           H
ATOM   5777  H1  UNL B 391      29.395  19.385  16.293  1.00  0.00           H
ATOM   5778  H2  UNL B 391      32.291  19.728  15.375  1.00  0.00           H
ATOM   5779  H3  UNL B 391      31.076  20.658  18.011  1.00  0.00           H
ATOM   5780  H4  UNL B 391      32.953  18.275  17.828  1.00  0.00           H
ATOM   5781  H5  UNL B 391      30.925  18.430  19.969  1.00  0.00           H
ATOM   5782  H6  UNL B 391      32.023  19.806  20.006  1.00  0.00           H
ATOM   5783  H7  UNL B 391      29.077  19.779  11.610  1.00  0.00           H
ATOM   5784  H8  UNL B 391      29.196  18.050  12.025  1.00  0.00           H
ATOM   5785  H9  UNL B 391      27.619  18.890  12.093  1.00  0.00           H
ATOM   5786  H10 UNL B 391      30.534  21.803  16.197  1.00  0.00           H
ATOM   5787  H11 UNL B 391      33.642  18.506  20.545  1.00  0.00           H
ATOM   5788  H12 UNL B 391      32.584  17.435  15.786  1.00  0.00           H
ATOM   5789  H13 UNL B 391      35.181  21.939  17.615  1.00  0.00           H
ATOM   5790  H14 UNL B 391      34.487  24.638  17.410  1.00  0.00           H
ATOM   5791  H15 UNL B 391      32.164  22.929  16.474  1.00  0.00           H
ATOM   5792  H16 UNL B 391      32.283  24.401  19.158  1.00  0.00           H
ATOM   5793  H17 UNL B 391      30.011  23.544  19.474  1.00  0.00           H
ATOM   5794  H18 UNL B 391      30.089  24.761  18.186  1.00  0.00           H
ATOM   5795  H19 UNL B 391      36.178  24.960  21.634  1.00  0.00           H
ATOM   5796  H20 UNL B 391      36.118  23.338  22.311  1.00  0.00           H
ATOM   5797  H21 UNL B 391      37.206  23.689  20.923  1.00  0.00           H
ATOM   5798  H22 UNL B 391      34.017  22.604  15.511  1.00  0.00           H
ATOM   5799  H23 UNL B 391      29.386  22.010  18.077  1.00  0.00           H
ATOM   5800  H24 UNL B 391      31.703  23.878  14.777  1.00  0.00           H
ATOM   5801  H25 UNL B 391      30.856  26.831  14.977  1.00  0.00           H
ATOM   5802  H26 UNL B 391      30.285  24.778  12.753  1.00  0.00           H
ATOM   5803  H27 UNL B 391      32.213  27.141  12.654  1.00  0.00           H
ATOM   5804  H28 UNL B 391      32.779  24.128  12.832  1.00  0.00           H
ATOM   5805  H29 UNL B 391      34.712  26.492  12.868  1.00  0.00           H
ATOM   5806  H30 UNL B 391      34.533  25.357  11.502  1.00  0.00           H
ATOM   5807  H31 UNL B 391      26.963  24.979  16.159  1.00  0.00           H
ATOM   5808  H32 UNL B 391      28.057  24.363  17.411  1.00  0.00           H
ATOM   5809  H33 UNL B 391      27.234  25.929  17.669  1.00  0.00           H
ATOM   5810  H34 UNL B 391      29.095  26.451  11.883  1.00  0.00           H
ATOM   5811  H35 UNL B 391      31.329  25.218  10.746  1.00  0.00           H
ATOM   5812  H36 UNL B 391      35.076  24.432  14.128  1.00  0.00           H
ATOM   5813  H37 UNL B 391      33.673  21.108  19.534  1.00  0.00           H
ATOM   5814  H38 UNL B 391      30.568  18.227  13.828  1.00  0.00           H
ATOM   5815  H39 UNL B 391      36.433  22.935  19.017  1.00  0.00           H
ATOM   5816  H40 UNL B 391      29.227  24.356  15.154  1.00  0.00           H
ATOM   5817  K      K   392      38.897  18.408  19.770  1.00  0.00           K
TER
ENDMDL
```
