# Supplementary material for: Elucidation of the Catalytic Apparatus and Mechanism of Human Chitotriosidase‑1
Source: ACS Catal. 2025 Sep 19;15(19):16748–61. doi: 10.1021/acscatal.5c00507 (PMC12502790; doi:10.1021/acscatal.5c00507)
Supplement: Supplementary file 6 [file cs5c00507_si_006.pdf]

Supporting Information

Elucidation of the Catalytic Apparatus and Mechanism of Human Chitotriosidase-1.

Dorota Niedzialek[†]*, *Grzegorz Wieczorek*[‡], *Katarzyna Drzewicka*[‡], *Anna Antosiewicz*[‡], *Mariusz Milewski*[‡§], *Agnieszka Bartoszewicz*[‡], *Jacek Olczak*[‡§]*, *Zbigniew Zasłona*[‡]*

[†]Institute of Biochemistry and Biophysics of the Polish Academy of Sciences, Pawińskiego 5a, 02-106 Warsaw, Poland
[‡]Molecure S.A., Żwirki i Wigury 101, 02-089 Warsaw, Poland
[§]ASCENTIA sp. z o.o., BRaIn Laboratories of the Medical University of Lodz, Czechosłowacka 4, 92-216 Lodz, Poland

*E-mail: dniedzialek@ibb.waw.pl

*E-mail: j.olczak@ascentiabio.com

*E-mail: z.zaslona@molecure.com

Supplementary Discussions

1. Roles of human chitinases in biological processes and human diseases:

GlcNAc-containing LacNAc glycan is the main ligand for sugar-binding receptors[18] known to be modified by hCHIT1[6]. Abnormal expressions of LacNAc are associated with cancers[19], autoimmune[11] inflammatory[12] and lysosomal storage diseases associated with overexpression of hCHIT1[13]. Since hCHIT1 exhibits high transglycosylation activity independent of typical 'transglycosylation conditions'[7], it may play a harmful role in the human body by contributing to pathological glycosylation processes.[14,20] For instance, a recent study demonstrated that increased expression and activity of hCHIT1 are linked to Alzheimer's disease.[15] This result is consistent with previous findings that genetic inactivation of hCHIT1 is strongly associated with human longevity and several phenotypes of healthy ageing.[8] Other evidence suggests that some genetic variations that disrupt the enzymatic activity of hCHIT1 protect against the progression of non-alcoholic fatty liver disease[16] and reduce susceptibility to sarcoidosis[17]. In

healthy individuals, macrophages produce low levels of hCHIT1. However, in response to pro-inflammatory signals, the secretion of hCHIT1 significantly increases.[9,21] Furthermore, GlcNAc oligomeric products generated by hCHIT1 function as molecular patterns that can be detected by toll-like receptors TLR9 in neutrophils and NOD-2 in macrophages (**Figure 1D**).[22] This, in turn, may lead to induction of enhanced hCHIT1 expression, production of various cytokines and chemokines, and initiation of adaptive immune responses.[23] Hence, hCHIT1 seems to have different pathogenic roles depending on the associated diseases and activated cell types during the immune reaction.[10]

2. The role of M^+ in facilitating proton transfer during the catalytic process

The hydrophobic characters of the neighboring Ala183, Met210, and Trp358 residues increase pK_a of Asp138 to about 10, resulting in its protonation at the optimal operational pH of 7.5 for hCHIT1. Glu140, which protonates the glycosidic bond at the first stage of the catalysis, has pK_a of 6 before substrate binding, and stays deprotonated but stabilized by the hydrogen bond interaction with the adjacent tyrosine (Tyr141). To enable the substrate binding and initiate the first stage of catalysis, protonated Asp138 must transfer hydrogen to deprotonated Glu140. To enable proton transfer, the pK_a value of Asp138 needs to drop significantly. The QM/MM simulations showed this happening after Met210 hides its methyl from the active site by rotation guided by the movement of M^+ (*i.e.*, in line with the MD simulations and analysis of existing hCHIT1 crystal structures). When the methyl group of methionine shifts away from Asp138, its p*Ka* value decreases sufficiently to allow proton release and subsequent transfer to Glu140. This finding corresponds with a previous report showing that, prior to sugar binding, the p*Ka* value of Glu140 increases from 6.2 to 8.7.[43] M^+, which acts as electrophile, by migrating along the main chain of the β4 strand closer to Glu140 can locally increase its p*Ka*. The primary role of Asp138 in the hydrolysis process is to form a hydrogen bond with the nitrogen atom of the 2-acetamido group, stabilizing the *twisted boat*

conformation with the *cis* orientation of the 2-acetamido group in the -1 sugar substrate within the active site and facilitating the transition between the transient oxazolinium and oxazoline intermediate states. The 2-acetamido group forms with Asp138 and Tyr212 strong hydrogen bonds, which tighten up as the transition state (TS1) approaches. Triggered by Brownian conformational fluctuations in the adjacent subdomains, the side chain of Tyr212 directs the bound carbonyl oxygen of the 2-acetamido group towards the anomeric carbon as the β6 strand moves outward from the enzyme. Simultaneously, the nitrogen of the 2-acetamido group, which shares a proton with the side chain of Asp138, is pushed in the opposite direction by the β4 strand. The conjugated proton transfer through the 2-acetamido group, specifically between Tyr212 and the carbonyl oxygen and subsequently between the nitrogen and Asp138, facilitates the formation of the intermediate state (oxazoline). This process is promoted by the sulfur orbitals of Met210, located 3.7 Å away, and can be further enhanced by the ionic interactions of alkali metal ions.[44] The polarized carbonyl oxygen acts as a nucleophile that attacks the C1 anomeric center and by creating transition state, breaks the glycosidic bond. The simultaneous proton transfer from Glu140 to the oxygen of the glycosylic bond of the leaving group promotes departure of the +1 sugar. The leaving group releases the tryptophane 'lid', letting water/oligosaccharide enter the active site and start the second stage of the catalysis. In hydrolysis, deprotonated Glu140 functions as a general base, facilitating the nucleophilic attack of a water molecule on the anomeric carbon. Subsequently, the broken oxazoline/oxazolinium ring reopens into the 2-acetamido group, leading to the ejection of the -1 sugar from the active site. The dual role of Glu140 as a general acid/base during catalytic reaction places specific demands upon its protonation state, and hence its pK_a values at each stage of catalysis.

Supplementary Figures

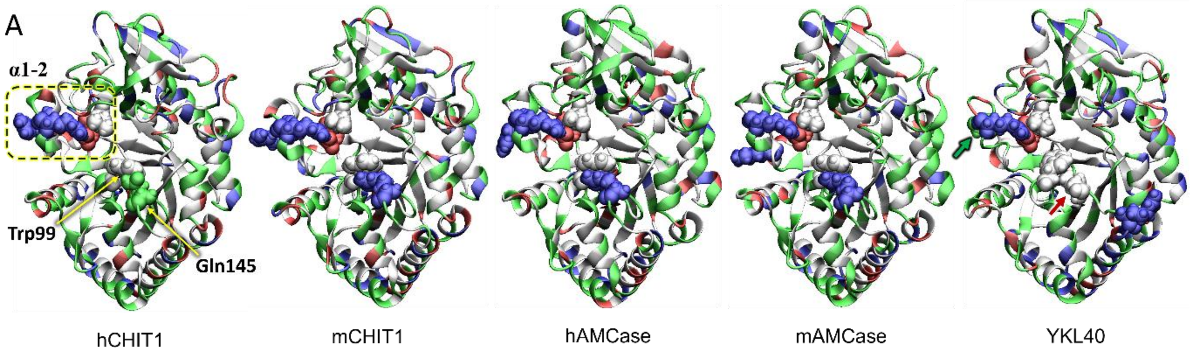

B

	Subunit α1-2<br>non-conserved	Motif I (β3)<br>KXXXXXGGW	Motif II (β4)<br>FDGLDXDWEXP	Loop L4<br>non-conserved	Motif III (β6)<br>MXYDXXG	Motif IV (β8)<br>GXXXWXXDLDD
hCHIT1	YRQGEAR-FL	KTLLAIGGW	FDGLDLDWEYP	GSQGSP	MAYDFHG	GAMVWALDLDD
mCHIT1	YRTEAVR-FF	KTLLAIGGW	FDGLDLDWEFP	GGRGSP	MAYDFHG	GAMVWVLDLDD
hAMCase	YRPGLGR-FM	KTLLAIGGW	FDGLDFDWEYP	GSRGSP	MTYDLHG	GAMVWAIDLDD
mAMCase	YRPGLGS-FK	KTLLAIGGW	FDGLDLDWEYP	GSRGSP	MTYDLHG	GAMIWAIDLDD
YKL40	YREGDGSCFP	KTLLSVGGW	FDGLDLAWL-P	GRRDKQ	MTYDFHG	GAMVWALDLDD

Figure S1. The four conserved motifs of hCHIT1, its closest homologues and catalytically inactive chitinase-like YKL40 protein. **A)** The catalytic domains of hCHIT1 (PDB ID: 4WKA), its mouse homologue (mCHIT1 (AlfaFold ID: AS-Q9D7Q1) as well as of human (hAMCase, PDB ID: 3FXY), mouse (mAMCase) homologues of Acidic Mammalian Chitinase and YKL40 (PDB ID: 7CJ2). All proteins comprise of similar classic TIM-barrel fold (*i.e.*, eight β strands tethered by eight α helices) despite substantial differences in composition (mCHIT1, hAMCase, mAMCase and YKL40 exhibit 78, 52, 50 and 52% similarity to hCHIT1). The colours of the cartoon representation correspond to hydrophobic (white), hydrophilic (green), acidic (red) and basic (blue) character of amino acids. The key residues at the entrance to the active site (Trp99 and X145) and residues of α1-2 subunit with the salt-bridge that connects it to the β8, together with conserved Leu362 are in vdW representation. Note, that only hCHIT1 has uncharged glutamine as residue X145 instead of arginine. The residues in a dotted circle are crucial for the inter-subunit mechanical signal transduction that trigger hCHIT1 heterodimer assembly/dissociation. The arrows indicate the main differences between YKL40

protein and the catalytically active chitinases: the red arrow indicates the E140A mutation within the catalytic triad while the green one emphasizes the lack of basic amino acid within the α1-2 subunit responsible for the heterodimer assembly. Note that only (h/m)CHIT1 and (h/m)AMCase homologs possess a carbohydrate binding domain, and other human chitinase-like proteins, such as YKL-40, consist only of the catalytic domain. **B)** Sequence comparison of the conserved motifs and key structural features in hCHIT1 and the four homologies pictured above. The Xs correspond to sequential differences within the conserved motifs. The key amino acids within each structural motif are marked in colour corresponding to its colour in the vdW representations above.

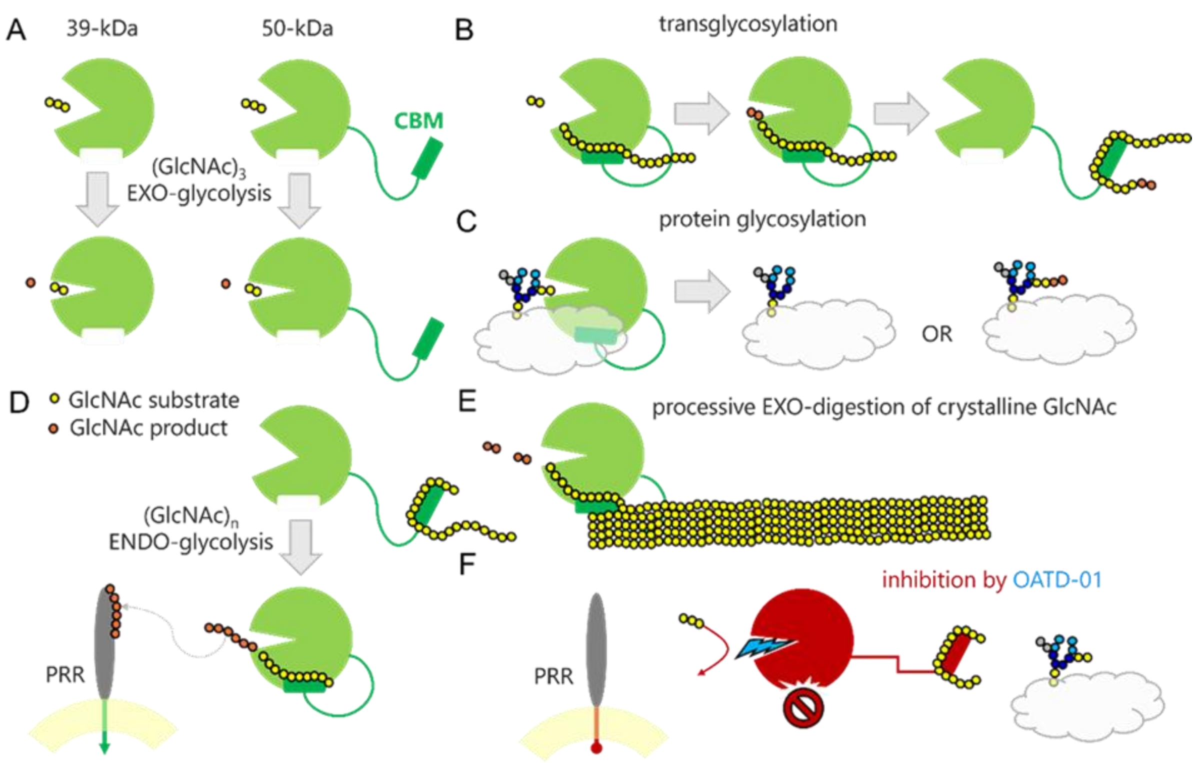

Figure S2. Schematic representation of hCHIT1 in its diverse modes of action. **A**) The full-length, 50-kDaisoform consists of the 39-kDa catalytic domain located at the N-terminus and the carbohydrate-binding module (CBM) connected by a linker (for details see Figure 1). Both isoforms can act as exochitinases that hydrolyze $(GlcNAc)_3$ substrate. **B**) Transglycosidase activity of hCHIT1 may be involved in **C**) pathological *N*-glycosylation processes. **D**) The full-length hCHIT1 can act as either an endochitinase capable of degrading GlcNAc polymers or **E)** an exochitinase able to processively digest crystalline $(GlcNAc)_n$ of pathogens. Product GlcNAc oligomers function as molecular patterns that can be detected by PRRs and initiate adaptive immune responses. **F**) The *first-in-class* inhibitor OATD-01 not only blocks the active site but, most importantly, interrupts communication across hCHIT1 subunits by generating structural distortions in the regions responsible for the assembly into the immunoglobulin-like 50-kDa isoform. This leads to impaired cooperativity with biological partners of hCHIT1.

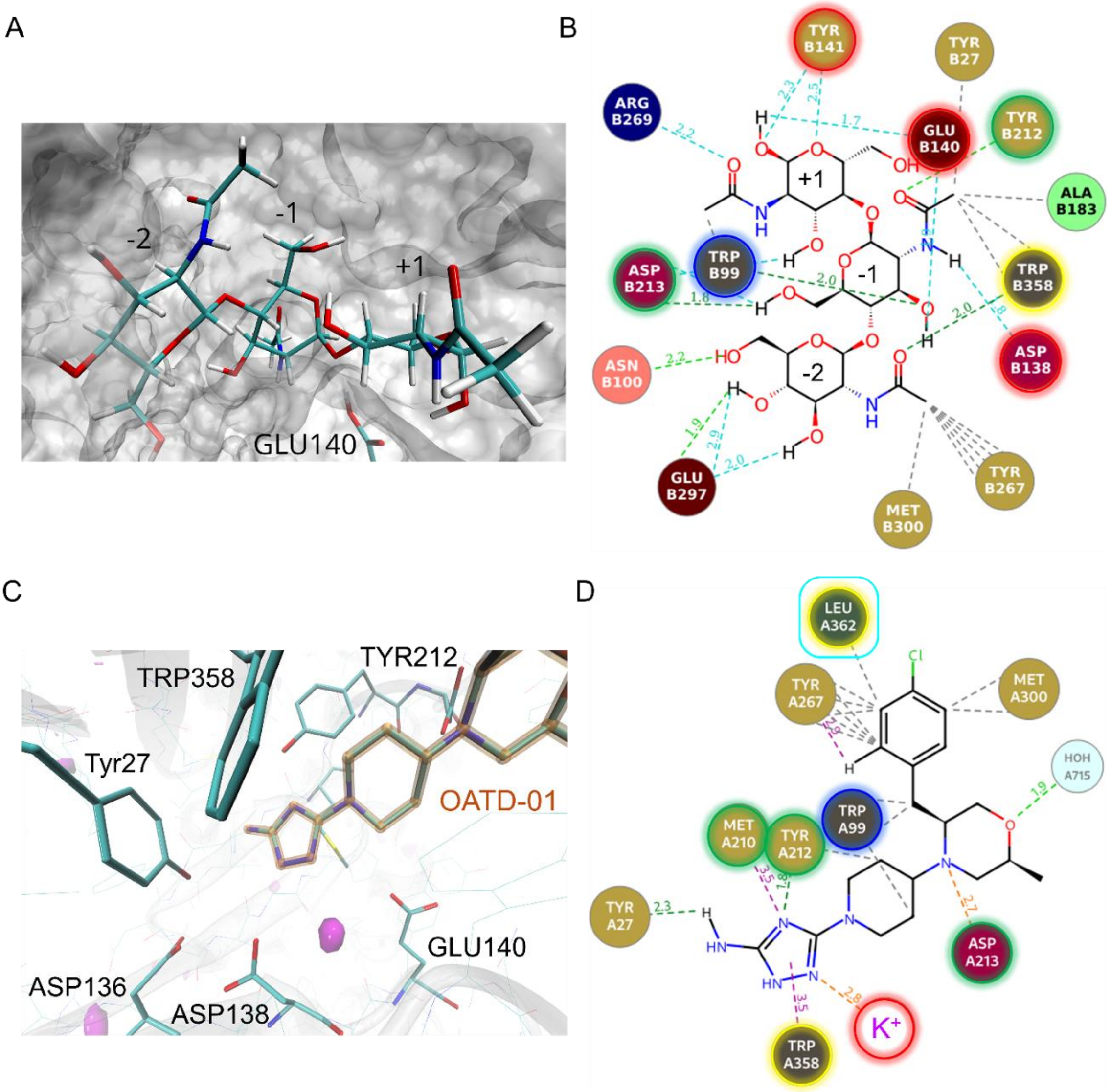

Figure S3. The substrate and OATD-01 inhibitor binding modes within the active site of hCHIT1. **A**) The white surface representation of the active site with the $(GlcNAc)_3$ substrate (represented as licorice) modelled at the QM/MM level of theory. The strong interaction between the active site cleft and GlcNAc substrate forces a distorted conformation of the -1 sugar and brings the susceptible glycosidic bond (between -1 and +1 GlcNAc units) in proximity to the proton donor residue (Glu140). **B**) The two-dimensional interaction map of the binding mode of $(GlcNAc)_3$ with the active site residues of hCHIT1. The colors of the frames around the symbols of the residues correspond to the colors of the conserved motifs assigned in Figure 1A. **C**) The electron density of the |Fo| − |Fc| map from the crystal structure

of hCHIT1 complexed with OATD-01 (PDB ID: 6ZE8) contoured at 5σ and shown in magenta surface representation. The analysis of both structures, including the elemental composition of the coordination sphere and the distances to the nearest atoms, identified the excess density as consistent with potassium ion being incorrectly modelled as water. **D**) The two-dimensional interaction map of the binding mode of OATD-01 with the active site residues of hCHIT1. The colors of the frames around the symbols of the residues correspond to the colors of the conserved motifs assigned in Figure 1A. The interaction with the alkali metal ion (here K^+) mediates the interaction with the motif I. The cyan frame indicates the residue Leu362 that plays a key role in the second mode of hCHIT1 inactivation by the OATD-01 inhibitor (*i.e.*, dissociation of the heterodimer).

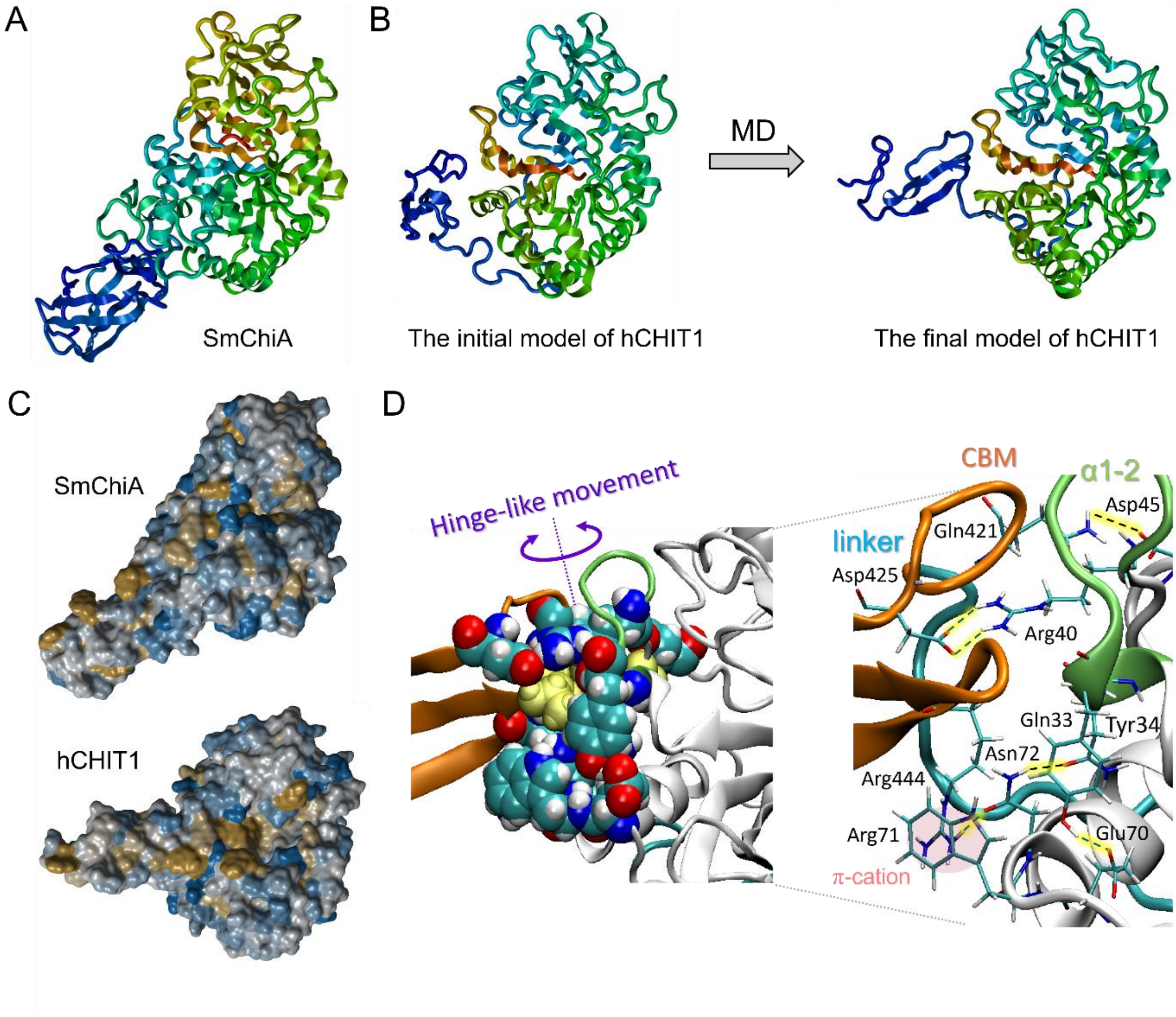

Figure S4. Modelling of the full-length hCHIT1 which, similarly to Chitinase A from *Serratia marcescens* (SmChiA), structurally belongs to the 'bacterial-type' chitinases. **A**) The rainbow cartoon representation of SmChiA (PDB ID: 5Z7M). **B)** The initial model of the full-length hCHIT1 was based on the 5HBF PDB entry with a structure of the unliganded hCHIT1 catalytic domain co-crystallized with CBM (without the linker). After modelling the missing linker, the structure was subjected to a series of extensive MD simulations until the final stable model of the immunoglobulin-like hCHIT1 heterodimer was obtained. **C**) The surface representations of the superimposed SmChiA structure (top) and the full-length hCHIT1 model (bottom). Both enzymes are colored according to hydrophobic/hydrophilic (yellow/blue) characters of amino acids. Note the similar substrate binding grooves with hydrophobic residues arranged linearly that continue along the carbohydrate-binding domain, promoting efficient binding of

polysaccharide substrates through CH-π interactions. **D)** The network of non-covalent interactions between the catalytic and CBM domains after assembly into the 50-kDa immunoglobulin-like heterodimer. The enzyme in cartoon representation is colored as in Figure 1A. The key residues shown in the left and right panels are in vdW and liquorice representations, respectively, and are colored according to atom types, except for the hydrophobic residues that are in the core of the assembly (*i.e.*, Leu42 within the α1-2 subunit is accommodated between Ala441 and Ala442 on CBM) which are marked in yellow. The hydrophobic residues are not shown in the right panel for clarity. The attachment of the domains is tight, so that the heterodimer remains stable at room temperature. However, it is flexible enough to perform hinge-like movement that allows the processed polysaccharide chain to slide towards the active site after each catalytic cycle.

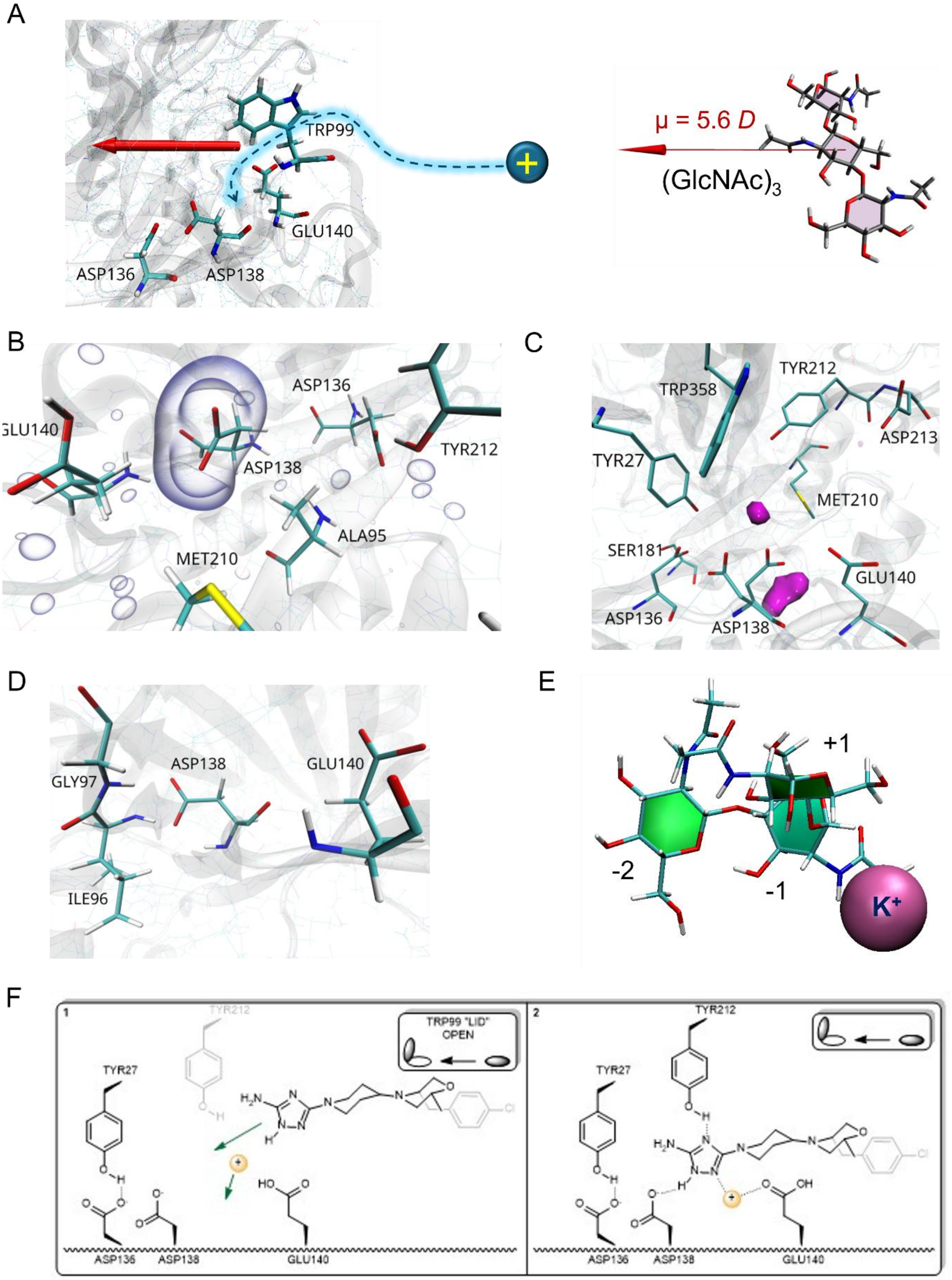

Figure S5. The electrostatics-based substrate/OATD-01 inhibitor attraction mechanism in hCHIT1. **A**) Electrostatic 'torch of guidance' effect in hCHIT1 which attracts $(GlcNAc)_3$ substrate and cations towards the catalytic site. The overall enzyme charge distribution

corresponds to the substantial dipole moment ($\mu = 242$ D) indicated by red block arrow, which aligns with the active site along the β4 strand. The optimized structure and the dipole moment for $(GlcNAc)_3$ were calculated at the DFT level of theory. The mutual alignment of the enzyme and substrate dipole moments guides the binding process. The enzyme is presented as a cartoon while the substrate and the key residues are in licorice representations. **B**) The three-dimensional isosurface map of electrostatic potential grid data, extracted from the QM/MM simulations of the unliganded hCHIT1, indicates a substantial negative charge above Asp138 responsible for electrostatic attraction of positive charges in this region. During substrate binding, M^+ diffuses towards the space between Asp138 and Glu140. **C**) The electron density of the $|Fo| - |Fc|$ map contoured at 5σ and shown in magenta surface representation extracted from the crystal structure of unliganded hCHIT1 (PDB ID: 5HBF). The excess density consistent with M^+ being incorrectly modelled as water. The analysis of the elemental composition of the coordination sphere and the distances to the nearest atoms, identified the excess densities as consistent with sodium ions. **D**) A snapshot from an MD simulation with polarizable force-field performed on ion-depleted, unliganded hCHIT1 system. In the absence of M^+, Asp138 bends towards the main chain of the neighboring β3 strand and forms a hydrogen bond with the main chain of Gly97 and Gly98 residues that immobilize the GGW loop. **E**) Preferential intermolecular arrangement of $(GlcNAc)_3$ molecule (in green licorice representation) and potassium ion (in pink vdW representation) during the substrate binding process, derived from the QM/MM calculations of the hCHIT1 with $(GlcNAc)_3$ model system. **F**) How OATD-01 exploits the 'torch of guidance' effect. Flowchart of the binding process of OATD-01, facilitated by the electrostatic attraction of the negatively charged triazole warhead with an alkali metal ion. The cation helps OATD-01 to adopt an optimal position in relation to the active site (*i.e.*, allowing optimal binding of the inhibitor warhead to Asp138 and Tyr212).

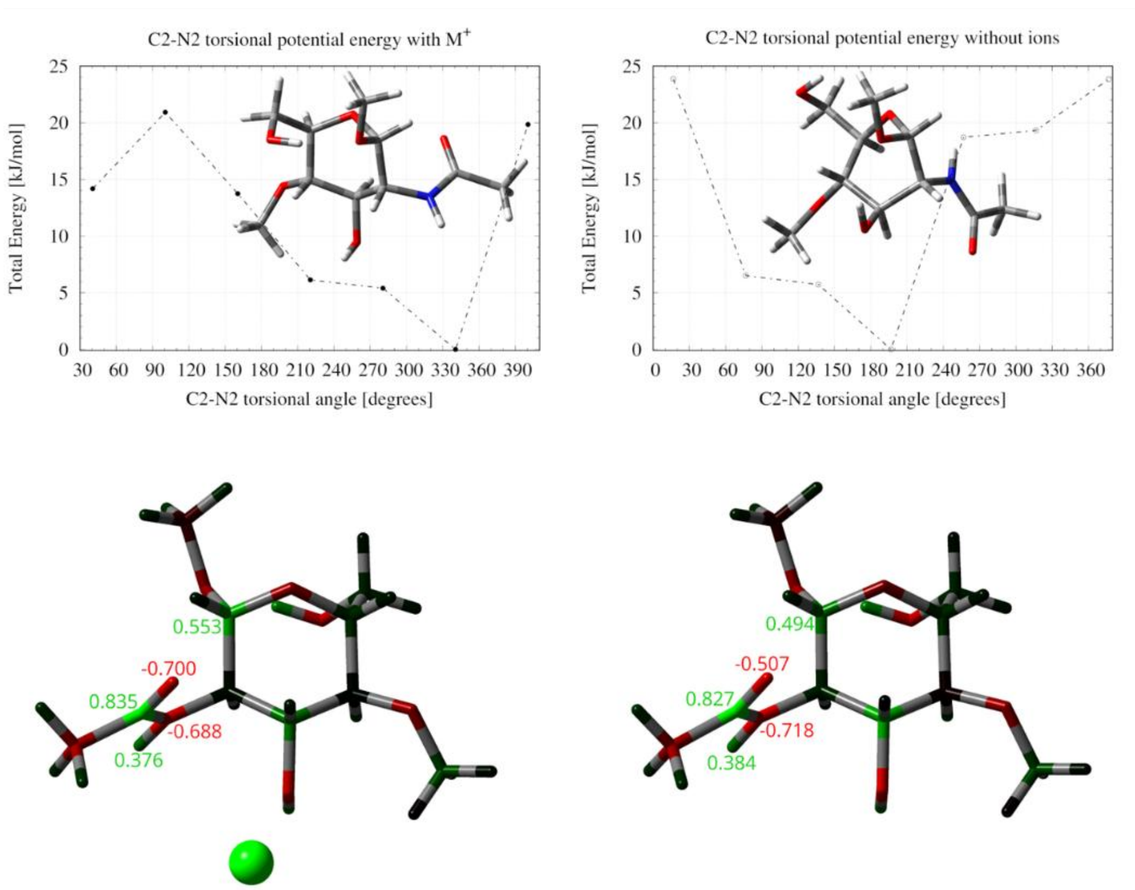

Figure S6. The influence of monovalent metal ions on achieving an optimal pre-catalytic *cis* conformation of the 2-acetamido group of the -1 subunit of the GlcNAc substrate. **A)** The torsional potential of the C2–N2 bond with (left) and without (right) alkali metal (M^+) ion. Note the difference in the position of global minimum between the two cases with the 2-acetamido group adapting *cis* and *trans* conformation with and without assistance of M^+ (sodium) ion, respectively. M^+ facilitates the correct orientation of the carbonyl oxygen of the 2-acetamido group for the nucleophilic attack of the carbonyl oxygen on the C1 anomeric center. **B)** Electrostatic potential charges distribution along the 2-acetamido group and the anomeric carbon with (left) and without (right) M^+ ion. Note the increase of the negative (red) and positive (green) charges on the carbonyl oxygen and the anomeric center, respectively, in the case with M^+ ion. The green sphere indicates M^+ ion, and the charges are in atomic units.

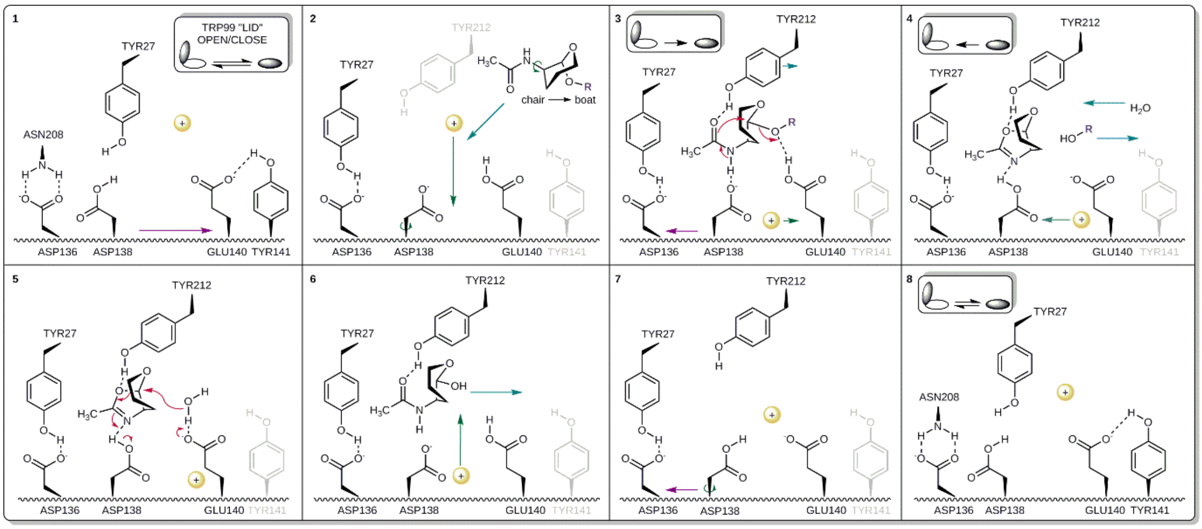

Figure S7. The revised mechanism of hCHIT1 catalysis based on multiscale simulations. For drawing clarity, the -2 sugar is not presented, the -1 sugar is presented without other substituents than the 2-acetamido group, and the +1 sugar is represented as "R". The electron transfer is indicated by red arrows. The diffusive movements of the cation are indicated by green arrows. **Step 1**: In the inactive form of hCHIT1, Asp136 (β4 strand) interacts with Asn208 (β6 strand) by hydrogen bonds. Tyr27 is situated between Asp136 and Asp138 residues. The protonated Asp138 (p*Ka* = ~10) faces Asp136 while unprotonated Glu140 (p*Ka* = 6) forms a hydrogen bond with the adjacent Tyr141. When 'lid' (β3 strand) and 'latch' (β4 strand) loops cooperatively open, the β4 strand slides towards the entrance of the active site cleft (purple arrow) and exposes Asp138 for binding the substrate; **Step 2**: The β4 strand stops when Asp136 forms hydrogen bond with Tyr27. The enzyme becomes active. The substrate, led by the electrostatic 'torch of guidance' effect enters the active site in the default chair conformation. Diffusive movement of M^+ facilitate the rotation of Asp138 towards Glu140 as well as otherwise unfavorable, 180° rotation of the 2-acetamido group of the -1 sugar. Upon substrate binding, M^+ diffuses towards Glu140 (p*Ka* ↑) facilitating proton transfer from Asp138 (p*Ka* ↓). **Step3**: The tight binding to the key residues of the active site cleft induces the boat-like conformation of the -1 sugar. The nitrogen and carbonyl oxygen from the 2-

acetamido group of the -1 sugar form strong hydrogen bonds with the side chains of Asp138 and Tyr212, respectively. The binding substrate pushes the β4 strand in the direction of its momentum (purple arrow). Consequently, Asp138 pushes the hydrogen shared with the nitrogen from the 2-acetamido group in one direction while the carbonyl oxygen is pushed by Tyr212 in the opposite direction. The general acid (Glu140) donates proton to the glycosidic bond between -1 and +1 sugar units, as the hydrogen bond between Asp138 and nitrogen of the 2-acetamido group tightens up. This process in coordinated by the movement of the M^+ from Glu140 (*pKa* ↓) to Asp138 (*pKa* ↑). Pushed by Tyr212, carbonyl oxygen performs a nucleophilic attack on the anomeric carbon, forming the oxazolinium/oxazoline intermediate and in turn breaking the glycosidic bond; **Step 4**: Upon +1 sugar departure, the 'lid' gets released and opens the active site, allowing water molecule to enter. **Step 5**: Deprotonated Glu140 becomes a general base that facilitates the nucleophilic attack of the water molecule on the anomeric carbon. **Step 6**: The breaking intermediate springs when oxazolinium/oxazoline ring opens back into 2-acetamido group and consequently -1 sugar detaches from the active site, while M^+ promotes the proton transfer between Glu140 and Asp138. **Step 7**: Detachment of the -1 sugar triggers the β4 strand to slide backward; **Step 8**: Glu140 and Asp136 again form hydrogen bonds with Tyr141 and Asn208, respectively. M^+ returns above Asp138 facilitating its protonation (*pKa* ↑) and rotation towards Asp136 that closes the catalytic cycle.

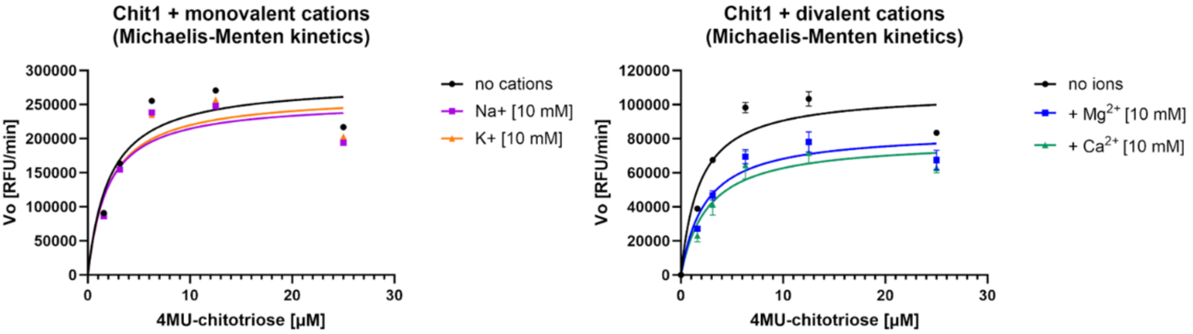

	no ions	Na+ [10 mM]	K+ [10 mM]
Michaelis-Menten			
Best-fit values			
Vmax	283323	255810	265013
Km	2.114	1.926	2.063

	no ions	Mg^{2+} [10 mM]	Ca^{2+} [10 mM]
Michaelis-Menten			
Best-fit values			
Vmax	107233	84496	79218
Km	1.838	2.392	2.634

Figure S8. The influence of the most abundant ions in the human body (Na^+, K^+, Ca^{2+} and Mg^{2+}) on $(GlcNAc)_3$ substrate evaluated by the means of biological assays. Lower values of Michaelis constant (K_M) indicate the tighter substrate binding when Na^+ or K^+ ions are added in the assay. The M^+ requirement for substrate binding with the maximum rate of reaction (V_{max}) is independent of [M^+] indicate the type of Ia (cofactor-like) mechanism of hCHIT1 activation by alkali ions. Note that the ‘no ions’ system was prepared by a desalination process of the enzyme, which may not have been entirely successful. In such a case, the influence of adding ions may be less pronounced. Moreover, hCHIT1 might not respond well to ionic concentrations exceeding that of its natural habitat.

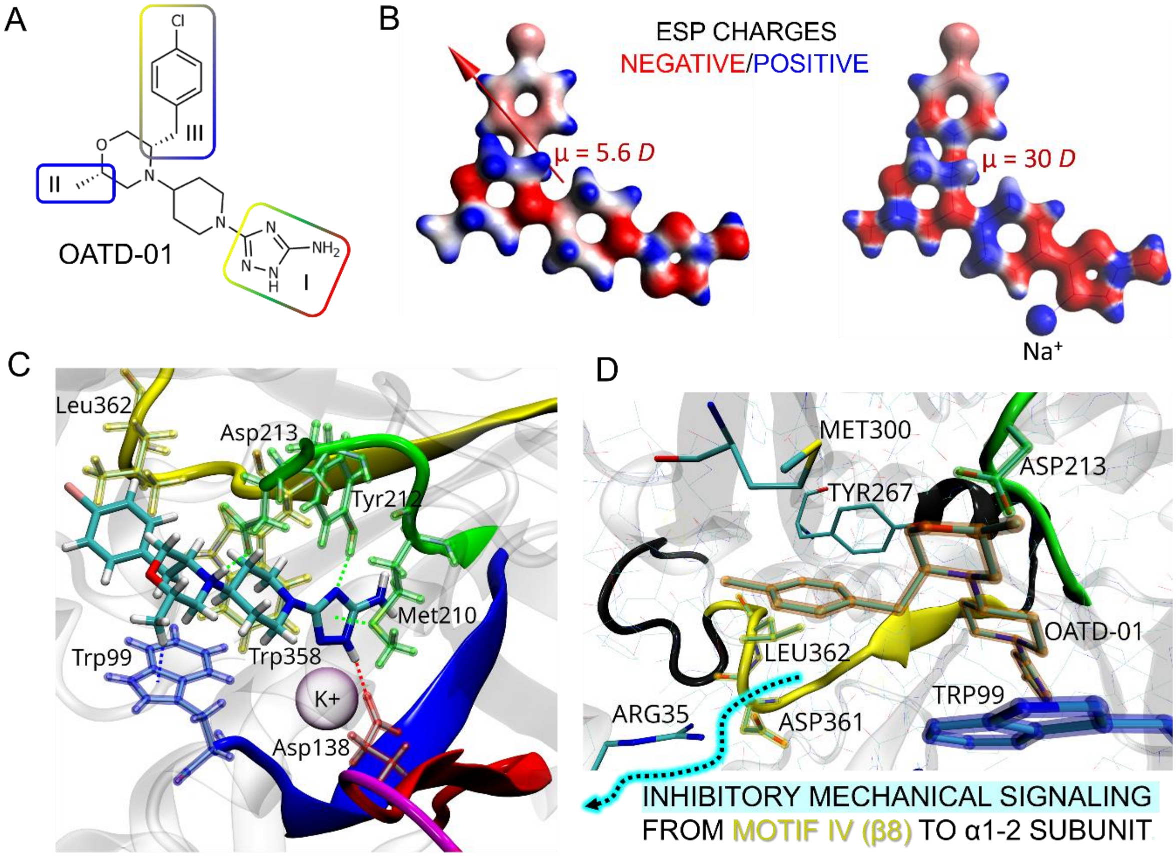

Figure S9. OATD-01 *first-in-class* hCHIT1 inhibitor. **A**) The structural features responsible for the high inhibitory potency against hCHIT1. The color(s) of each frame correspond to the color code of the four conserved motifs in Figure 1A. **B**) The electrostatic potential (ESP) charges mapped on the electron density surface of OATD-01 alone (left) and interacting with sodium cation (right). The red/blue colors correspond to negative/positive values of charge. The dipole moment is represented as a red arrow and rotated with the parallel orientation of the dipole moment of hCHIT1. The dipole moment value drastically increased upon interaction with the cation but did not change direction (not presented). **C**) The binding mode of the OATD-01 electrostatically stabilized in the active site of CHIT1 by potassium ion, based on 6ZE8 PDB entry (Figure S3C). The enzyme is presented as a cartoon while the inhibitor and the key residues are in licorice representations. The OATD-01 inhibitor, by tightly binding the

key residues involved in the catalysis, blocks the reciprocal movement of all four conserved motifs. **D)** The binding mode of the 4-chlorobenzene branch is responsible for the long-range effect of the OATD-01 inhibition. The 4-chlorobenzene moiety is locked inside a hydrophobic sub-pocket made up of residues Met300, Tyr276 and Leu362. The latter is next to the residue Asp361, which makes salt-bridge with Arg35, and therefore promotes optimal flexibility of α1-2 subunit and a correct orientation of the Arg40 residue for docking CBM. The allosteric inhibition of hCHIT1 by OATD-01 prevents the assembly of the catalytic and carbohydrate-binding domains into the immunoglobulin-like heterodimer. The black strand indicates the fragment within the conserved motif IV, which is removed by the most common genetic inactivation of the enzyme. OATD-01 has a comparable effect to this mutation.

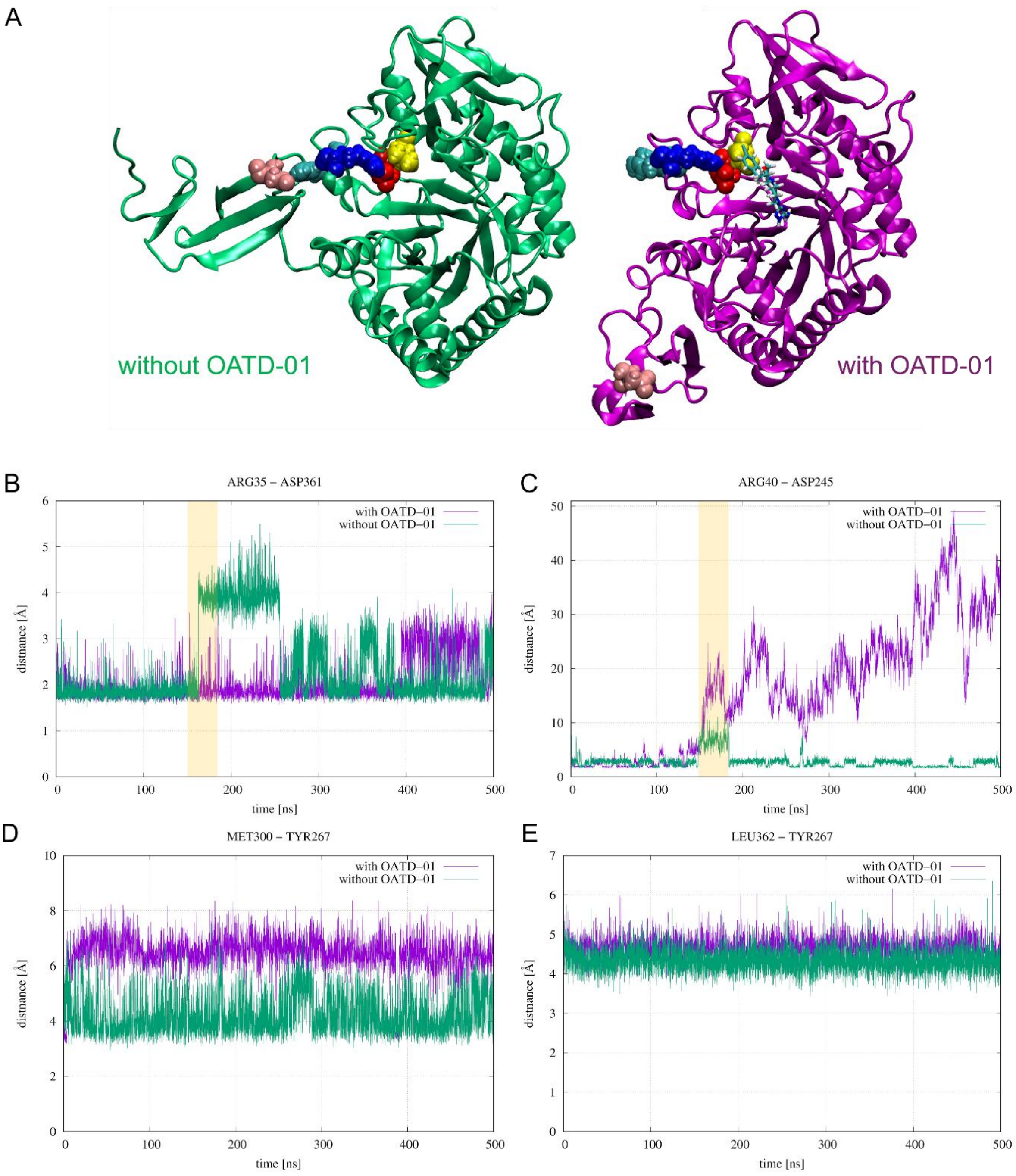

Figure S10. The effect of OATD-01 on the hCHIT1 heterodimer assembly and stability. **A**) Structures without and with OATD-01 ligand after 500 nanosecond MD simulations, colored in green and magenta, respectively. The enzyme is represented as a cartoon, the OATD-01 inhibitor as licorice, and the key amino acids are in vdW representations. Yellow, red, blue, cyan, and pink colors correspond to Leu362, Asp361, Arg35, Arg40 and Asp425 residues, respectively. These amino acids are involved in the inter-subunit mechanical signal

transduction between the catalytic domain (i.e., β8 strand) and CBM through α1-2 subunit. The detailed view of the reciprocal orientation of these resides and OATD-01 is shown in Figure S8C/D**)** The evolution of distances between the residues involved in the inter-subunit mechanical signaling: two salt bridges (Arg35–Asp361 and Arg40–Asp425) and residues involved in the hydrophobic sub-pocket accommodating the 4-chlorobenzene (branch III) of OATD-01 (Met300–Tyr267 and Leu362–Tyr267). OATD-01 exerts a strong interaction with residues Tyr267, Met300 and Leu362, thereby imposing rigidity on β8 strand **B**). The hydrophobic sub-pocket expands **D**/**E**) and tightens the hydrogen bonds between Asp361 and Arg35, which connect β8 strand with the α1-2 subunit. Consequently, the α1-2 structural feature becomes more rigid and induces stress on the adjacent salt bridge between Arg40 and Asp425, which connects the α1-2 and CBM subunits **C**). The Arg40–Asp425 salt bridge breaks, resulting in CBM dissociation. Increased rigidity of the α1-2 subunit, which contains the residue Arg40, prevents CBM from redocking. This change in the dynamic behavior of Arg40 can be observed when comparing the α1-2 regions within the crystal structures from the 4WKA and 6ZE8 PDB entries (*i.e.*, without and with OATD-01 inhibitor).
